# Renin–angiotensin system blockers and susceptibility to COVID-19: an international, open science, cohort analysis

**DOI:** 10.1016/S2589-7500(20)30289-2

**Published:** 2020-12-17

**Authors:** Daniel R Morales, Mitchell M Conover, Seng Chan You, Nicole Pratt, Kristin Kostka, Talita Duarte-Salles, Sergio Fernández-Bertolín, Maria Aragón, Scott L DuVall, Kristine Lynch, Thomas Falconer, Kees van Bochove, Cynthia Sung, Michael E Matheny, Christophe G Lambert, Fredrik Nyberg, Thamir M Alshammari, Andrew E Williams, Rae Woong Park, James Weaver, Anthony G Sena, Martijn J Schuemie, Peter R Rijnbeek, Ross D Williams, Jennifer C E Lane, Albert Prats-Uribe, Lin Zhang, Carlos Areia, Harlan M Krumholz, Daniel Prieto-Alhambra, Patrick B Ryan, George Hripcsak, Marc A Suchard

**Affiliations:** a Division of Population Health and Genomics, University of Dundee, Dundee, UK; b Observational Health Data Analytics, Janssen Research & Development, Titusville, NJ, USA; c Department of Biomedical Informatics, Ajou University School of Medicine, Suwon, South Korea; d Quality Use of Medicines and Pharmacy Research Centre, Clinical and Health Sciences, University of South Australia, Adelaide, SA, Australia; e Real World Solutions, IQVIA, Cambridge, MA, USA; f Fundació Institut Universitari per a la Recerca a l'Atenció Primària de Salut Jordi Gol i Gurina (IDIAPJGol), Barcelona, Spain; g Department of Veterans Affairs, Salt Lake City, UT, USA; h University of Utah School of Medicine, Salt Lake City, UT, USA; i Department of Biomedical Informatics, Columbia University, New York, NY, USA; j The Hyve, Utrecht, Netherlands; k Health Services and Systems Research, Duke-NUS Medical School, Singapore; l Geriatric Research Education and Clinical Care Center, Tennessee Valley Healthcare System VA, Nashville, TN, USA; m Department of Biomedical Informatics, Vanderbilt University Medical Center, Nashville, TN, USA; n Department of Internal Medicine, University of New Mexico Health Sciences Center, Albuquerque, NM, USA; o School of Public Health and Community Medicine, Institute of Medicine, Sahlgrenska Academy, University of Gothenburg, Gothenburg, Sweden; p Medication Safety Research Chair, King Saud University, Riyadh, Saudi Arabia; q Tufts Medical Center, Tufts University, Boston, MA, USA; r Department of Medical Informatics, Erasmus University Medical Center, Rotterdam, Netherlands; s Centre for Statistics in Medicine, Nuffield Department of Orthopaedics, Rheumatology and Musculoskeletal Sciences, University of Oxford, Oxford, UK; t Nuffield Department of Clinical Neurosciences, University of Oxford, Oxford, UK; u School of Public Health, Peking Union Medical College and Chinese Academy of Medical Sciences, Beijing, China; v Melbourne School of Public Health, The University of Melbourne, VIC, Australia; w Section of Cardiovascular Medicine, Department of Medicine, Yale University, New Haven, CT, USA; x Department of Biostatistics, Fielding School of Public Health, and Department of Computational Medicine, David Geffen School of Medicine at UCLA, University of California, Los Angeles, Los Angeles, CA, USA

## Abstract

**Background:**

Angiotensin-converting enzyme inhibitors (ACEIs) and angiotensin receptor blockers (ARBs) have been postulated to affect susceptibility to COVID-19. Observational studies so far have lacked rigorous ascertainment adjustment and international generalisability. We aimed to determine whether use of ACEIs or ARBs is associated with an increased susceptibility to COVID-19 in patients with hypertension.

**Methods:**

In this international, open science, cohort analysis, we used electronic health records from Spain (Information Systems for Research in Primary Care [SIDIAP]) and the USA (Columbia University Irving Medical Center data warehouse [CUIMC] and Department of Veterans Affairs Observational Medical Outcomes Partnership [VA-OMOP]) to identify patients aged 18 years or older with at least one prescription for ACEIs and ARBs (target cohort) or calcium channel blockers (CCBs) and thiazide or thiazide-like diuretics (THZs; comparator cohort) between Nov 1, 2019, and Jan 31, 2020. Users were defined separately as receiving either monotherapy with these four drug classes, or monotherapy or combination therapy (combination use) with other antihypertensive medications. We assessed four outcomes: COVID-19 diagnosis; hospital admission with COVID-19; hospital admission with pneumonia; and hospital admission with pneumonia, acute respiratory distress syndrome, acute kidney injury, or sepsis. We built large-scale propensity score methods derived through a data-driven approach and negative control experiments across ten pairwise comparisons, with results meta-analysed to generate 1280 study effects. For each study effect, we did negative control outcome experiments using a possible 123 controls identified through a data-rich algorithm. This process used a set of predefined baseline patient characteristics to provide the most accurate prediction of treatment and balance among patient cohorts across characteristics. The study is registered with the EU Post-Authorisation Studies register, EUPAS35296.

**Findings:**

Among 1 355 349 antihypertensive users (363 785 ACEI or ARB monotherapy users, 248 915 CCB or THZ monotherapy users, 711 799 ACEI or ARB combination users, and 473 076 CCB or THZ combination users) included in analyses, no association was observed between COVID-19 diagnosis and exposure to ACEI or ARB monotherapy versus CCB or THZ monotherapy (calibrated hazard ratio [HR] 0·98, 95% CI 0·84–1·14) or combination use exposure (1·01, 0·90–1·15). ACEIs alone similarly showed no relative risk difference when compared with CCB or THZ monotherapy (HR 0·91, 95% CI 0·68–1·21; with heterogeneity of >40%) or combination use (0·95, 0·83–1·07). Directly comparing ACEIs with ARBs demonstrated a moderately lower risk with ACEIs, which was significant with combination use (HR 0·88, 95% CI 0·79–0·99) and non-significant for monotherapy (0·85, 0·69–1·05). We observed no significant difference between drug classes for risk of hospital admission with COVID-19, hospital admission with pneumonia, or hospital admission with pneumonia, acute respiratory distress syndrome, acute kidney injury, or sepsis across all comparisons.

**Interpretation:**

No clinically significant increased risk of COVID-19 diagnosis or hospital admission-related outcomes associated with ACEI or ARB use was observed, suggesting users should not discontinue or change their treatment to decrease their risk of COVID-19.

**Funding:**

Wellcome Trust, UK National Institute for Health Research, US National Institutes of Health, US Department of Veterans Affairs, Janssen Research & Development, IQVIA, South Korean Ministry of Health and Welfare Republic, Australian National Health and Medical Research Council, and European Health Data and Evidence Network.

## Introduction

People with cardiovascular diseases and hypertension are more likely to develop severe complications from COVID-19, which is caused by severe acute respiratory syndrome coronavirus 2 (SARS-CoV-2), including hospital admission and death.^1^, ^2^, ^3^ Speculatively, angiotensin-converting enzyme inhibitors (ACEIs) and angiotensin receptor blockers (ARBs), both of which block the renin–angiotensin system (RAS), might affect people's susceptibility to COVID-19 and worsen its severity. Driving this hypothesis is the mechanism by which SARS-CoV-2 enters human cells: by binding to the membrane-bound aminopeptidase angiotensin-converting enzyme 2 (ACE2), the expression of which might be altered by chronic exposure to RAS therapy.^4^, ^5^, ^6^, ^7^, ^8^, ^9^, ^10^, ^11^, ^12^, ^13^ Speculation about the effects of RAS therapy on susceptibility to and severity of COVID-19 has generated substantial public health concerns, resulting in the release of statements from health regulatory agencies and clinical societies advocating that, in the absence of direct evidence of harm with COVID-19, these medicines should not be discontinued.^14^, ^15^ However, inconsistencies in recommendations have emerged, with suggestions that users of these medicines should be monitored closely. Unlike clinical trials that are being proposed to investigate the withdrawal of ACEIs and ARBs among symptomatic patients with COVID-19, it is less likely that large-scale, population-based trials assessing susceptibility to COVID-19 among users of RAS therapy will be done in a timely manner.^16^, ^17^ Withholding these medicines, however, might result in worse cardiovascular outcomes, with some studies reporting an increased risk of myocardial injury resulting from illness with COVID-19.^1^

**Research in context**

**Evidence before this study**

We systematically searched PubMed, Embase, clinical trial registries, and preprint servers for research articles published from inception until March 27, 2020. No language restriction was applied. We found no investigations of the real-world safety of first-line antihypertensive medications involving COVID-19 diagnoses. Studies examining the association between renin–angiotensin system inhibitor use and COVID-19 susceptibility have since been published that report no COVID-19 risk or a lower risk associated with use of angiotensin-converting enzyme inhibitors (ACEIs) and angiotensin receptor blockers (ARBs). However, these studies have small sample sizes, limited confounder adjustment, or methodological limitations such as immortal time bias and collider bias. We identified one small study directly comparing the effects of ACEI versus ARB use among symptomatic patients with COVID-19 that showed no difference in patient outcomes.

**Added value of this study**

This study comprehensively evaluates the safety of ACEIs and ARBs in COVID-19 by examining a large number of different comparisons using state-of-the-art methods to control for residual confounding and bias across a distributed network. Our study shows similar results across three databases from two countries. ACEI and ARB use does not confer increased risk of: COVID-19 diagnosis; hospital admission with COVID-19; hospital admission with pneumonia; or hospital admission with pneumonia, acute respiratory distress syndrome, acute kidney injury, or sepsis compared with people taking calcium channel blockers and thiazide or thiazide-like diuretics.

**Implications of all the available evidence**

Use of ACEIs and ARBs does not affect COVID-19 susceptibility risk, and these results are in keeping with medicines regulatory and clinical society recommendations that patients should not alter their treatment with these medicines to reduce their COVID-19 risk.

Several studies have emerged examining this conundrum. Although informative, they have had small sample sizes, limited confounder adjustment, used heterogeneous comparisons, or had methodological limitations, including immortal time bias and collider bias.^18^, ^19^, ^20^, ^21^ For example, comparing the risk of COVID-19 among users of ACEIs or ARBs with an unexposed control population can result in recruitment of non-comparable participants, confounding by indication, and the absence of a clear index date for when follow-up should start, all of which can induce bias. Reliable evidence should also be replicable, generalisable, and robust. To draw strong conclusions from observational studies, it is essential that consistent findings are produced from transparent, well designed analyses across multiple populations and data capture processes to ensure that any associations are not due to systematic error or applicable only in narrow contexts. We aimed to determine whether exposure to ACEIs or ARBs is associated with an increased susceptibility to COVID-19 among patients with hypertension.

## Methods

### Study design

We did a systematic and comprehensive federated active-comparator cohort study facilitated by a common data model. The protocol for the International Covid-ACE Receptor Inhibition Utilization and Safety (ICARIUS) studies was drafted and carried out by an international team of clinical, academic, government, and industry stakeholders through the Observational Health Data Sciences and Informatics (OHDSI) network.^22^

### Data sources

We identified patients in routinely collected electronic health records (EHRs) and claims data from the USA and Spain. All data sources had been mapped to the Observational Medical Outcomes Partnership (OMOP) common data model (version 5).^23^ Two particular benefits of this standardisation are that contributing centres can participate in distributed network analyses without needing to share patient-level information and that data provenance can be ensured while applying a common analytical code across all data sources in a consistent manner. The data sources included the Columbia University Irving Medical Center (New York, NY, USA) data warehouse (CUIMC), the Information Systems for Research in Primary Care (SIDIAP) database, and the US Department of Veterans Affairs OMOP (VA-OMOP) database. CUIMC EHRs contain data, including clinical diagnoses, prescriptions, laboratory tests, demographics, and COVID-19 tests and diagnosis, from approximately 6 million cumulative patients from the New York-Presbyterian Hospital and Columbia University Irving Medical Center in the USA.^24^ SIDIAP covers approximately 80% of the population of Catalonia (Spain), with approximately 6 million patients, and contains data collected since 2006 from general practice EHRs linked to hospital admissions, with information on diagnoses, prescriptions, laboratory tests, and lifestyle and sociodemographics, and the central database of RT-PCR COVID-19 tests.^25^ VA-OMOP covers approximately 12 million patients from 170 medical centres across the USA and includes administrative, clinical, laboratory, and pharmacy data repositories that are linked using unique patient identifiers.^26^

All data partners received institution review board approval or waiver in accordance with their institutional governance guidelines. Use of SIDIAP was approved by the Clinical Research Ethics Committee of the Institut Universitari d'Investigació en Atenció Primària Jordi Gol (Barcelona, Spain; project code 20/070-PCV). Use of VA-OMOP was reviewed by the US Department of Veterans Affairs Central Institutional Review Board and was determined to meet the criteria for exemption under Exemption Category 4(3) and approved the request for Waiver of HIPAA Authorization. Use of CUIMC was approved by the Columbia University Institutional Review Board as an OHDSI network study (AAAO7805).

### Cohort eligibility, study period, and follow-up

**Figure 1:**
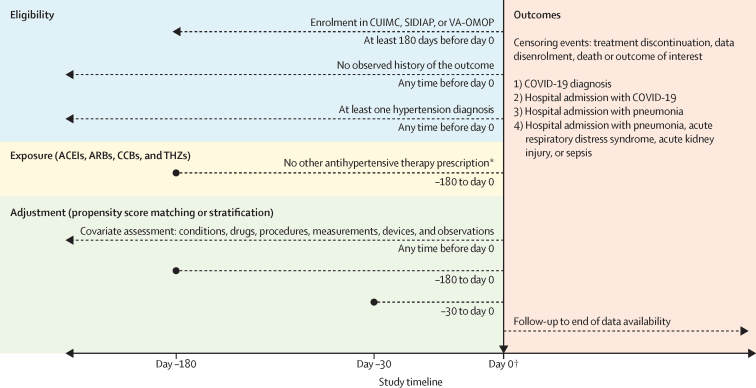
ICARIUS susceptibility study design We highlight eligibility criteria, exposure definitions, adjustment strategies, index date specification (day 0; horizontal black arrow), and outcome definitions and time at risk. Exposure involves prescriptions to drugs with RxNorm ingredients that map to the first-line antihypertensive drug classes: ACEIs, ARBs, CCBs, and THZs. ACEI=angiotensin-converting enzyme inhibitor. ARB=angiotensin receptor blocker. CCB=calcium channel blocker. CUIMC=Columbia University Irving Medical Center data warehouse. ICARIUS=International Covid-19 ACE Receptor Inhibition Utilization and Safety. SIDIAP=Information Systems for Research in Primary Care. THZ=thiazide or thiazide-like diuretic. VA-OMOP=US Department of Veterans Affairs Observational Medical Outcomes Partnership. *For monotherapy analysis only; other antihypertensive therapy included rate-limiting CCBs, diuretics, and β blockers. †Day 0 is the most recent observed prescription for target or drug comparator between Nov 1, 2019, and Jan 31, 2020.

Each cohort consisted of adults aged 18 years or older who received at least one outpatient prescription for an ACEI, ARB, calcium channel blocker (CCB), or thiazide or thiazide-like diuretic (THZ) between Nov 1, 2019, and Jan 31, 2020. The index date (ie, start of cohort follow-up) was set as the date of the last prescription in this time window (figure 1). We required patients to be observable in their data source for at least 180 days before the index date and have a diagnostic code for hypertension at any point before or including the index date. Cohort exit was the earliest of the occurrence of an outcome; the end of exposure, death, loss or deregistration from the database; or date of last data collection.

### Exposures

The exposures of interest were four first-line antihypertensive drug classes: ACEIs, ARBs, CCBs, or THZs. Users were defined separately as receiving either (1) monotherapy or (2) monotherapy or combination therapy (combination use) with other antihypertensive medications. Our primary comparison examined outpatient exposure to RAS blockers (ACEI or ARBs) with exposure to CCBs or THZs (included as active comparators). Further investigation compared class exposure to ACEIs with exposure to ARBs separately, and individual classes to various active comparators, leading to ten different target–comparator pairings for monotherapy and combination use each, as listed in the appendix (pp 2–13). For patients on monotherapy, we required the absence of any other antihypertensive treatment between –180 days and 0 days before the index date. We defined continuous drug exposures from the start of follow-up by grouping sequential prescriptions that had at most a 30-day gap between prescriptions, and defined the end of exposure as the end of the last prescription's drug supply in such a sequence.

### Outcomes

We investigated four COVID-19-related outcomes: COVID-19 diagnosis; hospital admission with COVID-19; hospital admission with pneumonia; and hospital admission with pneumonia, acute respiratory distress syndrome, acute kidney injury, or sepsis. Positive COVID-19 PCR test results or SNOMED diagnostic codes defined COVID-19 status. COVID-19 antibody tests were not available when we did the study. The full details of the participant cohorts and outcome definitions used can be found in the protocol.

### Statistical analysis

We undertook this study using all patients who met the eligibility criteria within each database. Therefore, we did not calculate the sample size a priori; instead, we provide a minimum detectable rate ratio (MDRR) for each target–comparator–outcome triplet across each data source.^27^

To adjust for potential measured confounding and to improve the balance between comparison cohorts, we built large-scale propensity score models for each comparison and data source using a consistent data-driven process through regularised regression.^27^, ^28^ This process used a large set of predefined baseline patient characteristics (including age, sex, race [US data], and other demographics) and previous conditions, drug exposures, procedures, and health service use behaviours to provide the most accurate prediction of treatment and balance patient cohorts across many characteristics. For computational efficiency, we excluded all features that occurred in fewer than 0·1% of patients within the target and comparator cohorts before propensity score model fitting.

In separate analyses, we stratified patients into five propensity score quintiles or variable-ratio matched patients by propensity, and used Cox proportional hazards models to estimate hazard ratios (HRs) between alternative target and comparator treatments for the risk of each outcome in each data source. The regression conditioned on the propensity score strata or matching units, with treatment allocation as the sole explanatory variable. We aggregated HR estimates across data sources to produce meta-analytic estimates using a random-effects meta-analysis.^29^ We only included estimates that passed propensity score diagnostics in the main meta-analysis, with meta-analytic estimates based on all data sources provided as a sensitivity analysis. For both monotherapy and combination use of the ACEI, ARB, CCB, THZ, ACEI or ARB, and CCB or THZ class groups (ten pairwise comparisons) to study four outcomes in three data sources (plus one meta-analysis) using two propensity score-adjustment approaches, we generated 1280 study effects.

Residual study bias from unmeasured and systematic sources often remains in observational studies even after controlling for measured confounding through propensity score adjustment.^30^, ^31^ For each study effect, we did negative control outcome experiments, for which the null hypothesis of no effect is believed to be true, using a possible 123 controls identified through a data-rich algorithm and validated in a previous antihypertensive comparative study.^32^, ^33^ Using the empirical null distributions from these experiments, we calibrated each study effect HR estimate, its 95% CI, and the p value to reject the null hypothesis of no differential effect.^34^ We declared an HR as significantly different from no effect when its calibrated p value was less than 0·05 without correcting for multiple testing.

These study diagnostics were presented to clinicians and epidemiologists who were masked to the HRs generated by the models for evaluation to provide an unbiased assessment of their validity. The suite of diagnostics included the MDRR, the preference score (a transformation of the propensity score that adjusts for prevalence differences between populations) distributions to evaluate empirical equipoise and population generalisability,^35^ extensive patient characteristics to evaluate cohort balance before and after adjustment using the propensity score, negative control calibration plots to assess residual bias, and Kaplan-Meier plots to examine HR proportionality assumptions. We defined target and comparator cohorts to stand in empirical equipoise if the majority of patients in both carried preference scores between 0·3 and 0·7 and to achieve sufficient balance if all after-adjustment baseline characteristics returned an absolute standardised mean differences of less than 0·1.^36^ Heterogeneity following meta-analysis was defined by an *I*^2^ value of more than 40%.

We did this study using the open-source OHDSI CohortMethod R package with large-scale analytics made possible through the Cyclops R package.^32^ The prespecified ICARIUS protocol and start-to-finish open and executable source code are available online. To promote transparency and facilitate sharing and exploration of the complete result set, an interactive web application provides study diagnostics and results for all study effects.

The study is registered with the EU Post-Authorisation Studies register, EUPAS35296.

### Role of the funding source

The funders of the study had no role in study design, study execution, data collection, data interpretation, writing of the report, or the decision to submit for publication, and all authors share responsibility for the decision to submit this work for publication. This was a federated data analysis, and no single author had access to all of the underlying data. TD-S, SF-B, and MA had access to the data in SIDIAP; TF and GH had access to the data in CUIMC; and SLD and KL had access to the data in VA-OMOP. DRM, MMC, SCY, GH, and MAS had full access to all aggregate results.

## Results

Among 1 355 349 patients included in the analysis, 363 785 patients with hypertension who received ACEI or ARB monotherapy were compared with 248 915 patients who received CCB or THZ monotherapy, contributing 121 213 person-years and 81 261 person-years of follow-up, respectively. The overall incidence of COVID-19 diagnosis was 5·6 per 1000 person-years among patients who received ACEI or ARB monotherapy compared with 4·8 per 1000 person-years among those who received CCB or THZ monotherapy, although incidence rates varied by data source.

Corresponding patient cohort size and diagnosis incidence rates were 268 711 and 5·6 per 1000 person-years for ACEI (alone) monotherapy users and 92 485 and 5·1 per 1000 person-years for ARB (alone) monotherapy users. Cohorts for combination users (as monotherapy or combination therapy) were as large as 711 799 for ACEI or ARB users and 473 076 for CCB or THZ users.

The aggregated patient cohort size, follow-up duration, incidences of each COVID-19-related outcome, and MDRR for each drug comparison and database are shown in table 1. The appendix (pp 2–13) provides further cohort size and outcome event information for all ten pairwise cohort comparisons across all four outcomes.

**Table 1:** Populations and COVID-19 diagnoses for ACEI, ARB, CCB, and THZ monotherapy and combination user cohorts

		**Patients**		**Time, years**		**Events**		**MDRR**
		Target cohort	Comparator cohort	Target cohort	Comparator cohort	Target cohort	Comparator cohort	
**ACEIs or ARBs *vs* CCBs or THZs**								
Monotherapy								
	SIDIAP	37 796	14 003	10 239	3 780	500	184	1·27
	VA-OMOP	320 450	229 063	110 380	76 856	145	183	1·37
	CUIMC	5539	5849	594	625	28	24	2·18
Combination use								
	SIDIAP	45 239	19 007	12 264	5175	627	250	1·23
	VA-OMOP	656 274	443 061	228 678	150 755	345	335	1·24
	CUIMC	10 286	11 008	1128	1185	59	58	1·68
**ACEIs *vs* CCBs or THZs**								
Monotherapy								
	SIDIAP	30 787	14 003	8293	3780	398	184	1·28
	VA-OMOP	235 348	229 063	80 760	76 856	96	183	1·40
	CUIMC	2576	5849	277	625	10	24	2·84
Combination use								
	SIDIAP	36 323	29 239	9803	7941	485	399	1·21
	VA-OMOP	457 557	639 500	158 721	221 239	218	494	1·24
	CUIMC	4811	16 302	511	1754	18	83	1·94
**ARBs *vs* CCBs or THZs**								
Monotherapy								
	SIDIAP	6753	14 003	1815	3780	95	184	1·43
	VA-OMOP	82 872	229 063	28 689	76 856	46	183	1·52
	CUIMC	2860	5849	301	625	17	24	2·54
Combination use								
	SIDIAP	9194	39 427	2457	10 714	137	519	1·32
	VA-OMOP	201 503	854 224	70 267	295 986	127	574	1·31
	CUIMC	5669	14 271	629	1533	41	77	1·77
**ACEIs *vs* ARBs**								
Monotherapy								
	SIDIAP	30 787	6753	8293	1815	398	95	1·39
	VA-OMOP	235 348	82 872	80 760	28 689	96	46	1·71
	CUIMC	2576	2860	277	301	10	17	2·94
Combination use								
	SIDIAP	56 465	19 148	15 333	5176	758	283	1·22
	VA-OMOP	865 931	395 156	303 491	140 071	441	282	1·25
	CUIMC	7880	10 769	826	1179	39	66	1·74

ACEI=angiotensin-converting enzyme inhibitor. ARB=angiotensin receptor blocker. CCB=calcium channel blocker. CUIMC=Columbia University Irving Medical Center data warehouse. MDRR=minimum detectable risk ratio. SIDIAP=Information Systems for Research in Primary Care. THZ=thiazide or thiazide-like diuretic. VA-OMOP=US Department of Veterans Affairs Observational Medical Outcomes Partnership.

Baseline characteristics of ACEI or ARB monotherapy users compared with CCB or THZ monotherapy users, before and after propensity score stratification, are shown in Table 2, Table 3, Table 4. There were baseline differences in sex, hyperlipidaemia, diabetes, renal impairment, heart failure, heart disease, atrial fibrillation, drugs for diabetes, lipid-modifying agents, antithrombotics, antacids, opioids, and race that varied by data source. Further information on the population characteristics for each cohort comparison and design evaluated for each data source are shown in the appendix (pp 14–162), one for each of the 60 comparisons across data sources.

**Table 2:** Baseline patient characteristics for prevalent use of ACEI or ARB (target) and CCB or THZ (comparator) monotherapy in the SIDIAP data source

	**Before stratification**			**After stratification**		
	Target SIDIAP cohort (n=37 796)	Comparator SIDIAP cohort (n=14 003)	Standardised mean difference	Target SIDIAP cohort (n=37 796)	Comparator SIDIAP cohort (n=14 003)	Standardised mean difference
**Age group, years**						
<25	0·1%	0·1%	0·00	0·1%	0·1%	−0·01
25–29	0·2%	0·2%	0·00	0·2%	0·2%	−0·01
30–34	0·6%	0·6%	0·01	0·6%	0·6%	0·00
35–39	1·6%	1·0%	0·04	1·4%	1·3%	0·01
40–44	3·8%	2·3%	0·08	3·4%	3·3%	0·01
45–49	7·0%	5·0%	0·08	6·4%	6·5	0·00
50–54	10·4%	7·9%	0·09	9·6%	9·9%	−0·01
55–59	12·5%	10·2%	0·07	11·8%	12·1%	−0·01
60–64	13·7%	12·5%	0·03	13·4%	13·2%	0·00
65–69	13·4%	13·4%	0·00	13·5%	13·2%	0·01
70–74	12·9%	14·6%	−0·05	13·4%	13·4%	0·00
75–79	9·8%	12·7%	−0·09	10·7%	10·5%	0·01
80–84	6·8%	9·0%	−0·08	7·4%	7·4%	0·00
85–89	4·7%	6·5%	−0·08	5·2%	5·2%	0·00
90–94	2·1%	2·9%	−0·06	2·3%	2·3%	0·00
≥95	0·5%	0·8%	−0·04	0·5%	0·7%	−0·02
**Sex**						
Female	46·8%	53·0%	−0·12	48·9%	47·8%	0·02
Male	53·2%	47·0%	−0·12	51·1%	52·2%	0·02
**Medical history: general**						
Acute respiratory disease	8·6%	8·4%	0·01	8·6%	8·5%	0·00
Attention deficit hyperactivity disorder	0·1%	0·1%	0	0·1%	0·1%	0·00
Chronic liver disease	1·5%	1·7%	−0·02	1·6%	1·7%	−0·01
Chronic obstructive lung disease	6·0%	7·0%	−0·04	6·2%	6·3%	0·00
Crohn's disease	0·2%	0·2%	0	0·2%	0·2%	0·01
Dementia	2·3%	2·7%	−0·03	2·5%	2·3%	0·01
Depressive disorder	13·6%	14·8%	−0·04	14·0%	13·7%	0·01
Diabetes	19·9%	22·8%	−0·07	20·7%	20·4%	0·01
Gastro-oesophageal reflux disease	10·0%	11·5%	−0·05	10·4%	10·4%	0·00
Gastrointestinal haemorrhage	0·7%	0·8%	−0·01	0·7%	0·7%	−0·01
HIV infection	0·5%	0·4%	0·02	0·5%	0·4%	0·01
Hyperlipidaemia	26·7%	27·9%	−0·03	27·0%	26·9%	0·00
Hypertensive disorder	99·2%	99·3%	−0·01	99·2%	99·3%	−0·01
Lesion of liver	0·8%	1·0%	−0·03	0·8%	0·9%	−0·01
Obesity	34·6%	37·5%	−0·06	35·5%	35·6%	0·00
Osteoarthritis	27·9%	33·2%	−0·12	29·6%	28·9%	0·01
Pneumonia	0·7%	0·6%	0·01	0·7%	0·6%	0·02
Psoriasis	3·7%	3·7%	0·00	3·7%	3·7%	0·00
Renal impairment	8·1%	13·8%	−0·18	9·4%	10·0%	−0·02
Rheumatoid arthritis	0·4%	0·5%	0·00	0·5%	0·4%	0·01
Schizophrenia	0·5%	0·5%	0·00	0·5%	0·5%	−0·01
Ulcerative colitis	0·4%	0·5%	−0·01	0·4%	0·4%	0·00
Urinary tract infectious disease	4·9%	5·3%	−0·02	5·1%	4·9%	0·01
Viral hepatitis C	1·2%	1·2%	0·00	1·2%	1·2%	0·00
Visual system disorder	36·5%	42·0%	−0·11	38·1%	37·5%	0·01
Atrial fibrillation	3·8%	4·6%	−0·04	4·1%	3·9%	0·01
Cerebrovascular disease	2·1%	2·4%	−0·02	2·2%	2·0%	0·01
Coronary arteriosclerosis	0	0	0·01	0	0	0·01
Heart disease	20·7%	24·9%	−0·10	21·8%	21·8%	0·00
Heart failure	1·7%	2·1%	−0·03	1·8%	1·7%	0·00
Ischaemic heart disease	3·9%	4·7%	−0·04	4·1%	4·0%	0·01
Peripheral vascular disease	2·7%	4·3%	−0·08	3·1%	3·2%	−0·01
Pulmonary embolism	0·5%	0·6%	−0·01	0·6%	0·5%	0·01
Venous thrombosis	1·0%	1·1%	−0·01	1·1%	0·9%	0·01
**Medical history: neoplasms**						
Haematological neoplasm	0·6%	0·7%	−0·01	0·6%	0·6%	0·00
Malignant lymphoma	0·4%	0·4%	−0·01	0·4%	0·4%	0·00
Malignant neoplasm of anorectum	0·4%	0·4%	−0·01	0·4%	0·4%	0·00
Malignant neoplastic disease	13·0%	15·3%	−0·06	13·6%	13·6%	0·00
Malignant tumour of breast	2·0%	2·0%	0·00	2·1%	1·8%	0·02
Malignant tumour of colon	1·4%	1·5%	−0·01	1·5%	1·4%	0·01
Malignant tumour of lung	0·1%	0·2%	−0·01	0·1%	0·1%	−0·01
Malignant tumour of urinary bladder	1·0%	1·4%	−0·03	1·1%	1·1%	0·00
Primary malignant neoplasm of prostate	2·0%	2·2%	−0·01	2·1%	2·2%	0·00
**Medication use**						
Antibacterials for systemic use	17·7%	18·8%	−0·03	18·0%	17·8%	0·00
Antidepressants	16·9%	18·1%	−0·03	17·3%	17·0%	0·01
Antiepileptics	7·0%	8·0%	−0·04	7·3%	7·2%	0·00
Anti-inflammatory and antirheumatic products	21·9%	20·9%	0·03	21·7%	21·3%	0·01
Antineoplastic agents	0·5%	0·6%	−0·01	0·6%	0·5%	0·01
Antipsoriatics	0·7%	1·0%	−0·04	0·7%	0·8%	−0·01
Antithrombotic agents	18·2%	22·3%	−0·10	19·4%	19·1%	0·01
Drugs for acid-related disorders	23·0%	28·1%	−0·12	24·5%	24·0%	0·01
Drugs for obstructive airway diseases	7·0%	7·9%	−0·03	7·3%	7·1%	0·00
Drugs used in diabetes	15·9%	18·2%	−0·06	16·6%	16·2%	0·01
Immunosuppressants	1·3%	2·4%	−0·08	1·5%	1·8%	−0·02
Lipid-modifying agents	29·8%	32·9%	−0·07	30·7%	30·7%	0·00
Opioids	9·2%	11·4%	−0·07	9·8%	9·8%	0·00
Psycholeptics	26·5%	29·2%	−0·06	27·4%	26·8%	0·01
Psychostimulants, agents used for attention deficit hyperactivity disorder, and nootropics	1·2%	1·4%	−0·02	1·3%	1·2%	0·01
**Race**						
American Indian or Alaska Native	..	..	..	..	..	..
Asian	..	..	..	..	..	..
Black or African American	..	..	..	..	..	..
Native Hawaiian or other Pacific Islander	..	..	..	..	..	..
White	..	..	..	..	..	..
Other or unknown	..	..	..	..	..	..
**Ethnicity**						
Hispanic or Latino	..	..	..	..	..	..
Not Hispanic or Latino	..	..	..	..	..	..

We report the proportion of selected baseline characteristics and standardised mean difference among ACEI, ARB, CCB, and THZ users before and after propensity score stratification. Less extreme standard differences through stratification suggest improved balance between patient cohorts through propensity score adjustment. ACEI=angiotensin-converting enzyme inhibitor. ARB=angiotensin receptor blocker. CCB=calcium channel blocker. SIDIAP=Information Systems for Research in Primary Care. THZ=thiazide or thiazide-like diuretic.

**Table 3:** Baseline patient characteristics for prevalent use of ACEI or ARB (target) and CCB or THZ (comparator) monotherapy in the VA-OMOP data source

	**Before stratification**			**After stratification**		
	Target VA-OMOP cohort (n=320 450)	Comparator cohort (n=229 063)	Standardised mean difference	Target VA-OMOP cohort (n=320 450)	Comparator cohort (n=229 063)	Standardised mean difference
**Age group, years**						
<25	..	..	..	..	..	..
25–29	0·2%	0·2%	0·00	0·2%	0·2%	0·00
30–34	0·8%	0·9%	−0·01	0·8%	0·8%	0·00
35–39	1·9%	2·0%	−0·01	2·0%	1·9%	0·01
40–44	2·7%	2·8%	−0·01	2·7%	2·6%	0·01
45–49	4·6%	4·4%	0·01	4·6%	4·4%	0·01
50–54	7·2%	7·1%	0·01	7·2%	6·9%	0·01
55–59	9·5%	10·0%	−0·02	9·6%	9·4%	0·01
60–64	12·0%	13·6%	−0·05	12·6%	12·4%	0·00
65–69	14·5%	14·8%	−0·01	14·5%	14·6%	0·00
70–74	25·2%	22·2%	0·07	23·9%	24·2%	−0·01
75–79	10·4%	9·5%	0·03	10·1%	10·4%	−0·01
80–84	5·2%	5·1%	0·00	5·2%	5·4%	−0·01
85–89	3·9%	4·4%	−0·03	4·2%	4·3%	−0·01
90–94	1·5%	2·1%	−0·04	1·8%	1·9%	−0·01
≥95	0·4%	0·7%	−0·04	0·5%	0·6%	0·00
**Sex**						
Female	5·2%	9·0%	−0·15	6·4%	6·8%	−0·02
Male	94·8%	91·0%	−0·15	93·6%	93·2%	−0·02
**Medical history: general**						
Acute respiratory disease	4·3%	5·0%	−0·04	4·6%	4·6%	0·00
Attention deficit hyperactivity disorder	0·7%	0·6%	0·02	0·7%	0·7%	0·00
Chronic liver disease	1·6%	2·5%	−0·06	1·9%	2·0%	−0·01
Chronic obstructive lung disease	7·7%	9·3%	−0·06	8·4%	8·6%	−0·01
Crohn's disease	0·2%	0·2%	−0·01	0·2%	0·2%	0·00
Dementia	2·0%	2·4%	−0·03	2·2%	2·3%	0·00
Depressive disorder	16·7%	17·7%	−0·03	17·1%	17·0%	0·00
Diabetes	37·8%	16·3%	0·50	29·5%	28·3%	0·03
Gastro-oesophageal reflux disease	13·9%	14·2%	−0·01	14·1%	14·3%	−0·01
Gastrointestinal haemorrhage	0·6%	0·8%	−0·02	0·7%	0·8%	−0·01
HIV infection	0·5%	0·8%	−0·03	0·6%	0·6%	0·00
Hyperlipidaemia	48·2%	39·6%	0·17	44·7%	44·2%	0·01
Hypertensive disorder	68·4%	71·1%	−0·06	69·4%	70·0%	−0·01
Lesion of liver	1·2%	1·6%	−0·03	1·3%	1·4%	−0·01
Obesity	12·7%	10·6%	0·06	11·9%	11·6%	0·01
Osteoarthritis	14·5%	15·8%	−0·04	15·0%	15·1%	0·00
Pneumonia	0·7%	0·9%	−0·03	0·8%	0·8%	−0·01
Psoriasis	1·2%	1·0%	0·02	1·1%	1·1%	0·00
Renal impairment	6·1%	7·1%	−0·04	6·7%	7·0%	−0·01
Rheumatoid arthritis	0·7%	0·8%	0·00	0·8%	0·7%	0·00
Schizophrenia	0·8%	1·2%	−0·03	1·0%	1·0%	−0·01
Ulcerative colitis	0·3%	0·3%	0·00	0·3%	0·3%	0·00
Urinary tract infectious disease	1·3%	1·5%	−0·02	1·4%	1·4%	0·00
Viral hepatitis C	1·0%	2·0%	−0·08	1·3%	1·5%	−0·01
Visual system disorder	28·5%	27·7%	0·02	28·2%	28·1%	0·00
Atrial fibrillation	2·8%	2·3%	0·04	2·7%	2·8%	−0·01
Cerebrovascular disease	2·3%	2·3%	0·00	2·3%	2·5%	−0·01
Coronary arteriosclerosis	6·4%	4·4%	0·09	5·8%	5·8%	0·00
Heart disease	14·0%	11·9%	0·06	13·3%	13·6%	−0·01
Heart failure	1·1%	0·8%	0·03	1·0%	0·9%	0·01
Ischaemic heart disease	1·9%	1·5%	0·03	1·7%	1·8%	0·00
Peripheral vascular disease	2·7%	2·5%	0·01	2·7%	2·7%	0·00
Pulmonary embolism	0·4%	0·4%	0·00	0·4%	0·4%	0·00
Venous thrombosis	0·8%	0·9%	−0·02	0·8%	0·9%	0·00
**Medical history: neoplasms**						
Haematological neoplasm	1·0%	1·2%	−0·02	1·1%	1·1%	0·00
Malignant lymphoma	0·6%	0·6%	0·00	0·6%	0·6%	0·00
Malignant neoplasm of anorectum	0·1%	0·1%	0·00	0·1%	0·1%	0·00
Malignant neoplastic disease	7·9%	9·7%	−0·06	8·6%	8·9%	−0·01
Malignant tumour of breast	0·1%	0·1%	−0·01	0·1%	0·1%	0·00
Malignant tumour of colon	0·3%	0·4%	−0·01	0·3%	0·3%	0·00
Malignant tumour of lung	0·3%	0·5%	−0·03	0·4%	0·4%	0·00
Malignant tumour of urinary bladder	..	..	..	..	..	..
Primary malignant neoplasm of prostate	3·0%	4·1%	−0·06	3·4%	3·5%	−0·01
**Medication use**						
Antibacterials for systemic use	15·6%	17·6%	−0·05	16·3%	16·6%	−0·01
Antidepressants	31·8%	31·6%	0·00	31·8%	31·8%	0·00
Antiepileptics	22·8%	21·2%	0·04	22·3%	22·3%	0·00
Anti-inflammatory and antirheumatic products	29·8%	33·3%	−0·07	31·1%	31·2%	0·00
Antineoplastic agents	2·5%	2·9%	−0·02	2·6%	2·7%	0·00
Antipsoriatics	0·6%	0·8%	−0·02	0·7%	0·7%	0·00
Antithrombotic agents	..	..	..	..	..	..
Drugs for acid-related disorders	34·2%	33·8%	0·01	34·0%	34·6%	−0·01
Drugs for obstructive airway diseases	26·1%	28·7%	−0·06	27·1%	27·5%	−0·01
Drugs used in diabetes	39·4%	14·8%	0·58	29·6%	28·5%	0·02
Immunosuppressants	2·6%	2·9%	−0·02	2·8%	2·8%	0·00
Lipid-modifying agents	64·8%	50·4%	0·30	58·9%	58·1%	0·02
Opioids	9·2%	9·8%	−0·02	9·5%	9·6%	−0·01
Psycholeptics	19·0%	20·6%	−0·04	19·7%	19·9%	−0·01
Psychostimulants, agents used for attention deficit hyperactivity disorder, and nootropics	1·4%	1·4%	0·01	1·4%	1·4%	0·00
**Race**						
American Indian or Alaska Native	0·9%	0·6%	0·03	0·7%	0·7%	0·00
Asian	1·2%	0·8%	0·04	1·1%	1·1%	0·00
Black or African American	12·9%	33·5%	−0·50	21·1%	21·3%	0·00
Native Hawaiian or other Pacific Islander	1·0%	0·8%	0·03	0·9%	0·9%	0·00
White	77·5%	58·6%	0·41	70·0%	69·9%	0·00
Other or unknown	6·6%	5·7%	0·03	6·2%	6·2%	0·00
**Ethnicity**						
Hispanic or Latino	7·6%	5·2%	0·10	6·7%	6·5%	0·01
Not Hispanic or Latino	89·7%	92·4%	−0·09	90·8%	90·9%	0·00

We report the proportion of selected baseline characteristics and standardised mean difference among ACEI, ARB, CCB, and THZ users before and after propensity score stratification. Less extreme standard differences through stratification suggest improved balance between patient cohorts through propensity score adjustment. ACEI=angiotensin-converting enzyme inhibitor. ARB=angiotensin receptor blocker. CCB=calcium channel blocker. THZ=thiazide or thiazide-like diuretic. VA-OMOP=US Department of Veterans Affairs Observational Medical Outcomes Partnership.

**Table 4:** Baseline patient characteristics for prevalent use of ACEI or ARB (target) and CCB or THZ (comparator) monotherapy in the CUIMC data source

	**Before stratification**			**After stratification**		
	Target CUIMC cohort (n=5539)	Comparator CIUMC cohort (n=5849)	Standardised mean difference	Target CUIMC cohort (n=5539)	Comparator CIUMC cohort (n=5849)	Standardised mean difference
**Age group, years**						
<25	0·6%	0·5%	0·01	0·7%	0·4%	0·04
25–29	0·7%	1·1%	−0·04	0·8%	0·8%	0·00
30–34	1·1%	1·5%	−0·03	1·3%	1·2%	0·01
35–39	1·8%	2·3%	−0·04	2·0%	1·9%	0·01
40–44	2·8%	3·5%	−0·04	2·8%	3·1%	−0·02
45–49	4·7%	5·3%	−0·03	5·1%	4·9%	0·01
50–54	7·1%	7·0%	0·00	7·3%	6·6%	0·03
55–59	9·9%	9·0%	0·03	9·7%	9·2%	0·02
60–64	13·1%	11·4%	0·05	12·5%	12·3%	0·01
65–69	14·9%	13·4%	0·04	14·4%	13·7%	0·02
70–74	15·4%	15·0%	0·01	15·1%	15·8%	−0·02
75–79	11·8%	11·9%	0·00	11·8%	12·4%	−0·02
80–84	8·3%	8·4%	0·00	8·1%	8·6%	−0·02
85–89	4·7%	5·5%	−0·03	5·0%	5·3%	−0·01
90–94	2·0%	3·0%	−0·06	2·3%	2·7%	−0·03
≥95	0·8%	0·8%	0·00	1·0%	0·8%	0·02
**Sex**						
Female	50·1%	58·7%	−0·17	55·2%	53·9%	0·03
Male	49·9%	41·3%	−0·17	44·8%	46·1%	0·03
**Medical history: general**						
Acute respiratory disease	3·6%	4·0%	−0·02	3·8%	3·8%	0·00
Attention deficit hyperactivity disorder	0·2%	0·2%	−0·02	0·2%	0·2%	−0·01
Chronic liver disease	0·7%	1·2%	−0·05	0·7%	1·0%	−0·04
Chronic obstructive lung disease	2·7%	3·1%	−0·02	2·9%	2·8%	0·00
Crohn's disease	0·3%	0·2%	0·01	0·3%	0·2%	0·03
Dementia	1·7%	2·3%	−0·04	1·9%	2·1%	−0·02
Depressive disorder	4·9%	6·0%	−0·05	5·6%	5·5%	0·01
Diabetes	21·5%	13·2%	0·22	16·7%	17·0%	−0·01
Gastro-oesophageal reflux disease	7·1%	7·1%	0·00	7·3%	6·8%	0·02
Gastrointestinal haemorrhage	0·8%	1·1%	−0·03	0·8%	0·8%	0·00
HIV infection	1·9%	1·7%	0·02	2·1%	1·5%	0·05
Hyperlipidaemia	38·3%	33·1%	0·11	35·5%	35·6%	0·00
Hypertensive disorder	61·1%	69·0%	−0·17	65·7%	64·3%	0·03
Lesion of liver	0·9%	1·5%	−0·06	1·0%	1·4%	−0·04
Obesity	9·2%	9·8%	−0·02	9·3%	9·7%	−0·01
Osteoarthritis	10·9%	12·2%	−0·04	11·8%	11·8%	0·00
Pneumonia	1·1%	1·9%	−0·06	1·2%	1·6%	−0·03
Psoriasis	0·7%	0·5%	0·03	0·6%	0·5%	0·02
Renal impairment	6·5%	9·7%	−0·12	7·8%	8·4%	−0·02
Rheumatoid arthritis	0·8%	0·8%	0·00	0·9%	0·8%	0·02
Schizophrenia	0·2%	0·3%	−0·02	0·2%	0·2%	0·00
Ulcerative colitis	0·2%	0·1%	0·01	0·2%	0·1%	0·02
Urinary tract infectious disease	2·1%	2·8%	−0·05	2·5%	2·5%	0·00
Viral hepatitis C	0·4%	0·8%	−0·05	0·5%	0·7%	−0·03
Visual system disorder	11·1%	10·2%	0·03	10·8%	10·2%	0·02
Atrial fibrillation	5·3%	4·6%	0·03	5·0%	4·8%	0·01
Cerebrovascular disease	5·3%	5·0%	0·01	5·2%	5·0%	0·01
Coronary arteriosclerosis	13·0%	10·7%	0·07	11·8%	12·1%	−0·01
Heart disease	27·7%	25·0%	0·06	26·4%	26·2%	0·00
Heart failure	4·2%	2·4%	0·10	3·5%	2·8%	0·04
Ischaemic heart disease	3·4%	3·3%	0·00	3·1%	3·6%	−0·03
Peripheral vascular disease	3·9%	3·2%	0·04	3·4%	3·3%	0·00
Pulmonary embolism	0·3%	0·5%	−0·02	0·4%	0·4%	0·00
Venous thrombosis	0·6%	1·2%	−0·06	0·7%	1·0%	−0·04
**Medical history: neoplasms**						
Haematological neoplasm	1·8%	1·8%	0·00	1·8%	1·5%	0·03
Malignant lymphoma	1·0%	1·3%	−0·03	1·0%	1·2%	−0·02
Malignant neoplasm of anorectum	0·2%	0·2%	0·01	0·2%	0·2%	0·01
Malignant neoplastic disease	9·5%	10·8%	−0·04	9·9%	10·2%	−0·01
Malignant tumour of breast	1·7%	1·7%	0·00	1·9%	1·5%	0·03
Malignant tumour of colon	0·3%	0·5%	−0·04	0·3%	0·5%	−0·03
Malignant tumour of lung	0·5%	0·6%	−0·02	0·6%	0·6%	0·00
Malignant tumour of urinary bladder	0·5%	0·5%	0·00	0·5%	0·5%	0·01
Primary malignant neoplasm of prostate	1·5%	1·8%	−0·03	1·4%	1·7%	−0·03
**Medication use**						
Antibacterials for systemic use	25·7%	27·1%	−0·03	26·1%	25·7%	0·01
Antidepressants	15·2%	15·5%	−0·01	15·4%	15·1%	0·01
Antiepileptics	13·3%	13·0%	0·01	13·4%	12·7%	0·02
Anti-inflammatory and antirheumatic products	17·1%	20·5%	−0·09	18·6%	18·9%	−0·01
Antineoplastic agents	3·2%	4·1%	−0·05	3·6%	3·6%	0·00
Antipsoriatics	0·6%	1·3%	−0·08	0·7%	1·1%	−0·04
Antithrombotic agents	21·9%	22·5%	−0·02	21·7%	22·0%	−0·01
Drugs for acid-related disorders	22·6%	26·9%	−0·10	24·3%	24·9%	−0·01
Drugs for obstructive airway diseases	14·0%	15·1%	−0·03	14·5%	14·3%	0·00
Drugs used in diabetes	22·7%	12·8%	0·26	17·0%	17·8%	−0·02
Immunosuppressants	5·4%	7·7%	−0·09	6·6%	6·3%	0·01
Lipid-modifying agents	43·2%	35·0%	0·17	38·4%	39·4%	−0·02
Opioids	10·3%	14·4%	−0·12	11·6%	12·6%	−0·03
Psycholeptics	14·3%	15·9%	−0·05	15·1%	15·1%	0·00
Psychostimulants, agents used for attention deficit hyperactivity disorder, and nootropics	1·5%	1·5%	0·00	1·5%	1·5%	0·00
**Race**						
American Indian or Alaska Native	0·2%	<0·1%	0·04	0·2%	<0·1%	0·04
Asian	2·3%	2·1%	0·02	2·3%	2·2%	0·02
Black or African American	5·9%	10·8%	−0·18	8·3%	8·3%	0·00
Native Hawaiian or other Pacific Islander	0·8%	0·6%	0·02	0·9%	0·5%	0·04
White	36·4%	31·0%	0·11	33·3%	33·9%	−0·01
Other or unknown	2·1%	2·3%	−0·03	2·0%	2·3%	−0·02
**Ethnicity**						
Hispanic or Latino	11·5%	13·9%	−0·07	12·6%	13·1%	−0·01
Not Hispanic or Latino	35·3%	34·4%	0·02	34·3%	35·2%	−0·02

We report the proportion of selected baseline characteristics and standardised mean difference among ACEI, ARB, CCB, and THZ users before and after propensity score stratification. Less extreme standard differences through stratification suggest improved balance between patient cohorts through propensity score adjustment. ACEI=angiotensin-converting enzyme inhibitor. ARB=angiotensin receptor blocker. CCB=calcium channel blocker. CUIMC=Columbia University Irving Medical Center data warehouse. THZ=thiazide or thiazide-like diuretic.

**Figure 2:**
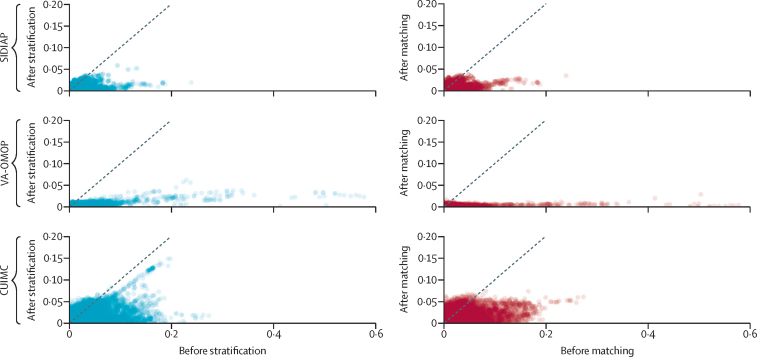
Cohort balance diagnostics comparing ACEI or ARB and CCB or THZ monotherapy prevalent use for the risk of COVID-19 diagnosis We plotted the absolute standardised difference in population proportions of all available patient characteristics (6571 in SIDIAP, 11 183 in VA-OMOP, and 18 291 in CUIMC) before and after propensity score stratification or matching across data sources. Using stratification, CUIMC fails study diagnostics for this comparison as the absolute standardised mean difference is not consistently less than 0·1. Dashed lines indicate no before and after adjustment. ACEI=angiotensin-converting enzyme inhibitor. ARB=angiotensin receptor blocker. CCB=calcium channel blocker. CUIMC=Columbia University Irving Medical Center data warehouse. SIDIAP=Information Systems for Research in Primary Care. THZ=thiazide or thiazide-like diuretic. VA-OMOP=US Department of Veterans Affairs Observational Medical Outcomes Partnership.

The number of baseline patient characteristics differed across comparison cohorts and data sources. The process generated more than 10 000 unique features in each data source; the number of characteristics present in at least 1% of patients in each comparison cohort ranged from 2284 to 2473 in SIDIAP, 2657 to 3366 in VA-OMOP, and 2694 to 3859 in CUIMC. After large-scale propensity score construction, followed by stratification or matching, standardised mean differences for all baseline characteristics were less than 0·1 in SIDIAP and VA-OMOP for each drug comparison, apart from the comparison between combination users of ARBs and CCBs or THZs in VA-OMOP. Standardised mean differences for all baseline characteristics before and after propensity score adjustment for ACEI or ARB monotherapy users compared with CCB or THZ monotherapy users for all data sources are plotted in figure 2. In CUIMC, all but one drug comparison (ACEI *vs* ARB monotherapy) with propensity score stratification showed residual cohort imbalances, with a standardised mean difference of 0·1 or more, which involved baseline characteristics related to pregnancy, renal transplantation, and heart failure and use of sacubitril. However, these cohort comparisons all passed study diagnostics for the propensity score matching design. The appendix (pp 163–282) shows study diagnostics for all comparisons and includes negative control effect estimate distributions. The number of negative control outcomes analysed ranged from 33 to 80 in CUIMC, 49 to 65 in SIDIAP, and 99 to 105 in VA-OMOP (appendix pp 314–17).

**Figure 3:**
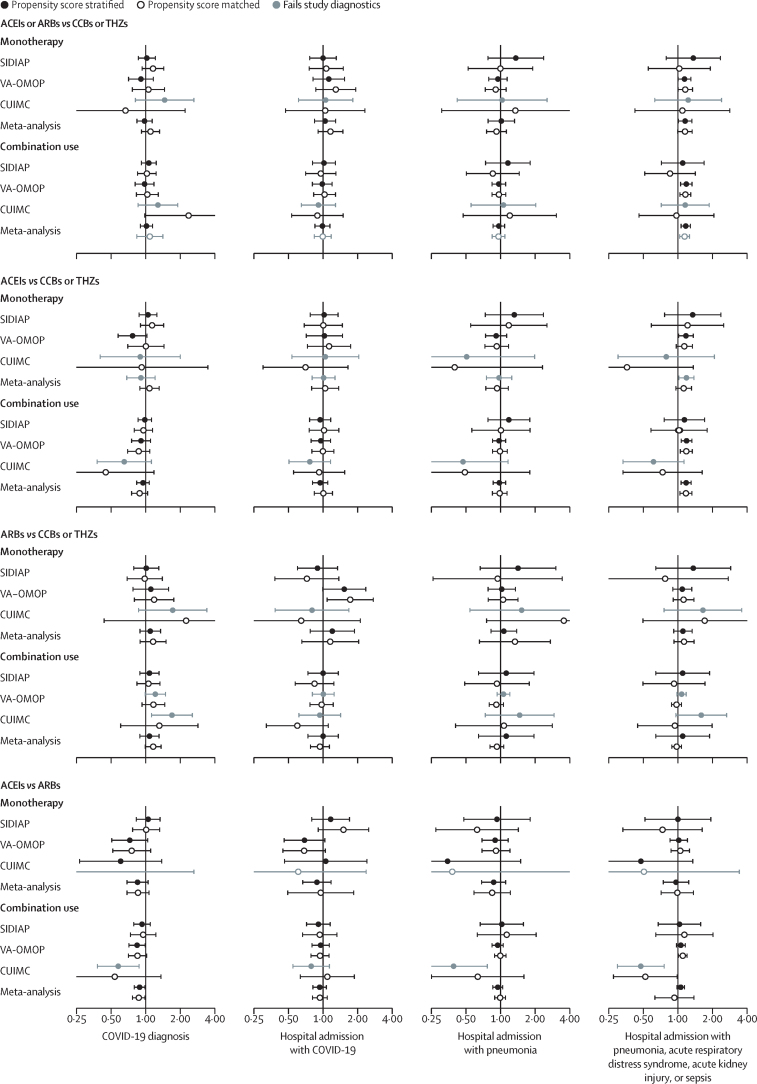
Calibrated HRs for COVID-19-related outcomes for ACEI, ARB, CCB, and THZ prevalent use across data sources Outcomes are COVID-19 diagnosis, hospital admission with COVID-19, hospital admission with pneumonia, and hospital admission with pneumonia, acute respiratory distress syndrome, acute kidney injury, or sepsis. We plot calibrated HRs and their 95% CIs (indicated by error bars) labelled by propensity score adjustment method. Greyed out data source entries do not pass study diagnostics and greyed out meta-analysis entries return a heterogeneity (*I*^2^) value of more than 40%. ACEI=angiotensin-converting enzyme inhibitor. ARB=angiotensin receptor blocker. CCB=calcium channel blocker. CUIMC=Columbia University Irving Medical Center data warehouse. HR=hazard ratio. SIDIAP=Information Systems for Research in Primary Care. THZ=thiazide or thiazide-like diuretic. VA-OMOP=US Department of Veterans Affairs Observational Medical Outcomes Partnership.

Calibrated HRs for the relative risk of incident COVID-19 diagnosis are presented in table 3 and figure 3 for propensity score-stratified and propensity score-matched analyses. In SIDIAP, there were 37 796 monotherapy and 45 239 combination users of ACEIs or ARBs. Compared with use of CCBs, the risk of COVID-19 diagnosis with propensity score stratification was not significantly different (HR 1·02, 95% CI 0·86–1·21, with monotherapy and 1·06, 0·92–1·24, with combination use). In VA-OMOP, there were 320 450 monotherapy and 656 274 combination users of ACEIs or ARBs. Compared with CCB or THZ use in VA-OMOP, the risk of COVID-19 diagnosis was not significantly different (HR 0·91, 95% CI 0·71–1·17, with monotherapy and 0·98, 0·81–1·18, with combination use). Propensity score stratification in CUIMC with 5539 monotherapy users and 10 286 combinations users of ACEI or ARB did not pass study diagnostics. The corresponding HRs for CUIMC using propensity score matching were 0·67 (95% CI 0·20–2·20) with monotherapy and 2·36 (0·98–5·68) with combination use. Meta-analytic HRs following propensity score stratification for ACEI or ARB use compared with CCB or THZ use were 0·98 (95% CI 0·84–1·14) for monotherapy and 1·01 (0·90–1·15) with combination use (table 5).

**Table 5:** Risk of COVID-19 diagnosis among users of ACEIs, ARBs, CCBs, and THZs

		**Propensity score stratified**		**Propensity score matched**	
		Calibrated HR (95% CI)	Calibrated p value	Calibrated HR (95% CI)	Calibrated p value
**ACEIs or ARBs *vs* CCBs or THZs**					
Monotherapy					
	SIDIAP	1·02 (0·86–1·21)	0·76	1·16 (0·93–1·44)	0·24
	VA-OMOP	0·91 (0·71–1·17)	0·48	1·06 (0·76–1·46)	0·74
	CUIMC	1·46 (0·81–2·62)*	0·22*	0·67 (0·20–2·20)	0·53
	Meta-analysis	0·98 (0·84–1·14)	0·76	1·10 (0·92–1·32)	0·31
Combination use					
	SIDIAP	1·06 (0·92–1·24)	0·47	1·02 (0·85–1·23)	0·76
	VA-OMOP	0·98 (0·81–1·18)	0·80	1·03 (0·83–1·29)	0·77
	CUIMC	1·28 (0·86–1·90)*	0·27*	2·36 (0·98–5·68)	0·06
	Meta-analysis	1·01 (0·90–1·15)	0·81	1·09 (0·84–1·41)†	0·54†
**ACEIs *vs* CCBs or THZs**					
Monotherapy					
	SIDIAP	1·05 (0·88–1·25)	0·68	1·14 (0·90–1·43)	0·34
	VA-OMOP	0·77 (0·57–1·03)	0·07	1·00 (0·70–1·44)	0·96
	CUIMC	0·90 (0·40–2·00)*	0·79*	0·92 (0·24–3·48)	0·86
	Meta-analysis	0·91 (0·68–1·21)†	0·51†	1·08 (0·89–1·31)	0·45
Combination use					
	SIDIAP	0·98 (0·86–1·12)	0·75	0·95 (0·79–1·15)	0·66
	VA-OMOP	0·91 (0·75–1·10)	0·33	0·87 (0·70–1·08)	0·21
	CUIMC	0·65 (0·38–1·12)*	0·14*	0·45 (0·17–1·18)	0·11
	Meta-analysis	0·95 (0·83–1·07)	0·38	0·88 (0·75–1·04)	0·14
**ARBs *vs* CCBs or THZs**					
Monotherapy					
	SIDIAP	1·01 (0·79–1·30)	0·76	0·98 (0·69–1·39)	0·81
	VA-OMOP	1·11 (0·78–1·58)	0·57	1·18 (0·80–1·76)	0·41
	CUIMC	1·72 (0·87–3·40)*	0·13*	2·25 (0·43–11·6)	0·34
	Meta-analysis	1·10 (0·89–1·35)	0·40	1·16 (0·89–1·50)	0·28
Combination use					
	SIDIAP	1·08 (0·89–1·31)	0·47	1·06 (0·84–1·33)	0·65
	VA-OMOP	1·21 (0·99–1·49)*	0·07*	1·17 (0·93–1·47)	0·19
	CUIMC	1·69 (1·12–2·55)*	0·02*	1·31 (0·61–2·85)	0·51
	Meta-analysis	1·08 (0·89–1·31)	0·49	1·16 (0·99–1·36)	0·08
**ACEIs *vs* ARBs**					
Monotherapy					
	SIDIAP	1·05 (0·83–1·33)	0·70	1·01 (0·77–1·32)	0·79
	VA-OMOP	0·73 (0·51–1·04)	0·09	0·75 (0·52–1·10)	0·15
	CUIMC	0·61 (0·27–1·38)	0·25	0·22 (0·02–2·63)*	0·24*
	Meta-analysis	0·85 (0·69–1·05)	0·14	0·86 (0·69–1·07)	0·18
Combination use					
	SIDIAP	0·93 (0·79–1·10)	0·46	0·95 (0·74–1·22)	0·67
	VA-OMOP	0·84 (0·71–0·99)	0·04	0·85 (0·71–1·02)	0·08
	CUIMC	0·58 (0·38–0·87)*	0·01*	0·54 (0·21–1·36)	0·20
	Meta-analysis	0·88 (0·79–0·99)	0·03	0·87 (0·77–0·99)	0·04

ACEI=angiotensin-converting enzyme inhibitor. ARB=angiotensin receptor blocker. CCB=calcium channel blocker. CUIMC=Columbia University Irving Medical Center data warehouse. HR=hazard ratio. SIDIAP=Information Systems for Research in Primary Care. THZ=thiazide or thiazide-like diuretic. VA-OMOP=US Department of Veterans Affairs Observational Medical Outcomes Partnership.

* Data source entries do not pass study diagnostics and not included in the meta-analytic estimate.

† Entries return heterogeneity (*I*^2^) values of more than 40%.

When comparing ACEI and ARB use separately to CCB or THZ use, we observed no significant difference with COVID-19 diagnosis for comparisons passing study diagnostics (table 3). For ACEI use, meta-analytic HRs following propensity score stratification were 0·91 (95% CI 0·68–1·21) for monotherapy (but with heterogeneity of more than 40%) and 0·95 (0·83–1·07) for combination use. For ARB use, meta-analytic HRs following propensity score stratification were 1·10 (95% CI 0·89–1·35) with monotherapy and 1·08 (0·89–1·31) with combination use.

When comparing ACEI use directly with ARB use, no significant difference in the risk of COVID-19 diagnosis was observed in individual databases, apart from combination use in VA-OMOP (HR 0·84, 95% CI 0·71–0·99). Meta-analytic HRs following propensity score stratification were 0·85 (95% CI 0·69–1·05) with monotherapy and 0·88 (0·79–0·99) for combination use. Propensity score matching, where comparisons from CUIMC passed all propensity score diagnostics, produced similar results (table 5).

Calibrated HRs for the risk of hospital admission with COVID-19 are presented in figure 3. We observed no significant association between incident hospital admission with COVID-19 for the comparison with ACEI or ARB use, evaluated either together or separately, compared with CCB or THZ use. For ACEI use compared with ARB use, using propensity score stratification, meta-analytic HRs were 0·88 (95% CI 0·66–1·17) for monotherapy and 0·93 (0·82–1·07) with combination use.

No significant associations with the risk of pneumonia were observed with any drug comparison that satisfied study diagnostics. No significant associations with the risk of hospital admission with pneumonia, acute respiratory distress syndrome, acute kidney injury, or sepsis were observed with any drug comparison that satisfied study diagnostics in SIDIAP and CUIMC. In VA-OMOP, no significant difference was observed in comparisons between ARB versus CCB or THZ use (HR 1·09, 95% CI 0·90–1·32) or ACEI versus ARB use (1·02, 0·85–1·21) and the risk of hospital admission with pneumonia, acute respiratory distress syndrome, acute kidney injury, or sepsis, although small significant associations were observed with ACEI versus CCB or THZ (1·17, 1·01–1·36; appendix p 290).

## Discussion

In this multicentre cohort study following more than 1·3 million patients with hypertension from the USA and Spain, we observed no clear association of increased risk of COVID-19 diagnosis, hospital admission, or subsequent complications associated with the outpatient prevalent ACEI or ARB use. Our findings support recent regulatory and clinical society recommendations that patients should not halt their ACEI or ARB therapy despite previously posited mechanisms of increased COVID-19 risk.^16^

Studies assessing the risk of COVID-19 among ACEI or ARB users have been published from Italy, Spain, the UK, and the USA.^37^, ^38^, ^39^, ^40^, ^41^, ^42^ After adjustment for the higher prevalence of cardiovascular conditions in patients with COVID-19, ACEI and ARB use was not associated with an increased risk of COVID-19 diagnosis. These case-control studies included only a limited number of covariates for model adjustment. We identified only two studies that compared the risk of COVID-19 susceptibility in ACEI or ARB users with an active comparator.^41^, ^42^ In this context, comparing patients with similarly indicated treatments is crucial for reducing the risk of bias resulting from confounding by indication (eg, hypertension), in which the absence of treatment indicates either too mild a disease to warrant pharmacological treatment (eg, mild hypertension under control with lifestyle and diet changes), the presence of contraindications, or extreme frailty precluding the use of preventive medicines (eg, at the end of life).^43^, ^44^, ^45^, ^46^ Indeed, de Abajo and colleagues clearly demonstrate that compared with other antihypertensive medication use, non-use was associated with a significantly reduced risk of being admitted to hospital with COVID-19, with an estimated odds ratio of 0·48 (95% CI 0·34–0·69) for severe cases and 0·57 (0·43–0·75) for less severe cases.^41^

We observed one nominally significant meta-analysis difference: users of ACEI combination therapy had a lower risk of COVID-19 diagnosis when compared with users of ARB combination therapy. There was, however, no corresponding difference detected in hospital admission or complications. Therefore, the observed association might be due to chance or residual bias. Even if true, there is only a 12% difference, and therefore favouring ACEIs over ARBs for mitigating COVID-19 is not strongly supported by our result. There is limited evidence directly comparing the risk of COVID-19 between ACEI and ARB use. Several studies have reported main effect odds ratios lower than 1 with ACEIs compared with ARBs, ranging from 0·61 (95% CI 0·41–0·93) to 0·92 (0·64–1·32) for ACEIs and 0·88 (0·61–1·26) to 1·10 (0·88–1·37) for ARBs.^38^, ^41^, ^42^, ^43^ However, not all observational studies have suggested a differential effect between ACEI and ARB use.^39^ Notably, one study comparing 124 ACEI users admitted to hospital with COVID-19 matched to 248 ARB users found no difference in the risk of 28-day all-cause mortality.^47^

Animal models suggest that although ACEIs increase ACE2 gene expression, they do not alter ACE2 activity, unlike ARBs, providing a potential mechanism for why differential effects might occur.^7^, ^48^ However, recent studies in humans have identified no difference in ACE2 levels following exposure to ACEI or ARB use.^49^, ^50^, ^51^ Therefore, our findings could also be explained by residual confounding, as suggested by recent comparisons of the incidence of *Staphylococcus aureus* infection and other outcomes between ACEI and ARB use, which suggest that ARB use is not a perfect comparator for ACEI use, although no large-scale propensity score adjustment was used.^52^

Furthermore, one study has reported an increased risk of hospital admission with COVID-19 and intensive care unit admission associated with use of ACEIs and ARBs.^43^ Although we did not observe a consistent increased risk of hospital admission with COVID-19, we did observe an increased risk of hospital admission with pneumonia, acute respiratory distress syndrome, acute kidney injury, or sepsis largely driven by ACEI use compared with CCB or THZ use. This finding might be related to the higher incidence of acute kidney injury associated with ACEI use because no increased risk was observed for pneumonia, and acute kidney injury would be considerably more frequent than acute respiratory distress syndrome or sepsis.

We used an open science approach to apply analyses across a network of observational databases so results can be directly compared and interpreted in aggregate. For these analyses, we used active comparators to reduce confounding by indication and, for the first time in such a study, applied large-scale propensity adjustment with full diagnostics and did a large set of negative control experiments. We published the study protocol ahead of time and kept results blinded when assessing propensity score diagnostics, helping to address concerns about reproducibility, robustness, and transparency that have emerged.^53^ Our study has also been recognised by the European Medicines Agency ENCePP Guide on Methodological Standards in Pharmacoepidemiology for COVID-19 studies.^54^

We examined outpatient prevalent use of antihypertensive therapy because a new-user design in the context of COVID-19, which has widely affected the provision of routine care, is infeasible. Therefore, mediators on the causal pathway between exposure and outcome might be included in the adjustment. However, this might not necessarily result in bias, as COVID-19 is a new illness and will not have affected the decision to initiate one drug over another. Similarly, biological mechanisms relating to ACE2 expression might require chronic exposure, hindering a new-user design. Previous treatment remains highly correlated with many baseline features that our large-scale propensity model considers when balancing patients and can provide some protection against this potential bias.

Furthermore, we defined COVID-19 diagnosis through the presence of diagnostic codes or positive test results that will underestimate the number of true COVID-19 cases, the extent of which will vary by site due to differences in testing strategies. To address this potential limitation, we included a hospital admission-based COVID-19 outcome and observed similar results. Differences in the incidence of outcomes were noted between data sources, with VA-OMOP having much lower incidence than SIDIAP and CUIMC. This finding might relate to differences in the capture of COVID-19 diagnosis and hospital admission within each database, and differences in baseline community incidence. For example, the population in CUIMC are from New York City, which was the epicentre for US cases during the conduct of the study. Finally, although exposure is based on prescription information, we cannot determine whether the patient ingested their medication. Nevertheless, these data are representative of how patients use such medications in the real world. Although we have used a rigorous approach to observational research,^33^ residual confounding is still possible.

Our findings stand in agreement with regulatory and clinical society advice that ACEI and ARB therapy should be continued in light of COVID-19. Furthermore, the marginal difference between ACEIs and ARBs does not warrant class switching to reduce COVID-19 susceptibility.

## Data sharing

As this is a distributed data analysis, individual patient-level data from each database cannot be shared due to database governance restrictions. The supplement is available online. The prespecified ICARIUS protocol and start-to-finish open and executable source code are available online. To promote transparency and facilitate sharing and exploration of the complete result set, an interactive web application provides study diagnostics and results for all study effects.
