# Supplementary material for: Renin–angiotensin system blockers and susceptibility to COVID-19: an international, open science, cohort analysis
Source: Lancet Digit Health. 2020 Dec 17;3(2):e98–e114. doi: 10.1016/S2589-7500(20)30289-2 (PMC7834915; doi:10.1016/S2589-7500(20)30289-2)
Supplement: Supplementary appendix [file mmc1.pdf]

THE LANCET
Digital Health

Supplementary appendix

This appendix formed part of the original submission and has been peer reviewed. We post it as supplied by the authors.

Supplement to: Morales DR, Conover MM, You SC et al. Renin–angiotensin system blockers and susceptibility to COVID-19: an international, open science, cohort analysis. *Lancet Digit Health* 2020; published online Dec 17. https://doi.org/10.1016/S2589-7500(20)30289-2.

Supplementary Material

for

Renin-angiotensin system blockers and susceptibility to COVID-19

by

Daniel R. Morales, Mitchell M. Conover, Seng Chan You, et al.

Contents

Sample sizes, total follow-up times and events **2**
- COVID-19 diagnoses . . . 2
- COVID-19 hospitalizations . . . 5
- Pneumonia . . . 8
- Pneumonia, acute respiratory distress syndrome, acute kidney injury or sepsis (PAAS) . . . 11

Baseline patient characteristics **14**
- SIDIAP . . . 14
- VA-OMOP . . . 55
- CUIMC . . . 103

Cohort balance and systematic error diagnostics **163**
- SIDIAP . . . 163
- VA-OMOP . . . 203
- CUIMC . . . 243

Effect estimates **283**
- PS Stratification . . . 283
  - SIDIAP . . . 283
  - VA-OMOP . . . 287
  - CUIMC . . . 291
  - Meta-analysis . . . 295
- PS Matching . . . 299
  - SIDIAP . . . 299
  - VA-OMOP . . . 303
  - CUIMC . . . 307
  - Meta-analysis . . . 311

Negative control outcomes **314**

Sample sizes, total follow-up times and events

COVID-19 diagnoses

Supplementary Table 1: Population size, total exposure time and COVID-19 diagnoses (events) for ACEI, ARB and CCB/THZ monotherapy and in-combination user target (T) and comparator (C) cohorts.

	Patients		Time (years)		Events		
	T	C	T	C	T	C	MDRR
**ACEI/ARB vs CCB/THZ**							
Monotherapy							
SIDIAP	37,796	14,003	10,239	3,780	500	184	1.27
VA-OMOP	320,450	229,063	110,380	76,856	145	183	1.37
CUIMC	5,539	5,849	594	625	28	24	2.18
+ Combination							
SIDIAP	45,239	19,007	12,264	5,175	627	250	1.23
VA-OMOP	656,274	443,061	228,678	150,755	345	335	1.24
CUIMC	10,286	11,008	1,128	1,185	59	58	1.68
**ACEI/ARB vs CCB**							
Monotherapy							
SIDIAP	37,796	8,682	10,239	2,303	500	107	1.34
VA-OMOP	320,450	138,471	110,380	45,904	145	108	1.47
CUIMC	5,539	3,818	594	378	28	16	2.36
+ Combination							
SIDIAP	67,055	12,292	18,260	3,294	921	163	1.27
VA-OMOP	921,091	312,032	322,619	105,336	474	259	1.27
CUIMC	14,222	7,923	1,582	799	80	43	1.69
**ACEI/ARB vs THZ**							
Monotherapy							
SIDIAP	37,796	4,957	10,239	1,340	500	66	1.44
VA-OMOP	320,450	59,235	110,380	19,675	145	41	1.76
CUIMC	5,539	1,521	594	170	28	5	3.27
+ Combination							
SIDIAP	50,208	7,304	13,567	1,980	712	88	1.35
VA-OMOP	889,171	185,520	311,337	62,694	526	112	1.34
CUIMC	13,487	3,939	1,442	431	79	16	1.99
**ACEI vs ARB**							
Monotherapy							
SIDIAP	30,787	6,753	8,293	1,815	398	95	1.39
VA-OMOP	235,348	82,872	80,760	28,689	96	46	1.71
CUIMC	2,576	2,860	277	301	10	17	2.94
+ Combination							
SIDIAP	56,465	19,148	15,333	5,176	758	283	1.22

(Continued)	Patients		Time (years)		Events		
	T	C	T	C	T	C	MDRR
VA-OMOP	865,931	395,156	303,491	140,071	441	282	1.25
CUIMC	7,880	10,769	826	1,179	39	66	1.74
**ACEI vs CCB/THZ**							
Monotherapy							
SIDIAP	30,787	14,003	8,293	3,780	398	184	1.28
VA-OMOP	235,348	229,063	80,760	76,856	96	183	1.40
CUIMC	2,576	5,849	277	625	10	24	2.84
+ Combination							
SIDIAP	36,323	29,239	9,803	7,941	485	399	1.21
VA-OMOP	457,557	639,500	158,721	221,239	218	494	1.24
CUIMC	4,811	16,302	511	1,754	18	83	1.94
**ACEI vs CCB**							
Monotherapy							
SIDIAP	30,787	8,682	8,293	2,303	398	107	1.35
VA-OMOP	235,348	138,471	80,760	45,904	96	108	1.50
CUIMC	2,576	3,818	277	378	10	16	3.07
+ Combination							
SIDIAP	51,414	15,799	13,920	4,214	682	219	1.25
VA-OMOP	647,931	434,028	225,841	148,898	301	375	1.25
CUIMC	6,182	10,653	666	1,060	31	58	1.85
**ACEI vs THZ**							
Monotherapy							
SIDIAP	30,787	4,957	8,293	1,340	398	66	1.46
VA-OMOP	235,348	59,235	80,760	19,675	96	41	1.82
CUIMC	2,576	1,521	277	170	10	5	4.47
+ Combination							
SIDIAP	39,341	15,585	10,583	4,221	536	202	1.26
VA-OMOP	607,700	299,205	211,487	102,799	323	184	1.30
CUIMC	6,332	7,553	658	817	26	31	2.11
**ARB vs CCB/THZ**							
Monotherapy							
SIDIAP	6,753	14,003	1,815	3,780	95	184	1.43
VA-OMOP	82,872	229,063	28,689	76,856	46	183	1.52
CUIMC	2,860	5,849	301	625	17	24	2.54
+ Combination							
SIDIAP	9,194	39,427	2,457	10,714	137	519	1.32
VA-OMOP	201,503	854,224	70,267	295,986	127	574	1.31
CUIMC	5,669	14,271	629	1,533	41	77	1.77
**ARB vs CCB**							
Monotherapy							

(Continued)	Patients		Time (years)		Events		
	T	C	T	C	T	C	MDRR
SIDIAP	6,753	8,682	1,815	2,303	95	107	1.49
VA-OMOP	82,872	138,471	28,689	45,904	46	108	1.59
CUIMC	2,860	3,818	301	378	17	16	2.68
+ Combination							
SIDIAP	16,120	17,822	4,339	4,748	233	237	1.30
VA-OMOP	277,010	533,884	97,228	183,297	174	408	1.28
CUIMC	8,320	9,901	934	994	49	51	1.75
**ARB vs THZ**							
Monotherapy							
SIDIAP	6,753	4,957	1,815	1,340	95	66	1.56
VA-OMOP	82,872	59,235	28,689	19,675	46	41	1.84
CUIMC	2,860	1,521	301	170	17	5	3.51
+ Combination							
SIDIAP	11,190	24,751	2,980	6,724	170	305	1.32
VA-OMOP	285,354	447,635	100,313	154,354	201	234	1.32
CUIMC	7,415	5,747	800	622	53	29	1.87

COVID-19 hospitalizations

Supplementary Table 2: Population size, total exposure time and COVID-19 hospitalizations (events) for ACEI, ARB and CCB/THZ monotherapy and in-combination user target (T) and comparator (C) cohorts.

	Patients		Time (years)		Events		
	T	C	T	C	T	C	MDRR
**ACEI/ARB vs CCB/THZ**							
Monotherapy							
SIDIAP	37,796	14,003	10,247	3,783	190	80	1.47
VA-OMOP	320,418	229,022	110,375	76,856	110	97	1.48
CUIMC	5,535	5,819	593	621	27	34	2.05
+ Combination							
SIDIAP	45,239	19,007	12,274	5,178	241	110	1.39
VA-OMOP	656,122	442,905	228,645	150,733	343	276	1.26
CUIMC	10,229	10,873	1,125	1,175	62	100	1.55
**ACEI/ARB vs CCB**							
Monotherapy							
SIDIAP	37,796	8,682	10,247	2,305	190	48	1.59
VA-OMOP	320,418	138,440	110,375	45,899	110	68	1.58
CUIMC	5,535	3,792	593	375	27	26	2.19
+ Combination							
SIDIAP	67,058	12,292	18,275	3,296	350	79	1.45
VA-OMOP	920,893	311,902	322,577	105,312	451	222	1.28
CUIMC	14,139	7,822	1,576	791	82	90	1.56
**ACEI/ARB vs THZ**							
Monotherapy							
SIDIAP	37,796	4,957	10,247	1,341	190	25	1.82
VA-OMOP	320,418	59,233	110,375	19,678	110	20	1.97
CUIMC	5,535	1,515	593	170	27	5	3.34
+ Combination							
SIDIAP	50,208	7,304	13,579	1,982	277	31	1.62
VA-OMOP	888,895	185,476	311,281	62,696	506	74	1.36
CUIMC	13,360	3,899	1,433	430	96	21	1.86
**ACEI vs ARB**							
Monotherapy							
SIDIAP	30,787	6,753	8,300	1,817	149	36	1.71
VA-OMOP	235,323	82,866	80,755	28,689	73	37	1.84
CUIMC	2,575	2,857	276	301	15	11	3.01
+ Combination							
SIDIAP	56,468	19,148	15,347	5,180	283	119	1.38
VA-OMOP	865,706	395,037	303,435	140,052	448	235	1.26
CUIMC	7,808	10,678	820	1,172	60	70	1.64

(Continued)	Patients		Time (years)		Events		
	T	C	T	C	T	C	MDRR
**ACEI vs CCB/THZ**							
Monotherapy							
SIDIAP	30,787	14,003	8,300	3,783	149	80	1.49
VA-OMOP	235,323	229,022	80,755	76,856	73	97	1.54
CUIMC	2,575	5,819	276	621	15	34	2.38
+ Combination							
SIDIAP	36,323	29,239	9,810	7,947	185	176	1.35
VA-OMOP	457,459	639,278	158,693	221,205	234	397	1.25
CUIMC	4,790	16,099	510	1,740	31	136	1.67
**ACEI vs CCB**							
Monotherapy							
SIDIAP	30,787	8,682	8,300	2,305	149	48	1.62
VA-OMOP	235,323	138,440	80,755	45,899	73	68	1.63
CUIMC	2,575	3,792	276	375	15	26	2.44
+ Combination							
SIDIAP	51,417	15,799	13,933	4,217	255	108	1.41
VA-OMOP	647,800	433,843	225,805	148,866	306	312	1.26
CUIMC	6,149	10,500	664	1,049	40	121	1.58
**ACEI vs THZ**							
Monotherapy							
SIDIAP	30,787	4,957	8,300	1,341	149	25	1.85
VA-OMOP	235,323	59,233	80,755	19,678	73	20	2.06
CUIMC	2,575	1,515	276	170	15	5	3.66
+ Combination							
SIDIAP	39,341	15,585	10,592	4,225	202	79	1.45
VA-OMOP	607,526	299,146	211,445	102,799	331	129	1.32
CUIMC	6,275	7,485	654	814	51	39	1.81
**ARB vs CCB/THZ**							
Monotherapy							
SIDIAP	6,753	14,003	1,817	3,783	36	80	1.74
VA-OMOP	82,866	229,022	28,689	76,856	37	97	1.73
CUIMC	2,857	5,819	301	621	11	34	2.43
+ Combination							
SIDIAP	9,194	39,430	2,459	10,723	54	207	1.56
VA-OMOP	201,450	853,932	70,263	295,933	111	490	1.34
CUIMC	5,632	14,083	627	1,518	31	126	1.64
**ARB vs CCB**							
Monotherapy							
SIDIAP	6,753	8,682	1,817	2,305	36	48	1.85
VA-OMOP	82,866	138,440	28,689	45,899	37	68	1.76
CUIMC	2,857	3,792	301	375	11	26	2.54

(Continued)	Patients		Time (years)		Events		
	T	C	T	C	T	C	MDRR
+ Combination							
SIDIAP	16,120	17,822	4,342	4,751	92	109	1.49
VA-OMOP	276,941	533,647	97,221	183,250	147	362	1.30
CUIMC	8,269	9,758	931	983	42	108	1.58
**ARB vs THZ**							
Monotherapy							
SIDIAP	6,753	4,957	1,817	1,341	36	25	2.07
VA-OMOP	82,866	59,233	28,689	19,678	37	20	2.12
CUIMC	2,857	1,515	301	170	11	5	4.36
+ Combination							
SIDIAP	11,190	24,754	2,982	6,730	73	109	1.57
VA-OMOP	285,249	447,539	100,298	154,343	179	190	1.35
CUIMC	7,340	5,688	795	620	46	30	1.91

Pneumonia

Supplementary Table 3: Population size, total exposure time and pneumonia (events) for ACEI, ARB and CCB/THZ monotherapy and in-combination user target (T) and comparator (C) cohorts.

	Patients		Time (years)		Events		
	T	C	T	C	T	C	MDRR
**ACEI/ARB vs CCB/THZ**							
Monotherapy							
SIDIAP	37,794	14,000	10,249	3,783	56	18	2.08
VA-OMOP	294,966	206,454	101,598	69,412	714	533	1.17
CUIMC	5,093	5,119	539	546	18	21	2.45
+ Combination							
SIDIAP	45,236	19,004	12,277	5,178	73	31	1.83
VA-OMOP	559,571	377,489	195,439	129,288	2,099	1,217	1.10
CUIMC	8,989	8,938	986	973	39	40	1.88
**ACEI/ARB vs CCB**							
Monotherapy							
SIDIAP	37,794	8,680	10,249	2,305	56	8	2.46
VA-OMOP	294,966	122,531	101,598	40,767	714	351	1.21
CUIMC	5,093	3,229	539	321	18	16	2.68
+ Combination							
SIDIAP	67,054	12,290	18,279	3,297	99	18	2.05
VA-OMOP	801,340	259,507	281,166	88,227	2,787	874	1.11
CUIMC	12,607	6,193	1,406	630	55	38	1.86
**ACEI/ARB vs THZ**							
Monotherapy							
SIDIAP	37,794	4,956	10,249	1,341	56	8	2.99
VA-OMOP	294,966	55,457	101,598	18,412	714	124	1.30
CUIMC	5,093	1,425	539	156	18	<5	6.55
+ Combination							
SIDIAP	50,204	7,303	13,581	1,982	80	13	2.39
VA-OMOP	751,635	165,524	264,047	56,300	3,004	499	1.13
CUIMC	11,567	3,441	1,238	382	57	7	2.30
**ACEI vs ARB**							
Monotherapy							
SIDIAP	30,786	6,752	8,302	1,817	43	12	2.67
VA-OMOP	215,877	77,046	74,082	26,662	535	175	1.27
CUIMC	2,356	2,641	251	274	6	12	3.75
+ Combination							
SIDIAP	56,465	19,146	15,349	5,182	85	32	1.81
VA-OMOP	750,435	338,525	263,693	120,292	2,842	1,280	1.10
CUIMC	6,735	9,516	709	1,044	29	54	1.87

(Continued)	Patients		Time (years)		Events		
	T	C	T	C	T	C	MDRR
**ACEI vs CCB/THZ**							
Monotherapy							
SIDIAP	30,786	14,000	8,302	3,783	43	18	2.17
VA-OMOP	215,877	206,454	74,082	69,412	535	533	1.19
CUIMC	2,356	5,119	251	546	6	21	3.19
+ Combination							
SIDIAP	36,321	29,234	9,812	7,948	55	44	1.76
VA-OMOP	391,311	547,695	136,106	190,547	1,465	1,819	1.10
CUIMC	4,160	13,617	445	1,482	14	62	2.14
**ACEI vs CCB**							
Monotherapy							
SIDIAP	30,786	8,680	8,302	2,305	43	8	2.58
VA-OMOP	215,877	122,531	74,082	40,767	535	351	1.22
CUIMC	2,356	3,229	251	321	6	16	3.35
+ Combination							
SIDIAP	51,414	15,795	13,935	4,218	72	22	1.98
VA-OMOP	565,487	362,097	197,496	125,012	1,969	1,272	1.11
CUIMC	5,398	8,495	585	852	19	52	1.98
**ACEI vs THZ**							
Monotherapy							
SIDIAP	30,786	4,956	8,302	1,341	43	8	3.11
VA-OMOP	215,877	55,457	74,082	18,412	535	124	1.31
CUIMC	2,356	1,425	251	156	6	<5	324.14
+ Combination							
SIDIAP	39,339	15,583	10,594	4,225	62	26	1.94
VA-OMOP	515,436	267,875	180,049	92,554	2,064	867	1.12
CUIMC	5,366	6,751	560	740	20	19	2.47
**ARB vs CCB/THZ**							
Monotherapy							
SIDIAP	6,752	14,000	1,817	3,783	12	18	2.98
VA-OMOP	77,046	206,454	26,662	69,412	175	533	1.27
CUIMC	2,641	5,119	274	546	12	21	2.80
+ Combination							
SIDIAP	9,193	39,426	2,459	10,724	18	61	2.24
VA-OMOP	170,394	738,653	59,583	257,331	646	2,502	1.14
CUIMC	4,989	11,665	551	1,264	26	51	2.01
**ARB vs CCB**							
Monotherapy							
SIDIAP	6,752	8,680	1,817	2,305	12	8	3.54
VA-OMOP	77,046	122,531	26,662	40,767	175	351	1.29
CUIMC	2,641	3,229	274	321	12	16	2.90

(Continued)	Patients		Time (years)		Events		
	T	C	T	C	T	C	MDRR
+ Combination							
SIDIAP	16,119	17,820	4,344	4,752	27	31	2.09
VA-OMOP	238,908	447,373	84,044	154,714	845	1,630	1.13
CUIMC	7,447	7,762	836	785	37	47	1.84
**ARB vs THZ**							
Monotherapy							
SIDIAP	6,752	4,956	1,817	1,341	12	8	3.55
VA-OMOP	77,046	55,457	26,662	18,412	175	124	1.39
CUIMC	2,641	1,425	274	156	12	<5	9.20
+ Combination							
SIDIAP	11,188	24,752	2,983	6,731	18	34	2.31
VA-OMOP	239,177	403,576	84,373	139,753	958	1,348	1.13
CUIMC	6,414	5,022	691	552	38	14	2.19

Pneumonia, acute respiratory distress syndrome, acute kidney injury or sepsis (PAAS)

Supplementary Table 4: Population size, total exposure time and PAAS (events) for ACEI, ARB and CCB/THZ monotherapy and in-combination user target (T) and comparator (C) cohorts.

	Patients		Time (years)		Events		
	T	C	T	C	T	C	MDRR
**ACEI/ARB vs CCB/THZ**							
Monotherapy							
SIDIAP	37,794	14,001	10,250	3,783	53	17	2.13
VA-OMOP	311,148	220,232	107,192	73,954	294	277	1.27
CUIMC	5,335	5,518	568	587	10	12	3.30
+ Combination							
SIDIAP	45,236	19,005	12,277	5,179	69	28	1.86
VA-OMOP	618,043	417,652	215,691	142,561	987	709	1.15
CUIMC	9,677	10,008	1,061	1,083	20	24	2.33
**ACEI/ARB vs CCB**							
Monotherapy							
SIDIAP	37,794	8,681	10,250	2,306	53	7	2.53
VA-OMOP	311,148	132,294	107,192	43,913	294	188	1.32
CUIMC	5,335	3,542	568	350	10	7	4.01
+ Combination							
SIDIAP	67,054	12,291	18,280	3,297	94	16	2.09
VA-OMOP	874,703	291,958	306,731	98,880	1,272	556	1.16
CUIMC	13,464	7,094	1,499	718	27	19	2.38
**ACEI/ARB vs THZ**							
Monotherapy							
SIDIAP	37,794	4,956	10,250	1,341	53	8	3.07
VA-OMOP	311,148	57,633	107,192	19,139	294	55	1.51
CUIMC	5,335	1,480	568	163	10	<5	20.88
+ Combination							
SIDIAP	50,204	7,303	13,582	1,982	76	11	2.46
VA-OMOP	836,337	177,597	293,383	60,219	1,472	223	1.20
CUIMC	12,577	3,700	1,345	409	31	7	2.96
**ACEI vs ARB**							
Monotherapy							
SIDIAP	30,786	6,752	8,302	1,817	40	12	2.75
VA-OMOP	228,356	80,641	78,366	27,923	211	82	1.45
CUIMC	2,471	2,765	265	288	<5	7	5.05
+ Combination							
SIDIAP	56,465	19,146	15,350	5,182	81	31	1.84
VA-OMOP	823,143	373,286	288,943	132,482	1,258	656	1.15
CUIMC	7,336	10,200	769	1,115	13	31	2.35

(Continued)	Patients		Time (years)		Events		
	T	C	T	C	T	C	MDRR
**ACEI vs CCB/THZ**							
Monotherapy							
SIDIAP	30,786	14,001	8,302	3,783	40	17	2.23
VA-OMOP	228,356	220,232	78,366	73,954	211	277	1.29
CUIMC	2,471	5,518	265	587	<5	12	4.35
+ Combination							
SIDIAP	36,321	29,235	9,812	7,949	52	41	1.79
VA-OMOP	431,744	604,621	150,022	209,740	663	1,017	1.15
CUIMC	4,492	15,017	480	1,623	6	39	2.70
**ACEI vs CCB**							
Monotherapy							
SIDIAP	30,786	8,681	8,302	2,306	40	7	2.68
VA-OMOP	228,356	132,294	78,366	43,913	211	188	1.34
CUIMC	2,471	3,542	265	350	<5	7	5.17
+ Combination							
SIDIAP	51,414	15,796	13,935	4,218	68	20	2.02
VA-OMOP	616,532	407,050	215,176	140,049	868	785	1.15
CUIMC	5,792	9,618	627	959	7	29	2.62
**ACEI vs THZ**							
Monotherapy							
SIDIAP	30,786	4,956	8,302	1,341	40	8	3.22
VA-OMOP	228,356	57,633	78,366	19,139	211	55	1.53
CUIMC	2,471	1,480	265	163	<5	<5	Inf
+ Combination							
SIDIAP	39,339	15,583	10,594	4,225	59	24	1.98
VA-OMOP	572,699	287,245	199,731	98,960	970	373	1.18
CUIMC	5,871	7,189	613	783	10	14	3.16
**ARB vs CCB/THZ**							
Monotherapy							
SIDIAP	6,752	14,001	1,817	3,783	12	17	3.04
VA-OMOP	80,641	220,232	27,923	73,954	82	277	1.40
CUIMC	2,765	5,518	288	587	7	12	3.91
+ Combination							
SIDIAP	9,193	39,427	2,459	10,725	17	57	2.30
VA-OMOP	188,830	811,540	65,956	281,947	334	1,256	1.20
CUIMC	5,366	13,031	593	1,404	14	30	2.53
**ARB vs CCB**							
Monotherapy							
SIDIAP	6,752	8,681	1,817	2,306	12	7	3.65
VA-OMOP	80,641	132,294	27,923	43,913	82	188	1.42
CUIMC	2,765	3,542	288	350	7	7	4.52

(Continued)	Patients		Time (years)		Events		
	T	C	T	C	T	C	MDRR
+ Combination							
SIDIAP	16,119	17,821	4,344	4,752	26	29	2.13
VA-OMOP	261,716	502,072	91,976	172,940	417	907	1.18
CUIMC	7,934	8,897	890	895	20	23	2.35
**ARB vs THZ**							
Monotherapy							
SIDIAP	6,752	4,956	1,817	1,341	12	8	3.55
VA-OMOP	80,641	57,633	27,923	19,139	82	55	1.62
CUIMC	2,765	1,480	288	163	7	<5	63.88
+ Combination							
SIDIAP	11,188	24,752	2,983	6,731	17	31	2.39
VA-OMOP	267,164	431,574	94,080	149,146	517	519	1.20
CUIMC	6,945	5,404	748	589	21	10	2.76

Baseline patient characteristics

SIDIAP

Supplementary Table 5: Baseline patient characteristcs for ACEI/ARB mono (T) and CCB/THZ mono (C) prevalent-users in the SIDIAP data source. We report proportion of initiators satisfying selected base-line characteristics and the standardized difference of population proportions (StdDiff) before and after stratification. Less extreme StdDiffs through stratification suggest improved balance between patient cohorts through propensity score adjustment.

	Before stratification			After stratification		
Characteristic	T (%)	C (%)	StdDiff	T (%)	C (%)	StdDiff
Age group						
15-19	0.0	0.0	-0.02	0.0	0.0	-0.02
20-24	0.1	0.1	0.00	0.1	0.1	-0.01
25-29	0.2	0.2	0.00	0.2	0.2	-0.01
30-34	0.6	0.6	0.01	0.6	0.6	0.00
35-39	1.6	1.0	0.04	1.4	1.3	0.01
40-44	3.8	2.3	0.08	3.4	3.3	0.01
45-49	7.0	5.0	0.08	6.4	6.5	0.00
50-54	10.4	7.9	0.09	9.6	9.9	-0.01
55-59	12.5	10.2	0.07	11.8	12.1	-0.01
60-64	13.7	12.5	0.03	13.4	13.2	0.00
65-69	13.4	13.4	0.00	13.5	13.2	0.01
70-74	12.9	14.6	-0.05	13.4	13.4	0.00
75-79	9.8	12.7	-0.09	10.7	10.5	0.01
80-84	6.8	9.0	-0.08	7.4	7.4	0.00
85-89	4.7	6.5	-0.08	5.2	5.2	0.00
90-94	2.1	2.9	-0.06	2.3	2.3	0.00
95-99	0.5	0.8	-0.04	0.5	0.7	-0.02
100-104	0.1	0.1	0.01	0.1	0.1	0.01
Gender: female	46.8	53.0	-0.12	48.9	47.8	0.02
Medical history: General						
Acute respiratory disease	8.6	8.4	0.01	8.6	8.5	0.00
Attention deficit hyperactivity disorder	0.1	0.1	0.00	0.1	0.1	0.00
Chronic liver disease	1.5	1.7	-0.02	1.6	1.7	-0.01
Chronic obstructive lung disease	6.0	7.0	-0.04	6.2	6.3	0.00
Crohn’s disease	0.2	0.2	0.00	0.2	0.2	0.01
Dementia	2.3	2.7	-0.03	2.5	2.3	0.01
Depressive disorder	13.6	14.8	-0.04	14.0	13.7	0.01
Diabetes mellitus	19.9	22.8	-0.07	20.7	20.4	0.01
Gastroesophageal reflux disease	10.0	11.5	-0.05	10.4	10.4	0.00
Gastrointestinal hemorrhage	0.7	0.8	-0.01	0.7	0.7	-0.01
Human immunodeficiency virus infection	0.5	0.4	0.02	0.5	0.4	0.01
Hyperlipidemia	26.7	27.9	-0.03	27.0	26.9	0.00
Hypertensive disorder	99.2	99.3	-0.01	99.2	99.3	-0.01
Lesion of liver	0.8	1.0	-0.03	0.8	0.9	-0.01
Obesity	34.6	37.5	-0.06	35.5	35.6	0.00

(Continued)	Before stratification			After stratification		
Characteristic	T (%)	C (%)	StdDiff	T (%)	C (%)	StdDiff
Osteoarthritis	27.9	33.2	-0.12	29.6	28.9	0.01
Pneumonia	0.7	0.6	0.01	0.7	0.6	0.02
Psoriasis	3.7	3.7	0.00	3.7	3.7	0.00
Renal impairment	8.1	13.8	-0.18	9.4	10.0	-0.02
Rheumatoid arthritis	0.4	0.5	0.00	0.5	0.4	0.01
Schizophrenia	0.5	0.5	0.00	0.5	0.5	-0.01
Ulcerative colitis	0.4	0.5	-0.01	0.4	0.4	0.00
Urinary tract infectious disease	4.9	5.3	-0.02	5.1	4.9	0.01
Viral hepatitis C	1.2	1.2	0.00	1.2	1.2	0.00
Visual system disorder	36.5	42.0	-0.11	38.1	37.5	0.01
Medical history: Cardiovascular disease						
Atrial fibrillation	3.8	4.6	-0.04	4.1	3.9	0.01
Cerebrovascular disease	2.1	2.4	-0.02	2.2	2.0	0.01
Coronary arteriosclerosis	0.0	0.0	0.01	0.0	0.0	0.01
Heart disease	20.7	24.9	-0.10	21.8	21.8	0.00
Heart failure	1.7	2.1	-0.03	1.8	1.7	0.00
Ischemic heart disease	3.9	4.7	-0.04	4.1	4.0	0.01
Peripheral vascular disease	2.7	4.3	-0.08	3.1	3.2	-0.01
Pulmonary embolism	0.5	0.6	-0.01	0.6	0.5	0.01
Venous thrombosis	1.0	1.1	-0.01	1.1	0.9	0.01
Medical history: Neoplasms						
Hematologic neoplasm	0.6	0.7	-0.01	0.6	0.6	0.00
Malignant lymphoma	0.4	0.4	-0.01	0.4	0.4	0.00
Malignant neoplasm of anorectum	0.4	0.4	-0.01	0.4	0.4	0.00
Malignant neoplastic disease	13.0	15.3	-0.06	13.6	13.6	0.00
Malignant tumor of breast	2.0	2.0	0.00	2.1	1.8	0.02
Malignant tumor of colon	1.4	1.5	-0.01	1.5	1.4	0.01
Malignant tumor of lung	0.1	0.2	-0.01	0.1	0.1	-0.01
Malignant tumor of urinary bladder	1.0	1.4	-0.03	1.1	1.1	0.00
Primary malignant neoplasm of prostate	2.0	2.2	-0.01	2.1	2.2	0.00
Medication use						
Antibacterials for systemic use	17.7	18.8	-0.03	18.0	17.8	0.00
Antidepressants	16.9	18.1	-0.03	17.3	17.0	0.01
Antiepileptics	7.0	8.0	-0.04	7.3	7.2	0.00
Antiinflammatory and antirheumatic products	21.9	20.9	0.03	21.7	21.3	0.01
Antineoplastic agents	0.5	0.6	-0.01	0.6	0.5	0.01
Antipsoriatics	0.7	1.0	-0.04	0.7	0.8	-0.01
Antithrombotic agents	18.2	22.3	-0.10	19.4	19.1	0.01
Drugs for acid related disorders	23.0	28.1	-0.12	24.5	24.0	0.01
Drugs for obstructive airway diseases	7.0	7.9	-0.03	7.3	7.1	0.00
Drugs used in diabetes	15.9	18.2	-0.06	16.6	16.2	0.01
Immunosuppressants	1.3	2.4	-0.08	1.5	1.8	-0.02
Lipid modifying agents	29.8	32.9	-0.07	30.7	30.7	0.00
Opioids	9.2	11.4	-0.07	9.8	9.8	0.00
Psycholeptics	26.5	29.2	-0.06	27.4	26.8	0.01

(Continued)	Before stratification			After stratification		
Characteristic	T (%)	C (%)	StdDiff	T (%)	C (%)	StdDiff
Psychostimulants, agents used for adhd and nootropics	1.2	1.4	-0.02	1.3	1.2	0.01

Supplementary Table 6: Baseline patient characteristcs for ACEI/ARB +combo (T) and CCB/THZ +combo (C) prevalent-users in the SIDIAP data source. We report proportion of initiators satisfying selected base-line characteristics and the standardized difference of population proportions (StdDiff) before and after stratification. Less extreme StdDiffs through stratification suggest improved balance between patient cohorts through propensity score adjustment.

	Before stratification			After stratification		
Characteristic	T (%)	C (%)	StdDiff	T (%)	C (%)	StdDiff
Age group						
15-19	0.0	0.0	-0.01	0.0	0.0	-0.01
20-24	0.1	0.1	-0.01	0.1	0.1	-0.01
25-29	0.2	0.2	0.01	0.2	0.2	0.00
30-34	0.5	0.5	0.01	0.5	0.5	0.01
35-39	1.4	1.0	0.04	1.3	1.2	0.01
40-44	3.4	2.3	0.07	3.0	3.0	0.00
45-49	6.3	4.7	0.07	5.8	6.0	-0.01
50-54	9.5	7.7	0.07	8.9	9.1	-0.01
55-59	11.7	10.1	0.05	11.1	11.3	-0.01
60-64	13.0	12.2	0.03	12.7	12.6	0.00
65-69	13.1	13.0	0.00	13.2	12.9	0.01
70-74	13.0	14.6	-0.04	13.5	13.6	0.00
75-79	10.4	13.0	-0.08	11.3	11.0	0.01
80-84	7.8	9.3	-0.05	8.3	8.2	0.00
85-89	5.9	7.2	-0.05	6.3	6.3	0.00
90-94	2.8	3.3	-0.03	3.0	2.9	0.00
95-99	0.7	0.9	-0.03	0.7	0.8	-0.01
100-104	0.1	0.1	0.01	0.1	0.1	0.01
Gender: female	47.2	52.6	-0.11	49.3	48.1	0.02
Medical history: General						
Acute respiratory disease	9.1	8.8	0.01	9.1	9.0	0.00
Attention deficit hyperactivity disorder	0.1	0.1	0.00	0.1	0.1	-0.01
Chronic liver disease	1.7	2.0	-0.02	1.8	1.9	-0.01
Chronic obstructive lung disease	7.0	7.4	-0.02	7.1	7.2	0.00
Crohn's disease	0.2	0.2	0.00	0.2	0.2	0.00
Dementia	2.8	2.9	0.00	2.9	2.7	0.01
Depressive disorder	14.4	15.4	-0.03	14.8	14.5	0.01
Diabetes mellitus	21.5	24.5	-0.07	22.3	22.3	0.00
Gastroesophageal reflux disease	10.3	11.7	-0.04	10.8	10.8	0.00
Gastrointestinal hemorrhage	0.7	0.8	-0.01	0.7	0.8	0.00
Human immunodeficiency virus infection	0.5	0.3	0.02	0.5	0.4	0.01
Hyperlipidemia	27.2	28.2	-0.02	27.5	27.4	0.00
Hypertensive disorder	99.2	99.4	-0.02	99.2	99.3	-0.01
Lesion of liver	1.0	1.4	-0.03	1.1	1.2	-0.01
Obesity	35.7	38.6	-0.06	36.7	36.6	0.00
Osteoarthritis	29.6	33.9	-0.09	31.2	30.4	0.02
Pneumonia	0.8	0.7	0.01	0.8	0.7	0.01
Psoriasis	3.6	3.6	0.00	3.7	3.7	0.00
Renal impairment	10.4	16.3	-0.18	11.9	12.4	-0.01

(Continued)	Before stratification			After stratification		
Characteristic	T (%)	C (%)	StdDiff	T (%)	C (%)	StdDiff
Rheumatoid arthritis	0.5	0.6	-0.01	0.5	0.5	0.01
Schizophrenia	0.5	0.5	0.00	0.5	0.5	0.00
Ulcerative colitis	0.4	0.5	-0.01	0.4	0.4	0.00
Urinary tract infectious disease	5.3	5.3	0.00	5.4	5.3	0.00
Viral hepatitis C	1.2	1.3	-0.01	1.2	1.3	0.00
Visual system disorder	38.2	42.5	-0.09	39.6	39.1	0.01
Medical history: Cardiovascular disease						
Atrial fibrillation	6.9	7.1	-0.01	7.0	6.9	0.00
Cerebrovascular disease	2.3	2.6	-0.01	2.4	2.3	0.01
Coronary arteriosclerosis	0.0	0.0	0.00	0.0	0.0	0.00
Heart disease	27.4	29.4	-0.04	28.0	27.9	0.00
Heart failure	4.3	4.0	0.02	4.2	4.1	0.00
Ischemic heart disease	7.0	6.9	0.00	7.0	6.8	0.01
Peripheral vascular disease	3.4	4.7	-0.06	3.8	3.8	0.00
Pulmonary embolism	0.7	0.7	0.00	0.7	0.6	0.01
Venous thrombosis	1.2	1.2	-0.01	1.2	1.1	0.01
Medical history: Neoplasms						
Hematologic neoplasm	0.6	0.8	-0.01	0.7	0.7	0.00
Malignant lymphoma	0.4	0.5	-0.01	0.4	0.4	0.00
Malignant neoplasm of anorectum	0.4	0.4	-0.01	0.4	0.4	0.00
Malignant neoplastic disease	14.0	15.7	-0.05	14.6	14.4	0.00
Malignant tumor of breast	2.1	2.1	0.00	2.2	2.0	0.01
Malignant tumor of colon	1.5	1.7	-0.01	1.6	1.5	0.01
Malignant tumor of lung	0.1	0.1	0.00	0.1	0.1	0.00
Malignant tumor of urinary bladder	1.2	1.4	-0.02	1.2	1.2	0.00
Primary malignant neoplasm of prostate	2.2	2.3	0.00	2.2	2.3	0.00
Medication use						
Antibacterials for systemic use	18.9	19.8	-0.02	19.2	19.2	0.00
Antidepressants	18.1	19.2	-0.03	18.6	18.1	0.01
Antiepileptics	7.6	8.5	-0.03	7.9	7.8	0.00
Antiinflammatory and antirheumatic products	20.9	20.2	0.02	20.8	20.5	0.01
Antineoplastic agents	0.5	0.6	-0.01	0.6	0.5	0.01
Antipsoriatics	0.8	1.2	-0.04	0.8	1.0	-0.02
Antithrombotic agents	24.1	26.1	-0.04	24.8	24.5	0.01
Drugs for acid related disorders	25.9	30.1	-0.09	27.3	26.8	0.01
Drugs for obstructive airway diseases	7.5	8.1	-0.02	7.7	7.6	0.00
Drugs used in diabetes	17.2	19.6	-0.06	17.9	17.8	0.00
Immunosuppressants	1.4	2.5	-0.08	1.6	2.0	-0.03
Lipid modifying agents	32.5	34.5	-0.04	33.1	33.1	0.00
Opioids	10.2	12.1	-0.06	10.9	10.6	0.01
Psycholeptics	28.3	30.9	-0.06	29.3	28.7	0.01
Psychostimulants, agents used for adhd and nootropics	2.0	2.0	0.00	2.0	2.0	0.01

Supplementary Table 7: Baseline patient characteristcs for ACEI/ARB mono (T) and CCB mono (C) prevalent-users in the SIDIAP data source. We report proportion of initiators satisfying selected base-line characteristics and the standardized difference of population proportions (StdDiff) before and after stratification. Less extreme StdDiffs through stratification suggest improved balance between patient cohorts through propensity score adjustment.

	Before stratification			After stratification		
Characteristic	T (%)	C (%)	StdDiff	T (%)	C (%)	StdDiff
Age group						
15-19	0.0	<0.1	-0.01	0.0	<0.1	-0.01
20-24	0.1	0.2	-0.02	0.1	0.2	-0.03
25-29	0.2	0.3	-0.01	0.2	0.3	-0.02
30-34	0.6	0.6	0.00	0.6	0.8	-0.03
35-39	1.6	1.1	0.04	1.5	1.3	0.01
40-44	3.8	2.5	0.07	3.5	3.4	0.01
45-49	7.0	5.0	0.08	6.6	6.5	0.00
50-54	10.4	8.0	0.09	9.9	10.0	0.00
55-59	12.5	10.6	0.06	12.1	12.1	0.00
60-64	13.7	12.4	0.04	13.5	13.1	0.01
65-69	13.4	13.1	0.01	13.3	13.2	0.00
70-74	12.9	14.1	-0.03	13.2	13.2	0.00
75-79	9.8	12.3	-0.08	10.3	10.2	0.00
80-84	6.8	9.1	-0.08	7.2	7.4	-0.01
85-89	4.7	6.9	-0.10	5.1	5.2	0.00
90-94	2.1	2.9	-0.05	2.2	2.2	0.00
95-99	0.5	0.9	-0.05	0.5	0.7	-0.02
100-104	0.1	<0.1	0.01	0.1	<0.1	0.02
Gender: female	46.8	44.4	0.05	46.4	46.3	0.00
Medical history: General						
Acute respiratory disease	8.6	8.5	0.00	8.6	8.6	0.00
Attention deficit hyperactivity disorder	0.1	0.1	0.00	0.1	0.1	-0.01
Chronic liver disease	1.5	2.1	-0.04	1.6	1.8	-0.01
Chronic obstructive lung disease	6.0	7.8	-0.07	6.3	6.4	0.00
Crohn’s disease	0.2	0.1	0.01	0.2	0.1	0.01
Dementia	2.3	2.8	-0.03	2.5	2.4	0.00
Depressive disorder	13.6	14.6	-0.03	13.8	13.5	0.01
Diabetes mellitus	19.9	26.5	-0.16	21.1	20.6	0.01
Gastroesophageal reflux disease	10.0	11.4	-0.04	10.3	10.2	0.00
Gastrointestinal hemorrhage	0.7	1.0	-0.03	0.7	0.9	-0.02
Human immunodeficiency virus infection	0.5	0.4	0.02	0.5	0.3	0.03
Hyperlipidemia	26.7	27.9	-0.03	27.0	26.6	0.01
Hypertensive disorder	99.2	99.4	-0.02	99.2	99.4	-0.02
Lesion of liver	0.8	1.3	-0.05	0.9	1.0	-0.01
Obesity	34.6	37.7	-0.06	35.3	35.0	0.01
Osteoarthritis	27.9	31.2	-0.07	28.7	28.2	0.01
Pneumonia	0.7	0.6	0.01	0.7	0.6	0.01
Psoriasis	3.7	3.8	0.00	3.7	3.7	0.00
Renal impairment	8.1	17.6	-0.29	9.5	10.4	-0.03

(Continued)	Before stratification			After stratification		
Characteristic	T (%)	C (%)	StdDiff	T (%)	C (%)	StdDiff
Rheumatoid arthritis	0.4	0.4	0.00	0.5	0.4	0.02
Schizophrenia	0.5	0.6	-0.01	0.5	0.6	-0.01
Ulcerative colitis	0.4	0.5	-0.01	0.4	0.4	0.00
Urinary tract infectious disease	4.9	5.0	0.00	5.0	4.8	0.01
Viral hepatitis C	1.2	1.5	-0.03	1.2	1.3	-0.01
Visual system disorder	36.5	41.8	-0.11	37.5	37.2	0.01
Medical history: Cardiovascular disease						
Atrial fibrillation	3.8	5.4	-0.08	4.1	4.0	0.00
Cerebrovascular disease	2.1	2.9	-0.05	2.2	2.2	0.01
Coronary arteriosclerosis	0.0	<0.1	0.01	0.0	<0.1	0.01
Heart disease	20.7	27.8	-0.17	22.0	21.8	0.01
Heart failure	1.7	2.8	-0.07	1.9	1.9	0.00
Ischemic heart disease	3.9	6.2	-0.11	4.3	4.3	0.00
Peripheral vascular disease	2.7	5.4	-0.14	3.1	3.5	-0.02
Pulmonary embolism	0.5	0.6	-0.01	0.6	0.5	0.01
Venous thrombosis	1.0	1.1	-0.01	1.0	0.9	0.01
Medical history: Neoplasms						
Hematologic neoplasm	0.6	0.8	-0.03	0.6	0.6	-0.01
Malignant lymphoma	0.4	0.4	-0.01	0.4	0.4	0.00
Malignant neoplasm of anorectum	0.4	0.5	-0.02	0.4	0.4	0.00
Malignant neoplastic disease	13.0	15.8	-0.08	13.5	13.6	0.00
Malignant tumor of breast	2.0	1.6	0.03	1.9	1.7	0.02
Malignant tumor of colon	1.4	1.5	-0.01	1.5	1.3	0.02
Malignant tumor of lung	0.1	0.2	-0.02	0.1	0.2	-0.01
Malignant tumor of urinary bladder	1.0	1.8	-0.06	1.1	1.2	-0.01
Primary malignant neoplasm of prostate	2.0	2.5	-0.03	2.2	2.2	0.00
Medication use						
Antibacterials for systemic use	17.7	18.9	-0.03	17.9	18.0	0.00
Antidepressants	16.9	17.9	-0.03	17.2	16.9	0.01
Antiepileptics	7.0	8.1	-0.04	7.2	7.2	0.00
Antiinflammatory and antirheumatic products	21.9	18.8	0.08	21.4	21.5	0.00
Antineoplastic agents	0.5	0.5	0.00	0.5	0.4	0.02
Antipsoriatics	0.7	1.2	-0.06	0.7	0.9	-0.02
Antithrombotic agents	18.2	25.7	-0.18	19.7	19.5	0.00
Drugs for acid related disorders	23.0	29.6	-0.15	24.3	23.7	0.01
Drugs for obstructive airway diseases	7.0	7.6	-0.02	7.2	7.0	0.01
Drugs used in diabetes	15.9	21.3	-0.14	17.0	16.3	0.02
Immunosuppressants	1.3	2.8	-0.10	1.5	1.9	-0.03
Lipid modifying agents	29.8	35.3	-0.12	31.0	30.7	0.00
Opioids	9.2	11.4	-0.07	9.6	9.6	0.00
Psycholeptics	26.5	29.1	-0.06	27.0	26.6	0.01
Psychostimulants, agents used for adhd and nootropics	1.2	1.8	-0.05	1.4	1.3	0.00

Supplementary Table 8: Baseline patient characteristcs for ACEI/ARB +combo (T) and CCB +combo (C) prevalent-users in the SIDIAP data source. We report proportion of initiators satisfying selected base-line characteristics and the standardized difference of population proportions (StdDiff) before and after stratification. Less extreme StdDiffs through stratification suggest improved balance between patient cohorts through propensity score adjustment.

	Before stratification			After stratification		
Characteristic	T (%)	C (%)	StdDiff	T (%)	C (%)	StdDiff
Age group						
15-19	0.0	0.0	-0.01	0.0	0.0	-0.01
20-24	0.1	0.1	-0.03	0.1	0.2	-0.04
25-29	0.2	0.2	0.00	0.2	0.2	-0.01
30-34	0.5	0.5	-0.01	0.4	0.6	-0.02
35-39	1.3	1.1	0.02	1.2	1.2	0.00
40-44	3.1	2.5	0.04	3.0	2.9	0.01
45-49	6.2	4.8	0.06	6.0	5.9	0.00
50-54	9.4	7.6	0.06	9.1	9.2	0.00
55-59	11.6	9.9	0.05	11.3	11.2	0.00
60-64	13.3	11.9	0.04	13.1	13.0	0.00
65-69	13.4	12.9	0.01	13.4	13.2	0.00
70-74	13.6	14.1	-0.01	13.6	13.4	0.01
75-79	10.8	12.7	-0.06	11.1	11.2	0.00
80-84	7.7	9.6	-0.07	8.0	8.2	-0.01
85-89	5.6	7.5	-0.07	6.0	6.0	0.00
90-94	2.5	3.4	-0.06	2.6	2.7	0.00
95-99	0.6	1.0	-0.04	0.6	0.7	-0.01
100-104	0.1	0.1	0.00	0.1	0.1	0.01
Gender: female	47.7	44.7	0.06	47.3	47.3	0.00
Medical history: General						
Acute respiratory disease	8.9	8.9	0.00	9.0	9.0	0.00
Attention deficit hyperactivity disorder	0.1	0.1	0.00	0.1	0.1	0.00
Chronic liver disease	1.7	2.3	-0.04	1.8	1.9	-0.01
Chronic obstructive lung disease	6.8	8.3	-0.06	7.1	7.1	0.00
Crohn's disease	0.2	0.2	0.00	0.2	0.2	0.00
Dementia	2.4	3.0	-0.03	2.5	2.5	0.00
Depressive disorder	14.3	15.1	-0.02	14.5	14.1	0.01
Diabetes mellitus	22.0	28.0	-0.14	22.9	22.8	0.00
Gastroesophageal reflux disease	10.5	11.7	-0.04	10.7	10.7	0.00
Gastrointestinal hemorrhage	0.7	1.0	-0.03	0.8	0.8	-0.01
Human immunodeficiency virus infection	0.4	0.4	0.01	0.4	0.3	0.02
Hyperlipidemia	27.8	28.3	-0.01	28.0	27.7	0.00
Hypertensive disorder	99.3	99.5	-0.02	99.3	99.5	-0.02
Lesion of liver	1.0	1.6	-0.05	1.1	1.1	-0.01
Obesity	38.0	38.8	-0.02	38.1	38.4	0.00
Osteoarthritis	30.0	32.3	-0.05	30.5	30.3	0.00
Pneumonia	0.8	0.8	-0.01	0.8	0.7	0.01
Psoriasis	3.7	3.7	0.00	3.7	3.6	0.00
Renal impairment	10.0	20.4	-0.29	11.4	12.2	-0.02

(Continued)	Before stratification			After stratification		
Characteristic	T (%)	C (%)	StdDiff	T (%)	C (%)	StdDiff
Rheumatoid arthritis	0.5	0.5	0.00	0.5	0.4	0.01
Schizophrenia	0.5	0.5	-0.01	0.5	0.5	-0.01
Ulcerative colitis	0.4	0.5	-0.01	0.4	0.4	0.00
Urinary tract infectious disease	5.1	5.0	0.00	5.1	5.0	0.00
Viral hepatitis C	1.3	1.5	-0.02	1.3	1.3	0.00
Visual system disorder	38.3	42.5	-0.09	39.0	38.9	0.00
Medical history: Cardiovascular disease						
Atrial fibrillation	6.2	7.8	-0.06	6.5	6.5	0.00
Cerebrovascular disease	2.3	3.1	-0.05	2.4	2.3	0.00
Coronary arteriosclerosis	0.0	0.0	-0.01	0.0	0.0	0.00
Heart disease	26.7	32.8	-0.13	27.6	27.6	0.00
Heart failure	3.5	4.9	-0.07	3.7	3.7	0.00
Ischemic heart disease	6.3	8.7	-0.09	6.7	6.5	0.01
Peripheral vascular disease	3.4	6.1	-0.13	3.7	4.0	-0.01
Pulmonary embolism	0.6	0.7	-0.02	0.6	0.6	0.00
Venous thrombosis	1.2	1.3	-0.01	1.2	1.1	0.01
Medical history: Neoplasms						
Hematologic neoplasm	0.6	0.8	-0.04	0.6	0.6	-0.01
Malignant lymphoma	0.4	0.5	-0.01	0.4	0.4	0.00
Malignant neoplasm of anorectum	0.4	0.5	-0.01	0.4	0.4	0.00
Malignant neoplastic disease	13.6	16.6	-0.08	14.1	14.1	0.00
Malignant tumor of breast	2.0	1.8	0.02	2.0	1.8	0.01
Malignant tumor of colon	1.5	1.8	-0.02	1.6	1.5	0.01
Malignant tumor of lung	0.1	0.2	-0.01	0.1	0.1	0.00
Malignant tumor of urinary bladder	1.1	1.7	-0.05	1.2	1.2	0.00
Primary malignant neoplasm of prostate	2.1	2.7	-0.04	2.2	2.3	0.00
Medication use						
Antibacterials for systemic use	18.4	20.2	-0.04	18.6	19.1	-0.01
Antidepressants	17.9	18.8	-0.02	18.1	17.8	0.01
Antiepileptics	7.6	8.8	-0.04	7.8	7.8	0.00
Antiinflammatory and antirheumatic products	20.8	18.2	0.07	20.5	20.6	0.00
Antineoplastic agents	0.5	0.5	0.00	0.5	0.4	0.03
Antipsoriatics	0.7	1.5	-0.07	0.8	1.0	-0.03
Antithrombotic agents	23.3	29.9	-0.15	24.4	24.1	0.01
Drugs for acid related disorders	25.8	32.0	-0.14	26.8	26.3	0.01
Drugs for obstructive airway diseases	7.3	8.1	-0.03	7.4	7.5	0.00
Drugs used in diabetes	17.7	22.7	-0.13	18.4	18.3	0.00
Immunosuppressants	1.3	3.1	-0.12	1.4	2.0	-0.05
Lipid modifying agents	33.1	37.1	-0.08	33.7	33.7	0.00
Opioids	10.5	12.3	-0.06	10.8	10.7	0.00
Psycholeptics	28.3	30.8	-0.06	28.7	28.4	0.01
Psychostimulants, agents used for adhd and nootropics	1.8	2.6	-0.05	1.9	1.9	0.00

Supplementary Table 9: Baseline patient characteristcs for ACEI/ARB mono (T) and THZ mono (C) prevalent-users in the SIDIAP data source. We report proportion of initiators satisfying selected base-line characteristics and the standardized difference of population proportions (StdDiff) before and after stratification. Less extreme StdDiffs through stratification suggest improved balance between patient cohorts through propensity score adjustment.

	Before stratification			After stratification		
Characteristic	T (%)	C (%)	StdDiff	T (%)	C (%)	StdDiff
Age group						
15-19	0.0	<0.1	-0.02	0.0	0.1	-0.03
20-24	0.1	<0.1	0.03	0.1	<0.1	0.03
25-29	0.2	0.1	0.02	0.2	0.2	0.02
30-34	0.6	0.5	0.02	0.6	0.5	0.01
35-39	1.6	1.0	0.05	1.5	1.1	0.04
40-44	3.8	2.0	0.11	3.6	3.3	0.02
45-49	7.0	5.0	0.08	6.7	6.9	-0.01
50-54	10.4	7.8	0.09	10.1	9.8	0.01
55-59	12.5	9.8	0.09	12.2	11.8	0.01
60-64	13.7	12.7	0.03	13.6	12.8	0.02
65-69	13.4	13.7	-0.01	13.4	13.6	-0.01
70-74	12.9	15.5	-0.07	13.2	13.2	0.00
75-79	9.8	13.5	-0.12	10.2	10.9	-0.02
80-84	6.8	8.7	-0.07	7.0	7.4	-0.01
85-89	4.7	6.0	-0.06	4.8	5.1	-0.02
90-94	2.1	2.8	-0.05	2.1	2.5	-0.03
95-99	0.5	0.7	-0.03	0.5	0.7	-0.03
100-104	0.1	<0.1	0.00	0.1	<0.1	0.00
Gender: female	46.8	67.6	-0.43	49.4	48.2	0.02
Medical history: General						
Acute respiratory disease	8.6	8.2	0.02	8.6	8.4	0.01
Attention deficit hyperactivity disorder	0.1	<0.1	0.01	0.1	<0.1	0.00
Chronic liver disease	1.5	1.1	0.04	1.5	1.3	0.02
Chronic obstructive lung disease	6.0	5.5	0.02	5.9	6.7	-0.03
Crohn's disease	0.2	0.2	-0.01	0.2	0.2	-0.01
Dementia	2.3	2.7	-0.02	2.4	2.7	-0.02
Depressive disorder	13.6	14.9	-0.04	13.8	14.0	0.00
Diabetes mellitus	19.9	16.4	0.09	19.5	19.5	0.00
Gastroesophageal reflux disease	10.0	11.5	-0.05	10.2	10.3	0.00
Gastrointestinal hemorrhage	0.7	0.6	0.01	0.7	0.7	0.00
Human immunodeficiency virus infection	0.5	0.3	0.03	0.5	0.5	0.00
Hyperlipidemia	26.7	27.6	-0.02	26.8	26.3	0.01
Hypertensive disorder	99.2	99.2	0.01	99.2	99.3	0.00
Lesion of liver	0.8	0.7	0.02	0.8	0.7	0.02
Obesity	34.6	37.1	-0.05	34.9	35.0	0.00
Osteoarthritis	27.9	36.4	-0.18	28.9	28.5	0.01
Pneumonia	0.7	0.5	0.02	0.7	0.6	0.02
Psoriasis	3.7	3.4	0.01	3.7	3.7	0.00
Renal impairment	8.1	7.3	0.03	8.0	8.3	-0.01

(Continued)	Before stratification			After stratification		
Characteristic	T (%)	C (%)	StdDiff	T (%)	C (%)	StdDiff
Rheumatoid arthritis	0.4	0.6	-0.02	0.5	0.4	0.01
Schizophrenia	0.5	0.4	0.01	0.5	0.5	0.00
Ulcerative colitis	0.4	0.4	0.00	0.4	0.4	0.01
Urinary tract infectious disease	4.9	5.6	-0.03	5.1	4.9	0.01
Viral hepatitis C	1.2	0.8	0.04	1.1	1.0	0.01
Visual system disorder	36.5	42.4	-0.12	37.2	37.6	-0.01
Medical history: Cardiovascular disease						
Atrial fibrillation	3.8	3.3	0.03	3.8	3.7	0.01
Cerebrovascular disease	2.1	1.6	0.04	2.1	1.8	0.02
Coronary arteriosclerosis	0.0	0.0		0.0	0.0	
Heart disease	20.7	19.8	0.02	20.6	20.7	0.00
Heart failure	1.7	0.9	0.06	1.6	1.5	0.01
Ischemic heart disease	3.9	2.1	0.10	3.7	3.9	-0.01
Peripheral vascular disease	2.7	2.1	0.04	2.7	2.7	0.00
Pulmonary embolism	0.5	0.5	0.00	0.5	0.5	0.01
Venous thrombosis	1.0	1.1	-0.01	1.0	1.0	0.00
Medical history: Neoplasms						
Hematologic neoplasm	0.6	0.5	0.01	0.6	0.4	0.03
Malignant lymphoma	0.4	0.4	0.00	0.4	0.3	0.00
Malignant neoplasm of anorectum	0.4	0.3	0.01	0.4	0.3	0.01
Malignant neoplastic disease	13.0	14.0	-0.03	13.1	12.9	0.01
Malignant tumor of breast	2.0	2.8	-0.05	2.1	1.9	0.01
Malignant tumor of colon	1.4	1.5	-0.01	1.4	1.3	0.00
Malignant tumor of lung	0.1	0.1	-0.01	0.1	0.1	0.00
Malignant tumor of urinary bladder	1.0	0.7	0.04	1.0	0.8	0.02
Primary malignant neoplasm of prostate	2.0	1.6	0.04	2.0	2.3	-0.02
Medication use						
Antibacterials for systemic use	17.7	18.7	-0.03	17.8	17.5	0.01
Antidepressants	16.9	18.7	-0.04	17.2	17.2	0.00
Antiepileptics	7.0	7.8	-0.03	7.1	7.3	-0.01
Antiinflammatory and antirheumatic products	21.9	24.7	-0.07	22.2	21.4	0.02
Antineoplastic agents	0.5	0.8	-0.04	0.5	0.7	-0.02
Antipsoriatics	0.7	0.6	0.00	0.7	0.7	0.00
Antithrombotic agents	18.2	16.3	0.05	18.0	18.1	0.00
Drugs for acid related disorders	23.0	25.4	-0.06	23.3	22.9	0.01
Drugs for obstructive airway diseases	7.0	8.1	-0.04	7.1	7.6	-0.02
Drugs used in diabetes	15.9	12.7	0.09	15.5	15.4	0.00
Immunosuppressants	1.3	1.6	-0.02	1.3	1.5	-0.01
Lipid modifying agents	29.8	28.4	0.03	29.7	30.0	-0.01
Opioids	9.2	11.2	-0.07	9.5	9.3	0.01
Psycholeptics	26.5	29.6	-0.07	26.9	26.8	0.00
Psychostimulants, agents used for adhd and nootropics	1.2	0.6	0.06	1.2	1.0	0.02

Supplementary Table 10: Baseline patient characteristcs for ACEI/ARB +combo (T) and THZ +combo (C) prevalent-users in the SIDIAP data source. We report proportion of initiators satisfying selected base-line characteristics and the standardized difference of population proportions (StdDiff) before and after stratification. Less extreme StdDiffs through stratification suggest improved balance between patient cohorts through propensity score adjustment.

	Before stratification			After stratification		
Characteristic	T (%)	C (%)	StdDiff	T (%)	C (%)	StdDiff
Age group						
15-19	0.0	<0.1	-0.01	0.0	<0.1	-0.02
20-24	0.1	<0.1	0.02	0.1	<0.1	0.02
25-29	0.2	0.1	0.02	0.2	0.1	0.02
30-34	0.5	0.3	0.03	0.5	0.4	0.03
35-39	1.4	0.9	0.04	1.4	1.0	0.03
40-44	3.3	2.1	0.08	3.2	2.9	0.02
45-49	6.2	4.6	0.07	6.0	6.2	-0.01
50-54	9.4	7.8	0.06	9.2	8.9	0.01
55-59	11.5	10.2	0.04	11.4	10.9	0.02
60-64	12.9	12.6	0.01	13.0	12.3	0.02
65-69	13.1	13.3	-0.01	13.1	12.5	0.02
70-74	13.2	15.4	-0.06	13.3	14.7	-0.04
75-79	10.6	13.3	-0.08	10.9	11.4	-0.01
80-84	7.9	8.8	-0.04	8.0	8.2	-0.01
85-89	6.1	6.5	-0.01	6.2	6.2	0.00
90-94	2.9	3.1	-0.01	2.9	3.2	-0.02
95-99	0.7	0.9	-0.02	0.7	0.9	-0.03
100-104	0.1	0.1	0.01	0.1	0.1	0.01
Gender: female	46.3	66.0	-0.40	49.0	47.8	0.02
Medical history: General						
Acute respiratory disease	9.1	8.8	0.01	9.1	8.9	0.01
Attention deficit hyperactivity disorder	0.1	0.1	0.00	0.1	0.1	0.00
Chronic liver disease	1.8	1.7	0.01	1.7	1.9	-0.01
Chronic obstructive lung disease	7.2	5.9	0.05	7.0	7.4	-0.01
Crohn's disease	0.2	0.2	0.00	0.2	0.2	0.00
Dementia	2.8	2.6	0.01	2.8	2.8	0.00
Depressive disorder	14.4	15.7	-0.04	14.7	14.9	-0.01
Diabetes mellitus	22.2	18.7	0.09	21.8	22.3	-0.01
Gastroesophageal reflux disease	10.3	11.8	-0.05	10.5	10.8	-0.01
Gastrointestinal hemorrhage	0.7	0.6	0.02	0.7	0.7	0.00
Human immunodeficiency virus infection	0.5	0.3	0.03	0.5	0.4	0.00
Hyperlipidemia	27.3	28.1	-0.02	27.3	27.4	0.00
Hypertensive disorder	99.3	99.2	0.00	99.2	99.4	-0.03
Lesion of liver	1.0	0.9	0.01	1.0	1.1	-0.01
Obesity	36.1	38.6	-0.05	36.3	36.7	-0.01
Osteoarthritis	29.7	36.5	-0.14	30.6	30.5	0.00
Pneumonia	0.8	0.7	0.02	0.8	0.8	0.00
Psoriasis	3.7	3.5	0.01	3.7	3.6	0.00
Renal impairment	11.1	9.3	0.06	10.9	11.7	-0.03

(Continued)	Before stratification			After stratification		
Characteristic	T (%)	C (%)	StdDiff	T (%)	C (%)	StdDiff
Rheumatoid arthritis	0.5	0.7	-0.03	0.5	0.5	0.00
Schizophrenia	0.5	0.4	0.01	0.5	0.5	0.00
Ulcerative colitis	0.4	0.4	-0.01	0.4	0.5	-0.01
Urinary tract infectious disease	5.4	5.7	-0.01	5.4	5.4	0.00
Viral hepatitis C	1.3	1.1	0.01	1.2	1.2	0.00
Visual system disorder	38.3	42.6	-0.09	38.8	39.4	-0.01
Medical history: Cardiovascular disease						
Atrial fibrillation	7.1	5.6	0.06	6.9	7.1	-0.01
Cerebrovascular disease	2.4	1.7	0.06	2.4	2.2	0.01
Coronary arteriosclerosis	0.0	<0.1	0.01	0.0	<0.1	-0.01
Heart disease	28.2	23.8	0.10	27.7	27.7	0.00
Heart failure	4.4	2.3	0.12	4.2	4.0	0.01
Ischemic heart disease	7.4	3.9	0.15	7.0	6.7	0.01
Peripheral vascular disease	3.7	2.5	0.07	3.5	3.7	-0.01
Pulmonary embolism	0.7	0.6	0.01	0.7	0.6	0.01
Venous thrombosis	1.2	1.2	0.00	1.2	1.2	0.00
Medical history: Neoplasms						
Hematologic neoplasm	0.7	0.6	0.00	0.7	0.6	0.00
Malignant lymphoma	0.5	0.5	0.00	0.5	0.5	0.00
Malignant neoplasm of anorectum	0.4	0.3	0.00	0.4	0.4	-0.01
Malignant neoplastic disease	14.3	14.3	0.00	14.3	14.2	0.00
Malignant tumor of breast	2.0	2.8	-0.05	2.1	2.0	0.01
Malignant tumor of colon	1.6	1.5	0.01	1.6	1.5	0.00
Malignant tumor of lung	0.1	0.1	0.01	0.1	0.1	0.01
Malignant tumor of urinary bladder	1.2	0.7	0.05	1.2	1.0	0.02
Primary malignant neoplasm of prostate	2.3	1.6	0.05	2.2	2.4	-0.01
Medication use						
Antibacterials for systemic use	19.0	18.9	0.00	19.0	18.8	0.00
Antidepressants	18.1	19.8	-0.04	18.3	18.4	0.00
Antiepileptics	7.7	8.1	-0.01	7.8	7.7	0.00
Antiinflammatory and antirheumatic products	20.5	23.7	-0.08	20.8	20.7	0.00
Antineoplastic agents	0.5	0.7	-0.02	0.5	0.6	-0.01
Antipsoriatics	0.8	0.8	0.00	0.8	1.0	-0.02
Antithrombotic agents	25.1	19.8	0.13	24.5	24.5	0.00
Drugs for acid related disorders	26.5	26.8	-0.01	26.6	26.3	0.01
Drugs for obstructive airway diseases	7.5	8.2	-0.03	7.6	8.1	-0.02
Drugs used in diabetes	17.8	14.4	0.09	17.4	17.7	-0.01
Immunosuppressants	1.5	1.6	-0.01	1.5	1.6	-0.01
Lipid modifying agents	33.2	30.3	0.06	32.8	33.0	0.00
Opioids	10.3	11.8	-0.05	10.6	10.3	0.01
Psycholeptics	28.5	31.0	-0.05	28.9	29.1	0.00
Psychostimulants, agents used for adhd and nootropics	2.1	1.1	0.08	2.0	1.6	0.03

Supplementary Table 11: Baseline patient characteristcs for ACEI mono (T) and ARB mono (C) prevalent-users in the SIDIAP data source. We report proportion of initiators satisfying selected base-line characteristics and the standardized difference of population proportions (StdDiff) before and after stratification. Less extreme StdDiffs through stratification suggest improved balance between patient cohorts through propensity score adjustment.

	Before stratification			After stratification		
Characteristic	T (%)	C (%)	StdDiff	T (%)	C (%)	StdDiff
Age group						
15-19	0.0	0.0		0.0	0.0	
20-24	0.1	0.1	0.00	0.1	0.1	0.00
25-29	0.3	0.1	0.03	0.2	0.1	0.03
30-34	0.7	0.4	0.04	0.6	0.4	0.03
35-39	1.7	1.0	0.06	1.6	1.2	0.04
40-44	4.0	2.7	0.07	3.8	3.6	0.01
45-49	7.5	4.6	0.12	6.9	7.0	0.00
50-54	10.9	8.0	0.10	10.4	10.4	0.00
55-59	12.8	10.9	0.06	12.5	12.6	0.00
60-64	13.7	13.3	0.01	13.6	14.0	-0.01
65-69	13.1	14.5	-0.04	13.4	13.5	0.00
70-74	12.4	15.1	-0.08	12.9	12.9	0.00
75-79	9.3	11.9	-0.08	9.8	9.9	0.00
80-84	6.5	8.3	-0.07	6.8	6.8	0.00
85-89	4.4	5.9	-0.07	4.7	4.8	0.00
90-94	1.9	2.7	-0.05	2.0	2.1	0.00
95-99	0.4	0.6	-0.03	0.5	0.5	0.00
100-104	0.1	0.1	-0.01	0.1	0.1	-0.01
Gender: female	45.9	50.5	-0.09	46.8	46.8	0.00
Medical history: General						
Acute respiratory disease	8.4	9.5	-0.04	8.6	8.4	0.01
Attention deficit hyperactivity disorder	0.1	0.1	-0.01	0.1	0.1	-0.01
Chronic liver disease	1.5	1.5	0.00	1.5	1.6	0.00
Chronic obstructive lung disease	5.9	6.4	-0.02	6.0	5.8	0.01
Crohn's disease	0.2	0.1	0.01	0.2	0.1	0.01
Dementia	2.2	2.7	-0.03	2.3	2.2	0.01
Depressive disorder	13.4	14.0	-0.02	13.6	13.1	0.01
Diabetes mellitus	19.7	20.9	-0.03	19.9	19.7	0.01
Gastroesophageal reflux disease	9.5	12.1	-0.08	9.9	10.1	0.00
Gastrointestinal hemorrhage	0.7	0.8	-0.01	0.7	0.7	-0.01
Human immunodeficiency virus infection	0.5	0.5	0.00	0.5	0.6	-0.01
Hyperlipidemia	26.4	28.2	-0.04	26.7	26.7	0.00
Hypertensive disorder	99.2	99.3	-0.01	99.2	99.3	-0.01
Lesion of liver	0.8	0.8	0.00	0.8	0.8	0.01
Obesity	34.6	34.9	-0.01	34.6	34.8	0.00
Osteoarthritis	26.9	32.1	-0.12	27.9	27.7	0.00
Pneumonia	0.7	0.6	0.01	0.7	0.5	0.03
Psoriasis	3.7	3.8	0.00	3.7	3.5	0.01
Renal impairment	7.4	11.3	-0.13	8.0	8.2	-0.01

(Continued)	Before stratification			After stratification		
Characteristic	T (%)	C (%)	StdDiff	T (%)	C (%)	StdDiff
Rheumatoid arthritis	0.5	0.4	0.00	0.5	0.3	0.02
Schizophrenia	0.5	0.4	0.01	0.5	0.5	0.00
Ulcerative colitis	0.4	0.5	-0.01	0.4	0.5	-0.01
Urinary tract infectious disease	4.8	5.4	-0.03	4.9	4.8	0.00
Viral hepatitis C	1.2	1.1	0.00	1.2	1.2	0.00
Visual system disorder	36.1	38.5	-0.05	36.6	36.0	0.01
Medical history: Cardiovascular disease						
Atrial fibrillation	3.4	5.3	-0.09	3.8	3.8	0.00
Cerebrovascular disease	2.0	2.4	-0.02	2.1	1.9	0.02
Coronary arteriosclerosis	0.0	<0.1	-0.02	0.0	<0.1	-0.01
Heart disease	19.6	26.0	-0.15	20.7	20.5	0.00
Heart failure	1.5	2.3	-0.06	1.7	1.6	0.00
Ischemic heart disease	3.6	5.2	-0.08	3.9	3.8	0.00
Peripheral vascular disease	2.7	3.0	-0.02	2.8	2.6	0.01
Pulmonary embolism	0.5	0.6	-0.02	0.5	0.5	0.00
Venous thrombosis	1.0	1.2	-0.02	1.0	1.0	0.00
Medical history: Neoplasms						
Hematologic neoplasm	0.5	0.6	-0.01	0.6	0.5	0.01
Malignant lymphoma	0.4	0.4	0.00	0.4	0.3	0.01
Malignant neoplasm of anorectum	0.4	0.4	-0.01	0.4	0.4	-0.01
Malignant neoplastic disease	12.7	14.4	-0.05	13.1	12.7	0.01
Malignant tumor of breast	1.9	2.3	-0.02	2.0	1.9	0.01
Malignant tumor of colon	1.4	1.4	0.00	1.4	1.2	0.02
Malignant tumor of lung	0.1	0.1	-0.01	0.1	0.1	0.00
Malignant tumor of urinary bladder	1.0	1.1	0.00	1.1	1.0	0.01
Primary malignant neoplasm of prostate	2.0	2.4	-0.03	2.0	2.0	0.00
Medication use						
Antibacterials for systemic use	17.4	18.7	-0.03	17.6	17.6	0.00
Antidepressants	16.8	17.7	-0.02	17.0	16.8	0.01
Antiepileptics	7.0	7.3	-0.01	7.1	7.0	0.00
Antiinflammatory and antirheumatic products	22.3	19.9	0.06	21.9	21.6	0.01
Antineoplastic agents	0.5	0.6	-0.02	0.5	0.5	0.00
Antipsoriatics	0.6	0.9	-0.03	0.6	0.7	-0.01
Antithrombotic agents	17.2	22.4	-0.13	18.2	18.1	0.00
Drugs for acid related disorders	21.9	28.0	-0.14	23.0	23.0	0.00
Drugs for obstructive airway diseases	6.7	8.5	-0.07	6.9	7.0	0.00
Drugs used in diabetes	15.7	16.8	-0.03	15.9	15.9	0.00
Immunosuppressants	1.2	2.0	-0.06	1.3	1.5	-0.02
Lipid modifying agents	28.9	34.4	-0.12	29.9	29.7	0.00
Opioids	9.1	9.7	-0.02	9.3	9.2	0.00
Psycholeptics	25.9	28.9	-0.07	26.4	26.3	0.00
Psychostimulants, agents used for adhd and nootropics	1.2	1.4	-0.02	1.3	1.1	0.01

Supplementary Table 12: Baseline patient characteristcs for ACEI +combo (T) and ARB +combo (C) prevalent-users in the SIDIAP data source. We report proportion of initiators satisfying selected base-line characteristics and the standardized difference of population proportions (StdDiff) before and after stratification. Less extreme StdDiffs through stratification suggest improved balance between patient cohorts through propensity score adjustment.

	Before stratification			After stratification		
Characteristic	T (%)	C (%)	StdDiff	T (%)	C (%)	StdDiff
Age group						
15-19	0.0	0.0	0.00	0.0	0.0	0.00
20-24	0.1	0.1	0.00	0.1	0.1	-0.01
25-29	0.2	0.1	0.02	0.2	0.1	0.02
30-34	0.5	0.3	0.04	0.5	0.4	0.02
35-39	1.4	0.7	0.07	1.3	1.1	0.01
40-44	3.4	2.3	0.06	3.1	3.1	0.00
45-49	6.7	4.4	0.10	6.1	6.2	0.00
50-54	10.0	7.6	0.08	9.3	9.3	0.00
55-59	12.1	9.9	0.07	11.5	11.7	-0.01
60-64	13.5	12.6	0.03	13.3	13.3	0.00
65-69	13.1	13.9	-0.02	13.4	13.2	0.00
70-74	13.0	15.5	-0.07	13.5	14.0	-0.01
75-79	10.3	13.0	-0.09	11.0	10.8	0.00
80-84	7.3	8.9	-0.06	7.7	7.6	0.01
85-89	5.4	6.9	-0.06	5.8	5.8	0.00
90-94	2.4	3.0	-0.03	2.6	2.6	0.00
95-99	0.6	0.8	-0.03	0.6	0.7	-0.01
100-104	0.1	0.1	-0.01	0.1	0.1	-0.01
Gender: female	45.3	50.4	-0.10	46.7	46.5	0.00
Medical history: General						
Acute respiratory disease	8.5	9.7	-0.04	8.8	8.8	0.00
Attention deficit hyperactivity disorder	0.1	0.1	0.00	0.1	0.1	-0.01
Chronic liver disease	1.7	1.7	0.00	1.7	1.7	0.00
Chronic obstructive lung disease	6.8	7.3	-0.02	7.0	6.9	0.00
Crohn’s disease	0.2	0.2	0.00	0.2	0.2	0.00
Dementia	2.4	2.4	0.00	2.5	2.4	0.00
Depressive disorder	14.0	15.2	-0.03	14.4	14.3	0.00
Diabetes mellitus	22.4	24.7	-0.05	22.9	22.9	0.00
Gastroesophageal reflux disease	9.8	12.2	-0.08	10.4	10.5	0.00
Gastrointestinal hemorrhage	0.7	0.8	0.00	0.7	0.8	-0.01
Human immunodeficiency virus infection	0.5	0.4	0.01	0.5	0.5	0.00
Hyperlipidemia	27.7	28.6	-0.02	28.0	28.0	0.00
Hypertensive disorder	99.3	99.5	-0.03	99.3	99.5	-0.02
Lesion of liver	1.0	0.9	0.02	1.0	0.9	0.01
Obesity	38.0	40.1	-0.04	38.5	38.4	0.00
Osteoarthritis	28.8	33.5	-0.10	30.0	29.8	0.00
Pneumonia	0.8	0.8	0.00	0.8	0.7	0.01
Psoriasis	3.7	3.9	-0.01	3.7	3.7	0.00
Renal impairment	9.5	13.8	-0.13	10.6	10.7	0.00

(Continued)	Before stratification			After stratification		
Characteristic	T (%)	C (%)	StdDiff	T (%)	C (%)	StdDiff
Rheumatoid arthritis	0.4	0.5	-0.01	0.5	0.4	0.01
Schizophrenia	0.5	0.3	0.04	0.5	0.4	0.02
Ulcerative colitis	0.4	0.5	-0.02	0.4	0.5	-0.01
Urinary tract infectious disease	4.9	5.6	-0.04	5.0	5.1	0.00
Viral hepatitis C	1.3	1.3	0.00	1.3	1.3	0.00
Visual system disorder	37.8	40.0	-0.05	38.4	38.3	0.00
Medical history: Cardiovascular disease						
Atrial fibrillation	5.8	8.1	-0.09	6.4	6.4	0.00
Cerebrovascular disease	2.2	2.7	-0.03	2.4	2.3	0.00
Coronary arteriosclerosis	0.0	0.0	-0.01	0.0	0.0	0.00
Heart disease	25.6	32.3	-0.15	27.3	27.3	0.00
Heart failure	3.2	4.4	-0.06	3.5	3.5	0.00
Ischemic heart disease	6.2	7.9	-0.07	6.7	6.5	0.00
Peripheral vascular disease	3.5	4.0	-0.02	3.7	3.6	0.00
Pulmonary embolism	0.5	0.7	-0.02	0.6	0.6	0.00
Venous thrombosis	1.1	1.3	-0.02	1.2	1.1	0.01
Medical history: Neoplasms						
Hematologic neoplasm	0.6	0.6	0.00	0.6	0.5	0.01
Malignant lymphoma	0.4	0.4	0.00	0.5	0.4	0.01
Malignant neoplasm of anorectum	0.4	0.4	0.00	0.4	0.3	0.01
Malignant neoplastic disease	13.3	15.0	-0.05	13.8	13.7	0.00
Malignant tumor of breast	1.9	2.1	-0.02	2.0	1.9	0.01
Malignant tumor of colon	1.5	1.8	-0.02	1.5	1.5	0.00
Malignant tumor of lung	0.1	0.1	0.00	0.1	0.1	0.00
Malignant tumor of urinary bladder	1.1	1.3	-0.01	1.2	1.1	0.00
Primary malignant neoplasm of prostate	2.1	2.3	-0.01	2.2	2.2	0.00
Medication use						
Antibacterials for systemic use	17.9	19.5	-0.04	18.3	18.4	0.00
Antidepressants	17.4	18.9	-0.04	17.8	17.7	0.00
Antiepileptics	7.6	8.2	-0.03	7.7	7.9	-0.01
Antiinflammatory and antirheumatic products	20.9	18.8	0.05	20.3	20.3	0.00
Antineoplastic agents	0.5	0.6	-0.01	0.5	0.5	0.01
Antipsoriatics	0.7	1.0	-0.03	0.7	0.8	-0.01
Antithrombotic agents	22.6	28.8	-0.14	24.2	24.2	0.00
Drugs for acid related disorders	24.6	30.7	-0.14	26.1	26.2	0.00
Drugs for obstructive airway diseases	6.9	8.4	-0.06	7.2	7.3	0.00
Drugs used in diabetes	18.0	20.0	-0.05	18.4	18.3	0.00
Immunosuppressants	1.2	1.9	-0.06	1.3	1.4	-0.01
Lipid modifying agents	32.5	37.5	-0.11	33.7	33.7	0.00
Opioids	10.3	11.3	-0.03	10.6	10.7	0.00
Psycholeptics	27.4	30.8	-0.07	28.3	28.1	0.00
Psychostimulants, agents used for adhd and nootropics	1.8	2.1	-0.02	1.9	1.9	0.00

Supplementary Table 13: Baseline patient characteristcs for ACEI mono (T) and CCB/THZ mono (C) prevalent-users in the SIDIAP data source. We report proportion of initiators satisfying selected base-line characteristics and the standardized difference of population proportions (StdDiff) before and after stratification. Less extreme StdDiffs through stratification suggest improved balance between patient cohorts through propensity score adjustment.

	Before stratification			After stratification		
Characteristic	T (%)	C (%)	StdDiff	T (%)	C (%)	StdDiff
Age group						
15-19	0.0	0.0	-0.01	0.0	0.1	-0.02
20-24	0.1	0.1	0.00	0.1	0.1	-0.01
25-29	0.3	0.2	0.00	0.2	0.3	0.00
30-34	0.7	0.6	0.01	0.6	0.7	0.00
35-39	1.7	1.0	0.06	1.5	1.4	0.01
40-44	4.0	2.3	0.10	3.5	3.4	0.00
45-49	7.5	5.0	0.10	6.6	6.8	-0.01
50-54	10.9	7.9	0.10	9.9	10.1	-0.01
55-59	12.8	10.2	0.08	11.9	12.2	-0.01
60-64	13.7	12.5	0.04	13.4	13.1	0.01
65-69	13.1	13.4	-0.01	13.2	13.2	0.00
70-74	12.4	14.6	-0.06	13.2	12.9	0.01
75-79	9.3	12.7	-0.11	10.5	10.3	0.01
80-84	6.5	9.0	-0.09	7.3	7.3	0.00
85-89	4.4	6.5	-0.09	5.1	5.1	0.00
90-94	1.9	2.9	-0.06	2.2	2.3	0.00
95-99	0.4	0.8	-0.05	0.5	0.6	-0.02
100-104	0.1	0.1	0.01	0.1	0.1	0.01
Gender: female	45.9	53.0	-0.14	48.6	47.6	0.02
Medical history: General						
Acute respiratory disease	8.4	8.4	0.00	8.4	8.3	0.00
Attention deficit hyperactivity disorder	0.1	0.1	0.00	0.1	0.1	0.00
Chronic liver disease	1.5	1.7	-0.01	1.6	1.7	-0.01
Chronic obstructive lung disease	5.9	7.0	-0.04	6.2	6.3	0.00
Crohn's disease	0.2	0.2	0.00	0.2	0.2	0.01
Dementia	2.2	2.7	-0.03	2.5	2.3	0.01
Depressive disorder	13.4	14.8	-0.04	14.0	13.7	0.01
Diabetes mellitus	19.7	22.8	-0.08	20.6	20.4	0.01
Gastroesophageal reflux disease	9.5	11.5	-0.07	10.2	10.1	0.00
Gastrointestinal hemorrhage	0.7	0.8	-0.02	0.7	0.7	-0.01
Human immunodeficiency virus infection	0.5	0.4	0.02	0.5	0.4	0.01
Hyperlipidemia	26.4	27.9	-0.03	26.8	26.8	0.00
Hypertensive disorder	99.2	99.3	-0.01	99.2	99.4	-0.02
Lesion of liver	0.8	1.0	-0.02	0.8	0.9	-0.01
Obesity	34.6	37.5	-0.06	35.6	35.8	0.00
Osteoarthritis	26.9	33.2	-0.14	29.1	28.5	0.01
Pneumonia	0.7	0.6	0.02	0.7	0.6	0.02
Psoriasis	3.7	3.7	0.00	3.7	3.7	0.00
Renal impairment	7.4	13.8	-0.21	9.1	9.8	-0.03

(Continued)	Before stratification			After stratification		
Characteristic	T (%)	C (%)	StdDiff	T (%)	C (%)	StdDiff
Rheumatoid arthritis	0.5	0.5	0.00	0.5	0.4	0.02
Schizophrenia	0.5	0.5	0.00	0.5	0.6	-0.01
Ulcerative colitis	0.4	0.5	-0.01	0.4	0.4	0.00
Urinary tract infectious disease	4.8	5.3	-0.02	5.0	4.8	0.01
Viral hepatitis C	1.2	1.2	0.00	1.2	1.2	-0.01
Visual system disorder	36.1	42.0	-0.12	38.1	37.5	0.01
Medical history: Cardiovascular disease						
Atrial fibrillation	3.4	4.6	-0.06	3.9	3.7	0.01
Cerebrovascular disease	2.0	2.4	-0.03	2.2	2.0	0.01
Coronary arteriosclerosis	0.0	0.0	0.01	0.0	0.0	0.01
Heart disease	19.6	24.9	-0.13	21.2	21.2	0.00
Heart failure	1.5	2.1	-0.04	1.7	1.7	0.00
Ischemic heart disease	3.6	4.7	-0.06	3.9	3.9	0.00
Peripheral vascular disease	2.7	4.3	-0.09	3.1	3.3	-0.01
Pulmonary embolism	0.5	0.6	-0.01	0.6	0.5	0.01
Venous thrombosis	1.0	1.1	-0.01	1.0	0.9	0.01
Medical history: Neoplasms						
Hematologic neoplasm	0.5	0.7	-0.02	0.6	0.6	0.00
Malignant lymphoma	0.4	0.4	-0.01	0.4	0.4	0.00
Malignant neoplasm of anorectum	0.4	0.4	-0.01	0.4	0.4	0.00
Malignant neoplastic disease	12.7	15.3	-0.07	13.5	13.4	0.00
Malignant tumor of breast	1.9	2.0	-0.01	2.0	1.8	0.02
Malignant tumor of colon	1.4	1.5	-0.01	1.5	1.3	0.01
Malignant tumor of lung	0.1	0.2	-0.02	0.1	0.1	0.00
Malignant tumor of urinary bladder	1.0	1.4	-0.03	1.1	1.1	0.00
Primary malignant neoplasm of prostate	2.0	2.2	-0.02	2.0	2.1	0.00
Medication use						
Antibacterials for systemic use	17.4	18.8	-0.04	17.8	17.7	0.00
Antidepressants	16.8	18.1	-0.03	17.3	17.0	0.01
Antiepileptics	7.0	8.0	-0.04	7.3	7.2	0.00
Antiinflammatory and antirheumatic products	22.3	20.9	0.04	22.0	21.6	0.01
Antineoplastic agents	0.5	0.6	-0.02	0.5	0.5	0.01
Antipsoriatics	0.6	1.0	-0.04	0.7	0.8	-0.01
Antithrombotic agents	17.2	22.3	-0.13	18.9	18.8	0.00
Drugs for acid related disorders	21.9	28.1	-0.14	23.9	23.6	0.01
Drugs for obstructive airway diseases	6.7	7.9	-0.05	7.0	7.0	0.00
Drugs used in diabetes	15.7	18.2	-0.07	16.5	16.3	0.01
Immunosuppressants	1.2	2.4	-0.09	1.4	1.7	-0.03
Lipid modifying agents	28.9	32.9	-0.09	30.2	30.2	0.00
Opioids	9.1	11.4	-0.08	9.8	9.8	0.00
Psycholeptics	25.9	29.2	-0.07	27.1	26.6	0.01
Psychostimulants, agents used for adhd and nootropics	1.2	1.4	-0.02	1.3	1.2	0.01

Supplementary Table 14: Baseline patient characteristcs for ACEI +combo (T) and CCB/THZ +combo (C) prevalent-users in the SIDIAP data source. We report proportion of initiators satisfying selected base-line characteristics and the standardized difference of population proportions (StdDiff) before and after stratification. Less extreme StdDiffs through stratification suggest improved balance between patient cohorts through propensity score adjustment.

	Before stratification			After stratification		
Characteristic	T (%)	C (%)	StdDiff	T (%)	C (%)	StdDiff
Age group						
15-19	0.0	0.0	-0.01	0.0	0.0	-0.01
20-24	0.1	0.1	0.00	0.1	0.1	0.00
25-29	0.2	0.1	0.02	0.2	0.2	0.01
30-34	0.6	0.4	0.03	0.5	0.5	0.01
35-39	1.5	0.9	0.06	1.2	1.2	0.01
40-44	3.6	2.2	0.08	3.0	2.9	0.00
45-49	6.8	4.7	0.09	5.8	6.0	-0.01
50-54	10.1	7.8	0.08	8.9	9.2	-0.01
55-59	12.1	10.0	0.07	11.0	11.3	-0.01
60-64	13.1	12.4	0.02	12.8	12.8	0.00
65-69	13.0	13.4	-0.01	13.3	13.1	0.01
70-74	12.5	15.0	-0.07	13.5	13.7	0.00
75-79	10.0	13.3	-0.10	11.5	11.3	0.01
80-84	7.5	9.1	-0.06	8.3	8.1	0.01
85-89	5.6	6.9	-0.06	6.2	6.1	0.00
90-94	2.6	3.1	-0.02	2.9	2.8	0.00
95-99	0.6	0.8	-0.03	0.7	0.8	-0.01
100-104	0.1	0.1	0.01	0.1	0.1	0.01
Gender: female	46.2	51.5	-0.11	48.9	48.2	0.01
Medical history: General						
Acute respiratory disease	8.8	8.9	0.00	8.9	8.8	0.00
Attention deficit hyperactivity disorder	0.1	0.1	0.00	0.1	0.1	0.00
Chronic liver disease	1.7	1.9	-0.01	1.8	1.8	0.00
Chronic obstructive lung disease	6.9	7.3	-0.02	7.1	7.0	0.00
Crohn’s disease	0.2	0.2	0.00	0.2	0.2	0.01
Dementia	2.7	2.5	0.01	2.7	2.6	0.01
Depressive disorder	14.2	15.3	-0.03	14.8	14.6	0.01
Diabetes mellitus	21.2	25.2	-0.09	22.9	22.9	0.00
Gastroesophageal reflux disease	9.8	11.8	-0.06	10.8	10.7	0.00
Gastrointestinal hemorrhage	0.7	0.8	-0.01	0.7	0.8	-0.01
Human immunodeficiency virus infection	0.5	0.3	0.02	0.5	0.4	0.01
Hyperlipidemia	27.1	28.6	-0.03	27.8	27.7	0.00
Hypertensive disorder	99.2	99.5	-0.04	99.3	99.4	-0.01
Lesion of liver	1.0	1.2	-0.01	1.1	1.1	0.00
Obesity	35.5	40.1	-0.10	37.7	37.5	0.00
Osteoarthritis	28.6	33.7	-0.11	31.2	30.6	0.01
Pneumonia	0.8	0.8	0.01	0.9	0.8	0.01
Psoriasis	3.6	3.8	-0.01	3.7	3.7	0.00
Renal impairment	9.3	15.3	-0.18	11.7	12.2	-0.01

(Continued)	Before stratification			After stratification		
Characteristic	T (%)	C (%)	StdDiff	T (%)	C (%)	StdDiff
Rheumatoid arthritis	0.5	0.6	-0.01	0.5	0.5	0.01
Schizophrenia	0.6	0.4	0.02	0.5	0.4	0.01
Ulcerative colitis	0.4	0.5	-0.01	0.4	0.5	-0.01
Urinary tract infectious disease	5.2	5.3	-0.01	5.3	5.2	0.01
Viral hepatitis C	1.2	1.3	-0.01	1.2	1.3	0.00
Visual system disorder	37.7	41.6	-0.08	39.7	39.2	0.01
Medical history: Cardiovascular disease						
Atrial fibrillation	6.3	7.1	-0.03	6.8	6.7	0.00
Cerebrovascular disease	2.3	2.7	-0.03	2.5	2.4	0.01
Coronary arteriosclerosis	0.0	0.0	0.00	0.0	0.0	0.00
Heart disease	26.0	30.2	-0.09	27.9	27.8	0.00
Heart failure	3.9	3.6	0.01	3.8	3.7	0.00
Ischemic heart disease	6.7	7.2	-0.02	6.9	6.8	0.00
Peripheral vascular disease	3.3	4.5	-0.06	3.8	3.9	-0.01
Pulmonary embolism	0.6	0.6	0.00	0.7	0.6	0.01
Venous thrombosis	1.1	1.3	-0.01	1.2	1.1	0.01
Medical history: Neoplasms						
Hematologic neoplasm	0.6	0.7	-0.01	0.7	0.6	0.01
Malignant lymphoma	0.4	0.5	0.00	0.5	0.4	0.01
Malignant neoplasm of anorectum	0.4	0.4	0.00	0.4	0.3	0.00
Malignant neoplastic disease	13.7	15.4	-0.05	14.5	14.4	0.00
Malignant tumor of breast	2.0	2.1	0.00	2.1	2.0	0.01
Malignant tumor of colon	1.5	1.7	-0.02	1.6	1.6	0.00
Malignant tumor of lung	0.1	0.1	0.00	0.1	0.1	0.00
Malignant tumor of urinary bladder	1.2	1.3	-0.01	1.2	1.2	0.00
Primary malignant neoplasm of prostate	2.2	2.3	-0.01	2.2	2.2	0.00
Medication use						
Antibacterials for systemic use	18.5	19.4	-0.02	18.9	18.9	0.00
Antidepressants	17.7	19.0	-0.03	18.4	18.1	0.01
Antiepileptics	7.5	8.5	-0.04	7.9	8.0	0.00
Antiinflammatory and antirheumatic products	21.4	19.7	0.04	20.7	20.6	0.00
Antineoplastic agents	0.5	0.6	-0.01	0.5	0.5	0.01
Antipsoriatics	0.7	1.1	-0.04	0.7	0.9	-0.02
Antithrombotic agents	22.9	27.0	-0.09	24.7	24.6	0.00
Drugs for acid related disorders	24.6	30.2	-0.12	27.2	26.9	0.01
Drugs for obstructive airway diseases	7.1	8.0	-0.03	7.5	7.5	0.00
Drugs used in diabetes	17.0	20.3	-0.09	18.3	18.4	0.00
Immunosuppressants	1.2	2.2	-0.08	1.5	1.8	-0.03
Lipid modifying agents	31.5	35.9	-0.09	33.5	33.4	0.00
Opioids	10.0	12.0	-0.06	11.0	10.8	0.00
Psycholeptics	27.7	30.8	-0.07	29.3	28.8	0.01
Psychostimulants, agents used for adhd and nootropics	2.0	2.0	0.00	2.1	2.0	0.01

Supplementary Table 15: Baseline patient characteristcs for ACEI mono (T) and CCB mono (C) prevalent-users in the SIDIAP data source. We report proportion of initiators satisfying selected base-line characteristics and the standardized difference of population proportions (StdDiff) before and after stratification. Less extreme StdDiffs through stratification suggest improved balance between patient cohorts through propensity score adjustment.

Characteristic	Before stratification			After stratification		
	T (%)	C (%)	StdDiff	T (%)	C (%)	StdDiff
Age group						
15-19	0.0	<0.1	-0.01	0.0	0.0	-0.01
20-24	0.1	0.2	-0.02	0.1	0.2	-0.03
25-29	0.3	0.3	-0.01	0.2	0.3	-0.02
30-34	0.7	0.6	0.00	0.6	0.8	-0.02
35-39	1.7	1.1	0.05	1.6	1.5	0.01
40-44	4.0	2.5	0.09	3.7	3.6	0.00
45-49	7.5	5.0	0.10	6.9	7.0	0.00
50-54	10.9	8.0	0.10	10.2	10.4	0.00
55-59	12.8	10.6	0.07	12.3	12.2	0.00
60-64	13.7	12.4	0.04	13.5	13.1	0.01
65-69	13.1	13.1	0.00	13.1	13.0	0.00
70-74	12.4	14.1	-0.05	12.8	12.8	0.00
75-79	9.3	12.3	-0.10	10.1	9.9	0.00
80-84	6.5	9.1	-0.10	7.1	7.2	0.00
85-89	4.4	6.9	-0.11	5.0	5.0	0.00
90-94	1.9	2.9	-0.06	2.1	2.2	0.00
95-99	0.4	0.9	-0.06	0.5	0.6	-0.02
100-104	0.1	<0.1	0.01	0.1	0.0	0.02
Gender: female	45.9	44.4	0.03	45.7	45.4	0.01
Medical history: General						
Acute respiratory disease	8.4	8.5	0.00	8.4	8.4	0.00
Attention deficit hyperactivity disorder	0.1	0.1	0.00	0.1	0.1	-0.01
Chronic liver disease	1.5	2.1	-0.04	1.6	1.8	-0.01
Chronic obstructive lung disease	5.9	7.8	-0.08	6.3	6.4	0.00
Crohn's disease	0.2	0.1	0.01	0.2	0.1	0.01
Dementia	2.2	2.8	-0.04	2.4	2.4	0.00
Depressive disorder	13.4	14.6	-0.04	13.8	13.4	0.01
Diabetes mellitus	19.7	26.5	-0.16	21.2	20.7	0.01
Gastroesophageal reflux disease	9.5	11.4	-0.06	10.0	9.8	0.00
Gastrointestinal hemorrhage	0.7	1.0	-0.04	0.7	0.8	-0.02
Human immunodeficiency virus infection	0.5	0.4	0.02	0.5	0.3	0.02
Hyperlipidemia	26.4	27.9	-0.04	26.8	26.4	0.01
Hypertensive disorder	99.2	99.4	-0.03	99.2	99.4	-0.02
Lesion of liver	0.8	1.3	-0.05	0.9	1.0	-0.01
Obesity	34.6	37.7	-0.06	35.4	35.3	0.00
Osteoarthritis	26.9	31.2	-0.10	28.1	27.6	0.01
Pneumonia	0.7	0.6	0.01	0.7	0.6	0.02
Psoriasis	3.7	3.8	-0.01	3.7	3.6	0.00
Renal impairment	7.4	17.6	-0.31	9.2	10.2	-0.04

(Continued)	Before stratification			After stratification		
Characteristic	T (%)	C (%)	StdDiff	T (%)	C (%)	StdDiff
Rheumatoid arthritis	0.5	0.4	0.01	0.5	0.3	0.02
Schizophrenia	0.5	0.6	-0.01	0.5	0.6	-0.01
Ulcerative colitis	0.4	0.5	-0.01	0.4	0.4	0.00
Urinary tract infectious disease	4.8	5.0	-0.01	4.9	4.7	0.01
Viral hepatitis C	1.2	1.5	-0.03	1.2	1.3	-0.01
Visual system disorder	36.1	41.8	-0.12	37.5	36.8	0.01
Medical history: Cardiovascular disease						
Atrial fibrillation	3.4	5.4	-0.10	3.9	3.9	0.00
Cerebrovascular disease	2.0	2.9	-0.05	2.3	2.1	0.01
Coronary arteriosclerosis	0.0	<0.1	0.00	0.0	0.0	0.01
Heart disease	19.6	27.8	-0.20	21.4	21.2	0.01
Heart failure	1.5	2.8	-0.09	1.8	1.8	0.00
Ischemic heart disease	3.6	6.2	-0.12	4.1	4.2	0.00
Peripheral vascular disease	2.7	5.4	-0.14	3.2	3.5	-0.02
Pulmonary embolism	0.5	0.6	-0.01	0.5	0.5	0.00
Venous thrombosis	1.0	1.1	-0.01	1.0	0.9	0.01
Medical history: Neoplasms						
Hematologic neoplasm	0.5	0.8	-0.03	0.6	0.6	0.00
Malignant lymphoma	0.4	0.4	-0.01	0.4	0.4	0.01
Malignant neoplasm of anorectum	0.4	0.5	-0.02	0.4	0.4	0.00
Malignant neoplastic disease	12.7	15.8	-0.09	13.4	13.3	0.00
Malignant tumor of breast	1.9	1.6	0.03	1.9	1.6	0.02
Malignant tumor of colon	1.4	1.5	-0.01	1.5	1.3	0.02
Malignant tumor of lung	0.1	0.2	-0.02	0.1	0.1	-0.01
Malignant tumor of urinary bladder	1.0	1.8	-0.06	1.2	1.2	-0.01
Primary malignant neoplasm of prostate	2.0	2.5	-0.04	2.1	2.1	0.00
Medication use						
Antibacterials for systemic use	17.4	18.9	-0.04	17.7	17.8	0.00
Antidepressants	16.8	17.9	-0.03	17.1	16.8	0.01
Antiepileptics	7.0	8.1	-0.04	7.2	7.1	0.00
Antiinflammatory and antirheumatic products	22.3	18.8	0.09	21.6	21.6	0.00
Antineoplastic agents	0.5	0.5	0.00	0.5	0.4	0.02
Antipsoriatics	0.6	1.2	-0.06	0.7	0.9	-0.02
Antithrombotic agents	17.2	25.7	-0.21	19.2	19.1	0.00
Drugs for acid related disorders	21.9	29.6	-0.18	23.6	23.2	0.01
Drugs for obstructive airway diseases	6.7	7.6	-0.04	6.9	6.7	0.01
Drugs used in diabetes	15.7	21.3	-0.14	17.0	16.4	0.01
Immunosuppressants	1.2	2.8	-0.12	1.3	1.8	-0.04
Lipid modifying agents	28.9	35.3	-0.14	30.4	30.2	0.01
Opioids	9.1	11.4	-0.07	9.6	9.6	0.00
Psycholeptics	25.9	29.1	-0.07	26.7	26.2	0.01
Psychostimulants, agents used for adhd and nootropics	1.2	1.8	-0.05	1.4	1.4	0.00

Supplementary Table 16: Baseline patient characteristcs for ACEI +combo (T) and CCB +combo (C) prevalent-users in the SIDIAP data source. We report proportion of initiators satisfying selected base-line characteristics and the standardized difference of population proportions (StdDiff) before and after stratification. Less extreme StdDiffs through stratification suggest improved balance between patient cohorts through propensity score adjustment.

	Before stratification			After stratification		
Characteristic	T (%)	C (%)	StdDiff	T (%)	C (%)	StdDiff
Age group						
15-19	0.0	0.0	-0.01	0.0	0.0	-0.01
20-24	0.1	0.1	-0.02	0.1	0.2	-0.03
25-29	0.2	0.2	0.00	0.2	0.2	0.00
30-34	0.5	0.5	0.00	0.5	0.6	-0.01
35-39	1.4	1.0	0.04	1.3	1.3	0.00
40-44	3.4	2.5	0.06	3.2	3.2	0.00
45-49	6.8	4.8	0.09	6.2	6.5	-0.01
50-54	10.0	7.7	0.08	9.4	9.6	0.00
55-59	12.2	10.0	0.07	11.6	11.7	0.00
60-64	13.6	12.1	0.04	13.2	13.2	0.00
65-69	13.2	13.0	0.01	13.2	13.0	0.01
70-74	12.9	14.3	-0.04	13.3	13.0	0.01
75-79	10.1	12.9	-0.09	10.9	10.7	0.01
80-84	7.3	9.2	-0.07	7.8	7.8	0.00
85-89	5.3	7.4	-0.09	5.9	5.8	0.00
90-94	2.3	3.3	-0.06	2.6	2.6	0.00
95-99	0.5	0.9	-0.04	0.6	0.7	-0.01
100-104	0.1	0.1	0.00	0.1	0.1	0.01
Gender: female	46.2	43.9	0.05	45.8	45.5	0.01
Medical history: General						
Acute respiratory disease	8.6	8.8	0.00	8.7	8.8	0.00
Attention deficit hyperactivity disorder	0.1	0.1	0.00	0.1	0.1	-0.01
Chronic liver disease	1.7	2.1	-0.03	1.8	1.9	-0.01
Chronic obstructive lung disease	6.7	8.3	-0.06	7.1	7.1	0.00
Crohn's disease	0.2	0.2	0.00	0.2	0.2	0.01
Dementia	2.4	2.8	-0.02	2.5	2.4	0.01
Depressive disorder	14.0	15.2	-0.03	14.4	14.1	0.01
Diabetes mellitus	21.7	28.7	-0.16	23.2	23.1	0.00
Gastroesophageal reflux disease	9.9	11.5	-0.05	10.3	10.2	0.00
Gastrointestinal hemorrhage	0.7	0.9	-0.03	0.8	0.8	-0.01
Human immunodeficiency virus infection	0.5	0.3	0.02	0.4	0.3	0.02
Hyperlipidemia	27.6	28.4	-0.02	27.8	27.8	0.00
Hypertensive disorder	99.3	99.5	-0.03	99.3	99.5	-0.02
Lesion of liver	1.0	1.4	-0.04	1.1	1.1	0.00
Obesity	37.5	39.6	-0.04	38.1	38.0	0.00
Osteoarthritis	28.7	32.0	-0.07	29.7	29.4	0.01
Pneumonia	0.8	0.9	-0.01	0.8	0.8	0.01
Psoriasis	3.6	3.8	-0.01	3.7	3.7	0.00
Renal impairment	9.1	19.6	-0.30	11.0	12.2	-0.04

(Continued)	Before stratification			After stratification		
Characteristic	T (%)	C (%)	StdDiff	T (%)	C (%)	StdDiff
Rheumatoid arthritis	0.5	0.5	-0.01	0.5	0.4	0.01
Schizophrenia	0.5	0.5	0.00	0.5	0.5	0.00
Ulcerative colitis	0.4	0.5	-0.01	0.4	0.4	0.00
Urinary tract infectious disease	4.9	5.1	-0.01	5.0	4.9	0.01
Viral hepatitis C	1.3	1.4	-0.01	1.3	1.3	0.00
Visual system disorder	37.7	41.9	-0.09	38.8	38.5	0.00
Medical history: Cardiovascular disease						
Atrial fibrillation	5.7	7.9	-0.09	6.3	6.2	0.00
Cerebrovascular disease	2.1	3.1	-0.06	2.4	2.3	0.00
Coronary arteriosclerosis	0.0	0.1	-0.01	0.0	0.0	0.00
Heart disease	25.1	33.1	-0.18	27.0	26.9	0.00
Heart failure	3.2	4.7	-0.08	3.5	3.6	0.00
Ischemic heart disease	5.9	8.9	-0.12	6.6	6.7	0.00
Peripheral vascular disease	3.3	6.0	-0.13	3.8	4.1	-0.01
Pulmonary embolism	0.5	0.7	-0.02	0.6	0.6	0.00
Venous thrombosis	1.1	1.3	-0.02	1.2	1.1	0.01
Medical history: Neoplasms						
Hematologic neoplasm	0.5	0.8	-0.03	0.6	0.6	0.00
Malignant lymphoma	0.4	0.5	-0.02	0.4	0.4	0.00
Malignant neoplasm of anorectum	0.4	0.4	0.00	0.4	0.3	0.01
Malignant neoplastic disease	13.2	16.3	-0.09	13.9	13.8	0.00
Malignant tumor of breast	2.0	1.7	0.02	1.9	1.8	0.01
Malignant tumor of colon	1.4	1.8	-0.03	1.5	1.4	0.01
Malignant tumor of lung	0.1	0.1	-0.01	0.1	0.1	0.00
Malignant tumor of urinary bladder	1.1	1.7	-0.06	1.2	1.3	-0.01
Primary malignant neoplasm of prostate	2.1	2.7	-0.04	2.2	2.2	0.00
Medication use						
Antibacterials for systemic use	18.0	20.1	-0.05	18.4	18.9	-0.01
Antidepressants	17.5	18.5	-0.03	17.8	17.5	0.01
Antiepileptics	7.4	8.6	-0.04	7.7	7.7	0.00
Antiinflammatory and antirheumatic products	21.4	18.0	0.08	20.6	20.9	-0.01
Antineoplastic agents	0.5	0.5	0.00	0.5	0.4	0.02
Antipsoriatics	0.7	1.4	-0.07	0.7	1.0	-0.03
Antithrombotic agents	21.9	30.8	-0.20	24.1	23.9	0.00
Drugs for acid related disorders	24.3	31.9	-0.17	26.1	25.8	0.01
Drugs for obstructive airway diseases	6.9	8.1	-0.05	7.2	7.3	0.00
Drugs used in diabetes	17.3	23.3	-0.15	18.6	18.6	0.00
Immunosuppressants	1.1	2.8	-0.12	1.3	1.9	-0.05
Lipid modifying agents	32.0	38.0	-0.13	33.4	33.4	0.00
Opioids	10.3	12.1	-0.06	10.7	10.7	0.00
Psycholeptics	27.4	30.5	-0.07	28.2	27.8	0.01
Psychostimulants, agents used for adhd and nootropics	1.7	2.6	-0.06	2.0	1.9	0.00

Supplementary Table 17: Baseline patient characteristcs for ACEI mono (T) and THZ mono (C) prevalent-users in the SIDIAP data source. We report proportion of initiators satisfying selected base-line characteristics and the standardized difference of population proportions (StdDiff) before and after stratification. Less extreme StdDiffs through stratification suggest improved balance between patient cohorts through propensity score adjustment.

	Before stratification			After stratification		
Characteristic	T (%)	C (%)	StdDiff	T (%)	C (%)	StdDiff
Age group						
15-19	0.0	<0.1	-0.02	0.0	0.1	-0.03
20-24	0.1	<0.1	0.03	0.1	-0.1	0.03
25-29	0.3	0.1	0.03	0.2	0.2	0.02
30-34	0.7	0.5	0.02	0.7	0.6	0.01
35-39	1.7	1.0	0.06	1.6	1.2	0.04
40-44	4.0	2.0	0.12	3.8	3.5	0.02
45-49	7.5	5.0	0.10	7.2	7.5	-0.01
50-54	10.9	7.8	0.11	10.5	10.3	0.00
55-59	12.8	9.8	0.10	12.4	12.0	0.01
60-64	13.7	12.7	0.03	13.7	12.9	0.02
65-69	13.1	13.7	-0.02	13.2	13.4	0.00
70-74	12.4	15.5	-0.09	12.8	12.9	0.00
75-79	9.3	13.5	-0.13	9.9	10.4	-0.02
80-84	6.5	8.7	-0.08	6.8	7.2	-0.02
85-89	4.4	6.0	-0.07	4.6	4.9	-0.01
90-94	1.9	2.8	-0.06	2.0	2.3	-0.02
95-99	0.4	0.7	-0.04	0.4	0.6	-0.03
100-104	0.1	<0.1	0.00	0.1	-0.1	0.00
Gender: female	45.9	67.6	-0.45	49.1	48.0	0.02
Medical history: General						
Acute respiratory disease	8.4	8.2	0.01	8.4	8.1	0.01
Attention deficit hyperactivity disorder	0.1	<0.1	0.00	0.1	-0.1	0.00
Chronic liver disease	1.5	1.1	0.04	1.5	1.3	0.02
Chronic obstructive lung disease	5.9	5.5	0.02	5.8	6.5	-0.03
Crohn's disease	0.2	0.2	-0.01	0.2	0.2	-0.01
Dementia	2.2	2.7	-0.03	2.3	2.5	-0.01
Depressive disorder	13.4	14.9	-0.04	13.7	14.0	-0.01
Diabetes mellitus	19.7	16.4	0.09	19.3	19.2	0.00
Gastroesophageal reflux disease	9.5	11.5	-0.07	9.7	10.1	-0.01
Gastrointestinal hemorrhage	0.7	0.6	0.01	0.6	0.6	0.00
Human immunodeficiency virus infection	0.5	0.3	0.02	0.5	0.5	0.00
Hyperlipidemia	26.4	27.6	-0.03	26.5	26.6	0.00
Hypertensive disorder	99.2	99.2	0.00	99.1	99.5	-0.04
Lesion of liver	0.8	0.7	0.02	0.8	0.6	0.02
Obesity	34.6	37.1	-0.05	34.8	35.7	-0.02
Osteoarthritis	26.9	36.4	-0.21	28.2	28.0	0.00
Pneumonia	0.7	0.5	0.02	0.7	0.5	0.02
Psoriasis	3.7	3.4	0.01	3.7	3.7	0.00
Renal impairment	7.4	7.3	0.00	7.4	7.7	-0.01

(Continued)	Before stratification			After stratification		
Characteristic	T (%)	C (%)	StdDiff	T (%)	C (%)	StdDiff
Rheumatoid arthritis	0.5	0.6	-0.02	0.5	0.4	0.01
Schizophrenia	0.5	0.4	0.02	0.5	0.5	0.01
Ulcerative colitis	0.4	0.4	0.00	0.4	0.4	0.01
Urinary tract infectious disease	4.8	5.6	-0.04	5.0	4.8	0.01
Viral hepatitis C	1.2	0.8	0.04	1.1	1.0	0.02
Visual system disorder	36.1	42.4	-0.13	36.9	37.6	-0.01
Medical history: Cardiovascular disease						
Atrial fibrillation	3.4	3.3	0.01	3.5	3.3	0.01
Cerebrovascular disease	2.0	1.6	0.03	2.0	1.8	0.02
Coronary arteriosclerosis	0.0	0.0		0.0	0.0	
Heart disease	19.6	19.8	-0.01	19.6	19.9	-0.01
Heart failure	1.5	0.9	0.05	1.5	1.3	0.01
Ischemic heart disease	3.6	2.1	0.09	3.5	3.7	-0.01
Peripheral vascular disease	2.7	2.1	0.04	2.6	2.7	-0.01
Pulmonary embolism	0.5	0.5	0.00	0.5	0.4	0.01
Venous thrombosis	1.0	1.1	-0.02	1.0	0.9	0.01
Medical history: Neoplasms						
Hematologic neoplasm	0.5	0.5	0.01	0.5	0.4	0.03
Malignant lymphoma	0.4	0.4	0.00	0.4	0.3	0.01
Malignant neoplasm of anorectum	0.4	0.3	0.01	0.4	0.3	0.01
Malignant neoplastic disease	12.7	14.0	-0.04	12.9	12.6	0.01
Malignant tumor of breast	1.9	2.8	-0.06	2.1	1.9	0.02
Malignant tumor of colon	1.4	1.5	-0.01	1.4	1.3	0.01
Malignant tumor of lung	0.1	0.1	-0.01	0.1	0.1	0.00
Malignant tumor of urinary bladder	1.0	0.7	0.04	1.0	0.9	0.02
Primary malignant neoplasm of prostate	2.0	1.6	0.03	1.9	2.1	-0.02
Medication use						
Antibacterials for systemic use	17.4	18.7	-0.03	17.6	17.4	0.01
Antidepressants	16.8	18.7	-0.05	17.1	16.9	0.01
Antiepileptics	7.0	7.8	-0.03	7.1	7.3	-0.01
Antiinflammatory and antirheumatic products	22.3	24.7	-0.06	22.7	21.9	0.02
Antineoplastic agents	0.5	0.8	-0.04	0.5	0.7	-0.02
Antipsoriatics	0.6	0.6	0.00	0.6	0.7	-0.01
Antithrombotic agents	17.2	16.3	0.03	17.1	17.2	0.00
Drugs for acid related disorders	21.9	25.4	-0.08	22.4	22.2	0.00
Drugs for obstructive airway diseases	6.7	8.1	-0.06	6.8	7.4	-0.02
Drugs used in diabetes	15.7	12.7	0.09	15.3	15.1	0.01
Immunosuppressants	1.2	1.6	-0.04	1.2	1.4	-0.02
Lipid modifying agents	28.9	28.4	0.01	28.8	29.2	-0.01
Opioids	9.1	11.2	-0.07	9.5	9.2	0.01
Psycholeptics	25.9	29.6	-0.08	26.5	26.3	0.01
Psychostimulants, agents used for adhd and nootropics	1.2	0.6	0.06	1.2	1.0	0.01

Supplementary Table 18: Baseline patient characteristcs for ACEI +combo (T) and THZ +combo (C) prevalent-users in the SIDIAP data source. We report proportion of initiators satisfying selected base-line characteristics and the standardized difference of population proportions (StdDiff) before and after stratification. Less extreme StdDiffs through stratification suggest improved balance between patient cohorts through propensity score adjustment.

	Before stratification			After stratification		
Characteristic	T (%)	C (%)	StdDiff	T (%)	C (%)	StdDiff
Age group						
15-19	0.0	0.0	0.00	0.0	0.0	0.00
20-24	0.1	0.0	0.02	0.1	0.0	0.02
25-29	0.2	0.1	0.03	0.2	0.1	0.03
30-34	0.6	0.3	0.04	0.5	0.4	0.02
35-39	1.5	0.7	0.08	1.3	1.1	0.02
40-44	3.5	2.0	0.09	3.1	3.1	0.00
45-49	6.7	4.6	0.09	6.1	6.2	-0.01
50-54	9.9	7.8	0.07	9.3	9.5	-0.01
55-59	11.9	10.0	0.06	11.4	11.4	0.00
60-64	13.1	12.8	0.01	13.1	12.9	0.00
65-69	12.9	13.8	-0.03	13.3	13.1	0.01
70-74	12.6	15.8	-0.09	13.3	14.0	-0.02
75-79	10.1	13.6	-0.11	11.1	11.1	0.00
80-84	7.5	8.8	-0.05	7.9	7.8	0.00
85-89	5.7	6.3	-0.02	5.9	5.8	0.00
90-94	2.8	2.7	0.00	2.8	2.6	0.01
95-99	0.6	0.7	-0.01	0.6	0.8	-0.02
100-104	0.1	0.1	0.01	0.1	0.1	0.00
Gender: female	45.4	58.3	-0.26	49.4	48.2	0.03
Medical history: General						
Acute respiratory disease	8.8	8.9	0.00	8.8	8.6	0.01
Attention deficit hyperactivity disorder	0.1	0.1	0.00	0.1	0.1	0.00
Chronic liver disease	1.8	1.7	0.01	1.7	1.8	0.00
Chronic obstructive lung disease	7.0	6.3	0.03	6.8	6.7	0.01
Crohn's disease	0.2	0.2	0.01	0.2	0.2	0.01
Dementia	2.8	2.1	0.04	2.6	2.5	0.00
Depressive disorder	14.2	15.4	-0.03	14.6	14.5	0.00
Diabetes mellitus	21.8	22.5	-0.02	22.0	22.0	0.00
Gastroesophageal reflux disease	9.8	12.1	-0.07	10.4	10.5	0.00
Gastrointestinal hemorrhage	0.7	0.7	0.00	0.7	0.8	-0.01
Human immunodeficiency virus infection	0.5	0.3	0.03	0.5	0.4	0.00
Hyperlipidemia	27.1	28.8	-0.04	27.6	27.6	0.00
Hypertensive disorder	99.2	99.5	-0.03	99.2	99.5	-0.04
Lesion of liver	1.1	0.8	0.02	1.0	0.9	0.01
Obesity	35.8	41.5	-0.12	37.4	37.4	0.00
Osteoarthritis	28.8	35.1	-0.14	30.7	30.3	0.01
Pneumonia	0.8	0.7	0.02	0.8	0.7	0.02
Psoriasis	3.6	3.7	0.00	3.7	3.7	0.00
Renal impairment	9.9	10.5	-0.02	10.2	10.3	0.00

(Continued)	Before stratification			After stratification		
Characteristic	T (%)	C (%)	StdDiff	T (%)	C (%)	StdDiff
Rheumatoid arthritis	0.5	0.6	-0.02	0.5	0.5	0.00
Schizophrenia	0.6	0.3	0.04	0.5	0.4	0.02
Ulcerative colitis	0.4	0.5	-0.02	0.4	0.5	-0.01
Urinary tract infectious disease	5.2	5.5	-0.01	5.3	5.1	0.01
Viral hepatitis C	1.3	1.2	0.00	1.2	1.3	0.00
Visual system disorder	37.8	41.1	-0.07	38.9	38.5	0.01
Medical history: Cardiovascular disease						
Atrial fibrillation	6.5	6.3	0.01	6.5	6.4	0.00
Cerebrovascular disease	2.3	2.2	0.01	2.3	2.2	0.01
Coronary arteriosclerosis	0.0	0.0	0.01	0.0	0.0	0.01
Heart disease	26.7	27.5	-0.02	26.9	27.1	0.00
Heart failure	4.0	2.4	0.09	3.6	3.4	0.01
Ischemic heart disease	7.0	5.4	0.07	6.6	6.5	0.01
Peripheral vascular disease	3.5	3.2	0.02	3.4	3.4	0.00
Pulmonary embolism	0.6	0.5	0.01	0.6	0.5	0.02
Venous thrombosis	1.1	1.2	-0.01	1.2	1.1	0.01
Medical history: Neoplasms						
Hematologic neoplasm	0.7	0.5	0.02	0.7	0.5	0.03
Malignant lymphoma	0.4	0.4	0.01	0.5	0.4	0.01
Malignant neoplasm of anorectum	0.4	0.4	0.00	0.4	0.3	0.01
Malignant neoplastic disease	13.9	14.3	-0.01	14.1	13.9	0.00
Malignant tumor of breast	2.0	2.4	-0.03	2.2	2.0	0.01
Malignant tumor of colon	1.5	1.6	-0.01	1.5	1.6	0.00
Malignant tumor of lung	0.1	0.1	0.01	0.1	0.1	0.01
Malignant tumor of urinary bladder	1.2	0.9	0.03	1.1	1.0	0.01
Primary malignant neoplasm of prostate	2.2	1.8	0.03	2.1	2.1	0.00
Medication use						
Antibacterials for systemic use	18.5	18.6	0.00	18.6	18.3	0.01
Antidepressants	17.7	19.1	-0.04	18.2	18.0	0.00
Antiepileptics	7.6	8.3	-0.02	7.8	8.0	-0.01
Antiinflammatory and antirheumatic products	21.1	21.3	-0.01	21.2	20.9	0.00
Antineoplastic agents	0.5	0.6	-0.01	0.5	0.5	0.00
Antipsoriatics	0.7	0.7	0.00	0.7	0.7	0.00
Antithrombotic agents	23.8	23.7	0.00	23.8	23.7	0.00
Drugs for acid related disorders	25.2	28.4	-0.07	26.1	25.7	0.01
Drugs for obstructive airway diseases	7.1	7.9	-0.03	7.3	7.3	0.00
Drugs used in diabetes	17.4	17.9	-0.01	17.6	17.6	0.00
Immunosuppressants	1.3	1.5	-0.02	1.3	1.4	-0.01
Lipid modifying agents	32.1	34.4	-0.05	32.7	32.8	0.00
Opioids	10.1	11.8	-0.05	10.7	10.6	0.00
Psycholeptics	27.9	30.8	-0.06	28.8	28.6	0.01
Psychostimulants, agents used for adhd and nootropics	2.1	1.6	0.04	2.0	1.8	0.01

Supplementary Table 19: Baseline patient characteristcs for ARB mono (T) and CCB/THZ mono (C) prevalent-users in the SIDIAP data source. We report proportion of initiators satisfying selected base-line characteristics and the standardized difference of population proportions (StdDiff) before and after stratification. Less extreme StdDiffs through stratification suggest improved balance between patient cohorts through propensity score adjustment.

	Before stratification			After stratification		
Characteristic	T (%)	C (%)	StdDiff	T (%)	C (%)	StdDiff
Age group						
15-19	0.0	0.0		0.0	0.0	
20-24	0.1	0.1	-0.01	0.1	0.1	-0.01
25-29	0.1	0.2	-0.03	0.1	0.2	-0.03
30-34	0.4	0.6	-0.03	0.4	0.6	-0.03
35-39	1.0	1.0	0.00	1.0	1.1	-0.01
40-44	2.7	2.3	0.02	2.7	2.3	0.02
45-49	4.6	5.0	-0.02	4.6	5.0	-0.02
50-54	8.0	7.9	0.00	7.9	7.8	0.00
55-59	10.9	10.2	0.02	10.8	10.4	0.01
60-64	13.3	12.5	0.02	12.9	12.7	0.01
65-69	14.5	13.4	0.03	13.7	13.7	0.00
70-74	15.1	14.6	0.01	15.0	14.7	0.01
75-79	11.9	12.7	-0.03	12.3	12.5	-0.01
80-84	8.3	9.0	-0.03	8.5	8.8	-0.01
85-89	5.9	6.5	-0.03	6.4	6.4	0.00
90-94	2.7	2.9	-0.02	2.9	2.8	0.00
95-99	0.6	0.8	-0.02	0.7	0.8	-0.01
100-104	0.1	0.1	0.02	0.1	0.1	0.03
Gender: female	50.5	53.0	-0.05	52.9	51.9	0.02
Medical history: General						
Acute respiratory disease	9.5	8.4	0.04	8.9	8.7	0.01
Attention deficit hyperactivity disorder	0.1	0.1	0.01	0.1	0.1	0.01
Chronic liver disease	1.5	1.7	-0.02	1.7	1.7	0.00
Chronic obstructive lung disease	6.4	7.0	-0.03	6.5	6.9	-0.02
Crohn’s disease	0.1	0.2	-0.01	0.2	0.2	0.00
Dementia	2.7	2.7	-0.01	2.8	2.6	0.01
Depressive disorder	14.0	14.8	-0.02	14.8	14.5	0.01
Diabetes mellitus	20.9	22.8	-0.05	22.2	22.2	0.00
Gastroesophageal reflux disease	12.1	11.5	0.02	12.0	11.6	0.01
Gastrointestinal hemorrhage	0.8	0.8	0.00	0.8	0.8	0.00
Human immunodeficiency virus infection	0.5	0.4	0.02	0.5	0.4	0.02
Hyperlipidemia	28.2	27.9	0.01	28.0	28.0	0.00
Hypertensive disorder	99.3	99.3	0.00	99.4	99.3	0.00
Lesion of liver	0.8	1.0	-0.03	0.8	1.0	-0.02
Obesity	34.9	37.5	-0.06	37.3	36.6	0.01
Osteoarthritis	32.1	33.2	-0.02	33.4	32.7	0.02
Pneumonia	0.6	0.6	0.00	0.6	0.6	0.00
Psoriasis	3.8	3.7	0.01	3.7	3.7	0.00
Renal impairment	11.3	13.8	-0.08	13.0	12.9	0.00

(Continued)	Before stratification			After stratification		
Characteristic	T (%)	C (%)	StdDiff	T (%)	C (%)	StdDiff
Rheumatoid arthritis	0.4	0.5	-0.01	0.4	0.5	-0.01
Schizophrenia	0.4	0.5	-0.01	0.5	0.5	-0.01
Ulcerative colitis	0.5	0.5	0.00	0.5	0.5	0.00
Urinary tract infectious disease	5.4	5.3	0.00	5.7	5.2	0.02
Viral hepatitis C	1.1	1.2	-0.01	1.2	1.2	0.01
Visual system disorder	38.5	42.0	-0.07	41.6	40.7	0.02
Medical history: Cardiovascular disease						
Atrial fibrillation	5.3	4.6	0.03	5.1	4.8	0.01
Cerebrovascular disease	2.4	2.4	0.00	2.4	2.4	0.00
Coronary arteriosclerosis	<0.1	0.0	0.02	<0.1	0.0	0.02
Heart disease	26.0	24.9	0.03	25.3	25.2	0.00
Heart failure	2.3	2.1	0.02	2.4	2.0	0.02
Ischemic heart disease	5.2	4.7	0.02	4.9	4.9	0.00
Peripheral vascular disease	3.0	4.3	-0.07	3.6	3.9	-0.01
Pulmonary embolism	0.6	0.6	0.01	0.7	0.6	0.01
Venous thrombosis	1.2	1.1	0.00	1.3	1.1	0.02
Medical history: Neoplasms						
Hematologic neoplasm	0.6	0.7	-0.01	0.6	0.7	0.00
Malignant lymphoma	0.4	0.4	0.00	0.4	0.4	0.00
Malignant neoplasm of anorectum	0.4	0.4	0.00	0.5	0.4	0.01
Malignant neoplastic disease	14.4	15.3	-0.02	14.8	15.1	-0.01
Malignant tumor of breast	2.3	2.0	0.02	2.3	2.1	0.01
Malignant tumor of colon	1.4	1.5	-0.01	1.5	1.5	-0.01
Malignant tumor of lung	0.1	0.2	-0.01	0.1	0.2	-0.01
Malignant tumor of urinary bladder	1.1	1.4	-0.03	1.1	1.3	-0.02
Primary malignant neoplasm of prostate	2.4	2.2	0.01	2.3	2.2	0.01
Medication use						
Antibacterials for systemic use	18.7	18.8	0.00	19.0	18.6	0.01
Antidepressants	17.7	18.1	-0.01	18.5	17.8	0.02
Antiepileptics	7.3	8.0	-0.03	7.9	7.7	0.01
Antiinflammatory and antirheumatic products	19.9	20.9	-0.02	20.8	20.4	0.01
Antineoplastic agents	0.6	0.6	0.00	0.7	0.6	0.01
Antipsoriatics	0.9	1.0	-0.01	0.9	1.0	-0.01
Antithrombotic agents	22.4	22.3	0.00	22.7	22.3	0.01
Drugs for acid related disorders	28.0	28.1	0.00	28.2	28.0	0.00
Drugs for obstructive airway diseases	8.5	7.9	0.02	8.0	8.1	0.00
Drugs used in diabetes	16.8	18.2	-0.04	18.1	17.7	0.01
Immunosuppressants	2.0	2.4	-0.03	2.1	2.3	-0.01
Lipid modifying agents	34.4	32.9	0.03	33.3	33.5	0.00
Opioids	9.7	11.4	-0.06	11.1	10.9	0.01
Psycholeptics	28.9	29.2	-0.01	29.6	28.9	0.02
Psychostimulants, agents used for adhd and nootropics	1.4	1.4	0.00	1.3	1.5	-0.01

Supplementary Table 20: Baseline patient characteristcs for ARB +combo (T) and CCB/THZ +combo (C) prevalent-users in the SIDIAP data source. We report proportion of initiators satisfying selected base-line characteristics and the standardized difference of population proportions (StdDiff) before and after stratification. Less extreme StdDiffs through stratification suggest improved balance between patient cohorts through propensity score adjustment.

	Before stratification			After stratification		
Characteristic	T (%)	C (%)	StdDiff	T (%)	C (%)	StdDiff
Age group						
15-19	0.0	0.0		0.0	0.0	
20-24	0.1	0.1	0.01	0.1	0.1	0.01
25-29	0.1	0.1	0.00	0.1	0.1	-0.01
30-34	0.3	0.4	-0.02	0.3	0.4	-0.02
35-39	0.9	1.1	-0.02	1.0	1.1	-0.01
40-44	2.5	2.6	0.00	2.9	2.5	0.02
45-49	4.3	5.6	-0.06	5.1	5.3	-0.01
50-54	7.5	8.8	-0.05	8.4	8.5	0.00
55-59	10.0	11.1	-0.04	11.3	10.9	0.01
60-64	12.5	13.3	-0.02	13.2	13.1	0.00
65-69	13.7	13.1	0.02	13.1	13.2	0.00
70-74	15.1	14.2	0.03	14.5	14.3	0.01
75-79	12.1	11.9	0.01	11.8	12.0	-0.01
80-84	9.1	8.1	0.04	8.0	8.3	-0.01
85-89	7.4	6.2	0.05	6.4	6.4	0.00
90-94	3.4	2.6	0.05	2.9	2.8	0.01
95-99	0.9	0.7	0.02	0.7	0.7	0.00
100-104	0.1	0.1	0.03	0.1	0.1	0.02
Gender: female	51.8	48.1	0.07	48.9	48.9	0.00
Medical history: General						
Acute respiratory disease	10.7	8.4	0.08	9.0	8.8	0.01
Attention deficit hyperactivity disorder	0.1	0.1	0.00	0.1	0.1	0.00
Chronic liver disease	1.7	1.9	-0.01	1.8	1.9	0.00
Chronic obstructive lung disease	7.5	7.1	0.02	7.0	7.2	-0.01
Crohn's disease	0.1	0.2	-0.01	0.1	0.2	0.00
Dementia	3.0	2.4	0.04	2.7	2.5	0.01
Depressive disorder	15.2	14.6	0.02	14.6	14.7	0.00
Diabetes mellitus	22.4	24.4	-0.05	24.4	24.0	0.01
Gastroesophageal reflux disease	12.4	10.7	0.05	11.2	11.0	0.01
Gastrointestinal hemorrhage	0.8	0.8	0.00	0.8	0.8	0.00
Human immunodeficiency virus infection	0.4	0.4	0.01	0.5	0.4	0.01
Hyperlipidemia	27.8	28.5	-0.02	28.5	28.4	0.00
Hypertensive disorder	99.3	99.5	-0.02	99.4	99.5	0.00
Lesion of liver	0.9	1.2	-0.03	1.0	1.2	-0.02
Obesity	36.7	40.6	-0.08	40.1	39.7	0.01
Osteoarthritis	33.6	31.4	0.05	31.8	31.8	0.00
Pneumonia	0.8	0.7	0.01	0.7	0.7	-0.01
Psoriasis	3.8	3.7	0.00	3.5	3.7	-0.01
Renal impairment	14.4	13.0	0.04	13.1	13.3	0.00

(Continued)	Before stratification			After stratification		
Characteristic	T (%)	C (%)	StdDiff	T (%)	C (%)	StdDiff
Rheumatoid arthritis	0.5	0.5	0.01	0.4	0.5	0.00
Schizophrenia	0.3	0.5	-0.02	0.4	0.5	-0.01
Ulcerative colitis	0.4	0.4	0.01	0.4	0.4	0.00
Urinary tract infectious disease	6.0	4.8	0.05	5.1	5.0	0.01
Viral hepatitis C	1.2	1.3	-0.01	1.3	1.3	0.00
Visual system disorder	40.0	40.2	0.00	40.1	40.2	0.00
Medical history: Cardiovascular disease						
Atrial fibrillation	9.0	5.9	0.12	6.7	6.4	0.01
Cerebrovascular disease	2.7	2.4	0.02	2.3	2.4	0.00
Coronary arteriosclerosis	0.1	0.0	0.02	0.1	0.0	0.01
Heart disease	33.0	27.1	0.13	28.1	28.2	0.00
Heart failure	5.8	2.9	0.14	3.8	3.3	0.03
Ischemic heart disease	8.2	6.1	0.08	6.4	6.5	0.00
Peripheral vascular disease	3.9	4.3	-0.02	4.1	4.3	-0.01
Pulmonary embolism	0.9	0.5	0.04	0.7	0.6	0.02
Venous thrombosis	1.4	1.2	0.01	1.2	1.2	0.00
Medical history: Neoplasms						
Hematologic neoplasm	0.7	0.6	0.00	0.6	0.6	0.00
Malignant lymphoma	0.4	0.5	-0.01	0.4	0.5	-0.02
Malignant neoplasm of anorectum	0.4	0.4	0.00	0.4	0.4	0.00
Malignant neoplastic disease	15.2	14.1	0.03	14.2	14.4	-0.01
Malignant tumor of breast	2.2	1.9	0.02	2.0	2.0	0.00
Malignant tumor of colon	1.7	1.5	0.02	1.6	1.6	0.00
Malignant tumor of lung	0.2	0.1	0.01	0.1	0.1	0.00
Malignant tumor of urinary bladder	1.3	1.2	0.01	1.2	1.2	0.00
Primary malignant neoplasm of prostate	2.3	2.2	0.01	2.1	2.2	-0.01
Medication use						
Antibacterials for systemic use	20.6	18.4	0.06	19.1	18.8	0.01
Antidepressants	19.4	18.0	0.04	18.4	18.2	0.00
Antiepileptics	8.0	8.1	0.00	8.1	8.1	0.00
Antiinflammatory and antirheumatic products	19.0	20.2	-0.03	20.2	19.9	0.01
Antineoplastic agents	0.7	0.6	0.01	0.6	0.6	0.00
Antipsoriatics	1.1	0.9	0.01	1.0	1.0	0.00
Antithrombotic agents	28.9	24.0	0.11	24.9	24.9	0.00
Drugs for acid related disorders	31.1	27.3	0.08	28.0	28.0	0.00
Drugs for obstructive airway diseases	9.1	7.2	0.07	7.6	7.5	0.00
Drugs used in diabetes	17.9	19.6	-0.04	19.7	19.2	0.01
Immunosuppressants	2.1	1.8	0.02	2.0	1.8	0.01
Lipid modifying agents	36.1	34.3	0.04	34.4	34.7	0.00
Opioids	11.0	11.5	-0.02	11.5	11.4	0.00
Psycholeptics	31.1	28.9	0.05	29.4	29.3	0.00
Psychostimulants, agents used for adhd and nootropics	2.1	1.8	0.03	1.7	1.9	-0.01

Supplementary Table 21: Baseline patient characteristcs for ARB mono (T) and CCB mono (C) prevalent-users in the SIDIAP data source. We report proportion of initiators satisfying selected base-line characteristics and the standardized difference of population proportions (StdDiff) before and after stratification. Less extreme StdDiffs through stratification suggest improved balance between patient cohorts through propensity score adjustment.

	Before stratification			After stratification		
Characteristic	T (%)	C (%)	StdDiff	T (%)	C (%)	StdDiff
Age group						
15-19	0.0	<0.1		0.0	<0.1	
20-24	0.1	0.2	-0.02	0.1	0.2	-0.03
25-29	0.1	0.3	-0.04	0.1	0.3	-0.04
30-34	0.4	0.6	-0.04	0.4	0.7	-0.04
35-39	1.0	1.1	-0.01	0.9	1.1	-0.02
40-44	2.7	2.5	0.01	2.6	2.6	0.00
45-49	4.6	5.0	-0.02	4.5	5.0	-0.02
50-54	8.0	8.0	0.00	7.9	8.0	0.00
55-59	10.9	10.6	0.01	10.9	10.7	0.01
60-64	13.3	12.4	0.03	13.0	12.6	0.01
65-69	14.5	13.1	0.04	13.6	13.5	0.00
70-74	15.1	14.1	0.03	14.8	14.1	0.02
75-79	11.9	12.3	-0.01	12.1	12.2	0.00
80-84	8.3	9.1	-0.03	8.7	8.8	0.00
85-89	5.9	6.9	-0.04	6.5	6.5	0.00
90-94	2.7	2.9	-0.01	3.0	2.7	0.01
95-99	0.6	0.9	-0.03	0.7	0.9	-0.02
100-104	0.1	<0.1	0.02	0.1	<0.1	0.03
Gender: female	50.5	44.4	0.12	46.7	47.5	-0.02
Medical history: General						
Acute respiratory disease	9.5	8.5	0.04	9.0	8.8	0.00
Attention deficit hyperactivity disorder	0.1	0.1	0.01	0.1	0.1	0.01
Chronic liver disease	1.5	2.1	-0.04	1.7	1.9	-0.01
Chronic obstructive lung disease	6.4	7.8	-0.06	6.9	7.2	-0.01
Crohn's disease	0.1	0.1	0.00	0.1	0.2	-0.01
Dementia	2.7	2.8	-0.01	3.0	2.6	0.02
Depressive disorder	14.0	14.6	-0.02	14.3	14.4	0.00
Diabetes mellitus	20.9	26.5	-0.13	24.3	23.7	0.01
Gastroesophageal reflux disease	12.1	11.4	0.02	12.0	11.5	0.01
Gastrointestinal hemorrhage	0.8	1.0	-0.02	0.8	0.9	-0.01
Human immunodeficiency virus infection	0.5	0.4	0.02	0.5	0.3	0.03
Hyperlipidemia	28.2	27.9	0.01	28.3	27.8	0.01
Hypertensive disorder	99.3	99.4	-0.01	99.4	99.4	0.00
Lesion of liver	0.8	1.3	-0.05	0.9	1.1	-0.03
Obesity	34.9	37.7	-0.06	36.8	36.3	0.01
Osteoarthritis	32.1	31.2	0.02	31.8	31.5	0.01
Pneumonia	0.6	0.6	0.00	0.6	0.6	0.00
Psoriasis	3.8	3.8	0.00	3.7	3.8	0.00
Renal impairment	11.3	17.6	-0.18	15.1	14.5	0.02

(Continued)	Before stratification			After stratification		
Characteristic	T (%)	C (%)	StdDiff	T (%)	C (%)	StdDiff
Rheumatoid arthritis	0.4	0.4	0.00	0.4	0.4	0.00
Schizophrenia	0.4	0.6	-0.02	0.5	0.6	-0.01
Ulcerative colitis	0.5	0.5	-0.01	0.5	0.5	0.00
Urinary tract infectious disease	5.4	5.0	0.02	5.5	5.1	0.02
Viral hepatitis C	1.1	1.5	-0.03	1.3	1.4	0.00
Visual system disorder	38.5	41.8	-0.07	40.6	40.4	0.00
Medical history: Cardiovascular disease						
Atrial fibrillation	5.3	5.4	0.00	5.6	5.3	0.02
Cerebrovascular disease	2.4	2.9	-0.03	2.6	2.6	0.00
Coronary arteriosclerosis	<0.1	<0.1	0.02	<0.1	<0.1	0.02
Heart disease	26.0	27.8	-0.04	27.4	26.7	0.02
Heart failure	2.3	2.8	-0.03	2.6	2.5	0.01
Ischemic heart disease	5.2	6.2	-0.04	6.0	5.7	0.01
Peripheral vascular disease	3.0	5.4	-0.12	4.1	4.4	-0.01
Pulmonary embolism	0.6	0.6	0.00	0.7	0.6	0.02
Venous thrombosis	1.2	1.1	0.01	1.3	1.0	0.03
Medical history: Neoplasms						
Hematologic neoplasm	0.6	0.8	-0.02	0.6	0.8	-0.02
Malignant lymphoma	0.4	0.4	-0.01	0.4	0.5	0.00
Malignant neoplasm of anorectum	0.4	0.5	-0.01	0.5	0.4	0.01
Malignant neoplastic disease	14.4	15.8	-0.04	15.0	15.3	-0.01
Malignant tumor of breast	2.3	1.6	0.05	2.0	1.8	0.02
Malignant tumor of colon	1.4	1.5	-0.01	1.6	1.5	0.01
Malignant tumor of lung	0.1	0.2	-0.01	0.1	0.2	-0.01
Malignant tumor of urinary bladder	1.1	1.8	-0.06	1.3	1.5	-0.02
Primary malignant neoplasm of prostate	2.4	2.5	-0.01	2.6	2.4	0.02
Medication use						
Antibacterials for systemic use	18.7	18.9	0.00	18.9	18.6	0.01
Antidepressants	17.7	17.9	0.00	18.0	17.9	0.00
Antiepileptics	7.3	8.1	-0.03	7.7	7.7	0.00
Antiinflammatory and antirheumatic products	19.9	18.8	0.03	19.2	19.3	0.00
Antineoplastic agents	0.6	0.5	0.02	0.6	0.5	0.02
Antipsoriatics	0.9	1.2	-0.04	1.0	1.1	-0.01
Antithrombotic agents	22.4	25.7	-0.08	24.8	23.9	0.02
Drugs for acid related disorders	28.0	29.6	-0.04	29.0	28.6	0.01
Drugs for obstructive airway diseases	8.5	7.6	0.03	8.1	7.9	0.01
Drugs used in diabetes	16.8	21.3	-0.12	19.7	19.0	0.02
Immunosuppressants	2.0	2.8	-0.05	2.2	2.6	-0.02
Lipid modifying agents	34.4	35.3	-0.02	35.1	34.6	0.01
Opioids	9.7	11.4	-0.05	10.8	10.8	0.00
Psycholeptics	28.9	29.1	0.00	29.5	28.7	0.02
Psychostimulants, agents used for adhd and nootropics	1.4	1.8	-0.03	1.6	1.7	-0.01

Supplementary Table 22: Baseline patient characteristcs for ARB +combo (T) and CCB +combo (C) prevalent-users in the SIDIAP data source. We report proportion of initiators satisfying selected base-line characteristics and the standardized difference of population proportions (StdDiff) before and after stratification. Less extreme StdDiffs through stratification suggest improved balance between patient cohorts through propensity score adjustment.

	Before stratification			After stratification		
Characteristic	T (%)	C (%)	StdDiff	T (%)	C (%)	StdDiff
Age group						
15-19	0.0	0.0		0.0	0.0	
20-24	0.1	0.1	-0.02	0.1	0.1	-0.01
25-29	0.1	0.2	-0.01	0.1	0.2	-0.01
30-34	0.3	0.5	-0.04	0.3	0.5	-0.03
35-39	0.7	1.1	-0.04	0.9	1.0	-0.01
40-44	2.3	2.6	-0.02	2.4	2.5	-0.01
45-49	4.4	5.0	-0.03	4.6	4.7	0.00
50-54	7.6	8.3	-0.03	7.9	8.0	0.00
55-59	9.9	10.4	-0.02	10.2	10.3	0.00
60-64	12.6	12.4	0.01	12.6	12.4	0.00
65-69	14.0	12.6	0.04	13.3	13.2	0.00
70-74	15.5	13.8	0.05	15.1	14.0	0.03
75-79	12.9	12.4	0.02	12.6	12.6	0.00
80-84	9.1	9.0	0.00	8.9	9.1	-0.01
85-89	6.7	7.3	-0.02	7.1	7.1	0.00
90-94	3.0	3.4	-0.02	3.2	3.3	-0.01
95-99	0.7	0.9	-0.01	0.8	0.9	-0.01
100-104	0.1	0.1	0.02	0.1	0.1	0.03
Gender: female	52.6	42.5	0.20	46.6	47.8	-0.03
Medical history: General						
Acute respiratory disease	10.1	8.5	0.06	9.3	9.2	0.00
Attention deficit hyperactivity disorder	0.1	0.1	0.00	0.1	0.1	0.00
Chronic liver disease	1.7	2.2	-0.03	1.9	2.0	0.00
Chronic obstructive lung disease	7.1	8.3	-0.04	7.7	7.8	0.00
Crohn's disease	0.1	0.2	-0.01	0.1	0.2	-0.01
Dementia	2.5	2.9	-0.03	2.8	2.7	0.01
Depressive disorder	15.1	14.8	0.01	14.8	14.8	0.00
Diabetes mellitus	23.1	28.3	-0.12	26.0	25.6	0.01
Gastroesophageal reflux disease	12.6	11.0	0.05	11.9	11.7	0.01
Gastrointestinal hemorrhage	0.8	0.9	-0.02	0.8	0.9	-0.01
Human immunodeficiency virus infection	0.4	0.4	-0.01	0.5	0.4	0.01
Hyperlipidemia	28.5	28.4	0.00	28.7	28.4	0.00
Hypertensive disorder	99.5	99.6	-0.01	99.5	99.6	-0.01
Lesion of liver	0.9	1.5	-0.06	1.1	1.2	-0.01
Obesity	39.4	40.0	-0.01	39.8	39.7	0.00
Osteoarthritis	33.9	31.3	0.06	32.5	32.5	0.00
Pneumonia	0.7	0.8	-0.01	0.7	0.8	0.00
Psoriasis	3.9	3.8	0.00	3.9	3.8	0.00
Renal impairment	13.2	18.4	-0.14	15.9	15.8	0.00

(Continued)	Before stratification			After stratification		
Characteristic	T (%)	C (%)	StdDiff	T (%)	C (%)	StdDiff
Rheumatoid arthritis	0.5	0.4	0.01	0.5	0.5	0.00
Schizophrenia	0.3	0.6	-0.04	0.3	0.5	-0.02
Ulcerative colitis	0.5	0.4	0.01	0.5	0.5	0.01
Urinary tract infectious disease	5.7	4.8	0.04	5.3	5.2	0.00
Viral hepatitis C	1.3	1.5	-0.01	1.4	1.4	0.00
Visual system disorder	40.0	41.2	-0.03	40.7	40.7	0.00
Medical history: Cardiovascular disease						
Atrial fibrillation	8.1	7.5	0.02	7.9	7.8	0.00
Cerebrovascular disease	2.6	3.1	-0.03	2.8	2.9	0.00
Coronary arteriosclerosis	0.0	0.0	0.00	0.0	0.0	0.00
Heart disease	31.8	32.2	-0.01	32.2	31.9	0.01
Heart failure	4.4	4.4	0.00	4.5	4.3	0.01
Ischemic heart disease	7.5	8.6	-0.04	8.1	8.0	0.00
Peripheral vascular disease	3.7	6.0	-0.11	4.8	4.9	0.00
Pulmonary embolism	0.7	0.7	0.00	0.7	0.7	0.00
Venous thrombosis	1.3	1.3	0.00	1.3	1.3	0.00
Medical history: Neoplasms						
Hematologic neoplasm	0.6	0.9	-0.03	0.6	0.8	-0.02
Malignant lymphoma	0.4	0.5	-0.03	0.4	0.5	-0.01
Malignant neoplasm of anorectum	0.4	0.4	0.00	0.4	0.4	0.00
Malignant neoplastic disease	14.8	15.9	-0.03	15.4	15.4	0.00
Malignant tumor of breast	2.3	1.6	0.05	1.9	1.9	0.00
Malignant tumor of colon	1.7	1.7	0.00	1.9	1.7	0.01
Malignant tumor of lung	0.2	0.2	0.00	0.2	0.1	0.00
Malignant tumor of urinary bladder	1.1	1.6	-0.04	1.4	1.4	0.00
Primary malignant neoplasm of prostate	2.2	2.6	-0.03	2.5	2.4	0.01
Medication use						
Antibacterials for systemic use	19.6	19.3	0.01	19.5	19.5	0.00
Antidepressants	19.2	18.2	0.02	18.7	18.6	0.00
Antiepileptics	8.2	8.8	-0.02	8.4	8.5	0.00
Antiinflammatory and antirheumatic products	19.3	18.0	0.03	18.7	18.6	0.00
Antineoplastic agents	0.6	0.5	0.01	0.6	0.5	0.01
Antipsoriatics	0.9	1.3	-0.04	1.1	1.1	-0.01
Antithrombotic agents	27.6	29.6	-0.04	28.9	28.6	0.01
Drugs for acid related disorders	30.5	30.8	-0.01	30.6	30.5	0.00
Drugs for obstructive airway diseases	8.4	7.7	0.03	8.0	8.1	0.00
Drugs used in diabetes	18.5	23.0	-0.11	21.1	20.6	0.01
Immunosuppressants	1.9	2.6	-0.05	2.1	2.3	-0.01
Lipid modifying agents	36.5	37.1	-0.01	36.9	36.7	0.00
Opioids	11.4	11.9	-0.02	11.8	11.7	0.00
Psycholeptics	31.2	29.9	0.03	30.5	30.6	0.00
Psychostimulants, agents used for adhd and nootropics	2.0	2.5	-0.04	2.2	2.3	0.00

Supplementary Table 23: Baseline patient characteristcs for ARB mono (T) and THZ mono (C) prevalent-users in the SIDIAP data source. We report proportion of initiators satisfying selected base-line characteristics and the standardized difference of population proportions (StdDiff) before and after stratification. Less extreme StdDiffs through stratification suggest improved balance between patient cohorts through propensity score adjustment.

	Before stratification			After stratification		
Characteristic	T (%)	C (%)	StdDiff	T (%)	C (%)	StdDiff
Age group						
15-19	0.0	<0.1		0.0	<0.1	
20-24	0.1	<0.1	0.03	0.1	<0.1	0.03
25-29	0.1	0.1	-0.01	0.1	0.1	0.00
30-34	0.4	0.5	-0.02	0.3	0.5	-0.02
35-39	1.0	1.0	0.00	1.0	0.9	0.01
40-44	2.7	2.0	0.04	2.6	2.1	0.04
45-49	4.6	5.0	-0.02	4.7	4.9	-0.01
50-54	8.0	7.8	0.01	8.1	7.4	0.02
55-59	10.9	9.8	0.04	10.6	10.3	0.01
60-64	13.3	12.7	0.02	13.1	12.5	0.02
65-69	14.5	13.7	0.02	14.4	13.9	0.01
70-74	15.1	15.5	-0.01	15.0	15.6	-0.02
75-79	11.9	13.5	-0.05	12.5	13.0	-0.02
80-84	8.3	8.7	-0.01	8.3	8.7	-0.02
85-89	5.9	6.0	0.00	5.9	6.1	-0.01
90-94	2.7	2.8	-0.01	2.5	2.9	-0.02
95-99	0.6	0.7	-0.01	0.6	0.7	-0.02
100-104	0.1	<0.1	0.01	0.1	<0.1	0.00
Gender: female	50.5	67.6	-0.36	58.8	56.2	0.05
Medical history: General						
Acute respiratory disease	9.5	8.2	0.05	9.1	8.8	0.01
Attention deficit hyperactivity disorder	0.1	<0.1	0.01	0.1	<0.1	0.01
Chronic liver disease	1.5	1.1	0.04	1.5	1.1	0.04
Chronic obstructive lung disease	6.4	5.5	0.04	5.9	6.7	-0.03
Crohn’s disease	0.1	0.2	-0.02	0.2	0.2	-0.01
Dementia	2.7	2.7	0.00	2.6	2.9	-0.01
Depressive disorder	14.0	14.9	-0.03	14.7	14.3	0.01
Diabetes mellitus	20.9	16.4	0.12	19.2	19.4	0.00
Gastroesophageal reflux disease	12.1	11.5	0.02	12.0	11.5	0.02
Gastrointestinal hemorrhage	0.8	0.6	0.02	0.7	0.6	0.01
Human immunodeficiency virus infection	0.5	0.3	0.03	0.4	0.4	0.01
Hyperlipidemia	28.2	27.6	0.01	27.7	28.0	-0.01
Hypertensive disorder	99.3	99.2	0.02	99.3	99.2	0.01
Lesion of liver	0.8	0.7	0.01	0.8	0.7	0.01
Obesity	34.9	37.1	-0.04	35.8	35.9	0.00
Osteoarthritis	32.1	36.4	-0.09	34.3	33.6	0.01
Pneumonia	0.6	0.5	0.01	0.5	0.6	-0.02
Psoriasis	3.8	3.4	0.02	3.6	3.6	0.00
Renal impairment	11.3	7.3	0.14	9.7	9.4	0.01

(Continued)	Before stratification			After stratification		
Characteristic	T (%)	C (%)	StdDiff	T (%)	C (%)	StdDiff
Rheumatoid arthritis	0.4	0.6	-0.02	0.4	0.5	-0.01
Schizophrenia	0.4	0.4	0.01	0.4	0.4	0.00
Ulcerative colitis	0.5	0.4	0.00	0.5	0.5	0.00
Urinary tract infectious disease	5.4	5.6	-0.01	5.8	5.4	0.01
Viral hepatitis C	1.1	0.8	0.04	1.1	0.8	0.04
Visual system disorder	38.5	42.4	-0.08	40.4	40.4	0.00
Medical history: Cardiovascular disease						
Atrial fibrillation	5.3	3.3	0.10	4.6	4.5	0.01
Cerebrovascular disease	2.4	1.6	0.06	2.2	1.9	0.02
Coronary arteriosclerosis	<0.1	0.0		<0.1	0.0	
Heart disease	26.0	19.8	0.15	23.4	23.4	0.00
Heart failure	2.3	0.9	0.11	2.0	1.4	0.04
Ischemic heart disease	5.2	2.1	0.16	4.1	3.6	0.03
Peripheral vascular disease	3.0	2.1	0.05	2.6	2.8	-0.01
Pulmonary embolism	0.6	0.5	0.02	0.6	0.5	0.00
Venous thrombosis	1.2	1.1	0.00	1.1	1.1	0.00
Medical history: Neoplasms						
Hematologic neoplasm	0.6	0.5	0.02	0.6	0.4	0.03
Malignant lymphoma	0.4	0.4	0.00	0.4	0.4	0.00
Malignant neoplasm of anorectum	0.4	0.3	0.02	0.4	0.3	0.02
Malignant neoplastic disease	14.4	14.0	0.01	14.1	14.1	0.00
Malignant tumor of breast	2.3	2.8	-0.03	2.6	2.3	0.02
Malignant tumor of colon	1.4	1.5	-0.01	1.3	1.5	-0.01
Malignant tumor of lung	0.1	0.1	0.00	0.1	0.1	0.00
Malignant tumor of urinary bladder	1.1	0.7	0.04	0.9	0.9	0.00
Primary malignant neoplasm of prostate	2.4	1.6	0.06	2.0	2.1	-0.01
Medication use						
Antibacterials for systemic use	18.7	18.7	0.00	18.8	18.5	0.01
Antidepressants	17.7	18.7	-0.03	18.4	18.0	0.01
Antiepileptics	7.3	7.8	-0.02	7.5	7.6	0.00
Antiinflammatory and antirheumatic products	19.9	24.7	-0.12	21.9	21.6	0.01
Antineoplastic agents	0.6	0.8	-0.02	0.7	0.8	-0.01
Antipsoriatics	0.9	0.6	0.03	0.8	0.7	0.00
Antithrombotic agents	22.4	16.3	0.16	20.0	19.9	0.00
Drugs for acid related disorders	28.0	25.4	0.06	27.1	26.6	0.01
Drugs for obstructive airway diseases	8.5	8.1	0.01	8.3	8.4	0.00
Drugs used in diabetes	16.8	12.7	0.12	15.2	15.3	0.00
Immunosuppressants	2.0	1.6	0.03	1.9	1.6	0.02
Lipid modifying agents	34.4	28.4	0.13	31.8	32.5	-0.01
Opioids	9.7	11.2	-0.05	10.4	10.2	0.01
Psycholeptics	28.9	29.6	-0.01	29.5	28.8	0.01
Psychostimulants, agents used for adhd and nootropics	1.4	0.6	0.08	1.2	1.0	0.02

Supplementary Table 24: Baseline patient characteristcs for ARB +combo (T) and THZ +combo (C) prevalent-users in the SIDIAP data source. We report proportion of initiators satisfying selected base-line characteristics and the standardized difference of population proportions (StdDiff) before and after stratification. Less extreme StdDiffs through stratification suggest improved balance between patient cohorts through propensity score adjustment.

	Before stratification			After stratification		
Characteristic	T (%)	C (%)	StdDiff	T (%)	C (%)	StdDiff
Age group						
15-19	0.0	0.0	0.00	0.0	0.0	0.00
20-24	0.1	0.0	0.03	0.1	0.0	0.04
25-29	0.2	0.1	0.01	0.2	0.1	0.01
30-34	0.3	0.4	-0.01	0.3	0.3	0.00
35-39	0.9	1.1	-0.02	1.0	1.0	0.00
40-44	2.5	2.7	-0.01	2.8	2.6	0.01
45-49	4.3	6.0	-0.08	5.3	5.5	-0.01
50-54	7.6	9.4	-0.07	8.7	8.8	0.00
55-59	10.0	11.8	-0.06	11.6	11.1	0.01
60-64	12.4	14.0	-0.05	13.2	13.5	-0.01
65-69	13.7	13.5	0.00	13.5	13.5	0.00
70-74	15.0	14.2	0.02	14.6	14.6	0.00
75-79	12.3	11.4	0.03	11.7	11.8	0.00
80-84	9.0	7.3	0.06	7.7	8.0	-0.01
85-89	7.4	5.3	0.09	5.9	5.9	0.00
90-94	3.4	2.1	0.08	2.6	2.4	0.01
95-99	0.9	0.6	0.04	0.7	0.7	0.00
100-104	0.1	0.0	0.03	0.1	0.1	0.02
Gender: female	49.7	51.3	-0.03	50.9	50.9	0.00
Medical history: General						
Acute respiratory disease	10.4	8.2	0.07	9.1	8.9	0.00
Attention deficit hyperactivity disorder	0.1	0.1	0.00	0.1	0.1	0.00
Chronic liver disease	1.7	1.7	0.00	1.7	1.7	0.00
Chronic obstructive lung disease	7.8	6.3	0.06	6.7	6.8	0.00
Crohn's disease	0.2	0.1	0.01	0.2	0.2	0.01
Dementia	2.9	2.0	0.06	2.3	2.2	0.00
Depressive disorder	15.3	14.3	0.03	14.5	14.6	0.00
Diabetes mellitus	23.6	22.2	0.03	22.9	22.5	0.01
Gastroesophageal reflux disease	12.2	10.4	0.06	11.1	11.0	0.00
Gastrointestinal hemorrhage	0.8	0.7	0.01	0.7	0.7	0.00
Human immunodeficiency virus infection	0.4	0.4	0.00	0.4	0.4	0.00
Hyperlipidemia	27.9	28.8	-0.02	28.7	28.6	0.00
Hypertensive disorder	99.4	99.4	0.00	99.5	99.4	0.01
Lesion of liver	1.0	0.9	0.00	0.9	1.0	-0.01
Obesity	36.9	41.6	-0.10	40.2	39.8	0.01
Osteoarthritis	33.1	31.1	0.04	31.7	31.7	0.00
Pneumonia	0.9	0.6	0.02	0.7	0.7	0.00
Psoriasis	3.9	3.7	0.01	3.6	3.7	-0.01
Renal impairment	15.3	8.8	0.20	10.9	10.6	0.01

(Continued)	Before stratification			After stratification		
Characteristic	T (%)	C (%)	StdDiff	T (%)	C (%)	StdDiff
Rheumatoid arthritis	0.5	0.5	0.01	0.5	0.5	-0.01
Schizophrenia	0.3	0.4	-0.02	0.3	0.4	-0.01
Ulcerative colitis	0.4	0.4	0.01	0.4	0.4	0.00
Urinary tract infectious disease	5.9	4.6	0.06	5.1	5.0	0.00
Viral hepatitis C	1.2	1.2	0.00	1.2	1.2	0.00
Visual system disorder	40.1	39.2	0.02	39.5	39.5	0.00
Medical history: Cardiovascular disease						
Atrial fibrillation	9.1	4.6	0.18	6.3	5.8	0.02
Cerebrovascular disease	2.8	1.9	0.06	2.3	2.1	0.01
Coronary arteriosclerosis	0.1	0.0	0.03	0.0	0.0	0.02
Heart disease	33.3	23.3	0.22	26.5	26.2	0.01
Heart failure	5.7	1.7	0.21	3.5	2.4	0.06
Ischemic heart disease	8.7	4.1	0.19	5.7	5.4	0.02
Peripheral vascular disease	4.2	3.3	0.05	3.6	3.6	0.00
Pulmonary embolism	0.9	0.4	0.06	0.7	0.5	0.02
Venous thrombosis	1.4	1.2	0.02	1.1	1.3	-0.01
Medical history: Neoplasms						
Hematologic neoplasm	0.7	0.4	0.03	0.5	0.5	0.01
Malignant lymphoma	0.5	0.4	0.01	0.4	0.4	-0.01
Malignant neoplasm of anorectum	0.4	0.4	0.00	0.3	0.4	-0.01
Malignant neoplastic disease	15.4	12.7	0.08	13.5	13.5	0.00
Malignant tumor of breast	2.1	2.0	0.01	2.0	2.1	-0.01
Malignant tumor of colon	1.7	1.3	0.03	1.4	1.5	-0.01
Malignant tumor of lung	0.2	0.1	0.02	0.1	0.1	0.01
Malignant tumor of urinary bladder	1.4	0.9	0.05	1.2	1.0	0.02
Primary malignant neoplasm of prostate	2.5	1.9	0.04	2.0	2.1	-0.01
Medication use						
Antibacterials for systemic use	20.6	17.3	0.08	18.5	18.3	0.01
Antidepressants	19.1	17.5	0.04	18.0	18.0	0.00
Antiepileptics	8.0	7.6	0.01	7.8	7.7	0.00
Antiinflammatory and antirheumatic products	18.5	21.5	-0.07	20.9	20.6	0.01
Antineoplastic agents	0.6	0.6	0.01	0.5	0.7	-0.03
Antipsoriatics	1.1	0.7	0.05	0.9	0.7	0.01
Antithrombotic agents	30.0	19.9	0.23	23.2	23.0	0.00
Drugs for acid related disorders	31.3	24.5	0.15	26.6	26.5	0.00
Drugs for obstructive airway diseases	9.0	6.9	0.08	7.6	7.5	0.00
Drugs used in diabetes	19.0	17.7	0.03	18.5	18.0	0.01
Immunosuppressants	2.2	1.1	0.09	1.6	1.3	0.02
Lipid modifying agents	37.1	32.5	0.10	33.9	33.8	0.00
Opioids	11.0	11.1	0.00	11.2	11.1	0.00
Psycholeptics	30.9	27.8	0.07	28.9	28.8	0.00
Psychostimulants, agents used for adhd and nootropics	2.1	1.2	0.08	1.5	1.5	0.00

VA-OMOP

Supplementary Table 25: Baseline patient characteristcs for ACEI/ARB mono (T) and CCB/THZ mono (C) prevalent-users in the VA-OMOP data source. We report proportion of initiators satisfying selected base-line characteristics and the standardized difference of population proportions (StdDiff) before and after stratification. Less extreme StdDiffs through stratification suggest improved balance between patient cohorts through propensity score adjustment.

	Before stratification			After stratification		
Characteristic	T (%)	C (%)	StdDiff	T (%)	C (%)	StdDiff
Age group						
25-29	0.2	0.2	0.00	0.2	0.2	0.00
30-34	0.8	0.9	-0.01	0.8	0.8	0.00
35-39	1.9	2.0	-0.01	2.0	1.9	0.01
40-44	2.7	2.8	-0.01	2.7	2.6	0.01
45-49	4.6	4.4	0.01	4.6	4.4	0.01
50-54	7.2	7.1	0.01	7.2	6.9	0.01
55-59	9.5	10.0	-0.02	9.6	9.4	0.01
60-64	12.0	13.6	-0.05	12.6	12.4	0.00
65-69	14.5	14.8	-0.01	14.5	14.6	0.00
70-74	25.2	22.2	0.07	23.9	24.2	-0.01
75-79	10.4	9.5	0.03	10.1	10.4	-0.01
80-84	5.2	5.1	0.00	5.2	5.4	-0.01
85-89	3.9	4.4	-0.03	4.2	4.3	-0.01
90-94	1.5	2.1	-0.04	1.8	1.9	-0.01
95-99	0.4	0.7	-0.04	0.5	0.6	0.00
Gender: female	5.2	9.0	-0.15	6.4	6.8	-0.02
Race						
race = Asian	1.2	0.8	0.04	1.1	1.1	0.00
race = Black or African American	12.9	33.5	-0.50	21.1	21.3	0.00
race = White	77.5	58.6	0.41	70.0	69.9	0.00
race = Unknown	6.6	5.7	0.03	6.2	6.2	0.00
race = Native Hawaiian or Other Pacific Islander	1.0	0.8	0.03	0.9	0.9	0.00
race = American Indian or Alaska Native	0.9	0.6	0.03	0.7	0.7	0.00
Ethnicity						
ethnicity = Hispanic or Latino	7.6	5.2	0.10	6.7	6.5	0.01
ethnicity = Not Hispanic or Latino	89.7	92.4	-0.09	90.8	90.9	0.00
Medical history: General						
Acute respiratory disease	4.3	5.0	-0.04	4.6	4.6	0.00
Attention deficit hyperactivity disorder	0.7	0.6	0.02	0.7	0.7	0.00
Chronic liver disease	1.6	2.5	-0.06	1.9	2.0	-0.01
Chronic obstructive lung disease	7.7	9.3	-0.06	8.4	8.6	-0.01
Crohn's disease	0.2	0.2	-0.01	0.2	0.2	0.00
Dementia	2.0	2.4	-0.03	2.2	2.3	0.00
Depressive disorder	16.7	17.7	-0.03	17.1	17.0	0.00
Diabetes mellitus	37.8	16.3	0.50	29.5	28.3	0.03
Gastroesophageal reflux disease	13.9	14.2	-0.01	14.1	14.3	-0.01
Gastrointestinal hemorrhage	0.6	0.8	-0.02	0.7	0.8	-0.01

(Continued)	Before stratification			After stratification		
Characteristic	T (%)	C (%)	StdDiff	T (%)	C (%)	StdDiff
Human immunodeficiency virus infection	0.5	0.8	-0.03	0.6	0.6	0.00
Hyperlipidemia	48.2	39.6	0.17	44.7	44.2	0.01
Hypertensive disorder	68.4	71.1	-0.06	69.4	70.0	-0.01
Lesion of liver	1.2	1.6	-0.03	1.3	1.4	-0.01
Obesity	12.7	10.6	0.06	11.9	11.6	0.01
Osteoarthritis	14.5	15.8	-0.04	15.0	15.1	0.00
Pneumonia	0.7	0.9	-0.03	0.8	0.8	-0.01
Psoriasis	1.2	1.0	0.02	1.1	1.1	0.00
Renal impairment	6.1	7.1	-0.04	6.7	7.0	-0.01
Rheumatoid arthritis	0.7	0.8	0.00	0.8	0.7	0.00
Schizophrenia	0.8	1.2	-0.03	1.0	1.0	-0.01
Ulcerative colitis	0.3	0.3	0.00	0.3	0.3	0.00
Urinary tract infectious disease	1.3	1.5	-0.02	1.4	1.4	0.00
Viral hepatitis C	1.0	2.0	-0.08	1.3	1.5	-0.01
Visual system disorder	28.5	27.7	0.02	28.2	28.1	0.00
Medical history: Cardiovascular disease						
Atrial fibrillation	2.8	2.3	0.04	2.7	2.8	-0.01
Cerebrovascular disease	2.3	2.3	0.00	2.3	2.5	-0.01
Coronary arteriosclerosis	6.4	4.4	0.09	5.8	5.8	0.00
Heart disease	14.0	11.9	0.06	13.3	13.6	-0.01
Heart failure	1.1	0.8	0.03	1.0	0.9	0.01
Ischemic heart disease	1.9	1.5	0.03	1.7	1.8	0.00
Peripheral vascular disease	2.7	2.5	0.01	2.7	2.7	0.00
Pulmonary embolism	0.4	0.4	0.00	0.4	0.4	0.00
Venous thrombosis	0.8	0.9	-0.02	0.8	0.9	0.00
Medical history: Neoplasms						
Hematologic neoplasm	1.0	1.2	-0.02	1.1	1.1	0.00
Malignant lymphoma	0.6	0.6	0.00	0.6	0.6	0.00
Malignant neoplasm of anorectum	0.1	0.1	0.00	0.1	0.1	0.00
Malignant neoplastic disease	7.9	9.7	-0.06	8.6	8.9	-0.01
Malignant tumor of breast	0.1	0.1	-0.01	0.1	0.1	0.00
Malignant tumor of colon	0.3	0.4	-0.01	0.3	0.3	0.00
Malignant tumor of lung	0.3	0.5	-0.03	0.4	0.4	0.00
Primary malignant neoplasm of prostate	3.0	4.1	-0.06	3.4	3.5	-0.01
Medication use						
Antibacterials for systemic use	15.6	17.6	-0.05	16.3	16.6	-0.01
Antidepressants	31.8	31.6	0.00	31.8	31.8	0.00
Antiepileptics	22.8	21.2	0.04	22.3	22.3	0.00
Antiinflammatory and antirheumatic products	29.8	33.3	-0.07	31.1	31.2	0.00
Antineoplastic agents	2.5	2.9	-0.02	2.6	2.7	0.00
Antipsoriatics	0.6	0.8	-0.02	0.7	0.7	0.00
Drugs for acid related disorders	34.2	33.8	0.01	34.0	34.6	-0.01
Drugs for obstructive airway diseases	26.1	28.7	-0.06	27.1	27.5	-0.01
Drugs used in diabetes	39.4	14.8	0.58	29.6	28.5	0.02
Immunosuppressants	2.6	2.9	-0.02	2.8	2.8	0.00

(Continued)	Before stratification			After stratification		
Characteristic	T (%)	C (%)	StdDiff	T (%)	C (%)	StdDiff
Lipid modifying agents	64.8	50.4	0.30	58.9	58.1	0.02
Opioids	9.2	9.8	-0.02	9.5	9.6	-0.01
Psycholeptics	19.0	20.6	-0.04	19.7	19.9	-0.01
Psychostimulants, agents used for adhd and nootropics	1.4	1.4	0.01	1.4	1.4	0.00

Supplementary Table 26: Baseline patient characteristcs for ACEI/ARB +combo (T) and CCB/THZ +combo (C) prevalent-users in the VA-OMOP data source. We report proportion of initiators satisfying selected base-line characteristics and the standardized difference of population proportions (StdDiff) before and after stratification. Less extreme StdDiffs through stratification suggest improved balance between patient cohorts through propensity score adjustment.

	Before stratification			After stratification		
Characteristic	T (%)	C (%)	StdDiff	T (%)	C (%)	StdDiff
Age group						
25-29	0.1	0.1	-0.01	0.1	0.1	0.00
30-34	0.5	0.6	-0.02	0.5	0.5	0.00
35-39	1.2	1.4	-0.02	1.3	1.2	0.00
40-44	1.7	2.0	-0.02	1.8	1.8	0.00
45-49	3.2	3.5	-0.02	3.3	3.2	0.01
50-54	5.3	5.9	-0.02	5.4	5.4	0.00
55-59	7.6	8.6	-0.04	7.9	7.8	0.00
60-64	11.0	12.5	-0.05	11.5	11.4	0.00
65-69	14.9	15.1	0.00	14.9	14.9	0.00
70-74	28.2	24.8	0.08	26.9	27.1	0.00
75-79	12.4	11.0	0.04	12.0	12.0	0.00
80-84	6.3	6.1	0.01	6.3	6.4	0.00
85-89	4.9	5.1	-0.01	5.2	5.1	0.00
90-94	2.0	2.4	-0.02	2.2	2.2	0.00
95-99	0.6	0.8	-0.03	0.7	0.7	0.00
Gender: female	4.4	8.0	-0.15	5.6	5.9	-0.01
Race						
race = Asian	1.0	0.7	0.03	0.9	0.9	0.00
race = Black or African American	13.0	30.3	-0.43	19.3	19.8	-0.01
race = White	78.2	61.9	0.36	72.4	71.8	0.01
race = Unknown	6.1	5.6	0.02	5.9	6.0	0.00
race = American Indian or Alaska Native	0.8	0.6	0.02	0.7	0.7	0.00
Ethnicity						
ethnicity = Hispanic or Latino	6.3	4.8	0.07	5.7	5.6	0.01
ethnicity = Not Hispanic or Latino	91.2	92.9	-0.06	91.8	91.9	0.00
Medical history: General						
Acute respiratory disease	5.7	5.9	-0.01	5.7	5.7	0.00
Attention deficit hyperactivity disorder	0.5	0.5	0.00	0.5	0.5	0.00
Chronic liver disease	2.1	2.8	-0.05	2.3	2.4	0.00
Chronic obstructive lung disease	12.2	11.6	0.02	12.1	11.9	0.01
Crohn’s disease	0.2	0.2	-0.01	0.2	0.2	0.00
Diabetes mellitus	41.2	24.5	0.36	35.0	35.1	0.00
Gastroesophageal reflux disease	15.4	15.6	-0.01	15.5	15.6	0.00
Human immunodeficiency virus infection	0.5	0.6	-0.02	0.5	0.5	0.00
Hyperlipidemia	51.9	44.5	0.15	49.2	48.7	0.01
Hypertensive disorder	70.3	73.0	-0.06	71.4	72.0	-0.01
Lesion of liver	1.7	2.1	-0.03	1.8	1.8	0.00
Obesity	14.6	12.5	0.06	13.9	13.7	0.01

(Continued)	Before stratification			After stratification		
Characteristic	T (%)	C (%)	StdDiff	T (%)	C (%)	StdDiff
Osteoarthritis	15.2	16.3	-0.03	15.7	15.8	0.00
Pneumonia	1.4	1.5	-0.01	1.5	1.5	0.00
Psoriasis	1.2	1.0	0.02	1.2	1.1	0.00
Renal impairment	10.6	13.1	-0.08	11.8	12.2	-0.01
Rheumatoid arthritis	0.8	0.8	0.00	0.8	0.8	0.00
Schizophrenia	0.9	1.1	-0.03	1.0	1.0	0.00
Ulcerative colitis	0.3	0.3	0.00	0.3	0.3	0.00
Urinary tract infectious disease	1.8	2.1	-0.02	1.9	1.9	0.00
Viral hepatitis C	1.2	2.0	-0.06	1.4	1.5	-0.01
Visual system disorder	31.1	30.2	0.02	30.8	30.9	0.00
Medical history: Cardiovascular disease						
Atrial fibrillation	11.0	6.2	0.17	9.4	8.7	0.03
Cerebrovascular disease	3.4	3.4	0.00	3.5	3.5	0.00
Coronary arteriosclerosis	19.6	11.2	0.23	16.6	15.7	0.02
Heart disease	35.4	23.1	0.27	31.1	29.7	0.03
Heart failure	9.7	4.0	0.23	7.8	6.1	0.07
Ischemic heart disease	7.3	4.2	0.13	6.2	5.7	0.02
Pulmonary embolism	0.6	0.5	0.01	0.6	0.6	0.00
Venous thrombosis	1.1	1.2	-0.01	1.1	1.2	-0.01
Medical history: Neoplasms						
Hematologic neoplasm	1.2	1.3	-0.01	1.3	1.2	0.00
Malignant lymphoma	0.7	0.7	0.00	0.7	0.7	0.00
Malignant neoplastic disease	9.1	10.6	-0.05	9.7	9.8	0.00
Malignant tumor of breast	0.1	0.1	-0.01	0.1	0.1	0.00
Malignant tumor of colon	0.3	0.4	-0.01	0.4	0.4	0.00
Malignant tumor of lung	0.5	0.7	-0.02	0.6	0.6	0.00
Malignant tumor of urinary bladder	0.6	0.6	0.00	0.6	0.6	0.00
Primary malignant neoplasm of prostate	3.3	4.2	-0.05	3.6	3.7	0.00
Medication use						
Antibacterials for systemic use	19.2	20.1	-0.02	19.6	19.6	0.00
Antidepressants	33.1	33.3	0.00	33.3	33.2	0.00
Antiepileptics	25.6	24.3	0.03	25.2	25.4	0.00
Antiinflammatory and antirheumatic products	27.5	31.3	-0.09	28.9	29.0	0.00
Antineoplastic agents	2.8	3.1	-0.02	3.0	3.0	0.00
Antipsoriatics	1.0	1.6	-0.06	1.2	1.4	-0.02
Antithrombotic agents	40.1	30.9	0.19	36.8	36.1	0.01
Drugs for acid related disorders	40.8	39.6	0.02	40.5	40.8	-0.01
Drugs for obstructive airway diseases	31.5	31.7	0.00	31.8	31.6	0.00
Drugs used in diabetes	42.7	23.5	0.42	35.3	35.6	0.00
Immunosuppressants	2.9	3.4	-0.03	3.1	3.2	0.00
Lipid modifying agents	73.5	60.2	0.29	68.5	68.0	0.01
Psychostimulants, agents used for adhd and nootropics	1.2	1.3	-0.01	1.3	1.3	0.00

Supplementary Table 27: Baseline patient characteristcs for ACEI/ARB mono (T) and CCB mono (C) prevalent-users in the VA-OMOP data source. We report proportion of initiators satisfying selected base-line characteristics and the standardized difference of population proportions (StdDiff) before and after stratification. Less extreme StdDiffs through stratification suggest improved balance between patient cohorts through propensity score adjustment.

	Before stratification			After stratification		
Characteristic	T (%)	C (%)	StdDiff	T (%)	C (%)	StdDiff
Age group						
25-29	0.2	0.2	0.00	0.2	0.2	0.00
30-34	0.8	0.9	-0.01	0.8	0.8	0.00
35-39	1.9	1.9	0.00	1.9	1.8	0.01
40-44	2.7	2.4	0.01	2.6	2.5	0.01
45-49	4.6	4.0	0.03	4.5	4.2	0.02
50-54	7.2	6.4	0.03	7.0	6.6	0.02
55-59	9.5	9.5	0.00	9.4	9.2	0.01
60-64	12.0	13.5	-0.04	12.3	12.3	0.00
65-69	14.5	14.8	-0.01	14.5	14.6	0.00
70-74	25.2	22.3	0.07	24.3	24.8	-0.01
75-79	10.4	9.8	0.02	10.3	10.6	-0.01
80-84	5.2	5.6	-0.02	5.4	5.6	-0.01
85-89	3.9	5.0	-0.06	4.4	4.5	-0.01
90-94	1.5	2.5	-0.07	1.9	1.9	0.00
Gender: female	5.2	7.3	-0.09	5.7	5.7	0.00
Race						
race = Black or African American	12.9	33.1	-0.50	17.9	19.2	-0.03
race = White	77.5	58.7	0.41	72.8	71.8	0.02
race = Unknown	6.6	5.8	0.03	6.5	6.2	0.01
race = American Indian or Alaska Native	0.9	0.6	0.03	0.8	0.8	0.00
Ethnicity						
ethnicity = Hispanic or Latino	7.6	5.4	0.09	7.0	6.8	0.01
ethnicity = Not Hispanic or Latino	89.7	92.2	-0.09	90.4	90.6	-0.01
Medical history: General						
Acute respiratory disease	4.3	5.0	-0.03	4.5	4.5	0.00
Attention deficit hyperactivity disorder	0.7	0.6	0.02	0.7	0.6	0.01
Chronic liver disease	1.6	2.8	-0.08	1.8	2.0	-0.01
Chronic obstructive lung disease	7.7	9.9	-0.08	8.4	8.6	-0.01
Crohn’s disease	0.2	0.2	-0.01	0.2	0.2	0.00
Dementia	2.0	3.0	-0.06	2.3	2.5	-0.01
Depressive disorder	16.7	17.6	-0.02	16.9	17.1	0.00
Diabetes mellitus	37.8	16.0	0.51	31.6	31.1	0.01
Gastroesophageal reflux disease	13.9	14.2	-0.01	14.0	14.3	-0.01
Gastrointestinal hemorrhage	0.6	0.9	-0.03	0.7	0.8	-0.01
Human immunodeficiency virus infection	0.5	0.8	-0.03	0.6	0.6	0.00
Hyperlipidemia	48.2	39.1	0.18	45.6	45.1	0.01
Hypertensive disorder	68.4	71.0	-0.06	69.1	70.0	-0.02
Lesion of liver	1.2	1.8	-0.04	1.3	1.4	-0.01
Obesity	12.7	9.0	0.12	11.7	11.4	0.01

(Continued)	Before stratification			After stratification		
Characteristic	T (%)	C (%)	StdDiff	T (%)	C (%)	StdDiff
Osteoarthritis	14.5	15.2	-0.02	14.7	14.9	0.00
Pneumonia	0.7	1.1	-0.04	0.8	0.9	-0.01
Psoriasis	1.2	1.0	0.02	1.1	1.1	0.00
Renal impairment	6.1	8.5	-0.09	6.7	7.5	-0.03
Rheumatoid arthritis	0.7	0.8	0.00	0.7	0.7	0.00
Schizophrenia	0.8	1.3	-0.05	0.9	1.1	-0.01
Urinary tract infectious disease	1.3	1.8	-0.04	1.4	1.5	-0.01
Viral hepatitis C	1.0	2.2	-0.09	1.3	1.5	-0.02
Visual system disorder	28.5	27.8	0.02	28.3	28.3	0.00
Medical history: Cardiovascular disease						
Atrial fibrillation	2.8	2.5	0.02	2.8	3.0	-0.01
Cerebrovascular disease	2.3	2.6	-0.02	2.4	2.6	-0.01
Coronary arteriosclerosis	6.4	5.1	0.06	6.1	6.5	-0.02
Heart disease	14.0	12.9	0.03	13.8	14.6	-0.02
Heart failure	1.1	0.9	0.02	1.0	1.0	0.00
Ischemic heart disease	1.9	1.7	0.01	1.8	2.0	-0.01
Peripheral vascular disease	2.7	2.8	-0.01	2.7	2.9	-0.01
Venous thrombosis	0.8	1.0	-0.03	0.8	0.9	-0.01
Medical history: Neoplasms						
Hematologic neoplasm	1.0	1.3	-0.03	1.1	1.1	0.00
Malignant neoplasm of anorectum	0.1	0.1	-0.01	0.1	0.1	0.00
Malignant neoplastic disease	7.9	10.5	-0.09	8.6	9.0	-0.01
Malignant tumor of breast	0.1	0.1	-0.01	0.1	0.1	0.00
Malignant tumor of colon	0.3	0.4	-0.02	0.3	0.3	-0.01
Malignant tumor of lung	0.3	0.6	-0.04	0.4	0.4	0.00
Primary malignant neoplasm of prostate	3.0	4.3	-0.07	3.4	3.5	-0.01
Medication use						
Antibacterials for systemic use	15.6	17.9	-0.06	16.2	16.4	-0.01
Antidepressants	31.8	31.3	0.01	31.7	31.9	0.00
Antiepileptics	22.8	21.0	0.04	22.3	22.5	-0.01
Antineoplastic agents	2.5	2.9	-0.02	2.6	2.6	0.00
Antipsoriatics	0.6	0.9	-0.03	0.7	0.7	-0.01
Antithrombotic agents	23.4	21.6	0.04	22.9	23.3	-0.01
Drugs for acid related disorders	34.2	33.9	0.01	34.1	34.7	-0.01
Drugs for obstructive airway diseases	26.1	28.3	-0.05	26.8	27.0	0.00
Drugs used in diabetes	39.4	14.2	0.59	32.1	31.4	0.02
Immunosuppressants	2.6	3.1	-0.03	2.8	2.9	-0.01
Lipid modifying agents	64.8	49.6	0.31	60.4	59.6	0.02
Opioids	9.2	9.8	-0.02	9.3	9.6	-0.01
Psycholeptics	19.0	21.0	-0.05	19.5	20.1	-0.01
Psychostimulants, agents used for adhd and nootropics	1.4	1.3	0.02	1.4	1.4	0.00

Supplementary Table 28: Baseline patient characteristcs for ACEI/ARB +combo (T) and CCB +combo (C) prevalent-users in the VA-OMOP data source. We report proportion of initiators satisfying selected base-line characteristics and the standardized difference of population proportions (StdDiff) before and after stratification. Less extreme StdDiffs through stratification suggest improved balance between patient cohorts through propensity score adjustment.

	Before stratification			After stratification		
Characteristic	T (%)	C (%)	StdDiff	T (%)	C (%)	StdDiff
Age group						
25-29	0.1	0.1	-0.01	0.1	0.1	0.00
30-34	0.5	0.6	-0.01	0.5	0.5	0.01
35-39	1.2	1.3	-0.01	1.2	1.1	0.01
40-44	1.8	1.8	0.00	1.8	1.7	0.01
45-49	3.4	3.2	0.01	3.3	3.2	0.01
50-54	5.7	5.5	0.01	5.6	5.5	0.01
55-59	8.1	8.3	-0.01	8.2	8.0	0.01
60-64	11.5	12.6	-0.04	11.7	11.7	0.00
70-74	28.2	24.8	0.08	27.3	27.7	-0.01
75-79	12.0	11.1	0.03	11.8	11.9	0.00
80-84	5.9	6.3	-0.02	6.0	6.1	0.00
85-89	4.4	5.5	-0.05	4.8	4.7	0.00
90-94	1.7	2.6	-0.06	2.0	2.0	0.00
95-99	0.5	0.9	-0.05	0.6	0.6	0.00
Gender: female	4.9	6.6	-0.07	5.4	5.3	0.00
Race						
race = Asian	0.9	0.8	0.01	0.9	0.9	-0.01
race = Black or African American	15.3	32.1	-0.40	19.0	19.6	-0.01
race = White	76.2	60.2	0.35	72.6	72.0	0.01
race = Unknown	6.1	5.6	0.02	6.0	5.9	0.00
race = Native Hawaiian or Other Pacific Islander	0.9	0.8	0.01	0.8	0.9	0.00
race = American Indian or Alaska Native	0.7	0.6	0.02	0.7	0.7	0.00
Ethnicity						
ethnicity = Hispanic or Latino	6.0	4.9	0.05	5.7	5.8	0.00
ethnicity = Not Hispanic or Latino	91.5	92.7	-0.05	91.8	91.7	0.00
Medical history: General						
Attention deficit hyperactivity disorder	0.5	0.4	0.01	0.5	0.5	0.00
Chronic liver disease	2.0	3.1	-0.07	2.2	2.3	-0.01
Chronic obstructive lung disease	11.2	12.1	-0.03	11.4	11.3	0.00
Crohn's disease	0.2	0.2	-0.01	0.2	0.2	0.00
Dementia	2.1	3.1	-0.06	2.3	2.5	-0.01
Depressive disorder	16.4	18.0	-0.04	16.8	17.0	-0.01
Diabetes mellitus	40.0	25.3	0.32	36.4	37.2	-0.02
Gastroesophageal reflux disease	15.1	15.7	-0.02	15.2	15.5	-0.01
Gastrointestinal hemorrhage	0.9	1.2	-0.03	0.9	1.0	0.00
Human immunodeficiency virus infection	0.5	0.7	-0.03	0.5	0.5	0.00
Hyperlipidemia	51.2	44.8	0.13	49.6	49.7	0.00
Hypertensive disorder	71.2	74.1	-0.07	71.8	72.7	-0.02
Lesion of liver	1.6	2.3	-0.05	1.7	1.8	0.00

(Continued)	Before stratification			After stratification		
Characteristic	T (%)	C (%)	StdDiff	T (%)	C (%)	StdDiff
Obesity	15.2	11.6	0.11	14.4	14.5	0.00
Osteoarthritis	15.5	16.1	-0.01	15.7	15.9	-0.01
Pneumonia	1.3	1.7	-0.04	1.3	1.4	-0.01
Psoriasis	1.2	1.0	0.02	1.2	1.1	0.00
Renal impairment	9.9	15.3	-0.16	10.8	12.0	-0.04
Rheumatoid arthritis	0.8	0.8	0.00	0.8	0.8	0.00
Schizophrenia	0.8	1.3	-0.05	0.9	1.0	-0.01
Ulcerative colitis	0.3	0.3	0.00	0.3	0.3	0.00
Urinary tract infectious disease	1.6	2.3	-0.05	1.7	1.9	-0.01
Viral hepatitis C	1.2	2.3	-0.08	1.4	1.5	-0.01
Visual system disorder	30.8	30.8	0.00	30.6	31.1	-0.01
Medical history: Cardiovascular disease						
Atrial fibrillation	9.5	6.1	0.12	8.8	8.3	0.02
Cerebrovascular disease	3.2	3.8	-0.04	3.3	3.4	-0.01
Coronary arteriosclerosis	16.8	12.2	0.13	15.8	15.4	0.01
Heart disease	31.2	24.5	0.15	29.6	29.3	0.01
Heart failure	7.7	4.4	0.14	6.9	5.9	0.04
Ischemic heart disease	6.2	4.7	0.07	5.8	5.7	0.00
Peripheral vascular disease	4.6	4.9	-0.01	4.6	4.8	-0.01
Pulmonary embolism	0.5	0.5	0.00	0.5	0.5	0.00
Venous thrombosis	1.0	1.3	-0.02	1.1	1.1	-0.01
Medical history: Neoplasms						
Hematologic neoplasm	1.1	1.4	-0.03	1.2	1.2	0.00
Malignant lymphoma	0.7	0.7	-0.01	0.7	0.7	0.00
Malignant neoplasm of anorectum	0.1	0.1	-0.01	0.1	0.1	0.00
Malignant neoplastic disease	8.8	11.3	-0.08	9.3	9.6	-0.01
Malignant tumor of breast	0.1	0.1	-0.01	0.1	0.1	0.00
Malignant tumor of colon	0.3	0.4	-0.02	0.3	0.3	0.00
Malignant tumor of lung	0.5	0.7	-0.04	0.5	0.5	0.00
Malignant tumor of urinary bladder	0.6	0.7	-0.02	0.6	0.6	0.00
Primary malignant neoplasm of prostate	3.3	4.4	-0.06	3.5	3.7	-0.01
Medication use						
Antibacterials for systemic use	18.6	20.7	-0.05	18.9	19.3	-0.01
Antidepressants	32.5	33.3	-0.01	32.8	33.1	-0.01
Antiepileptics	25.0	24.4	0.01	24.9	25.2	-0.01
Antiinflammatory and antirheumatic products	28.8	30.4	-0.03	29.3	29.4	0.00
Antineoplastic agents	2.8	3.2	-0.02	2.9	2.9	0.00
Antipsoriatics	0.9	1.9	-0.09	1.0	1.4	-0.03
Antithrombotic agents	36.9	32.9	0.08	35.8	36.0	0.00
Drugs for acid related disorders	40.0	40.1	0.00	39.9	40.6	-0.01
Drugs for obstructive airway diseases	30.7	31.7	-0.02	31.0	31.1	0.00
Drugs used in diabetes	41.6	24.1	0.38	37.2	38.0	-0.02
Immunosuppressants	2.8	3.6	-0.04	2.9	3.1	-0.01
Lipid modifying agents	72.4	60.7	0.25	69.5	69.5	0.00
Opioids	11.0	12.1	-0.03	11.2	11.6	-0.01

(Continued)	Before stratification			After stratification		
Characteristic	T (%)	C (%)	StdDiff	T (%)	C (%)	StdDiff
Psycholeptics	19.9	22.5	-0.06	20.4	20.9	-0.01
Psychostimulants, agents used for adhd and nootropics	1.2	1.2	0.00	1.2	1.2	0.00

Supplementary Table 29: Baseline patient characteristcs for ACEI/ARB mono (T) and THZ mono (C) prevalent-users in the VA-OMOP data source. We report proportion of initiators satisfying selected base-line characteristics and the standardized difference of population proportions (StdDiff) before and after stratification. Less extreme StdDiffs through stratification suggest improved balance between patient cohorts through propensity score adjustment.

	Before stratification			After stratification		
Characteristic	T (%)	C (%)	StdDiff	T (%)	C (%)	StdDiff
Age group						
25-29	0.2	0.3	-0.02	0.2	0.2	0.01
30-34	0.8	1.2	-0.04	0.9	0.8	0.01
35-39	1.9	2.6	-0.05	2.0	1.9	0.01
40-44	2.7	3.5	-0.05	2.8	2.7	0.00
45-49	4.6	5.4	-0.03	4.7	4.6	0.01
50-54	7.2	8.3	-0.04	7.4	7.0	0.01
55-59	9.5	10.9	-0.05	9.6	9.5	0.00
60-64	12.0	13.1	-0.03	12.2	11.8	0.01
65-69	14.5	14.3	0.00	14.5	14.6	0.00
70-74	25.2	22.0	0.08	24.8	24.6	0.00
75-79	10.4	9.0	0.05	10.2	10.7	-0.02
80-84	5.2	4.4	0.04	5.0	5.4	-0.01
85-89	3.9	3.2	0.04	3.8	4.1	-0.02
90-94	1.5	1.3	0.01	1.5	1.7	-0.02
95-99	0.4	0.4	0.00	0.4	0.5	-0.01
Gender: female	5.2	12.9	-0.27	6.1	6.6	-0.02
Race						
race = Asian	1.2	0.7	0.06	1.2	1.0	0.01
race = Black or African American	12.9	28.8	-0.40	14.9	15.5	-0.02
race = White	77.5	63.3	0.31	75.7	75.6	0.00
race = Unknown	6.6	5.8	0.03	6.5	6.2	0.01
race = Native Hawaiian or Other Pacific Islander	1.0	0.8	0.02	1.0	1.0	0.00
race = American Indian or Alaska Native	0.9	0.6	0.04	0.8	0.7	0.01
Ethnicity						
ethnicity = Hispanic or Latino	7.6	5.1	0.10	7.3	7.1	0.01
ethnicity = Not Hispanic or Latino	89.7	92.5	-0.10	90.1	90.4	-0.01
Medical history: General						
Acute respiratory disease	4.3	4.9	-0.03	4.4	4.5	-0.01
Attention deficit hyperactivity disorder	0.7	0.7	0.00	0.7	0.8	0.00
Chronic liver disease	1.6	1.9	-0.02	1.6	1.7	-0.01
Chronic obstructive lung disease	7.7	8.0	-0.01	7.7	8.2	-0.02
Crohn’s disease	0.2	0.2	0.00	0.2	0.2	0.01
Diabetes mellitus	37.8	15.5	0.52	34.4	34.2	0.00
Gastroesophageal reflux disease	13.9	14.0	0.00	13.9	14.4	-0.01
Gastrointestinal hemorrhage	0.6	0.6	0.00	0.6	0.7	0.00
Human immunodeficiency virus infection	0.5	0.7	-0.03	0.5	0.6	0.00
Hyperlipidemia	48.2	39.3	0.18	46.9	46.0	0.02
Hypertensive disorder	68.4	68.2	0.00	68.4	69.1	-0.02
Obesity	12.7	13.0	-0.01	12.7	12.8	0.00

(Continued)	Before stratification			After stratification		
Characteristic	T (%)	C (%)	StdDiff	T (%)	C (%)	StdDiff
Osteoarthritis	14.5	16.1	-0.05	14.7	15.2	-0.01
Pneumonia	0.7	0.6	0.01	0.7	0.7	-0.01
Psoriasis	1.2	1.0	0.01	1.2	1.1	0.01
Renal impairment	6.1	4.1	0.09	5.8	6.4	-0.02
Rheumatoid arthritis	0.7	0.8	-0.01	0.7	0.7	0.00
Schizophrenia	0.8	0.9	-0.01	0.8	0.9	-0.01
Ulcerative colitis	0.3	0.3	0.00	0.3	0.4	-0.01
Urinary tract infectious disease	1.3	1.1	0.01	1.2	1.3	-0.01
Viral hepatitis C	1.0	1.4	-0.03	1.1	1.2	-0.01
Visual system disorder	28.5	26.7	0.04	28.2	28.1	0.00
Medical history: Cardiovascular disease						
Atrial fibrillation	2.8	1.9	0.06	2.7	3.1	-0.03
Cerebrovascular disease	2.3	1.5	0.06	2.2	2.3	0.00
Coronary arteriosclerosis	6.4	3.2	0.15	5.9	6.4	-0.02
Heart disease	14.0	9.6	0.14	13.3	14.1	-0.03
Heart failure	1.1	0.6	0.05	1.0	1.0	0.00
Ischemic heart disease	1.9	1.0	0.07	1.7	1.8	-0.01
Peripheral vascular disease	2.7	1.9	0.05	2.5	2.6	-0.01
Pulmonary embolism	0.4	0.3	0.01	0.4	0.4	0.00
Medical history: Neoplasms						
Hematologic neoplasm	1.0	1.0	0.00	1.0	1.1	-0.01
Malignant lymphoma	0.6	0.6	0.01	0.6	0.6	0.00
Malignant neoplasm of anorectum	0.1	0.1	0.01	0.1	0.1	0.01
Malignant neoplastic disease	7.9	7.9	0.00	7.9	8.1	-0.01
Malignant tumor of breast	0.1	0.2	-0.02	0.1	0.1	0.00
Malignant tumor of colon	0.3	0.2	0.01	0.3	0.2	0.00
Malignant tumor of lung	0.3	0.4	-0.01	0.3	0.4	-0.01
Malignant tumor of urinary bladder	0.5	0.4	0.02	0.4	0.4	0.00
Primary malignant neoplasm of prostate	3.0	3.3	-0.02	3.1	3.1	0.00
Medication use						
Antibacterials for systemic use	15.6	16.3	-0.02	15.7	16.0	-0.01
Antidepressants	31.8	31.7	0.00	31.7	32.1	-0.01
Antiepileptics	22.8	20.9	0.04	22.4	23.2	-0.02
Antiinflammatory and antirheumatic products	29.8	35.0	-0.11	30.5	30.8	0.00
Antineoplastic agents	2.5	2.9	-0.03	2.6	2.7	-0.01
Antipsoriatics	0.6	0.6	0.00	0.6	0.7	-0.01
Antithrombotic agents	23.4	16.3	0.18	22.3	22.2	0.00
Drugs for acid related disorders	34.2	32.8	0.03	33.9	34.9	-0.02
Drugs for obstructive airway diseases	26.1	28.7	-0.06	26.4	27.3	-0.02
Drugs used in diabetes	39.4	14.2	0.59	35.6	34.8	0.02
Immunosuppressants	2.6	2.7	0.00	2.6	2.7	0.00
Lipid modifying agents	64.8	49.2	0.32	62.4	60.7	0.03
Opioids	9.2	9.4	-0.01	9.2	9.6	-0.01
Psycholeptics	19.0	19.5	-0.01	19.0	19.4	-0.01

(Continued)

Characteristic	Before stratification			After stratification		
	T (%)	C (%)	StdDiff	T (%)	C (%)	StdDiff
Psychostimulants, agents used for adhd and nootropics	1.4	1.7	-0.02	1.5	1.6	-0.01

Supplementary Table 30: Baseline patient characteristcs for ACEI/ARB +combo (T) and THZ +combo (C) prevalent-users in the VA-OMOP data source. We report proportion of initiators satisfying selected base-line characteristics and the standardized difference of population proportions (StdDiff) before and after stratification. Less extreme StdDiffs through stratification suggest improved balance between patient cohorts through propensity score adjustment.

	Before stratification			After stratification		
Characteristic	T (%)	C (%)	StdDiff	T (%)	C (%)	StdDiff
Age group						
25-29	0.1	0.1	-0.01	0.1	0.1	0.01
30-34	0.4	0.7	-0.04	0.4	0.5	0.00
35-39	1.0	1.6	-0.05	1.1	1.1	0.00
45-49	2.9	4.0	-0.06	3.1	3.1	0.00
50-54	5.0	6.7	-0.07	5.3	5.2	0.00
60-64	11.0	12.8	-0.05	11.3	11.2	0.00
65-69	15.2	15.1	0.00	15.2	15.0	0.01
75-79	12.6	10.5	0.07	12.3	12.5	0.00
80-84	6.5	5.4	0.05	6.3	6.4	0.00
85-89	5.0	4.1	0.04	4.9	4.9	0.00
90-94	2.0	1.7	0.03	2.0	2.1	-0.01
95-99	0.6	0.5	0.01	0.6	0.6	0.00
Gender: female	4.3	10.5	-0.24	5.1	5.6	-0.02
Race						
race = Asian	1.0	0.5	0.05	0.9	0.9	0.01
race = Black or African American	16.1	30.2	-0.34	18.2	18.8	-0.01
race = White	75.2	62.4	0.28	73.4	72.8	0.01
race = Unknown	6.0	5.6	0.02	5.9	6.0	0.00
race = Native Hawaiian or Other Pacific Islander	0.9	0.7	0.02	0.9	0.9	0.00
race = American Indian or Alaska Native	0.7	0.5	0.02	0.7	0.7	0.00
Ethnicity						
ethnicity = Hispanic or Latino	6.2	4.3	0.08	5.8	5.8	0.00
ethnicity = Not Hispanic or Latino	91.3	93.4	-0.08	91.7	91.7	0.00
Medical history: General						
Acute respiratory disease	5.7	5.6	0.01	5.7	5.6	0.00
Attention deficit hyperactivity disorder	0.5	0.6	-0.01	0.5	0.5	0.00
Chronic liver disease	2.2	2.3	0.00	2.3	2.3	-0.01
Chronic obstructive lung disease	12.1	10.4	0.06	11.9	11.7	0.01
Crohn's disease	0.2	0.2	0.00	0.2	0.2	0.00
Dementia	2.6	1.6	0.07	2.5	2.4	0.00
Depressive disorder	16.8	17.7	-0.02	16.9	16.9	0.00
Diabetes mellitus	41.8	24.1	0.38	39.0	39.7	-0.02
Gastroesophageal reflux disease	15.5	15.2	0.01	15.5	15.5	0.00
Gastrointestinal hemorrhage	1.0	0.9	0.01	1.0	0.9	0.01
Human immunodeficiency virus infection	0.5	0.6	-0.02	0.5	0.5	0.00
Hyperlipidemia	52.0	44.4	0.15	50.8	50.4	0.01
Hypertensive disorder	72.1	72.3	0.00	72.1	73.0	-0.02
Lesion of liver	1.8	1.7	0.01	1.8	1.8	0.00
Obesity	14.7	14.6	0.00	14.7	14.9	-0.01

(Continued)	Before stratification			After stratification		
Characteristic	T (%)	C (%)	StdDiff	T (%)	C (%)	StdDiff
Osteoarthritis	15.5	17.0	-0.04	15.8	15.9	0.00
Pneumonia	1.5	1.1	0.03	1.4	1.4	0.00
Psoriasis	1.2	1.1	0.01	1.2	1.1	0.00
Renal impairment	12.3	9.0	0.11	11.9	12.7	-0.02
Rheumatoid arthritis	0.8	0.8	0.00	0.8	0.8	0.00
Schizophrenia	0.9	0.9	0.00	0.9	0.9	0.00
Ulcerative colitis	0.3	0.3	0.00	0.3	0.3	0.00
Urinary tract infectious disease	1.9	1.5	0.03	1.8	1.8	0.00
Viral hepatitis C	1.4	1.5	-0.01	1.4	1.4	0.00
Visual system disorder	32.0	29.6	0.05	31.6	31.6	0.00
Medical history: Cardiovascular disease						
Atrial fibrillation	10.4	5.8	0.17	9.7	9.1	0.02
Cerebrovascular disease	3.8	2.6	0.07	3.6	3.7	0.00
Coronary arteriosclerosis	19.2	8.8	0.30	17.6	16.4	0.03
Heart disease	34.9	19.8	0.34	32.5	31.2	0.03
Heart failure	9.1	3.1	0.25	8.2	6.5	0.06
Ischemic heart disease	7.2	3.2	0.18	6.6	5.9	0.03
Peripheral vascular disease	5.4	3.5	0.09	5.1	5.1	0.00
Pulmonary embolism	0.6	0.5	0.01	0.5	0.5	0.00
Venous thrombosis	1.2	0.9	0.02	1.1	1.1	0.00
Medical history: Neoplasms						
Hematologic neoplasm	1.2	1.1	0.01	1.2	1.2	0.00
Malignant lymphoma	0.7	0.6	0.01	0.7	0.7	0.00
Malignant neoplasm of anorectum	0.1	0.1	0.00	0.1	0.1	0.00
Malignant neoplastic disease	9.5	9.3	0.01	9.5	9.6	0.00
Malignant tumor of breast	0.1	0.2	-0.02	0.1	0.1	0.00
Malignant tumor of colon	0.3	0.3	0.01	0.3	0.3	0.00
Malignant tumor of lung	0.5	0.5	0.01	0.5	0.5	0.00
Malignant tumor of urinary bladder	0.6	0.5	0.02	0.6	0.6	0.00
Primary malignant neoplasm of prostate	3.6	3.9	-0.02	3.6	3.6	0.00
Medication use						
Antibacterials for systemic use	19.6	18.9	0.02	19.5	19.6	0.00
Antidepressants	33.1	33.3	0.00	33.1	33.2	0.00
Antiepileptics	25.7	24.1	0.04	25.5	25.9	-0.01
Antiinflammatory and antirheumatic products	27.8	34.0	-0.13	28.7	29.1	-0.01
Antipsoriatics	1.2	1.0	0.01	1.2	1.3	-0.01
Antithrombotic agents	40.7	27.3	0.28	38.6	37.8	0.02
Drugs for acid related disorders	41.6	38.7	0.06	41.1	41.4	0.00
Drugs for obstructive airway diseases	31.7	31.8	0.00	31.7	31.8	0.00
Drugs used in diabetes	43.1	23.6	0.42	39.9	40.9	-0.02
Immunosuppressants	3.0	2.9	0.00	3.0	3.0	0.00
Lipid modifying agents	74.0	60.2	0.30	71.7	71.1	0.01
Opioids	11.6	11.2	0.01	11.6	11.6	0.00
Psycholeptics	20.7	21.0	-0.01	20.7	20.7	0.00

(Continued)	Before stratification			After stratification		
Characteristic	T (%)	C (%)	StdDiff	T (%)	C (%)	StdDiff
Psychostimulants, agents used for adhd and nootropics	1.2	1.5	-0.03	1.2	1.3	-0.01

Supplementary Table 31: Baseline patient characteristcs for ACEI mono (T) and ARB mono (C) prevalent-users in the VA-OMOP data source. We report proportion of initiators satisfying selected base-line characteristics and the standardized difference of population proportions (StdDiff) before and after stratification. Less extreme StdDiffs through stratification suggest improved balance between patient cohorts through propensity score adjustment.

	Before stratification			After stratification		
Characteristic	T (%)	C (%)	StdDiff	T (%)	C (%)	StdDiff
Age group						
25-29	0.2	0.1	0.02	0.2	0.2	0.01
30-34	0.9	0.6	0.04	0.8	0.8	0.00
45-49	4.8	4.1	0.03	4.7	4.7	0.00
50-54	7.4	6.7	0.03	7.3	7.3	0.00
55-59	9.7	8.7	0.04	9.5	9.4	0.00
60-64	12.4	10.9	0.05	12.0	12.0	0.00
65-69	14.8	13.6	0.03	14.5	14.4	0.00
70-74	25.1	25.7	-0.01	25.2	25.3	0.00
75-79	9.9	11.7	-0.06	10.4	10.3	0.00
80-84	4.7	6.4	-0.08	5.1	5.1	0.00
85-89	3.5	5.1	-0.08	3.9	3.8	0.00
90-94	1.3	2.0	-0.05	1.5	1.5	0.00
95-99	0.4	0.5	-0.02	0.4	0.4	0.00
Race						
race = Asian	1.0	1.9	-0.08	1.2	1.3	-0.01
race = Black or African American	12.6	13.7	-0.03	13.0	12.9	0.00
race = White	78.0	75.9	0.05	77.5	77.3	0.00
race = Unknown	6.5	6.7	-0.01	6.5	6.6	0.00
race = Native Hawaiian or Other Pacific Islander	1.0	1.1	-0.01	1.0	1.0	0.00
race = American Indian or Alaska Native	0.9	0.7	0.02	0.9	0.8	0.00
Ethnicity						
ethnicity = Hispanic or Latino	7.8	7.3	0.02	7.7	7.7	0.00
ethnicity = Not Hispanic or Latino	89.6	90.0	-0.01	89.7	89.6	0.00
Medical history: General						
Attention deficit hyperactivity disorder	0.8	0.7	0.01	0.7	0.7	0.00
Chronic liver disease	1.7	1.3	0.03	1.6	1.5	0.01
Crohn’s disease	0.2	0.2	0.00	0.2	0.2	0.00
Dementia	2.0	1.9	0.01	2.0	2.0	0.00
Depressive disorder	16.9	15.9	0.03	16.7	16.6	0.00
Diabetes mellitus	38.0	37.0	0.02	37.7	37.8	0.00
Gastroesophageal reflux disease	13.6	14.8	-0.04	13.9	13.8	0.00
Gastrointestinal hemorrhage	0.7	0.6	0.01	0.7	0.6	0.00
Human immunodeficiency virus infection	0.6	0.4	0.02	0.5	0.5	0.00
Hyperlipidemia	48.3	47.7	0.01	48.1	48.2	0.00
Hypertensive disorder	68.0	68.9	-0.02	68.3	68.3	0.00
Lesion of liver	1.3	1.1	0.01	1.2	1.2	0.00
Obesity	12.4	13.2	-0.02	12.6	12.7	0.00
Osteoarthritis	14.2	15.1	-0.03	14.4	14.5	0.00
Pneumonia	0.7	0.7	-0.01	0.7	0.7	0.00

(Continued)	Before stratification			After stratification		
Characteristic	T (%)	C (%)	StdDiff	T (%)	C (%)	StdDiff
Psoriasis	1.2	1.1	0.01	1.2	1.2	0.00
Renal impairment	5.7	7.2	-0.06	6.1	6.1	0.00
Rheumatoid arthritis	0.7	0.8	-0.02	0.7	0.7	0.00
Schizophrenia	0.9	0.5	0.05	0.9	0.7	0.01
Urinary tract infectious disease	1.3	1.2	0.00	1.3	1.2	0.00
Viral hepatitis C	1.1	0.8	0.04	1.1	1.0	0.01
Visual system disorder	28.3	29.0	-0.02	28.5	28.6	0.00
Medical history: Cardiovascular disease						
Atrial fibrillation	2.4	3.9	-0.08	2.7	3.0	-0.02
Cerebrovascular disease	2.3	2.3	0.00	2.3	2.3	0.00
Coronary arteriosclerosis	5.9	7.9	-0.08	6.3	6.5	-0.01
Heart disease	13.0	16.5	-0.10	13.7	14.1	-0.01
Heart failure	0.8	1.8	-0.09	0.9	1.3	-0.05
Ischemic heart disease	1.7	2.2	-0.04	1.8	1.9	-0.01
Peripheral vascular disease	2.7	2.5	0.01	2.7	2.6	0.00
Venous thrombosis	0.7	0.8	0.00	0.8	0.8	0.00
Medical history: Neoplasms						
Hematologic neoplasm	1.0	1.1	-0.01	1.0	1.0	0.00
Malignant lymphoma	0.6	0.7	-0.01	0.6	0.6	0.00
Malignant neoplasm of anorectum	0.1	0.1	0.01	0.1	0.1	0.00
Malignant neoplastic disease	7.8	8.2	-0.01	7.9	8.0	0.00
Malignant tumor of breast	0.1	0.1	-0.01	0.1	0.1	-0.01
Malignant tumor of colon	0.3	0.3	0.00	0.3	0.3	0.00
Malignant tumor of lung	0.3	0.3	0.00	0.3	0.3	0.00
Malignant tumor of urinary bladder	0.5	0.5	0.00	0.5	0.5	0.00
Primary malignant neoplasm of prostate	3.0	3.1	-0.01	3.0	3.0	0.00
Medication use						
Antibacterials for systemic use	15.6	15.5	0.00	15.6	15.5	0.00
Antidepressants	32.2	30.8	0.03	31.9	31.8	0.00
Antiepileptics	23.2	21.6	0.04	22.8	22.7	0.00
Antiinflammatory and antirheumatic products	30.2	28.6	0.04	29.9	29.9	0.00
Antineoplastic agents	2.4	2.8	-0.02	2.5	2.5	0.00
Antithrombotic agents	23.4	23.5	0.00	23.3	23.5	0.00
Drugs for acid related disorders	33.6	35.5	-0.04	34.2	33.9	0.01
Drugs for obstructive airway diseases	25.1	28.6	-0.08	26.0	25.9	0.00
Drugs used in diabetes	40.1	37.6	0.05	39.4	39.6	0.00
Immunosuppressants	2.5	3.0	-0.03	2.6	2.7	0.00
Lipid modifying agents	65.4	63.0	0.05	64.8	64.9	0.00
Opioids	9.5	8.2	0.05	9.2	9.1	0.00
Psycholeptics	19.3	17.8	0.04	19.0	18.8	0.00
Psychostimulants, agents used for adhd and nootropics	1.5	1.4	0.00	1.4	1.5	-0.01

Supplementary Table 32: Baseline patient characteristcs for ACEI +combo (T) and ARB +combo (C) prevalent-users in the VA-OMOP data source. We report proportion of initiators satisfying selected base-line characteristics and the standardized difference of population proportions (StdDiff) before and after stratification. Less extreme StdDiffs through stratification suggest improved balance between patient cohorts through propensity score adjustment.

	Before stratification			After stratification		
Characteristic	T (%)	C (%)	StdDiff	T (%)	C (%)	StdDiff
Age group						
25-29	0.1	0.1	0.01	0.1	0.1	0.01
30-34	0.4	0.3	0.03	0.4	0.4	0.00
35-39	1.1	0.8	0.03	1.0	1.0	0.00
40-44	1.7	1.4	0.03	1.6	1.6	0.00
50-54	5.6	5.1	0.02	5.5	5.4	0.00
55-59	8.1	7.5	0.03	7.9	7.9	0.00
60-64	11.9	10.6	0.04	11.5	11.5	0.00
65-69	15.8	14.7	0.03	15.5	15.3	0.00
70-74	28.5	29.0	-0.01	28.5	28.8	-0.01
75-79	11.7	13.2	-0.05	12.1	12.2	0.00
80-84	5.6	6.8	-0.05	6.0	5.9	0.00
85-89	4.1	5.1	-0.05	4.5	4.4	0.00
90-94	1.6	2.0	-0.03	1.8	1.7	0.00
95-99	0.4	0.5	-0.01	0.5	0.5	0.00
Race						
race = Asian	0.7	1.4	-0.07	0.9	1.0	-0.01
race = Black or African American	17.6	19.9	-0.06	18.3	18.4	0.00
race = Unknown	5.9	6.0	0.00	6.0	5.9	0.00
race = Native Hawaiian or Other Pacific Islander	0.8	0.9	-0.01	0.9	0.9	0.00
Ethnicity						
ethnicity = Not Hispanic or Latino	91.6	91.5	0.00	91.5	91.7	-0.01
Medical history: General						
Acute respiratory disease	5.1	6.2	-0.04	5.4	5.6	-0.01
Chronic liver disease	2.3	2.0	0.02	2.2	2.1	0.01
Chronic obstructive lung disease	10.7	11.8	-0.04	11.0	11.1	0.00
Crohn’s disease	0.2	0.2	0.00	0.2	0.2	0.00
Dementia	2.2	2.2	0.00	2.3	2.1	0.01
Diabetes mellitus	40.4	43.1	-0.05	41.2	41.4	0.00
Gastroesophageal reflux disease	14.6	16.4	-0.05	15.2	15.2	0.00
Gastrointestinal hemorrhage	0.9	1.0	0.00	0.9	0.9	0.00
Human immunodeficiency virus infection	0.5	0.4	0.01	0.5	0.5	0.00
Hyperlipidemia	51.2	52.2	-0.02	51.4	51.7	0.00
Hypertensive disorder	72.1	74.4	-0.05	72.9	72.8	0.00
Obesity	14.7	17.0	-0.06	15.4	15.5	0.00
Pneumonia	1.2	1.5	-0.02	1.3	1.3	0.00
Renal impairment	10.2	14.1	-0.12	11.3	11.6	-0.01
Rheumatoid arthritis	0.7	0.9	-0.01	0.8	0.8	0.00
Schizophrenia	0.9	0.6	0.04	0.9	0.8	0.01
Urinary tract infectious disease	1.7	1.8	-0.01	1.7	1.7	0.00

(Continued)	Before stratification			After stratification		
Characteristic	T (%)	C (%)	StdDiff	T (%)	C (%)	StdDiff
Viral hepatitis C	1.4	1.1	0.03	1.4	1.3	0.01
Visual system disorder	31.0	33.3	-0.05	31.7	31.8	0.00
Medical history: Cardiovascular disease						
Atrial fibrillation	8.3	10.5	-0.07	8.7	9.2	-0.02
Cerebrovascular disease	3.4	3.7	-0.02	3.5	3.5	0.00
Coronary arteriosclerosis	15.7	18.8	-0.08	16.3	17.0	-0.02
Heart disease	29.1	34.9	-0.12	30.4	31.6	-0.03
Heart failure	6.0	9.6	-0.13	6.5	7.9	-0.05
Ischemic heart disease	5.7	7.0	-0.05	6.0	6.3	-0.01
Peripheral vascular disease	4.9	5.1	-0.01	4.9	4.9	0.00
Pulmonary embolism	0.5	0.6	-0.01	0.5	0.5	0.00
Venous thrombosis	1.0	1.1	-0.01	1.1	1.1	0.00
Medical history: Neoplasms						
Hematologic neoplasm	1.1	1.2	-0.01	1.1	1.1	0.00
Malignant lymphoma	0.6	0.7	-0.01	0.7	0.7	0.00
Malignant neoplasm of anorectum	0.1	0.1	0.01	0.1	0.1	0.00
Malignant neoplastic disease	9.0	9.5	-0.02	9.2	9.3	0.00
Malignant tumor of breast	0.1	0.1	-0.01	0.1	0.1	0.00
Malignant tumor of colon	0.3	0.3	0.00	0.3	0.3	0.00
Malignant tumor of lung	0.5	0.5	0.00	0.5	0.4	0.00
Primary malignant neoplasm of prostate	3.5	3.8	-0.01	3.6	3.6	0.00
Medication use						
Antibacterials for systemic use	18.4	19.9	-0.04	18.8	19.0	0.00
Antiepileptics	25.2	25.4	0.00	25.3	25.2	0.00
Antiinflammatory and antirheumatic products	29.3	28.8	0.01	29.3	29.2	0.00
Antineoplastic agents	2.7	3.1	-0.02	2.8	2.9	0.00
Antipsoriatics	0.9	1.4	-0.05	1.0	1.1	-0.01
Antithrombotic agents	36.7	40.2	-0.07	37.4	38.2	-0.02
Drugs for obstructive airway diseases	29.1	34.5	-0.12	30.7	30.9	0.00
Drugs used in diabetes	42.1	44.2	-0.04	42.7	42.9	0.00
Immunosuppressants	2.6	3.4	-0.04	2.8	2.9	0.00
Lipid modifying agents	73.0	73.5	-0.01	73.0	73.4	-0.01
Opioids	11.4	11.0	0.01	11.3	11.3	0.00
Psycholeptics	20.1	19.9	0.00	20.1	20.0	0.00

Supplementary Table 33: Baseline patient characteristcs for ACEI mono (T) and CCB/THZ mono (C) prevalent-users in the VA-OMOP data source. We report proportion of initiators satisfying selected base-line characteristics and the standardized difference of population proportions (StdDiff) before and after stratification. Less extreme StdDiffs through stratification suggest improved balance between patient cohorts through propensity score adjustment.

	Before stratification			After stratification		
Characteristic	T (%)	C (%)	StdDiff	T (%)	C (%)	StdDiff
Age group						
30-34	0.9	0.9	0.00	0.9	0.9	0.00
35-39	2.0	2.0	0.00	2.0	2.0	0.00
45-49	4.8	4.4	0.02	4.6	4.5	0.01
50-54	7.4	7.1	0.01	7.2	7.0	0.01
55-59	9.7	10.0	-0.01	9.8	9.6	0.01
60-64	12.4	13.6	-0.03	12.9	12.7	0.00
70-74	25.1	22.2	0.07	23.6	23.9	-0.01
75-79	9.9	9.5	0.01	9.8	10.0	-0.01
80-84	4.7	5.1	-0.02	5.0	5.2	-0.01
85-89	3.5	4.4	-0.05	4.0	4.1	-0.01
90-94	1.3	2.1	-0.06	1.7	1.8	0.00
95-99	0.4	0.7	-0.04	0.6	0.6	0.00
Gender: female	4.7	9.0	-0.17	6.3	6.9	-0.02
Race						
race = Asian	1.0	0.8	0.01	0.9	0.9	0.00
race = Black or African American	12.6	33.5	-0.51	22.6	22.8	0.00
race = White	78.0	58.6	0.43	68.8	68.6	0.00
race = Unknown	6.5	5.7	0.03	6.1	6.1	0.00
race = American Indian or Alaska Native	0.9	0.6	0.04	0.7	0.7	0.00
Ethnicity						
ethnicity = Hispanic or Latino	7.8	5.2	0.10	6.6	6.4	0.01
ethnicity = Not Hispanic or Latino	89.6	92.4	-0.10	90.9	91.1	-0.01
Medical history: General						
Acute respiratory disease	4.2	5.0	-0.04	4.6	4.7	0.00
Attention deficit hyperactivity disorder	0.8	0.6	0.02	0.7	0.7	0.00
Chronic liver disease	1.7	2.5	-0.06	2.1	2.1	0.00
Chronic obstructive lung disease	7.6	9.3	-0.06	8.4	8.7	-0.01
Crohn's disease	0.2	0.2	-0.01	0.2	0.2	0.00
Dementia	2.0	2.4	-0.02	2.3	2.3	0.00
Depressive disorder	16.9	17.7	-0.02	17.3	17.3	0.00
Diabetes mellitus	38.0	16.3	0.50	28.0	26.6	0.03
Gastroesophageal reflux disease	13.6	14.2	-0.02	13.9	14.1	-0.01
Gastrointestinal hemorrhage	0.7	0.8	-0.02	0.7	0.8	0.00
Human immunodeficiency virus infection	0.6	0.8	-0.02	0.7	0.7	0.00
Hyperlipidemia	48.3	39.6	0.17	44.0	43.5	0.01
Hypertensive disorder	68.0	71.1	-0.07	69.5	69.9	-0.01
Lesion of liver	1.3	1.6	-0.03	1.4	1.5	0.00
Obesity	12.4	10.6	0.06	11.5	11.3	0.01
Osteoarthritis	14.2	15.8	-0.04	15.0	15.1	0.00

(Continued)	Before stratification			After stratification		
Characteristic	T (%)	C (%)	StdDiff	T (%)	C (%)	StdDiff
Pneumonia	0.7	0.9	-0.03	0.8	0.8	-0.01
Psoriasis	1.2	1.0	0.02	1.1	1.1	0.00
Renal impairment	5.7	7.1	-0.06	6.5	6.8	-0.01
Rheumatoid arthritis	0.7	0.8	-0.01	0.7	0.7	0.00
Schizophrenia	0.9	1.2	-0.02	1.1	1.1	0.00
Urinary tract infectious disease	1.3	1.5	-0.02	1.4	1.5	0.00
Viral hepatitis C	1.1	2.0	-0.07	1.5	1.6	-0.01
Visual system disorder	28.3	27.7	0.01	28.0	28.0	0.00
Medical history: Cardiovascular disease						
Atrial fibrillation	2.4	2.3	0.01	2.4	2.5	-0.01
Cerebrovascular disease	2.3	2.3	0.00	2.4	2.4	0.00
Coronary arteriosclerosis	5.9	4.4	0.07	5.4	5.4	0.00
Heart disease	13.0	11.9	0.04	12.6	13.0	-0.01
Heart failure	0.8	0.8	0.00	0.8	0.8	0.00
Ischemic heart disease	1.7	1.5	0.02	1.6	1.7	0.00
Peripheral vascular disease	2.7	2.5	0.01	2.7	2.7	0.00
Pulmonary embolism	0.4	0.4	0.00	0.4	0.4	0.00
Venous thrombosis	0.7	0.9	-0.02	0.8	0.9	0.00
Medical history: Neoplasms						
Hematologic neoplasm	1.0	1.2	-0.02	1.0	1.1	-0.01
Malignant lymphoma	0.6	0.6	-0.01	0.6	0.6	0.00
Malignant neoplasm of anorectum	0.1	0.1	0.00	0.1	0.1	0.00
Malignant neoplastic disease	7.8	9.7	-0.07	8.6	9.0	-0.01
Malignant tumor of colon	0.3	0.4	-0.01	0.3	0.3	0.00
Malignant tumor of lung	0.3	0.5	-0.03	0.4	0.4	0.00
Malignant tumor of urinary bladder	0.5	0.5	-0.01	0.5	0.5	0.00
Primary malignant neoplasm of prostate	3.0	4.1	-0.06	3.5	3.6	-0.01
Medication use						
Antibacterials for systemic use	15.6	17.6	-0.05	16.4	16.7	-0.01
Antiepileptics	23.2	21.2	0.05	22.4	22.3	0.00
Antiinflammatory and antirheumatic products	30.2	33.3	-0.06	31.6	31.7	0.00
Antineoplastic agents	2.4	2.9	-0.03	2.6	2.7	-0.01
Antipsoriatics	0.6	0.8	-0.02	0.7	0.7	0.00
Antithrombotic agents	23.4	20.3	0.07	22.1	22.0	0.00
Drugs for acid related disorders	33.6	33.8	0.00	33.7	34.3	-0.01
Drugs for obstructive airway diseases	25.1	28.7	-0.08	26.8	27.2	-0.01
Drugs used in diabetes	40.1	14.8	0.59	28.0	26.8	0.03
Immunosuppressants	2.5	2.9	-0.03	2.7	2.8	0.00
Lipid modifying agents	65.4	50.4	0.31	58.1	57.3	0.01
Opioids	9.5	9.8	-0.01	9.7	9.8	0.00
Psycholeptics	19.3	20.6	-0.03	20.0	20.2	0.00
Psychostimulants, agents used for adhd and nootropics	1.5	1.4	0.01	1.4	1.4	0.00

Supplementary Table 34: Baseline patient characteristcs for ACEI +combo (T) and CCB/THZ +combo (C) prevalent-users in the VA-OMOP data source. We report proportion of initiators satisfying selected base-line characteristics and the standardized difference of population proportions (StdDiff) before and after stratification. Less extreme StdDiffs through stratification suggest improved balance between patient cohorts through propensity score adjustment.

	Before stratification			After stratification		
Characteristic	T (%)	C (%)	StdDiff	T (%)	C (%)	StdDiff
Age group						
30-34	0.5	0.5	0.01	0.5	0.5	0.00
35-39	1.3	1.2	0.01	1.3	1.2	0.00
40-44	1.8	1.8	0.00	1.8	1.8	0.00
50-54	5.6	5.7	-0.01	5.5	5.6	0.00
55-59	7.9	8.4	-0.02	8.0	8.1	0.00
60-64	11.5	12.1	-0.02	11.7	11.8	0.00
65-69	15.3	15.1	0.00	15.3	15.1	0.00
75-79	11.8	11.6	0.01	11.9	11.7	0.00
85-89	4.5	4.8	-0.01	4.7	4.8	0.00
90-94	1.8	2.1	-0.02	2.1	2.1	0.00
Gender: female	4.0	7.5	-0.15	5.6	6.0	-0.02
Race						
race = Asian	0.8	0.9	-0.02	0.8	0.9	0.00
race = Black or African American	12.3	28.7	-0.42	21.3	21.8	-0.01
race = White	79.2	63.2	0.36	70.5	69.9	0.01
race = Unknown	6.1	5.7	0.02	5.9	5.9	0.00
race = Native Hawaiian or Other Pacific Islander	0.9	0.8	0.01	0.8	0.9	0.00
race = American Indian or Alaska Native	0.8	0.6	0.03	0.7	0.6	0.00
Ethnicity						
ethnicity = Hispanic or Latino	6.4	5.1	0.06	5.7	5.6	0.00
ethnicity = Not Hispanic or Latino	91.1	92.5	-0.05	91.8	91.9	0.00
Medical history: General						
Attention deficit hyperactivity disorder	0.6	0.5	0.01	0.5	0.5	0.00
Chronic liver disease	2.2	2.6	-0.03	2.4	2.4	0.00
Chronic obstructive lung disease	11.8	11.3	0.02	11.7	11.5	0.01
Crohn’s disease	0.2	0.2	0.00	0.2	0.2	0.00
Depressive disorder	16.9	17.6	-0.02	17.2	17.3	0.00
Diabetes mellitus	40.8	30.5	0.22	35.3	34.8	0.01
Gastrointestinal hemorrhage	1.0	1.0	-0.01	1.0	1.0	0.00
Human immunodeficiency virus infection	0.5	0.6	-0.01	0.6	0.5	0.00
Hyperlipidemia	51.8	46.9	0.10	49.0	48.7	0.00
Hypertensive disorder	69.8	74.4	-0.10	72.5	72.7	-0.01
Lesion of liver	1.7	1.9	-0.01	1.8	1.8	0.00
Obesity	14.1	14.2	0.00	14.1	14.2	0.00
Osteoarthritis	14.9	16.7	-0.05	15.9	16.0	0.00
Pneumonia	1.4	1.5	-0.01	1.4	1.4	0.00
Psoriasis	1.2	1.1	0.01	1.2	1.1	0.00
Renal impairment	9.5	13.7	-0.13	11.8	12.2	-0.01
Schizophrenia	1.0	1.0	0.00	1.0	1.0	0.00

(Continued)	Before stratification			After stratification		
Characteristic	T (%)	C (%)	StdDiff	T (%)	C (%)	StdDiff
Ulcerative colitis	0.3	0.3	0.00	0.3	0.3	0.00
Urinary tract infectious disease	1.8	1.9	-0.01	1.9	1.9	0.00
Viral hepatitis C	1.3	1.8	-0.04	1.5	1.6	0.00
Visual system disorder	30.5	31.4	-0.02	31.0	31.2	0.00
Medical history: Cardiovascular disease						
Atrial fibrillation	10.1	6.7	0.12	8.5	7.9	0.02
Cerebrovascular disease	3.4	3.5	-0.01	3.5	3.5	0.00
Coronary arteriosclerosis	18.5	12.6	0.16	15.4	14.7	0.02
Heart disease	33.4	25.1	0.18	29.2	28.1	0.02
Heart failure	8.2	4.6	0.15	6.6	5.4	0.05
Ischemic heart disease	6.9	4.7	0.09	5.8	5.3	0.02
Peripheral vascular disease	5.0	4.6	0.02	4.8	4.8	0.00
Pulmonary embolism	0.6	0.5	0.01	0.5	0.5	0.00
Venous thrombosis	1.1	1.2	-0.01	1.1	1.1	0.00
Medical history: Neoplasms						
Malignant lymphoma	0.7	0.7	0.00	0.7	0.7	0.00
Malignant neoplastic disease	9.0	10.3	-0.04	9.7	9.8	0.00
Malignant tumor of colon	0.3	0.4	0.00	0.3	0.4	0.00
Malignant tumor of lung	0.5	0.6	-0.01	0.5	0.6	0.00
Malignant tumor of urinary bladder	0.6	0.6	0.00	0.6	0.6	0.00
Primary malignant neoplasm of prostate	3.2	4.1	-0.05	3.7	3.8	-0.01
Medication use						
Antibacterials for systemic use	18.9	20.0	-0.03	19.5	19.5	0.00
Antidepressants	33.2	33.3	0.00	33.2	33.3	0.00
Antiepileptics	25.7	24.6	0.03	25.3	25.2	0.00
Antiinflammatory and antirheumatic products	27.8	31.2	-0.07	29.5	30.1	-0.01
Antineoplastic agents	2.7	3.1	-0.02	2.9	3.0	0.00
Antipsoriatics	0.9	1.6	-0.07	1.1	1.3	-0.02
Antithrombotic agents	39.1	33.1	0.13	36.1	35.4	0.01
Drugs used in diabetes	42.6	30.3	0.26	35.8	35.4	0.01
Immunosuppressants	2.7	3.4	-0.04	3.1	3.2	0.00
Lipid modifying agents	73.7	64.3	0.20	68.4	68.1	0.01
Opioids	11.5	11.6	0.00	11.6	11.6	0.00
Psycholeptics	20.8	21.4	-0.01	21.2	21.1	0.00
Psychostimulants, agents used for adhd and nootropics	1.2	1.3	0.00	1.3	1.3	0.00

Supplementary Table 35: Baseline patient characteristcs for ACEI mono (T) and CCB mono (C) prevalent-users in the VA-OMOP data source. We report proportion of initiators satisfying selected base-line characteristics and the standardized difference of population proportions (StdDiff) before and after stratification. Less extreme StdDiffs through stratification suggest improved balance between patient cohorts through propensity score adjustment.

	Before stratification			After stratification		
Characteristic	T (%)	C (%)	StdDiff	T (%)	C (%)	StdDiff
Age group						
25-29	0.2	0.2	0.00	0.2	0.2	0.01
30-34	0.9	0.9	0.00	0.9	0.8	0.00
35-39	2.0	1.9	0.01	2.0	1.9	0.00
40-44	2.8	2.4	0.02	2.7	2.5	0.01
45-49	4.8	4.0	0.04	4.6	4.3	0.01
50-54	7.4	6.4	0.04	7.1	6.6	0.02
55-59	9.7	9.5	0.01	9.6	9.3	0.01
60-64	12.4	13.5	-0.03	12.6	12.5	0.00
65-69	14.8	14.8	0.00	14.7	14.8	0.00
70-74	25.1	22.3	0.07	24.0	24.5	-0.01
75-79	9.9	9.8	0.00	9.9	10.2	-0.01
80-84	4.7	5.6	-0.04	5.1	5.4	-0.01
85-89	3.5	5.0	-0.08	4.2	4.3	-0.01
90-94	1.3	2.5	-0.09	1.9	1.8	0.00
95-99	0.4	0.9	-0.06	0.6	0.6	0.00
Gender: female	4.7	7.3	-0.11	5.4	5.6	-0.01
Race						
race = Black or African American	12.6	33.1	-0.50	19.2	20.3	-0.03
race = White	78.0	58.7	0.42	71.7	71.0	0.02
race = Unknown	6.5	5.8	0.03	6.4	6.2	0.01
race = Native Hawaiian or Other Pacific Islander	1.0	0.8	0.02	0.9	0.9	0.00
race = American Indian or Alaska Native	0.9	0.6	0.03	0.8	0.8	0.00
Ethnicity						
ethnicity = Hispanic or Latino	7.8	5.4	0.10	7.0	6.8	0.01
ethnicity = Not Hispanic or Latino	89.6	92.2	-0.09	90.5	90.7	-0.01
Medical history: General						
Acute respiratory disease	4.2	5.0	-0.04	4.4	4.5	0.00
Attention deficit hyperactivity disorder	0.8	0.6	0.02	0.7	0.7	0.00
Chronic liver disease	1.7	2.8	-0.07	2.0	2.1	-0.01
Chronic obstructive lung disease	7.6	9.9	-0.08	8.4	8.7	-0.01
Crohn’s disease	0.2	0.2	-0.01	0.2	0.2	0.00
Dementia	2.0	3.0	-0.06	2.4	2.6	-0.01
Depressive disorder	16.9	17.6	-0.02	17.1	17.3	0.00
Diabetes mellitus	38.0	16.0	0.51	30.3	29.5	0.02
Gastroesophageal reflux disease	13.6	14.2	-0.02	13.8	14.1	-0.01
Gastrointestinal hemorrhage	0.7	0.9	-0.03	0.7	0.8	-0.01
Human immunodeficiency virus infection	0.6	0.8	-0.03	0.7	0.6	0.00
Hyperlipidemia	48.3	39.1	0.18	45.0	44.4	0.01
Hypertensive disorder	68.0	71.0	-0.06	69.0	69.8	-0.02

(Continued)	Before stratification			After stratification		
Characteristic	T (%)	C (%)	StdDiff	T (%)	C (%)	StdDiff
Lesion of liver	1.3	1.8	-0.04	1.4	1.5	0.00
Obesity	12.4	9.0	0.11	11.3	11.0	0.01
Osteoarthritis	14.2	15.2	-0.03	14.5	14.8	-0.01
Pneumonia	0.7	1.1	-0.05	0.8	0.9	-0.01
Psoriasis	1.2	1.0	0.02	1.1	1.1	0.00
Renal impairment	5.7	8.5	-0.11	6.6	7.3	-0.03
Schizophrenia	0.9	1.3	-0.04	1.0	1.1	-0.01
Urinary tract infectious disease	1.3	1.8	-0.04	1.4	1.5	-0.01
Viral hepatitis C	1.1	2.2	-0.08	1.4	1.6	-0.01
Visual system disorder	28.3	27.8	0.01	28.1	28.1	0.00
Medical history: Cardiovascular disease						
Atrial fibrillation	2.4	2.5	-0.01	2.5	2.7	-0.01
Cerebrovascular disease	2.3	2.6	-0.02	2.4	2.6	-0.01
Coronary arteriosclerosis	5.9	5.1	0.03	5.7	6.1	-0.01
Heart disease	13.0	12.9	0.00	13.1	13.9	-0.02
Heart failure	0.8	0.9	-0.01	0.8	0.9	-0.01
Peripheral vascular disease	2.7	2.8	0.00	2.8	2.9	-0.01
Venous thrombosis	0.7	1.0	-0.03	0.8	0.9	-0.01
Medical history: Neoplasms						
Hematologic neoplasm	1.0	1.3	-0.03	1.1	1.1	-0.01
Malignant lymphoma	0.6	0.7	-0.01	0.6	0.6	0.00
Malignant neoplasm of anorectum	0.1	0.1	-0.01	0.1	0.1	0.00
Malignant neoplastic disease	7.8	10.5	-0.09	8.7	9.1	-0.02
Malignant tumor of breast	0.1	0.1	-0.02	0.1	0.1	0.00
Malignant tumor of colon	0.3	0.4	-0.02	0.3	0.4	-0.01
Malignant tumor of lung	0.3	0.6	-0.04	0.4	0.4	0.00
Malignant tumor of urinary bladder	0.5	0.6	-0.02	0.5	0.5	0.00
Primary malignant neoplasm of prostate	3.0	4.3	-0.07	3.4	3.6	-0.01
Medication use						
Antibacterials for systemic use	15.6	17.9	-0.06	16.3	16.7	-0.01
Antidepressants	32.2	31.3	0.02	31.9	32.1	0.00
Antiepileptics	23.2	21.0	0.05	22.5	22.6	0.00
Antiinflammatory and antirheumatic products	30.2	31.5	-0.03	30.6	30.5	0.00
Antineoplastic agents	2.4	2.9	-0.03	2.6	2.6	0.00
Antipsoriatics	0.6	0.9	-0.03	0.6	0.7	-0.01
Antithrombotic agents	23.4	21.6	0.04	22.8	23.1	-0.01
Drugs for acid related disorders	33.6	33.9	-0.01	33.7	34.3	-0.01
Drugs for obstructive airway diseases	25.1	28.3	-0.07	26.3	26.6	-0.01
Drugs used in diabetes	40.1	14.2	0.61	30.8	29.9	0.02
Immunosuppressants	2.5	3.1	-0.04	2.7	2.8	-0.01
Lipid modifying agents	65.4	49.6	0.32	59.8	58.8	0.02
Opioids	9.5	9.8	-0.01	9.6	9.9	-0.01
Psycholeptics	19.3	21.0	-0.04	19.9	20.4	-0.01
Psychostimulants, agents used for adhd and nootropics	1.5	1.3	0.02	1.4	1.4	0.00

Supplementary Table 36: Baseline patient characteristcs for ACEI +combo (T) and CCB +combo (C) prevalent-users in the VA-OMOP data source. We report proportion of initiators satisfying selected base-line characteristics and the standardized difference of population proportions (StdDiff) before and after stratification. Less extreme StdDiffs through stratification suggest improved balance between patient cohorts through propensity score adjustment.

	Before stratification			After stratification		
Characteristic	T (%)	C (%)	StdDiff	T (%)	C (%)	StdDiff
Age group						
25-29	0.1	0.1	0.01	0.1	0.1	0.01
30-34	0.5	0.5	0.01	0.5	0.5	0.00
35-39	1.3	1.1	0.02	1.2	1.2	0.00
40-44	1.9	1.6	0.02	1.8	1.7	0.01
45-49	3.5	3.1	0.03	3.4	3.2	0.01
50-54	5.9	5.3	0.03	5.6	5.6	0.00
55-59	8.4	8.1	0.01	8.2	8.2	0.00
60-64	11.9	12.1	-0.01	12.0	11.9	0.00
65-69	15.5	15.3	0.01	15.4	15.4	0.00
70-74	27.9	26.2	0.04	27.2	27.5	-0.01
75-79	11.4	11.7	-0.01	11.5	11.7	0.00
80-84	5.5	6.4	-0.04	5.9	5.8	0.00
85-89	4.0	5.3	-0.06	4.6	4.6	0.00
90-94	1.6	2.4	-0.06	1.9	2.0	-0.01
95-99	0.4	0.8	-0.05	0.6	0.6	-0.01
Race						
race = Asian	0.7	1.0	-0.03	0.8	0.8	0.00
race = Black or African American	14.7	30.7	-0.39	20.7	21.3	-0.01
race = White	77.0	61.2	0.35	71.0	70.6	0.01
race = Unknown	6.0	5.7	0.01	5.9	5.9	0.00
race = Native Hawaiian or Other Pacific Islander	0.8	0.8	0.00	0.8	0.8	0.00
race = American Indian or Alaska Native	0.8	0.6	0.02	0.7	0.7	0.00
Ethnicity						
ethnicity = Hispanic or Latino	6.1	5.2	0.04	5.8	5.7	0.00
ethnicity = Not Hispanic or Latino	91.4	92.4	-0.04	91.8	91.9	0.00
Medical history: General						
Acute respiratory disease	5.2	6.1	-0.04	5.4	5.5	0.00
Attention deficit hyperactivity disorder	0.6	0.4	0.02	0.5	0.5	0.00
Chronic liver disease	2.1	2.9	-0.05	2.3	2.4	-0.01
Chronic obstructive lung disease	10.8	11.8	-0.03	11.2	11.2	0.00
Crohn’s disease	0.2	0.2	-0.01	0.2	0.2	0.00
Dementia	2.1	2.9	-0.05	2.4	2.5	-0.01
Depressive disorder	16.5	17.8	-0.04	16.9	17.1	0.00
Diabetes mellitus	39.4	31.2	0.17	36.0	36.2	0.00
Gastroesophageal reflux disease	14.6	16.0	-0.04	15.1	15.2	0.00
Gastrointestinal hemorrhage	0.9	1.1	-0.02	1.0	1.0	0.00
Human immunodeficiency virus infection	0.5	0.6	-0.01	0.5	0.5	0.00
Hyperlipidemia	50.9	47.1	0.08	49.4	49.3	0.00
Hypertensive disorder	70.5	75.3	-0.11	72.4	72.8	-0.01

(Continued)	Before stratification			After stratification		
Characteristic	T (%)	C (%)	StdDiff	T (%)	C (%)	StdDiff
Lesion of liver	1.6	2.1	-0.04	1.8	1.8	-0.01
Obesity	14.6	13.3	0.04	14.1	14.1	0.00
Osteoarthritis	15.2	16.5	-0.04	15.7	15.9	0.00
Pneumonia	1.2	1.7	-0.04	1.3	1.4	-0.01
Renal impairment	8.9	16.1	-0.22	10.8	12.4	-0.05
Rheumatoid arthritis	0.7	0.8	-0.01	0.8	0.8	0.00
Schizophrenia	0.9	1.1	-0.02	1.0	1.0	0.00
Ulcerative colitis	0.3	0.3	0.00	0.3	0.3	0.00
Urinary tract infectious disease	1.6	2.2	-0.04	1.8	1.9	-0.01
Viral hepatitis C	1.3	2.1	-0.06	1.5	1.6	-0.01
Visual system disorder	30.1	32.2	-0.04	30.8	31.2	-0.01
Medical history: Cardiovascular disease						
Atrial fibrillation	8.6	6.7	0.07	8.0	7.8	0.01
Cerebrovascular disease	3.1	4.0	-0.05	3.4	3.5	-0.01
Coronary arteriosclerosis	15.8	13.8	0.06	15.0	15.0	0.00
Heart disease	29.2	26.8	0.05	28.1	28.4	-0.01
Heart failure	6.4	5.2	0.05	5.9	5.7	0.01
Ischemic heart disease	5.7	5.3	0.02	5.5	5.5	0.00
Peripheral vascular disease	4.6	5.2	-0.03	4.7	4.8	0.00
Venous thrombosis	1.0	1.3	-0.03	1.1	1.1	-0.01
Medical history: Neoplasms						
Hematologic neoplasm	1.1	1.4	-0.03	1.2	1.2	-0.01
Malignant lymphoma	0.6	0.7	-0.01	0.7	0.7	0.00
Malignant neoplasm of anorectum	0.1	0.1	-0.01	0.1	0.1	0.00
Malignant neoplastic disease	8.7	11.0	-0.08	9.4	9.8	-0.01
Malignant tumor of breast	0.1	0.1	-0.01	0.1	0.1	0.00
Malignant tumor of colon	0.3	0.4	-0.01	0.3	0.4	0.00
Malignant tumor of lung	0.5	0.7	-0.03	0.5	0.5	0.00
Malignant tumor of urinary bladder	0.5	0.7	-0.01	0.6	0.6	0.00
Primary malignant neoplasm of prostate	3.2	4.4	-0.06	3.6	3.7	0.00
Medication use						
Antibacterials for systemic use	18.2	20.7	-0.06	18.9	19.4	-0.01
Antidepressants	32.5	33.5	-0.02	32.9	33.1	0.00
Antiepileptics	25.1	25.0	0.00	25.0	25.2	0.00
Antiinflammatory and antirheumatic products	29.1	30.3	-0.03	29.8	29.7	0.00
Antineoplastic agents	2.7	3.2	-0.03	2.9	3.0	-0.01
Antipsoriatics	0.8	1.9	-0.10	0.9	1.4	-0.04
Antithrombotic agents	35.8	35.4	0.01	35.4	35.8	-0.01
Drugs for acid related disorders	38.7	41.6	-0.06	39.8	40.2	-0.01
Drugs for obstructive airway diseases	29.2	32.5	-0.07	30.4	30.7	0.00
Drugs used in diabetes	41.2	30.7	0.22	36.9	37.1	0.00
Immunosuppressants	2.6	3.6	-0.06	2.9	3.1	-0.01
Lipid modifying agents	72.3	64.9	0.16	69.3	69.4	0.00
Opioids	11.2	12.1	-0.03	11.4	11.8	-0.01

(Continued)	Before stratification			After stratification		
Characteristic	T (%)	C (%)	StdDiff	T (%)	C (%)	StdDiff
Psycholeptics	20.1	22.0	-0.05	20.7	21.0	-0.01

Supplementary Table 37: Baseline patient characteristcs for ACEI mono (T) and THZ mono (C) prevalent-users in the VA-OMOP data source. We report proportion of initiators satisfying selected base-line characteristics and the standardized difference of population proportions (StdDiff) before and after stratification. Less extreme StdDiffs through stratification suggest improved balance between patient cohorts through propensity score adjustment.

	Before stratification			After stratification		
Characteristic	T (%)	C (%)	StdDiff	T (%)	C (%)	StdDiff
Age group						
25-29	0.2	0.3	-0.01	0.3	0.2	0.01
30-34	0.9	1.2	-0.03	0.9	0.9	0.01
35-39	2.0	2.6	-0.04	2.1	2.1	0.01
40-44	2.8	3.5	-0.04	2.9	2.8	0.01
45-49	4.8	5.4	-0.03	4.9	4.7	0.01
50-54	7.4	8.3	-0.03	7.6	7.2	0.01
55-59	9.7	10.9	-0.04	9.9	9.7	0.01
60-64	12.4	13.1	-0.02	12.6	12.1	0.01
65-69	14.8	14.3	0.01	14.7	14.8	0.00
70-74	25.1	22.0	0.07	24.5	24.5	0.00
75-79	9.9	9.0	0.03	9.8	10.3	-0.02
80-84	4.7	4.4	0.02	4.7	5.0	-0.02
85-89	3.5	3.2	0.01	3.4	3.7	-0.02
90-94	1.3	1.3	0.00	1.3	1.6	-0.02
95-99	0.4	0.4	-0.01	0.4	0.5	-0.01
Gender: female	4.7	12.9	-0.29	6.0	6.6	-0.03
Race						
race = Asian	1.0	0.7	0.03	0.9	0.9	0.01
race = Black or African American	12.6	28.8	-0.41	15.4	15.8	-0.01
race = White	78.0	63.3	0.33	75.5	75.4	0.00
race = Unknown	6.5	5.8	0.03	6.4	6.2	0.01
race = Native Hawaiian or Other Pacific Islander	1.0	0.8	0.02	1.0	1.0	0.00
race = American Indian or Alaska Native	0.9	0.6	0.04	0.8	0.7	0.01
Ethnicity						
ethnicity = Hispanic or Latino	7.8	5.1	0.11	7.3	7.1	0.00
ethnicity = Not Hispanic or Latino	89.6	92.5	-0.10	90.2	90.4	-0.01
Medical history: General						
Acute respiratory disease	4.2	4.9	-0.04	4.3	4.6	-0.01
Chronic liver disease	1.7	1.9	-0.01	1.7	1.8	0.00
Chronic obstructive lung disease	7.6	8.0	-0.02	7.6	8.1	-0.02
Crohn's disease	0.2	0.2	0.00	0.2	0.2	0.01
Depressive disorder	16.9	17.6	-0.02	17.0	17.0	0.00
Diabetes mellitus	38.0	15.5	0.53	33.6	33.1	0.01
Gastroesophageal reflux disease	13.6	14.0	-0.01	13.7	14.1	-0.01
Gastrointestinal hemorrhage	0.7	0.6	0.00	0.7	0.6	0.00
Human immunodeficiency virus infection	0.6	0.7	-0.02	0.6	0.6	0.00
Hyperlipidemia	48.3	39.3	0.18	46.5	45.7	0.02
Hypertensive disorder	68.0	68.2	0.00	68.0	68.8	-0.02
Lesion of liver	1.3	1.2	0.01	1.2	1.2	0.00

(Continued)	Before stratification			After stratification		
Characteristic	T (%)	C (%)	StdDiff	T (%)	C (%)	StdDiff
Obesity	12.4	13.0	-0.02	12.5	12.6	0.00
Osteoarthritis	14.2	16.1	-0.05	14.5	15.1	-0.01
Pneumonia	0.7	0.6	0.00	0.7	0.7	-0.01
Psoriasis	1.2	1.0	0.01	1.2	1.1	0.01
Renal impairment	5.7	4.1	0.08	5.4	6.0	-0.03
Rheumatoid arthritis	0.7	0.8	-0.01	0.7	0.7	0.00
Schizophrenia	0.9	0.9	0.00	0.9	1.0	0.00
Urinary tract infectious disease	1.3	1.1	0.01	1.2	1.3	0.00
Viral hepatitis C	1.1	1.4	-0.02	1.2	1.3	-0.01
Medical history: Cardiovascular disease						
Atrial fibrillation	2.4	1.9	0.04	2.3	2.8	-0.03
Cerebrovascular disease	2.3	1.5	0.05	2.1	2.2	-0.01
Coronary arteriosclerosis	5.9	3.2	0.13	5.4	5.8	-0.02
Heart disease	13.0	9.6	0.11	12.3	13.2	-0.03
Heart failure	0.8	0.6	0.02	0.8	0.9	-0.01
Ischemic heart disease	1.7	1.0	0.06	1.6	1.7	-0.01
Peripheral vascular disease	2.7	1.9	0.05	2.6	2.7	-0.01
Pulmonary embolism	0.4	0.3	0.01	0.4	0.4	0.00
Medical history: Neoplasms						
Hematologic neoplasm	1.0	1.0	0.00	0.9	1.1	-0.01
Malignant lymphoma	0.6	0.6	0.00	0.6	0.6	0.00
Malignant neoplasm of anorectum	0.1	0.1	0.01	0.1	0.1	0.02
Malignant neoplastic disease	7.8	7.9	0.00	7.8	8.0	-0.01
Malignant tumor of colon	0.3	0.2	0.01	0.3	0.2	0.00
Malignant tumor of lung	0.3	0.4	-0.01	0.3	0.4	-0.01
Malignant tumor of urinary bladder	0.5	0.4	0.02	0.4	0.4	0.00
Primary malignant neoplasm of prostate	3.0	3.3	-0.02	3.1	3.2	-0.01
Medication use						
Antibacterials for systemic use	15.6	16.3	-0.02	15.7	16.0	-0.01
Antidepressants	32.2	31.7	0.01	32.0	32.3	-0.01
Antiepileptics	23.2	20.9	0.05	22.7	23.3	-0.01
Antiinflammatory and antirheumatic products	30.2	35.0	-0.10	31.1	31.2	0.00
Antineoplastic agents	2.4	2.9	-0.03	2.5	2.6	-0.01
Antipsoriatics	0.6	0.6	0.00	0.6	0.6	-0.01
Antithrombotic agents	23.4	16.3	0.18	22.0	21.8	0.00
Drugs for acid related disorders	33.6	32.8	0.02	33.4	34.4	-0.02
Drugs for obstructive airway diseases	25.1	28.7	-0.08	25.7	26.7	-0.02
Drugs used in diabetes	40.1	14.2	0.61	35.0	34.0	0.02
Immunosuppressants	2.5	2.7	-0.01	2.5	2.6	0.00
Lipid modifying agents	65.4	49.2	0.33	62.2	60.4	0.04
Opioids	9.5	9.4	0.00	9.5	9.8	-0.01
Psycholeptics	19.3	19.5	0.00	19.3	19.7	-0.01
Psychostimulants, agents used for adhd and nootropics	1.5	1.7	-0.02	1.5	1.7	-0.01

Supplementary Table 38: Baseline patient characteristcs for ACEI +combo (T) and THZ +combo (C) prevalent-users in the VA-OMOP data source. We report proportion of initiators satisfying selected base-line characteristics and the standardized difference of population proportions (StdDiff) before and after stratification. Less extreme StdDiffs through stratification suggest improved balance between patient cohorts through propensity score adjustment.

	Before stratification			After stratification		
Characteristic	T (%)	C (%)	StdDiff	T (%)	C (%)	StdDiff
Age group						
30-34	0.5	0.5	-0.01	0.5	0.5	0.00
35-39	1.1	1.3	-0.02	1.2	1.1	0.00
40-44	1.6	2.1	-0.03	1.7	1.8	0.00
45-49	3.1	3.8	-0.04	3.3	3.3	0.00
50-54	5.2	6.5	-0.06	5.6	5.6	0.00
55-59	7.7	9.2	-0.05	8.1	8.2	0.00
60-64	11.5	12.5	-0.03	11.7	11.7	0.00
65-69	15.5	15.3	0.01	15.4	15.4	0.00
70-74	28.4	26.6	0.04	27.9	28.1	0.00
75-79	12.1	11.1	0.03	11.9	11.8	0.00
80-84	6.0	5.3	0.03	5.9	5.8	0.00
85-89	4.6	3.8	0.04	4.4	4.4	0.00
90-94	1.9	1.5	0.03	1.8	1.8	0.00
Gender: female	3.8	9.2	-0.22	5.1	5.7	-0.03
Race						
race = Asian	0.8	0.8	0.00	0.8	0.8	0.00
race = Black or African American	15.3	28.1	-0.31	19.2	19.6	-0.01
race = White	76.3	64.1	0.27	72.5	72.1	0.01
race = Unknown	6.0	5.7	0.01	5.9	6.0	0.00
race = Native Hawaiian or Other Pacific Islander	0.9	0.8	0.01	0.8	0.9	0.00
race = American Indian or Alaska Native	0.8	0.6	0.02	0.7	0.7	0.00
Ethnicity						
ethnicity = Hispanic or Latino	6.2	4.9	0.06	5.8	5.8	0.00
ethnicity = Not Hispanic or Latino	91.3	92.8	-0.05	91.7	91.8	0.00
Medical history: General						
Acute respiratory disease	5.4	5.6	-0.01	5.5	5.4	0.00
Attention deficit hyperactivity disorder	0.5	0.5	0.00	0.5	0.5	0.00
Chronic liver disease	2.3	2.1	0.02	2.3	2.2	0.00
Chronic obstructive lung disease	11.8	10.0	0.06	11.3	11.1	0.00
Crohn’s disease	0.2	0.2	0.00	0.2	0.2	0.00
Dementia	2.6	1.5	0.08	2.3	2.1	0.01
Depressive disorder	16.8	17.3	-0.01	16.9	16.9	0.00
Diabetes mellitus	41.2	31.3	0.21	38.2	38.5	0.00
Gastroesophageal reflux disease	15.0	15.5	-0.01	15.2	15.2	0.00
Gastrointestinal hemorrhage	1.0	0.8	0.02	0.9	0.9	0.00
Human immunodeficiency virus infection	0.5	0.5	0.00	0.5	0.5	0.00
Hyperlipidemia	51.8	47.2	0.09	50.3	50.2	0.00
Hypertensive disorder	71.5	74.0	-0.06	72.3	72.8	-0.01
Lesion of liver	1.8	1.6	0.02	1.7	1.7	0.00

(Continued)	Before stratification			After stratification		
Characteristic	T (%)	C (%)	StdDiff	T (%)	C (%)	StdDiff
Obesity	14.1	16.3	-0.06	14.8	15.1	-0.01
Osteoarthritis	15.1	17.3	-0.06	15.8	15.8	0.00
Pneumonia	1.4	1.1	0.03	1.3	1.2	0.01
Psoriasis	1.2	1.1	0.01	1.2	1.2	0.00
Renal impairment	11.0	9.8	0.04	10.9	11.0	0.00
Rheumatoid arthritis	0.8	0.8	0.00	0.8	0.8	0.00
Schizophrenia	1.0	0.8	0.02	0.9	0.9	0.00
Urinary tract infectious disease	1.9	1.5	0.03	1.8	1.6	0.01
Viral hepatitis C	1.4	1.4	0.01	1.4	1.4	0.00
Visual system disorder	31.3	30.8	0.01	31.1	31.3	0.00
Medical history: Cardiovascular disease						
Atrial fibrillation	9.7	6.1	0.14	8.7	8.0	0.02
Cerebrovascular disease	3.7	2.8	0.05	3.4	3.4	0.00
Coronary arteriosclerosis	18.3	10.3	0.23	15.9	14.9	0.03
Heart disease	33.1	22.0	0.25	29.7	28.6	0.03
Heart failure	7.8	3.4	0.19	6.6	5.3	0.05
Ischemic heart disease	6.8	3.7	0.14	5.9	5.3	0.02
Peripheral vascular disease	5.3	3.7	0.08	4.9	4.7	0.01
Pulmonary embolism	0.6	0.5	0.01	0.5	0.5	0.00
Venous thrombosis	1.1	0.9	0.02	1.1	1.0	0.00
Medical history: Neoplasms						
Hematologic neoplasm	1.2	1.1	0.01	1.1	1.1	0.00
Malignant lymphoma	0.7	0.6	0.01	0.7	0.7	0.00
Malignant neoplasm of anorectum	0.1	0.1	0.01	0.1	0.1	0.00
Malignant neoplastic disease	9.4	9.0	0.01	9.3	9.2	0.00
Malignant tumor of colon	0.4	0.3	0.01	0.3	0.3	0.00
Malignant tumor of lung	0.5	0.4	0.02	0.5	0.5	0.00
Malignant tumor of urinary bladder	0.6	0.5	0.02	0.6	0.6	0.00
Medication use						
Antibacterials for systemic use	19.2	18.7	0.01	19.1	18.8	0.01
Antiepileptics	25.8	24.4	0.03	25.3	25.6	-0.01
Antiinflammatory and antirheumatic products	28.1	33.4	-0.12	29.7	30.1	-0.01
Antineoplastic agents	2.7	3.0	-0.02	2.8	2.8	0.00
Antipsoriatics	1.0	1.0	0.00	1.0	1.1	-0.01
Antithrombotic agents	39.8	29.8	0.21	36.7	36.1	0.01
Drugs for acid related disorders	40.3	40.0	0.01	40.2	40.3	0.00
Drugs for obstructive airway diseases	30.1	32.2	-0.04	30.8	31.0	0.00
Drugs used in diabetes	42.7	31.7	0.23	39.3	39.9	-0.01
Immunosuppressants	2.8	2.9	-0.01	2.8	2.9	0.00
Lipid modifying agents	74.0	64.9	0.20	71.0	70.9	0.00
Opioids	11.8	10.9	0.03	11.5	11.4	0.00
Psycholeptics	20.9	20.2	0.02	20.7	20.4	0.00
Psychostimulants, agents used for adhd and nootropics	1.2	1.4	-0.02	1.2	1.3	0.00

Supplementary Table 39: Baseline patient characteristcs for ARB mono (T) and CCB/THZ mono (C) prevalent-users in the VA-OMOP data source. We report proportion of initiators satisfying selected base-line characteristics and the standardized difference of population proportions (StdDiff) before and after stratification. Less extreme StdDiffs through stratification suggest improved balance between patient cohorts through propensity score adjustment.

	Before stratification			After stratification		
Characteristic	T (%)	C (%)	StdDiff	T (%)	C (%)	StdDiff
Age group						
25-29	0.1	0.2	-0.02	0.2	0.2	0.00
30-34	0.6	0.9	-0.04	0.8	0.8	0.00
40-44	2.3	2.8	-0.03	2.7	2.6	0.00
45-49	4.1	4.4	-0.02	4.3	4.4	0.00
50-54	6.7	7.1	-0.02	7.0	7.0	0.00
55-59	8.7	10.0	-0.05	9.5	9.6	0.00
60-64	10.9	13.6	-0.08	12.8	12.8	0.00
65-69	13.6	14.8	-0.03	14.3	14.5	0.00
70-74	25.7	22.2	0.08	23.0	23.0	0.00
75-79	11.7	9.5	0.07	10.2	10.1	0.00
80-84	6.4	5.1	0.06	5.6	5.5	0.00
85-89	5.1	4.4	0.03	4.7	4.6	0.00
90-94	2.0	2.1	0.00	2.2	2.1	0.00
95-99	0.5	0.7	-0.03	0.7	0.7	0.00
Gender: female	6.5	9.0	-0.10	8.3	8.3	0.00
Race						
race = Asian	1.9	0.8	0.09	1.1	1.1	0.00
race = Black or African American	13.7	33.5	-0.48	27.9	28.2	-0.01
race = White	75.9	58.6	0.38	63.6	63.3	0.01
race = Unknown	6.7	5.7	0.04	5.9	6.0	0.00
race = Native Hawaiian or Other Pacific Islander	1.1	0.8	0.03	0.8	0.8	0.00
race = American Indian or Alaska Native	0.7	0.6	0.02	0.6	0.6	0.00
Ethnicity						
ethnicity = Hispanic or Latino	7.3	5.2	0.09	5.8	5.7	0.00
ethnicity = Not Hispanic or Latino	90.0	92.4	-0.09	91.8	91.8	0.00
Medical history: General						
Acute respiratory disease	4.4	5.0	-0.03	4.8	4.9	0.00
Attention deficit hyperactivity disorder	0.7	0.6	0.01	0.6	0.6	0.00
Chronic liver disease	1.3	2.5	-0.09	1.9	2.2	-0.03
Chronic obstructive lung disease	7.9	9.3	-0.05	9.1	9.0	0.01
Crohn’s disease	0.2	0.2	-0.01	0.2	0.2	0.00
Dementia	1.9	2.4	-0.03	2.4	2.3	0.01
Depressive disorder	15.9	17.7	-0.05	17.2	17.2	0.00
Diabetes mellitus	37.0	16.3	0.48	23.4	21.0	0.06
Gastroesophageal reflux disease	14.8	14.2	0.02	14.6	14.4	0.01
Gastrointestinal hemorrhage	0.6	0.8	-0.03	0.7	0.8	-0.01
Human immunodeficiency virus infection	0.4	0.8	-0.05	0.7	0.7	0.00
Hyperlipidemia	47.7	39.6	0.16	42.1	41.5	0.01
Hypertensive disorder	68.9	71.1	-0.05	71.0	70.5	0.01

(Continued)	Before stratification			After stratification		
Characteristic	T (%)	C (%)	StdDiff	T (%)	C (%)	StdDiff
Lesion of liver	1.1	1.6	-0.04	1.4	1.5	-0.01
Obesity	13.2	10.6	0.08	11.6	11.1	0.01
Osteoarthritis	15.1	15.8	-0.02	15.9	15.6	0.01
Pneumonia	0.7	0.9	-0.02	0.8	0.9	-0.01
Psoriasis	1.1	1.0	0.01	1.0	1.0	0.00
Renal impairment	7.2	7.1	0.00	7.7	7.2	0.02
Rheumatoid arthritis	0.8	0.8	0.01	0.9	0.8	0.01
Schizophrenia	0.5	1.2	-0.07	0.9	1.0	-0.01
Ulcerative colitis	0.3	0.3	0.00	0.3	0.3	0.00
Urinary tract infectious disease	1.2	1.5	-0.03	1.6	1.5	0.01
Viral hepatitis C	0.8	2.0	-0.10	1.4	1.7	-0.03
Visual system disorder	29.0	27.7	0.03	28.4	27.9	0.01
Medical history: Cardiovascular disease						
Atrial fibrillation	3.9	2.3	0.10	3.0	2.7	0.02
Cerebrovascular disease	2.3	2.3	0.00	2.5	2.4	0.01
Coronary arteriosclerosis	7.9	4.4	0.14	5.8	5.2	0.03
Heart disease	16.5	11.9	0.14	14.0	13.0	0.03
Heart failure	1.8	0.8	0.09	1.3	0.9	0.04
Ischemic heart disease	2.2	1.5	0.05	1.7	1.6	0.01
Peripheral vascular disease	2.5	2.5	0.00	2.6	2.5	0.00
Pulmonary embolism	0.4	0.4	0.00	0.4	0.4	0.01
Venous thrombosis	0.8	0.9	-0.01	0.9	0.9	0.00
Medical history: Neoplasms						
Hematologic neoplasm	1.1	1.2	-0.01	1.2	1.2	0.00
Malignant lymphoma	0.7	0.6	0.00	0.7	0.6	0.00
Malignant neoplasm of anorectum	0.1	0.1	-0.01	0.1	0.1	0.00
Malignant tumor of breast	0.1	0.1	0.00	0.2	0.1	0.01
Malignant tumor of colon	0.3	0.4	-0.02	0.3	0.3	0.00
Malignant tumor of lung	0.3	0.5	-0.03	0.5	0.5	0.00
Primary malignant neoplasm of prostate	3.1	4.1	-0.05	3.7	3.8	0.00
Medication use						
Antibacterials for systemic use	15.5	17.6	-0.06	17.0	17.0	0.00
Antidepressants	30.8	31.6	-0.02	31.7	31.4	0.01
Antiepileptics	21.6	21.2	0.01	21.9	21.2	0.02
Antiinflammatory and antirheumatic products	28.6	33.3	-0.10	32.1	32.0	0.00
Antineoplastic agents	2.8	2.9	-0.01	2.9	2.8	0.00
Antipsoriatics	0.7	0.8	-0.01	0.8	0.8	0.00
Antithrombotic agents	23.5	20.3	0.08	21.8	21.1	0.02
Drugs for acid related disorders	35.5	33.8	0.04	34.7	34.4	0.01
Drugs for obstructive airway diseases	28.6	28.7	0.00	28.9	28.7	0.00
Drugs used in diabetes	37.6	14.8	0.54	22.0	20.0	0.05
Immunosuppressants	3.0	2.9	0.01	3.2	3.0	0.01
Opioids	8.2	9.8	-0.06	9.6	9.4	0.00
Psycholeptics	17.8	20.6	-0.07	20.1	19.9	0.00

(Continued)

Characteristic	Before stratification			After stratification		
	T (%)	C (%)	StdDiff	T (%)	C (%)	StdDiff
Psychostimulants, agents used for adhd and nootropics	1.4	1.4	0.00	1.5	1.4	0.01

Supplementary Table 40: Baseline patient characteristcs for ARB +combo (T) and CCB/THZ +combo (C) prevalent-users in the VA-OMOP data source. We report proportion of initiators satisfying selected base-line characteristics and the standardized difference of population proportions (StdDiff) before and after stratification. Less extreme StdDiffs through stratification suggest improved balance between patient cohorts through propensity score adjustment.

	Before stratification			After stratification		
Characteristic	T (%)	C (%)	StdDiff	T (%)	C (%)	StdDiff
Age group						
25-29	0.1	0.1	-0.01	0.1	0.1	0.00
30-34	0.3	0.5	-0.02	0.5	0.4	0.00
35-39	0.9	1.2	-0.03	1.2	1.1	0.00
45-49	2.8	3.3	-0.03	3.2	3.2	0.00
50-54	4.8	5.7	-0.04	5.6	5.5	0.00
55-59	7.0	8.5	-0.06	8.3	8.2	0.00
60-64	10.0	12.4	-0.08	12.0	11.9	0.00
65-69	14.1	15.6	-0.04	15.2	15.3	0.00
70-74	28.6	26.9	0.04	26.9	27.2	-0.01
75-79	13.7	11.3	0.07	11.9	11.7	0.00
80-84	7.5	5.8	0.07	6.1	6.1	0.00
85-89	5.9	4.4	0.07	4.7	4.7	0.00
90-94	2.4	1.9	0.04	2.0	2.0	0.00
95-99	0.6	0.6	0.01	0.6	0.6	0.00
Gender: female	5.5	6.2	-0.03	6.0	6.1	0.00
Race						
race = Asian	1.4	0.7	0.07	0.8	0.8	0.00
race = Black or African American	14.7	27.1	-0.31	24.9	24.5	0.01
race = White	76.0	65.2	0.24	67.1	67.4	-0.01
race = Unknown	6.2	5.7	0.02	5.7	5.8	-0.01
race = Native Hawaiian or Other Pacific Islander	1.0	0.8	0.02	0.8	0.8	0.00
race = American Indian or Alaska Native	0.7	0.6	0.01	0.6	0.6	0.00
Ethnicity						
ethnicity = Hispanic or Latino	6.0	5.1	0.04	5.3	5.3	0.00
ethnicity = Not Hispanic or Latino	91.4	92.5	-0.04	92.5	92.2	0.01
Medical history: General						
Acute respiratory disease	6.4	5.4	0.04	5.8	5.5	0.01
Attention deficit hyperactivity disorder	0.5	0.5	0.00	0.5	0.5	0.00
Chronic liver disease	1.9	2.6	-0.04	2.3	2.4	-0.01
Chronic obstructive lung disease	13.2	10.6	0.08	11.4	10.9	0.01
Crohn's disease	0.2	0.2	0.00	0.2	0.2	0.00
Dementia	2.4	2.3	0.01	2.3	2.3	0.00
Depressive disorder	16.4	16.9	-0.01	17.0	16.8	0.00
Diabetes mellitus	42.2	32.0	0.21	34.8	34.1	0.01
Gastrointestinal hemorrhage	1.0	1.0	0.00	1.0	1.0	0.00
Human immunodeficiency virus infection	0.4	0.6	-0.02	0.5	0.5	0.00
Hyperlipidemia	52.3	47.5	0.10	48.9	48.4	0.01
Hypertensive disorder	71.7	73.9	-0.05	73.9	73.6	0.00
Obesity	15.9	13.9	0.06	14.6	14.3	0.01

(Continued)	Before stratification			After stratification		
Characteristic	T (%)	C (%)	StdDiff	T (%)	C (%)	StdDiff
Osteoarthritis	16.0	16.1	0.00	16.3	16.1	0.00
Pneumonia	1.7	1.3	0.03	1.4	1.3	0.01
Psoriasis	1.2	1.1	0.01	1.1	1.1	0.00
Renal impairment	13.2	12.1	0.03	13.2	12.3	0.03
Rheumatoid arthritis	0.9	0.7	0.02	0.8	0.8	0.00
Schizophrenia	0.6	1.0	-0.05	0.9	0.9	-0.01
Ulcerative colitis	0.3	0.3	0.01	0.3	0.3	0.00
Urinary tract infectious disease	1.8	1.8	0.00	1.9	1.8	0.00
Viral hepatitis C	1.0	1.8	-0.07	1.5	1.7	-0.01
Visual system disorder	32.5	30.9	0.04	31.5	31.2	0.01
Medical history: Cardiovascular disease						
Atrial fibrillation	13.3	6.3	0.24	8.4	7.2	0.05
Cerebrovascular disease	3.6	3.4	0.01	3.5	3.4	0.01
Coronary arteriosclerosis	22.0	11.9	0.27	14.8	13.4	0.04
Heart disease	40.1	23.8	0.36	28.5	26.2	0.05
Heart failure	13.4	3.9	0.34	7.7	4.6	0.13
Ischemic heart disease	8.4	4.4	0.16	5.6	4.9	0.03
Peripheral vascular disease	5.2	4.5	0.03	4.8	4.6	0.01
Pulmonary embolism	0.6	0.5	0.02	0.5	0.5	0.01
Venous thrombosis	1.2	1.1	0.01	1.1	1.1	0.00
Medical history: Neoplasms						
Hematologic neoplasm	1.3	1.2	0.01	1.2	1.2	0.00
Malignant lymphoma	0.8	0.6	0.02	0.7	0.7	0.00
Malignant neoplasm of anorectum	0.1	0.1	-0.01	0.1	0.1	0.00
Malignant neoplastic disease	9.5	9.9	-0.01	9.9	9.8	0.00
Malignant tumor of breast	0.1	0.1	0.00	0.1	0.1	0.00
Malignant tumor of lung	0.5	0.5	0.00	0.5	0.5	0.00
Malignant tumor of urinary bladder	0.6	0.6	0.00	0.6	0.6	0.00
Primary malignant neoplasm of prostate	3.5	4.0	-0.03	3.9	3.9	0.00
Medication use						
Antibacterials for systemic use	20.2	19.1	0.03	19.8	19.1	0.02
Antidepressants	32.7	32.5	0.00	32.7	32.6	0.00
Antiepileptics	25.2	24.5	0.02	24.9	24.6	0.01
Antiinflammatory and antirheumatic products	26.6	31.2	-0.10	30.4	30.4	0.00
Antithrombotic agents	42.6	32.4	0.21	35.3	34.0	0.03
Drugs for acid related disorders	43.5	39.2	0.09	40.4	40.0	0.01
Drugs for obstructive airway diseases	35.1	30.1	0.11	31.3	30.9	0.01
Drugs used in diabetes	42.9	32.2	0.22	34.8	34.5	0.00
Immunosuppressants	3.4	3.0	0.03	3.1	3.1	0.00
Lipid modifying agents	73.3	66.0	0.16	67.8	67.4	0.01
Opioids	10.9	11.6	-0.02	11.6	11.4	0.01
Psycholeptics	20.0	20.7	-0.02	20.9	20.5	0.01
Psychostimulants, agents used for adhd and nootropics	1.2	1.2	0.00	1.2	1.2	0.00

Supplementary Table 41: Baseline patient characteristcs for ARB mono (T) and CCB mono (C) prevalent-users in the VA-OMOP data source. We report proportion of initiators satisfying selected base-line characteristics and the standardized difference of population proportions (StdDiff) before and after stratification. Less extreme StdDiffs through stratification suggest improved balance between patient cohorts through propensity score adjustment.

	Before stratification			After stratification		
Characteristic	T (%)	C (%)	StdDiff	T (%)	C (%)	StdDiff
Age group						
25-29	0.1	0.2	-0.02	0.2	0.2	0.00
30-34	0.6	0.9	-0.03	0.7	0.7	0.00
35-39	1.5	1.9	-0.03	1.8	1.7	0.00
40-44	2.3	2.4	-0.01	2.4	2.4	0.00
45-49	4.1	4.0	0.00	4.1	4.0	0.00
50-54	6.7	6.4	0.01	6.5	6.5	0.00
55-59	8.7	9.5	-0.03	9.2	9.1	0.00
60-64	10.9	13.5	-0.08	12.4	12.4	0.00
65-69	13.6	14.8	-0.03	14.2	14.3	0.00
70-74	25.7	22.3	0.08	23.4	23.5	0.00
75-79	11.7	9.8	0.06	10.6	10.6	0.00
80-84	6.4	5.6	0.03	6.1	6.1	0.00
85-89	5.1	5.0	0.00	5.1	5.2	0.00
90-94	2.0	2.5	-0.03	2.4	2.4	0.00
95-99	0.5	0.9	-0.05	0.8	0.8	0.00
Gender: female	6.5	7.3	-0.03	7.1	6.9	0.01
Race						
race = Asian	1.9	1.0	0.08	1.3	1.3	0.00
race = Black or African American	13.7	33.1	-0.47	25.6	25.8	0.00
race = White	75.9	58.7	0.37	65.5	65.2	0.01
race = Unknown	6.7	5.8	0.03	6.1	6.1	0.00
race = Native Hawaiian or Other Pacific Islander	1.1	0.8	0.03	0.8	0.9	0.00
race = American Indian or Alaska Native	0.7	0.6	0.02	0.6	0.7	0.00
Ethnicity						
ethnicity = Hispanic or Latino	7.3	5.4	0.08	6.1	6.0	0.00
ethnicity = Not Hispanic or Latino	90.0	92.2	-0.08	91.3	91.4	0.00
Medical history: General						
Acute respiratory disease	4.4	5.0	-0.02	4.7	4.7	0.00
Attention deficit hyperactivity disorder	0.7	0.6	0.01	0.6	0.6	0.00
Chronic liver disease	1.3	2.8	-0.10	1.8	2.2	-0.03
Chronic obstructive lung disease	7.9	9.9	-0.07	9.2	9.3	0.00
Crohn’s disease	0.2	0.2	-0.01	0.2	0.2	0.00
Dementia	1.9	3.0	-0.07	2.6	2.6	0.00
Depressive disorder	15.9	17.6	-0.04	16.9	17.0	0.00
Diabetes mellitus	37.0	16.0	0.49	25.0	23.2	0.04
Gastrointestinal hemorrhage	0.6	0.9	-0.04	0.7	0.8	-0.01
Human immunodeficiency virus infection	0.4	0.8	-0.05	0.6	0.6	0.00
Hyperlipidemia	47.7	39.1	0.17	42.7	42.1	0.01
Hypertensive disorder	68.9	71.0	-0.04	70.5	70.3	0.00

(Continued)	Before stratification			After stratification		
Characteristic	T (%)	C (%)	StdDiff	T (%)	C (%)	StdDiff
Lesion of liver	1.1	1.8	-0.05	1.4	1.5	-0.01
Obesity	13.2	9.0	0.13	10.9	10.4	0.02
Osteoarthritis	15.1	15.2	0.00	15.3	15.2	0.00
Pneumonia	0.7	1.1	-0.04	0.9	1.0	-0.01
Psoriasis	1.1	1.0	0.02	1.1	1.0	0.00
Renal impairment	7.2	8.5	-0.05	8.2	8.2	0.00
Rheumatoid arthritis	0.8	0.8	0.01	0.8	0.8	0.01
Schizophrenia	0.5	1.3	-0.08	0.9	1.1	-0.02
Urinary tract infectious disease	1.2	1.8	-0.04	1.6	1.6	0.00
Viral hepatitis C	0.8	2.2	-0.12	1.3	1.7	-0.03
Visual system disorder	29.0	27.8	0.03	28.3	28.1	0.00
Medical history: Cardiovascular disease						
Atrial fibrillation	3.9	2.5	0.08	3.2	3.0	0.01
Coronary arteriosclerosis	7.9	5.1	0.11	6.4	6.1	0.01
Heart disease	16.5	12.9	0.10	14.8	14.3	0.01
Heart failure	1.8	0.9	0.08	1.4	1.0	0.04
Peripheral vascular disease	2.5	2.8	-0.02	2.6	2.7	0.00
Pulmonary embolism	0.4	0.4	0.00	0.4	0.4	0.00
Venous thrombosis	0.8	1.0	-0.03	0.9	1.0	0.00
Medical history: Neoplasms						
Hematologic neoplasm	1.1	1.3	-0.02	1.2	1.2	0.00
Malignant lymphoma	0.7	0.7	0.00	0.7	0.7	0.01
Malignant neoplasm of anorectum	0.1	0.1	-0.02	0.1	0.1	-0.01
Malignant neoplastic disease	8.2	10.5	-0.08	9.6	9.7	0.00
Malignant tumor of breast	0.1	0.1	0.00	0.1	0.1	0.01
Malignant tumor of colon	0.3	0.4	-0.03	0.3	0.4	0.00
Malignant tumor of lung	0.3	0.6	-0.04	0.5	0.5	0.00
Malignant tumor of urinary bladder	0.5	0.6	-0.02	0.5	0.5	0.00
Primary malignant neoplasm of prostate	3.1	4.3	-0.06	3.8	3.9	-0.01
Medication use						
Antibacterials for systemic use	15.5	17.9	-0.06	16.7	17.0	-0.01
Antidepressants	30.8	31.3	-0.01	31.3	31.1	0.00
Antiepileptics	21.6	21.0	0.02	21.5	21.2	0.01
Antiinflammatory and antirheumatic products	28.6	31.5	-0.06	30.3	30.4	0.00
Antineoplastic agents	2.8	2.9	-0.01	2.8	2.8	0.00
Antipsoriatics	0.7	0.9	-0.02	0.8	0.8	0.00
Antithrombotic agents	23.5	21.6	0.04	22.5	22.3	0.00
Drugs for acid related disorders	35.5	33.9	0.03	34.6	34.7	0.00
Drugs for obstructive airway diseases	28.6	28.3	0.01	28.5	28.4	0.00
Drugs used in diabetes	37.6	14.2	0.55	23.7	22.3	0.03
Immunosuppressants	3.0	3.1	0.00	3.2	3.1	0.01
Lipid modifying agents	63.0	49.6	0.27	54.7	54.5	0.00
Opioids	8.2	9.8	-0.06	9.2	9.3	0.00
Psycholeptics	17.8	21.0	-0.08	19.9	20.0	0.00

(Continued)

Characteristic	Before stratification			After stratification		
	T (%)	C (%)	StdDiff	T (%)	C (%)	StdDiff
Psychostimulants, agents used for adhd and nootropics	1.4	1.3	0.01	1.4	1.3	0.01

Supplementary Table 42: Baseline patient characteristcs for ARB +combo (T) and CCB +combo (C) prevalent-users in the VA-OMOP data source. We report proportion of initiators satisfying selected base-line characteristics and the standardized difference of population proportions (StdDiff) before and after stratification. Less extreme StdDiffs through stratification suggest improved balance between patient cohorts through propensity score adjustment.

	Before stratification			After stratification		
Characteristic	T (%)	C (%)	StdDiff	T (%)	C (%)	StdDiff
Age group						
25-29	0.1	0.1	-0.01	0.1	0.1	0.00
30-34	0.3	0.4	-0.01	0.4	0.4	0.00
35-39	0.9	1.0	-0.01	1.0	1.0	0.00
40-44	1.5	1.5	0.00	1.5	1.5	0.00
45-49	3.0	2.9	0.01	2.9	2.9	0.00
55-59	7.5	8.0	-0.02	7.8	7.8	0.00
60-64	10.5	12.3	-0.06	11.7	11.7	0.00
65-69	14.4	15.7	-0.04	15.1	15.3	0.00
70-74	28.6	27.0	0.04	27.5	27.6	0.00
75-79	13.2	11.7	0.05	12.2	12.2	0.00
80-84	6.9	6.2	0.03	6.5	6.4	0.00
85-89	5.2	5.0	0.01	5.1	5.1	0.00
90-94	2.1	2.2	-0.01	2.2	2.2	0.00
95-99	0.5	0.7	-0.02	0.6	0.7	0.00
Gender: female	6.2	5.2	0.04	5.5	5.6	0.00
Race						
race = Black or African American	16.6	29.6	-0.31	25.3	24.9	0.01
race = White	74.3	62.6	0.25	66.6	66.8	0.00
race = Unknown	6.1	5.6	0.02	5.7	5.9	0.00
race = Native Hawaiian or Other Pacific Islander	0.9	0.8	0.02	0.8	0.8	0.00
race = American Indian or Alaska Native	0.7	0.6	0.01	0.6	0.6	0.00
Ethnicity						
ethnicity = Hispanic or Latino	6.0	5.2	0.03	5.4	5.5	0.00
ethnicity = Not Hispanic or Latino	91.5	92.4	-0.03	92.2	92.0	0.01
Medical history: General						
Acute respiratory disease	6.2	5.7	0.02	5.9	5.8	0.00
Attention deficit hyperactivity disorder	0.5	0.4	0.02	0.5	0.4	0.00
Chronic liver disease	1.8	2.9	-0.07	2.3	2.6	-0.02
Chronic obstructive lung disease	12.2	11.5	0.02	11.8	11.5	0.01
Crohn’s disease	0.2	0.2	0.00	0.2	0.2	0.00
Dementia	2.1	2.9	-0.05	2.5	2.6	-0.01
Depressive disorder	16.4	17.3	-0.02	17.0	17.0	0.00
Diabetes mellitus	41.6	32.9	0.18	36.4	36.1	0.01
Gastroesophageal reflux disease	16.4	15.3	0.03	15.6	15.7	0.00
Gastrointestinal hemorrhage	0.9	1.1	-0.01	1.0	1.0	0.00
Hyperlipidemia	51.8	47.9	0.08	49.6	49.2	0.01
Hypertensive disorder	72.8	75.3	-0.06	74.5	74.7	0.00
Lesion of liver	1.6	2.1	-0.04	1.8	1.9	-0.01
Obesity	16.7	13.1	0.10	14.7	14.3	0.01

(Continued)	Before stratification			After stratification		
Characteristic	T (%)	C (%)	StdDiff	T (%)	C (%)	StdDiff
Osteoarthritis	16.4	16.1	0.01	16.3	16.2	0.00
Pneumonia	1.5	1.6	-0.01	1.5	1.5	0.00
Psoriasis	1.2	1.0	0.02	1.1	1.1	0.00
Renal impairment	12.3	14.9	-0.08	13.9	14.0	0.00
Rheumatoid arthritis	0.9	0.8	0.01	0.8	0.8	0.00
Schizophrenia	0.6	1.2	-0.07	0.9	1.0	-0.01
Ulcerative colitis	0.3	0.3	0.01	0.3	0.3	0.00
Urinary tract infectious disease	1.7	2.1	-0.03	1.8	2.0	-0.01
Viral hepatitis C	1.0	2.1	-0.09	1.5	1.8	-0.02
Visual system disorder	32.2	31.9	0.01	32.2	32.1	0.00
Medical history: Cardiovascular disease						
Atrial fibrillation	11.6	6.6	0.17	8.8	7.8	0.04
Cerebrovascular disease	3.4	4.0	-0.03	3.8	3.8	0.00
Coronary arteriosclerosis	19.4	13.6	0.16	16.1	15.1	0.03
Heart disease	36.0	26.4	0.21	30.6	28.9	0.04
Heart failure	10.8	4.7	0.23	7.7	5.5	0.09
Ischemic heart disease	7.3	5.2	0.09	6.2	5.6	0.02
Peripheral vascular disease	4.8	5.3	-0.02	5.1	5.1	0.00
Pulmonary embolism	0.6	0.5	0.01	0.6	0.5	0.00
Venous thrombosis	1.1	1.2	-0.01	1.2	1.2	0.00
Medical history: Neoplasms						
Hematologic neoplasm	1.2	1.3	-0.01	1.3	1.2	0.00
Malignant lymphoma	0.8	0.7	0.01	0.7	0.7	0.00
Malignant neoplasm of anorectum	0.1	0.1	-0.01	0.1	0.1	0.00
Malignant neoplastic disease	9.2	10.8	-0.05	10.2	10.3	0.00
Malignant tumor of breast	0.1	0.1	0.01	0.1	0.1	0.00
Malignant tumor of colon	0.3	0.4	-0.02	0.4	0.4	0.00
Malignant tumor of lung	0.4	0.6	-0.03	0.5	0.6	-0.01
Primary malignant neoplasm of prostate	3.5	4.3	-0.04	4.1	4.0	0.00
Medication use						
Antibacterials for systemic use	19.6	20.1	-0.01	19.8	19.8	0.00
Antidepressants	32.6	32.9	0.00	32.8	32.9	0.00
Antiinflammatory and antirheumatic products	28.2	30.2	-0.04	29.5	29.7	0.00
Antineoplastic agents	3.0	3.0	0.00	3.0	3.0	0.00
Antipsoriatics	1.2	1.6	-0.04	1.4	1.5	-0.01
Antithrombotic agents	39.7	35.6	0.08	37.4	36.5	0.02
Diuretics	0.1	0.1	0.00	0.1	0.1	0.00
Drugs for obstructive airway diseases	34.5	30.7	0.08	32.2	31.9	0.01
Drugs used in diabetes	42.6	32.8	0.20	36.5	36.5	0.00
Lipid modifying agents	72.5	66.7	0.13	69.0	68.7	0.01
Opioids	10.7	12.1	-0.05	11.5	11.6	0.00
Psycholeptics	19.6	21.6	-0.05	20.8	21.0	-0.01

Supplementary Table 43: Baseline patient characteristcs for ARB mono (T) and THZ mono (C) prevalent-users in the VA-OMOP data source. We report proportion of initiators satisfying selected base-line characteristics and the standardized difference of population proportions (StdDiff) before and after stratification. Less extreme StdDiffs through stratification suggest improved balance between patient cohorts through propensity score adjustment.

	Before stratification			After stratification		
Characteristic	T (%)	C (%)	StdDiff	T (%)	C (%)	StdDiff
Age group						
25-29	0.1	0.3	-0.04	0.2	0.2	-0.01
35-39	1.5	2.6	-0.08	1.9	1.9	0.00
40-44	2.3	3.5	-0.07	2.8	2.7	0.00
45-49	4.1	5.4	-0.06	4.7	4.6	0.01
50-54	6.7	8.3	-0.06	7.4	7.2	0.01
55-59	8.7	10.9	-0.07	9.6	9.5	0.00
60-64	10.9	13.1	-0.07	11.9	11.8	0.00
65-69	13.6	14.3	-0.02	13.9	14.1	0.00
70-74	25.7	22.0	0.09	23.9	24.0	0.00
75-79	11.7	9.0	0.09	10.6	10.7	0.00
80-84	6.4	4.4	0.09	5.6	5.7	0.00
85-89	5.1	3.2	0.09	4.3	4.4	-0.01
90-94	2.0	1.3	0.05	1.8	1.8	0.00
95-99	0.5	0.4	0.01	0.5	0.5	0.00
Gender: female	6.5	12.9	-0.22	8.9	9.0	-0.01
Race						
race = Asian	1.9	0.7	0.11	1.4	1.2	0.02
race = Black or African American	13.7	28.8	-0.38	20.2	19.5	0.02
race = White	75.9	63.3	0.28	70.5	71.4	-0.02
race = Unknown	6.7	5.8	0.04	6.3	6.2	0.00
race = Native Hawaiian or Other Pacific Islander	1.1	0.8	0.03	0.9	1.0	-0.01
race = American Indian or Alaska Native	0.7	0.6	0.02	0.7	0.6	0.01
Ethnicity						
ethnicity = Hispanic or Latino	7.3	5.1	0.09	6.4	6.3	0.00
ethnicity = Not Hispanic or Latino	90.0	92.5	-0.09	91.1	91.2	0.00
Medical history: General						
Acute respiratory disease	4.4	4.9	-0.02	4.5	4.7	-0.01
Attention deficit hyperactivity disorder	0.7	0.7	-0.01	0.7	0.7	0.00
Chronic liver disease	1.3	1.9	-0.04	1.5	1.6	-0.01
Chronic obstructive lung disease	7.9	8.0	0.00	8.0	8.2	-0.01
Crohn's disease	0.2	0.2	0.00	0.2	0.2	0.00
Dementia	1.9	1.3	0.05	1.7	1.7	0.00
Depressive disorder	15.9	17.6	-0.04	16.4	16.3	0.00
Diabetes mellitus	37.0	15.5	0.50	28.3	28.4	0.00
Gastroesophageal reflux disease	14.8	14.0	0.02	14.5	14.6	0.00
Gastrointestinal hemorrhage	0.6	0.6	0.00	0.6	0.6	0.00
Human immunodeficiency virus infection	0.4	0.7	-0.05	0.5	0.5	-0.01
Hyperlipidemia	47.7	39.3	0.17	44.1	44.0	0.00
Hypertensive disorder	68.9	68.2	0.01	68.8	68.8	0.00

(Continued)	Before stratification			After stratification		
Characteristic	T (%)	C (%)	StdDiff	T (%)	C (%)	StdDiff
Obesity	13.2	13.0	0.01	13.2	13.0	0.00
Osteoarthritis	15.1	16.1	-0.03	15.6	15.8	0.00
Pneumonia	0.7	0.6	0.01	0.7	0.7	0.00
Psoriasis	1.1	1.0	0.01	1.1	1.1	0.00
Renal impairment	7.2	4.1	0.13	6.0	6.0	0.00
Rheumatoid arthritis	0.8	0.8	0.01	0.8	0.8	0.01
Schizophrenia	0.5	0.9	-0.04	0.6	0.7	-0.01
Ulcerative colitis	0.3	0.3	0.00	0.3	0.3	0.00
Urinary tract infectious disease	1.2	1.1	0.01	1.2	1.2	0.00
Viral hepatitis C	0.8	1.4	-0.06	1.0	1.0	-0.01
Medical history: Cardiovascular disease						
Atrial fibrillation	3.9	1.9	0.12	3.2	3.1	0.00
Cerebrovascular disease	2.3	1.5	0.06	2.0	2.0	0.00
Coronary arteriosclerosis	7.9	3.2	0.21	6.1	5.8	0.01
Heart disease	16.5	9.6	0.21	13.9	13.5	0.01
Heart failure	1.8	0.6	0.11	1.4	0.9	0.04
Ischemic heart disease	2.2	1.0	0.10	1.7	1.6	0.01
Peripheral vascular disease	2.5	1.9	0.04	2.2	2.3	0.00
Pulmonary embolism	0.4	0.3	0.01	0.4	0.4	0.00
Venous thrombosis	0.8	0.7	0.01	0.8	0.7	0.01
Medical history: Neoplasms						
Hematologic neoplasm	1.1	1.0	0.01	1.0	1.1	0.00
Malignant lymphoma	0.7	0.6	0.01	0.6	0.6	0.00
Malignant neoplasm of anorectum	0.1	0.1	0.00	0.1	0.1	0.00
Malignant neoplastic disease	8.2	7.9	0.01	8.0	8.2	-0.01
Malignant tumor of breast	0.1	0.2	-0.01	0.1	0.1	0.00
Malignant tumor of colon	0.3	0.2	0.00	0.3	0.2	0.01
Malignant tumor of lung	0.3	0.4	-0.01	0.3	0.4	-0.01
Malignant tumor of urinary bladder	0.5	0.4	0.01	0.4	0.4	0.00
Primary malignant neoplasm of prostate	3.1	3.3	-0.01	3.1	3.3	-0.01
Medication use						
Antibacterials for systemic use	15.5	16.3	-0.02	15.7	15.8	0.00
Antidepressants	30.8	31.7	-0.02	31.1	31.1	0.00
Antiepileptics	21.6	20.9	0.02	21.4	21.5	0.00
Antiinflammatory and antirheumatic products	28.6	35.0	-0.14	31.2	31.2	0.00
Antineoplastic agents	2.8	2.9	-0.01	2.8	2.9	-0.01
Antipsoriatics	0.7	0.6	0.01	0.7	0.7	0.00
Antithrombotic agents	23.5	16.3	0.18	20.5	20.4	0.00
Drugs for acid related disorders	35.5	32.8	0.06	34.3	34.7	-0.01
Drugs for obstructive airway diseases	28.6	28.7	0.00	28.5	28.7	0.00
Immunosuppressants	3.0	2.7	0.02	2.9	2.8	0.00
Lipid modifying agents	63.0	49.2	0.28	56.7	56.7	0.00
Opioids	8.2	9.4	-0.04	8.7	8.9	0.00
Psycholeptics	17.8	19.5	-0.04	18.4	18.6	0.00

(Continued)	Before stratification			After stratification		
Characteristic	T (%)	C (%)	StdDiff	T (%)	C (%)	StdDiff
Psychostimulants, agents used for adhd and nootropics	1.4	1.7	-0.02	1.6	1.6	0.00

Supplementary Table 44: Baseline patient characteristcs for ARB +combo (T) and THZ +combo (C) prevalent-users in the VA-OMOP data source. We report proportion of initiators satisfying selected base-line characteristics and the standardized difference of population proportions (StdDiff) before and after stratification. Less extreme StdDiffs through stratification suggest improved balance between patient cohorts through propensity score adjustment.

	Before stratification			After stratification		
Characteristic	T (%)	C (%)	StdDiff	T (%)	C (%)	StdDiff
Age group						
25-29	0.1	0.1	-0.02	0.1	0.1	0.00
30-34	0.3	0.5	-0.04	0.4	0.4	0.00
40-44	1.3	2.0	-0.06	1.8	1.8	0.00
45-49	2.6	3.8	-0.07	3.4	3.3	0.00
50-54	4.6	6.5	-0.08	5.9	5.8	0.01
55-59	7.0	9.3	-0.08	8.5	8.4	0.00
70-74	28.8	27.1	0.04	27.6	27.8	-0.01
75-79	13.7	10.7	0.09	11.8	11.8	0.00
80-84	7.4	5.0	0.10	5.9	5.9	0.00
85-89	5.8	3.4	0.12	4.3	4.3	0.00
90-94	2.4	1.3	0.08	1.7	1.7	0.00
Gender: female	5.3	7.2	-0.08	6.3	6.5	-0.01
Race						
race = Asian	1.5	0.6	0.09	0.9	0.9	0.00
race = Black or African American	18.0	26.1	-0.20	23.0	22.7	0.01
race = White	72.8	66.3	0.14	68.9	69.1	0.00
race = Unknown	6.1	5.7	0.02	5.8	5.9	0.00
race = Native Hawaiian or Other Pacific Islander	1.0	0.7	0.03	0.8	0.8	0.00
race = American Indian or Alaska Native	0.7	0.6	0.01	0.6	0.6	0.00
Ethnicity						
ethnicity = Hispanic or Latino	6.0	4.9	0.05	5.4	5.3	0.00
ethnicity = Not Hispanic or Latino	91.5	92.7	-0.05	92.3	92.1	0.00
Medical history: General						
Acute respiratory disease	6.5	5.0	0.06	5.7	5.4	0.01
Attention deficit hyperactivity disorder	0.5	0.5	-0.01	0.5	0.5	0.00
Chronic liver disease	2.1	2.2	-0.01	2.1	2.1	0.00
Chronic obstructive lung disease	12.9	9.2	0.12	10.8	10.3	0.02
Crohn's disease	0.2	0.2	0.00	0.2	0.2	0.00
Dementia	2.6	1.4	0.08	1.9	1.8	0.01
Diabetes mellitus	43.1	32.7	0.22	37.3	37.2	0.00
Gastroesophageal reflux disease	16.6	14.4	0.06	15.3	15.2	0.00
Gastrointestinal hemorrhage	1.0	0.8	0.03	0.9	0.8	0.01
Human immunodeficiency virus infection	0.4	0.5	-0.02	0.5	0.5	0.00
Hyperlipidemia	52.5	47.6	0.10	49.7	49.5	0.00
Hypertensive disorder	73.5	73.1	0.01	73.5	73.6	0.00
Lesion of liver	1.7	1.6	0.01	1.6	1.6	0.00
Obesity	16.1	15.6	0.01	16.0	15.8	0.00
Pneumonia	1.7	0.9	0.07	1.3	1.1	0.01
Psoriasis	1.2	1.1	0.01	1.1	1.1	0.00

(Continued)	Before stratification			After stratification		
Characteristic	T (%)	C (%)	StdDiff	T (%)	C (%)	StdDiff
Renal impairment	15.2	8.7	0.20	11.8	11.0	0.03
Rheumatoid arthritis	0.9	0.7	0.02	0.8	0.8	0.00
Schizophrenia	0.6	0.8	-0.02	0.8	0.8	0.00
Ulcerative colitis	0.3	0.3	0.01	0.3	0.3	0.00
Urinary tract infectious disease	1.9	1.4	0.04	1.6	1.6	0.00
Visual system disorder	33.6	30.1	0.07	31.6	31.4	0.00
Medical history: Cardiovascular disease						
Atrial fibrillation	12.1	5.4	0.24	8.3	7.4	0.04
Cerebrovascular disease	4.0	2.6	0.07	3.3	3.1	0.01
Coronary arteriosclerosis	21.4	9.3	0.34	14.5	13.1	0.04
Heart disease	38.8	20.1	0.42	28.1	26.1	0.04
Heart failure	12.1	2.6	0.37	7.2	4.4	0.12
Ischemic heart disease	8.2	3.2	0.21	5.5	4.6	0.04
Peripheral vascular disease	5.6	3.7	0.09	4.5	4.3	0.01
Pulmonary embolism	0.6	0.4	0.03	0.5	0.5	0.00
Venous thrombosis	1.2	0.9	0.03	1.0	1.0	0.00
Medical history: Neoplasms						
Hematologic neoplasm	1.3	1.0	0.03	1.1	1.1	0.00
Malignant lymphoma	0.8	0.6	0.03	0.7	0.6	0.00
Malignant neoplasm of anorectum	0.1	0.1	0.00	0.1	0.1	0.00
Malignant neoplastic disease	9.9	8.7	0.04	9.2	9.1	0.00
Malignant tumor of breast	0.1	0.1	0.00	0.1	0.1	0.00
Malignant tumor of colon	0.3	0.3	0.00	0.3	0.3	0.00
Malignant tumor of lung	0.5	0.4	0.02	0.4	0.4	0.00
Malignant tumor of urinary bladder	0.6	0.5	0.02	0.5	0.5	0.00
Medication use						
Antibacterials for systemic use	20.7	17.7	0.08	19.0	18.7	0.01
Antidepressants	33.2	32.0	0.03	32.4	32.6	0.00
Antiinflammatory and antirheumatic products	27.3	33.0	-0.12	31.0	31.0	0.00
Antineoplastic agents	3.1	2.8	0.02	2.9	2.9	0.00
Antipsoriatics	1.6	0.9	0.06	1.2	1.1	0.01
Antithrombotic agents	43.0	28.7	0.30	34.8	33.6	0.03
Drugs for acid related disorders	44.4	37.6	0.14	40.3	40.2	0.00
Drugs for obstructive airway diseases	35.3	29.1	0.13	31.7	31.2	0.01
Drugs used in diabetes	43.9	33.6	0.21	38.1	38.2	0.00
Immunosuppressants	3.5	2.6	0.06	2.9	2.9	0.00
Lipid modifying agents	73.9	66.4	0.17	69.3	69.3	0.00
Opioids	11.3	10.9	0.01	11.1	11.0	0.00
Psycholeptics	20.4	19.5	0.02	19.9	19.8	0.00
Psychostimulants, agents used for adhd and nootropics	1.2	1.3	-0.01	1.3	1.3	0.00

CUIMC

Supplementary Table 45: Baseline patient characteristcs for ACEI/ARB mono (T) and CCB/THZ mono (C) prevalent-users in the CUIMC data source. We report proportion of initiators satisfying selected base-line characteristics and the standardized difference of population proportions (StdDiff) before and after stratification. Less extreme StdDiffs through stratification suggest improved balance between patient cohorts through propensity score adjustment.

	Before stratification			After stratification		
Characteristic	T (%)	C (%)	StdDiff	T (%)	C (%)	StdDiff
Age group						
15-19	0.1	0.2	-0.02	0.2	0.2	0.00
20-24	0.6	0.5	0.01	0.7	0.4	0.04
25-29	0.7	1.1	-0.04	0.8	0.8	0.00
30-34	1.1	1.5	-0.03	1.3	1.2	0.01
35-39	1.8	2.3	-0.04	2.0	1.9	0.01
40-44	2.8	3.5	-0.04	2.8	3.1	-0.02
45-49	4.7	5.3	-0.03	5.1	4.9	0.01
50-54	7.1	7.0	0.00	7.3	6.6	0.03
55-59	9.9	9.0	0.03	9.7	9.2	0.02
60-64	13.1	11.4	0.05	12.5	12.3	0.01
65-69	14.9	13.4	0.04	14.4	13.7	0.02
70-74	15.4	15.0	0.01	15.1	15.8	-0.02
75-79	11.8	11.9	0.00	11.8	12.4	-0.02
80-84	8.3	8.4	0.00	8.1	8.6	-0.02
85-89	4.7	5.5	-0.03	5.0	5.3	-0.01
90-94	2.0	3.0	-0.06	2.3	2.7	-0.03
95-99	0.8	0.8	0.00	1.0	0.8	0.02
100-104	<0.1	0.3	-0.04	<0.1	0.2	-0.04
Gender: female	50.1	58.7	-0.17	55.2	53.9	0.03
Race						
race = Asian	1.7	1.4	0.02	1.6	1.4	0.02
race = Black or African American	5.9	10.8	-0.18	8.3	8.3	0.00
race = Other Race	0.2	<0.1	0.03	0.1	<0.1	0.04
race = White	36.4	31.0	0.11	33.3	33.9	-0.01
race = Unknown	1.9	2.3	-0.03	1.9	2.3	-0.02
race = Native Hawaiian or Other Pacific Islander	<0.1	0.0		<0.1	0.0	
race = American Indian or Alaska Native	0.2	<0.1	0.04	0.2	<0.1	0.04
race = Asian Indian	0.2	0.2	0.00	0.2	0.3	-0.02
race = Bangladeshi	0.0	<0.1		0.0	<0.1	
race = Chinese	<0.1	<0.1	0.03	<0.1	<0.1	0.03
race = Filipino	<0.1	<0.1	-0.01	<0.1	<0.1	-0.01
race = Japanese	<0.1	0.0		<0.1	0.0	
race = Korean	<0.1	<0.1	0.00	<0.1	<0.1	0.01
race = Laotian	0.4	0.5	-0.02	0.5	0.5	-0.01
race = Pakistani	0.0	<0.1		0.0	<0.1	
race = Thai	<0.1	<0.1	0.00	<0.1	<0.1	0.00
race = Vietnamese	<0.1	<0.1	0.00	<0.1	<0.1	0.00

(Continued)	Before stratification			After stratification		
Characteristic	T (%)	C (%)	StdDiff	T (%)	C (%)	StdDiff
race = Micronesian	<0.1	0.0		<0.1	0.0	
race = Other Pacific Islander	0.8	0.6	0.02	0.9	0.5	0.04
Ethnicity						
ethnicity = Hispanic or Latino	11.5	13.9	-0.07	12.6	13.1	-0.01
ethnicity = Not Hispanic or Latino	35.3	34.4	0.02	34.3	35.2	-0.02
Medical history: General						
Acute respiratory disease	3.6	4.0	-0.02	3.8	3.8	0.00
Attention deficit hyperactivity disorder	0.2	0.2	-0.02	0.2	0.2	-0.01
Chronic liver disease	0.7	1.2	-0.05	0.7	1.0	-0.04
Chronic obstructive lung disease	2.7	3.1	-0.02	2.9	2.8	0.00
Crohn’s disease	0.3	0.2	0.01	0.3	0.2	0.03
Dementia	1.7	2.3	-0.04	1.9	2.1	-0.02
Depressive disorder	4.9	6.0	-0.05	5.6	5.5	0.01
Diabetes mellitus	21.5	13.2	0.22	16.7	17.0	-0.01
Gastroesophageal reflux disease	7.1	7.1	0.00	7.3	6.8	0.02
Gastrointestinal hemorrhage	0.8	1.1	-0.03	0.8	0.8	0.00
Human immunodeficiency virus infection	1.9	1.7	0.02	2.1	1.5	0.05
Hyperlipidemia	38.3	33.1	0.11	35.5	35.6	0.00
Hypertensive disorder	61.1	69.0	-0.17	65.7	64.3	0.03
Lesion of liver	0.9	1.5	-0.06	1.0	1.4	-0.04
Obesity	9.2	9.8	-0.02	9.3	9.7	-0.01
Osteoarthritis	10.9	12.2	-0.04	11.8	11.8	0.00
Pneumonia	1.1	1.9	-0.06	1.2	1.6	-0.03
Psoriasis	0.7	0.5	0.03	0.6	0.5	0.02
Renal impairment	6.5	9.7	-0.12	7.8	8.4	-0.02
Rheumatoid arthritis	0.8	0.8	0.00	0.9	0.8	0.02
Schizophrenia	0.2	0.3	-0.02	0.2	0.2	0.00
Ulcerative colitis	0.2	0.1	0.01	0.2	0.1	0.02
Urinary tract infectious disease	2.1	2.8	-0.05	2.5	2.5	0.00
Viral hepatitis C	0.4	0.8	-0.05	0.5	0.7	-0.03
Visual system disorder	11.1	10.2	0.03	10.8	10.2	0.02
Medical history: Cardiovascular disease						
Atrial fibrillation	5.3	4.6	0.03	5.0	4.8	0.01
Cerebrovascular disease	5.3	5.0	0.01	5.2	5.0	0.01
Coronary arteriosclerosis	13.0	10.7	0.07	11.8	12.1	-0.01
Heart disease	27.7	25.0	0.06	26.4	26.2	0.00
Heart failure	4.2	2.4	0.10	3.5	2.8	0.04
Ischemic heart disease	3.4	3.3	0.00	3.1	3.6	-0.03
Peripheral vascular disease	3.9	3.2	0.04	3.4	3.3	0.00
Pulmonary embolism	0.3	0.5	-0.02	0.4	0.4	0.00
Venous thrombosis	0.6	1.2	-0.06	0.7	1.0	-0.04
Medical history: Neoplasms						
Hematologic neoplasm	1.8	1.8	0.00	1.8	1.5	0.03
Malignant lymphoma	1.0	1.3	-0.03	1.0	1.2	-0.02
Malignant neoplasm of anorectum	0.2	0.2	0.01	0.2	0.2	0.01

(Continued)	Before stratification			After stratification		
Characteristic	T (%)	C (%)	StdDiff	T (%)	C (%)	StdDiff
Malignant neoplastic disease	9.5	10.8	-0.04	9.9	10.2	-0.01
Malignant tumor of breast	1.7	1.7	0.00	1.9	1.5	0.03
Malignant tumor of colon	0.3	0.5	-0.04	0.3	0.5	-0.03
Malignant tumor of lung	0.5	0.6	-0.02	0.6	0.6	0.00
Malignant tumor of urinary bladder	0.5	0.5	0.00	0.5	0.5	0.01
Primary malignant neoplasm of prostate	1.5	1.8	-0.03	1.4	1.7	-0.03
Medication use						
Antibacterials for systemic use	25.7	27.1	-0.03	26.1	25.7	0.01
Antidepressants	15.2	15.5	-0.01	15.4	15.1	0.01
Antiepileptics	13.3	13.0	0.01	13.4	12.7	0.02
Antiinflammatory and antirheumatic products	17.1	20.5	-0.09	18.6	18.9	-0.01
Antineoplastic agents	3.2	4.1	-0.05	3.6	3.6	0.00
Antipsoriatics	0.6	1.3	-0.08	0.7	1.1	-0.04
Antithrombotic agents	21.9	22.5	-0.02	21.7	22.0	-0.01
Diuretics	<0.1	<0.1	0.00	<0.1	<0.1	0.00
Drugs for acid related disorders	22.6	26.9	-0.10	24.3	24.9	-0.01
Drugs for obstructive airway diseases	14.0	15.1	-0.03	14.5	14.3	0.00
Drugs used in diabetes	22.7	12.8	0.26	17.0	17.8	-0.02
Immunosuppressants	5.4	7.7	-0.09	6.6	6.3	0.01
Lipid modifying agents	43.2	35.0	0.17	38.4	39.4	-0.02
Opioids	10.3	14.4	-0.12	11.6	12.6	-0.03
Psycholeptics	14.3	15.9	-0.05	15.1	15.1	0.00
Psychostimulants, agents used for adhd and nootropics	1.5	1.5	0.00	1.5	1.5	0.00

Supplementary Table 46: Baseline patient characteristcs for ACEI/ARB +combo (T) and CCB/THZ +combo (C) prevalent-users in the CUIMC data source. We report proportion of initiators satisfying selected base-line characteristics and the standardized difference of population proportions (StdDiff) before and after stratification. Less extreme StdDiffs through stratification suggest improved balance between patient cohorts through propensity score adjustment.

	Before stratification			After stratification		
Characteristic	T (%)	C (%)	StdDiff	T (%)	C (%)	StdDiff
Age group						
15-19	0.2	0.2	0.00	0.2	0.1	0.01
20-24	0.4	0.5	-0.01	0.5	0.4	0.02
25-29	0.5	0.8	-0.04	0.6	0.6	-0.01
30-34	0.9	1.5	-0.06	1.0	1.2	-0.02
35-39	1.6	2.3	-0.05	1.7	1.8	0.00
40-44	2.3	3.1	-0.05	2.6	2.7	-0.01
45-49	3.8	4.5	-0.03	4.4	4.0	0.02
50-54	6.0	6.0	0.00	6.2	5.7	0.02
55-59	9.3	8.8	0.02	9.1	8.7	0.01
60-64	12.6	11.1	0.05	11.7	11.7	0.00
65-69	14.4	13.5	0.03	14.0	13.6	0.01
70-74	15.7	14.9	0.02	15.4	15.6	0.00
75-79	12.7	12.6	0.00	12.8	13.1	-0.01
80-84	9.6	9.6	0.00	9.5	9.8	-0.01
85-89	6.2	6.3	0.00	6.2	6.5	-0.01
90-94	3.0	3.3	-0.01	3.1	3.4	-0.01
95-99	0.9	1.0	-0.01	0.9	1.0	0.00
100-104	0.1	0.2	-0.04	0.1	0.2	-0.03
Gender: female	47.1	57.0	-0.20	52.9	51.8	0.02
Race						
race = Asian	1.5	1.4	0.01	1.6	1.3	0.02
race = Black or African American	7.3	11.6	-0.15	9.5	9.3	0.00
race = Other Race	0.1	0.1	0.00	0.1	0.1	0.01
race = White	37.1	31.9	0.11	34.1	34.9	-0.02
race = Unknown	2.1	2.2	-0.01	2.1	2.3	-0.01
race = Native Hawaiian or Other Pacific Islander	0.0	0.0	0.00	<0.1	0.0	0.00
race = American Indian or Alaska Native	0.1	0.1	0.03	0.1	0.0	0.03
race = Asian Indian	0.2	0.3	-0.02	0.2	0.3	-0.04
race = Bangladeshi	0.0	0.0	-0.01	<0.1	0.0	-0.01
race = Chinese	0.1	0.0	0.02	0.1	0.0	0.03
race = Filipino	0.0	0.0	-0.01	<0.1	0.0	-0.01
race = Indonesian	0.0	0.0		<0.1	0.0	
race = Japanese	0.0	0.0	0.00	<0.1	0.0	0.01
race = Korean	0.0	0.0	0.01	<0.1	0.0	0.01
race = Laotian	0.5	0.7	-0.02	0.6	0.6	0.00
race = Pakistani	0.0	0.0	-0.01	<0.1	0.0	-0.01
race = Thai	0.0	0.0	0.00	<0.1	0.0	0.00
race = Vietnamese	0.0	0.0	0.00	<0.1	0.0	0.00
race = Micronesian	0.0	0.0		<0.1	0.0	

(Continued)	Before stratification			After stratification		
Characteristic	T (%)	C (%)	StdDiff	T (%)	C (%)	StdDiff
race = Other Pacific Islander	0.6	0.7	-0.01	0.6	0.6	0.01
Ethnicity						
ethnicity = Hispanic or Latino	12.0	14.0	-0.06	12.8	13.0	-0.01
ethnicity = Not Hispanic or Latino	36.4	35.3	0.02	35.5	36.2	-0.01
Medical history: General						
Acute respiratory disease	4.6	5.9	-0.06	5.1	5.0	0.00
Attention deficit hyperactivity disorder	0.1	0.2	-0.02	0.1	0.2	-0.01
Chronic liver disease	1.2	1.8	-0.05	1.3	1.5	-0.02
Chronic obstructive lung disease	4.4	4.5	0.00	4.4	4.2	0.01
Crohn’s disease	0.3	0.3	0.00	0.3	0.2	0.01
Dementia	2.3	2.8	-0.03	2.6	2.6	0.00
Depressive disorder	5.3	7.1	-0.08	6.3	6.4	0.00
Diabetes mellitus	24.8	19.9	0.12	20.8	22.8	-0.05
Gastroesophageal reflux disease	8.1	9.2	-0.04	8.6	8.4	0.01
Gastrointestinal hemorrhage	1.2	1.7	-0.04	1.4	1.5	-0.01
Human immunodeficiency virus infection	1.6	1.7	-0.01	1.9	1.5	0.03
Hyperlipidemia	44.2	38.8	0.11	40.9	41.8	-0.02
Hypertensive disorder	66.5	72.4	-0.13	69.7	69.6	0.00
Lesion of liver	1.4	2.1	-0.06	1.5	1.8	-0.02
Obesity	10.8	11.3	-0.02	10.7	11.3	-0.02
Osteoarthritis	11.6	13.8	-0.07	12.8	12.9	0.00
Pneumonia	2.0	3.7	-0.10	2.2	2.9	-0.05
Psoriasis	0.7	0.6	0.01	0.7	0.6	0.01
Renal impairment	11.3	19.1	-0.22	14.4	15.3	-0.03
Rheumatoid arthritis	0.9	0.9	0.00	1.1	0.9	0.02
Schizophrenia	0.2	0.3	-0.02	0.2	0.3	-0.01
Ulcerative colitis	0.1	0.2	-0.01	0.1	0.2	-0.01
Urinary tract infectious disease	2.9	4.3	-0.07	3.5	3.5	0.00
Viral hepatitis C	0.7	1.0	-0.04	0.7	0.9	-0.02
Visual system disorder	10.9	11.7	-0.03	11.3	11.3	0.00
Medical history: Cardiovascular disease						
Atrial fibrillation	13.2	9.8	0.11	11.3	11.3	0.00
Cerebrovascular disease	7.3	7.1	0.01	7.0	7.3	-0.01
Coronary arteriosclerosis	23.6	17.1	0.16	19.9	20.2	-0.01
Heart disease	46.4	36.8	0.20	40.5	41.1	-0.01
Heart failure	15.0	8.6	0.20	11.9	9.9	0.06
Ischemic heart disease	9.2	7.0	0.08	7.5	8.0	-0.02
Peripheral vascular disease	5.8	5.5	0.01	5.3	5.8	-0.02
Pulmonary embolism	0.7	0.9	-0.02	0.8	0.8	-0.01
Venous thrombosis	1.3	2.2	-0.07	1.3	1.8	-0.04
Medical history: Neoplasms						
Hematologic neoplasm	1.8	2.2	-0.03	2.0	1.9	0.01
Malignant lymphoma	1.2	1.4	-0.03	1.2	1.3	-0.01
Malignant neoplasm of anorectum	0.2	0.3	-0.01	0.2	0.2	-0.01
Malignant neoplastic disease	10.0	11.8	-0.06	10.6	10.8	-0.01

(Continued)	Before stratification			After stratification		
Characteristic	T (%)	C (%)	StdDiff	T (%)	C (%)	StdDiff
Malignant tumor of breast	1.5	1.5	0.00	1.8	1.4	0.03
Malignant tumor of colon	0.4	0.6	-0.03	0.4	0.5	-0.03
Malignant tumor of lung	0.6	0.9	-0.03	0.7	0.8	-0.01
Malignant tumor of urinary bladder	0.6	0.6	0.00	0.6	0.6	0.00
Primary malignant neoplasm of prostate	1.6	1.6	0.00	1.5	1.7	-0.02
Medication use						
Antibacterials for systemic use	30.1	34.1	-0.09	31.1	32.0	-0.02
Antidepressants	16.9	17.9	-0.03	17.7	17.5	0.01
Antiepileptics	15.8	17.2	-0.04	16.4	16.4	0.00
Antiinflammatory and antirheumatic products	18.0	20.4	-0.06	19.3	19.4	0.00
Antineoplastic agents	3.5	4.8	-0.07	3.9	4.2	-0.01
Antipsoriatics	0.9	2.4	-0.12	1.3	1.8	-0.04
Antithrombotic agents	36.7	34.8	0.04	34.1	35.7	-0.03
Diuretics	0.1	0.3	-0.04	0.1	0.2	-0.03
Drugs for acid related disorders	29.4	34.2	-0.10	30.7	31.9	-0.03
Drugs for obstructive airway diseases	18.0	19.5	-0.04	18.2	18.7	-0.01
Drugs used in diabetes	25.6	19.6	0.14	20.7	23.2	-0.06
Immunosuppressants	5.9	10.6	-0.17	7.6	8.4	-0.03
Lipid modifying agents	52.6	43.8	0.18	46.9	48.4	-0.03
Opioids	14.2	20.3	-0.16	15.9	17.5	-0.04
Psycholeptics	17.4	21.0	-0.09	18.5	19.2	-0.02
Psychostimulants, agents used for adhd and nootropics	1.4	1.7	-0.02	1.4	1.5	-0.01

Supplementary Table 47: Baseline patient characteristcs for ACEI/ARB mono (T) and CCB mono (C) prevalent-users in the CUIMC data source. We report proportion of initiators satisfying selected base-line characteristics and the standardized difference of population proportions (StdDiff) before and after stratification. Less extreme StdDiffs through stratification suggest improved balance between patient cohorts through propensity score adjustment.

	Before stratification			After stratification		
Characteristic	T (%)	C (%)	StdDiff	T (%)	C (%)	StdDiff
Age group						
15-19	0.1	0.3	-0.04	0.2	0.3	-0.01
20-24	0.6	0.7	-0.01	0.6	0.5	0.01
25-29	0.7	1.4	-0.07	0.8	0.9	-0.02
30-34	1.1	1.9	-0.06	1.3	1.3	0.00
35-39	1.8	2.8	-0.07	2.0	2.1	0.00
40-44	2.8	3.4	-0.04	2.9	3.0	-0.01
45-49	4.7	5.0	-0.01	5.0	4.6	0.02
50-54	7.1	6.5	0.02	7.2	6.2	0.04
55-59	9.9	8.3	0.06	9.4	8.8	0.02
60-64	13.1	10.8	0.07	12.3	12.0	0.01
65-69	14.9	12.8	0.06	14.3	13.6	0.02
70-74	15.4	15.3	0.00	15.2	16.0	-0.02
75-79	11.8	11.7	0.00	12.0	12.3	-0.01
80-84	8.3	8.9	-0.02	8.5	9.0	-0.02
85-89	4.7	5.9	-0.05	5.1	5.4	-0.01
90-94	2.0	3.1	-0.07	2.3	2.8	-0.03
95-99	0.8	0.9	-0.01	1.0	0.8	0.01
100-104	<0.1	0.4	-0.07	<0.1	0.3	-0.06
Gender: female	50.1	56.7	-0.13	53.2	52.5	0.01
Race						
race = Asian	1.7	1.8	-0.01	1.7	1.9	-0.01
race = Black or African American	5.9	11.0	-0.18	7.9	7.9	0.00
race = Other Race	0.2	<0.1	0.03	0.2	<0.1	0.04
race = White	36.4	31.2	0.11	33.8	34.6	-0.02
race = Unknown	1.9	1.9	0.00	1.9	2.0	-0.01
race = Native Hawaiian or Other Pacific Islander	<0.1	0.0		<0.1	0.0	
race = American Indian or Alaska Native	0.2	<0.1	0.02	0.2	<0.1	0.02
race = Asian Indian	0.2	0.3	-0.02	0.2	0.4	-0.03
race = Bangladeshi	0.0	<0.1		0.0	<0.1	
race = Chinese	<0.1	<0.1	0.02	<0.1	<0.1	0.03
race = Filipino	<0.1	<0.1	-0.02	<0.1	<0.1	-0.02
race = Japanese	<0.1	0.0		<0.1	0.0	
race = Korean	<0.1	<0.1	-0.01	<0.1	<0.1	0.00
race = Laotian	0.4	0.5	-0.02	0.4	0.5	-0.01
race = Pakistani	0.0	<0.1		0.0	<0.1	
race = Thai	<0.1	0.0		<0.1	0.0	
race = Vietnamese	<0.1	<0.1	0.00	<0.1	<0.1	-0.01
race = Micronesian	<0.1	0.0		<0.1	0.0	
race = Other Pacific Islander	0.8	0.7	0.02	0.8	0.6	0.04

(Continued)	Before stratification			After stratification		
Characteristic	T (%)	C (%)	StdDiff	T (%)	C (%)	StdDiff
Ethnicity						
ethnicity = Hispanic or Latino	11.5	14.0	-0.08	12.2	13.1	-0.03
ethnicity = Not Hispanic or Latino	35.3	34.6	0.02	34.6	35.0	-0.01
Medical history: General						
Acute respiratory disease	3.6	4.2	-0.03	3.9	3.8	0.01
Attention deficit hyperactivity disorder	0.2	0.2	-0.02	0.2	0.2	-0.02
Chronic liver disease	0.7	1.3	-0.06	0.7	1.1	-0.03
Chronic obstructive lung disease	2.7	3.6	-0.05	3.0	2.9	0.00
Crohn’s disease	0.3	0.2	0.02	0.3	0.2	0.03
Dementia	1.7	2.7	-0.06	2.0	2.3	-0.03
Depressive disorder	4.9	6.0	-0.05	5.5	5.2	0.01
Diabetes mellitus	21.5	14.3	0.19	18.1	18.1	0.00
Gastroesophageal reflux disease	7.1	7.1	0.00	7.3	6.7	0.02
Gastrointestinal hemorrhage	0.8	1.2	-0.04	0.8	0.9	-0.01
Human immunodeficiency virus infection	1.9	1.8	0.01	2.2	1.5	0.05
Hyperlipidemia	38.3	31.9	0.13	35.7	35.8	0.00
Hypertensive disorder	61.1	68.7	-0.16	64.5	63.3	0.02
Lesion of liver	0.9	1.7	-0.08	1.0	1.5	-0.04
Obesity	9.2	9.0	0.01	9.1	9.2	0.00
Osteoarthritis	10.9	11.9	-0.03	11.7	11.6	0.00
Pneumonia	1.1	2.5	-0.10	1.4	1.8	-0.03
Psoriasis	0.7	0.6	0.02	0.7	0.5	0.02
Renal impairment	6.5	11.5	-0.17	7.8	8.8	-0.04
Rheumatoid arthritis	0.8	0.8	0.00	1.0	0.7	0.03
Schizophrenia	0.2	0.3	-0.02	0.2	0.2	0.00
Ulcerative colitis	0.2	<0.1	0.02	0.2	<0.1	0.03
Urinary tract infectious disease	2.1	3.2	-0.07	2.5	2.6	0.00
Viral hepatitis C	0.4	0.8	-0.05	0.4	0.7	-0.04
Visual system disorder	11.1	10.1	0.03	11.0	10.1	0.03
Medical history: Cardiovascular disease						
Atrial fibrillation	5.3	4.4	0.04	5.1	4.5	0.03
Cerebrovascular disease	5.3	5.4	0.00	5.4	5.1	0.01
Coronary arteriosclerosis	13.0	11.8	0.04	12.5	12.8	-0.01
Heart disease	27.7	26.3	0.03	27.3	27.0	0.01
Heart failure	4.2	2.5	0.09	3.8	2.9	0.05
Ischemic heart disease	3.4	3.8	-0.02	3.3	3.9	-0.03
Peripheral vascular disease	3.9	3.6	0.01	3.6	3.6	0.00
Pulmonary embolism	0.3	0.5	-0.02	0.4	0.4	0.00
Venous thrombosis	0.6	1.3	-0.07	0.7	1.0	-0.03
Medical history: Neoplasms						
Hematologic neoplasm	1.8	1.8	0.00	2.0	1.4	0.04
Malignant lymphoma	1.0	1.4	-0.03	1.0	1.2	-0.02
Malignant neoplasm of anorectum	0.2	0.2	0.02	0.2	0.1	0.02
Malignant neoplastic disease	9.5	11.1	-0.05	10.0	10.1	0.00
Malignant tumor of breast	1.7	1.4	0.02	1.8	1.4	0.04

(Continued)	Before stratification			After stratification		
Characteristic	T (%)	C (%)	StdDiff	T (%)	C (%)	StdDiff
Malignant tumor of colon	0.3	0.5	-0.03	0.3	0.4	-0.02
Malignant tumor of lung	0.5	0.8	-0.04	0.6	0.6	-0.01
Malignant tumor of urinary bladder	0.5	0.4	0.01	0.6	0.4	0.03
Primary malignant neoplasm of prostate	1.5	1.8	-0.03	1.5	1.7	-0.02
Medication use						
Antibacterials for systemic use	25.7	29.1	-0.07	26.7	26.1	0.01
Antidepressants	15.2	15.8	-0.02	15.5	15.0	0.02
Antiepileptics	13.3	13.3	0.00	13.6	12.6	0.03
Antiinflammatory and antirheumatic products	17.1	20.2	-0.08	18.4	18.3	0.00
Antineoplastic agents	3.2	4.5	-0.07	3.6	3.7	0.00
Antipsoriatics	0.6	1.5	-0.09	0.7	1.1	-0.04
Antithrombotic agents	21.9	25.7	-0.09	23.0	22.9	0.00
Diuretics	<0.1	<0.1	0.01	<0.1	<0.1	0.01
Drugs for acid related disorders	22.6	28.8	-0.14	24.5	25.4	-0.02
Drugs for obstructive airway diseases	14.0	15.5	-0.04	14.8	14.2	0.02
Drugs used in diabetes	22.7	13.0	0.25	18.4	19.0	-0.02
Immunosuppressants	5.4	9.5	-0.16	6.7	7.0	-0.01
Lipid modifying agents	43.2	35.1	0.17	39.8	40.5	-0.01
Opioids	10.3	15.9	-0.16	11.7	13.0	-0.04
Psycholeptics	14.3	16.9	-0.07	15.3	15.3	0.00
Psychostimulants, agents used for adhd and nootropics	1.5	1.6	-0.01	1.5	1.5	0.00

Supplementary Table 48: Baseline patient characteristcs for ACEI/ARB +combo (T) and CCB +combo (C) prevalent-users in the CUIMC data source. We report proportion of initiators satisfying selected base-line characteristics and the standardized difference of population proportions (StdDiff) before and after stratification. Less extreme StdDiffs through stratification suggest improved balance between patient cohorts through propensity score adjustment.

	Before stratification			After stratification		
Characteristic	T (%)	C (%)	StdDiff	T (%)	C (%)	StdDiff
Age group						
15-19	0.1	0.2	-0.02	0.2	0.1	0.01
20-24	0.3	0.6	-0.04	0.4	0.4	0.00
25-29	0.4	1.1	-0.08	0.5	0.7	-0.03
30-34	0.8	1.7	-0.08	0.9	1.2	-0.03
35-39	1.4	2.7	-0.09	1.5	1.9	-0.03
40-44	2.3	3.0	-0.05	2.5	2.5	0.00
45-49	3.8	4.3	-0.03	4.2	3.8	0.02
50-54	6.2	5.9	0.01	6.3	6.0	0.01
55-59	9.6	8.5	0.04	9.2	9.2	0.00
60-64	12.7	11.0	0.05	11.9	11.9	0.00
65-69	14.5	13.3	0.04	14.1	13.8	0.01
70-74	15.9	14.8	0.03	15.7	15.4	0.01
75-79	12.9	12.1	0.02	12.9	12.9	0.00
80-84	9.7	9.5	0.00	9.7	9.9	-0.01
85-89	5.7	6.5	-0.03	6.2	5.8	0.02
90-94	2.9	3.3	-0.03	3.1	3.1	0.00
95-99	0.7	1.1	-0.04	0.8	1.0	-0.02
100-104	0.1	0.3	-0.05	0.1	0.2	-0.04
Gender: female	50.1	54.6	-0.09	52.1	51.9	0.00
Race						
race = Asian	1.4	1.6	-0.02	1.4	1.6	-0.01
race = Black or African American	7.9	12.4	-0.15	9.5	9.2	0.01
race = Other Race	0.1	0.1	-0.01	0.1	0.1	-0.01
race = White	35.5	31.4	0.09	33.8	34.4	-0.01
race = Unknown	2.1	2.0	0.01	2.0	2.3	-0.01
race = Native Hawaiian or Other Pacific Islander	0.0	<0.1	0.01	0.0	0.0	0.01
race = American Indian or Alaska Native	0.1	0.1	0.02	0.1	0.1	0.03
race = Asian Indian	0.2	0.4	-0.04	0.2	0.4	-0.05
race = Bangladeshi	0.0	<0.1	-0.02	0.0	0.1	-0.03
race = Chinese	0.1	<0.1	0.02	0.1	0.0	0.02
race = Filipino	0.0	0.1	-0.02	0.0	0.1	-0.02
race = Indonesian	0.0	0.0		0.0	0.0	
race = Japanese	0.0	<0.1	-0.01	0.0	0.0	0.00
race = Korean	0.1	<0.1	0.01	0.1	0.0	0.01
race = Laotian	0.5	0.7	-0.03	0.5	0.6	0.00
race = Pakistani	0.0	<0.1	-0.01	0.0	0.0	-0.01
race = Thai	0.0	<0.1	-0.01	0.0	0.0	-0.01
race = Vietnamese	0.0	<0.1	-0.01	0.0	0.0	0.00
race = Micronesian	0.0	0.0		0.0	0.0	

(Continued)	Before stratification			After stratification		
Characteristic	T (%)	C (%)	StdDiff	T (%)	C (%)	StdDiff
race = Other Pacific Islander	0.6	0.7	-0.01	0.7	0.6	0.01
Ethnicity						
ethnicity = Hispanic or Latino	12.5	14.5	-0.06	13.2	13.4	-0.01
ethnicity = Not Hispanic or Latino	35.4	35.3	0.00	35.1	35.7	-0.01
Medical history: General						
Acute respiratory disease	4.5	6.2	-0.08	4.9	5.0	0.00
Attention deficit hyperactivity disorder	0.1	0.2	-0.02	0.1	0.2	-0.02
Chronic liver disease	1.1	1.9	-0.06	1.4	1.4	0.00
Chronic obstructive lung disease	4.0	4.8	-0.04	4.3	4.0	0.01
Crohn’s disease	0.2	0.3	-0.01	0.2	0.2	0.00
Dementia	2.0	3.2	-0.08	2.4	2.4	0.00
Depressive disorder	5.1	7.5	-0.10	5.9	6.2	-0.01
Diabetes mellitus	23.5	21.4	0.05	21.6	23.0	-0.03
Gastroesophageal reflux disease	7.9	9.7	-0.06	8.4	8.5	0.00
Gastrointestinal hemorrhage	1.2	1.9	-0.06	1.4	1.5	-0.01
Human immunodeficiency virus infection	1.5	1.8	-0.03	1.8	1.5	0.02
Hyperlipidemia	43.3	38.5	0.10	41.3	42.0	-0.01
Hypertensive disorder	67.5	73.7	-0.14	69.7	69.6	0.00
Lesion of liver	1.3	2.4	-0.08	1.5	1.7	-0.01
Obesity	11.5	10.6	0.03	10.9	11.4	-0.02
Osteoarthritis	12.5	13.5	-0.03	13.2	13.2	0.00
Pneumonia	1.9	4.2	-0.14	2.2	2.8	-0.04
Psoriasis	0.7	0.6	0.02	0.7	0.5	0.02
Renal impairment	10.5	22.2	-0.32	13.4	14.9	-0.04
Rheumatoid arthritis	0.9	0.9	-0.01	1.0	0.9	0.01
Schizophrenia	0.2	0.4	-0.04	0.2	0.3	-0.01
Ulcerative colitis	0.2	0.2	-0.01	0.2	0.2	-0.01
Urinary tract infectious disease	2.8	4.8	-0.10	3.3	3.4	-0.01
Viral hepatitis C	0.6	1.2	-0.06	0.8	0.9	-0.01
Visual system disorder	10.9	12.0	-0.03	11.3	11.3	0.00
Medical history: Cardiovascular disease						
Atrial fibrillation	11.9	9.2	0.09	11.0	10.3	0.02
Cerebrovascular disease	6.9	7.3	-0.02	7.0	6.7	0.01
Coronary arteriosclerosis	21.3	18.5	0.07	20.0	19.8	0.01
Heart disease	42.8	38.2	0.09	40.6	40.1	0.01
Heart failure	12.4	8.7	0.12	11.1	9.2	0.06
Ischemic heart disease	8.5	8.0	0.02	7.9	8.0	-0.01
Peripheral vascular disease	5.5	6.0	-0.02	5.4	5.7	-0.01
Pulmonary embolism	0.7	0.8	-0.01	0.8	0.7	0.01
Venous thrombosis	1.2	2.4	-0.09	1.4	1.7	-0.02
Medical history: Neoplasms						
Hematologic neoplasm	1.6	2.4	-0.06	2.0	1.8	0.01
Malignant lymphoma	1.1	1.6	-0.04	1.2	1.2	-0.01
Malignant neoplasm of anorectum	0.2	0.3	-0.01	0.2	0.2	0.01
Malignant neoplastic disease	9.6	12.4	-0.09	10.5	10.4	0.00

(Continued)	Before stratification			After stratification		
Characteristic	T (%)	C (%)	StdDiff	T (%)	C (%)	StdDiff
Malignant tumor of breast	1.5	1.3	0.02	1.6	1.3	0.03
Malignant tumor of colon	0.4	0.6	-0.04	0.4	0.5	-0.02
Malignant tumor of lung	0.6	1.0	-0.05	0.7	0.8	-0.01
Malignant tumor of urinary bladder	0.6	0.6	0.00	0.6	0.5	0.02
Primary malignant neoplasm of prostate	1.6	1.8	-0.01	1.7	1.7	0.00
Medication use						
Antibacterials for systemic use	29.9	35.8	-0.12	31.1	32.1	-0.02
Antidepressants	16.8	18.4	-0.04	17.4	17.2	0.01
Antiepileptics	15.8	18.1	-0.06	16.6	16.4	0.00
Antiinflammatory and antirheumatic products	19.6	20.6	-0.03	19.9	20.3	-0.01
Antineoplastic agents	3.4	5.1	-0.09	3.8	4.0	-0.01
Antipsoriatics	0.8	2.8	-0.15	1.1	1.7	-0.05
Antithrombotic agents	34.7	38.4	-0.08	34.9	35.7	-0.02
Diuretics	0.1	0.1	-0.02	0.1	0.1	0.00
Drugs for acid related disorders	29.0	36.5	-0.16	30.8	32.1	-0.03
Drugs for obstructive airway diseases	18.0	20.1	-0.05	18.2	18.7	-0.01
Drugs used in diabetes	25.0	20.5	0.11	22.0	24.4	-0.06
Immunosuppressants	5.6	12.7	-0.25	7.1	8.5	-0.05
Lipid modifying agents	51.3	44.6	0.14	48.3	49.5	-0.03
Opioids	14.0	22.5	-0.22	15.8	17.4	-0.04
Psycholeptics	17.1	22.2	-0.13	18.3	19.1	-0.02
Psychostimulants, agents used for adhd and nootropics	1.4	1.8	-0.03	1.5	1.6	-0.01

Supplementary Table 49: Baseline patient characteristcs for ACEI/ARB mono (T) and THZ mono (C) prevalent-users in the CUIMC data source. We report proportion of initiators satisfying selected base-line characteristics and the standardized difference of population proportions (StdDiff) before and after stratification. Less extreme StdDiffs through stratification suggest improved balance between patient cohorts through propensity score adjustment.

	Before stratification			After stratification		
Characteristic	T (%)	C (%)	StdDiff	T (%)	C (%)	StdDiff
Age group						
15-19	0.1	<0.3	0.00	0.2	<0.3	0.00
20-24	0.6	<0.3	0.07	0.6	<0.3	0.09
25-29	0.7	0.3	0.05	0.7	<0.3	0.06
30-34	1.1	0.7	0.04	1.2	0.6	0.06
35-39	1.8	1.1	0.06	1.8	0.8	0.09
40-44	2.8	3.8	-0.06	2.7	3.1	-0.02
45-49	4.7	5.8	-0.05	4.8	5.1	-0.01
50-54	7.1	8.1	-0.04	7.2	6.9	0.01
55-59	9.9	10.6	-0.02	10.2	9.6	0.02
60-64	13.1	12.5	0.02	13.2	12.7	0.02
65-69	14.9	14.6	0.01	14.8	15.3	-0.01
70-74	15.4	13.9	0.04	15.1	14.7	0.01
75-79	11.8	12.8	-0.03	11.9	14.0	-0.06
80-84	8.3	7.6	0.02	8.0	8.7	-0.02
85-89	4.7	4.9	0.00	4.7	4.9	-0.01
90-94	2.0	2.4	-0.03	2.0	2.4	-0.03
95-99	0.8	0.5	0.04	0.9	0.8	0.01
100-104	<0.1	0.0		<0.1	0.0	
Gender: female	50.1	63.3	-0.27	53.5	49.2	0.09
Race						
race = Asian	1.7	0.9	0.07	1.6	0.6	0.10
race = Black or African American	5.9	9.8	-0.15	6.6	6.5	0.00
race = Other Race	0.2	0.0		0.2	0.0	
race = White	36.4	31.6	0.10	35.4	35.0	0.01
race = Unknown	1.9	3.0	-0.07	1.9	2.8	-0.06
race = Native Hawaiian or Other Pacific Islander	<0.1	0.0		<0.1	0.0	
race = American Indian or Alaska Native	0.2	<0.3	0.03	0.2	<0.3	0.05
race = Asian Indian	0.2	<0.3	0.04	0.2	<0.3	0.04
race = Chinese	<0.1	0.0		<0.1	0.0	
race = Filipino	<0.1	0.0		<0.1	0.0	
race = Japanese	<0.1	0.0		<0.1	0.0	
race = Korean	<0.1	0.0		<0.1	0.0	
race = Laotian	0.4	0.6	-0.03	0.4	0.8	-0.05
race = Thai	<0.1	0.0		<0.1	0.0	
race = Vietnamese	<0.1	0.0		<0.1	0.0	
race = Micronesian	<0.1	0.0		<0.1	0.0	
race = Other Pacific Islander	0.8	0.3	0.06	0.9	0.3	0.07
Ethnicity						
ethnicity = Hispanic or Latino	11.5	14.2	-0.08	11.9	12.7	-0.03

(Continued)	Before stratification			After stratification		
Characteristic	T (%)	C (%)	StdDiff	T (%)	C (%)	StdDiff
ethnicity = Not Hispanic or Latino	35.3	33.9	0.03	34.9	35.7	-0.02
Medical history: General						
Acute respiratory disease	3.6	3.7	-0.01	3.5	3.6	0.00
Attention deficit hyperactivity disorder	0.2	<0.3	0.01	0.2	<0.3	0.03
Chronic liver disease	0.7	1.0	-0.03	0.6	1.3	-0.06
Chronic obstructive lung disease	2.7	2.0	0.05	2.6	2.3	0.02
Crohn’s disease	0.3	<0.3	0.02	0.3	<0.3	0.04
Dementia	1.7	1.3	0.04	1.7	1.2	0.04
Depressive disorder	4.9	5.7	-0.04	5.1	5.5	-0.02
Diabetes mellitus	21.5	10.7	0.30	19.1	20.2	-0.03
Gastroesophageal reflux disease	7.1	6.3	0.03	7.1	5.9	0.05
Gastrointestinal hemorrhage	0.8	0.5	0.03	0.7	0.3	0.05
Human immunodeficiency virus infection	1.9	1.4	0.04	1.9	1.3	0.04
Hyperlipidemia	38.3	33.7	0.10	37.0	38.0	-0.02
Hypertensive disorder	61.1	65.7	-0.10	62.1	62.3	0.00
Lesion of liver	0.9	1.2	-0.03	0.9	1.4	-0.05
Obesity	9.2	11.7	-0.08	9.6	10.2	-0.02
Osteoarthritis	10.9	12.7	-0.06	11.1	12.1	-0.03
Pneumonia	1.1	0.8	0.03	1.0	0.9	0.01
Psoriasis	0.7	0.5	0.03	0.7	0.4	0.03
Renal impairment	6.5	5.6	0.04	6.2	8.7	-0.10
Rheumatoid arthritis	0.8	0.6	0.03	0.8	0.7	0.02
Schizophrenia	0.2	<0.3	0.03	0.2	<0.3	0.03
Ulcerative colitis	0.2	0.0		0.2	0.0	
Urinary tract infectious disease	2.1	1.8	0.02	2.1	1.5	0.05
Viral hepatitis C	0.4	0.5	-0.03	0.3	0.8	-0.06
Visual system disorder	11.1	10.5	0.02	10.9	10.4	0.01
Medical history: Cardiovascular disease						
Atrial fibrillation	5.3	5.3	0.00	5.1	6.1	-0.04
Cerebrovascular disease	5.3	3.9	0.07	5.1	4.6	0.02
Coronary arteriosclerosis	13.0	8.8	0.14	12.1	13.0	-0.03
Heart disease	27.7	21.9	0.14	26.5	27.1	-0.01
Heart failure	4.2	2.1	0.12	3.9	3.2	0.04
Ischemic heart disease	3.4	2.4	0.06	3.1	4.2	-0.06
Peripheral vascular disease	3.9	2.6	0.07	3.5	3.8	-0.01
Pulmonary embolism	0.3	0.5	-0.03	0.3	0.6	-0.04
Venous thrombosis	0.6	0.5	0.02	0.6	0.5	0.01
Medical history: Neoplasms						
Hematologic neoplasm	1.8	1.3	0.04	1.7	1.2	0.04
Malignant lymphoma	1.0	1.1	0.00	1.0	0.8	0.03
Malignant neoplasm of anorectum	0.2	<0.3	0.02	0.2	<0.3	0.03
Malignant neoplastic disease	9.5	9.5	0.00	9.4	9.2	0.01
Malignant tumor of breast	1.7	2.2	-0.04	1.9	1.5	0.03
Malignant tumor of colon	0.3	0.3	-0.01	0.3	0.3	-0.01
Malignant tumor of lung	0.5	<0.3	0.04	0.5	0.4	0.01

(Continued)	Before stratification			After stratification		
Characteristic	T (%)	C (%)	StdDiff	T (%)	C (%)	StdDiff
Malignant tumor of urinary bladder	0.5	0.7	-0.03	0.5	0.9	-0.04
Primary malignant neoplasm of prostate	1.5	1.6	-0.01	1.4	1.9	-0.04
Medication use						
Antibacterials for systemic use	25.7	22.2	0.08	24.9	22.9	0.05
Antidepressants	15.2	14.4	0.02	15.1	14.0	0.03
Antiepileptics	13.3	12.1	0.03	13.0	11.9	0.04
Antiinflammatory and antirheumatic products	17.1	19.7	-0.07	17.5	17.3	0.00
Antineoplastic agents	3.2	3.0	0.01	3.1	2.5	0.04
Antipsoriatics	0.6	0.8	-0.03	0.6	0.7	-0.02
Antithrombotic agents	21.9	14.2	0.20	20.0	20.8	-0.02
Diuretics	<0.1	<0.3	-0.01	<0.1	<0.3	-0.03
Drugs for acid related disorders	22.6	21.9	0.02	22.3	22.9	-0.01
Drugs for obstructive airway diseases	14.0	13.7	0.01	13.7	13.6	0.00
Drugs used in diabetes	22.7	12.4	0.27	20.3	21.4	-0.03
Immunosuppressants	5.4	3.9	0.07	5.2	4.6	0.03
Lipid modifying agents	43.2	33.4	0.20	40.7	41.9	-0.02
Opioids	10.3	10.5	-0.01	10.3	10.3	0.00
Psycholeptics	14.3	13.4	0.03	14.3	13.4	0.03
Psychostimulants, agents used for adhd and nootropics	1.5	1.2	0.02	1.5	0.9	0.05

Supplementary Table 50: Baseline patient characteristcs for ACEI/ARB +combo (T) and THZ +combo (C) prevalent-users in the CUIMC data source. We report proportion of initiators satisfying selected base-line characteristics and the standardized difference of population proportions (StdDiff) before and after stratification. Less extreme StdDiffs through stratification suggest improved balance between patient cohorts through propensity score adjustment.

	Before stratification			After stratification		
Characteristic	T (%)	C (%)	StdDiff	T (%)	C (%)	StdDiff
Age group						
15-19	0.1	<0.1	0.01	0.2	0.1	0.01
20-24	0.3	0.2	0.04	0.4	<0.1	0.06
25-29	0.5	0.2	0.04	0.5	0.2	0.04
30-34	0.9	0.9	0.00	0.9	0.8	0.02
35-39	1.6	1.3	0.02	1.6	1.3	0.03
40-44	2.3	3.2	-0.06	2.3	2.5	-0.01
45-49	3.7	4.9	-0.06	3.9	4.0	0.00
50-54	5.9	6.2	-0.01	6.1	5.5	0.03
55-59	9.0	9.4	-0.01	9.1	8.8	0.01
60-64	12.1	11.6	0.02	12.2	11.9	0.01
65-69	14.3	13.9	0.01	14.1	14.7	-0.02
70-74	15.7	15.2	0.01	15.5	15.8	-0.01
75-79	13.0	13.5	-0.02	13.0	13.8	-0.02
80-84	10.0	9.6	0.02	9.8	10.4	-0.02
85-89	6.4	5.9	0.02	6.3	6.0	0.01
90-94	3.2	3.2	0.00	3.2	3.2	0.00
95-99	0.9	0.6	0.03	0.9	0.8	0.01
100-104	0.1	<0.1	0.00	0.1	<0.1	-0.01
10-14	0.0	0.0		0.0	0.0	
Gender: female	47.2	61.5	-0.29	51.0	48.1	0.06
Race						
race = Asian	1.5	0.8	0.07	1.5	0.9	0.05
race = Black or African American	8.3	10.4	-0.07	8.7	9.0	-0.01
race = Other Race	0.1	<0.1	0.02	0.1	<0.1	0.02
race = White	35.7	32.1	0.07	34.9	34.6	0.01
race = Unknown	2.1	2.8	-0.04	2.2	2.6	-0.03
race = Native Hawaiian or Other Pacific Islander	0.0	0.0		0.0	0.0	
race = American Indian or Alaska Native	0.1	<0.1	0.04	0.1	<0.1	0.04
race = Asian Indian	0.2	0.2	0.01	0.2	0.4	-0.03
race = Bangladeshi	0.0	0.0		0.0	0.0	
race = Chinese	0.1	0.0		0.1	0.0	
race = Filipino	0.0	<0.1	0.01	0.0	<0.1	0.01
race = Indonesian	0.0	0.0		0.0	0.0	
race = Japanese	0.0	0.0		0.0	0.0	
race = Korean	0.1	0.0		0.1	0.0	
race = Laotian	0.6	0.5	0.02	0.6	0.6	0.00
race = Pakistani	0.0	0.0		0.0	0.0	
race = Sri Lankan	0.0	0.0		0.0	0.0	
race = Thai	0.0	<0.1	-0.01	0.0	<0.1	-0.01

(Continued)	Before stratification			After stratification		
Characteristic	T (%)	C (%)	StdDiff	T (%)	C (%)	StdDiff
race = Vietnamese	0.0	0.0		0.0	0.0	
race = Micronesian	0.0	0.0		0.0	0.0	
race = Other Pacific Islander	0.6	0.5	0.01	0.6	0.5	0.02
Ethnicity						
ethnicity = Hispanic or Latino	12.9	13.3	-0.01	12.8	13.8	-0.03
ethnicity = Not Hispanic or Latino	35.6	35.1	0.01	35.3	35.3	0.00
Medical history: General						
Acute respiratory disease	4.8	5.6	-0.03	4.9	5.0	-0.01
Attention deficit hyperactivity disorder	0.1	0.2	-0.01	0.1	<0.1	0.01
Chronic liver disease	1.4	1.4	0.00	1.3	1.6	-0.02
Chronic obstructive lung disease	4.6	3.9	0.03	4.3	3.9	0.02
Crohn’s disease	0.3	0.3	0.00	0.3	0.3	0.00
Dementia	2.7	2.0	0.05	2.5	2.5	0.00
Depressive disorder	5.7	5.9	-0.01	5.6	5.9	-0.01
Diabetes mellitus	25.7	16.9	0.22	23.4	25.8	-0.06
Gastroesophageal reflux disease	8.5	8.2	0.01	8.5	8.0	0.02
Gastrointestinal hemorrhage	1.3	1.2	0.01	1.3	1.0	0.03
Human immunodeficiency virus infection	1.6	1.4	0.02	1.6	1.4	0.01
Hyperlipidemia	44.6	39.8	0.10	43.2	43.8	-0.01
Hypertensive disorder	68.3	70.4	-0.05	68.6	69.6	-0.02
Lesion of liver	1.5	1.4	0.01	1.4	1.4	0.00
Obesity	10.7	12.6	-0.06	11.0	11.5	-0.01
Osteoarthritis	12.7	14.5	-0.05	13.0	13.6	-0.02
Pneumonia	2.4	2.6	-0.01	2.3	2.4	0.00
Psoriasis	0.7	0.7	0.00	0.7	0.7	0.00
Renal impairment	13.4	13.1	0.01	13.2	14.2	-0.03
Rheumatoid arthritis	0.9	0.9	0.00	0.9	0.8	0.01
Schizophrenia	0.3	0.2	0.02	0.3	0.4	-0.02
Ulcerative colitis	0.1	0.3	-0.03	0.1	0.3	-0.04
Urinary tract infectious disease	3.4	3.3	0.01	3.4	2.9	0.02
Viral hepatitis C	0.9	0.7	0.03	0.9	0.9	0.00
Visual system disorder	11.5	11.7	0.00	11.5	11.2	0.01
Medical history: Cardiovascular disease						
Atrial fibrillation	12.5	10.9	0.05	11.8	12.2	-0.01
Cerebrovascular disease	7.9	6.7	0.05	7.5	7.9	-0.01
Coronary arteriosclerosis	23.7	14.7	0.23	21.4	22.0	-0.02
Heart disease	45.8	34.5	0.23	42.8	43.3	-0.01
Heart failure	14.4	8.7	0.18	12.9	10.8	0.07
Ischemic heart disease	9.3	5.7	0.14	8.3	8.4	0.00
Peripheral vascular disease	6.2	4.5	0.08	5.7	6.2	-0.02
Pulmonary embolism	0.7	1.0	-0.03	0.7	1.0	-0.04
Venous thrombosis	1.4	1.9	-0.03	1.4	2.0	-0.04
Medical history: Neoplasms						
Hematologic neoplasm	1.8	1.7	0.01	1.7	1.5	0.02
Malignant lymphoma	1.2	1.2	0.00	1.2	1.0	0.01

(Continued)	Before stratification			After stratification		
Characteristic	T (%)	C (%)	StdDiff	T (%)	C (%)	StdDiff
Malignant neoplasm of anorectum	0.2	0.3	0.00	0.2	0.3	-0.01
Malignant neoplastic disease	10.5	10.5	0.00	10.4	10.5	0.00
Malignant tumor of breast	1.5	1.9	-0.03	1.6	1.4	0.02
Malignant tumor of colon	0.4	0.6	-0.02	0.4	0.7	-0.04
Malignant tumor of lung	0.7	0.5	0.03	0.7	0.5	0.02
Malignant tumor of urinary bladder	0.6	0.7	-0.01	0.6	1.1	-0.05
Primary malignant neoplasm of prostate	1.8	1.3	0.04	1.7	1.8	-0.01
Medication use						
Antibacterials for systemic use	31.0	30.7	0.01	30.6	30.0	0.01
Antidepressants	17.5	16.8	0.02	17.3	16.7	0.02
Antiepileptics	16.7	15.8	0.03	16.4	16.0	0.01
Antiinflammatory and antirheumatic products	19.1	20.6	-0.04	19.5	19.4	0.00
Antineoplastic agents	3.8	3.8	0.00	3.8	3.3	0.03
Antipsoriatics	1.2	1.6	-0.04	1.2	1.3	0.00
Antithrombotic agents	38.6	28.2	0.22	35.6	37.6	-0.04
Diuretics	0.1	0.5	-0.07	0.1	0.3	-0.04
Drugs for acid related disorders	31.3	29.8	0.03	30.5	31.3	-0.02
Drugs for obstructive airway diseases	18.7	18.8	0.00	18.4	18.5	0.00
Drugs used in diabetes	26.7	18.5	0.20	24.5	26.6	-0.05
Immunosuppressants	6.2	6.5	-0.01	6.3	7.0	-0.03
Lipid modifying agents	53.9	43.0	0.22	50.8	53.0	-0.04
Opioids	15.5	16.2	-0.02	15.3	16.1	-0.02
Psycholeptics	18.5	18.6	0.00	18.2	18.4	0.00
Psychostimulants, agents used for adhd and nootropics	1.4	1.4	0.00	1.4	1.2	0.02

Supplementary Table 51: Baseline patient characteristcs for ACEI mono (T) and ARB mono (C) prevalent-users in the CUIMC data source. We report proportion of initiators satisfying selected base-line characteristics and the standardized difference of population proportions (StdDiff) before and after stratification. Less extreme StdDiffs through stratification suggest improved balance between patient cohorts through propensity score adjustment.

	Before stratification			After stratification		
Characteristic	T (%)	C (%)	StdDiff	T (%)	C (%)	StdDiff
Age group						
15-19	0.3	0.0		0.3	0.0	
20-24	0.9	0.3	0.07	0.9	0.3	0.07
25-29	1.0	0.4	0.07	1.0	0.5	0.07
30-34	1.6	0.7	0.08	1.4	0.8	0.06
35-39	2.3	1.3	0.07	2.1	1.3	0.06
40-44	3.1	2.5	0.03	2.8	2.7	0.01
45-49	5.1	4.3	0.04	4.9	4.3	0.03
50-54	8.2	6.1	0.08	7.5	6.7	0.03
55-59	10.2	9.7	0.02	9.6	10.2	-0.02
60-64	13.1	13.0	0.00	12.5	13.3	-0.02
65-69	14.8	14.9	0.00	15.1	15.0	0.00
70-74	14.5	16.3	-0.05	14.8	16.0	-0.04
75-79	11.2	12.4	-0.04	11.9	12.2	-0.01
80-84	7.1	9.4	-0.08	8.0	8.8	-0.03
85-89	4.3	5.0	-0.03	4.7	4.8	-0.01
90-94	1.6	2.4	-0.06	1.7	2.1	-0.03
95-99	0.7	1.0	-0.03	0.7	0.9	-0.02
100-104	<0.2	<0.2	-0.03	<0.2	<0.2	-0.03
Gender: female	47.2	52.5	-0.10	50.8	49.7	0.02
Race						
race = Asian	1.1	2.1	-0.08	1.2	2.0	-0.07
race = Black or African American	6.5	5.4	0.05	6.3	5.6	0.03
race = Other Race	<0.2	0.2	0.00	<0.2	<0.2	0.00
race = White	35.0	37.6	-0.05	36.4	36.5	0.00
race = Unknown	1.7	2.1	-0.03	1.9	2.1	-0.02
race = Native Hawaiian or Other Pacific Islander	0.0	<0.2		0.0	<0.2	
race = American Indian or Alaska Native	<0.2	0.2	-0.01	<0.2	0.2	-0.02
race = Asian Indian	0.2	0.2	0.00	<0.2	0.2	0.00
race = Chinese	<0.2	<0.2	0.00	<0.2	<0.2	0.00
race = Filipino	0.0	<0.2		0.0	<0.2	
race = Japanese	<0.2	0.0		<0.2	0.0	
race = Korean	0.0	<0.2		0.0	<0.2	
race = Laotian	0.5	0.4	0.01	0.3	0.5	-0.02
race = Thai	0.0	<0.2		0.0	<0.2	
race = Vietnamese	<0.2	0.0		<0.2	0.0	
race = Micronesian	<0.2	0.0		<0.2	0.0	
race = Other Pacific Islander	1.1	0.5	0.07	1.1	0.5	0.07
Ethnicity						
ethnicity = Hispanic or Latino	14.6	8.5	0.19	11.2	11.6	-0.01

(Continued)	Before stratification			After stratification		
Characteristic	T (%)	C (%)	StdDiff	T (%)	C (%)	StdDiff
ethnicity = Not Hispanic or Latino	32.6	37.8	-0.11	35.3	35.3	0.00
Medical history: General						
Acute respiratory disease	3.6	3.4	0.01	3.6	3.3	0.02
Attention deficit hyperactivity disorder	0.2	<0.2	0.04	0.2	<0.2	0.05
Chronic liver disease	0.9	0.5	0.05	0.8	0.6	0.03
Chronic obstructive lung disease	2.4	3.0	-0.04	2.4	2.9	-0.03
Crohn’s disease	0.2	0.4	-0.03	0.2	0.3	-0.01
Dementia	2.1	1.4	0.06	1.9	1.6	0.02
Depressive disorder	5.3	4.3	0.05	4.8	4.6	0.01
Diabetes mellitus	24.5	18.5	0.14	21.0	21.1	0.00
Gastroesophageal reflux disease	7.3	6.8	0.02	6.9	7.0	0.00
Gastrointestinal hemorrhage	0.8	0.7	0.01	0.7	0.8	-0.01
Human immunodeficiency virus infection	3.1	0.9	0.16	2.3	1.3	0.07
Hyperlipidemia	37.6	38.3	-0.01	37.5	38.2	-0.01
Hypertensive disorder	61.7	60.0	0.03	60.4	60.7	-0.01
Lesion of liver	1.0	0.7	0.04	1.0	0.7	0.03
Obesity	8.9	9.4	-0.02	8.4	9.8	-0.05
Osteoarthritis	10.9	10.7	0.00	10.3	11.3	-0.03
Pneumonia	1.0	1.1	-0.01	1.0	1.0	0.00
Psoriasis	0.8	0.7	0.01	0.8	0.7	0.02
Renal impairment	6.2	6.8	-0.03	6.0	6.9	-0.04
Rheumatoid arthritis	0.8	0.8	0.00	0.7	0.9	-0.02
Schizophrenia	0.3	<0.2	0.04	0.2	0.2	0.01
Ulcerative colitis	<0.2	0.2	0.00	<0.2	0.2	-0.03
Urinary tract infectious disease	2.1	2.0	0.01	1.9	2.1	-0.01
Viral hepatitis C	0.5	0.2	0.05	0.5	0.2	0.04
Visual system disorder	11.3	10.9	0.01	10.9	11.2	-0.01
Medical history: Cardiovascular disease						
Atrial fibrillation	5.2	5.2	0.00	5.5	5.0	0.02
Cerebrovascular disease	5.1	5.3	-0.01	5.5	5.2	0.01
Coronary arteriosclerosis	12.4	13.3	-0.03	12.9	12.9	0.00
Heart disease	26.0	29.0	-0.07	27.4	27.5	0.00
Heart failure	3.1	5.1	-0.10	3.4	4.6	-0.06
Ischemic heart disease	3.2	3.5	-0.02	3.1	3.6	-0.03
Peripheral vascular disease	3.8	3.8	0.00	3.7	3.5	0.01
Pulmonary embolism	0.3	0.4	-0.02	0.3	0.3	0.00
Venous thrombosis	0.5	0.8	-0.04	0.4	0.9	-0.05
Medical history: Neoplasms						
Hematologic neoplasm	1.5	2.0	-0.04	1.6	1.8	-0.02
Malignant lymphoma	0.9	1.2	-0.02	1.1	1.0	0.00
Malignant neoplasm of anorectum	<0.2	0.3	-0.05	<0.2	0.3	-0.04
Malignant neoplastic disease	8.3	10.4	-0.07	8.9	9.7	-0.03
Malignant tumor of breast	1.2	2.0	-0.06	1.4	1.8	-0.03
Malignant tumor of colon	0.2	0.3	-0.01	0.3	0.3	0.00
Malignant tumor of lung	0.4	0.5	-0.02	0.5	0.5	0.00

(Continued)	Before stratification			After stratification		
Characteristic	T (%)	C (%)	StdDiff	T (%)	C (%)	StdDiff
Malignant tumor of urinary bladder	0.4	0.6	-0.03	0.5	0.6	-0.02
Primary malignant neoplasm of prostate	1.4	1.5	-0.01	1.4	1.5	-0.01
Medication use						
Antibacterials for systemic use	23.4	27.4	-0.09	25.2	25.2	0.00
Antidepressants	14.7	15.4	-0.02	14.6	15.1	-0.01
Antiepileptics	13.4	13.0	0.01	12.9	13.0	0.00
Antiinflammatory and antirheumatic products	17.1	16.8	0.01	16.8	16.8	0.00
Antineoplastic agents	2.9	3.4	-0.03	3.2	3.1	0.01
Antipsoriatics	0.6	0.6	0.00	0.6	0.6	-0.01
Antithrombotic agents	23.2	20.3	0.07	21.9	21.7	0.01
Diuretics	<0.2	<0.2	0.00	<0.2	<0.2	0.00
Drugs for acid related disorders	21.7	22.8	-0.03	21.9	23.2	-0.03
Drugs for obstructive airway diseases	11.7	15.5	-0.11	13.7	13.7	0.00
Drugs used in diabetes	25.7	19.6	0.15	22.1	22.7	-0.01
Immunosuppressants	5.3	5.3	0.00	5.5	5.3	0.01
Lipid modifying agents	45.8	40.8	0.10	43.1	43.6	-0.01
Opioids	10.7	9.8	0.03	10.6	10.3	0.01
Psycholeptics	14.6	13.9	0.02	14.3	13.3	0.03
Psychostimulants, agents used for adhd and nootropics	1.6	1.4	0.01	1.8	1.4	0.03

Supplementary Table 52: Baseline patient characteristcs for ACEI +combo (T) and ARB +combo (C) prevalent-users in the CUIMC data source. We report proportion of initiators satisfying selected base-line characteristics and the standardized difference of population proportions (StdDiff) before and after stratification. Less extreme StdDiffs through stratification suggest improved balance between patient cohorts through propensity score adjustment.

	Before stratification			After stratification		
Characteristic	T (%)	C (%)	StdDiff	T (%)	C (%)	StdDiff
Age group						
15-19	0.2	0.0	0.06	0.2	0.0	0.05
20-24	0.4	0.2	0.03	0.4	0.2	0.03
25-29	0.5	0.3	0.04	0.5	0.3	0.03
30-34	1.1	0.7	0.04	1.0	0.7	0.03
35-39	1.7	1.2	0.04	1.4	1.2	0.02
40-44	2.6	2.1	0.03	2.5	2.2	0.02
45-49	4.2	3.4	0.04	3.8	3.6	0.01
50-54	6.8	5.7	0.05	6.3	6.0	0.01
55-59	9.9	9.0	0.03	9.3	9.3	0.00
60-64	12.3	12.3	0.00	12.0	12.5	-0.02
65-69	14.5	14.4	0.00	14.3	14.7	-0.01
70-74	15.5	16.0	-0.01	15.7	15.9	-0.01
75-79	12.4	13.8	-0.04	13.3	13.2	0.00
80-84	9.1	10.6	-0.05	10.1	10.0	0.00
85-89	5.5	6.2	-0.03	5.8	6.1	-0.01
90-94	2.6	3.3	-0.04	2.7	3.1	-0.03
95-99	0.7	0.8	-0.01	0.7	0.8	-0.01
100-104	<0.1	0.1	-0.01	<0.1	0.1	-0.01
10-14	0.0	0.0		0.0	0.0	
Gender: female	46.1	52.6	-0.13	52.4	48.9	0.07
Race						
race = Asian	0.9	1.8	-0.08	1.3	1.5	-0.02
race = Black or African American	9.2	8.9	0.01	9.0	8.9	0.00
race = Other Race	0.1	0.1	0.00	0.2	0.1	0.01
race = White	33.4	34.7	-0.03	34.5	34.0	0.01
race = Unknown	2.1	2.1	0.00	2.2	2.1	0.01
race = Native Hawaiian or Other Pacific Islander	<0.1	0.0	-0.01	<0.1	0.0	-0.01
race = American Indian or Alaska Native	0.1	0.1	0.00	0.1	0.1	0.00
race = Asian Indian	0.1	0.3	-0.04	0.1	0.2	-0.04
race = Bangladeshi	0.0	0.0		0.0	0.0	
race = Chinese	<0.1	0.1	-0.02	<0.1	0.1	-0.03
race = Filipino	<0.1	0.0	-0.02	<0.1	0.1	-0.02
race = Indonesian	<0.1	0.0	0.00	<0.1	0.0	0.01
race = Japanese	<0.1	0.0		<0.1	0.0	
race = Korean	<0.1	0.1	-0.03	<0.1	0.1	-0.03
race = Laotian	0.7	0.4	0.04	0.6	0.5	0.01
race = Pakistani	0.0	0.0		0.0	0.0	
race = Sri Lankan	<0.1	0.0		<0.1	0.0	
race = Thai	0.0	0.0		0.0	0.0	

(Continued)	Before stratification			After stratification		
Characteristic	T (%)	C (%)	StdDiff	T (%)	C (%)	StdDiff
race = Vietnamese	<0.1	0.0		<0.1	0.0	
race = Polynesian	0.0	0.0		0.0	0.0	
race = Micronesian	<0.1	0.0	-0.01	<0.1	0.0	-0.01
race = Other Pacific Islander	0.7	0.6	0.02	0.7	0.6	0.01
Ethnicity						
ethnicity = Hispanic or Latino	15.7	11.5	0.12	12.8	13.5	-0.02
ethnicity = Not Hispanic or Latino	32.4	36.5	-0.09	35.0	34.5	0.01
Medical history: General						
Acute respiratory disease	4.8	4.6	0.01	4.7	4.6	0.01
Attention deficit hyperactivity disorder	0.2	0.1	0.02	0.2	0.1	0.01
Chronic liver disease	1.5	1.0	0.05	1.3	1.1	0.01
Chronic obstructive lung disease	4.1	4.2	-0.01	3.8	4.2	-0.02
Crohn’s disease	0.2	0.3	-0.01	0.2	0.3	-0.01
Dementia	2.7	2.1	0.04	2.4	2.4	0.00
Depressive disorder	6.3	5.0	0.06	5.5	5.3	0.01
Diabetes mellitus	26.8	23.2	0.08	23.9	24.8	-0.02
Gastroesophageal reflux disease	7.9	8.6	-0.02	8.1	8.4	-0.01
Gastrointestinal hemorrhage	1.5	1.1	0.03	1.3	1.2	0.00
Human immunodeficiency virus infection	2.5	0.9	0.13	1.7	1.3	0.04
Hyperlipidemia	43.3	44.4	-0.02	43.7	44.0	0.00
Hypertensive disorder	69.4	69.2	0.00	69.8	69.4	0.01
Lesion of liver	1.7	1.2	0.05	1.5	1.2	0.02
Obesity	11.0	11.9	-0.03	11.1	11.6	-0.02
Osteoarthritis	13.6	13.5	0.00	13.6	13.6	0.00
Pneumonia	2.2	2.3	-0.01	1.9	2.2	-0.02
Psoriasis	0.7	0.7	0.00	0.7	0.7	0.00
Renal impairment	12.0	12.6	-0.02	11.6	12.5	-0.03
Rheumatoid arthritis	0.8	0.8	0.00	0.8	0.8	0.00
Schizophrenia	0.4	0.1	0.07	0.3	0.1	0.04
Ulcerative colitis	0.2	0.2	0.00	0.1	0.2	-0.01
Urinary tract infectious disease	3.3	3.0	0.01	3.2	3.1	0.00
Viral hepatitis C	1.0	0.6	0.04	0.8	0.8	0.00
Visual system disorder	12.1	11.0	0.03	11.7	11.2	0.02
Medical history: Cardiovascular disease						
Atrial fibrillation	10.7	11.6	-0.03	10.5	11.4	-0.03
Cerebrovascular disease	7.1	7.6	-0.02	7.4	7.4	0.00
Coronary arteriosclerosis	21.8	20.9	0.02	20.2	21.3	-0.03
Heart disease	41.3	43.0	-0.04	40.1	42.7	-0.05
Heart failure	10.7	12.4	-0.05	9.6	11.9	-0.08
Ischemic heart disease	8.6	8.3	0.01	7.6	8.7	-0.04
Peripheral vascular disease	5.9	5.8	0.00	5.7	5.9	-0.01
Pulmonary embolism	0.8	0.8	0.01	0.8	0.8	0.00
Venous thrombosis	1.5	1.2	0.03	1.4	1.3	0.01
Medical history: Neoplasms						
Hematologic neoplasm	1.8	1.7	0.01	1.7	1.7	0.00

(Continued)	Before stratification			After stratification		
Characteristic	T (%)	C (%)	StdDiff	T (%)	C (%)	StdDiff
Malignant lymphoma	1.1	1.2	-0.01	1.1	1.1	0.00
Malignant neoplasm of anorectum	0.2	0.3	-0.01	0.2	0.3	-0.02
Malignant neoplastic disease	10.2	10.1	0.00	10.2	10.1	0.00
Malignant tumor of breast	1.5	1.5	0.00	1.6	1.4	0.02
Malignant tumor of colon	0.4	0.4	0.00	0.4	0.4	0.00
Malignant tumor of lung	0.8	0.6	0.03	0.8	0.6	0.02
Malignant tumor of urinary bladder	0.6	0.6	-0.01	0.6	0.6	0.00
Primary malignant neoplasm of prostate	1.9	1.7	0.02	1.7	1.7	0.00
Medication use						
Antibacterials for systemic use	28.8	31.6	-0.06	30.5	30.1	0.01
Antidepressants	17.3	17.4	0.00	17.5	17.2	0.01
Antiepileptics	17.1	16.4	0.02	16.4	16.6	-0.01
Antiinflammatory and antirheumatic products	20.9	20.6	0.01	21.2	20.5	0.01
Antineoplastic agents	3.8	3.8	0.00	3.8	3.8	0.00
Antipsoriatics	0.9	1.2	-0.02	1.0	1.1	-0.01
Antithrombotic agents	39.2	34.9	0.09	35.2	37.1	-0.04
Diuretics	0.1	0.1	0.02	0.1	0.1	0.02
Drugs for acid related disorders	30.3	31.3	-0.02	30.8	30.9	0.00
Drugs for obstructive airway diseases	16.9	19.7	-0.07	18.8	18.3	0.01
Drugs used in diabetes	28.3	24.9	0.08	25.6	26.4	-0.02
Immunosuppressants	5.5	6.2	-0.03	5.6	5.9	-0.01
Lipid modifying agents	55.3	51.3	0.08	51.9	53.7	-0.04
Opioids	16.0	14.9	0.03	15.3	15.4	0.00
Psycholeptics	18.5	17.6	0.02	18.0	17.8	0.00
Psychostimulants, agents used for adhd and nootropics	1.4	1.4	0.00	1.5	1.3	0.01

Supplementary Table 53: Baseline patient characteristcs for ACEI mono (T) and CCB/THZ mono (C) prevalent-users in the CUIMC data source. We report proportion of initiators satisfying selected base-line characteristics and the standardized difference of population proportions (StdDiff) before and after stratification. Less extreme StdDiffs through stratification suggest improved balance between patient cohorts through propensity score adjustment.

	Before stratification			After stratification		
Characteristic	T (%)	C (%)	StdDiff	T (%)	C (%)	StdDiff
Age group						
15-19	0.3	0.2	0.01	0.4	0.2	0.03
20-24	0.9	0.5	0.05	1.1	0.4	0.08
25-29	1.0	1.1	0.00	1.1	0.9	0.02
30-34	1.6	1.5	0.00	1.8	1.3	0.04
35-39	2.3	2.3	0.00	2.4	2.0	0.03
40-44	3.1	3.5	-0.02	3.0	3.3	-0.02
45-49	5.1	5.3	-0.01	5.1	5.2	0.00
50-54	8.2	7.0	0.04	7.5	7.1	0.01
55-59	10.2	9.0	0.04	9.8	9.2	0.02
60-64	13.1	11.4	0.05	12.5	11.8	0.02
65-69	14.8	13.4	0.04	14.5	13.6	0.03
70-74	14.5	15.0	-0.01	13.8	15.6	-0.05
75-79	11.2	11.9	-0.02	11.2	12.1	-0.03
80-84	7.1	8.4	-0.05	7.8	8.2	-0.02
85-89	4.3	5.5	-0.05	5.0	5.4	-0.02
90-94	1.6	3.0	-0.09	2.0	2.7	-0.04
95-99	0.7	0.8	-0.01	0.9	0.8	0.01
100-104	<0.2	0.3	-0.06	<0.2	0.2	-0.03
Gender: female	47.2	58.7	-0.23	57.0	54.6	0.05
Race						
race = Asian	1.1	1.4	-0.03	1.1	1.4	-0.03
race = Black or African American	6.5	10.8	-0.15	9.2	9.5	-0.01
race = Other Race	<0.2	<0.1	0.03	<0.2	<0.1	0.04
race = White	35.0	31.0	0.09	32.1	32.3	0.00
race = Unknown	1.7	2.3	-0.04	1.9	2.2	-0.02
race = American Indian or Alaska Native	<0.2	<0.1	0.03	<0.2	<0.1	0.04
race = Asian Indian	0.2	0.2	-0.01	<0.2	0.2	-0.03
race = Bangladeshi	0.0	<0.1		0.0	<0.1	
race = Chinese	<0.2	<0.1	0.03	<0.2	<0.1	0.03
race = Filipino	0.0	<0.1		0.0	<0.1	
race = Japanese	<0.2	0.0		<0.2	0.0	
race = Korean	0.0	<0.1		0.0	<0.1	
race = Laotian	0.5	0.5	-0.01	0.3	0.6	-0.03
race = Pakistani	0.0	<0.1		0.0	<0.1	
race = Thai	0.0	<0.1		0.0	<0.1	
race = Vietnamese	<0.2	<0.1	0.01	<0.2	<0.1	0.00
race = Micronesian	<0.2	0.0		<0.2	0.0	
race = Other Pacific Islander	1.1	0.6	0.05	1.4	0.5	0.09
Ethnicity						

(Continued)	Before stratification			After stratification		
Characteristic	T (%)	C (%)	StdDiff	T (%)	C (%)	StdDiff
ethnicity = Hispanic or Latino	14.6	13.9	0.02	15.0	13.9	0.03
ethnicity = Not Hispanic or Latino	32.6	34.4	-0.04	32.9	34.0	-0.03
Medical history: General						
Acute respiratory disease	3.6	4.0	-0.02	4.3	3.9	0.02
Attention deficit hyperactivity disorder	0.2	0.2	0.00	0.2	0.2	0.00
Chronic liver disease	0.9	1.2	-0.03	0.9	1.1	-0.02
Chronic obstructive lung disease	2.4	3.1	-0.04	2.9	2.9	0.00
Crohn’s disease	0.2	0.2	0.00	0.3	0.2	0.02
Dementia	2.1	2.3	-0.01	2.3	2.2	0.01
Depressive disorder	5.3	6.0	-0.03	6.1	5.8	0.01
Diabetes mellitus	24.5	13.2	0.29	16.0	16.0	0.00
Gastroesophageal reflux disease	7.3	7.1	0.01	7.9	7.0	0.03
Gastrointestinal hemorrhage	0.8	1.1	-0.03	0.8	0.9	-0.02
Human immunodeficiency virus infection	3.1	1.7	0.09	2.5	2.0	0.04
Hyperlipidemia	37.6	33.1	0.10	34.2	34.3	0.00
Hypertensive disorder	61.7	69.0	-0.15	67.4	66.3	0.02
Lesion of liver	1.0	1.5	-0.04	1.2	1.4	-0.02
Obesity	8.9	9.8	-0.03	9.3	9.7	-0.01
Osteoarthritis	10.9	12.2	-0.04	12.3	11.9	0.01
Pneumonia	1.0	1.9	-0.07	1.2	1.7	-0.04
Psoriasis	0.8	0.5	0.03	0.7	0.5	0.02
Renal impairment	6.2	9.7	-0.13	8.1	8.8	-0.03
Rheumatoid arthritis	0.8	0.8	0.00	0.9	0.7	0.02
Schizophrenia	0.3	0.3	0.00	0.3	0.3	0.00
Ulcerative colitis	<0.2	0.1	0.01	<0.2	0.1	0.01
Urinary tract infectious disease	2.1	2.8	-0.05	2.7	2.6	0.01
Viral hepatitis C	0.5	0.8	-0.03	0.6	0.7	-0.01
Visual system disorder	11.3	10.2	0.03	10.8	10.1	0.02
Medical history: Cardiovascular disease						
Atrial fibrillation	5.2	4.6	0.03	4.9	4.6	0.02
Cerebrovascular disease	5.1	5.0	0.00	5.3	4.9	0.02
Coronary arteriosclerosis	12.4	10.7	0.05	11.1	11.5	-0.01
Heart disease	26.0	25.0	0.02	25.1	25.3	0.00
Heart failure	3.1	2.4	0.04	3.1	2.5	0.04
Ischemic heart disease	3.2	3.3	-0.01	2.6	3.5	-0.05
Peripheral vascular disease	3.8	3.2	0.03	3.2	3.2	0.00
Pulmonary embolism	0.3	0.5	-0.03	0.4	0.4	-0.01
Venous thrombosis	0.5	1.2	-0.08	0.6	1.0	-0.04
Medical history: Neoplasms						
Hematologic neoplasm	1.5	1.8	-0.02	1.6	1.5	0.00
Malignant lymphoma	0.9	1.3	-0.04	1.2	1.2	0.00
Malignant neoplasm of anorectum	<0.2	0.2	-0.01	<0.2	0.2	-0.01
Malignant neoplastic disease	8.3	10.8	-0.09	9.4	10.1	-0.02
Malignant tumor of breast	1.2	1.7	-0.04	1.7	1.5	0.02
Malignant tumor of colon	0.2	0.5	-0.04	0.3	0.5	-0.02

(Continued)	Before stratification			After stratification		
Characteristic	T (%)	C (%)	StdDiff	T (%)	C (%)	StdDiff
Malignant tumor of lung	0.4	0.6	-0.03	0.6	0.5	0.00
Malignant tumor of urinary bladder	0.4	0.5	-0.02	0.4	0.5	-0.01
Primary malignant neoplasm of prostate	1.4	1.8	-0.03	1.2	1.8	-0.04
Medication use						
Antibacterials for systemic use	23.4	27.1	-0.09	25.3	25.6	0.00
Antidepressants	14.7	15.5	-0.02	14.7	15.2	-0.01
Antiepileptics	13.4	13.0	0.01	13.9	12.8	0.03
Antiinflammatory and antirheumatic products	17.1	20.5	-0.09	19.4	19.5	0.00
Antineoplastic agents	2.9	4.1	-0.06	3.9	3.7	0.01
Antipsoriatics	0.6	1.3	-0.08	0.8	1.2	-0.04
Antithrombotic agents	23.2	22.5	0.02	22.2	22.5	-0.01
Diuretics	<0.2	<0.1	0.00	<0.2	<0.1	-0.01
Drugs for acid related disorders	21.7	26.9	-0.12	24.4	25.4	-0.02
Drugs for obstructive airway diseases	11.7	15.1	-0.10	13.5	14.1	-0.02
Drugs used in diabetes	25.7	12.8	0.33	16.2	16.4	0.00
Immunosuppressants	5.3	7.7	-0.10	6.9	6.8	0.00
Lipid modifying agents	45.8	35.0	0.22	37.3	38.5	-0.03
Opioids	10.7	14.4	-0.11	12.8	13.2	-0.01
Psycholeptics	14.6	15.9	-0.04	15.5	15.6	0.00
Psychostimulants, agents used for adhd and nootropics	1.6	1.5	0.00	1.6	1.5	0.01

Supplementary Table 54: Baseline patient characteristcs for ACEI +combo (T) and CCB/THZ +combo (C) prevalent-users in the CUIMC data source. We report proportion of initiators satisfying selected base-line characteristics and the standardized difference of population proportions (StdDiff) before and after stratification. Less extreme StdDiffs through stratification suggest improved balance between patient cohorts through propensity score adjustment.

	Before stratification			After stratification		
Characteristic	T (%)	C (%)	StdDiff	T (%)	C (%)	StdDiff
Age group						
15-19	0.3	0.1	0.04	0.4	0.1	0.05
20-24	0.5	0.4	0.03	0.7	0.4	0.05
25-29	0.7	0.7	0.00	0.7	0.6	0.02
30-34	1.1	1.3	-0.01	1.2	1.2	0.00
35-39	1.7	1.8	-0.01	1.8	1.7	0.01
40-44	2.5	2.8	-0.02	2.6	2.7	0.00
45-49	4.3	4.1	0.01	4.6	4.0	0.03
50-54	6.4	6.0	0.02	6.2	6.0	0.01
55-59	9.9	8.8	0.04	9.1	9.0	0.00
60-64	12.7	11.5	0.04	11.8	11.7	0.00
65-69	14.6	13.9	0.02	14.2	14.2	0.00
70-74	15.4	15.2	0.01	14.5	15.6	-0.03
75-79	11.9	13.1	-0.04	12.9	12.9	0.00
80-84	8.6	10.0	-0.05	9.4	9.7	-0.01
85-89	5.9	6.1	-0.01	6.1	6.2	0.00
90-94	2.5	3.2	-0.04	3.0	3.1	-0.01
95-99	0.7	0.9	-0.01	0.8	0.9	-0.01
100-104	<0.1	0.2	-0.02	0.1	0.2	-0.01
10-14	0.0	0.0		0.0	0.0	
Gender: female	44.3	56.6	-0.25	55.1	53.4	0.04
Race						
race = Asian	1.0	1.5	-0.04	1.1	1.4	-0.03
race = Black or African American	7.8	11.4	-0.12	10.2	10.5	-0.01
race = Other Race	0.1	0.1	0.00	0.2	0.1	0.02
race = White	36.5	31.8	0.10	32.5	33.0	-0.01
race = Unknown	2.0	2.2	-0.01	2.0	2.2	-0.01
race = Native Hawaiian or Other Pacific Islander	0.0	0.0		0.0	0.0	
race = American Indian or Alaska Native	0.1	0.1	0.02	0.1	0.1	0.03
race = Asian Indian	0.2	0.3	-0.03	0.1	0.3	-0.04
race = Bangladeshi	0.0	0.0		0.0	0.0	
race = Chinese	<0.1	0.0	0.00	<0.1	0.0	0.00
race = Filipino	<0.1	0.0	-0.01	<0.1	0.0	-0.02
race = Indonesian	<0.1	0.0		<0.1	0.0	
race = Japanese	<0.1	0.0	0.01	<0.1	0.0	0.02
race = Korean	<0.1	0.1	-0.02	<0.1	0.1	-0.02
race = Laotian	0.6	0.6	0.01	0.6	0.6	0.00
race = Pakistani	0.0	0.0		0.0	0.0	
race = Thai	0.0	0.0		0.0	0.0	
race = Vietnamese	<0.1	0.0	0.01	<0.1	0.0	0.01

(Continued)	Before stratification			After stratification		
Characteristic	T (%)	C (%)	StdDiff	T (%)	C (%)	StdDiff
race = Polynesian	0.0	0.0		0.0	0.0	
race = Micronesian	<0.1	0.0	0.01	<0.1	0.0	0.01
race = Other Pacific Islander	0.8	0.7	0.02	1.0	0.6	0.04
Ethnicity						
ethnicity = Hispanic or Latino	14.1	13.7	0.01	14.1	13.5	0.02
ethnicity = Not Hispanic or Latino	34.5	35.2	-0.01	34.5	35.2	-0.02
Medical history: General						
Acute respiratory disease	4.6	5.5	-0.04	5.0	5.1	0.00
Attention deficit hyperactivity disorder	0.2	0.2	0.00	0.2	0.2	0.00
Chronic liver disease	1.5	1.6	0.00	1.5	1.5	0.00
Chronic obstructive lung disease	4.0	4.2	-0.01	4.1	4.1	0.00
Crohn’s disease	0.2	0.3	-0.01	0.2	0.3	0.00
Dementia	2.5	2.6	-0.01	2.8	2.5	0.02
Depressive disorder	5.8	6.5	-0.03	6.5	6.3	0.01
Diabetes mellitus	26.7	21.0	0.13	20.7	22.1	-0.03
Gastroesophageal reflux disease	8.0	9.1	-0.04	9.1	8.7	0.01
Gastrointestinal hemorrhage	1.4	1.5	-0.01	1.6	1.4	0.01
Human immunodeficiency virus infection	2.4	1.5	0.07	1.9	1.6	0.02
Hyperlipidemia	43.9	40.7	0.06	41.1	41.5	-0.01
Hypertensive disorder	66.6	72.3	-0.12	71.6	71.0	0.01
Lesion of liver	1.6	1.8	-0.01	1.7	1.7	0.00
Obesity	10.3	11.8	-0.04	11.3	11.5	-0.01
Osteoarthritis	11.5	14.3	-0.09	13.9	13.7	0.01
Pneumonia	2.1	3.3	-0.07	2.2	3.0	-0.05
Psoriasis	0.7	0.7	0.01	0.6	0.6	0.00
Renal impairment	10.7	17.2	-0.19	15.2	15.6	-0.01
Rheumatoid arthritis	0.9	0.9	0.00	1.1	0.8	0.02
Schizophrenia	0.3	0.3	0.01	0.3	0.3	0.00
Ulcerative colitis	0.1	0.2	-0.02	0.1	0.2	-0.02
Urinary tract infectious disease	3.1	4.0	-0.04	4.1	3.6	0.02
Viral hepatitis C	0.8	0.9	-0.01	0.7	0.9	-0.02
Visual system disorder	11.3	11.6	-0.01	11.8	11.4	0.01
Medical history: Cardiovascular disease						
Atrial fibrillation	12.4	9.6	0.09	10.4	10.1	0.01
Cerebrovascular disease	7.1	7.3	-0.01	7.3	7.3	0.00
Coronary arteriosclerosis	24.1	17.6	0.16	18.4	18.8	-0.01
Heart disease	45.3	37.3	0.16	37.3	38.7	-0.03
Heart failure	13.3	8.4	0.16	9.6	8.8	0.03
Ischemic heart disease	9.6	7.3	0.08	7.2	7.7	-0.02
Peripheral vascular disease	6.0	5.7	0.01	5.3	5.8	-0.02
Pulmonary embolism	0.7	0.9	-0.02	0.9	0.8	0.00
Venous thrombosis	1.3	1.9	-0.05	1.6	1.8	-0.02
Medical history: Neoplasms						
Hematologic neoplasm	1.7	2.0	-0.02	1.8	1.9	-0.01
Malignant lymphoma	1.2	1.4	-0.02	1.2	1.3	-0.01

(Continued)	Before stratification			After stratification		
Characteristic	T (%)	C (%)	StdDiff	T (%)	C (%)	StdDiff
Malignant neoplasm of anorectum	0.1	0.3	-0.03	0.1	0.2	-0.02
Malignant neoplastic disease	9.8	11.2	-0.05	10.8	10.8	0.00
Malignant tumor of breast	1.4	1.4	-0.01	1.9	1.3	0.04
Malignant tumor of colon	0.3	0.5	-0.03	0.4	0.5	-0.02
Malignant tumor of lung	0.7	0.8	0.00	1.0	0.7	0.03
Malignant tumor of urinary bladder	0.6	0.6	-0.01	0.7	0.6	0.00
Primary malignant neoplasm of prostate	1.7	1.7	0.00	1.4	1.8	-0.03
Medication use						
Antibacterials for systemic use	28.4	33.4	-0.11	31.5	32.0	-0.01
Antidepressants	16.9	17.9	-0.03	17.7	17.6	0.00
Antiepileptics	16.2	17.3	-0.03	17.2	16.8	0.01
Antiinflammatory and antirheumatic products	18.7	21.5	-0.07	21.4	20.8	0.01
Antineoplastic agents	3.2	4.5	-0.07	3.8	4.2	-0.02
Antipsoriatics	0.8	2.0	-0.11	1.2	1.8	-0.04
Antithrombotic agents	38.7	34.8	0.08	34.2	35.3	-0.02
Diuretics	0.1	0.2	-0.02	0.2	0.2	0.00
Drugs for acid related disorders	29.1	33.9	-0.10	32.2	32.5	-0.01
Drugs for obstructive airway diseases	16.1	19.7	-0.09	18.4	18.7	-0.01
Drugs used in diabetes	28.0	21.8	0.14	21.4	23.1	-0.04
Immunosuppressants	5.6	9.2	-0.14	8.0	8.3	-0.01
Lipid modifying agents	55.1	46.6	0.17	46.9	48.6	-0.03
Opioids	14.8	19.1	-0.11	17.2	17.9	-0.02
Psycholeptics	18.1	20.2	-0.05	19.5	19.5	0.00
Psychostimulants, agents used for adhd and nootropics	1.5	1.6	-0.01	1.8	1.6	0.01

Supplementary Table 55: Baseline patient characteristcs for ACEI mono (T) and CCB mono (C) prevalent-users in the CUIMC data source. We report proportion of initiators satisfying selected base-line characteristics and the standardized difference of population proportions (StdDiff) before and after stratification. Less extreme StdDiffs through stratification suggest improved balance between patient cohorts through propensity score adjustment.

	Before stratification			After stratification		
Characteristic	T (%)	C (%)	StdDiff	T (%)	C (%)	StdDiff
Age group						
15-19	0.3	0.3	0.00	0.4	0.3	0.02
20-24	0.9	0.7	0.02	1.1	0.6	0.06
25-29	1.0	1.4	-0.04	1.1	1.1	0.00
30-34	1.6	1.9	-0.02	1.8	1.5	0.03
35-39	2.3	2.8	-0.03	2.4	2.3	0.00
40-44	3.1	3.4	-0.02	3.2	3.2	0.00
45-49	5.1	5.0	0.00	5.3	4.9	0.02
50-54	8.2	6.5	0.06	7.5	6.7	0.03
55-59	10.2	8.3	0.07	9.2	8.6	0.02
60-64	13.1	10.8	0.07	12.0	11.4	0.02
65-69	14.8	12.8	0.06	14.1	13.2	0.03
70-74	14.5	15.3	-0.02	14.0	16.1	-0.06
75-79	11.2	11.7	-0.02	11.2	12.2	-0.03
80-84	7.1	8.9	-0.07	8.1	8.5	-0.01
85-89	4.3	5.9	-0.07	5.2	5.6	-0.02
90-94	1.6	3.1	-0.10	2.2	2.8	-0.04
95-99	0.7	0.9	-0.02	0.9	0.8	0.01
100-104	<0.2	0.4	-0.08	<0.2	0.3	-0.05
Gender: female	47.2	56.7	-0.19	54.3	52.3	0.04
Race						
race = Asian	1.1	1.8	-0.06	1.0	1.7	-0.06
race = Black or African American	6.5	11.0	-0.16	8.8	9.2	-0.01
race = Other Race	<0.2	<0.1	0.03	<0.2	<0.1	0.03
race = White	35.0	31.2	0.08	32.5	32.7	0.00
race = Unknown	1.7	1.9	-0.02	1.8	1.9	-0.01
race = American Indian or Alaska Native	<0.2	<0.1	0.01	<0.2	<0.1	0.01
race = Asian Indian	0.2	0.3	-0.02	<0.2	0.3	-0.04
race = Bangladeshi	0.0	<0.1		0.0	<0.1	
race = Chinese	<0.2	<0.1	0.02	<0.2	<0.1	0.03
race = Filipino	0.0	<0.1		0.0	<0.1	
race = Japanese	<0.2	0.0		<0.2	0.0	
race = Korean	0.0	<0.1		0.0	<0.1	
race = Laotian	0.5	0.5	-0.01	0.4	0.5	-0.03
race = Pakistani	0.0	<0.1		0.0	<0.1	
race = Vietnamese	<0.2	<0.1	0.01	<0.2	<0.1	0.00
race = Micronesian	<0.2	0.0		<0.2	0.0	
race = Other Pacific Islander	1.1	0.7	0.05	1.4	0.6	0.08
Ethnicity						
ethnicity = Hispanic or Latino	14.6	14.0	0.02	14.6	14.1	0.01

(Continued)	Before stratification			After stratification		
Characteristic	T (%)	C (%)	StdDiff	T (%)	C (%)	StdDiff
ethnicity = Not Hispanic or Latino	32.6	34.6	-0.04	32.9	33.9	-0.02
Medical history: General						
Acute respiratory disease	3.6	4.2	-0.03	4.7	3.9	0.04
Attention deficit hyperactivity disorder	0.2	0.2	0.00	0.2	0.3	-0.01
Chronic liver disease	0.9	1.3	-0.03	0.9	1.1	-0.02
Chronic obstructive lung disease	2.4	3.6	-0.07	3.0	3.2	-0.01
Crohn's disease	0.2	0.2	0.00	0.3	0.2	0.03
Dementia	2.1	2.7	-0.04	2.3	2.5	-0.01
Depressive disorder	5.3	6.0	-0.03	6.0	5.6	0.02
Diabetes mellitus	24.5	14.3	0.26	17.4	17.4	0.00
Gastroesophageal reflux disease	7.3	7.1	0.01	8.0	6.9	0.04
Gastrointestinal hemorrhage	0.8	1.2	-0.04	0.9	1.0	-0.01
Human immunodeficiency virus infection	3.1	1.8	0.08	2.8	2.0	0.05
Hyperlipidemia	37.6	31.9	0.12	34.1	34.0	0.00
Hypertensive disorder	61.7	68.7	-0.15	65.8	65.4	0.01
Lesion of liver	1.0	1.7	-0.06	1.2	1.5	-0.03
Obesity	8.9	9.0	0.00	8.9	9.1	0.00
Osteoarthritis	10.9	11.9	-0.03	12.4	11.6	0.03
Pneumonia	1.0	2.5	-0.11	1.5	2.0	-0.04
Psoriasis	0.8	0.6	0.02	0.7	0.5	0.03
Renal impairment	6.2	11.5	-0.19	8.1	9.5	-0.05
Rheumatoid arthritis	0.8	0.8	0.00	1.0	0.7	0.03
Schizophrenia	0.3	0.3	0.00	0.3	0.2	0.01
Ulcerative colitis	<0.2	<0.1	0.02	<0.2	<0.1	0.02
Urinary tract infectious disease	2.1	3.2	-0.07	2.6	2.8	-0.01
Viral hepatitis C	0.5	0.8	-0.03	0.6	0.7	-0.01
Visual system disorder	11.3	10.1	0.04	11.2	10.0	0.04
Medical history: Cardiovascular disease						
Atrial fibrillation	5.2	4.4	0.04	5.2	4.3	0.04
Cerebrovascular disease	5.1	5.4	-0.01	5.4	5.0	0.01
Coronary arteriosclerosis	12.4	11.8	0.02	11.7	12.4	-0.02
Heart disease	26.0	26.3	-0.01	26.0	26.1	0.00
Heart failure	3.1	2.5	0.03	3.2	2.6	0.04
Ischemic heart disease	3.2	3.8	-0.03	2.8	3.9	-0.06
Peripheral vascular disease	3.8	3.6	0.01	3.6	3.6	0.00
Pulmonary embolism	0.3	0.5	-0.04	0.4	0.4	0.00
Venous thrombosis	0.5	1.3	-0.09	0.7	1.1	-0.04
Medical history: Neoplasms						
Hematologic neoplasm	1.5	1.8	-0.03	1.8	1.5	0.02
Malignant lymphoma	0.9	1.4	-0.04	1.2	1.2	0.00
Malignant neoplasm of anorectum	<0.2	0.2	-0.01	<0.2	0.2	-0.01
Malignant neoplastic disease	8.3	11.1	-0.10	9.3	10.0	-0.02
Malignant tumor of breast	1.2	1.4	-0.01	1.5	1.3	0.02
Malignant tumor of colon	0.2	0.5	-0.04	0.3	0.4	-0.02
Malignant tumor of lung	0.4	0.8	-0.05	0.6	0.7	-0.01

(Continued)	Before stratification			After stratification		
Characteristic	T (%)	C (%)	StdDiff	T (%)	C (%)	StdDiff
Malignant tumor of urinary bladder	0.4	0.4	0.00	0.4	0.4	0.01
Primary malignant neoplasm of prostate	1.4	1.8	-0.03	1.2	1.7	-0.04
Medication use						
Antibacterials for systemic use	23.4	29.1	-0.13	25.8	26.1	0.00
Antidepressants	14.7	15.8	-0.03	15.0	15.1	0.00
Antiepileptics	13.4	13.3	0.00	13.8	12.7	0.03
Antiinflammatory and antirheumatic products	17.1	20.2	-0.08	19.1	18.7	0.01
Antineoplastic agents	2.9	4.5	-0.08	3.9	3.8	0.01
Antipsoriatics	0.6	1.5	-0.09	0.8	1.2	-0.04
Antithrombotic agents	23.2	25.7	-0.06	24.3	24.4	0.00
Diuretics	<0.2	<0.1	0.01	<0.2	<0.1	0.00
Drugs for acid related disorders	21.7	28.8	-0.16	24.6	26.1	-0.04
Drugs for obstructive airway diseases	11.7	15.5	-0.11	13.6	14.0	-0.01
Drugs used in diabetes	25.7	13.0	0.32	17.5	17.6	0.00
Immunosuppressants	5.3	9.5	-0.16	7.5	7.7	-0.01
Lipid modifying agents	45.8	35.1	0.22	38.8	39.7	-0.02
Opioids	10.7	15.9	-0.15	13.1	13.8	-0.02
Psycholeptics	14.6	16.9	-0.06	15.9	15.8	0.00
Psychostimulants, agents used for adhd and nootropics	1.6	1.6	-0.01	1.6	1.6	0.00

Supplementary Table 56: Baseline patient characteristcs for ACEI +combo (T) and CCB +combo (C) prevalent-users in the CUIMC data source. We report proportion of initiators satisfying selected base-line characteristics and the standardized difference of population proportions (StdDiff) before and after stratification. Less extreme StdDiffs through stratification suggest improved balance between patient cohorts through propensity score adjustment.

	Before stratification			After stratification		
Characteristic	T (%)	C (%)	StdDiff	T (%)	C (%)	StdDiff
Age group						
15-19	0.2	0.1	0.02	0.3	0.1	0.04
20-24	0.5	0.5	-0.01	0.6	0.4	0.02
25-29	0.6	0.9	-0.04	0.7	0.7	0.00
30-34	1.1	1.5	-0.04	1.1	1.3	-0.01
35-39	1.8	2.4	-0.04	1.7	2.0	-0.02
40-44	2.6	2.8	-0.02	2.8	2.7	0.00
45-49	4.4	4.1	0.02	4.5	4.0	0.02
50-54	6.7	5.8	0.04	6.1	6.0	0.00
55-59	10.1	8.4	0.06	9.1	8.9	0.01
60-64	12.8	11.2	0.05	11.6	11.8	0.00
65-69	14.5	13.5	0.03	13.9	13.9	0.00
70-74	15.6	15.0	0.02	14.7	15.5	-0.02
75-79	11.9	12.7	-0.02	12.6	12.6	0.00
80-84	8.7	10.0	-0.04	9.9	9.6	0.01
85-89	5.4	6.6	-0.05	6.4	6.2	0.01
90-94	2.4	3.3	-0.06	2.9	3.1	-0.01
95-99	0.6	1.1	-0.04	0.7	1.0	-0.03
100-104	<0.1	0.2	-0.04	<0.1	0.2	-0.03
10-14	0.0	0.0		0.0	0.0	
Gender: female	46.5	53.9	-0.15	51.9	51.0	0.02
Race						
race = Asian	0.9	1.7	-0.07	1.1	1.5	-0.04
race = Black or African American	8.1	12.2	-0.14	10.3	10.7	-0.01
race = Other Race	0.1	0.1	0.00	0.1	0.1	0.00
race = White	35.1	31.4	0.08	32.6	32.8	0.00
race = Unknown	2.0	2.0	0.00	1.9	2.1	-0.01
race = Native Hawaiian or Other Pacific Islander	<0.1	0.0	0.01	<0.1	0.0	0.00
race = American Indian or Alaska Native	0.1	0.1	0.02	0.2	0.1	0.03
race = Asian Indian	0.1	0.4	-0.04	0.1	0.4	-0.05
race = Bangladeshi	0.0	0.0		0.0	0.0	
race = Chinese	<0.1	0.0	-0.01	<0.1	0.0	-0.01
race = Filipino	<0.1	0.1	-0.02	<0.1	0.1	-0.03
race = Indonesian	<0.1	0.0		<0.1	0.0	
race = Japanese	<0.1	0.0	0.01	<0.1	0.0	0.01
race = Korean	<0.1	0.1	-0.02	<0.1	0.1	-0.02
race = Laotian	0.6	0.7	-0.01	0.7	0.7	0.00
race = Pakistani	0.0	0.0		0.0	0.0	
race = Thai	0.0	0.0		0.0	0.0	
race = Vietnamese	<0.1	0.0	0.01	<0.1	0.0	0.00

(Continued)	Before stratification			After stratification		
Characteristic	T (%)	C (%)	StdDiff	T (%)	C (%)	StdDiff
race = Polynesian	0.0	0.0		0.0	0.0	
race = Micronesian	<0.1	0.0	0.01	<0.1	0.0	0.00
race = Other Pacific Islander	0.7	0.7	0.01	0.9	0.6	0.03
Ethnicity						
ethnicity = Hispanic or Latino	14.6	14.1	0.01	14.4	14.1	0.01
ethnicity = Not Hispanic or Latino	33.3	35.1	-0.04	34.1	34.7	-0.01
Medical history: General						
Acute respiratory disease	4.6	5.9	-0.06	5.4	5.2	0.01
Attention deficit hyperactivity disorder	0.2	0.2	0.00	0.1	0.2	-0.01
Chronic liver disease	1.4	1.7	-0.03	1.5	1.5	0.00
Chronic obstructive lung disease	3.9	4.8	-0.04	4.5	4.4	0.00
Crohn’s disease	0.2	0.3	-0.02	0.2	0.3	-0.01
Dementia	2.2	3.2	-0.06	2.8	2.8	0.00
Depressive disorder	5.8	7.2	-0.05	6.5	6.7	-0.01
Diabetes mellitus	25.5	22.8	0.06	22.6	23.5	-0.02
Gastroesophageal reflux disease	7.9	9.8	-0.07	9.3	9.0	0.01
Gastrointestinal hemorrhage	1.3	1.7	-0.04	1.7	1.5	0.01
Human immunodeficiency virus infection	2.5	1.7	0.06	2.2	1.9	0.02
Hyperlipidemia	42.8	40.5	0.04	41.4	41.3	0.00
Hypertensive disorder	67.7	74.0	-0.14	71.8	71.6	0.00
Lesion of liver	1.5	2.1	-0.04	1.8	1.8	0.00
Obesity	10.9	10.9	0.00	11.0	11.0	0.00
Osteoarthritis	12.1	14.1	-0.06	13.5	13.5	0.00
Pneumonia	2.0	4.1	-0.12	2.4	3.3	-0.05
Psoriasis	0.7	0.6	0.01	0.6	0.6	0.00
Renal impairment	10.3	21.2	-0.30	16.7	17.0	-0.01
Rheumatoid arthritis	0.9	0.9	0.00	1.0	0.8	0.02
Schizophrenia	0.4	0.3	0.00	0.3	0.3	0.00
Ulcerative colitis	0.1	0.2	-0.01	0.2	0.2	-0.01
Urinary tract infectious disease	3.0	4.7	-0.09	4.0	3.9	0.00
Viral hepatitis C	0.8	1.1	-0.03	0.9	1.0	-0.01
Visual system disorder	11.6	12.2	-0.02	12.3	11.9	0.01
Medical history: Cardiovascular disease						
Atrial fibrillation	11.4	9.3	0.07	10.4	9.8	0.02
Cerebrovascular disease	6.7	7.8	-0.04	7.5	7.4	0.00
Coronary arteriosclerosis	22.0	19.2	0.07	19.9	19.8	0.00
Heart disease	42.3	39.2	0.06	39.3	39.6	-0.01
Heart failure	11.7	9.1	0.08	10.1	9.2	0.03
Ischemic heart disease	8.8	8.2	0.02	7.6	8.3	-0.02
Peripheral vascular disease	5.7	6.3	-0.03	5.9	6.1	-0.01
Pulmonary embolism	0.8	0.9	-0.01	0.9	0.8	0.01
Venous thrombosis	1.4	2.3	-0.07	1.8	1.9	-0.01
Medical history: Neoplasms						
Hematologic neoplasm	1.7	2.3	-0.04	2.1	2.0	0.00
Malignant lymphoma	1.1	1.5	-0.04	1.2	1.4	-0.01

(Continued)	Before stratification			After stratification		
Characteristic	T (%)	C (%)	StdDiff	T (%)	C (%)	StdDiff
Malignant neoplasm of anorectum	0.2	0.3	-0.02	0.2	0.3	-0.02
Malignant neoplastic disease	9.6	12.1	-0.08	10.8	11.1	-0.01
Malignant tumor of breast	1.6	1.4	0.01	1.8	1.3	0.04
Malignant tumor of colon	0.4	0.6	-0.03	0.4	0.5	-0.01
Malignant tumor of lung	0.7	0.9	-0.03	0.9	0.8	0.01
Malignant tumor of urinary bladder	0.6	0.7	-0.01	0.7	0.6	0.01
Primary malignant neoplasm of prostate	1.6	1.8	-0.02	1.7	1.7	0.00
Medication use						
Antibacterials for systemic use	28.2	35.0	-0.15	31.8	32.2	-0.01
Antidepressants	17.3	18.9	-0.04	18.1	18.3	0.00
Antiepileptics	16.3	18.4	-0.06	17.6	17.4	0.01
Antiinflammatory and antirheumatic products	20.2	21.6	-0.04	21.6	21.0	0.01
Antineoplastic agents	3.2	5.0	-0.09	4.0	4.3	-0.02
Antipsoriatics	0.7	2.5	-0.14	1.1	2.0	-0.07
Antithrombotic agents	37.1	39.0	-0.04	37.3	37.9	-0.01
Diuretics	0.1	0.1	0.00	0.2	0.1	0.02
Drugs for acid related disorders	28.8	36.8	-0.17	33.5	33.6	0.00
Drugs for obstructive airway diseases	16.4	20.5	-0.11	18.7	18.8	0.00
Drugs used in diabetes	27.3	22.8	0.10	22.8	24.5	-0.04
Immunosuppressants	5.4	11.3	-0.22	8.2	9.2	-0.03
Lipid modifying agents	54.0	47.8	0.12	49.3	50.2	-0.02
Opioids	14.7	21.8	-0.18	18.1	19.1	-0.03
Psycholeptics	17.4	21.7	-0.11	19.6	19.9	-0.01
Psychostimulants, agents used for adhd and nootropics	1.5	1.7	-0.02	1.7	1.6	0.01

Supplementary Table 57: Baseline patient characteristcs for ACEI mono (T) and THZ mono (C) prevalent-users in the CUIMC data source. We report proportion of initiators satisfying selected base-line characteristics and the standardized difference of population proportions (StdDiff) before and after stratification. Less extreme StdDiffs through stratification suggest improved balance between patient cohorts through propensity score adjustment.

	Before stratification			After stratification		
Characteristic	T (%)	C (%)	StdDiff	T (%)	C (%)	StdDiff
Age group						
15-19	0.3	<0.3	0.04	0.4	<0.3	0.05
20-24	0.9	<0.3	0.10	0.9	<0.3	0.11
25-29	1.0	0.3	0.08	0.9	<0.3	0.08
30-34	1.6	0.7	0.08	1.6	0.6	0.10
35-39	2.3	1.1	0.09	2.3	1.0	0.10
40-44	3.1	3.8	-0.04	2.8	3.2	-0.02
45-49	5.1	5.8	-0.03	5.0	5.1	0.00
50-54	8.2	8.1	0.00	8.2	7.8	0.02
55-59	10.2	10.6	-0.01	10.7	10.2	0.02
60-64	13.1	12.5	0.02	13.0	13.0	0.00
65-69	14.8	14.6	0.00	15.0	15.0	0.00
70-74	14.5	13.9	0.02	14.1	14.6	-0.01
75-79	11.2	12.8	-0.05	11.6	13.2	-0.05
80-84	7.1	7.6	-0.02	6.8	8.5	-0.06
85-89	4.3	4.9	-0.03	4.3	4.6	-0.01
90-94	1.6	2.4	-0.06	1.6	2.2	-0.04
95-99	0.7	0.5	0.03	0.8	0.7	0.02
100-104	<0.2	0.0		<0.2	0.0	
Gender: female	47.2	63.3	-0.33	54.6	49.9	0.10
Race						
race = Asian	1.1	0.9	0.02	1.0	0.6	0.04
race = Black or African American	6.5	9.8	-0.12	7.4	7.8	-0.02
race = Other Race	<0.2	0.0		0.2	0.0	
race = White	35.0	31.6	0.07	33.8	33.9	0.00
race = Unknown	1.7	3.0	-0.08	1.8	2.6	-0.06
race = American Indian or Alaska Native	<0.2	<0.3	0.03	<0.2	<0.3	0.04
race = Asian Indian	0.2	<0.3	0.04	<0.2	<0.3	0.03
race = Chinese	<0.2	0.0		<0.2	0.0	
race = Japanese	<0.2	0.0		<0.2	0.0	
race = Laotian	0.5	0.6	-0.02	0.4	1.0	-0.07
race = Vietnamese	<0.2	0.0		<0.2	0.0	
race = Micronesian	<0.2	0.0		<0.2	0.0	
race = Other Pacific Islander	1.1	0.3	0.09	1.3	0.4	0.10
Ethnicity						
ethnicity = Hispanic or Latino	14.6	14.2	0.01	14.7	15.0	-0.01
ethnicity = Not Hispanic or Latino	32.6	33.9	-0.03	32.5	34.2	-0.04
Medical history: General						
Acute respiratory disease	3.6	3.7	0.00	3.6	3.8	-0.01
Attention deficit hyperactivity disorder	0.2	<0.3	0.02	0.3	<0.3	0.04

(Continued)	Before stratification			After stratification		
Characteristic	T (%)	C (%)	StdDiff	T (%)	C (%)	StdDiff
Chronic liver disease	0.9	1.0	-0.01	0.9	1.3	-0.04
Chronic obstructive lung disease	2.4	2.0	0.03	2.4	2.2	0.01
Crohn’s disease	0.2	<0.3	0.01	0.2	<0.3	0.02
Dementia	2.1	1.3	0.06	2.1	1.4	0.06
Depressive disorder	5.3	5.7	-0.02	5.6	5.9	-0.01
Diabetes mellitus	24.5	10.7	0.37	19.2	20.1	-0.02
Gastroesophageal reflux disease	7.3	6.3	0.04	7.5	6.2	0.05
Gastrointestinal hemorrhage	0.8	0.5	0.04	0.7	0.3	0.05
Human immunodeficiency virus infection	3.1	1.4	0.11	2.8	1.9	0.06
Hyperlipidemia	37.6	33.7	0.08	35.6	36.5	-0.02
Hypertensive disorder	61.7	65.7	-0.08	63.3	63.5	0.00
Lesion of liver	1.0	1.2	-0.01	1.1	1.4	-0.03
Obesity	8.9	11.7	-0.09	9.7	10.5	-0.03
Osteoarthritis	10.9	12.7	-0.06	11.2	12.2	-0.03
Pneumonia	1.0	0.8	0.02	0.8	1.0	-0.01
Psoriasis	0.8	0.5	0.04	0.7	0.5	0.02
Renal impairment	6.2	5.6	0.03	5.5	8.0	-0.10
Rheumatoid arthritis	0.8	0.6	0.03	0.7	0.6	0.01
Schizophrenia	0.3	<0.3	0.05	0.3	<0.3	0.05
Ulcerative colitis	<0.2	0.0		<0.2	0.0	
Urinary tract infectious disease	2.1	1.8	0.02	2.1	1.7	0.03
Viral hepatitis C	0.5	0.5	0.00	0.5	0.7	-0.03
Visual system disorder	11.3	10.5	0.03	11.0	10.5	0.02
Medical history: Cardiovascular disease						
Atrial fibrillation	5.2	5.3	-0.01	4.9	5.7	-0.04
Cerebrovascular disease	5.1	3.9	0.06	4.9	4.2	0.03
Coronary arteriosclerosis	12.4	8.8	0.12	11.2	11.4	-0.01
Heart disease	26.0	21.9	0.10	24.5	24.7	0.00
Heart failure	3.1	2.1	0.06	2.9	2.8	0.01
Ischemic heart disease	3.2	2.4	0.05	2.7	3.6	-0.05
Peripheral vascular disease	3.8	2.6	0.07	3.3	3.4	0.00
Pulmonary embolism	0.3	0.5	-0.04	0.3	0.5	-0.03
Venous thrombosis	0.5	0.5	0.00	0.5	0.6	-0.01
Medical history: Neoplasms						
Hematologic neoplasm	1.5	1.3	0.01	1.2	1.3	-0.01
Malignant lymphoma	0.9	1.1	-0.01	0.8	1.0	-0.01
Malignant neoplasm of anorectum	<0.2	<0.3	0.00	<0.2	<0.3	0.01
Malignant neoplastic disease	8.3	9.5	-0.04	8.3	9.3	-0.04
Malignant tumor of breast	1.2	2.2	-0.08	1.5	1.5	0.00
Malignant tumor of colon	0.2	0.3	-0.02	0.3	0.3	0.00
Malignant tumor of lung	0.4	<0.3	0.02	0.4	0.3	0.02
Malignant tumor of urinary bladder	0.4	0.7	-0.04	0.4	0.9	-0.06
Primary malignant neoplasm of prostate	1.4	1.6	-0.02	1.2	1.9	-0.06
Medication use						
Antibacterials for systemic use	23.4	22.2	0.03	22.8	21.1	0.04

(Continued)	Before stratification			After stratification		
Characteristic	T (%)	C (%)	StdDiff	T (%)	C (%)	StdDiff
Antidepressants	14.7	14.4	0.01	14.5	14.3	0.01
Antiepileptics	13.4	12.1	0.04	13.1	11.8	0.04
Antiinflammatory and antirheumatic products	17.1	19.7	-0.07	17.9	17.7	0.00
Antineoplastic agents	2.9	3.0	0.00	2.9	2.5	0.02
Antipsoriatics	0.6	0.8	-0.03	0.5	0.7	-0.03
Antithrombotic agents	23.2	14.2	0.23	19.6	19.6	0.00
Diuretics	<0.2	<0.3	-0.01	<0.2	<0.3	-0.03
Drugs for acid related disorders	21.7	21.9	0.00	21.4	22.1	-0.02
Drugs for obstructive airway diseases	11.7	13.7	-0.06	11.7	12.4	-0.02
Drugs used in diabetes	25.7	12.4	0.34	20.4	20.8	-0.01
Immunosuppressants	5.3	3.9	0.07	5.0	4.2	0.04
Lipid modifying agents	45.8	33.4	0.26	40.2	41.7	-0.03
Opioids	10.7	10.5	0.01	10.4	10.6	-0.01
Psycholeptics	14.6	13.4	0.03	14.4	13.2	0.03
Psychostimulants, agents used for adhd and nootropics	1.6	1.2	0.03	1.6	1.0	0.05

Supplementary Table 58: Baseline patient characteristcs for ACEI +combo (T) and THZ +combo (C) prevalent-users in the CUIMC data source. We report proportion of initiators satisfying selected base-line characteristics and the standardized difference of population proportions (StdDiff) before and after stratification. Less extreme StdDiffs through stratification suggest improved balance between patient cohorts through propensity score adjustment.

	Before stratification			After stratification		
Characteristic	T (%)	C (%)	StdDiff	T (%)	C (%)	StdDiff
Age group						
15-19	0.3	0.1	0.05	0.3	0.1	0.05
20-24	0.4	0.1	0.06	0.5	0.1	0.07
25-29	0.6	0.2	0.06	0.6	0.2	0.06
30-34	1.1	0.8	0.03	1.1	0.7	0.04
35-39	1.7	0.9	0.06	1.5	1.1	0.04
40-44	2.5	2.8	-0.02	2.3	2.6	-0.02
45-49	4.1	4.1	0.00	4.4	3.8	0.03
50-54	6.5	6.4	0.01	6.5	6.1	0.01
55-59	9.6	9.6	0.00	9.7	9.5	0.01
60-64	12.2	12.2	0.00	12.2	12.3	0.00
65-69	14.6	14.6	0.00	14.7	15.2	-0.01
70-74	15.5	15.7	0.00	15.2	16.0	-0.02
75-79	12.1	13.6	-0.04	12.6	13.0	-0.01
80-84	9.2	9.9	-0.02	9.2	9.9	-0.03
85-89	6.0	5.6	0.02	5.8	5.6	0.01
90-94	2.7	2.9	-0.01	2.6	3.1	-0.03
95-99	0.8	0.5	0.04	0.8	0.5	0.04
100-104	<0.1	0.1	0.00	<0.1	0.1	-0.01
Gender: female	44.5	60.0	-0.31	54.1	51.6	0.05
Race						
race = Asian	1.0	1.1	-0.01	1.0	1.0	0.00
race = Black or African American	8.9	10.8	-0.06	9.7	10.0	-0.01
race = Other Race	0.1	0.1	0.01	0.2	0.1	0.03
race = White	34.7	31.6	0.06	32.8	33.2	-0.01
race = Unknown	2.1	2.5	-0.03	2.2	2.3	-0.01
race = Native Hawaiian or Other Pacific Islander	0.0	<0.1		0.0	<0.1	
race = American Indian or Alaska Native	0.1	<0.1	0.03	0.1	<0.1	0.03
race = Asian Indian	0.1	0.2	-0.01	0.1	0.2	-0.01
race = Chinese	<0.1	<0.1	0.00	<0.1	<0.1	-0.01
race = Filipino	<0.1	<0.1	0.00	<0.1	<0.1	-0.01
race = Indonesian	<0.1	0.0		<0.1	0.0	
race = Japanese	<0.1	0.0		<0.1	0.0	
race = Korean	<0.1	<0.1	0.00	<0.1	<0.1	-0.01
race = Laotian	0.7	0.3	0.05	0.5	0.4	0.02
race = Sri Lankan	<0.1	0.0		<0.1	0.0	
race = Thai	0.0	<0.1		0.0	<0.1	
race = Vietnamese	<0.1	0.0		<0.1	0.0	
race = Polynesian	0.0	<0.1		0.0	<0.1	
race = Micronesian	<0.1	<0.1	0.00	<0.1	<0.1	0.00

(Continued)	Before stratification			After stratification		
Characteristic	T (%)	C (%)	StdDiff	T (%)	C (%)	StdDiff
race = Other Pacific Islander	0.8	0.6	0.01	0.8	0.6	0.02
Ethnicity						
ethnicity = Hispanic or Latino	15.5	13.2	0.07	14.5	14.1	0.01
ethnicity = Not Hispanic or Latino	33.5	34.9	-0.03	33.7	34.7	-0.02
Medical history: General						
Acute respiratory disease	4.9	4.9	0.00	4.8	4.8	0.00
Attention deficit hyperactivity disorder	0.2	0.1	0.01	0.2	0.1	0.01
Chronic liver disease	1.7	1.2	0.04	1.4	1.4	-0.01
Chronic obstructive lung disease	4.3	3.5	0.04	3.9	3.6	0.01
Crohn’s disease	0.2	0.2	0.00	0.2	0.2	0.00
Dementia	3.0	1.8	0.08	2.5	2.1	0.03
Depressive disorder	6.2	5.3	0.04	5.8	5.6	0.01
Diabetes mellitus	27.6	19.1	0.20	22.7	23.1	-0.01
Gastroesophageal reflux disease	8.2	8.1	0.00	8.1	8.1	0.00
Gastrointestinal hemorrhage	1.5	1.2	0.03	1.3	1.1	0.01
Human immunodeficiency virus infection	2.4	1.1	0.10	1.8	1.4	0.04
Hyperlipidemia	44.4	41.7	0.05	42.2	43.0	-0.02
Hypertensive disorder	68.5	70.9	-0.05	70.0	70.0	0.00
Lesion of liver	1.8	1.2	0.04	1.5	1.4	0.01
Obesity	10.5	13.2	-0.08	11.9	12.1	-0.01
Osteoarthritis	13.0	15.0	-0.06	14.0	14.1	0.00
Pneumonia	2.4	2.2	0.01	2.1	2.3	-0.01
Psoriasis	0.7	0.7	0.00	0.7	0.7	0.00
Renal impairment	12.6	11.4	0.04	11.9	12.1	-0.01
Rheumatoid arthritis	0.8	0.9	0.00	0.9	0.8	0.01
Schizophrenia	0.4	0.1	0.06	0.4	0.2	0.03
Ulcerative colitis	0.2	0.3	-0.02	0.1	0.3	-0.03
Urinary tract infectious disease	3.5	2.9	0.04	3.5	3.0	0.03
Viral hepatitis C	1.1	0.5	0.06	0.8	0.7	0.02
Visual system disorder	12.0	11.2	0.03	11.8	11.3	0.02
Medical history: Cardiovascular disease						
Atrial fibrillation	11.8	9.9	0.06	10.3	10.8	-0.02
Cerebrovascular disease	7.6	6.6	0.04	6.9	7.1	-0.01
Coronary arteriosclerosis	24.3	15.5	0.22	19.0	19.3	-0.01
Heart disease	44.8	35.1	0.20	38.8	39.3	-0.01
Heart failure	12.9	7.5	0.18	9.9	9.1	0.03
Ischemic heart disease	9.8	6.4	0.12	7.7	7.9	-0.01
Peripheral vascular disease	6.5	5.0	0.06	5.3	6.0	-0.03
Pulmonary embolism	0.7	0.9	-0.02	0.7	0.9	-0.01
Venous thrombosis	1.6	1.5	0.01	1.5	1.5	0.00
Medical history: Neoplasms						
Hematologic neoplasm	1.9	1.6	0.02	1.6	1.5	0.01
Malignant lymphoma	1.2	1.1	0.00	1.1	1.1	0.00
Malignant neoplasm of anorectum	0.2	0.3	-0.02	0.1	0.3	-0.03
Malignant neoplastic disease	10.4	9.9	0.02	10.0	9.9	0.00

(Continued)	Before stratification			After stratification		
Characteristic	T (%)	C (%)	StdDiff	T (%)	C (%)	StdDiff
Malignant tumor of breast	1.3	1.6	-0.03	1.7	1.4	0.02
Malignant tumor of colon	0.4	0.5	-0.01	0.4	0.6	-0.02
Malignant tumor of lung	0.9	0.5	0.05	0.8	0.5	0.03
Malignant tumor of urinary bladder	0.6	0.6	0.00	0.5	0.7	-0.03
Primary malignant neoplasm of prostate	1.9	1.5	0.03	1.5	1.7	-0.01
Medication use						
Antibacterials for systemic use	29.6	30.6	-0.02	29.8	29.8	0.00
Antidepressants	17.0	16.6	0.01	16.8	16.4	0.01
Antiepileptics	17.0	16.0	0.03	16.8	16.2	0.02
Antiinflammatory and antirheumatic products	19.7	22.5	-0.07	21.3	21.2	0.00
Antineoplastic agents	3.7	3.7	0.00	3.7	3.6	0.00
Antipsoriatics	1.1	1.3	-0.02	1.1	1.2	-0.01
Antithrombotic agents	40.8	29.6	0.24	34.1	35.0	-0.02
Diuretics	0.1	0.3	-0.04	0.1	0.3	-0.03
Drugs for acid related disorders	31.0	30.1	0.02	30.2	30.5	-0.01
Drugs for obstructive airway diseases	17.1	18.6	-0.04	17.7	18.0	-0.01
Drugs used in diabetes	28.9	21.6	0.17	24.6	25.1	-0.01
Immunosuppressants	5.7	5.8	0.00	5.7	5.9	-0.01
Lipid modifying agents	56.1	46.3	0.20	49.7	51.2	-0.03
Opioids	15.9	15.4	0.01	15.3	15.5	0.00
Psycholeptics	19.2	18.1	0.03	18.6	18.2	0.01
Psychostimulants, agents used for adhd and nootropics	1.4	1.4	0.00	1.6	1.3	0.03

Supplementary Table 59: Baseline patient characteristcs for ARB mono (T) and CCB/THZ mono (C) prevalent-users in the CUIMC data source. We report proportion of initiators satisfying selected base-line characteristics and the standardized difference of population proportions (StdDiff) before and after stratification. Less extreme StdDiffs through stratification suggest improved balance between patient cohorts through propensity score adjustment.

	Before stratification			After stratification		
Characteristic	T (%)	C (%)	StdDiff	T (%)	C (%)	StdDiff
Age group						
15-19	0.0	0.2		0.0	0.2	
20-24	0.3	0.5	-0.02	0.4	0.4	0.00
25-29	0.4	1.1	-0.07	0.6	0.8	-0.03
30-34	0.7	1.5	-0.07	1.0	1.3	-0.03
35-39	1.3	2.3	-0.07	1.5	2.0	-0.04
40-44	2.5	3.5	-0.06	2.6	3.2	-0.04
45-49	4.3	5.3	-0.04	5.2	5.0	0.01
50-54	6.1	7.0	-0.04	6.9	6.5	0.02
55-59	9.7	9.0	0.02	9.7	9.1	0.02
60-64	13.0	11.4	0.05	12.7	11.8	0.03
65-69	14.9	13.4	0.04	13.5	13.6	0.00
70-74	16.3	15.0	0.04	15.4	15.6	0.00
75-79	12.4	11.9	0.02	12.4	12.5	0.00
80-84	9.4	8.4	0.03	9.1	8.5	0.02
85-89	5.0	5.5	-0.02	5.1	5.6	-0.02
90-94	2.4	3.0	-0.04	2.5	2.8	-0.02
95-99	1.0	0.8	0.02	1.3	0.8	0.05
100-104	<0.2	0.3	-0.04	<0.2	0.2	-0.04
Gender: female	52.5	58.7	-0.12	57.3	56.4	0.02
Race						
race = Asian	2.1	1.4	0.05	1.9	1.5	0.03
race = Black or African American	5.4	10.8	-0.20	9.4	8.9	0.02
race = Other Race	0.2	<0.1	0.04	<0.2	<0.1	0.03
race = White	37.6	31.0	0.14	32.5	33.4	-0.02
race = Unknown	2.1	2.3	-0.01	1.9	2.3	-0.02
race = Native Hawaiian or Other Pacific Islander	<0.2	0.0		<0.2	0.0	
race = American Indian or Alaska Native	0.2	<0.1	0.04	<0.2	<0.1	0.04
race = Asian Indian	0.2	0.2	0.00	0.2	0.2	-0.01
race = Bangladeshi	0.0	<0.1		0.0	<0.1	
race = Chinese	<0.2	<0.1	0.03	<0.2	<0.1	0.03
race = Filipino	<0.2	<0.1	0.00	<0.2	<0.1	0.00
race = Korean	<0.2	<0.1	0.02	<0.2	<0.1	0.02
race = Laotian	0.4	0.5	-0.02	0.6	0.5	0.02
race = Pakistani	0.0	<0.1		0.0	<0.1	
race = Thai	<0.2	<0.1	0.01	<0.2	<0.1	0.00
race = Vietnamese	0.0	<0.1		0.0	<0.1	
race = Other Pacific Islander	0.5	0.6	-0.02	0.5	0.6	-0.01
Ethnicity						
ethnicity = Hispanic or Latino	8.5	13.9	-0.17	12.2	12.0	0.01

(Continued)	Before stratification			After stratification		
Characteristic	T (%)	C (%)	StdDiff	T (%)	C (%)	StdDiff
ethnicity = Not Hispanic or Latino	37.8	34.4	0.07	34.8	35.8	-0.02
Medical history: General						
Acute respiratory disease	3.4	4.0	-0.03	3.5	3.8	-0.02
Attention deficit hyperactivity disorder	<0.2	0.2	-0.04	<0.2	0.2	-0.05
Chronic liver disease	0.5	1.2	-0.08	0.6	1.1	-0.05
Chronic obstructive lung disease	3.0	3.1	-0.01	3.1	2.9	0.02
Crohn's disease	0.4	0.2	0.03	0.3	0.2	0.03
Dementia	1.4	2.3	-0.07	1.6	2.1	-0.04
Depressive disorder	4.3	6.0	-0.08	5.4	5.3	0.00
Diabetes mellitus	18.5	13.2	0.15	14.5	14.4	0.00
Gastroesophageal reflux disease	6.8	7.1	-0.01	7.1	6.7	0.02
Gastrointestinal hemorrhage	0.7	1.1	-0.04	1.1	0.9	0.01
Human immunodeficiency virus infection	0.9	1.7	-0.07	1.4	1.4	0.00
Hyperlipidemia	38.3	33.1	0.11	34.5	34.7	0.00
Hypertensive disorder	60.0	69.0	-0.19	67.2	65.2	0.04
Lesion of liver	0.7	1.5	-0.08	0.9	1.4	-0.05
Obesity	9.4	9.8	-0.01	9.3	9.5	-0.01
Osteoarthritis	10.7	12.2	-0.05	12.3	11.8	0.02
Pneumonia	1.1	1.9	-0.07	1.5	1.6	-0.01
Psoriasis	0.7	0.5	0.02	0.7	0.5	0.02
Renal impairment	6.8	9.7	-0.10	8.4	8.7	-0.01
Rheumatoid arthritis	0.8	0.8	0.00	1.0	0.8	0.03
Schizophrenia	<0.2	0.3	-0.04	0.3	0.2	0.01
Ulcerative colitis	0.2	0.1	0.01	0.2	0.1	0.03
Urinary tract infectious disease	2.0	2.8	-0.06	2.7	2.4	0.02
Viral hepatitis C	0.2	0.8	-0.08	0.4	0.7	-0.05
Visual system disorder	10.9	10.2	0.02	10.2	10.2	0.00
Medical history: Cardiovascular disease						
Atrial fibrillation	5.2	4.6	0.03	4.7	4.7	0.00
Cerebrovascular disease	5.3	5.0	0.01	4.8	5.1	-0.01
Coronary arteriosclerosis	13.3	10.7	0.08	12.1	11.5	0.02
Heart disease	29.0	25.0	0.09	27.1	26.0	0.03
Heart failure	5.1	2.4	0.14	3.7	2.7	0.06
Ischemic heart disease	3.5	3.3	0.01	3.5	3.5	0.00
Peripheral vascular disease	3.8	3.2	0.04	3.1	3.3	-0.01
Pulmonary embolism	0.4	0.5	-0.01	0.5	0.4	0.01
Venous thrombosis	0.8	1.2	-0.04	0.9	1.1	-0.02
Medical history: Neoplasms						
Hematologic neoplasm	2.0	1.8	0.01	2.1	1.6	0.04
Malignant lymphoma	1.2	1.3	-0.01	1.1	1.2	-0.01
Malignant neoplasm of anorectum	0.3	0.2	0.04	0.4	0.2	0.04
Malignant neoplastic disease	10.4	10.8	-0.01	10.7	10.5	0.01
Malignant tumor of breast	2.0	1.7	0.03	1.9	1.6	0.02
Malignant tumor of colon	0.3	0.5	-0.04	0.4	0.5	-0.02
Malignant tumor of lung	0.5	0.6	-0.01	0.5	0.6	-0.01

(Continued)	Before stratification			After stratification		
Characteristic	T (%)	C (%)	StdDiff	T (%)	C (%)	StdDiff
Malignant tumor of urinary bladder	0.6	0.5	0.02	0.6	0.5	0.02
Primary malignant neoplasm of prostate	1.5	1.8	-0.02	1.6	1.8	-0.02
Medication use						
Antibacterials for systemic use	27.4	27.1	0.01	26.6	26.9	-0.01
Antidepressants	15.4	15.5	0.00	15.4	15.3	0.00
Antiepileptics	13.0	13.0	0.00	13.4	12.6	0.02
Antiinflammatory and antirheumatic products	16.8	20.5	-0.10	18.5	19.3	-0.02
Antineoplastic agents	3.4	4.1	-0.04	4.1	3.8	0.01
Antipsoriatics	0.6	1.3	-0.07	0.8	1.2	-0.03
Antithrombotic agents	20.3	22.5	-0.06	21.7	21.3	0.01
Diuretics	<0.2	<0.1	0.00	<0.2	<0.1	0.01
Drugs for acid related disorders	22.8	26.9	-0.09	25.4	25.7	-0.01
Drugs for obstructive airway diseases	15.5	15.1	0.01	15.2	15.1	0.00
Drugs used in diabetes	19.6	12.8	0.19	14.5	14.9	-0.01
Immunosuppressants	5.3	7.7	-0.10	7.1	6.8	0.01
Lipid modifying agents	40.8	35.0	0.12	36.5	37.0	-0.01
Opioids	9.8	14.4	-0.14	11.7	12.9	-0.04
Psycholeptics	13.9	15.9	-0.06	14.1	15.4	-0.04
Psychostimulants, agents used for adhd and nootropics	1.4	1.5	-0.01	1.4	1.5	0.00

Supplementary Table 60: Baseline patient characteristcs for ARB +combo (T) and CCB/THZ +combo (C) prevalent-users in the CUIMC data source. We report proportion of initiators satisfying selected base-line characteristics and the standardized difference of population proportions (StdDiff) before and after stratification. Less extreme StdDiffs through stratification suggest improved balance between patient cohorts through propensity score adjustment.

	Before stratification			After stratification		
Characteristic	T (%)	C (%)	StdDiff	T (%)	C (%)	StdDiff
Age group						
15-19	<0.1	0.2	-0.04	<0.1	0.1	-0.04
20-24	0.3	0.4	-0.02	0.4	0.4	0.01
25-29	0.3	0.7	-0.06	0.5	0.6	-0.01
30-34	0.7	1.4	-0.07	0.9	1.2	-0.03
35-39	1.4	2.2	-0.06	1.5	1.9	-0.03
40-44	2.0	3.0	-0.06	2.3	2.8	-0.03
45-49	3.4	4.4	-0.05	4.0	4.2	-0.01
50-54	5.6	6.3	-0.03	6.1	5.9	0.01
55-59	8.9	9.0	-0.01	9.5	8.8	0.02
60-64	12.7	11.3	0.04	11.6	11.6	0.00
65-69	14.1	13.6	0.01	13.3	13.5	-0.01
70-74	16.1	15.1	0.03	15.7	15.5	0.01
75-79	13.3	12.6	0.02	13.0	13.2	0.00
80-84	10.3	9.6	0.02	10.0	9.9	0.00
85-89	6.5	5.9	0.02	6.4	6.2	0.01
90-94	3.5	3.2	0.02	3.6	3.2	0.02
95-99	0.9	0.9	0.00	1.1	0.9	0.02
100-104	0.1	0.2	-0.02	<0.1	0.2	-0.03
Gender: female	49.3	55.1	-0.12	53.4	53.8	-0.01
Race						
race = Asian	1.9	1.3	0.05	1.7	1.4	0.02
race = Black or African American	7.1	11.6	-0.16	10.4	10.0	0.01
race = Other Race	0.1	0.1	0.00	0.1	0.1	0.00
race = White	37.4	31.1	0.13	32.8	33.4	-0.01
race = Unknown	2.1	2.2	-0.01	2.0	2.3	-0.02
race = Native Hawaiian or Other Pacific Islander	<0.1	0.0	0.01	<0.1	0.0	0.00
race = American Indian or Alaska Native	0.2	0.1	0.03	0.1	0.1	0.02
race = Asian Indian	0.2	0.2	0.00	0.2	0.2	-0.02
race = Bangladeshi	<0.1	0.0	0.00	<0.1	0.0	0.00
race = Chinese	<0.1	0.0	0.02	0.1	0.0	0.04
race = Filipino	<0.1	0.0	0.00	<0.1	0.0	0.00
race = Indonesian	<0.1	0.0		<0.1	0.0	
race = Japanese	0.0	0.0		0.0	0.0	
race = Korean	<0.1	0.0	0.02	<0.1	0.0	0.01
race = Laotian	0.5	0.7	-0.03	0.6	0.6	0.00
race = Pakistani	<0.1	0.0	0.00	<0.1	0.0	0.00
race = Sri Lankan	0.0	0.0		0.0	0.0	
race = Thai	<0.1	0.0	0.01	<0.1	0.0	0.00
race = Vietnamese	0.0	0.0		0.0	0.0	

(Continued)	Before stratification			After stratification		
Characteristic	T (%)	C (%)	StdDiff	T (%)	C (%)	StdDiff
race = Micronesian	<0.1	0.0		<0.1	0.0	
race = Other Pacific Islander	0.4	0.6	-0.03	0.5	0.6	-0.02
Ethnicity						
ethnicity = Hispanic or Latino	10.4	15.0	-0.14	14.3	13.3	0.03
ethnicity = Not Hispanic or Latino	37.8	33.9	0.08	34.4	35.3	-0.02
Medical history: General						
Acute respiratory disease	4.8	5.8	-0.04	5.5	5.2	0.01
Attention deficit hyperactivity disorder	<0.1	0.2	-0.03	<0.1	0.2	-0.03
Chronic liver disease	1.0	1.7	-0.07	1.2	1.5	-0.02
Chronic obstructive lung disease	4.8	4.5	0.02	5.0	4.3	0.03
Crohn’s disease	0.3	0.3	0.01	0.3	0.2	0.02
Dementia	2.1	2.8	-0.05	2.8	2.5	0.02
Depressive disorder	4.8	7.1	-0.09	6.5	6.3	0.01
Diabetes mellitus	23.3	21.5	0.04	21.0	21.8	-0.02
Gastroesophageal reflux disease	8.3	9.0	-0.03	8.8	8.5	0.01
Gastrointestinal hemorrhage	1.1	1.7	-0.04	1.4	1.4	0.00
Human immunodeficiency virus infection	0.8	1.9	-0.09	1.4	1.6	-0.01
Hyperlipidemia	44.9	39.8	0.10	40.9	41.5	-0.01
Hypertensive disorder	66.7	72.6	-0.13	71.2	70.9	0.01
Lesion of liver	1.2	2.1	-0.07	1.6	1.8	-0.01
Obesity	11.2	11.5	-0.01	11.1	11.4	-0.01
Osteoarthritis	11.7	14.4	-0.08	14.5	13.6	0.03
Pneumonia	1.9	3.4	-0.09	2.2	2.9	-0.05
Psoriasis	0.7	0.6	0.00	0.7	0.6	0.01
Renal impairment	12.0	17.9	-0.17	15.7	15.7	0.00
Rheumatoid arthritis	1.0	0.9	0.01	1.3	0.9	0.04
Schizophrenia	0.1	0.4	-0.05	0.2	0.3	-0.02
Ulcerative colitis	0.1	0.2	-0.01	0.2	0.2	0.00
Urinary tract infectious disease	2.8	4.1	-0.07	3.7	3.6	0.01
Viral hepatitis C	0.6	1.1	-0.06	0.8	0.9	-0.02
Visual system disorder	10.5	12.1	-0.05	11.3	11.7	-0.01
Medical history: Cardiovascular disease						
Atrial fibrillation	14.1	9.5	0.14	11.3	10.4	0.03
Cerebrovascular disease	7.6	7.2	0.02	6.6	7.4	-0.03
Coronary arteriosclerosis	23.9	17.6	0.16	19.6	19.1	0.01
Heart disease	48.2	36.9	0.23	40.3	39.5	0.02
Heart failure	17.2	8.5	0.26	12.4	9.0	0.11
Ischemic heart disease	9.3	7.3	0.07	7.6	7.7	0.00
Peripheral vascular disease	5.8	5.7	0.01	5.3	5.8	-0.03
Pulmonary embolism	0.7	0.9	-0.02	0.8	0.8	0.00
Venous thrombosis	1.2	2.1	-0.07	1.3	1.8	-0.04
Medical history: Neoplasms						
Hematologic neoplasm	1.8	2.1	-0.03	2.2	1.9	0.02
Malignant lymphoma	1.1	1.3	-0.02	1.1	1.3	-0.02
Malignant neoplasm of anorectum	0.3	0.3	0.00	0.3	0.3	0.01

(Continued)	Before stratification			After stratification		
Characteristic	T (%)	C (%)	StdDiff	T (%)	C (%)	StdDiff
Malignant neoplastic disease	10.2	11.6	-0.05	10.9	11.2	-0.01
Malignant tumor of breast	1.7	1.5	0.01	1.8	1.5	0.03
Malignant tumor of colon	0.4	0.6	-0.02	0.5	0.5	0.00
Malignant tumor of lung	0.6	0.9	-0.03	0.7	0.8	-0.02
Malignant tumor of urinary bladder	0.6	0.6	0.00	0.7	0.6	0.01
Primary malignant neoplasm of prostate	1.5	1.8	-0.03	1.6	1.8	-0.01
Medication use						
Antibacterials for systemic use	31.9	33.2	-0.03	32.3	32.6	-0.01
Antidepressants	16.9	17.9	-0.03	17.9	17.7	0.01
Antiepileptics	15.5	17.5	-0.05	16.8	16.7	0.00
Antiinflammatory and antirheumatic products	17.5	21.2	-0.10	20.3	20.3	0.00
Antineoplastic agents	3.7	4.8	-0.05	4.5	4.4	0.00
Antipsoriatics	1.0	2.1	-0.09	1.4	1.8	-0.03
Antithrombotic agents	35.7	36.2	-0.01	36.1	35.2	0.02
Diuretics	<0.1	0.3	-0.04	<0.1	0.2	-0.05
Drugs for acid related disorders	29.9	33.9	-0.09	32.7	32.4	0.01
Drugs for obstructive airway diseases	19.9	19.4	0.01	19.2	19.3	0.00
Drugs used in diabetes	23.6	21.7	0.05	21.0	22.3	-0.03
Immunosuppressants	6.2	9.4	-0.12	8.1	8.4	-0.01
Lipid modifying agents	50.6	46.6	0.08	47.8	47.7	0.00
Opioids	13.8	19.7	-0.16	17.0	17.8	-0.02
Psycholeptics	16.9	20.7	-0.10	17.9	19.4	-0.04
Psychostimulants, agents used for adhd and nootropics	1.3	1.6	-0.02	1.2	1.5	-0.02

Supplementary Table 61: Baseline patient characteristcs for ARB mono (T) and CCB mono (C) prevalent-users in the CUIMC data source. We report proportion of initiators satisfying selected base-line characteristics and the standardized difference of population proportions (StdDiff) before and after stratification. Less extreme StdDiffs through stratification suggest improved balance between patient cohorts through propensity score adjustment.

	Before stratification			After stratification		
Characteristic	T (%)	C (%)	StdDiff	T (%)	C (%)	StdDiff
Age group						
15-19	0.0	0.3		0.0	0.3	
20-24	0.3	0.7	-0.05	0.3	0.5	-0.03
25-29	0.4	1.4	-0.11	0.7	1.0	-0.04
30-34	0.7	1.9	-0.10	0.9	1.4	-0.04
35-39	1.3	2.8	-0.10	1.7	2.2	-0.04
40-44	2.5	3.4	-0.05	2.6	3.1	-0.03
45-49	4.3	5.0	-0.03	5.0	4.6	0.02
50-54	6.1	6.5	-0.01	6.9	5.9	0.04
55-59	9.7	8.3	0.05	9.3	8.5	0.03
60-64	13.0	10.8	0.07	12.2	11.6	0.02
65-69	14.9	12.8	0.06	13.9	13.4	0.01
70-74	16.3	15.3	0.03	15.6	15.9	-0.01
75-79	12.4	11.7	0.02	12.4	12.4	0.00
80-84	9.4	8.9	0.02	9.4	9.1	0.01
85-89	5.0	5.9	-0.04	5.1	5.8	-0.03
90-94	2.4	3.1	-0.04	2.7	3.0	-0.02
95-99	1.0	0.9	0.00	1.2	0.9	0.03
100-104	<0.2	0.4	-0.06	<0.2	0.4	-0.07
Gender: female	52.5	56.7	-0.08	55.0	54.9	0.00
Race						
race = Asian	2.1	1.8	0.02	2.0	1.9	0.01
race = Black or African American	5.4	11.0	-0.20	9.1	8.4	0.02
race = Other Race	0.2	<0.1	0.04	<0.2	<0.1	0.04
race = White	37.6	31.2	0.14	33.4	34.3	-0.02
race = Unknown	2.1	1.9	0.01	2.1	2.0	0.00
race = Native Hawaiian or Other Pacific Islander	<0.2	0.0		<0.2	0.0	
race = American Indian or Alaska Native	0.2	<0.1	0.03	<0.2	<0.1	0.02
race = Asian Indian	0.2	0.3	-0.02	0.3	0.3	-0.01
race = Bangladeshi	0.0	<0.1		0.0	<0.1	
race = Chinese	<0.2	<0.1	0.02	<0.2	<0.1	0.02
race = Filipino	<0.2	<0.1	-0.01	<0.2	<0.1	-0.01
race = Korean	<0.2	<0.1	0.01	<0.2	<0.1	0.02
race = Laotian	0.4	0.5	-0.02	0.7	0.4	0.03
race = Pakistani	0.0	<0.1		0.0	<0.1	
race = Thai	<0.2	0.0		<0.2	0.0	
race = Vietnamese	0.0	<0.1		0.0	<0.1	
race = Other Pacific Islander	0.5	0.7	-0.03	0.5	0.6	-0.01
Ethnicity						
ethnicity = Hispanic or Latino	8.5	14.0	-0.17	11.6	11.6	0.00

(Continued)	Before stratification			After stratification		
Characteristic	T (%)	C (%)	StdDiff	T (%)	C (%)	StdDiff
ethnicity = Not Hispanic or Latino	37.8	34.6	0.07	35.7	36.2	-0.01
Medical history: General						
Acute respiratory disease	3.4	4.2	-0.04	3.7	3.8	-0.01
Attention deficit hyperactivity disorder	<0.2	0.2	-0.04	<0.2	0.3	-0.06
Chronic liver disease	0.5	1.3	-0.08	0.6	1.1	-0.04
Chronic obstructive lung disease	3.0	3.6	-0.03	3.4	3.2	0.01
Crohn's disease	0.4	0.2	0.03	0.3	0.2	0.03
Dementia	1.4	2.7	-0.09	1.9	2.3	-0.02
Depressive disorder	4.3	6.0	-0.08	5.4	5.2	0.01
Diabetes mellitus	18.5	14.3	0.12	15.8	15.5	0.01
Gastroesophageal reflux disease	6.8	7.1	-0.01	7.5	6.5	0.04
Gastrointestinal hemorrhage	0.7	1.2	-0.05	1.1	1.0	0.00
Human immunodeficiency virus infection	0.9	1.8	-0.08	1.4	1.4	0.00
Hyperlipidemia	38.3	31.9	0.13	34.6	35.0	-0.01
Hypertensive disorder	60.0	68.7	-0.18	65.5	64.3	0.03
Lesion of liver	0.7	1.7	-0.10	0.9	1.5	-0.05
Obesity	9.4	9.0	0.01	9.0	8.9	0.00
Osteoarthritis	10.7	11.9	-0.04	12.3	11.4	0.03
Pneumonia	1.1	2.5	-0.10	1.7	1.9	-0.02
Psoriasis	0.7	0.6	0.01	0.6	0.6	0.01
Renal impairment	6.8	11.5	-0.16	9.0	9.3	-0.01
Rheumatoid arthritis	0.8	0.8	0.00	1.2	0.8	0.04
Schizophrenia	<0.2	0.3	-0.04	0.3	0.2	0.02
Ulcerative colitis	0.2	<0.1	0.03	0.3	<0.1	0.05
Urinary tract infectious disease	2.0	3.2	-0.08	2.8	2.6	0.01
Viral hepatitis C	0.2	0.8	-0.08	0.4	0.7	-0.03
Visual system disorder	10.9	10.1	0.02	10.6	10.1	0.02
Medical history: Cardiovascular disease						
Atrial fibrillation	5.2	4.4	0.04	4.9	4.5	0.02
Cerebrovascular disease	5.3	5.4	0.00	5.2	5.3	0.00
Coronary arteriosclerosis	13.3	11.8	0.05	12.6	12.6	0.00
Heart disease	29.0	26.3	0.06	28.1	27.1	0.02
Heart failure	5.1	2.5	0.13	4.1	2.9	0.07
Ischemic heart disease	3.5	3.8	-0.01	3.6	3.9	-0.01
Peripheral vascular disease	3.8	3.6	0.01	3.2	3.7	-0.03
Pulmonary embolism	0.4	0.5	-0.02	0.5	0.4	0.00
Venous thrombosis	0.8	1.3	-0.05	1.0	1.1	-0.01
Medical history: Neoplasms						
Hematologic neoplasm	2.0	1.8	0.01	2.4	1.6	0.06
Malignant lymphoma	1.2	1.4	-0.02	1.2	1.2	0.00
Malignant neoplasm of anorectum	0.3	0.2	0.04	0.4	0.1	0.04
Malignant neoplastic disease	10.4	11.1	-0.03	11.1	10.8	0.01
Malignant tumor of breast	2.0	1.4	0.04	2.1	1.5	0.04
Malignant tumor of colon	0.3	0.5	-0.03	0.3	0.4	-0.02
Malignant tumor of lung	0.5	0.8	-0.03	0.5	0.7	-0.03

(Continued)	Before stratification			After stratification		
Characteristic	T (%)	C (%)	StdDiff	T (%)	C (%)	StdDiff
Malignant tumor of urinary bladder	0.6	0.4	0.03	0.6	0.4	0.03
Primary malignant neoplasm of prostate	1.5	1.8	-0.02	1.7	1.8	-0.01
Medication use						
Antibacterials for systemic use	27.4	29.1	-0.04	27.9	28.0	0.00
Antidepressants	15.4	15.8	-0.01	15.7	15.2	0.01
Antiepileptics	13.0	13.3	-0.01	13.4	12.5	0.03
Antiinflammatory and antirheumatic products	16.8	20.2	-0.09	18.7	18.8	0.00
Antineoplastic agents	3.4	4.5	-0.06	4.1	4.0	0.00
Antipsoriatics	0.6	1.5	-0.09	0.8	1.1	-0.04
Antithrombotic agents	20.3	25.7	-0.13	23.2	22.6	0.01
Diuretics	<0.2	<0.1	0.00	<0.2	<0.1	0.02
Drugs for acid related disorders	22.8	28.8	-0.14	26.2	26.4	0.00
Drugs for obstructive airway diseases	15.5	15.5	0.00	15.8	15.5	0.01
Drugs used in diabetes	19.6	13.0	0.18	15.5	15.8	-0.01
Immunosuppressants	5.3	9.5	-0.16	7.7	7.7	0.00
Lipid modifying agents	40.8	35.1	0.12	37.7	37.9	0.00
Opioids	9.8	15.9	-0.18	12.3	13.4	-0.03
Psycholeptics	13.9	16.9	-0.08	14.9	15.8	-0.02
Psychostimulants, agents used for adhd and nootropics	1.4	1.6	-0.02	1.4	1.6	-0.02

Supplementary Table 62: Baseline patient characteristcs for ARB +combo (T) and CCB +combo (C) prevalent-users in the CUIMC data source. We report proportion of initiators satisfying selected base-line characteristics and the standardized difference of population proportions (StdDiff) before and after stratification. Less extreme StdDiffs through stratification suggest improved balance between patient cohorts through propensity score adjustment.

	Before stratification			After stratification		
Characteristic	T (%)	C (%)	StdDiff	T (%)	C (%)	StdDiff
Age group						
15-19	<0.1	0.2	-0.05	<0.1	0.2	-0.03
20-24	0.2	0.5	-0.05	0.3	0.4	-0.01
25-29	0.3	0.9	-0.08	0.4	0.7	-0.03
30-34	0.6	1.6	-0.09	0.7	1.2	-0.05
35-39	1.2	2.5	-0.10	1.5	2.0	-0.04
40-44	2.0	2.9	-0.06	2.2	2.6	-0.03
45-49	3.4	4.2	-0.04	3.6	3.9	-0.01
50-54	5.8	6.1	-0.01	6.1	5.7	0.02
55-59	9.2	8.7	0.02	9.5	8.9	0.02
60-64	12.7	11.0	0.05	11.4	11.7	-0.01
65-69	14.5	13.4	0.03	13.8	13.8	0.00
70-74	16.2	15.0	0.04	15.8	15.4	0.01
75-79	13.5	12.4	0.03	13.4	13.0	0.01
80-84	10.3	9.7	0.02	10.6	10.0	0.02
85-89	5.9	6.3	-0.01	6.3	6.2	0.00
90-94	3.2	3.3	0.00	3.4	3.2	0.01
95-99	0.7	1.0	-0.03	0.8	0.9	-0.02
100-104	0.1	0.2	-0.04	<0.1	0.2	-0.04
Gender: female	52.7	52.7	0.00	52.6	53.7	-0.02
Race						
race = Asian	1.8	1.5	0.02	1.8	1.5	0.02
race = Black or African American	8.0	12.6	-0.15	10.3	10.1	0.01
race = Other Race	0.1	0.1	-0.01	0.1	0.2	-0.01
race = White	35.6	30.7	0.11	32.9	33.3	-0.01
race = Unknown	2.2	2.1	0.00	2.0	2.3	-0.02
race = Native Hawaiian or Other Pacific Islander	<0.1	<0.1	0.01	<0.1	0.0	0.01
race = American Indian or Alaska Native	0.1	0.1	0.02	0.1	0.1	0.01
race = Asian Indian	0.2	0.3	-0.01	0.2	0.3	-0.02
race = Bangladeshi	<0.1	<0.1	-0.01	<0.1	0.0	-0.01
race = Chinese	0.1	<0.1	0.02	0.1	0.0	0.03
race = Filipino	<0.1	0.1	0.00	<0.1	0.1	-0.01
race = Indonesian	<0.1	0.0		<0.1	0.0	
race = Japanese	0.0	<0.1		0.0	0.0	
race = Korean	0.1	<0.1	0.02	0.1	0.0	0.01
race = Laotian	0.4	0.8	-0.05	0.5	0.6	-0.03
race = Pakistani	<0.1	<0.1	-0.01	<0.1	0.0	-0.01
race = Sri Lankan	0.0	<0.1		0.0	0.0	
race = Thai	<0.1	<0.1	0.00	<0.1	0.0	0.00
race = Vietnamese	0.0	<0.1		0.0	0.0	

(Continued)	Before stratification			After stratification		
Characteristic	T (%)	C (%)	StdDiff	T (%)	C (%)	StdDiff
race = Micronesian	<0.1	0.0		<0.1	0.0	
race = Other Pacific Islander	0.6	0.7	-0.01	0.6	0.6	0.00
Ethnicity						
ethnicity = Hispanic or Latino	11.2	15.6	-0.13	13.8	13.4	0.01
ethnicity = Not Hispanic or Latino	36.8	34.1	0.06	35.2	35.5	-0.01
Medical history: General						
Acute respiratory disease	4.4	6.1	-0.07	5.3	5.1	0.01
Attention deficit hyperactivity disorder	0.1	0.2	-0.03	0.1	0.2	-0.01
Chronic liver disease	1.0	2.0	-0.08	1.5	1.5	0.00
Chronic obstructive lung disease	4.2	4.9	-0.03	4.7	4.2	0.03
Crohn’s disease	0.3	0.3	0.00	0.2	0.3	0.00
Dementia	1.8	3.3	-0.10	2.7	2.6	0.01
Depressive disorder	4.6	7.5	-0.12	6.0	6.0	0.00
Diabetes mellitus	22.1	23.3	-0.03	21.6	22.5	-0.02
Gastroesophageal reflux disease	8.1	9.5	-0.05	8.7	8.6	0.00
Gastrointestinal hemorrhage	1.2	1.9	-0.06	1.5	1.5	0.00
Human immunodeficiency virus infection	0.7	1.9	-0.10	1.3	1.4	-0.01
Hyperlipidemia	44.0	39.9	0.08	41.9	41.9	0.00
Hypertensive disorder	67.6	73.9	-0.14	71.1	70.9	0.00
Lesion of liver	1.2	2.4	-0.09	1.8	1.9	-0.01
Obesity	12.0	10.8	0.04	11.0	11.3	-0.01
Osteoarthritis	12.9	14.4	-0.04	14.3	13.9	0.01
Pneumonia	1.9	4.1	-0.13	2.5	3.0	-0.03
Psoriasis	0.7	0.6	0.01	0.6	0.5	0.01
Renal impairment	10.8	21.3	-0.29	15.4	15.9	-0.01
Rheumatoid arthritis	0.9	0.9	0.00	1.1	0.9	0.02
Schizophrenia	0.1	0.5	-0.07	0.2	0.3	-0.03
Ulcerative colitis	0.2	0.2	-0.01	0.2	0.2	0.00
Urinary tract infectious disease	2.7	4.7	-0.11	3.5	3.7	-0.01
Viral hepatitis C	0.5	1.3	-0.08	0.9	1.0	-0.01
Visual system disorder	10.3	12.3	-0.06	11.3	11.7	-0.01
Medical history: Cardiovascular disease						
Atrial fibrillation	12.5	9.2	0.10	10.9	10.1	0.03
Cerebrovascular disease	7.1	7.6	-0.02	7.1	7.2	0.00
Coronary arteriosclerosis	21.3	19.3	0.05	20.6	19.3	0.03
Heart disease	43.8	38.8	0.10	41.3	39.9	0.03
Heart failure	13.6	9.0	0.14	11.7	8.8	0.10
Ischemic heart disease	8.6	8.3	0.01	8.4	8.0	0.02
Peripheral vascular disease	5.5	6.3	-0.03	5.5	5.9	-0.02
Pulmonary embolism	0.8	0.9	-0.01	0.9	0.8	0.00
Venous thrombosis	1.1	2.3	-0.10	1.3	1.7	-0.04
Medical history: Neoplasms						
Hematologic neoplasm	1.6	2.4	-0.05	2.2	1.9	0.02
Malignant lymphoma	1.1	1.5	-0.04	1.2	1.3	-0.01
Malignant neoplasm of anorectum	0.3	0.3	-0.01	0.3	0.3	0.02

(Continued)	Before stratification			After stratification		
Characteristic	T (%)	C (%)	StdDiff	T (%)	C (%)	StdDiff
Malignant neoplastic disease	9.8	12.5	-0.09	11.2	11.2	0.00
Malignant tumor of breast	1.5	1.3	0.02	1.6	1.4	0.02
Malignant tumor of colon	0.4	0.6	-0.03	0.5	0.6	0.00
Malignant tumor of lung	0.6	1.1	-0.06	0.7	0.8	-0.01
Malignant tumor of urinary bladder	0.6	0.6	0.00	0.7	0.5	0.02
Primary malignant neoplasm of prostate	1.6	2.0	-0.03	1.8	1.9	0.00
Medication use						
Antibacterials for systemic use	31.6	35.2	-0.08	32.6	33.6	-0.02
Antidepressants	16.4	18.2	-0.05	17.2	17.3	0.00
Antiepileptics	15.5	18.5	-0.08	16.9	16.8	0.00
Antiinflammatory and antirheumatic products	19.3	21.1	-0.05	20.2	20.8	-0.01
Antineoplastic agents	3.6	5.2	-0.08	4.4	4.5	0.00
Antipsoriatics	0.9	2.6	-0.13	1.3	1.9	-0.04
Antithrombotic agents	33.5	40.2	-0.14	37.1	35.7	0.03
Diuretics	<0.1	0.2	-0.04	<0.1	0.1	-0.03
Drugs for acid related disorders	29.5	36.6	-0.15	32.6	33.1	-0.01
Drugs for obstructive airway diseases	19.5	20.2	-0.02	19.2	19.8	-0.02
Drugs used in diabetes	23.4	22.7	0.02	21.8	23.4	-0.04
Immunosuppressants	5.8	11.3	-0.20	8.0	8.8	-0.03
Lipid modifying agents	49.5	47.7	0.04	49.0	48.4	0.01
Opioids	13.6	22.1	-0.22	16.7	18.1	-0.04
Psycholeptics	16.9	22.3	-0.14	18.7	19.8	-0.03
Psychostimulants, agents used for adhd and nootropics	1.4	1.7	-0.02	1.4	1.6	-0.01

Supplementary Table 63: Baseline patient characteristcs for ARB mono (T) and THZ mono (C) prevalent-users in the CUIMC data source. We report proportion of initiators satisfying selected base-line characteristics and the standardized difference of population proportions (StdDiff) before and after stratification. Less extreme StdDiffs through stratification suggest improved balance between patient cohorts through propensity score adjustment.

	Before stratification			After stratification		
Characteristic	T (%)	C (%)	StdDiff	T (%)	C (%)	StdDiff
Age group						
15-19	0.0	<0.3		0.0	<0.3	
20-24	0.3	<0.3	0.04	0.3	<0.3	0.06
25-29	0.4	0.3	0.01	0.4	<0.3	0.03
30-34	0.7	0.7	0.00	0.8	0.7	0.02
35-39	1.3	1.1	0.02	1.3	0.9	0.04
40-44	2.5	3.8	-0.07	2.5	3.2	-0.04
45-49	4.3	5.8	-0.07	4.7	5.2	-0.02
50-54	6.1	8.1	-0.08	6.4	6.9	-0.02
55-59	9.7	10.6	-0.03	10.2	10.0	0.01
60-64	13.0	12.5	0.02	13.4	12.4	0.03
65-69	14.9	14.6	0.01	14.4	14.6	-0.01
70-74	16.3	13.9	0.07	15.8	14.4	0.04
75-79	12.4	12.8	-0.01	12.3	14.4	-0.06
80-84	9.4	7.6	0.06	9.0	8.4	0.02
85-89	5.0	4.9	0.00	4.9	5.3	-0.02
90-94	2.4	2.4	0.00	2.3	2.3	0.00
95-99	1.0	0.5	0.05	1.1	0.9	0.02
100-104	<0.2	0.0		<0.2	0.0	
Gender: female	52.5	63.3	-0.22	56.9	54.0	0.06
Race						
race = Asian	2.1	0.9	0.10	2.0	0.7	0.11
race = Black or African American	5.4	9.8	-0.17	6.8	6.7	0.00
race = Other Race	0.2	0.0		<0.2	0.0	
race = White	37.6	31.6	0.13	35.3	36.1	-0.02
race = Unknown	2.1	3.0	-0.06	1.9	2.9	-0.06
race = Native Hawaiian or Other Pacific Islander	<0.2	0.0		<0.2	0.0	
race = American Indian or Alaska Native	0.2	<0.3	0.04	0.2	<0.3	0.05
race = Asian Indian	0.2	<0.3	0.04	0.2	<0.3	0.04
race = Chinese	<0.2	0.0		<0.2	0.0	
race = Filipino	<0.2	0.0		<0.2	0.0	
race = Korean	<0.2	0.0		<0.2	0.0	
race = Laotian	0.4	0.6	-0.03	0.4	0.4	0.00
race = Thai	<0.2	0.0		<0.2	0.0	
race = Other Pacific Islander	0.5	0.3	0.02	0.5	0.3	0.03
Ethnicity						
ethnicity = Hispanic or Latino	8.5	14.2	-0.18	10.5	10.3	0.00
ethnicity = Not Hispanic or Latino	37.8	33.9	0.08	36.2	37.2	-0.02
Medical history: General						
Acute respiratory disease	3.4	3.7	-0.01	3.4	3.5	0.00

(Continued)	Before stratification			After stratification		
Characteristic	T (%)	C (%)	StdDiff	T (%)	C (%)	StdDiff
Attention deficit hyperactivity disorder	<0.2	<0.3	-0.02	<0.2	<0.3	-0.01
Chronic liver disease	0.5	1.0	-0.06	0.4	1.2	-0.09
Chronic obstructive lung disease	3.0	2.0	0.06	2.7	2.1	0.04
Crohn’s disease	0.4	<0.3	0.04	0.4	<0.3	0.06
Dementia	1.4	1.3	0.01	1.3	1.0	0.02
Depressive disorder	4.3	5.7	-0.07	4.5	4.9	-0.02
Diabetes mellitus	18.5	10.7	0.22	15.8	15.9	0.00
Gastroesophageal reflux disease	6.8	6.3	0.02	6.8	5.8	0.04
Gastrointestinal hemorrhage	0.7	0.5	0.02	0.8	0.5	0.04
Human immunodeficiency virus infection	0.9	1.4	-0.05	1.0	1.0	0.00
Hyperlipidemia	38.3	33.7	0.10	36.3	37.5	-0.03
Hypertensive disorder	60.0	65.7	-0.12	62.0	61.8	0.00
Lesion of liver	0.7	1.2	-0.05	0.6	1.4	-0.08
Obesity	9.4	11.7	-0.08	9.9	10.5	-0.02
Osteoarthritis	10.7	12.7	-0.06	11.6	11.9	-0.01
Pneumonia	1.1	0.8	0.03	0.9	0.9	0.00
Psoriasis	0.7	0.5	0.03	0.7	0.4	0.03
Renal impairment	6.8	5.6	0.05	6.3	7.5	-0.05
Rheumatoid arthritis	0.8	0.6	0.03	0.9	0.6	0.04
Schizophrenia	<0.2	<0.3	0.01	<0.2	<0.3	0.02
Ulcerative colitis	0.2	0.0		0.2	0.0	
Urinary tract infectious disease	2.0	1.8	0.01	2.1	1.4	0.05
Viral hepatitis C	0.2	0.5	-0.05	0.2	0.7	-0.08
Visual system disorder	10.9	10.5	0.01	10.7	10.2	0.01
Medical history: Cardiovascular disease						
Atrial fibrillation	5.2	5.3	0.00	5.0	6.2	-0.05
Cerebrovascular disease	5.3	3.9	0.07	4.7	4.6	0.00
Coronary arteriosclerosis	13.3	8.8	0.14	11.9	12.6	-0.02
Heart disease	29.0	21.9	0.16	26.8	27.5	-0.01
Heart failure	5.1	2.1	0.16	4.2	3.3	0.04
Ischemic heart disease	3.5	2.4	0.07	3.2	4.1	-0.05
Peripheral vascular disease	3.8	2.6	0.07	3.3	3.8	-0.03
Pulmonary embolism	0.4	0.5	-0.02	0.3	0.5	-0.03
Venous thrombosis	0.8	0.5	0.04	0.7	0.4	0.04
Medical history: Neoplasms						
Hematologic neoplasm	2.0	1.3	0.05	1.9	1.3	0.05
Malignant lymphoma	1.2	1.1	0.01	1.1	0.9	0.02
Malignant neoplasm of anorectum	0.3	<0.3	0.04	0.3	<0.3	0.05
Malignant neoplastic disease	10.4	9.5	0.03	9.9	9.4	0.02
Malignant tumor of breast	2.0	2.2	-0.02	2.1	1.6	0.04
Malignant tumor of colon	0.3	0.3	-0.01	0.3	0.3	-0.01
Malignant tumor of lung	0.5	<0.3	0.04	0.5	0.4	0.01
Malignant tumor of urinary bladder	0.6	0.7	-0.01	0.6	0.7	-0.02
Primary malignant neoplasm of prostate	1.5	1.6	-0.01	1.3	1.8	-0.04
Medication use						

(Continued)	Before stratification			After stratification		
Characteristic	T (%)	C (%)	StdDiff	T (%)	C (%)	StdDiff
Antibacterials for systemic use	27.4	22.2	0.12	25.3	24.5	0.02
Antidepressants	15.4	14.4	0.03	15.1	14.3	0.02
Antiepileptics	13.0	12.1	0.02	12.6	11.7	0.03
Antiinflammatory and antirheumatic products	16.8	19.7	-0.07	17.4	17.6	-0.01
Antineoplastic agents	3.4	3.0	0.03	3.3	2.6	0.04
Antipsoriatics	0.6	0.8	-0.02	0.7	0.8	-0.01
Antithrombotic agents	20.3	14.2	0.16	18.0	18.6	-0.01
Diuretics	<0.2	<0.3	-0.01	<0.2	<0.3	-0.03
Drugs for acid related disorders	22.8	21.9	0.02	22.2	22.8	-0.01
Drugs for obstructive airway diseases	15.5	13.7	0.05	14.5	14.6	0.00
Drugs used in diabetes	19.6	12.4	0.20	16.9	17.6	-0.02
Immunosuppressants	5.3	3.9	0.07	5.2	4.6	0.03
Lipid modifying agents	40.8	33.4	0.15	37.6	38.7	-0.02
Opioids	9.8	10.5	-0.02	10.0	9.9	0.00
Psycholeptics	13.9	13.4	0.02	13.7	13.3	0.01
Psychostimulants, agents used for adhd and nootropics	1.4	1.2	0.02	1.4	1.2	0.01

Supplementary Table 64: Baseline patient characteristcs for ARB +combo (T) and THZ +combo (C) prevalent-users in the CUIMC data source. We report proportion of initiators satisfying selected base-line characteristics and the standardized difference of population proportions (StdDiff) before and after stratification. Less extreme StdDiffs through stratification suggest improved balance between patient cohorts through propensity score adjustment.

	Before stratification			After stratification		
Characteristic	T (%)	C (%)	StdDiff	T (%)	C (%)	StdDiff
Age group						
15-19	<0.1	<0.1	-0.02	<0.1	<0.1	-0.02
20-24	0.3	0.1	0.03	0.3	0.1	0.04
25-29	0.3	0.2	0.02	0.4	0.2	0.03
30-34	0.7	0.9	-0.02	0.8	0.8	0.00
35-39	1.5	1.5	0.00	1.4	1.3	0.01
40-44	2.0	3.1	-0.07	2.3	2.6	-0.02
45-49	3.4	4.8	-0.07	3.9	4.0	-0.01
50-54	5.3	6.6	-0.05	5.8	5.8	0.00
55-59	8.5	9.9	-0.05	8.9	8.8	0.00
60-64	12.3	12.2	0.00	12.6	11.9	0.02
65-69	13.9	13.9	0.00	13.7	13.8	0.00
70-74	15.9	15.3	0.01	15.6	15.7	0.00
75-79	13.7	13.3	0.01	13.3	14.6	-0.04
80-84	10.7	9.3	0.05	10.2	10.4	-0.01
85-89	6.7	5.2	0.06	6.3	6.1	0.01
90-94	3.6	2.9	0.04	3.4	3.1	0.02
95-99	1.0	0.5	0.06	1.0	0.7	0.04
100-104	0.1	<0.1	0.01	<0.1	<0.1	0.01
10-14	<0.1	0.0		<0.1	0.0	
Gender: female	49.5	58.4	-0.18	53.9	53.0	0.02
Race						
race = Asian	2.0	0.7	0.11	1.5	1.2	0.03
race = Black or African American	7.9	10.6	-0.09	8.9	8.3	0.02
race = Other Race	0.1	0.1	0.00	0.1	0.1	0.01
race = White	36.4	31.0	0.11	33.7	35.0	-0.03
race = Unknown	2.2	2.6	-0.03	2.1	2.4	-0.02
race = Native Hawaiian or Other Pacific Islander	<0.1	<0.1	0.01	<0.1	<0.1	0.01
race = American Indian or Alaska Native	0.2	0.1	0.02	0.2	<0.1	0.03
race = Asian Indian	0.3	0.1	0.04	0.3	0.1	0.04
race = Bangladeshi	<0.1	0.0		<0.1	0.0	
race = Chinese	0.1	0.0		0.1	0.0	
race = Filipino	<0.1	<0.1	0.02	<0.1	<0.1	0.02
race = Indonesian	<0.1	0.0		<0.1	0.0	
race = Korean	0.1	0.0		0.1	0.0	
race = Laotian	0.5	0.5	0.00	0.5	0.5	0.01
race = Pakistani	<0.1	0.0		<0.1	0.0	
race = Thai	<0.1	<0.1	0.00	<0.1	<0.1	0.00
race = Micronesian	<0.1	0.0		<0.1	0.0	
race = Other Pacific Islander	0.5	0.5	-0.01	0.5	0.5	0.00

(Continued)	Before stratification			After stratification		
Characteristic	T (%)	C (%)	StdDiff	T (%)	C (%)	StdDiff
Ethnicity						
ethnicity = Hispanic or Latino	10.9	14.3	-0.10	12.5	12.1	0.01
ethnicity = Not Hispanic or Latino	37.2	33.0	0.09	35.2	35.7	-0.01
Medical history: General						
Acute respiratory disease	4.9	5.3	-0.02	4.9	4.9	0.00
Attention deficit hyperactivity disorder	0.1	0.2	-0.02	0.1	0.2	-0.01
Chronic liver disease	1.1	1.2	-0.02	1.1	1.3	-0.02
Chronic obstructive lung disease	5.0	3.9	0.05	4.6	4.2	0.02
Crohn’s disease	0.3	0.2	0.02	0.3	0.2	0.02
Dementia	2.4	1.9	0.03	2.3	2.0	0.02
Depressive disorder	5.3	6.1	-0.04	5.6	5.6	0.00
Diabetes mellitus	24.2	19.2	0.12	21.6	22.5	-0.02
Gastroesophageal reflux disease	9.0	8.1	0.03	8.7	8.7	0.00
Gastrointestinal hemorrhage	1.2	1.2	0.00	1.1	1.0	0.01
Human immunodeficiency virus infection	1.0	1.8	-0.07	1.2	1.3	-0.02
Hyperlipidemia	45.2	40.2	0.10	42.7	43.8	-0.02
Hypertensive disorder	68.3	71.3	-0.07	69.7	70.1	-0.01
Lesion of liver	1.2	1.4	-0.02	1.2	1.4	-0.01
Obesity	11.0	12.7	-0.05	11.6	11.9	-0.01
Osteoarthritis	12.5	14.8	-0.07	13.6	13.5	0.00
Pneumonia	2.5	2.3	0.01	2.3	2.2	0.01
Psoriasis	0.7	0.7	0.00	0.7	0.7	0.00
Renal impairment	14.2	12.3	0.06	13.3	13.3	0.00
Rheumatoid arthritis	0.9	0.9	0.00	1.0	1.0	0.00
Schizophrenia	0.1	0.3	-0.03	0.2	0.3	-0.02
Ulcerative colitis	0.1	0.2	-0.02	0.1	0.2	-0.02
Urinary tract infectious disease	3.3	3.0	0.02	3.3	2.8	0.03
Viral hepatitis C	0.7	0.7	0.00	0.7	0.7	0.00
Visual system disorder	11.1	11.8	-0.02	11.1	11.7	-0.02
Medical history: Cardiovascular disease						
Atrial fibrillation	13.3	10.0	0.10	11.6	11.9	-0.01
Cerebrovascular disease	8.3	6.5	0.07	7.1	7.8	-0.02
Coronary arteriosclerosis	23.8	14.9	0.22	19.4	20.3	-0.02
Heart disease	47.4	33.8	0.28	40.5	41.9	-0.03
Heart failure	16.2	7.9	0.26	12.7	9.9	0.09
Ischemic heart disease	9.4	5.8	0.14	7.5	7.8	-0.01
Peripheral vascular disease	6.1	4.6	0.07	5.3	5.9	-0.03
Pulmonary embolism	0.7	1.0	-0.03	0.7	1.1	-0.04
Venous thrombosis	1.4	1.7	-0.03	1.3	1.5	-0.02
Medical history: Neoplasms						
Hematologic neoplasm	1.8	1.7	0.01	1.8	1.5	0.02
Malignant lymphoma	1.2	1.1	0.01	1.2	1.0	0.01
Malignant neoplasm of anorectum	0.3	0.3	0.00	0.3	0.2	0.01
Malignant neoplastic disease	10.5	10.3	0.01	10.4	10.4	0.00
Malignant tumor of breast	1.6	1.9	-0.03	1.8	1.7	0.01

(Continued)	Before stratification			After stratification		
Characteristic	T (%)	C (%)	StdDiff	T (%)	C (%)	StdDiff
Malignant tumor of colon	0.4	0.5	-0.01	0.4	0.5	-0.02
Malignant tumor of lung	0.6	0.6	0.01	0.6	0.5	0.01
Malignant tumor of urinary bladder	0.7	0.6	0.00	0.6	0.7	-0.01
Primary malignant neoplasm of prostate	1.7	1.5	0.02	1.5	1.7	-0.01
Medication use						
Antibacterials for systemic use	32.6	29.7	0.06	30.8	31.4	-0.01
Antidepressants	18.0	17.3	0.02	17.6	17.9	-0.01
Antiepileptics	16.5	16.4	0.00	16.4	16.0	0.01
Antiinflammatory and antirheumatic products	18.6	21.9	-0.08	20.1	20.1	0.00
Antineoplastic agents	3.9	3.9	0.00	3.8	3.6	0.01
Antipsoriatics	1.3	1.3	0.00	1.3	1.3	0.00
Antithrombotic agents	37.3	30.4	0.15	33.6	34.3	-0.02
Diuretics	0.1	0.4	-0.06	0.1	0.3	-0.05
Drugs for acid related disorders	31.9	29.6	0.05	30.5	31.1	-0.01
Drugs for obstructive airway diseases	20.5	18.5	0.05	19.3	19.4	0.00
Drugs used in diabetes	25.0	21.0	0.10	22.9	23.6	-0.02
Immunosuppressants	6.7	5.9	0.03	6.5	6.3	0.00
Lipid modifying agents	52.3	46.0	0.13	48.8	50.1	-0.02
Opioids	15.3	16.4	-0.03	15.4	15.6	-0.01
Psycholeptics	18.0	18.1	0.00	17.5	18.1	-0.01
Psychostimulants, agents used for adhd and nootropics	1.4	1.3	0.01	1.4	1.3	0.00

Cohort balance and systematic error diagnostics

SIDIAP

Supplementary Figure 1: Preference score distributions, propensity score stratified (middle column) and matched (right column) covariate balance and negative control error distributions (bottom row) for ACE/ARB mono vs CCB/THZ mono in the SIDIAP data source.

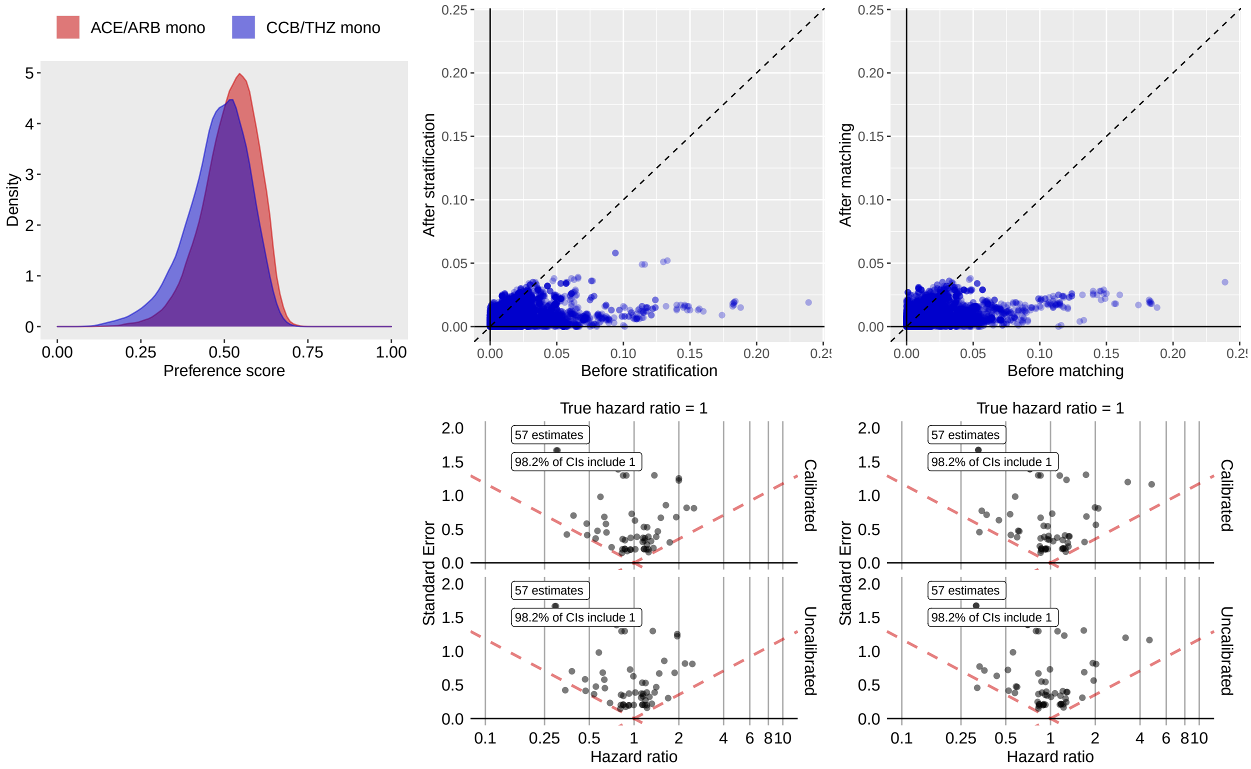

Supplementary Table 65: Most extreme patient characteristics balance diagnostics after stratification for ACE/ARB mono vs CCB/THZ mono in the SIDIAP data source.

	Before stratification			After stratification		
Characteristic (total count = 16388)	T (%)	C (%)	SDf	T (%)	C (%)	SDf
condition_era group during day -180 through 0 days relative to index: Transplanted kidney present	0.2	0.9	-0.09	0.2	0.6	-0.06
condition_era group during day -30 through 0 days relative to index: Transplanted kidney present	0.2	0.9	-0.09	0.2	0.6	-0.06
condition_era group during day -180 through 0 days relative to index: Potassium disorder	1.2	3.1	-0.13	1.4	2.1	-0.05
condition_era group during day -30 through 0 days relative to index: Potassium disorder	1.1	2.9	-0.13	1.3	2.0	-0.05
condition_era group during day -180 through 0 days relative to index: Hyperkalemia	0.8	2.2	-0.12	0.9	1.5	-0.05

(Continued)	T (%)	C (%)	SDf	T (%)	C (%)	SDf
condition_era group during day -30 through 0 days relative to index: Hyperkalemia	0.7	2.1	-0.11	0.9	1.4	-0.05
drug_era group during day -180 through 0 days relative to index: Mycophenolic Acid	0.1	0.5	-0.07	0.2	0.3	-0.04
condition_era group during day -30 through 0 days relative to index: Angioedema	0.1	0.3	-0.05	0.1	0.3	-0.04
drug_era group during day -180 through 0 days relative to index: mycophenolate mofetil	0.1	0.4	-0.06	0.1	0.3	-0.04
drug_era group during day -30 through 0 days relative to index: Mycophenolic Acid	0.1	0.5	-0.07	0.1	0.3	-0.04

Supplementary Table 66: Most extreme patient characteristics balance diagnostics after matching for ACE/ARB mono vs CCB/THZ mono in the SIDIAP data source.

	Before matching			After matching		
Characteristic (total count = 16378)	T (%)	C (%)	SDf	T (%)	C (%)	SDf
condition_era group during day -30 through 0 days relative to index: Angioedema	0.1	0.3	-0.05	0.1	0.3	-0.04
condition_era group during day -180 through 0 days relative to index: Angioedema	0.1	0.3	-0.05	0.1	0.3	-0.04
drug_era group during day 0 through 0 days relative to index: Haloperidol	0.2	0.1	0.03	0.3	0.1	0.04
drug_era group during day 0 through 0 days relative to index: Butyrophenone derivatives	0.2	0.1	0.03	0.3	0.1	0.04
CHADS2VASc	257.0	287.9	-0.24	291.4	286.9	0.04
condition_era group during day -30 through 0 days relative to index: Pre-eclampsia	0.0	0.1	-0.03	0.0	0.1	-0.04
drug_era group during day -180 through 0 days relative to index: Cromolyn	0.1	0.0	0.03	0.1	0.0	0.04
drug_era group during day -180 through 0 days relative to index: Antiallergic agents, excl. corticosteroids	0.1	0.0	0.03	0.1	0.0	0.04
condition_era group during day -180 through 0 days relative to index: Pityriasis versicolor	0.1	0.0	0.04	0.1	0.0	0.03
condition_era group during day -180 through 0 days relative to index: Pre-eclampsia	0.0	0.1	-0.03	0.0	0.1	-0.03

Supplementary Figure 2: Preference score distributions, propensity score stratified (middle column) and matched (right column) covariate balance and negative control error distributions (bottom row) for ACE/ARB +combo vs CCB/THZ +combo in the SIDIAP data source.

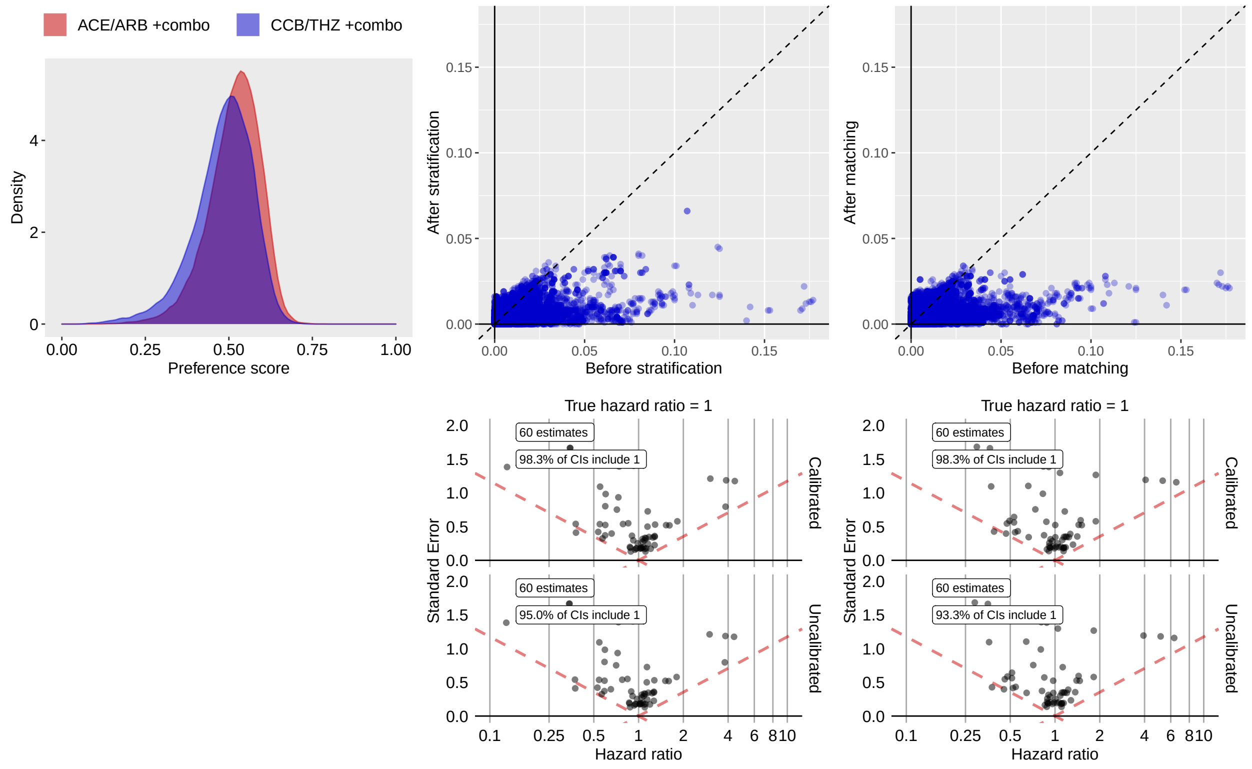

Supplementary Table 67: Most extreme patient characteristics balance diagnostics after stratification for ACE/ARB +combo vs CCB/THZ +combo in the SIDIAP data source.

	Before stratification			After stratification		
Characteristic (total count = 16941)	T (%)	C (%)	SDf	T (%)	C (%)	SDf
condition_era group during day -180 through 0 days relative to index: Transplanted kidney present	0.2	1.1	-0.11	0.3	0.8	-0.07
condition_era group during day -30 through 0 days relative to index: Transplanted kidney present	0.2	1.1	-0.11	0.3	0.8	-0.07
condition_era group during day -30 through 0 days relative to index: Potassium disorder	1.4	3.2	-0.12	1.6	2.3	-0.04
condition_era group during day -180 through 0 days relative to index: Potassium disorder	1.4	3.3	-0.12	1.7	2.3	-0.04
drug_era group during day 0 through 0 days relative to index: Selective immunosuppressants	0.4	1.1	-0.08	0.5	0.8	-0.04
drug_era group during day -180 through 0 days relative to index: mycophenolate mofetil	0.1	0.4	-0.06	0.1	0.3	-0.04
drug_era group during day -180 through 0 days relative to index: Selective immunosuppressants	0.5	1.2	-0.08	0.5	0.9	-0.04
drug_era group during day -30 through 0 days relative to index: Selective immunosuppressants	0.4	1.1	-0.08	0.5	0.8	-0.04

(Continued)	T (%)	C (%)	SDf	T (%)	C (%)	SDf
drug_era group during day 0 through 0 days relative to index: Trimethoprim	0.2	0.6	-0.07	0.2	0.4	-0.04
drug_era group during day 0 through 0 days relative to index: Sulfamethoxazole	0.2	0.6	-0.07	0.2	0.4	-0.04

Supplementary Table 68: Most extreme patient characteristics balance diagnostics after matching for ACE/ARB +combo vs CCB/THZ +combo in the SIDIAP data source.

	Before matching			After matching		
Characteristic (total count = 16933)	T (%)	C (%)	SDf	T (%)	C (%)	SDf
drug_era group during day -180 through 0 days relative to index: Cromolyn	0.1	0.0	0.03	0.1	0.0	0.03
drug_era group during day -180 through 0 days relative to index: Antiallergic agents, excl. corticosteroids	0.1	0.0	0.03	0.1	0.0	0.03
condition_era group during day -180 through 0 days relative to index: Pre-eclampsia	0.0	0.1	-0.03	0.0	0.1	-0.03
condition_era group during day -180 through 0 days relative to index: Pityriasis	0.2	0.1	0.03	0.2	0.1	0.03
condition_era group during day -180 through 0 days relative to index: Pityriasis versicolor	0.1	0.0	0.03	0.1	0.0	0.03
condition_era group during day -30 through 0 days relative to index: Pre-eclampsia	0.0	0.1	-0.03	0.0	0.1	-0.03
CHADS2VASc	272.7	296.0	-0.17	299.5	295.5	0.03
condition_era group during day -180 through 0 days relative to index: Atherosclerosis of renal artery	0.0	0.2	-0.04	0.1	0.2	-0.03
condition_era group during day -30 through 0 days relative to index: Atherosclerosis of renal artery	0.0	0.2	-0.04	0.1	0.2	-0.03
condition_era group during day -30 through 0 days relative to index: Angioedema	0.1	0.3	-0.05	0.2	0.3	-0.03

Supplementary Figure 3: Preference score distributions, propensity score stratified (middle column) and matched (right column) covariate balance and negative control error distributions (bottom row) for ACE/ARB mono vs CCB mono in the SIDIAP data source.

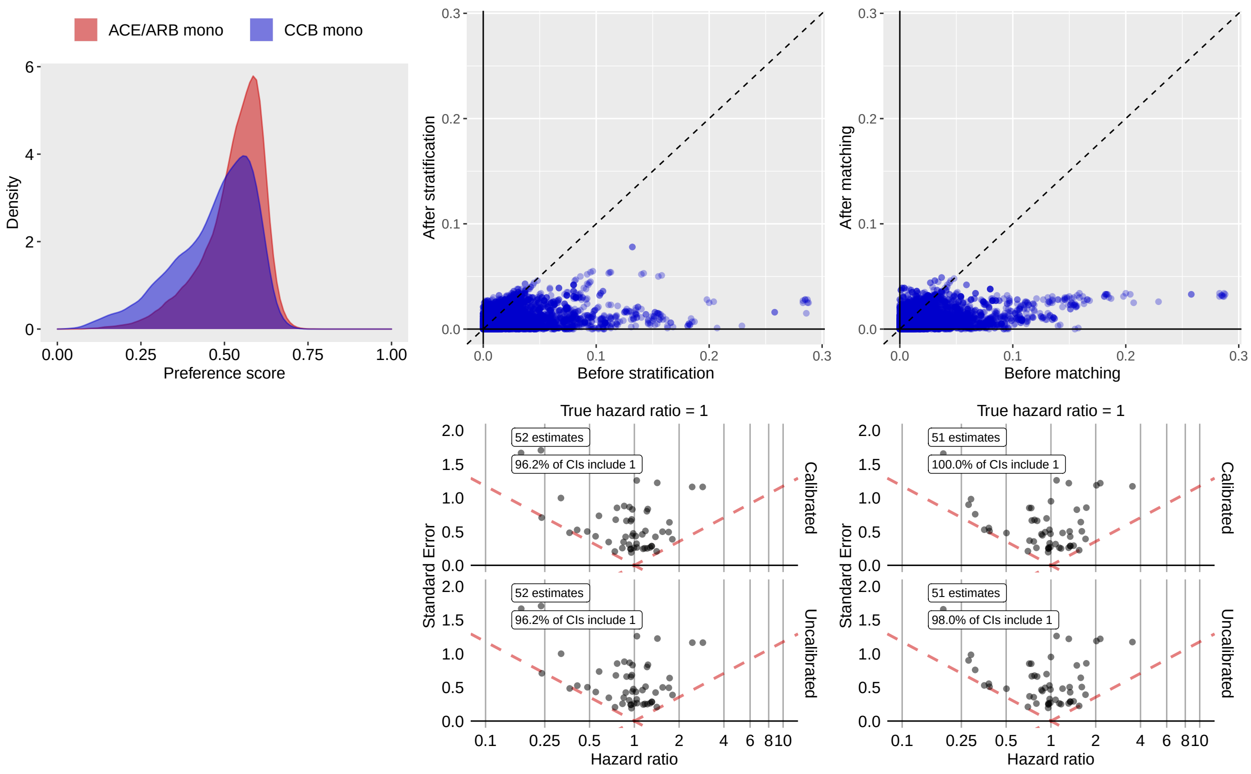

Supplementary Table 69: Most extreme patient characteristics balance diagnostics after stratification for ACE/ARB mono vs CCB mono in the SIDIAP data source.

	Before stratification			After stratification		
Characteristic (total count = 16182)	T (%)	C (%)	SDf	T (%)	C (%)	SDf
condition_era group during day -180 through 0 days relative to index: Transplanted kidney present	0.2	1.3	-0.13	0.2	0.8	-0.08
condition_era group during day -30 through 0 days relative to index: Transplanted kidney present	0.2	1.3	-0.13	0.2	0.8	-0.08
drug_era group during day -180 through 0 days relative to index: Mycophenolic Acid	0.1	0.8	-0.10	0.2	0.5	-0.06
drug_era group during day -30 through 0 days relative to index: Mycophenolic Acid	0.1	0.7	-0.10	0.1	0.4	-0.05
drug_era group during day -180 through 0 days relative to index: Selective immunosuppressants	0.4	1.5	-0.11	0.5	0.9	-0.05
drug_era group during day -30 through 0 days relative to index: Selective immunosuppressants	0.4	1.4	-0.11	0.4	0.9	-0.05
condition_era group during day -180 through 0 days relative to index: Hyperkalemia	0.8	2.6	-0.14	0.9	1.5	-0.05
drug_era group during day 0 through 0 days relative to index: Selective immunosuppressants	0.4	1.4	-0.11	0.4	0.8	-0.05

(Continued)	T (%)	C (%)	SDf	T (%)	C (%)	SDf
condition_era group during day -30 through 0 days relative to index: Hyperkalemia	0.7	2.5	-0.14	0.9	1.5	-0.05
drug_era group during day 0 through 0 days relative to index: Mycophenolic Acid	0.1	0.7	-0.09	0.1	0.4	-0.05

Supplementary Table 70: Most extreme patient characteristics balance diagnostics after matching for ACE/ARB mono vs CCB mono in the SIDIAP data source.

	Before matching			After matching		
Characteristic (total count = 16176)	T (%)	C (%)	SDf	T (%)	C (%)	SDf
drug_era group during day 0 through 0 days relative to index: Haloperidol	0.2	0.1	0.04	0.3	0.1	0.05
drug_era group during day 0 through 0 days relative to index: Butyrophenone derivatives	0.2	0.1	0.04	0.3	0.1	0.05
condition_era group during day -30 through 0 days relative to index: Pre-eclampsia	0.0	0.2	-0.05	0.0	0.2	-0.05
condition_era group during day -180 through 0 days relative to index: Pre-eclampsia	0.0	0.2	-0.04	0.0	0.2	-0.05
drug_era group during day -30 through 0 days relative to index: Haloperidol	0.3	0.1	0.03	0.4	0.1	0.05
drug_era group during day -30 through 0 days relative to index: Butyrophenone derivatives	0.3	0.1	0.03	0.4	0.1	0.05
drug_era group during day -180 through 0 days relative to index: silodosin	0.3	0.1	0.03	0.4	0.1	0.04
drug_era group during day -180 through 0 days relative to index: Haloperidol	0.3	0.2	0.03	0.4	0.2	0.04
condition_era group during day -30 through 0 days relative to index: Pregnancy-induced hypertension	0.1	0.2	-0.04	0.1	0.2	-0.04
drug_era group during day -180 through 0 days relative to index: Butyrophenone derivatives	0.3	0.2	0.03	0.4	0.2	0.04

Supplementary Figure 4: Preference score distributions, propensity score stratified (middle column) and matched (right column) covariate balance and negative control error distributions (bottom row) for ACE/ARB +combo vs CCB +combo in the SIDIAP data source.

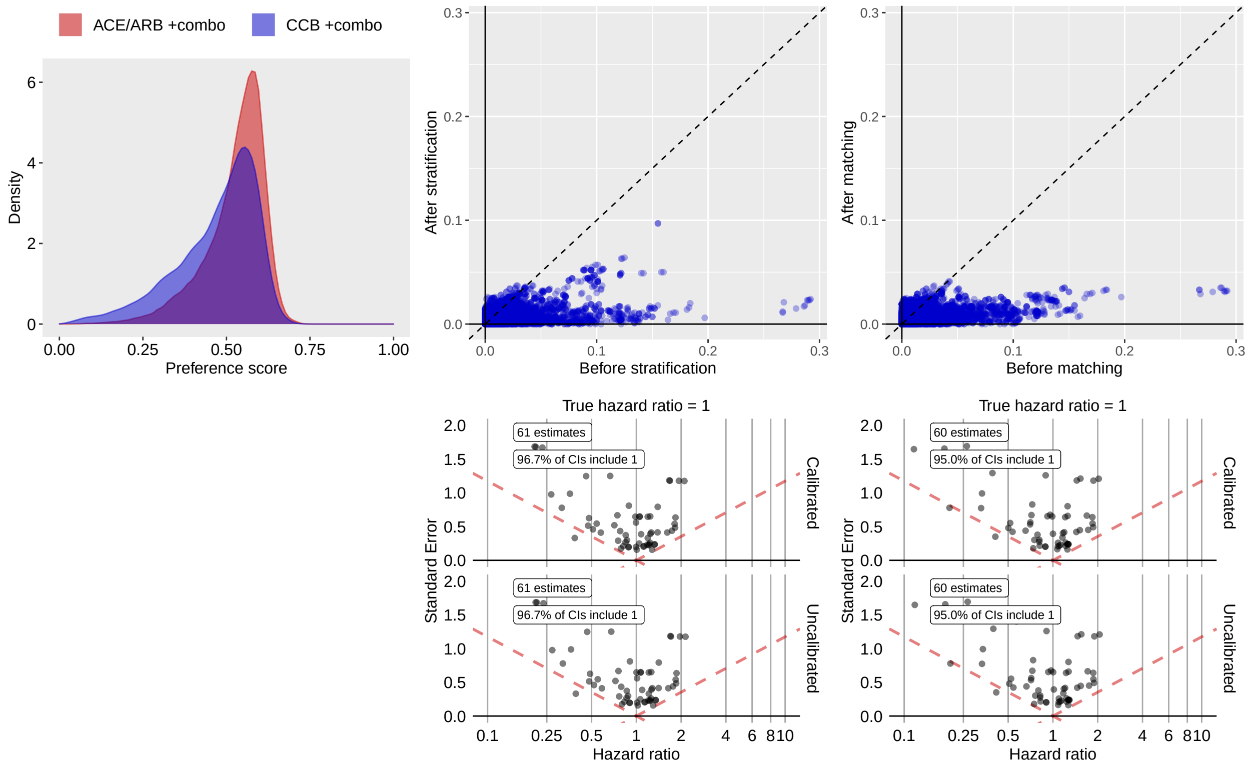

Supplementary Table 71: Most extreme patient characteristics balance diagnostics after stratification for ACE/ARB +combo vs CCB +combo in the SIDIAP data source.

Characteristic (total count = 17578)	Before stratification			After stratification		
	T (%)	C (%)	SDf	T (%)	C (%)	SDf
condition_era group during day -180 through 0 days relative to index: Transplanted kidney present	0.2	1.7	-0.15	0.2	0.9	-0.10
condition_era group during day -30 through 0 days relative to index: Transplanted kidney present	0.2	1.7	-0.15	0.2	0.9	-0.10
drug_era group during day -180 through 0 days relative to index: Selective immunosuppressants	0.4	1.7	-0.12	0.5	1.0	-0.06
drug_era group during day -30 through 0 days relative to index: Selective immunosuppressants	0.4	1.6	-0.12	0.4	1.0	-0.06
drug_era group during day 0 through 0 days relative to index: Selective immunosuppressants	0.4	1.6	-0.12	0.4	0.9	-0.06
drug_era group during day -180 through 0 days relative to index: Mycophenolic Acid	0.1	0.9	-0.10	0.2	0.5	-0.06
drug_era group during day -30 through 0 days relative to index: Mycophenolic Acid	0.1	0.8	-0.10	0.2	0.5	-0.06
drug_era group during day 0 through 0 days relative to index: Mycophenolic Acid	0.1	0.8	-0.10	0.2	0.5	-0.06

(Continued)	T (%)	C (%)	SDf	T (%)	C (%)	SDf
drug_era group during day -180 through 0 days relative to index: mycophenolate mofetil	0.1	0.6	-0.09	0.1	0.4	-0.05
drug_era group during day 0 through 0 days relative to index: Tacrolimus	0.4	1.3	-0.10	0.4	0.8	-0.05

Supplementary Table 72: Most extreme patient characteristics balance diagnostics after matching for ACE/ARB +combo vs CCB +combo in the SIDIAP data source.

	Before matching			After matching		
Characteristic (total count = 17556)	T (%)	C (%)	SDf	T (%)	C (%)	SDf
condition_era group during day -180 through 0 days relative to index: Pre-eclampsia	0.0	0.1	-0.04	0.0	0.1	-0.04
condition_era group during day -30 through 0 days relative to index: Pre-eclampsia	0.0	0.1	-0.04	0.0	0.1	-0.04
condition_era group during day -180 through 0 days relative to index: Pregnancy-induced hypertension	0.1	0.2	-0.04	0.1	0.2	-0.04
condition_era group during day -30 through 0 days relative to index: Pregnancy-induced hypertension	0.0	0.2	-0.04	0.1	0.2	-0.04
condition_era group during day -180 through 0 days relative to index: Degenerative disorder of muscle	0.2	0.1	0.03	0.3	0.1	0.04
condition_era group during day -30 through 0 days relative to index: Degenerative disorder of muscle	0.2	0.1	0.03	0.2	0.1	0.04
condition_era group during day -180 through 0 days relative to index: Chronic disease of genitourinary system	11.2	21.7	-0.29	22.5	21.1	0.04
condition_era group during day -30 through 0 days relative to index: Chronic disease of genitourinary system	11.1	21.6	-0.29	22.4	21.0	0.04
drug_era group during day -30 through 0 days relative to index: ANTITHROMBOTIC AGENTS	21.8	28.1	-0.15	29.5	27.9	0.04
drug_era group during day -30 through 0 days relative to index: ANTITHROMBOTIC AGENTS	21.8	28.1	-0.15	29.5	27.9	0.04

Supplementary Figure 5: Preference score distributions, propensity score stratified (middle column) and matched (right column) covariate balance and negative control error distributions (bottom row) for ACE/ARB mono vs THZ mono in the SIDIAP data source.

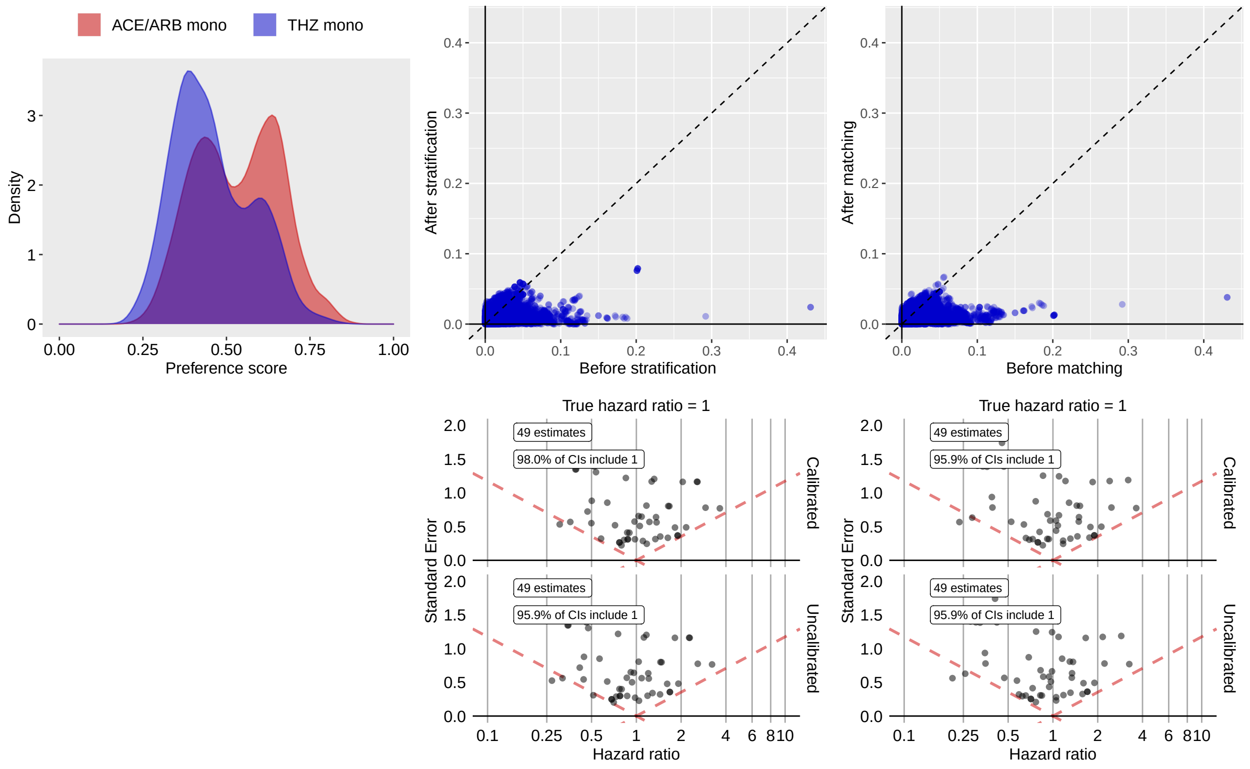

Supplementary Table 73: Most extreme patient characteristics balance diagnostics after stratification for ACE/ARB mono vs THZ mono in the SIDIAP data source.

	Before stratification			After stratification		
Characteristic (total count = 16123)	T (%)	C (%)	SDf	T (%)	C (%)	SDf
condition_era group during day -30 through 0 days relative to index: Gout	4.9	1.4	0.20	4.5	3.0	0.08
condition_era group during day -30 through 0 days relative to index: Blood urate abnormal	4.9	1.4	0.20	4.5	3.0	0.08
condition_era group during day -30 through 0 days relative to index: Blood urate raised	4.9	1.4	0.20	4.5	3.0	0.08
condition_era group during day -30 through 0 days relative to index: Increased uric acid level	4.9	1.4	0.20	4.5	3.0	0.08
condition_era group during day -180 through 0 days relative to index: Gout	4.9	1.4	0.20	4.6	3.1	0.08
condition_era group during day -180 through 0 days relative to index: Blood urate abnormal	4.9	1.4	0.20	4.6	3.1	0.08
condition_era group during day -180 through 0 days relative to index: Blood urate raised	4.9	1.4	0.20	4.6	3.1	0.08
condition_era group during day -180 through 0 days relative to index: Increased uric acid level	4.9	1.4	0.20	4.6	3.1	0.08

(Continued)	T (%)	C (%)	SDf	T (%)	C (%)	SDf
drug_era group during day 0 through 0 days relative to index: potassium citrate	0.0	0.2	-0.05	0.0	0.3	-0.06
drug_era group during day 0 through 0 days relative to index: Ascorbic Acid	0.0	0.2	-0.05	0.0	0.3	-0.06

Supplementary Table 74: Most extreme patient characteristics balance diagnostics after matching for ACE/ARB mono vs THZ mono in the SIDIAP data source.

	Before matching			After matching		
Characteristic (total count = 15690)	T (%)	C (%)	SDf	T (%)	C (%)	SDf
condition_era group during day -180 through 0 days relative to index: Hypo-osmolality and or hyponatremia	0.4	0.1	0.06	0.5	0.1	0.07
condition_era group during day -30 through 0 days relative to index: Hypo-osmolality and or hyponatremia	0.4	0.1	0.06	0.5	0.1	0.07
drug_era group during day -30 through 0 days relative to index: rizatriptan	0.3	-0.1	0.05	0.3	-0.1	0.06
drug_era group during day 0 through 0 days relative to index: rizatriptan	0.2	-0.1	0.05	0.3	-0.1	0.06
drug_era group during day -180 through 0 days relative to index: rizatriptan	0.3	0.1	0.04	0.4	0.1	0.06
condition_era group during day -180 through 0 days relative to index: Transient cerebral ischemia	0.6	0.2	0.06	0.6	0.2	0.05
condition_era group during day -30 through 0 days relative to index: Transient cerebral ischemia	0.6	0.2	0.05	0.5	0.2	0.05
drug_era group during day -180 through 0 days relative to index: Clarithromycin	0.4	0.2	0.04	0.5	0.2	0.05
drug_era group during day -180 through 0 days relative to index: potassium citrate	0.1	0.3	-0.05	0.1	0.3	-0.05
drug_era group during day 0 through 0 days relative to index: rifaximin	0.3	0.2	0.03	0.4	0.2	0.05

Supplementary Figure 6: Preference score distributions, propensity score stratified (middle column) and matched (right column) covariate balance and negative control error distributions (bottom row) for ACE/ARB +combo vs THZ +combo in the SIDIAP data source.

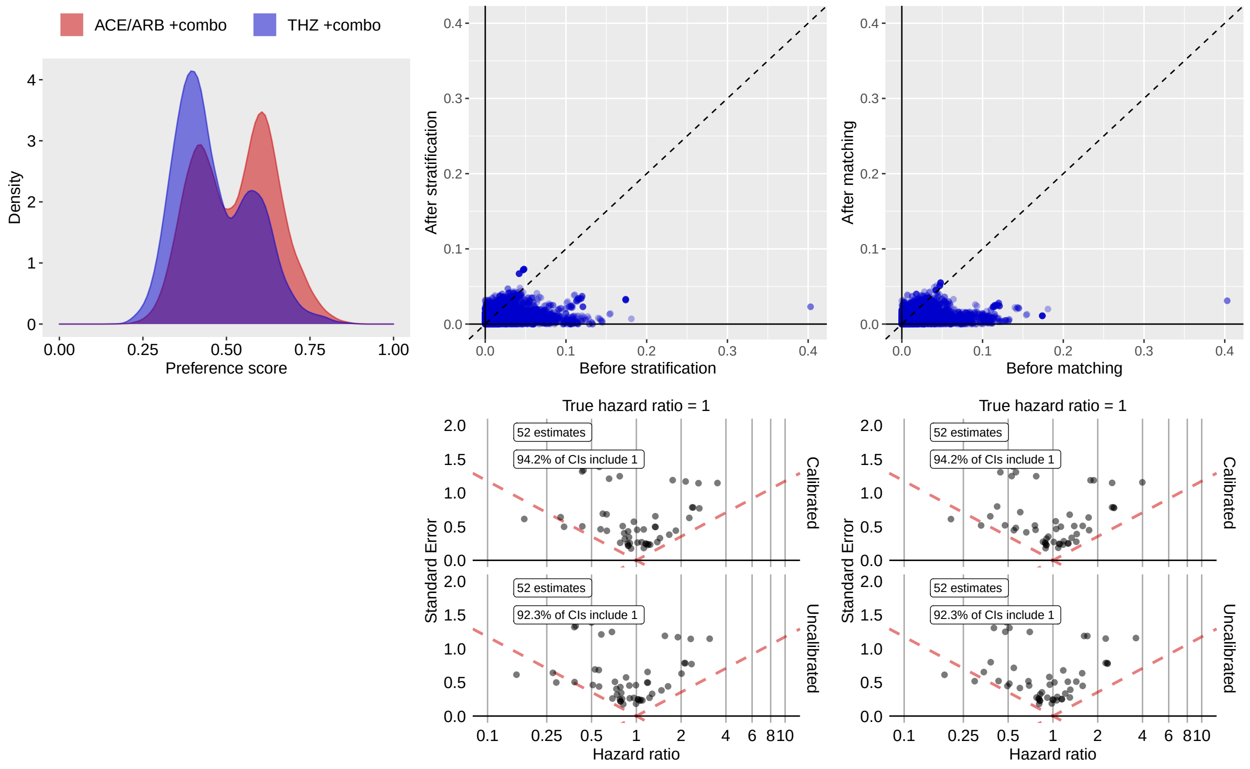

Supplementary Table 75: Most extreme patient characteristics balance diagnostics after stratification for ACE/ARB +combo vs THZ +combo in the SIDIAP data source.

Characteristic (total count = 17025)	Before stratification			After stratification		
	T (%)	C (%)	SDf	T (%)	C (%)	SDf
drug_era group during day 0 through 0 days relative to index: potassium citrate	0.1	0.3	-0.05	0.1	0.5	-0.07
drug_era group during day 0 through 0 days relative to index: Ascorbic Acid	0.1	0.3	-0.05	0.1	0.5	-0.07
drug_era group during day 0 through 0 days relative to index: ASCORBIC ACID (VITAMIN C), INCL. COMBINATIONS	0.1	0.3	-0.05	0.1	0.5	-0.07
drug_era group during day 0 through 0 days relative to index: Ascorbic acid (vitamin C), plain	0.1	0.3	-0.05	0.1	0.5	-0.07
drug_era group during day 0 through 0 days relative to index: POTASSIUM	0.1	0.3	-0.05	0.1	0.5	-0.07
drug_era group during day 0 through 0 days relative to index: Potassium	0.1	0.3	-0.05	0.1	0.5	-0.07
drug_era group during day -180 through 0 days relative to index: potassium citrate	0.1	0.4	-0.05	0.1	0.6	-0.07
drug_era group during day -180 through 0 days relative to index: Ascorbic Acid	0.1	0.4	-0.05	0.1	0.6	-0.07

(Continued)	T (%)	C (%)	SDf	T (%)	C (%)	SDf
drug_era group during day -180 through 0 days relative to index: ASCORBIC ACID (VITAMIN C), INCL. COMBINATIONS	0.1	0.4	-0.05	0.1	0.6	-0.07
drug_era group during day -180 through 0 days relative to index: Ascorbic acid (vitamin C), plain	0.1	0.4	-0.05	0.1	0.6	-0.07

Supplementary Table 76: Most extreme patient characteristics balance diagnostics after matching for ACE/ARB +combo vs THZ +combo in the SIDIAP data source.

	Before matching			After matching		
Characteristic (total count = 16689)	T (%)	C (%)	SDf	T (%)	C (%)	SDf
drug_era group during day 0 through 0 days relative to index: potassium citrate	0.1	0.3	-0.05	0.1	0.3	-0.06
drug_era group during day 0 through 0 days relative to index: Ascorbic Acid	0.1	0.3	-0.05	0.1	0.3	-0.06
drug_era group during day 0 through 0 days relative to index: ASCORBIC ACID (VITAMIN C), INCL. COMBINATIONS	0.1	0.3	-0.05	0.1	0.3	-0.06
drug_era group during day 0 through 0 days relative to index: Ascorbic acid (vitamin C), plain	0.1	0.3	-0.05	0.1	0.3	-0.06
drug_era group during day 0 through 0 days relative to index: POTASSIUM	0.1	0.3	-0.05	0.1	0.3	-0.06
drug_era group during day 0 through 0 days relative to index: Potassium	0.1	0.3	-0.05	0.1	0.3	-0.06
condition_era group during day -180 through 0 days relative to index: Hypo-osmolality and or hyponatremia	0.5	0.2	0.04	0.6	0.2	0.05
drug_era group during day -180 through 0 days relative to index: potassium citrate	0.1	0.4	-0.05	0.1	0.4	-0.05
drug_era group during day -180 through 0 days relative to index: Ascorbic Acid	0.1	0.4	-0.05	0.1	0.4	-0.05
drug_era group during day -180 through 0 days relative to index: ASCORBIC ACID (VITAMIN C), INCL. COMBINATIONS	0.1	0.4	-0.05	0.1	0.4	-0.05

Supplementary Figure 7: Preference score distributions, propensity score stratified (middle column) and matched (right column) covariate balance and negative control error distributions (bottom row) for ACE mono vs ARB mono in the SIDIAP data source.

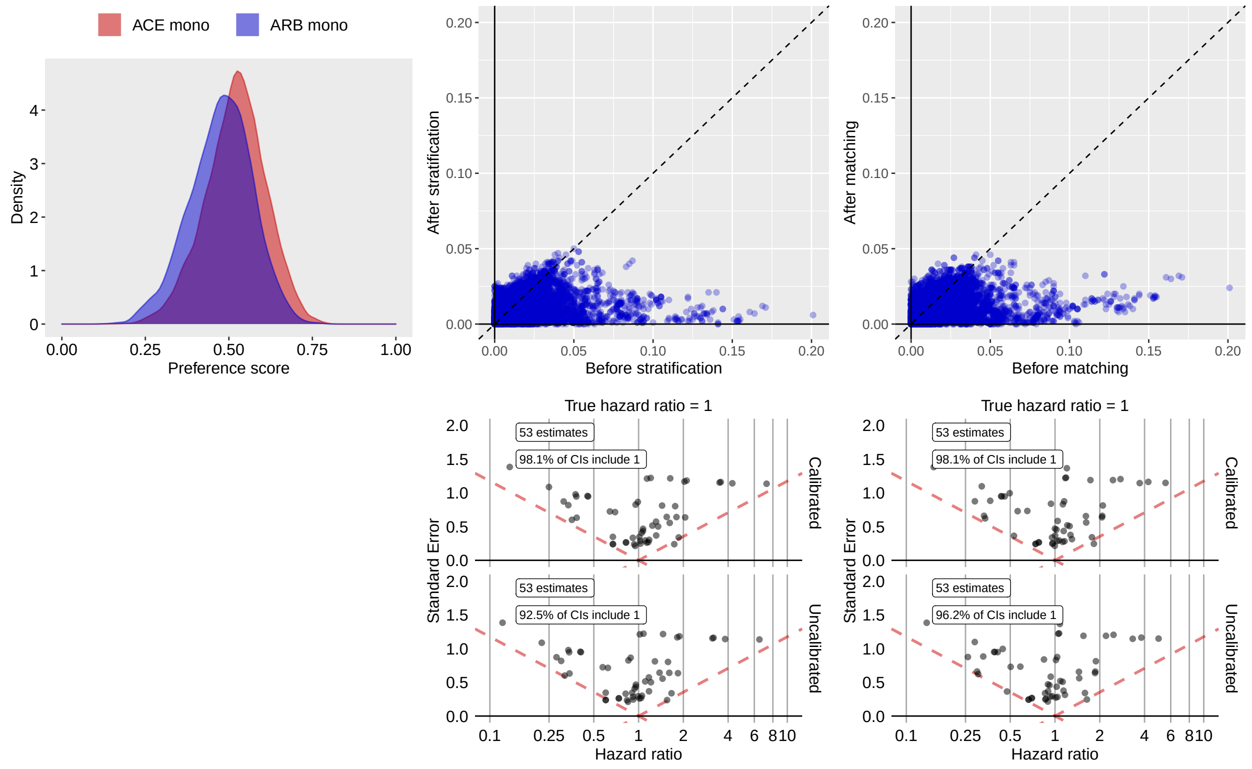

Supplementary Table 77: Most extreme patient characteristics balance diagnostics after stratification for ACE mono vs ARB mono in the SIDIAP data source.

	Before stratification			After stratification		
Characteristic (total count = 15840)	T (%)	C (%)	SDf	T (%)	C (%)	SDf
condition_era group during day -180 through 0 days relative to index: Angioedema	0.1	0.3	-0.05	0.1	0.3	-0.05
condition_era group during day -180 through 0 days relative to index: Multiple congenital cysts of kidney	0.3	0.6	-0.05	0.3	0.6	-0.05
condition_era group during day -30 through 0 days relative to index: Multiple congenital cysts of kidney	0.3	0.6	-0.05	0.3	0.6	-0.05
condition_era group during day -180 through 0 days relative to index: Spontaneous intracranial hemorrhage	0.3	0.1	0.04	0.3	0.1	0.05
condition_era group during day -30 through 0 days relative to index: Angioedema	0.1	0.2	-0.04	0.1	0.2	-0.05
condition_era group during day -30 through 0 days relative to index: Spontaneous intracranial hemorrhage	0.3	0.1	0.04	0.3	0.1	0.05
drug_era group during day -30 through 0 days relative to index: Tobramycin	0.5	0.2	0.04	0.5	0.2	0.04
condition_era group during day -180 through 0 days relative to index: Cocaine dependence	0.3	-0.1	0.05	0.2	0.1	0.04

(Continued)	T (%)	C (%)	SDf	T (%)	C (%)	SDf
drug_era group during day 0 through 0 days relative to index: rosuvastatin	0.5	1.3	-0.09	0.6	0.9	-0.04
condition_era group during day -180 through 0 days relative to index: Moderate major depression, single episode	0.2	-0.1	0.04	0.2	-0.1	0.04

Supplementary Table 78: Most extreme patient characteristics balance diagnostics after matching for ACE mono vs ARB mono in the SIDIAP data source.

	Before matching			After matching		
Characteristic (total count = 15748)	T (%)	C (%)	SDf	T (%)	C (%)	SDf
condition_era group during day -180 through 0 days relative to index: Angioedema	0.1	0.3	-0.05	0.1	0.3	-0.05
drug_era group during day -30 through 0 days relative to index: Tobramycin	0.5	0.2	0.04	0.5	0.2	0.05
condition_era group during day -180 through 0 days relative to index: Moderate major depression, single episode	0.2	-0.1	0.04	0.2	-0.1	0.04
condition_era group during day -30 through 0 days relative to index: Moderate major depression, single episode	0.2	-0.1	0.04	0.2	-0.1	0.04
condition_era group during day -180 through 0 days relative to index: Collapse of vertebra	0.4	0.2	0.03	0.5	0.2	0.04
condition_era group during day -30 through 0 days relative to index: Collapse of vertebra	0.4	0.2	0.03	0.5	0.2	0.04
condition_era group during day -30 through 0 days relative to index: Angioedema	0.1	0.2	-0.04	0.1	0.2	-0.04
drug_era group during day -180 through 0 days relative to index: mycophenolate mofetil	0.0	0.3	-0.06	0.1	0.3	-0.04
drug_era group during day -30 through 0 days relative to index: mycophenolate mofetil	0.0	0.3	-0.06	0.1	0.3	-0.04
drug_era group during day -30 through 0 days relative to index: Calcineurin inhibitors	0.4	0.9	-0.07	0.5	0.9	-0.04

Supplementary Figure 8: Preference score distributions, propensity score stratified (middle column) and matched (right column) covariate balance and negative control error distributions (bottom row) for ACE +combo vs ARB +combo in the SIDIAP data source.

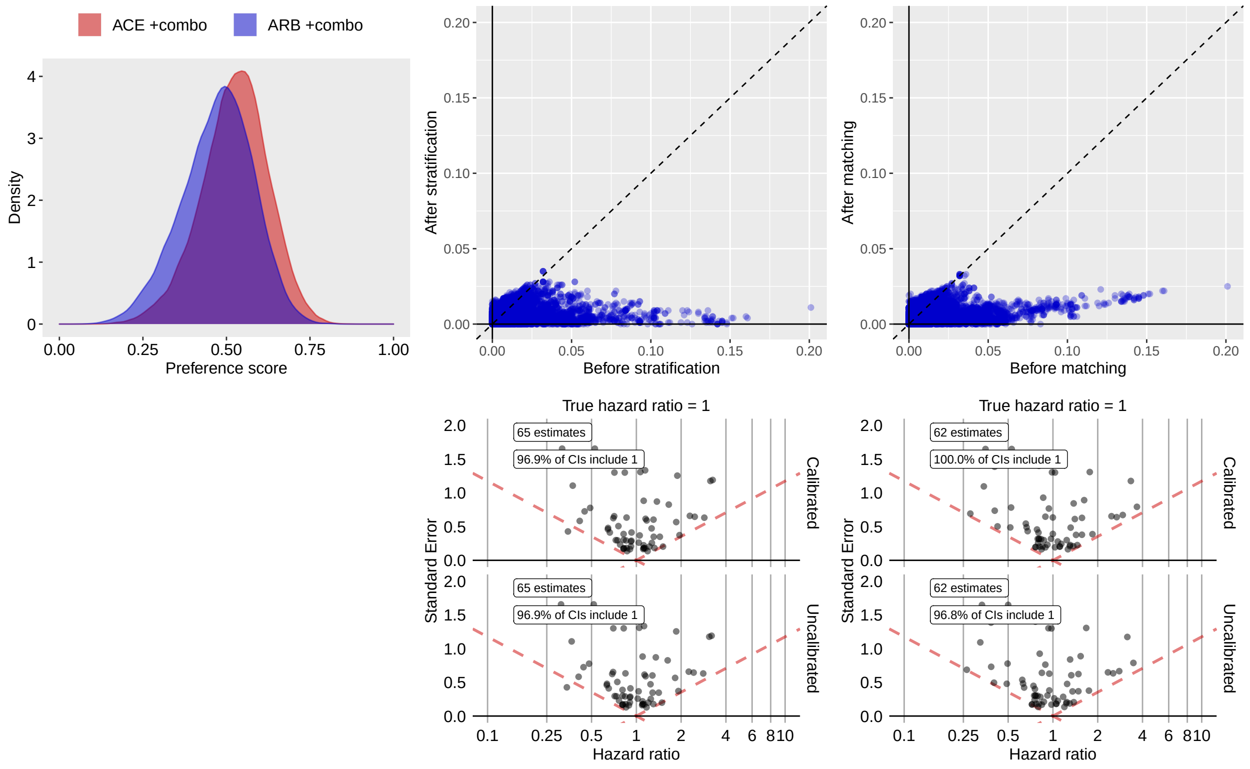

Supplementary Table 79: Most extreme patient characteristics balance diagnostics after stratification for ACE +combo vs ARB +combo in the SIDIAP data source.

	Before stratification			After stratification		
Characteristic (total count = 17908)	T (%)	C (%)	SDf	T (%)	C (%)	SDf
condition_era group during day -180 through 0 days relative to index: Disorder due to and following poisoning	0.0	0.1	-0.03	0.0	0.1	-0.04
condition_era group during day -30 through 0 days relative to index: Disorder due to and following poisoning	0.0	0.1	-0.03	0.0	0.1	-0.04
condition_era group during day -180 through 0 days relative to index: Disorder due to and following drug poisoning	0.0	0.1	-0.03	0.0	0.1	-0.04
condition_era group during day -30 through 0 days relative to index: Disorder due to and following drug poisoning	0.0	0.1	-0.03	0.0	0.1	-0.04
condition_era group during day -180 through 0 days relative to index: Atherosclerosis of renal artery	0.0	0.1	-0.04	0.0	0.1	-0.03
condition_era group during day -180 through 0 days relative to index: Acquired hypermelanotic disorder	0.1	0.0	0.03	0.1	0.0	0.03
condition_era group during day -30 through 0 days relative to index: Acquired hypermelanotic disorder	0.1	0.0	0.03	0.1	0.0	0.03
condition_era group during day -180 through 0 days relative to index: Acquired disorder of skin color	0.1	0.0	0.03	0.1	0.0	0.03

(Continued)	T (%)	C (%)	SDf	T (%)	C (%)	SDf
condition_era group during day -30 through 0 days relative to index: Acquired disorder of skin color	0.1	0.0	0.03	0.1	0.0	0.03
condition_era group during day -180 through 0 days relative to index: Chloasma	0.1	0.0	0.03	0.1	0.0	0.03

Supplementary Table 80: Most extreme patient characteristics balance diagnostics after matching for ACE +combo vs ARB +combo in the SIDIAP data source.

	Before matching			After matching		
Characteristic (total count = 17898)	T (%)	C (%)	SDf	T (%)	C (%)	SDf
condition_era group during day -180 through 0 days relative to index: Atherosclerosis of renal artery	0.0	0.1	-0.04	0.0	0.1	-0.03
condition_era group during day -180 through 0 days relative to index: Disorder due to and following poisoning	0.0	0.1	-0.03	0.0	0.1	-0.03
condition_era group during day -30 through 0 days relative to index: Disorder due to and following poisoning	0.0	0.1	-0.03	0.0	0.1	-0.03
condition_era group during day -180 through 0 days relative to index: Disorder due to and following drug poisoning	0.0	0.1	-0.03	0.0	0.1	-0.03
condition_era group during day -30 through 0 days relative to index: Disorder due to and following drug poisoning	0.0	0.1	-0.03	0.0	0.1	-0.03
condition_era group during day -30 through 0 days relative to index: Atherosclerosis of renal artery	0.0	0.1	-0.04	0.0	0.1	-0.03
condition_era group during day -180 through 0 days relative to index: Acquired hypermelanotic disorder	0.1	0.0	0.03	0.1	0.0	0.03
condition_era group during day -30 through 0 days relative to index: Acquired hypermelanotic disorder	0.1	0.0	0.03	0.1	0.0	0.03
condition_era group during day -180 through 0 days relative to index: Acquired disorder of skin color	0.1	0.0	0.03	0.1	0.0	0.03
condition_era group during day -30 through 0 days relative to index: Acquired disorder of skin color	0.1	0.0	0.03	0.1	0.0	0.03

Supplementary Figure 9: Preference score distributions, propensity score stratified (middle column) and matched (right column) covariate balance and negative control error distributions (bottom row) for ACE mono vs CCB/THZ mono in the SIDIAP data source.

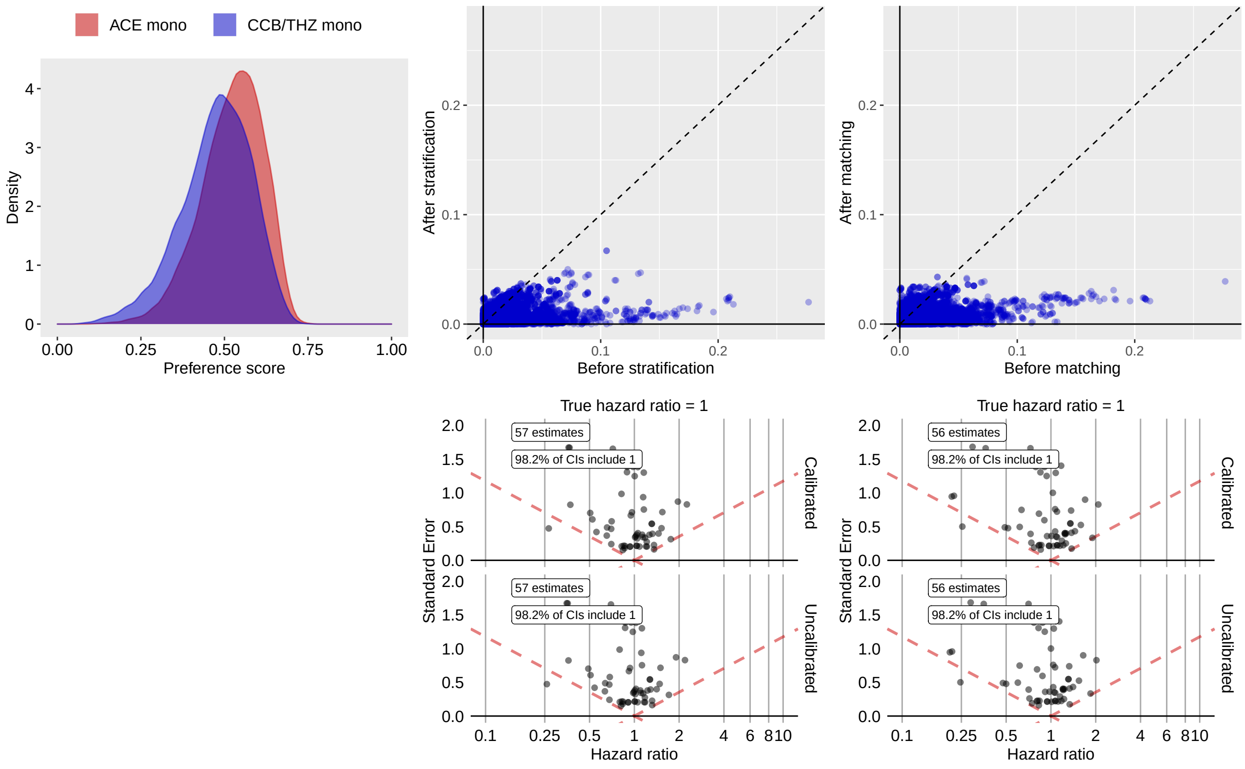

Supplementary Table 81: Most extreme patient characteristics balance diagnostics after stratification for ACE mono vs CCB/THZ mono in the SIDIAP data source.

	Before stratification			After stratification		
Characteristic (total count = 16044)	T (%)	C (%)	SDf	T (%)	C (%)	SDf
condition_era group during day -180 through 0 days relative to index: Transplanted kidney present	0.1	0.9	-0.10	0.2	0.6	-0.07
condition_era group during day -30 through 0 days relative to index: Transplanted kidney present	0.1	0.9	-0.10	0.2	0.6	-0.07
drug_era group during day -180 through 0 days relative to index: mycophenolate mofetil	0.0	0.4	-0.07	0.1	0.3	-0.05
condition_era group during day -180 through 0 days relative to index: Potassium disorder	1.1	3.1	-0.13	1.5	2.1	-0.05
drug_era group during day -30 through 0 days relative to index: mycophenolate mofetil	0.0	0.4	-0.07	0.1	0.2	-0.05
drug_era group during day -30 through 0 days relative to index: Mycophenolic Acid	0.1	0.5	-0.07	0.1	0.3	-0.05
condition_era group during day -30 through 0 days relative to index: Potassium disorder	1.1	2.9	-0.13	1.4	2.0	-0.05
drug_era group during day 0 through 0 days relative to index: mycophenolate mofetil	0.0	0.4	-0.07	0.1	0.2	-0.05

(Continued)	T (%)	C (%)	SDf	T (%)	C (%)	SDf
drug_era group during day -180 through 0 days relative to index: Mycophenolic Acid	0.1	0.5	-0.07	0.1	0.3	-0.05
drug_era group during day 0 through 0 days relative to index: Mycophenolic Acid	0.1	0.5	-0.07	0.1	0.3	-0.04

Supplementary Table 82: Most extreme patient characteristics balance diagnostics after matching for ACE mono vs CCB/THZ mono in the SIDIAP data source.

	Before matching			After matching		
Characteristic (total count = 16005)	T (%)	C (%)	SDf	T (%)	C (%)	SDf
drug_era group during day 0 through 0 days relative to index: Haloperidol	0.2	0.1	0.03	0.3	0.1	0.04
drug_era group during day 0 through 0 days relative to index: Butyrophenone derivatives	0.2	0.1	0.03	0.3	0.1	0.04
condition_era group during day -30 through 0 days relative to index: Angioedema	0.1	0.3	-0.06	0.1	0.3	-0.04
condition_era group during day -180 through 0 days relative to index: Angioedema	0.1	0.3	-0.06	0.1	0.3	-0.04
CHADS2VASc	252.4	287.9	-0.28	290.1	285.1	0.04
drug_era group during day -180 through 0 days relative to index: mycophenolate mofetil	0.0	0.4	-0.07	0.1	0.3	-0.04
drug_era group during day -30 through 0 days relative to index: mycophenolate mofetil	0.0	0.4	-0.07	0.1	0.2	-0.04
drug_era group during day 0 through 0 days relative to index: mycophenolate mofetil	0.0	0.4	-0.07	0.1	0.2	-0.04
drug_era group during day -180 through 0 days relative to index: paricalcitol	0.0	0.3	-0.06	0.1	0.2	-0.04
condition_era group during day -180 through 0 days relative to index: Benign neoplasm of respiratory system	0.1	0.2	-0.04	0.1	0.2	-0.04

Supplementary Figure 10: Preference score distributions, propensity score stratified (middle column) and matched (right column) covariate balance and negative control error distributions (bottom row) for ACE +combo vs CCB/THZ +combo in the SIDIAP data source.

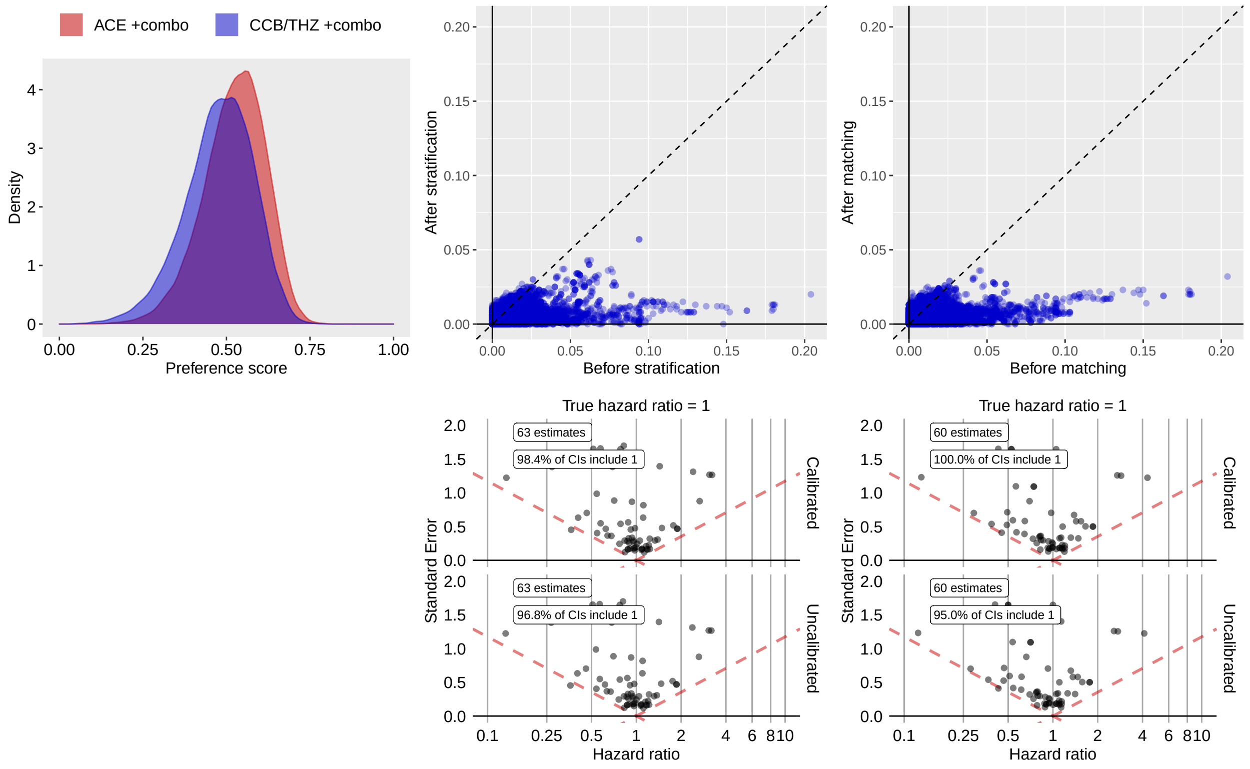

Supplementary Table 83: Most extreme patient characteristics balance diagnostics after stratification for ACE +combo vs CCB/THZ +combo in the SIDIAP data source.

	Before stratification			After stratification		
Characteristic (total count = 17019)	T (%)	C (%)	SDf	T (%)	C (%)	SDf
condition_era group during day -180 through 0 days relative to index: Transplanted kidney present	0.2	0.8	-0.09	0.2	0.6	-0.06
condition_era group during day -30 through 0 days relative to index: Transplanted kidney present	0.2	0.8	-0.09	0.2	0.6	-0.06
drug_era group during day -180 through 0 days relative to index: mycophenolate mofetil	0.1	0.3	-0.06	0.1	0.3	-0.04
drug_era group during day 0 through 0 days relative to index: mycophenolate mofetil	0.0	0.3	-0.06	0.1	0.2	-0.04
drug_era group during day -30 through 0 days relative to index: mycophenolate mofetil	0.1	0.3	-0.06	0.1	0.2	-0.04
drug_era group during day -180 through 0 days relative to index: paricalcitol	0.1	0.4	-0.06	0.1	0.3	-0.04
drug_era group during day -180 through 0 days relative to index: ANTI-PARATHYROID AGENTS	0.1	0.4	-0.06	0.1	0.3	-0.04
drug_era group during day -180 through 0 days relative to index: Other anti-parathyroid agents	0.1	0.4	-0.06	0.1	0.3	-0.04

(Continued)	T (%)	C (%)	SDf	T (%)	C (%)	SDf
condition_era group during day -180 through 0 days relative to index: Atherosclerosis of renal artery	0.0	0.2	-0.05	0.0	0.1	-0.04
condition_era group during day -30 through 0 days relative to index: Atherosclerosis of renal artery	0.0	0.2	-0.04	0.0	0.1	-0.04

Supplementary Table 84: Most extreme patient characteristics balance diagnostics after matching for ACE +combo vs CCB/THZ +combo in the SIDIAP data source.

	Before matching			After matching		
Characteristic (total count = 16913)	T (%)	C (%)	SDf	T (%)	C (%)	SDf
condition_era group during day -180 through 0 days relative to index: Atherosclerosis of renal artery	0.0	0.2	-0.05	0.0	0.1	-0.04
condition_era group during day -30 through 0 days relative to index: Atherosclerosis of renal artery	0.0	0.2	-0.04	0.0	0.1	-0.04
CHADS2VASc	267.1	294.6	-0.20	288.8	284.6	0.03
condition_era group during day -180 through 0 days relative to index: Nephrosclerosis	0.0	0.2	-0.04	0.1	0.2	-0.03
condition_era group during day -30 through 0 days relative to index: Nephrosclerosis	0.0	0.2	-0.04	0.1	0.2	-0.03
condition_era group during day -180 through 0 days relative to index: Renal vascular disorder	0.1	0.2	-0.04	0.1	0.2	-0.03
condition_era group during day -30 through 0 days relative to index: Renal vascular disorder	0.1	0.2	-0.04	0.1	0.2	-0.03
drug_era group during day -180 through 0 days relative to index: Cromolyn	0.1	0.0	0.03	0.1	0.0	0.03
drug_era group during day -180 through 0 days relative to index: Antiallergic agents, excl. corticosteroids	0.1	0.0	0.03	0.1	0.0	0.03
drug_era group during day 0 through 0 days relative to index: paricalcitol	0.1	0.3	-0.05	0.1	0.2	-0.03

Supplementary Figure 11: Preference score distributions, propensity score stratified (middle column) and matched (right column) covariate balance and negative control error distributions (bottom row) for ACE mono vs CCB mono in the SIDIAP data source.

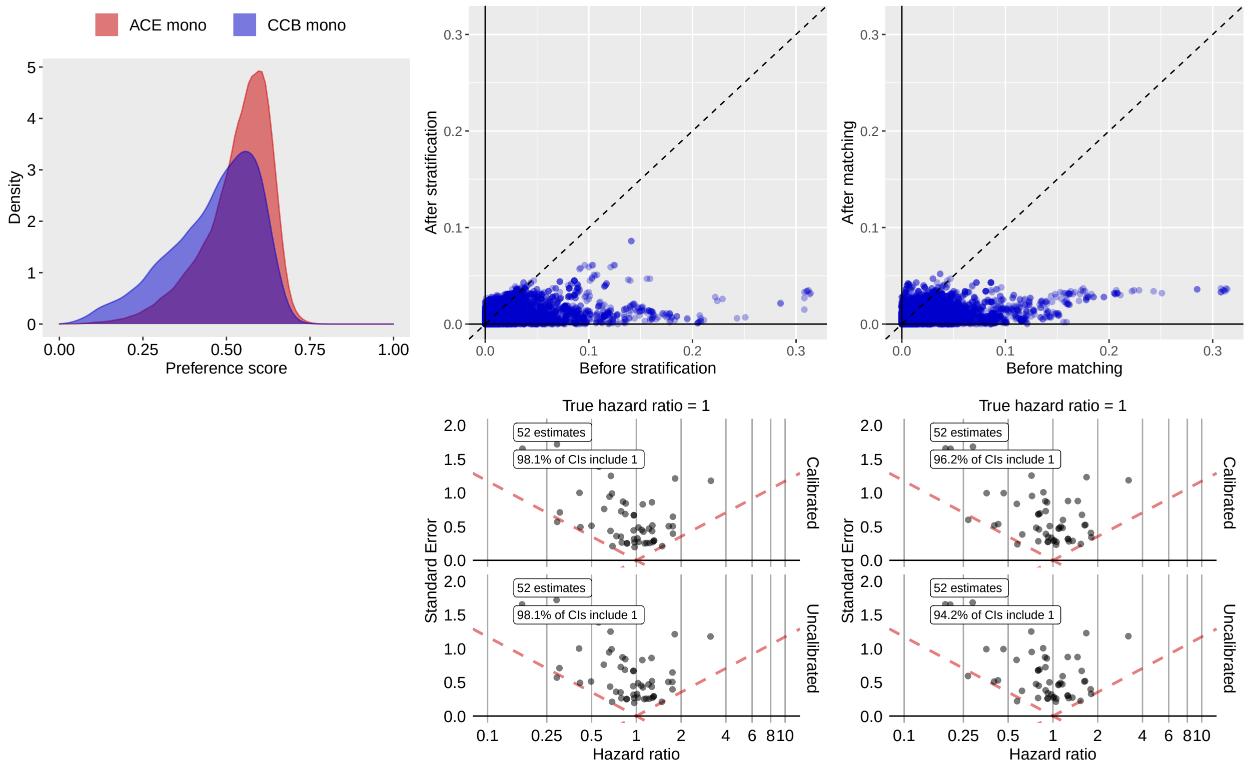

Supplementary Table 85: Most extreme patient characteristics balance diagnostics after stratification for ACE mono vs CCB mono in the SIDIAP data source.

	Before stratification			After stratification		
Characteristic (total count = 15793)	T (%)	C (%)	SDf	T (%)	C (%)	SDf
condition_era group during day -180 through 0 days relative to index: Transplanted kidney present	0.1	1.3	-0.14	0.2	0.7	-0.09
condition_era group during day -30 through 0 days relative to index: Transplanted kidney present	0.1	1.3	-0.14	0.2	0.7	-0.09
drug_era group during day -180 through 0 days relative to index: mycophenolate mofetil	0.0	0.6	-0.10	0.1	0.3	-0.06
drug_era group during day -180 through 0 days relative to index: Mycophenolic Acid	0.1	0.8	-0.10	0.1	0.4	-0.06
drug_era group during day -30 through 0 days relative to index: Mycophenolic Acid	0.1	0.7	-0.10	0.1	0.4	-0.06
drug_era group during day -180 through 0 days relative to index: Selective immunosuppressants	0.3	1.5	-0.12	0.4	0.9	-0.06
drug_era group during day -30 through 0 days relative to index: Selective immunosuppressants	0.3	1.4	-0.12	0.4	0.8	-0.06
drug_era group during day 0 through 0 days relative to index: Mycophenolic Acid	0.1	0.7	-0.10	0.1	0.4	-0.06

(Continued)	T (%)	C (%)	SDf	T (%)	C (%)	SDf
drug_era group during day 0 through 0 days relative to index: Selective immunosuppressants	0.3	1.4	-0.12	0.3	0.8	-0.06
drug_era group during day -30 through 0 days relative to index: mycophenolate mofetil	0.0	0.6	-0.09	0.1	0.3	-0.06

Supplementary Table 86: Most extreme patient characteristics balance diagnostics after matching for ACE mono vs CCB mono in the SIDIAP data source.

	Before matching			After matching		
Characteristic (total count = 15774)	T (%)	C (%)	SDf	T (%)	C (%)	SDf
drug_era group during day 0 through 0 days relative to index: Haloperidol	0.2	0.1	0.04	0.3	0.1	0.05
drug_era group during day 0 through 0 days relative to index: Butyrophenone derivatives	0.2	0.1	0.04	0.3	0.1	0.05
condition_era group during day -30 through 0 days relative to index: Pre-eclampsia	0.0	0.2	-0.05	0.0	0.2	-0.05
drug_era group during day -30 through 0 days relative to index: Haloperidol	0.3	0.1	0.03	0.4	0.1	0.05
drug_era group during day -30 through 0 days relative to index: Butyrophenone derivatives	0.3	0.1	0.03	0.4	0.1	0.05
condition_era group during day -180 through 0 days relative to index: Pre-eclampsia	0.0	0.2	-0.04	0.0	0.2	-0.04
drug_era group during day -180 through 0 days relative to index: paricalcitol	0.0	0.4	-0.07	0.1	0.3	-0.04
drug_era group during day 0 through 0 days relative to index: Trimethoprim	0.1	0.7	-0.09	0.3	0.6	-0.04
drug_era group during day 0 through 0 days relative to index: Sulfamethoxazole	0.1	0.7	-0.09	0.3	0.6	-0.04
drug_era group during day -180 through 0 days relative to index: silodosin	0.3	0.1	0.03	0.4	0.1	0.04

Supplementary Figure 12: Preference score distributions, propensity score stratified (middle column) and matched (right column) covariate balance and negative control error distributions (bottom row) for ACE +combo vs CCB +combo in the SIDIAP data source.

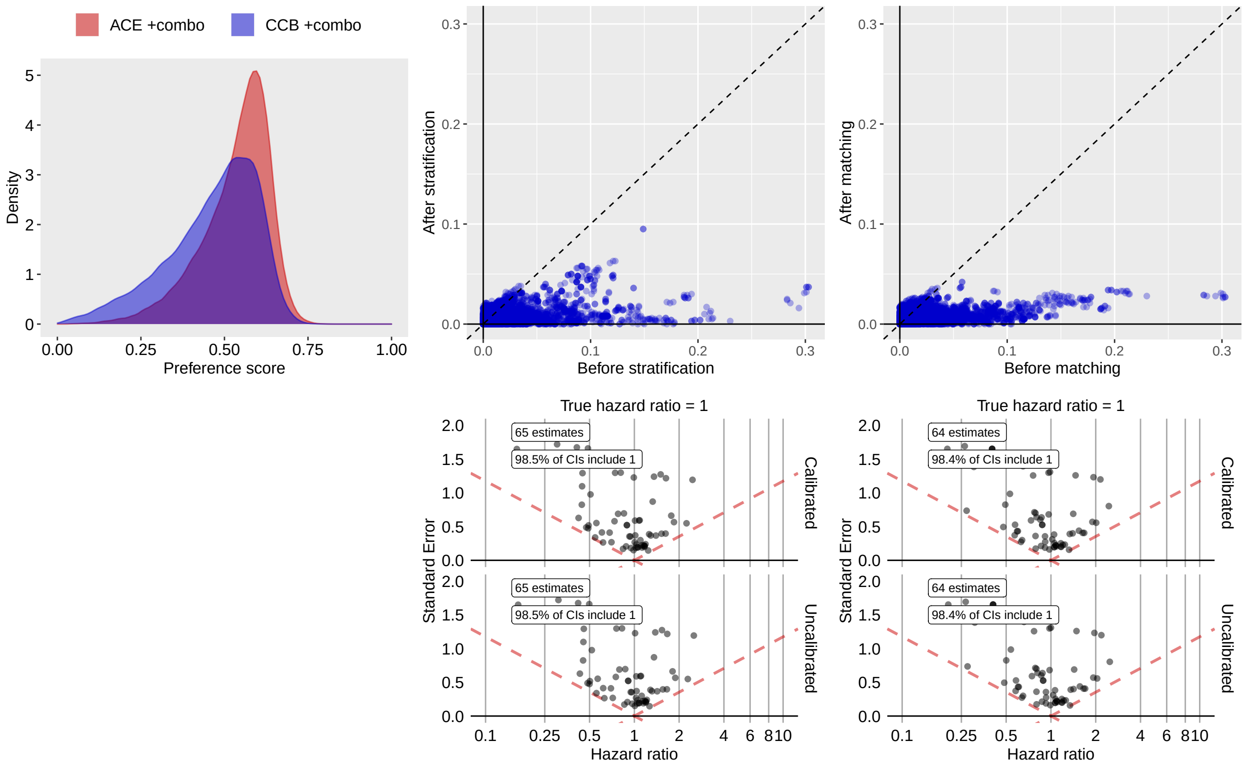

Supplementary Table 87: Most extreme patient characteristics balance diagnostics after stratification for ACE +combo vs CCB +combo in the SIDIAP data source.

	Before stratification			After stratification		
Characteristic (total count = 17207)	T (%)	C (%)	SDf	T (%)	C (%)	SDf
condition_era group during day -180 through 0 days relative to index: Transplanted kidney present	0.1	1.4	-0.15	0.2	0.8	-0.10
condition_era group during day -30 through 0 days relative to index: Transplanted kidney present	0.1	1.4	-0.15	0.2	0.8	-0.10
drug_era group during day -180 through 0 days relative to index: Selective immunosuppressants	0.3	1.5	-0.12	0.4	0.9	-0.06
drug_era group during day -30 through 0 days relative to index: Selective immunosuppressants	0.3	1.4	-0.12	0.4	0.8	-0.06
drug_era group during day 0 through 0 days relative to index: Selective immunosuppressants	0.3	1.4	-0.12	0.3	0.8	-0.06
drug_era group during day -180 through 0 days relative to index: paricalcitol	0.1	0.6	-0.09	0.1	0.3	-0.06
drug_era group during day -180 through 0 days relative to index: mycophenolate mofetil	0.1	0.6	-0.09	0.1	0.3	-0.06
drug_era group during day -180 through 0 days relative to index: ANTI-PARATHYROID AGENTS	0.1	0.6	-0.09	0.1	0.3	-0.06

(Continued)	T (%)	C (%)	SDf	T (%)	C (%)	SDf
drug_era group during day -180 through 0 days relative to index: Other anti-parathyroid agents	0.1	0.6	-0.09	0.1	0.3	-0.06
drug_era group during day -30 through 0 days relative to index: mycophenolate mofetil	0.0	0.5	-0.09	0.1	0.3	-0.06

Supplementary Table 88: Most extreme patient characteristics balance diagnostics after matching for ACE +combo vs CCB +combo in the SIDIAP data source.

	Before matching			After matching		
Characteristic (total count = 17189)	T (%)	C (%)	SDf	T (%)	C (%)	SDf
condition_era group during day -180 through 0 days relative to index: Atherosclerosis of renal artery	0.0	0.2	-0.06	0.0	0.2	-0.04
condition_era group during day -30 through 0 days relative to index: Atherosclerosis of renal artery	0.0	0.2	-0.06	0.0	0.2	-0.04
condition_era group during day -180 through 0 days relative to index: Renal vascular disorder	0.0	0.3	-0.06	0.1	0.2	-0.04
condition_era group during day -30 through 0 days relative to index: Renal vascular disorder	0.0	0.3	-0.06	0.1	0.2	-0.04
condition_era group during day -180 through 0 days relative to index: Pre-eclampsia	0.0	0.1	-0.04	0.0	0.1	-0.04
condition_era group during day -180 through 0 days relative to index: Nephrosclerosis	0.0	0.3	-0.06	0.1	0.2	-0.04
condition_era group during day -30 through 0 days relative to index: Nephrosclerosis	0.0	0.3	-0.06	0.1	0.2	-0.04
condition_era group during day -30 through 0 days relative to index: Pre-eclampsia	0.0	0.1	-0.03	0.0	0.1	-0.04
condition_era group during day -180 through 0 days relative to index: Pregnancy-induced hypertension	0.1	0.2	-0.03	0.1	0.2	-0.04
drug_era group during day -30 through 0 days relative to index: ANTITHROMBOTIC AGENTS	20.4	28.9	-0.20	29.4	27.9	0.03

Supplementary Figure 13: Preference score distributions, propensity score stratified (middle column) and matched (right column) covariate balance and negative control error distributions (bottom row) for ACE mono vs THZ mono in the SIDIAP data source.

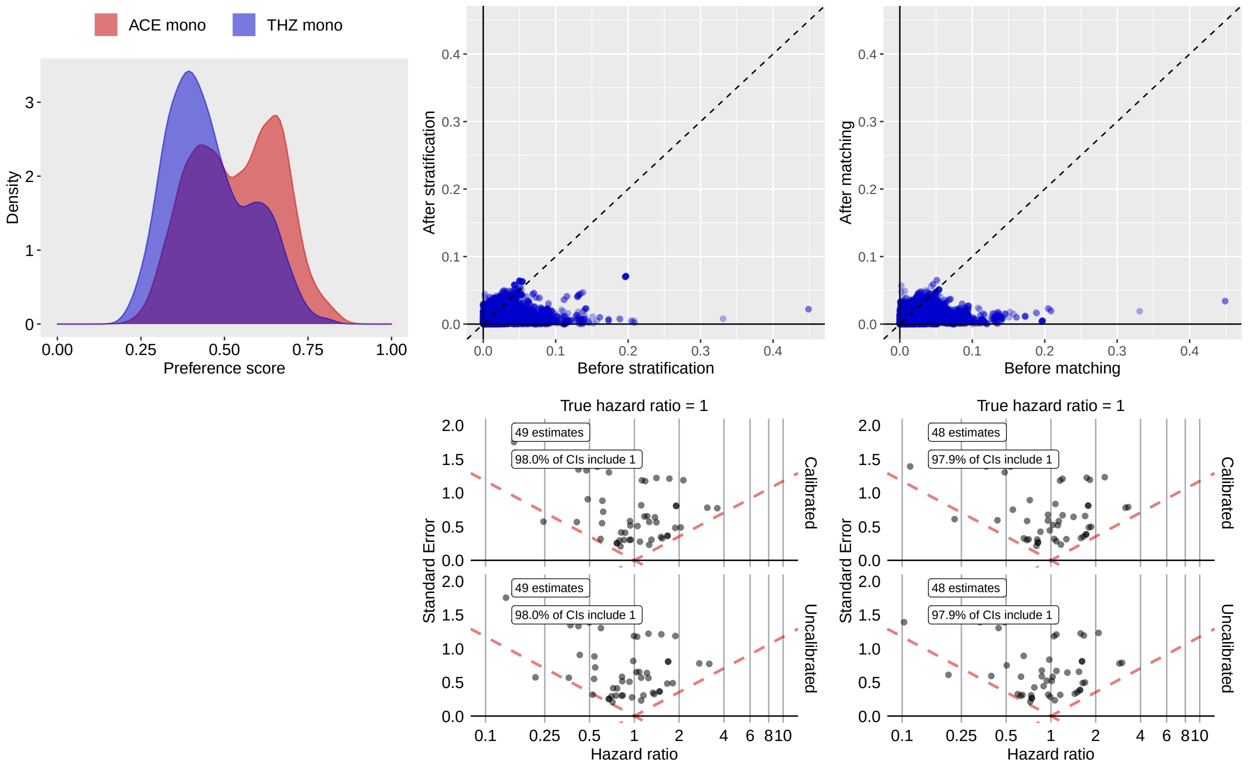

Supplementary Table 89: Most extreme patient characteristics balance diagnostics after stratification for ACE mono vs THZ mono in the SIDIAP data source.

	Before stratification			After stratification		
Characteristic (total count = 15689)	T (%)	C (%)	SDf	T (%)	C (%)	SDf
condition_era group during day -30 through 0 days relative to index: Gout	4.8	1.4	0.20	4.4	3.0	0.07
condition_era group during day -30 through 0 days relative to index: Blood urate abnormal	4.8	1.4	0.20	4.4	3.0	0.07
condition_era group during day -30 through 0 days relative to index: Blood urate raised	4.8	1.4	0.20	4.4	3.0	0.07
condition_era group during day -30 through 0 days relative to index: Increased uric acid level	4.8	1.4	0.20	4.4	3.0	0.07
condition_era group during day -180 through 0 days relative to index: Gout	4.8	1.4	0.20	4.4	3.1	0.07
condition_era group during day -180 through 0 days relative to index: Blood urate abnormal	4.8	1.4	0.20	4.4	3.1	0.07
condition_era group during day -180 through 0 days relative to index: Blood urate raised	4.8	1.4	0.20	4.4	3.1	0.07
condition_era group during day -180 through 0 days relative to index: Increased uric acid level	4.8	1.4	0.20	4.4	3.1	0.07

(Continued)	T (%)	C (%)	SDf	T (%)	C (%)	SDf
drug_era group during day 0 through 0 days relative to index: potassium citrate	0.0	0.2	-0.05	0.0	0.3	-0.06
drug_era group during day 0 through 0 days relative to index: Ascorbic Acid	0.0	0.2	-0.05	0.0	0.3	-0.06

Supplementary Table 90: Most extreme patient characteristics balance diagnostics after matching for ACE mono vs THZ mono in the SIDIAP data source.

	Before matching			After matching		
Characteristic (total count = 15346)	T (%)	C (%)	SDf	T (%)	C (%)	SDf
condition_era group during day -180 through 0 days relative to index: Hypo-osmolality and or hyponatremia	0.4	0.1	0.05	0.5	0.1	0.07
condition_era group during day -30 through 0 days relative to index: Hypo-osmolality and or hyponatremia	0.4	0.1	0.05	0.5	0.1	0.07
drug_era group during day -30 through 0 days relative to index: rizatriptan	0.2	-0.1	0.05	0.3	-0.1	0.06
drug_era group during day 0 through 0 days relative to index: rizatriptan	0.2	-0.1	0.05	0.3	-0.1	0.06
condition_era group during day -180 through 0 days relative to index: Hypertensive disorder	99.2	99.2	0.00	98.6	99.2	-0.06
drug_era group during day -180 through 0 days relative to index: rizatriptan	0.3	0.1	0.04	0.4	0.1	0.06
drug_era group during day -180 through 0 days relative to index: potassium citrate	0.0	0.3	-0.05	0.1	0.3	-0.05
drug_era group during day -180 through 0 days relative to index: Ascorbic Acid	0.0	0.3	-0.05	0.1	0.3	-0.05
drug_era group during day -180 through 0 days relative to index: ASCORBIC ACID (VITAMIN C), INCL. COMBINATIONS	0.0	0.3	-0.05	0.1	0.3	-0.05
drug_era group during day -180 through 0 days relative to index: Ascorbic acid (vitamin C), plain	0.0	0.3	-0.05	0.1	0.3	-0.05

Supplementary Figure 14: Preference score distributions, propensity score stratified (middle column) and matched (right column) covariate balance and negative control error distributions (bottom row) for ACE +combo vs THZ +combo in the SIDIAP data source.

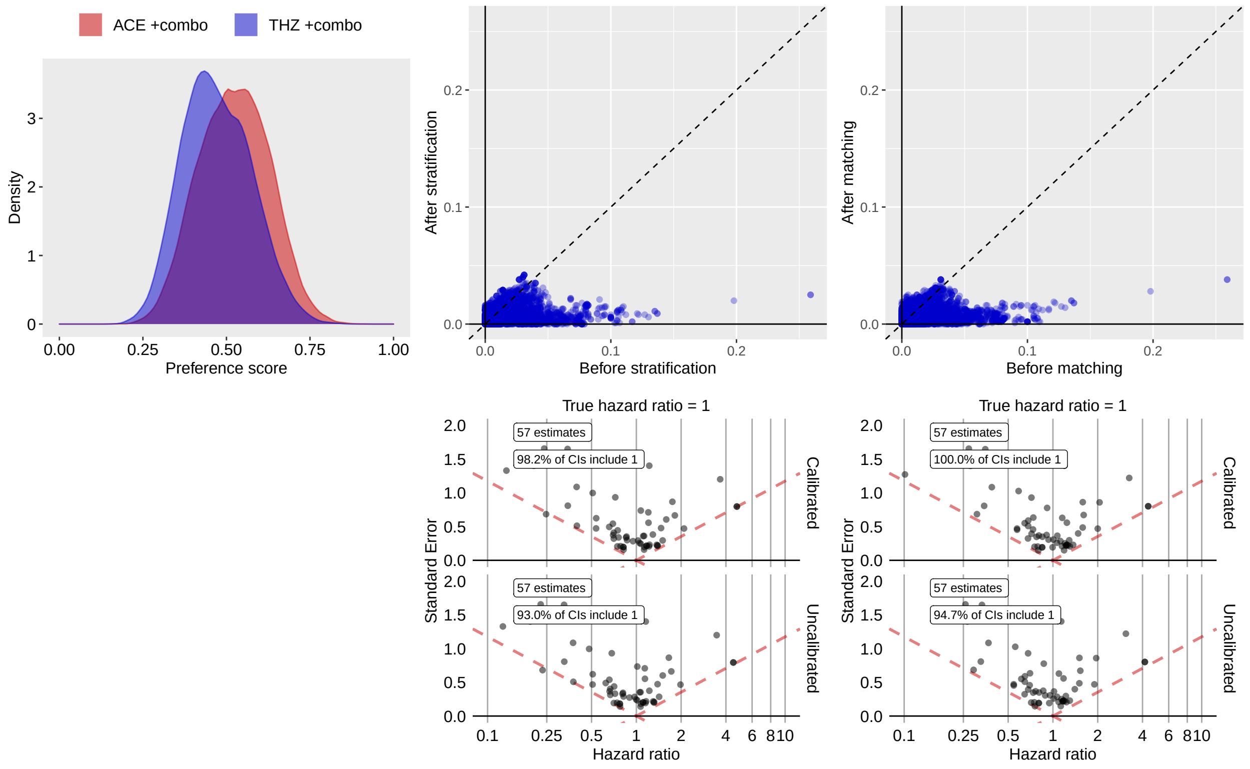

Supplementary Table 91: Most extreme patient characteristics balance diagnostics after stratification for ACE +combo vs THZ +combo in the SIDIAP data source.

	Before stratification			After stratification		
Characteristic (total count = 16868)	T (%)	C (%)	SDf	T (%)	C (%)	SDf
drug_era group during day 0 through 0 days relative to index: potassium citrate	0.1	0.2	-0.03	0.1	0.2	-0.04
drug_era group during day 0 through 0 days relative to index: Ascorbic Acid	0.1	0.2	-0.03	0.1	0.2	-0.04
drug_era group during day 0 through 0 days relative to index: ASCORBIC ACID (VITAMIN C), INCL. COMBINATIONS	0.1	0.2	-0.03	0.1	0.2	-0.04
drug_era group during day 0 through 0 days relative to index: Ascorbic acid (vitamin C), plain	0.1	0.2	-0.03	0.1	0.2	-0.04
drug_era group during day 0 through 0 days relative to index: POTASSIUM	0.1	0.2	-0.03	0.1	0.2	-0.04
drug_era group during day 0 through 0 days relative to index: Potassium	0.1	0.2	-0.03	0.1	0.2	-0.04
drug_era group during day -180 through 0 days relative to index: potassium citrate	0.1	0.3	-0.03	0.1	0.3	-0.04
drug_era group during day -180 through 0 days relative to index: Ascorbic Acid	0.1	0.3	-0.03	0.1	0.3	-0.04

(Continued)	T (%)	C (%)	SDf	T (%)	C (%)	SDf
drug_era group during day -180 through 0 days relative to index: ASCORBIC ACID (VITAMIN C), INCL. COMBINATIONS	0.1	0.3	-0.03	0.1	0.3	-0.04
drug_era group during day -180 through 0 days relative to index: Ascorbic acid (vitamin C), plain	0.1	0.3	-0.03	0.1	0.3	-0.04

Supplementary Table 92: Most extreme patient characteristics balance diagnostics after matching for ACE +combo vs THZ +combo in the SIDIAP data source.

	Before matching			After matching		
Characteristic (total count = 16834)	T (%)	C (%)	SDf	T (%)	C (%)	SDf
gender = MALE	54.6	41.7	0.26	40.1	41.9	-0.04
gender = FEMALE	45.4	58.3	-0.26	59.9	58.1	0.04
drug_era group during day 0 through 0 days relative to index: potassium citrate	0.1	0.2	-0.03	0.1	0.2	-0.04
drug_era group during day 0 through 0 days relative to index: Ascorbic Acid	0.1	0.2	-0.03	0.1	0.2	-0.04
drug_era group during day 0 through 0 days relative to index: ASCORBIC ACID (VITAMIN C), INCL. COMBINATIONS	0.1	0.2	-0.03	0.1	0.2	-0.04
drug_era group during day 0 through 0 days relative to index: Ascorbic acid (vitamin C), plain	0.1	0.2	-0.03	0.1	0.2	-0.04
drug_era group during day 0 through 0 days relative to index: POTASSIUM	0.1	0.2	-0.03	0.1	0.2	-0.04
drug_era group during day 0 through 0 days relative to index: Potassium	0.1	0.2	-0.03	0.1	0.2	-0.04
condition_era group during day -180 through 0 days relative to index: Atherosclerosis of renal artery	0.0	0.1	-0.03	0.0	0.1	-0.04
condition_era group during day -30 through 0 days relative to index: Atherosclerosis of renal artery	0.0	0.1	-0.03	0.0	0.1	-0.03

Supplementary Figure 15: Preference score distributions, propensity score stratified (middle column) and matched (right column) covariate balance and negative control error distributions (bottom row) for ARB mono vs CCB/THZ mono in the SIDIAP data source.

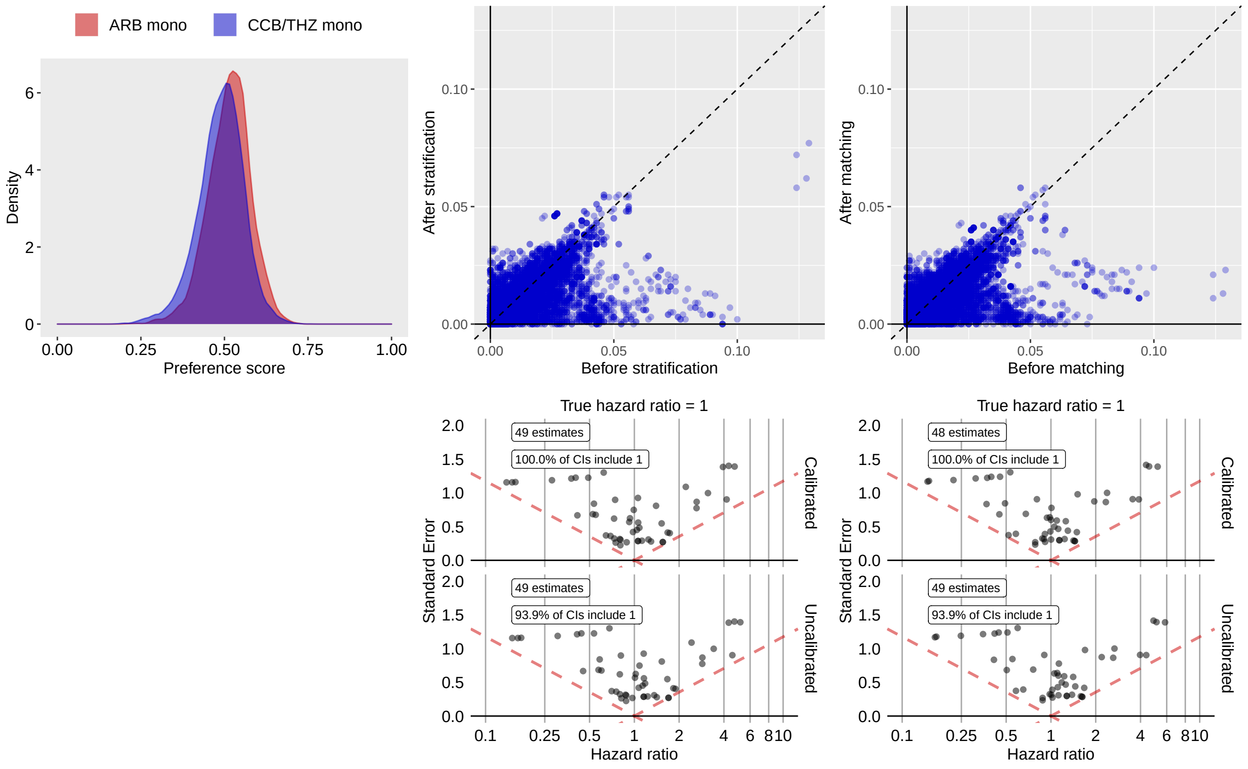

Supplementary Table 93: Most extreme patient characteristics balance diagnostics after stratification for ARB mono vs CCB/THZ mono in the SIDIAP data source.

	Before stratification			After stratification		
Characteristic (total count = 14032)	T (%)	C (%)	SDf	T (%)	C (%)	SDf
condition_era group during day -180 through 0 days relative to index: Hyperkalemia	0.7	2.2	-0.13	1.0	1.9	-0.08
condition_era group during day -30 through 0 days relative to index: Hyperkalemia	0.7	2.1	-0.12	1.0	1.8	-0.07
condition_era group during day -180 through 0 days relative to index: Potassium disorder	1.2	3.1	-0.13	1.8	2.7	-0.06
condition_era group during day -30 through 0 days relative to index: Potassium disorder	1.2	2.9	-0.12	1.7	2.6	-0.06
drug_era group during day -180 through 0 days relative to index: Haloperidol	0.5	0.2	0.05	0.5	0.2	0.06
condition_era group during day -180 through 0 days relative to index: Moderate major depression, single episode	-0.1	0.3	-0.06	-0.1	0.3	-0.06
drug_era group during day -180 through 0 days relative to index: Butyrophenone derivatives	0.5	0.2	0.05	0.5	0.2	0.06
condition_era group during day -180 through 0 days relative to index: Spontaneous intracranial hemorrhage	0.1	0.3	-0.05	0.1	0.4	-0.06

(Continued)	T (%)	C (%)	SDf	T (%)	C (%)	SDf
drug_era group during day -30 through 0 days relative to index: Tobramycin	0.2	0.6	-0.06	0.2	0.5	-0.05
drug_era group during day -180 through 0 days relative to index: rizatriptan	0.4	0.1	0.05	0.4	0.1	0.05

Supplementary Table 94: Most extreme patient characteristics balance diagnostics after matching for ARB mono vs CCB/THZ mono in the SIDIAP data source.

	Before matching			After matching		
Characteristic (total count = 14029)	T (%)	C (%)	SDf	T (%)	C (%)	SDf
drug_era group during day -180 through 0 days relative to index: Haloperidol	0.5	0.2	0.05	0.5	0.2	0.06
condition_era group during day -180 through 0 days relative to index: Moderate major depression, single episode	-0.1	0.3	-0.06	-0.1	0.3	-0.06
drug_era group during day -180 through 0 days relative to index: Butyrophenone derivatives	0.5	0.2	0.05	0.5	0.2	0.06
condition_era group during day -30 through 0 days relative to index: Moderate major depression, single episode	-0.1	0.3	-0.06	-0.1	0.3	-0.06
condition_era group during day -180 through 0 days relative to index: Spontaneous intracranial hemorrhage	0.1	0.3	-0.05	0.1	0.4	-0.06
condition_era group during day -30 through 0 days relative to index: Spontaneous intracranial hemorrhage	0.1	0.3	-0.05	0.1	0.4	-0.05
drug_era group during day -30 through 0 days relative to index: Tobramycin	0.2	0.6	-0.06	0.2	0.5	-0.05
drug_era group during day -180 through 0 days relative to index: rizatriptan	0.4	0.1	0.05	0.4	0.1	0.05
condition_era group during day -180 through 0 days relative to index: Type B viral hepatitis	0.3	0.6	-0.05	0.3	0.6	-0.05
condition_era group during day -30 through 0 days relative to index: Type B viral hepatitis	0.3	0.6	-0.05	0.3	0.6	-0.05

Supplementary Figure 16: Preference score distributions, propensity score stratified (middle column) and matched (right column) covariate balance and negative control error distributions (bottom row) for ARB +combo vs CCB/THZ +combo in the SIDIAP data source.

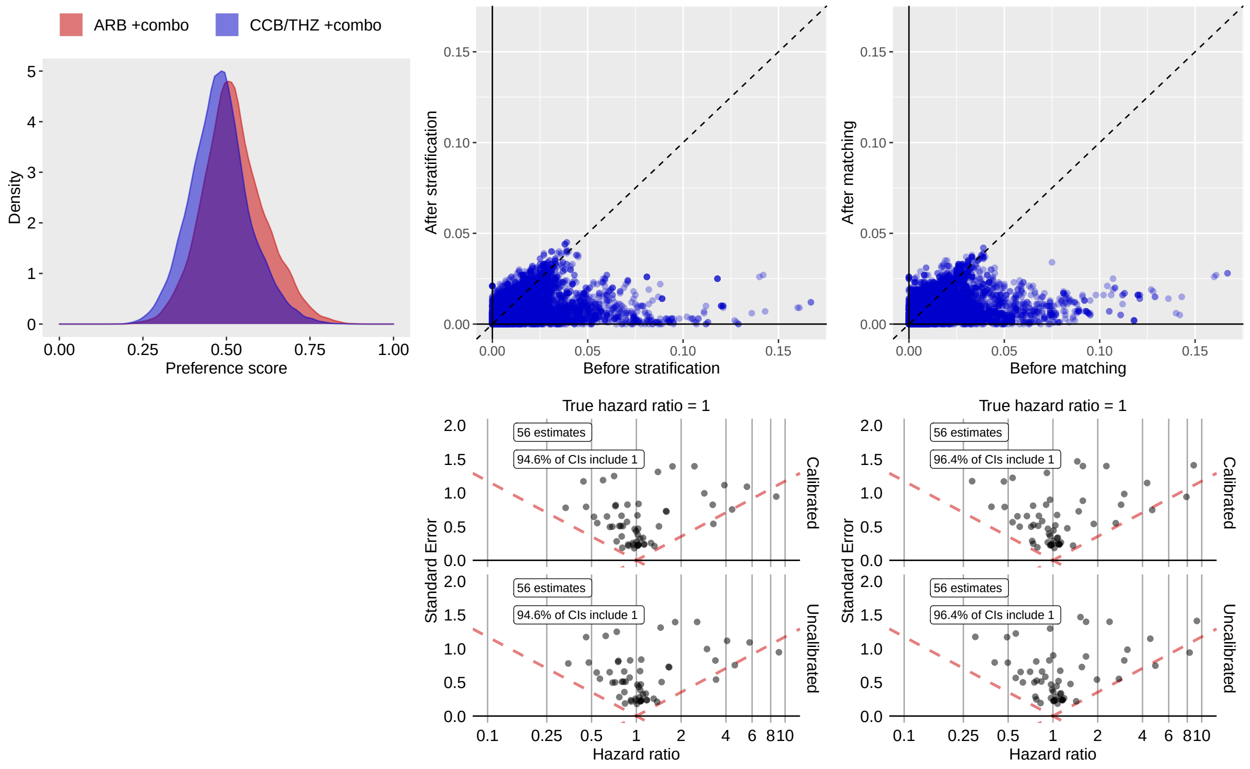

Supplementary Table 95: Most extreme patient characteristics balance diagnostics after stratification for ARB +combo vs CCB/THZ +combo in the SIDIAP data source.

	Before stratification			After stratification		
Characteristic (total count = 15991)	T (%)	C (%)	SDf	T (%)	C (%)	SDf
condition_era group during day -180 through 0 days relative to index: Disorder due to and following drug poisoning	0.1	0.0	0.04	0.1	0.0	0.04
condition_era group during day -30 through 0 days relative to index: Disorder due to and following drug poisoning	0.1	0.0	0.04	0.1	0.0	0.04
drug_era group during day -30 through 0 days relative to index: Tobramycin	0.3	0.5	-0.04	0.2	0.5	-0.04
condition_era group during day -180 through 0 days relative to index: Disorder due to and following poisoning	0.1	0.0	0.04	0.1	0.0	0.04
condition_era group during day -30 through 0 days relative to index: Disorder due to and following poisoning	0.1	0.0	0.04	0.1	0.0	0.04
condition_era group during day -180 through 0 days relative to index: Moderate major depression, single episode	0.1	0.2	-0.04	0.1	0.2	-0.04
condition_era group during day -180 through 0 days relative to index: Diffuse cervicobrachial syndrome	0.2	0.4	-0.04	0.1	0.3	-0.04
condition_era group during day -180 through 0 days relative to index: Pityriasis	0.2	0.1	0.03	0.2	0.1	0.04

(Continued)	T (%)	C (%)	SDf	T (%)	C (%)	SDf
condition_era group during day -30 through 0 days relative to index: Moderate major depression, single episode	0.1	0.2	-0.04	0.1	0.2	-0.04
condition_era group during day -180 through 0 days relative to index: Type B viral hepatitis	0.4	0.6	-0.04	0.3	0.6	-0.04

Supplementary Table 96: Most extreme patient characteristics balance diagnostics after matching for ARB +combo vs CCB/THZ +combo in the SIDIAP data source.

	Before matching			After matching		
Characteristic (total count = 15981)	T (%)	C (%)	SDf	T (%)	C (%)	SDf
condition_era group during day -180 through 0 days relative to index: Moderate major depression, single episode	0.1	0.2	-0.04	0.1	0.2	-0.04
condition_era group during day -30 through 0 days relative to index: Moderate major depression, single episode	0.1	0.2	-0.04	0.1	0.2	-0.04
condition_era group during day -180 through 0 days relative to index: Disorder due to and following drug poisoning	0.1	0.0	0.04	0.1	0.0	0.04
condition_era group during day -30 through 0 days relative to index: Disorder due to and following drug poisoning	0.1	0.0	0.04	0.1	0.0	0.04
condition_era group during day -180 through 0 days relative to index: Disorder due to and following poisoning	0.1	0.0	0.04	0.1	0.0	0.04
condition_era group during day -30 through 0 days relative to index: Disorder due to and following poisoning	0.1	0.0	0.04	0.1	0.0	0.04
condition_era group during day -30 through 0 days relative to index: Malignant epithelial neoplasm	-0.1	0.1	-0.03	-0.1	0.1	-0.04
condition_era group during day -180 through 0 days relative to index: Diffuse cervicobrachial syndrome	0.2	0.4	-0.04	0.2	0.3	-0.04
condition_era group during day -30 through 0 days relative to index: Diffuse cervicobrachial syndrome	0.2	0.3	-0.04	0.2	0.3	-0.04
condition_era group during day -30 through 0 days relative to index: Primary malignant neoplasm of liver	-0.1	0.1	-0.03	-0.1	0.1	-0.04

Supplementary Figure 17: Preference score distributions, propensity score stratified (middle column) and matched (right column) covariate balance and negative control error distributions (bottom row) for ARB mono vs CCB mono in the SIDIAP data source.

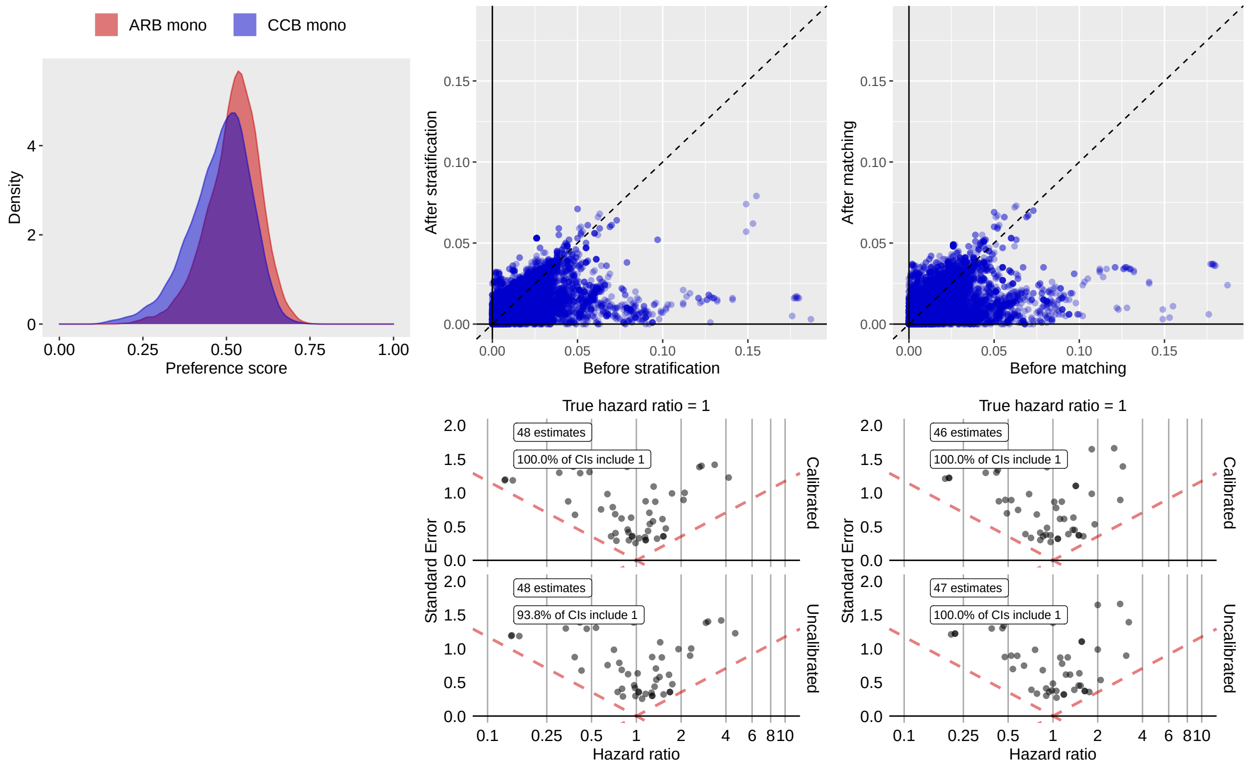

Supplementary Table 97: Most extreme patient characteristics balance diagnostics after stratification for ARB mono vs CCB mono in the SIDIAP data source.

Characteristic (total count = 13437)	Before stratification			After stratification		
	T (%)	C (%)	SDf	T (%)	C (%)	SDf
condition_era group during day -180 through 0 days relative to index: Hyperkalemia	0.7	2.6	-0.15	1.0	2.0	-0.08
condition_era group during day -30 through 0 days relative to index: Hyperkalemia	0.7	2.5	-0.15	1.0	1.9	-0.07
drug_era group during day -180 through 0 days relative to index: Haloperidol	0.5	0.2	0.05	0.6	0.2	0.07
drug_era group during day -180 through 0 days relative to index: Butyrophenone derivatives	0.5	0.2	0.05	0.6	0.2	0.07
condition_era group during day -180 through 0 days relative to index: Moderate major depression, single episode	-0.1	0.3	-0.06	-0.1	0.3	-0.07
condition_era group during day -30 through 0 days relative to index: Moderate major depression, single episode	-0.1	0.3	-0.06	-0.1	0.3	-0.07
drug_era group during day -180 through 0 days relative to index: Lithium Carbonate	0.1	0.6	-0.07	0.2	0.5	-0.06
drug_era group during day -180 through 0 days relative to index: Lithium	0.1	0.6	-0.07	0.2	0.5	-0.06

(Continued)	T (%)	C (%)	SDf	T (%)	C (%)	SDf
condition_era group during day -180 through 0 days relative to index: Potassium disorder	1.2	3.5	-0.15	1.9	2.8	-0.06
drug_era group during day -30 through 0 days relative to index: Lithium Carbonate	0.1	0.5	-0.07	0.1	0.5	-0.06

Supplementary Table 98: Most extreme patient characteristics balance diagnostics after matching for ARB mono vs CCB mono in the SIDIAP data source.

	Before matching			After matching		
Characteristic (total count = 13387)	T (%)	C (%)	SDf	T (%)	C (%)	SDf
condition_era group during day -180 through 0 days relative to index: Moderate major depression, single episode	-0.1	0.3	-0.06	-0.1	0.4	-0.07
condition_era group during day -30 through 0 days relative to index: Moderate major depression, single episode	-0.1	0.3	-0.06	-0.1	0.3	-0.07
drug_era group during day -180 through 0 days relative to index: Lithium Carbonate	0.1	0.6	-0.07	0.1	0.5	-0.07
drug_era group during day -180 through 0 days relative to index: Lithium	0.1	0.6	-0.07	0.1	0.5	-0.07
drug_era group during day -180 through 0 days relative to index: Haloperidol	0.5	0.2	0.05	0.5	0.1	0.07
drug_era group during day -180 through 0 days relative to index: Butyrophenone derivatives	0.5	0.2	0.05	0.5	0.1	0.07
condition_era group during day -180 through 0 days relative to index: Finding related to pregnancy	0.3	0.7	-0.05	0.3	0.8	-0.07
drug_era group during day -30 through 0 days relative to index: Lithium Carbonate	0.1	0.5	-0.07	0.1	0.5	-0.07
drug_era group during day -30 through 0 days relative to index: Lithium	0.1	0.5	-0.07	0.1	0.5	-0.07
condition_era group during day -30 through 0 days relative to index: Finding related to pregnancy	0.3	0.6	-0.05	0.3	0.8	-0.07

Supplementary Figure 18: Preference score distributions, propensity score stratified (middle column) and matched (right column) covariate balance and negative control error distributions (bottom row) for ARB +combo vs CCB +combo in the SIDIAP data source.

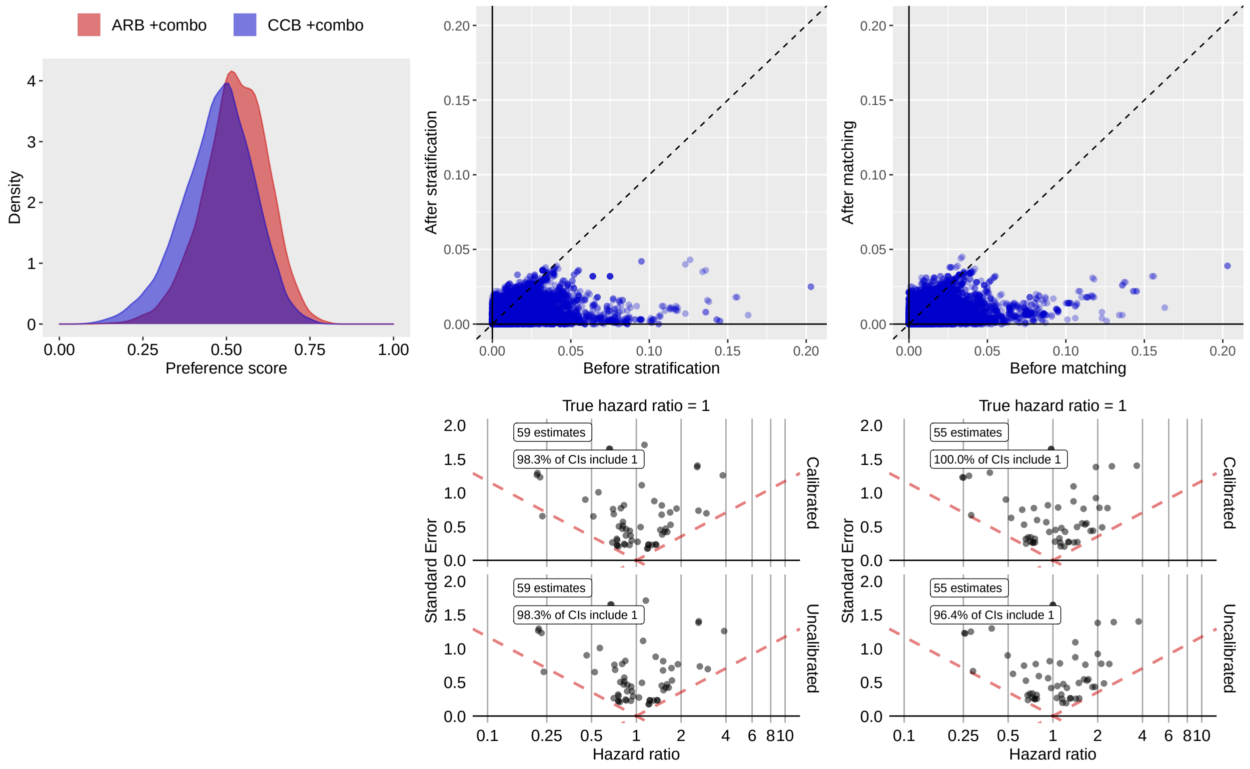

Supplementary Table 99: Most extreme patient characteristics balance diagnostics after stratification for ARB +combo vs CCB +combo in the SIDIAP data source.

	Before stratification			After stratification		
Characteristic (total count = 15238)	T (%)	C (%)	SDf	T (%)	C (%)	SDf
condition_era group during day -180 through 0 days relative to index: Hyperkalemia	0.8	2.4	-0.13	1.2	1.8	-0.04
condition_era group during day -180 through 0 days relative to index: Transplanted kidney present	0.4	1.3	-0.10	0.6	1.0	-0.04
condition_era group during day -30 through 0 days relative to index: Transplanted kidney present	0.4	1.3	-0.10	0.6	1.0	-0.04
condition_era group during day -30 through 0 days relative to index: Hyperkalemia	0.8	2.3	-0.12	1.2	1.7	-0.04
condition_era group during day -180 through 0 days relative to index: Systemic sclerosis	0.1	0.3	-0.04	0.1	0.3	-0.04
condition_era group during day -180 through 0 days relative to index: Moderate major depression, single episode	0.1	0.2	-0.03	0.1	0.2	-0.04
condition_era group during day -180 through 0 days relative to index: Autoimmune connective tissue disorder	0.1	0.3	-0.04	0.1	0.3	-0.04
condition_era group during day -30 through 0 days relative to index: Systemic sclerosis	0.1	0.3	-0.04	0.1	0.3	-0.04

(Continued)	T (%)	C (%)	SDf	T (%)	C (%)	SDf
condition_era group during day -180 through 0 days relative to index: Complication of pregnancy, childbirth and/or the puerperium	0.1	0.3	-0.04	0.1	0.3	-0.04
condition_era group during day -30 through 0 days relative to index: Moderate major depression, single episode	0.1	0.2	-0.03	0.1	0.2	-0.04

Supplementary Table 100: Most extreme patient characteristics balance diagnostics after matching for ARB +combo vs CCB +combo in the SIDIAP data source.

	Before matching			After matching		
Characteristic (total count = 15045)	T (%)	C (%)	SDf	T (%)	C (%)	SDf
condition_era group during day -180 through 0 days relative to index: Moderate major depression, single episode	0.1	0.2	-0.03	0.1	0.3	-0.04
condition_era group during day -30 through 0 days relative to index: Moderate major depression, single episode	0.1	0.2	-0.03	0.1	0.3	-0.04
gender = MALE	47.4	57.5	-0.20	53.1	51.2	0.04
gender = FEMALE	52.6	42.5	0.20	46.9	48.8	-0.04
condition_era group during day -180 through 0 days relative to index: Diffuse cervicobrachial syndrome	0.2	0.3	-0.03	0.1	0.3	-0.04
condition_era group during day -30 through 0 days relative to index: Diffuse cervicobrachial syndrome	0.2	0.3	-0.03	0.1	0.3	-0.04
condition_era group during day -180 through 0 days relative to index: Moderate depression	0.2	0.3	-0.03	0.1	0.3	-0.04
condition_era group during day -30 through 0 days relative to index: Moderate depression	0.2	0.3	-0.03	0.1	0.3	-0.04
condition_era group during day -180 through 0 days relative to index: Moderate major depression	0.2	0.3	-0.03	0.1	0.3	-0.04
condition_era group during day -30 through 0 days relative to index: Moderate major depression	0.2	0.3	-0.03	0.1	0.3	-0.04

Supplementary Figure 19: Preference score distributions, propensity score stratified (middle column) and matched (right column) covariate balance and negative control error distributions (bottom row) for ARB mono vs THZ mono in the SIDIAP data source.

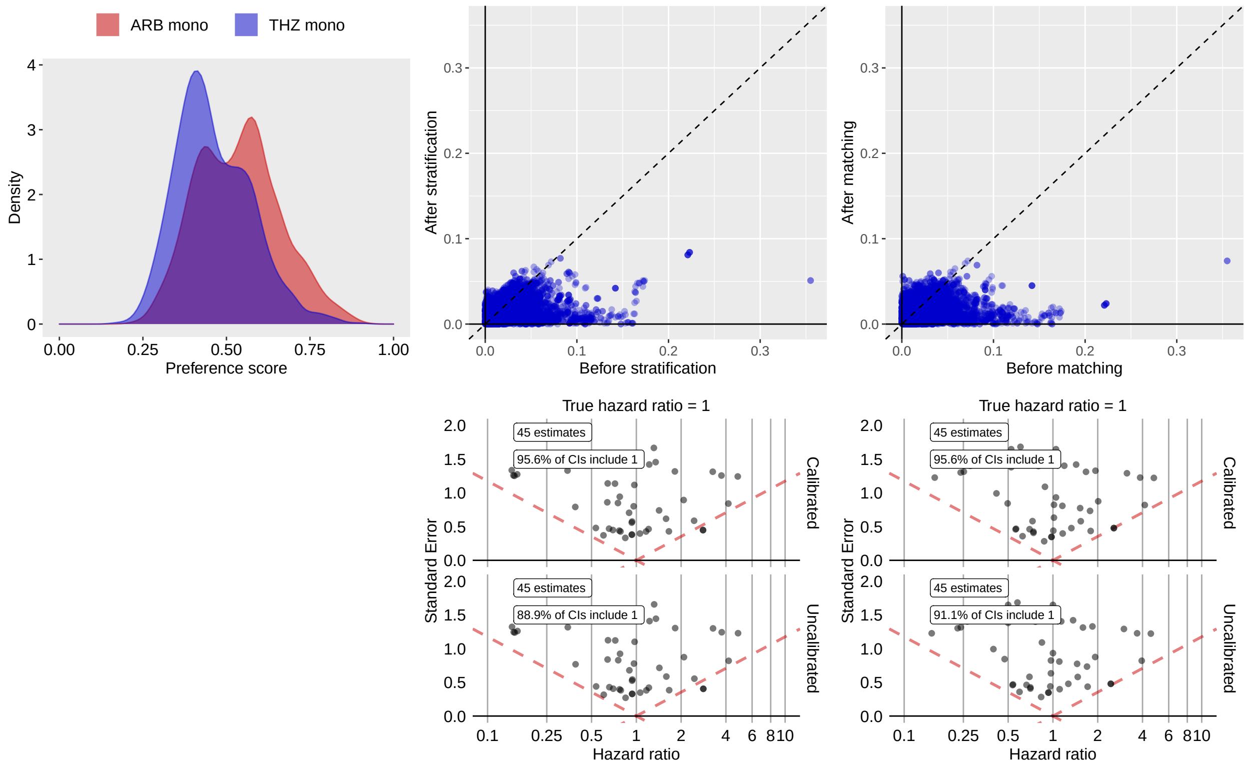

Supplementary Table 101: Most extreme patient characteristics balance diagnostics after stratification for ARB mono vs THZ mono in the SIDIAP data source.

	Before stratification			After stratification		
Characteristic (total count = 12815)	T (%)	C (%)	SDf	T (%)	C (%)	SDf
condition_era group during day -30 through 0 days relative to index: Gout	5.4	1.4	0.22	4.0	2.5	0.08
condition_era group during day -30 through 0 days relative to index: Blood urate abnormal	5.4	1.4	0.22	4.0	2.5	0.08
condition_era group during day -30 through 0 days relative to index: Blood urate raised	5.4	1.4	0.22	4.0	2.5	0.08
condition_era group during day -30 through 0 days relative to index: Increased uric acid level	5.4	1.4	0.22	4.0	2.5	0.08
condition_era group during day -180 through 0 days relative to index: Gout	5.4	1.4	0.22	4.0	2.6	0.08
condition_era group during day -180 through 0 days relative to index: Blood urate abnormal	5.4	1.4	0.22	4.0	2.6	0.08
condition_era group during day -180 through 0 days relative to index: Blood urate raised	5.4	1.4	0.22	4.0	2.6	0.08
condition_era group during day -180 through 0 days relative to index: Increased uric acid level	5.4	1.4	0.22	4.0	2.6	0.08

(Continued)	T (%)	C (%)	SDf	T (%)	C (%)	SDf
condition_era group during day -180 through 0 days relative to index: Multiple congenital cysts of kidney	0.6	0.1	0.08	0.6	0.1	0.08
condition_era group during day -30 through 0 days relative to index: Multiple congenital cysts of kidney	0.6	0.1	0.08	0.6	0.1	0.08

Supplementary Table 102: Most extreme patient characteristics balance diagnostics after matching for ARB mono vs THZ mono in the SIDIAP data source.

	Before matching			After matching		
Characteristic (total count = 12634)	T (%)	C (%)	SDf	T (%)	C (%)	SDf
gender = MALE	49.5	32.4	0.35	33.6	37.1	-0.07
gender = FEMALE	50.5	67.6	-0.35	66.4	62.9	0.07
condition_era group during day -180 through 0 days relative to index: Hypo-osmolality and or hyponatremia	0.5	0.1	0.07	0.6	0.1	0.07
condition_era group during day -30 through 0 days relative to index: Hypo-osmolality and or hyponatremia	0.5	0.1	0.07	0.6	0.1	0.07
condition_era group during day -180 through 0 days relative to index: Multiple congenital cysts of kidney	0.6	0.1	0.08	0.5	0.1	0.07
condition_era group during day -30 through 0 days relative to index: Multiple congenital cysts of kidney	0.6	0.1	0.08	0.5	0.1	0.07
drug_era group during day -180 through 0 days relative to index: Other psychostimulants and nootropics	0.3	-0.1	0.06	0.3	-0.1	0.07
drug_era group during day -180 through 0 days relative to index: rizatriptan	0.4	0.1	0.05	0.4	0.1	0.06
drug_era group during day -30 through 0 days relative to index: rizatriptan	0.3	-0.1	0.06	0.3	-0.1	0.06
drug_era group during day -30 through 0 days relative to index: ANTIMIGRAINE PREPARATIONS	0.8	0.5	0.04	1.0	0.5	0.06

Supplementary Figure 20: Preference score distributions, propensity score stratified (middle column) and matched (right column) covariate balance and negative control error distributions (bottom row) for ARB +combo vs THZ +combo in the SIDIAP data source.

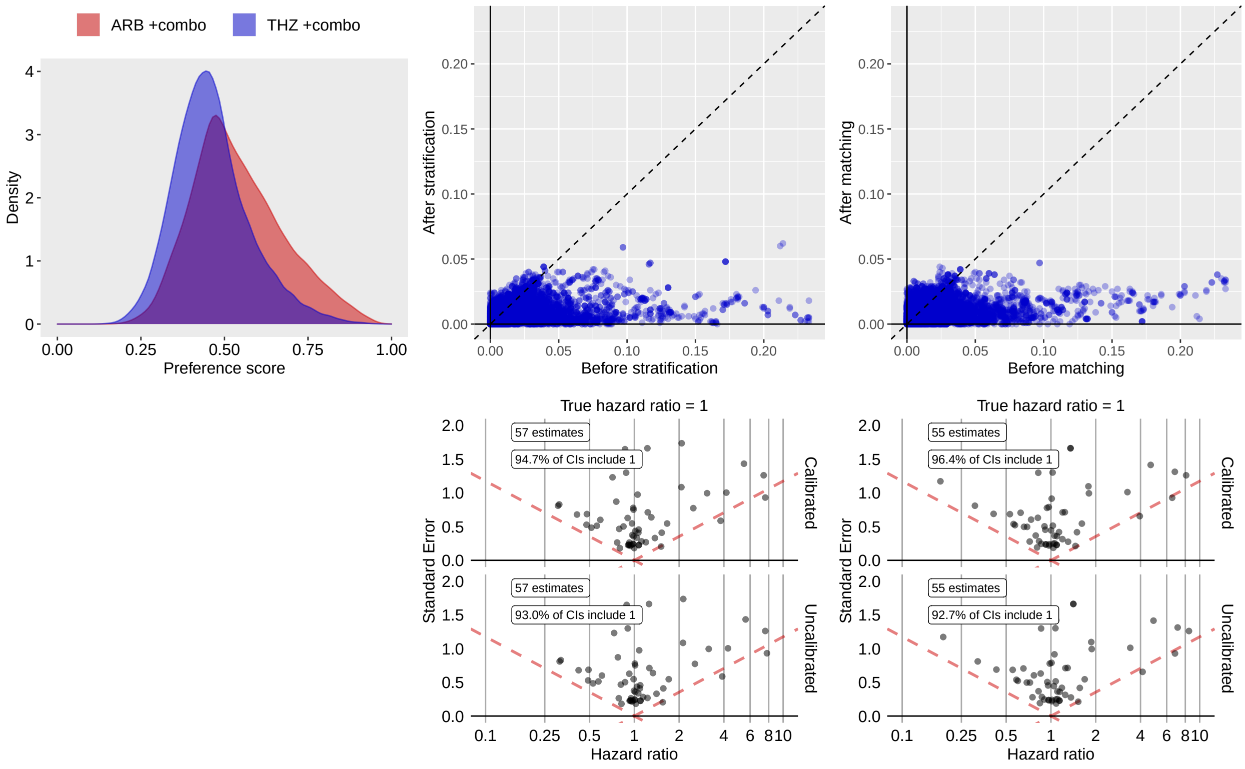

Supplementary Table 103: Most extreme patient characteristics balance diagnostics after stratification for ARB +combo vs THZ +combo in the SIDIAP data source.

Characteristic (total count = 15434)	Before stratification			After stratification		
	T (%)	C (%)	SDf	T (%)	C (%)	SDf
condition_era group during day -180 through 0 days relative to index: Heart failure	5.7	1.7	0.21	3.5	2.4	0.06
condition_era group during day -30 through 0 days relative to index: Heart failure	5.7	1.7	0.21	3.4	2.4	0.06
condition_era group during day -180 through 0 days relative to index: Transplanted kidney present	0.6	0.1	0.10	0.4	0.1	0.06
condition_era group during day -30 through 0 days relative to index: Transplanted kidney present	0.6	0.1	0.10	0.4	0.1	0.06
condition_era group during day -180 through 0 days relative to index: Abnormal cardiovascular function	3.9	1.2	0.17	2.3	1.7	0.05
condition_era group during day -30 through 0 days relative to index: Abnormal cardiovascular function	3.9	1.2	0.17	2.3	1.7	0.05
condition_era group during day -180 through 0 days relative to index: Congestive heart failure	3.9	1.2	0.17	2.3	1.7	0.05
condition_era group during day -30 through 0 days relative to index: Congestive heart failure	3.9	1.2	0.17	2.3	1.7	0.05

(Continued)	T (%)	C (%)	SDf	T (%)	C (%)	SDf
condition_era group during day -180 through 0 days relative to index: Systolic dysfunction	1.4	0.3	0.12	0.8	0.5	0.05
condition_era group during day -180 through 0 days relative to index: Systolic heart failure	1.4	0.3	0.12	0.8	0.5	0.05

Supplementary Table 104: Most extreme patient characteristics balance diagnostics after matching for ARB +combo vs THZ +combo in the SIDIAP data source.

	Before matching			After matching		
Characteristic (total count = 15350)	T (%)	C (%)	SDf	T (%)	C (%)	SDf
condition_era group during day -180 through 0 days relative to index: Transplanted kidney present	0.6	0.1	0.10	0.4	0.1	0.05
condition_era group during day -30 through 0 days relative to index: Transplanted kidney present	0.6	0.1	0.10	0.4	0.1	0.05
drug_era group during day -180 through 0 days relative to index: donepezil	0.1	0.2	-0.02	0.1	0.2	-0.04
drug_era group during day 0 through 0 days relative to index: donepezil	0.1	0.2	-0.03	0.1	0.2	-0.04
condition_era group during day -180 through 0 days relative to index: Disorder due to and following poisoning	0.1	0.0	0.04	0.1	0.0	0.04
condition_era group during day -30 through 0 days relative to index: Disorder due to and following poisoning	0.1	0.0	0.04	0.1	0.0	0.04
condition_era group during day -180 through 0 days relative to index: Disorder due to and following drug poisoning	0.1	0.0	0.04	0.1	0.0	0.04
condition_era group during day -30 through 0 days relative to index: Disorder due to and following drug poisoning	0.1	0.0	0.04	0.1	0.0	0.04
condition_era group during day -180 through 0 days relative to index: Diffuse cervicobrachial syndrome	0.1	0.4	-0.05	0.1	0.3	-0.04
drug_era group during day -30 through 0 days relative to index: donepezil	0.1	0.2	-0.02	0.1	0.2	-0.04

VA-OMOP

Supplementary Figure 21: Preference score distributions, propensity score stratified (middle column) and matched (right column) covariate balance and negative control error distributions (bottom row) for ACE/ARB mono vs CCB/THZ mono in the VA-OMOP data source.

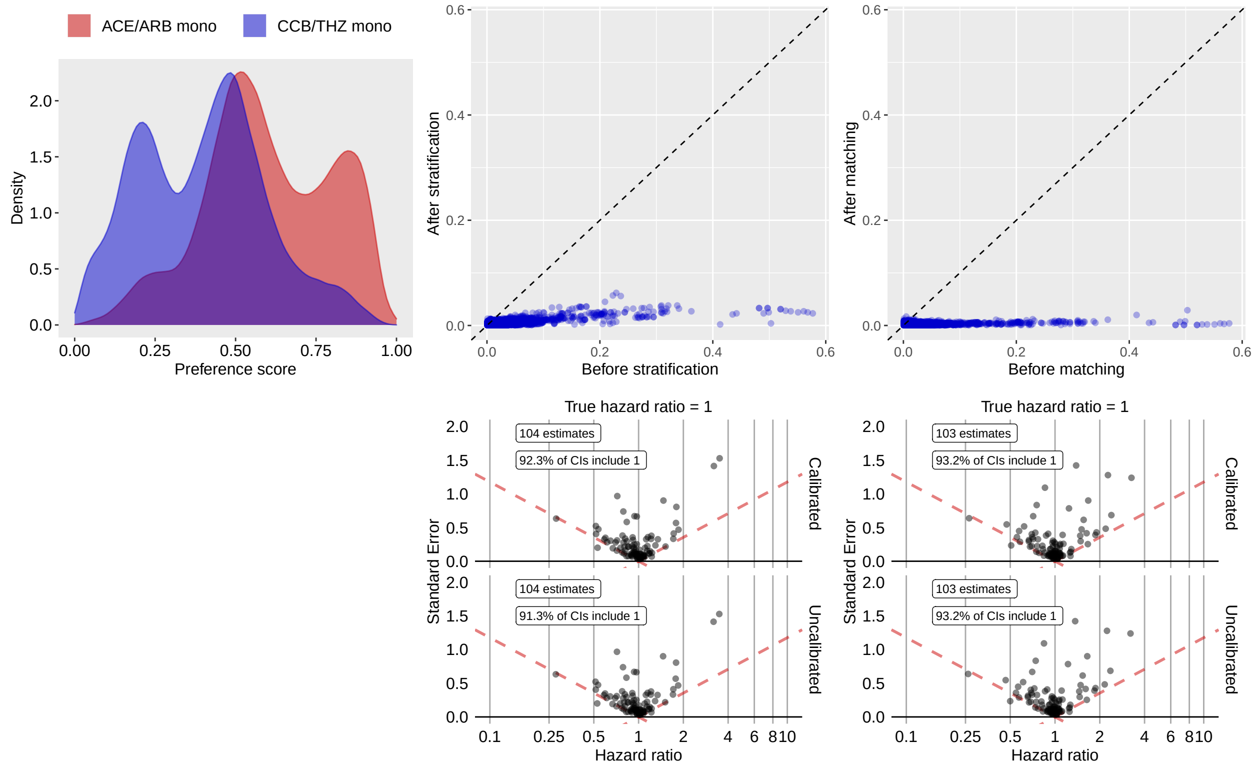

Supplementary Table 105: Most extreme patient characteristics balance diagnostics after stratification for ACE/ARB mono vs CCB/THZ mono in the VA-OMOP data source.

	Before stratification			After stratification		
Characteristic (total count = 11183)	T (%)	C (%)	SDf	T (%)	C (%)	SDf
drug_era group during day 0 through 0 days relative to index: Potassium Chloride	1.5	5.7	-0.23	2.4	3.5	-0.06
drug_era group during day -30 through 0 days relative to index: Potassium Chloride	2.6	7.4	-0.22	3.7	4.9	-0.06
measurement below normal range during day -180 through 0 days relative to index: Potassium [Moles/volume] in Serum or Plasma	2.2	7.2	-0.24	3.5	4.6	-0.06
drug_era group during day -180 through 0 days relative to index: Potassium Chloride	7.0	13.2	-0.21	8.6	9.9	-0.04
drug_era group during day -180 through 0 days relative to index: INSULINS AND ANALOGUES	14.3	4.6	0.34	10.5	9.4	0.04
drug_era group during day -30 through 0 days relative to index: INSULINS AND ANALOGUES	12.4	3.8	0.32	9.1	8.0	0.04

(Continued)	T (%)	C (%)	SDf	T (%)	C (%)	SDf
drug_era group during day 0 through 0 days relative to index: INSULINS AND ANALOGUES	11.3	3.4	0.31	8.3	7.2	0.04
drug_era group during day -180 through 0 days relative to index: Sodium-glucose co-transporter 2 (SGLT2) inhibitors	2.9	0.6	0.18	2.0	1.5	0.04
drug_era group during day 0 through 0 days relative to index: Sodium-glucose co-transporter 2 (SGLT2) inhibitors	2.5	0.5	0.16	1.7	1.3	0.04
drug_era group during day -180 through 0 days relative to index: Insulins and analogues for injection, long-acting	11.7	3.6	0.31	8.5	7.6	0.04

Supplementary Table 106: Most extreme patient characteristics balance diagnostics after matching for ACE/ARB mono vs CCB/THZ mono in the VA-OMOP data source.

	Before matching			After matching		
Characteristic (total count = 10623)	T (%)	C (%)	SDf	T (%)	C (%)	SDf
race = Black or African American	12.9	33.5	-0.50	19.8	18.6	0.03
race = White	77.5	58.6	0.41	71.1	72.0	-0.02
condition_era group during day -180 through 0 days relative to index: Nephritis	0.1	0.1	0.01	0.1	0.1	0.02
condition_era group during day -180 through 0 days relative to index: Corneal edema	0.1	0.1	-0.00	0.1	0.1	0.01
condition_era group during day -180 through 0 days relative to index: Transplanted liver present	0.1	0.1	-0.02	0.1	0.1	-0.01
Diabetes Comorbidity Severity Index (DCSI)	258.0	201.5	0.23	209.7	206.8	0.01
observation during day -180 through 0 days relative to index: Overexertion, strenuous movements and vibration	0.1	0.1	0.01	0.1	0.1	0.01
CHADS2VASc	270.3	249.5	0.16	255.8	254.4	0.01
condition_era group during day -180 through 0 days relative to index: Diabetic complication	21.0	8.4	0.36	10.5	10.1	0.01
drug_era group during day -180 through 0 days relative to index: Ticagrelor	0.1	0.1	0.02	0.1	0.1	0.01

Supplementary Figure 22: Preference score distributions, propensity score stratified (middle column) and matched (right column) covariate balance and negative control error distributions (bottom row) for ACE/ARB +combo vs CCB/THZ +combo in the VA-OMOP data source.

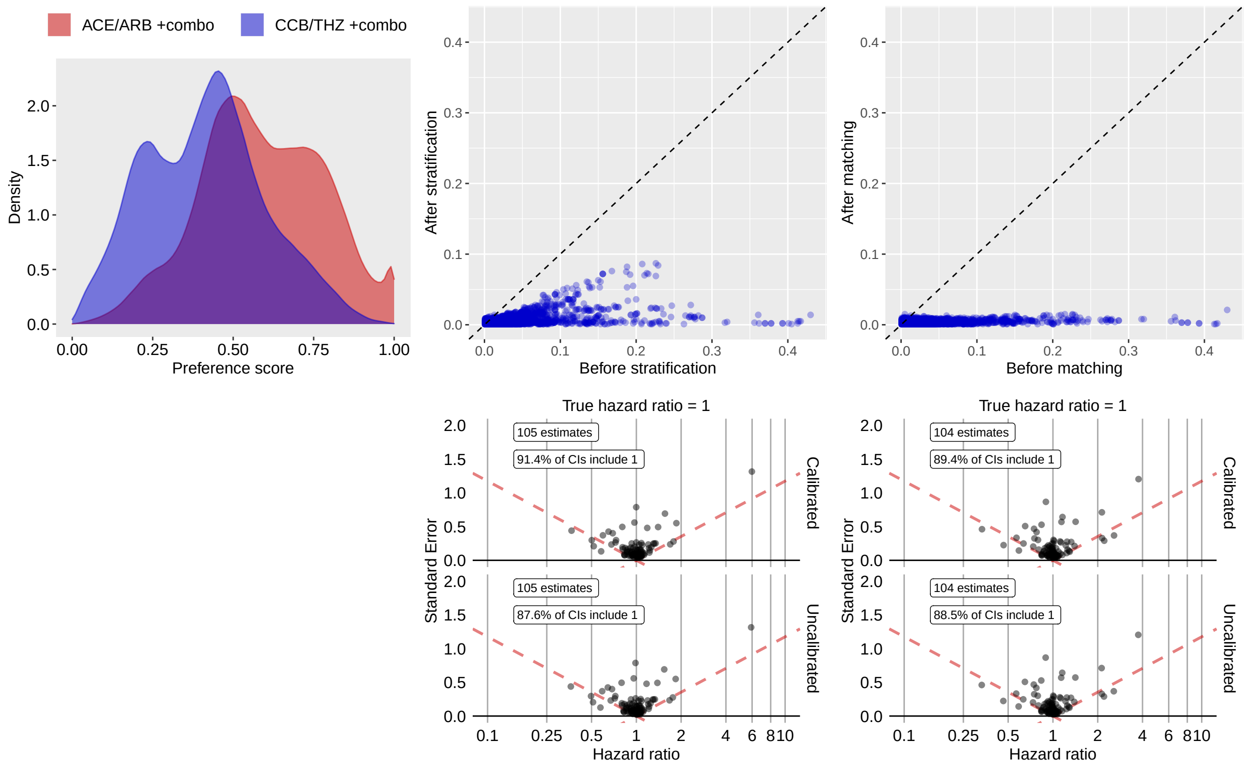

Supplementary Table 107: Most extreme patient characteristics balance diagnostics after stratification for ACE/ARB +combo vs CCB/THZ +combo in the VA-OMOP data source.

	Before stratification			After stratification		
Characteristic (total count = 11817)	T (%)	C (%)	SDf	T (%)	C (%)	SDf
condition_era group during day -180 through 0 days relative to index: Systolic heart failure	6.2	1.8	0.23	4.8	3.1	0.09
condition_era group during day -180 through 0 days relative to index: Chronic systolic heart failure	4.7	1.2	0.21	3.6	2.1	0.09
condition_era group during day -180 through 0 days relative to index: Systolic dysfunction	6.6	2.0	0.23	5.0	3.4	0.08
condition_era group during day -180 through 0 days relative to index: Chronic congestive heart failure	3.5	0.8	0.19	2.6	1.4	0.08
observation during day -180 through 0 days relative to index: History of cardiovascular surgery	2.7	0.6	0.17	2.0	1.1	0.08
condition_era group during day -180 through 0 days relative to index: Cardiomyopathy	3.5	0.8	0.19	2.6	1.5	0.07
condition_era group during day -180 through 0 days relative to index: Myocardial disease	7.4	2.7	0.21	5.8	4.2	0.07
condition_era group during day -180 through 0 days relative to index: Abnormal cardiovascular function	8.0	3.1	0.22	6.4	4.7	0.07

(Continued)	T (%)	C (%)	SDf	T (%)	C (%)	SDf
condition_era group during day -180 through 0 days relative to index: Ischemic myocardial dysfunction	2.1	0.4	0.16	1.5	0.8	0.07
condition_era group during day -180 through 0 days relative to index: Generalized ischemic myocardial dysfunction	2.1	0.4	0.16	1.5	0.8	0.07

Supplementary Table 108: Most extreme patient characteristics balance diagnostics after matching for ACE/ARB +combo vs CCB/THZ +combo in the VA-OMOP data source.

	Before matching			After matching		
Characteristic (total count = 11614)	T (%)	C (%)	SDf	T (%)	C (%)	SDf
race = Black or African American	13.0	30.3	-0.43	19.7	18.9	0.02
condition_era group during day -180 through 0 days relative to index: Abnormal cardiovascular function	8.0	3.1	0.22	3.5	3.2	0.01
condition_era group during day -180 through 0 days relative to index: Systolic heart failure	6.2	1.8	0.23	2.2	2.0	0.01
condition_era group during day -180 through 0 days relative to index: Congenital renal cyst	0.1	0.1	-0.01	0.1	0.1	0.01
condition_era group during day -180 through 0 days relative to index: Systolic dysfunction	6.6	2.0	0.23	2.4	2.2	0.01
condition_era group during day -180 through 0 days relative to index: Heart failure	9.7	4.0	0.23	4.5	4.2	0.01
condition_era group during day -180 through 0 days relative to index: Congestive heart failure	8.2	3.3	0.21	3.6	3.4	0.01
condition_era group during day -30 through 0 days relative to index: Systolic heart failure	2.8	0.8	0.15	1.0	0.9	0.01
condition_era group during day -180 through 0 days relative to index: Chronic heart failure	6.0	2.2	0.19	2.5	2.3	0.01
condition_era group during day -30 through 0 days relative to index: Heart failure	4.6	1.9	0.15	2.1	1.9	0.01

Supplementary Figure 23: Preference score distributions, propensity score stratified (middle column) and matched (right column) covariate balance and negative control error distributions (bottom row) for ACE/ARB mono vs CCB mono in the VA-OMOP data source.

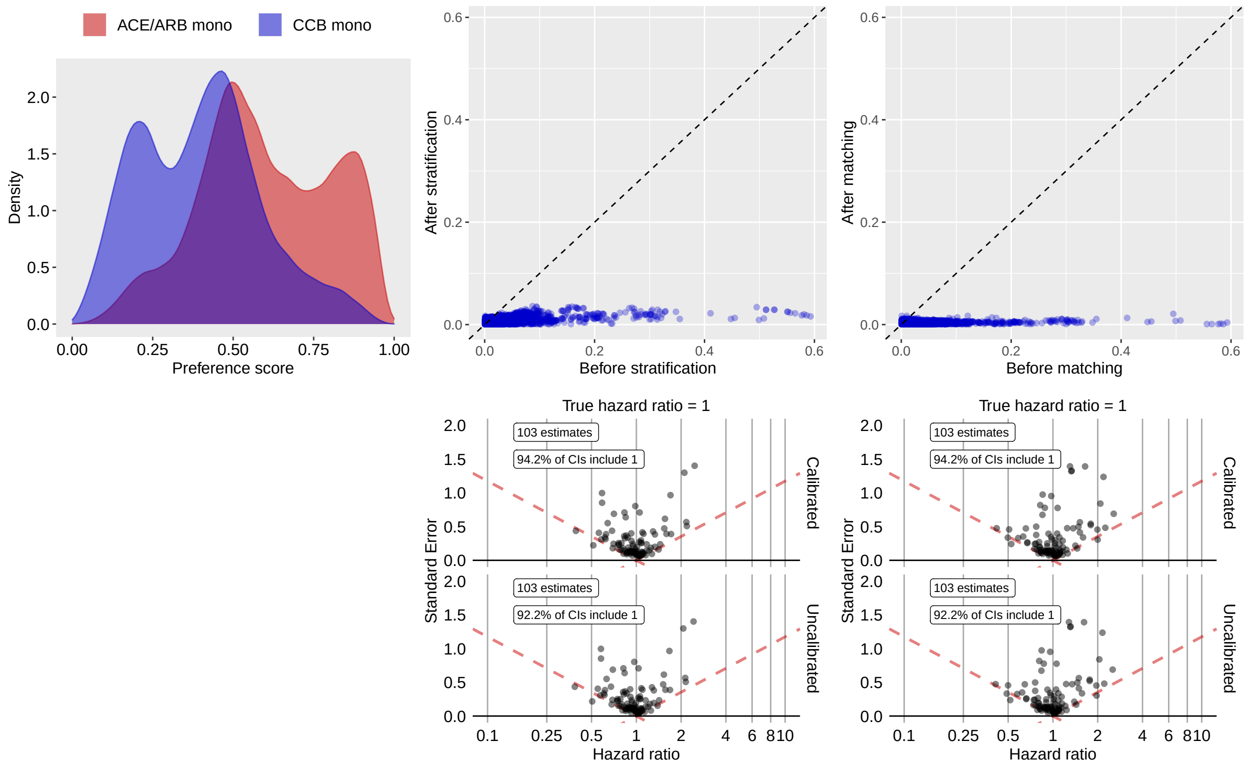

Supplementary Table 109: Most extreme patient characteristics balance diagnostics after stratification for ACE/ARB mono vs CCB mono in the VA-OMOP data source.

	Before stratification			After stratification		
Characteristic (total count = 11555)	T (%)	C (%)	SDf	T (%)	C (%)	SDf
condition_era group during day -180 through 0 days relative to index: End-stage renal disease	0.1	0.6	-0.09	0.2	0.3	-0.04
drug_era group during day -30 through 0 days relative to index: Glucagon-like peptide-1 (GLP-1) analogues	2.3	0.4	0.16	1.7	1.3	0.04
measurement above normal range during day -180 through 0 days relative to index: Creatinine [Mass/volume] in Serum or Plasma	11.3	14.4	-0.09	12.1	13.3	-0.04
race = Black or African American	12.9	33.1	-0.49	17.9	19.2	-0.03
condition_era group during day -180 through 0 days relative to index: Renal failure syndrome	0.8	2.0	-0.10	1.0	1.4	-0.03
drug_era group during day -180 through 0 days relative to index: Glucagon-like peptide-1 (GLP-1) analogues	2.5	0.5	0.17	2.0	1.5	0.03
measurement below normal range during day -180 through 0 days relative to index: Potassium [Moles/volume] in Serum or Plasma	2.2	5.0	-0.15	2.8	3.4	-0.03
drug_era group during day 0 through 0 days relative to index: Glucagon-like peptide-1 (GLP-1) analogues	2.1	0.4	0.15	1.6	1.2	0.03

(Continued)	T (%)	C (%)	SDf	T (%)	C (%)	SDf
drug_era group during day 0 through 0 days relative to index: Sodium-glucose co-transporter 2 (SGLT2) inhibitors	2.5	0.5	0.17	1.9	1.5	0.03
drug_era group during day -30 through 0 days relative to index: Simvastatin	15.2	6.9	0.27	12.8	11.7	0.03

Supplementary Table 110: Most extreme patient characteristics balance diagnostics after matching for ACE/ARB mono vs CCB mono in the VA-OMOP data source.

	Before matching			After matching		
Characteristic (total count = 11106)	T (%)	C (%)	SDf	T (%)	C (%)	SDf
race = Black or African American	12.9	33.1	-0.49	24.2	23.3	0.02
CHADS2VASc	270.3	254.8	0.12	258.7	256.5	0.02
condition_era group during day -180 through 0 days relative to index: Nephritis	0.1	0.1	-0.00	0.1	0.1	0.02
drug_era group during day -180 through 0 days relative to index: PLANT ALKALOIDS AND OTHER NATURAL PRODUCTS	0.1	0.1	-0.01	0.1	0.1	0.02
Charlson index - Romano adaptation	311.8	283.6	0.10	280.4	276.4	0.01
CHADS2	202.1	184.7	0.17	188.0	186.5	0.01
Diabetes Comorbidity Severity Index (DCSI)	258.0	214.7	0.17	216.3	213.3	0.01
race = White	77.5	58.7	0.41	66.8	67.4	-0.01
condition_era group during day -180 through 0 days relative to index: End-stage renal disease	0.1	0.6	-0.09	0.2	0.3	-0.01
measurement above normal range during day -180 through 0 days relative to index: Creatinine [Mass/volume] in Body fluid	0.1	0.1	-0.01	0.1	0.1	0.01

Supplementary Figure 24: Preference score distributions, propensity score stratified (middle column) and matched (right column) covariate balance and negative control error distributions (bottom row) for ACE/ARB +combo vs CCB +combo in the VA-OMOP data source.

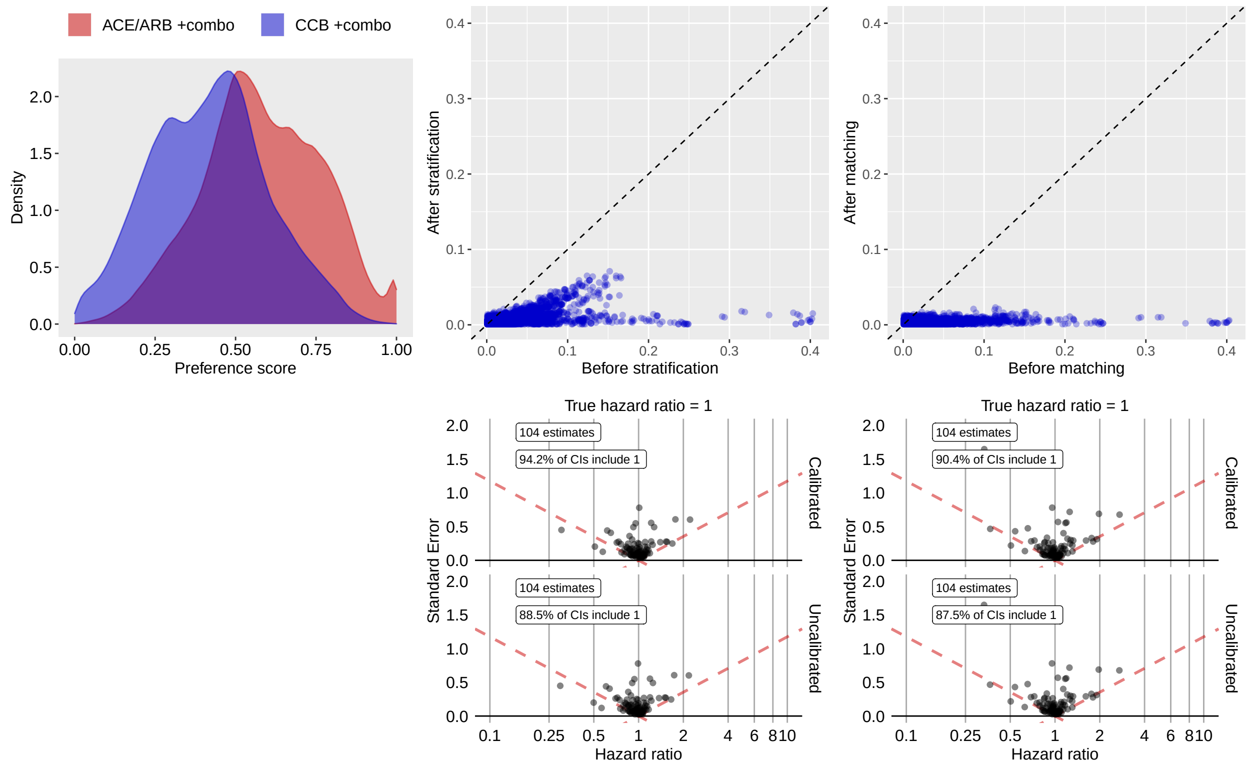

Supplementary Table 111: Most extreme patient characteristics balance diagnostics after stratification for ACE/ARB +combo vs CCB +combo in the VA-OMOP data source.

	Before stratification			After stratification		
Characteristic (total count = 12315)	T (%)	C (%)	SDf	T (%)	C (%)	SDf
condition_era group during day -180 through 0 days relative to index: End-stage renal disease	0.3	1.8	-0.15	0.4	0.9	-0.07
condition_era group during day -180 through 0 days relative to index: Chronic kidney disease stage 5	0.1	1.0	-0.12	0.2	0.5	-0.07
condition_era group during day -180 through 0 days relative to index: Chronic systolic heart failure	3.7	1.2	0.16	3.1	2.1	0.07
condition_era group during day -180 through 0 days relative to index: Systolic heart failure	4.8	1.9	0.17	4.2	3.0	0.06
observation during day -180 through 0 days relative to index: History of cardiovascular surgery	2.1	0.6	0.13	1.8	1.0	0.06
drug_era group during day -180 through 0 days relative to index: Drugs for treatment of hyperkalemia and hyperphosphatemia	0.4	2.1	-0.15	0.6	1.2	-0.06
condition_era group during day -180 through 0 days relative to index: Systolic dysfunction	5.1	2.1	0.17	4.4	3.2	0.06
condition_era group during day -180 through 0 days relative to index: Chronic kidney disease stage 4	0.6	2.6	-0.16	0.8	1.5	-0.06

(Continued)	T (%)	C (%)	SDf	T (%)	C (%)	SDf
condition_era group during day -180 through 0 days relative to index: Chronic congestive heart failure	2.7	0.8	0.15	2.2	1.4	0.06
condition_era group during day -180 through 0 days relative to index: Hypertensive renal failure	0.1	0.9	-0.11	0.2	0.5	-0.06

Supplementary Table 112: Most extreme patient characteristics balance diagnostics after matching for ACE/ARB +combo vs CCB +combo in the VA-OMOP data source.

	Before matching			After matching		
Characteristic (total count = 11864)	T (%)	C (%)	SDf	T (%)	C (%)	SDf
CHADS2VASc	308.5	292.1	0.11	292.8	289.5	0.02
Diabetes Comorbidity Severity Index (DCSI)	333.3	307.6	0.09	302.8	296.6	0.02
measurement above normal range during day -180 through 0 days relative to index: Creatinine [Mass/volume] in Serum or Plasma	17.6	22.4	-0.12	20.7	19.9	0.02
CHADS2	228.3	214.5	0.11	214.7	212.2	0.02
condition_era group during day -180 through 0 days relative to index: End-stage renal disease	0.3	1.8	-0.15	0.7	0.8	-0.02
condition_era group during day -180 through 0 days relative to index: Heart disease	31.2	24.5	0.15	24.7	24.0	0.02
measurement within normal range during day -180 through 0 days relative to index: Carbon dioxide, total [Moles/volume] in Serum or Plasma	69.5	68.4	0.02	68.1	67.3	0.02
Charlson index - Romano adaptation	381.2	369.4	0.04	359.8	354.6	0.02
condition_era group during day -180 through 0 days relative to index: Chronic kidney disease stage 3	5.2	7.4	-0.09	6.9	6.5	0.02
condition_era group during day -180 through 0 days relative to index: Chronic kidney disease stage 5	0.1	1.0	-0.12	0.3	0.4	-0.02

Supplementary Figure 25: Preference score distributions, propensity score stratified (middle column) and matched (right column) covariate balance and negative control error distributions (bottom row) for ACE/ARB mono vs THZ mono in the VA-OMOP data source.

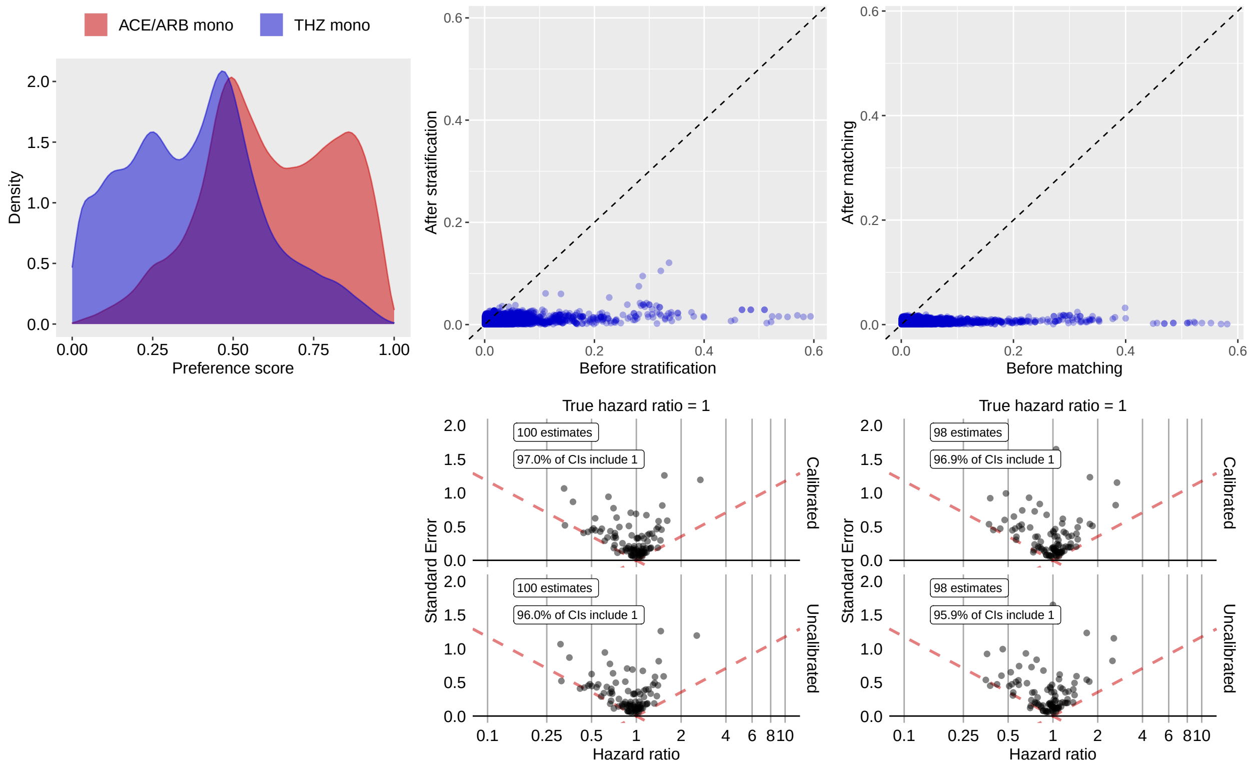

Supplementary Table 113: Most extreme patient characteristics balance diagnostics after stratification for ACE/ARB mono vs THZ mono in the VA-OMOP data source.

	Before stratification			After stratification		
Characteristic (total count = 11069)	T (%)	C (%)	SDf	T (%)	C (%)	SDf
drug_era group during day 0 through 0 days relative to index: Potassium Chloride	1.5	8.8	-0.34	1.8	3.8	-0.12
drug_era group during day -30 through 0 days relative to index: Potassium Chloride	2.6	10.4	-0.32	2.9	4.9	-0.10
measurement below normal range during day -180 through 0 days relative to index: Potassium [Moles/volume] in Serum or Plasma	2.2	8.7	-0.29	2.6	4.3	-0.10
drug_era group during day -180 through 0 days relative to index: Potassium Chloride	7.0	15.8	-0.28	7.4	9.5	-0.07
condition_era group during day -180 through 0 days relative to index: Potassium disorder	0.8	2.2	-0.11	0.9	1.6	-0.06
condition_era group during day -180 through 0 days relative to index: Hypokalemia	0.4	1.9	-0.14	0.5	1.0	-0.06
measurement above normal range during day -180 through 0 days relative to index: Carbon dioxide, total [Moles/volume] in Serum or Plasma	2.1	6.8	-0.23	2.6	3.5	-0.05

(Continued)	T (%)	C (%)	SDf	T (%)	C (%)	SDf
drug_era group during day -30 through 0 days relative to index: HMG CoA reductase inhibitors	57.7	43.1	0.29	55.4	53.3	0.04
drug_era group during day 0 through 0 days relative to index: HMG CoA reductase inhibitors	55.1	41.0	0.28	52.8	50.8	0.04
CHADS2VASc	270.3	238.4	0.26	265.3	270.4	-0.04

Supplementary Table 114: Most extreme patient characteristics balance diagnostics after matching for ACE/ARB mono vs THZ mono in the VA-OMOP data source.

	Before matching			After matching		
Characteristic (total count = 10870)	T (%)	C (%)	SDf	T (%)	C (%)	SDf
race = Black or African American	12.9	28.8	-0.40	27.4	26.0	0.03
race = White	77.5	63.3	0.31	64.6	65.8	-0.02
measurement below normal range during day -180 through 0 days relative to index: Potassium [Moles/volume] in Blood	0.1	0.2	-0.04	0.1	0.2	-0.02
condition_era group during day -30 through 0 days relative to index: Gastrointestinal obstruction	0.1	0.1	0.01	0.1	0.1	0.02
drug_era group during day -30 through 0 days relative to index: LIPID MODIFYING AGENTS	59.3	44.5	0.30	44.4	45.3	-0.02
drug_era group during day 0 through 0 days relative to index: LIPID MODIFYING AGENTS	56.6	42.3	0.29	42.2	43.1	-0.02
drug_era group during day -30 through 0 days relative to index: LIPID MODIFYING AGENTS, PLAIN	59.3	44.5	0.30	44.4	45.3	-0.02
drug_era group during day 0 through 0 days relative to index: LIPID MODIFYING AGENTS, PLAIN	56.6	42.3	0.29	42.2	43.1	-0.02
condition_era group during day -180 through 0 days relative to index: Dehydration	0.5	0.3	0.03	0.4	0.3	0.02
procedure_occurrence during day -30 through 0 days relative to index: Detoxification Services for Substance Abuse Treatment	0.1	0.1	0.01	0.1	0.1	0.02

Supplementary Figure 26: Preference score distributions, propensity score stratified (middle column) and matched (right column) covariate balance and negative control error distributions (bottom row) for ACE/ARB +combo vs THZ +combo in the VA-OMOP data source.

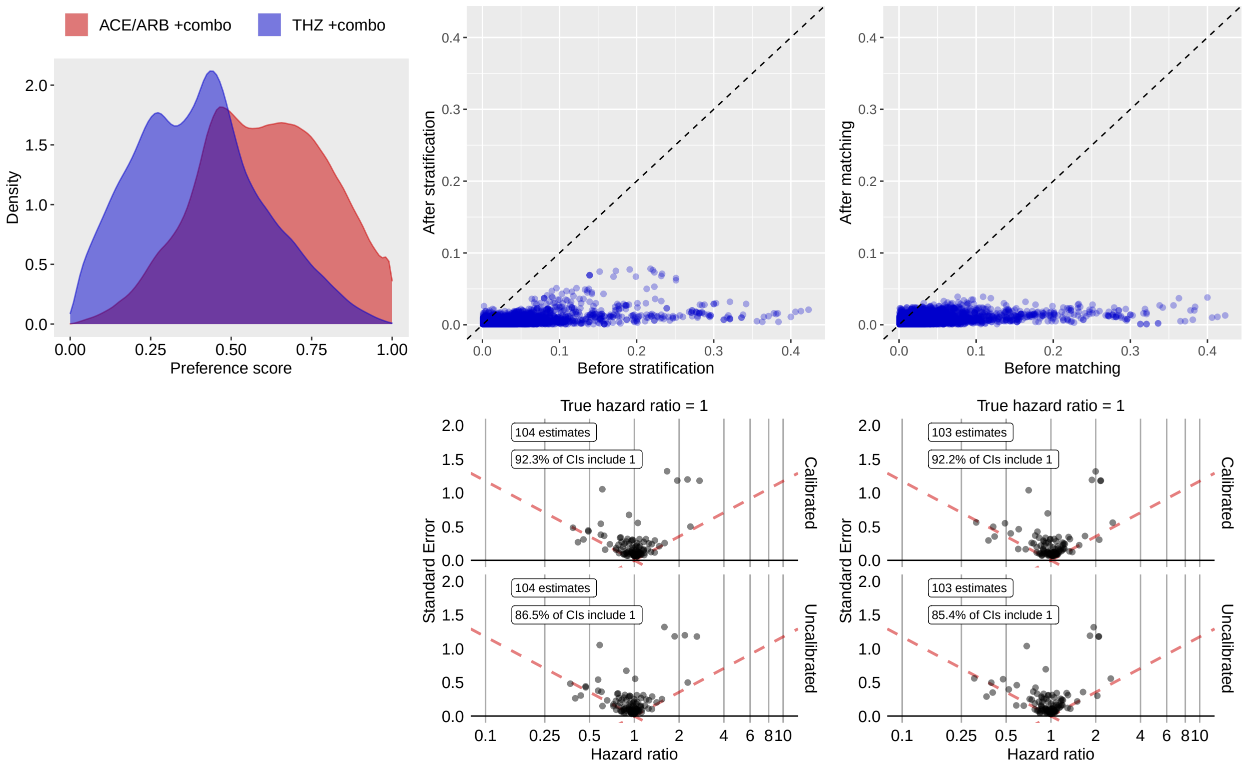

Supplementary Table 115: Most extreme patient characteristics balance diagnostics after stratification for ACE/ARB +combo vs THZ +combo in the VA-OMOP data source.

	Before stratification			After stratification		
Characteristic (total count = 11939)	T (%)	C (%)	SDf	T (%)	C (%)	SDf
condition_era group during day -180 through 0 days relative to index: Systolic heart failure	5.5	1.5	0.22	4.9	3.4	0.08
condition_era group during day -180 through 0 days relative to index: Chronic systolic heart failure	4.2	1.1	0.19	3.7	2.4	0.08
condition_era group during day -180 through 0 days relative to index: Systolic dysfunction	5.9	1.7	0.22	5.2	3.7	0.08
condition_era group during day -180 through 0 days relative to index: Chronic congestive heart failure	3.0	0.7	0.17	2.6	1.6	0.07
observation during day -180 through 0 days relative to index: History of cardiovascular surgery	2.3	0.5	0.15	2.0	1.1	0.07
condition_era group during day -180 through 0 days relative to index: Myocardial disease	6.8	2.2	0.22	6.0	4.4	0.07
condition_era group during day -180 through 0 days relative to index: Abnormal cardiovascular function	7.4	2.4	0.23	6.7	5.0	0.07
condition_era group during day -180 through 0 days relative to index: Ischemic myocardial dysfunction	1.8	0.3	0.14	1.5	0.8	0.07

(Continued)	T (%)	C (%)	SDf	T (%)	C (%)	SDf
condition_era group during day -180 through 0 days relative to index: Generalized ischemic myocardial dysfunction	1.8	0.3	0.14	1.5	0.8	0.07
condition_era group during day -180 through 0 days relative to index: Myocardial dysfunction	1.8	0.3	0.14	1.5	0.8	0.07

Supplementary Table 116: Most extreme patient characteristics balance diagnostics after matching for ACE/ARB +combo vs THZ +combo in the VA-OMOP data source.

	Before matching			After matching		
Characteristic (total count = 12154)	T (%)	C (%)	SDf	T (%)	C (%)	SDf
condition_era group during day -180 through 0 days relative to index: Renal impairment	12.3	9.0	0.11	10.1	9.0	0.04
measurement above normal range during day -180 through 0 days relative to index: Creatinine [Mass/volume] in Serum or Plasma	19.9	17.0	0.08	18.1	16.6	0.04
Diabetes Comorbidity Severity Index (DCSI)	367.5	253.2	0.40	267.3	257.2	0.04
Charlson index - Romano adaptation	416.0	305.5	0.36	319.4	308.8	0.04
condition_era group during day -180 through 0 days relative to index: Kidney disease	15.8	11.7	0.12	12.9	11.7	0.04
condition_era group during day -180 through 0 days relative to index: Chronic disease of genitourinary system	11.3	8.5	0.09	9.4	8.4	0.04
condition_era group during day -180 through 0 days relative to index: Chronic kidney disease	11.1	8.3	0.10	9.2	8.2	0.04
measurement above normal range during day -180 through 0 days relative to index: Urea nitrogen [Mass/volume] in Serum or Plasma	20.7	17.4	0.08	18.4	17.1	0.03
condition_era group during day -180 through 0 days relative to index: Renal failure syndrome	2.8	1.9	0.06	2.3	1.8	0.03
measurement below normal range during day -180 through 0 days relative to index: Hemoglobin [Mass/volume] in Blood	23.9	17.3	0.16	18.4	17.2	0.03

Supplementary Figure 27: Preference score distributions, propensity score stratified (middle column) and matched (right column) covariate balance and negative control error distributions (bottom row) for ACE mono vs ARB mono in the VA-OMOP data source.

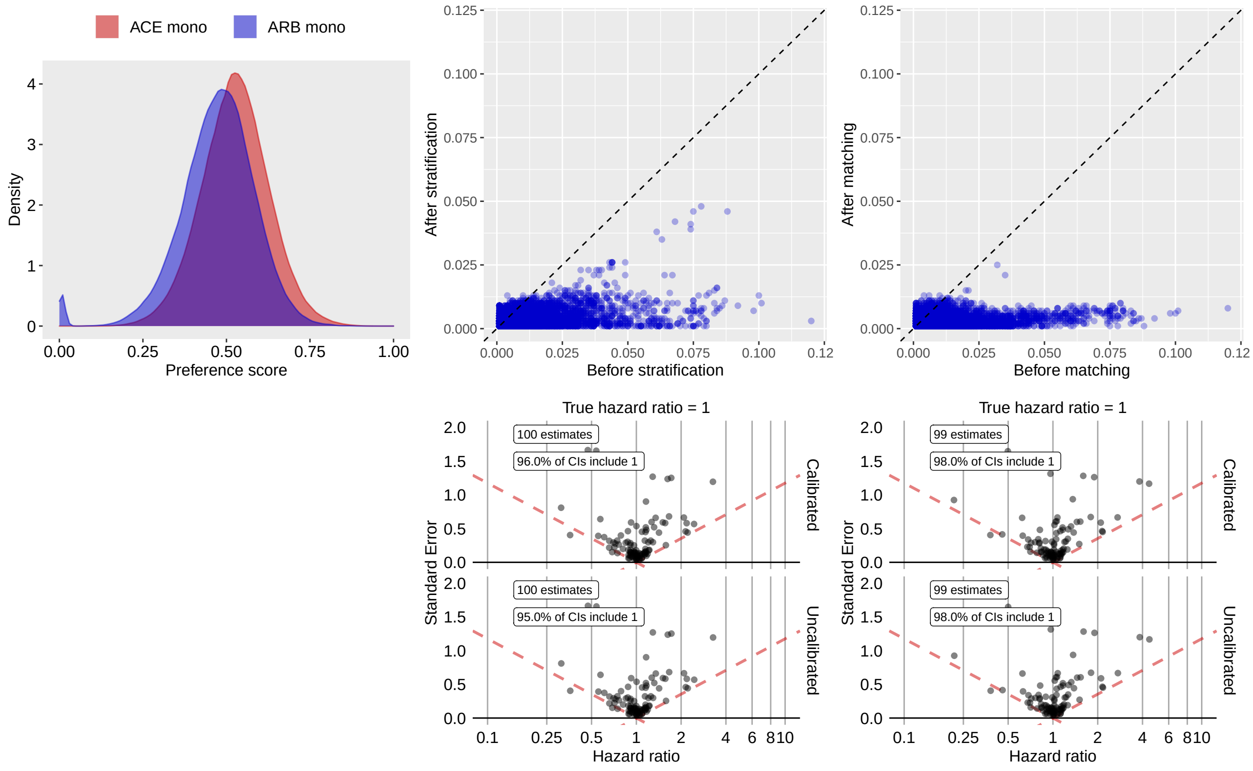

Supplementary Table 117: Most extreme patient characteristics balance diagnostics after stratification for ACE mono vs ARB mono in the VA-OMOP data source.

	Before stratification			After stratification		
Characteristic (total count = 10638)	T (%)	C (%)	SDf	T (%)	C (%)	SDf
condition_era group during day -180 through 0 days relative to index: Systolic dysfunction	0.3	0.9	-0.08	0.3	0.7	-0.05
condition_era group during day -180 through 0 days relative to index: Heart failure	0.8	1.8	-0.09	0.9	1.3	-0.05
condition_era group during day -180 through 0 days relative to index: Systolic heart failure	0.3	0.9	-0.07	0.3	0.6	-0.05
condition_era group during day -180 through 0 days relative to index: Chronic systolic heart failure	0.2	0.6	-0.07	0.2	0.4	-0.04
condition_era group during day -180 through 0 days relative to index: Abnormal cardiovascular function	0.5	1.2	-0.07	0.5	0.9	-0.04
condition_era group during day -180 through 0 days relative to index: Congestive heart failure	0.6	1.2	-0.07	0.6	0.9	-0.04
condition_era group during day -180 through 0 days relative to index: Chronic congestive heart failure	0.2	0.5	-0.06	0.2	0.4	-0.04
condition_era group during day -180 through 0 days relative to index: Chronic heart failure	0.3	0.8	-0.06	0.3	0.6	-0.04

(Continued)	T (%)	C (%)	SDf	T (%)	C (%)	SDf
condition_era group during day -30 through 0 days relative to index: Systolic dysfunction	0.1	0.3	-0.04	0.1	0.2	-0.03
condition_era group during day -30 through 0 days relative to index: Heart failure	0.3	0.6	-0.05	0.3	0.5	-0.03

Supplementary Table 118: Most extreme patient characteristics balance diagnostics after matching for ACE mono vs ARB mono in the VA-OMOP data source.

	Before matching			After matching		
Characteristic (total count = 10501)	T (%)	C (%)	SDf	T (%)	C (%)	SDf
condition_era group during day -180 through 0 days relative to index: End-stage renal disease	0.1	0.2	-0.03	0.1	0.2	-0.03
condition_era group during day -180 through 0 days relative to index: Cough variant asthma	0.1	0.2	-0.04	0.1	0.2	-0.02
condition_era group during day -180 through 0 days relative to index: Nephritis	0.1	0.1	-0.02	0.1	0.1	-0.01
condition_era group during day -180 through 0 days relative to index: Glomerular disease	0.1	0.1	-0.02	0.1	0.1	-0.01
condition_era group during day -30 through 0 days relative to index: Mitral valve disorder	0.1	0.1	0.01	0.1	0.1	0.01
measurement during day -30 through 0 days relative to index: Urobilinogen, urine; quantitative, timed specimen	0.1	0.1	0.00	0.2	0.1	0.01
measurement during day -180 through 0 days relative to index: Infectious agent antigen detection by immunoassay with direct optical observation; Influenza	0.1	0.1	0.01	0.1	0.1	0.01
measurement during day -180 through 0 days relative to index: Copper	0.1	0.1	-0.01	0.1	0.1	-0.01
device_exposure during day -180 through 0 days relative to index: Knee orthosis, double upright, thigh and calf, with adjustable flexion and extension joint (unicentric or polycentric), medial-lateral and rotation control, with or without varus/valgus adjustment, prefabricated item that has been trimmed, bent, molded, a	0.1	0.1	-0.01	0.1	0.1	-0.01
condition_era group during day -180 through 0 days relative to index: Congenital anomaly of digit	0.1	0.1	-0.01	0.1	0.1	-0.01

Supplementary Figure 28: Preference score distributions, propensity score stratified (middle column) and matched (right column) covariate balance and negative control error distributions (bottom row) for ACE +combo vs ARB +combo in the VA-OMOP data source.

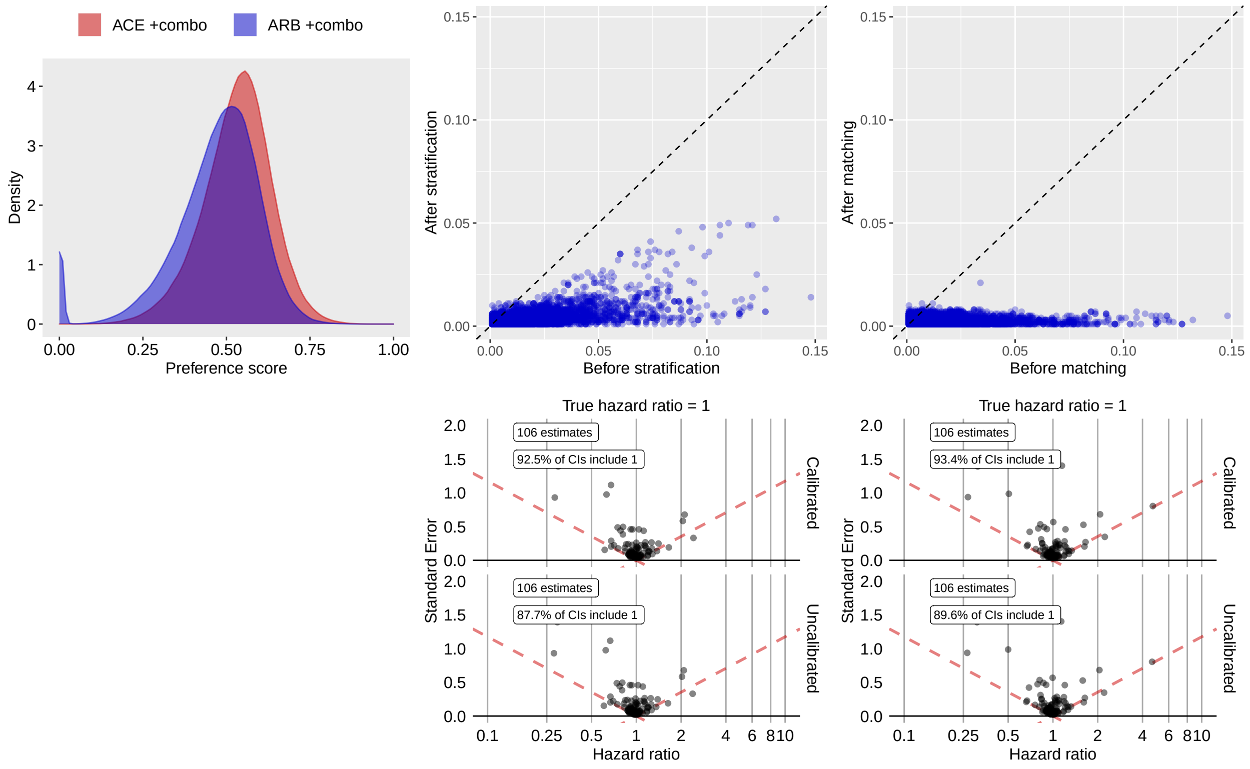

Supplementary Table 119: Most extreme patient characteristics balance diagnostics after stratification for ACE +combo vs ARB +combo in the VA-OMOP data source.

	Before stratification			After stratification		
Characteristic (total count = 11429)	T (%)	C (%)	SDf	T (%)	C (%)	SDf
condition_era group during day -180 through 0 days relative to index: Heart failure	6.0	9.6	-0.13	6.5	7.9	-0.05
condition_era group during day -180 through 0 days relative to index: Systolic dysfunction	3.8	6.1	-0.11	4.0	5.1	-0.05
condition_era group during day -180 through 0 days relative to index: Abnormal cardiovascular function	4.9	7.8	-0.12	5.2	6.4	-0.05
condition_era group during day -180 through 0 days relative to index: Congestive heart failure	5.0	8.0	-0.12	5.4	6.6	-0.05
condition_era group during day -180 through 0 days relative to index: Systolic heart failure	3.5	5.8	-0.11	3.8	4.7	-0.05
condition_era group during day -180 through 0 days relative to index: Chronic systolic heart failure	2.6	4.4	-0.10	2.8	3.6	-0.05
condition_era group during day -180 through 0 days relative to index: Chronic congestive heart failure	1.8	3.2	-0.09	1.9	2.6	-0.05
condition_era group during day -180 through 0 days relative to index: Chronic heart failure	3.6	5.8	-0.11	3.8	4.7	-0.04

(Continued)	T (%)	C (%)	SDf	T (%)	C (%)	SDf
observation during day -180 through 0 days relative to index: History of cardiovascular surgery	1.4	2.5	-0.07	1.5	2.1	-0.04
condition_era group during day -30 through 0 days relative to index: Heart failure	2.8	4.6	-0.09	3.1	3.8	-0.04

Supplementary Table 120: Most extreme patient characteristics balance diagnostics after matching for ACE +combo vs ARB +combo in the VA-OMOP data source.

	Before matching			After matching		
Characteristic (total count = 11055)	T (%)	C (%)	SDf	T (%)	C (%)	SDf
condition_era group during day -180 through 0 days relative to index: Cough variant asthma	0.1	0.2	-0.03	0.1	0.2	-0.02
observation during day -180 through 0 days relative to index: Underweight	0.1	0.1	0.01	0.1	0.1	0.01
measurement below normal range during day -180 through 0 days relative to index: Thyrotropin [Units/volume] in Serum or Plasma by Detection limit <= 0.05 mIU/L	0.1	0.1	0.01	0.1	0.1	0.01
ethnicity = Not Hispanic or Latino	91.6	91.5	0.00	91.2	91.5	-0.01
condition_era group during day -180 through 0 days relative to index: Hypertrophy of breast	0.1	0.1	-0.01	0.1	0.1	-0.01
condition_era group during day -180 through 0 days relative to index: Fracture of femur	0.1	0.1	0.01	0.1	0.1	0.01
condition_era group during day -180 through 0 days relative to index: Hyperplasia of prostate	10.9	11.9	-0.03	12.1	11.8	0.01
condition_era group during day -180 through 0 days relative to index: Benign prostatic hyperplasia	10.9	11.9	-0.03	12.0	11.7	0.01
condition_era group during day -180 through 0 days relative to index: Bronchiectasis	0.1	0.2	-0.02	0.1	0.1	-0.01
condition_era group during day -180 through 0 days relative to index: Closed fracture of femur	0.1	0.1	0.01	0.1	0.1	0.01

Supplementary Figure 29: Preference score distributions, propensity score stratified (middle column) and matched (right column) covariate balance and negative control error distributions (bottom row) for ACE mono vs CCB/THZ mono in the VA-OMOP data source.

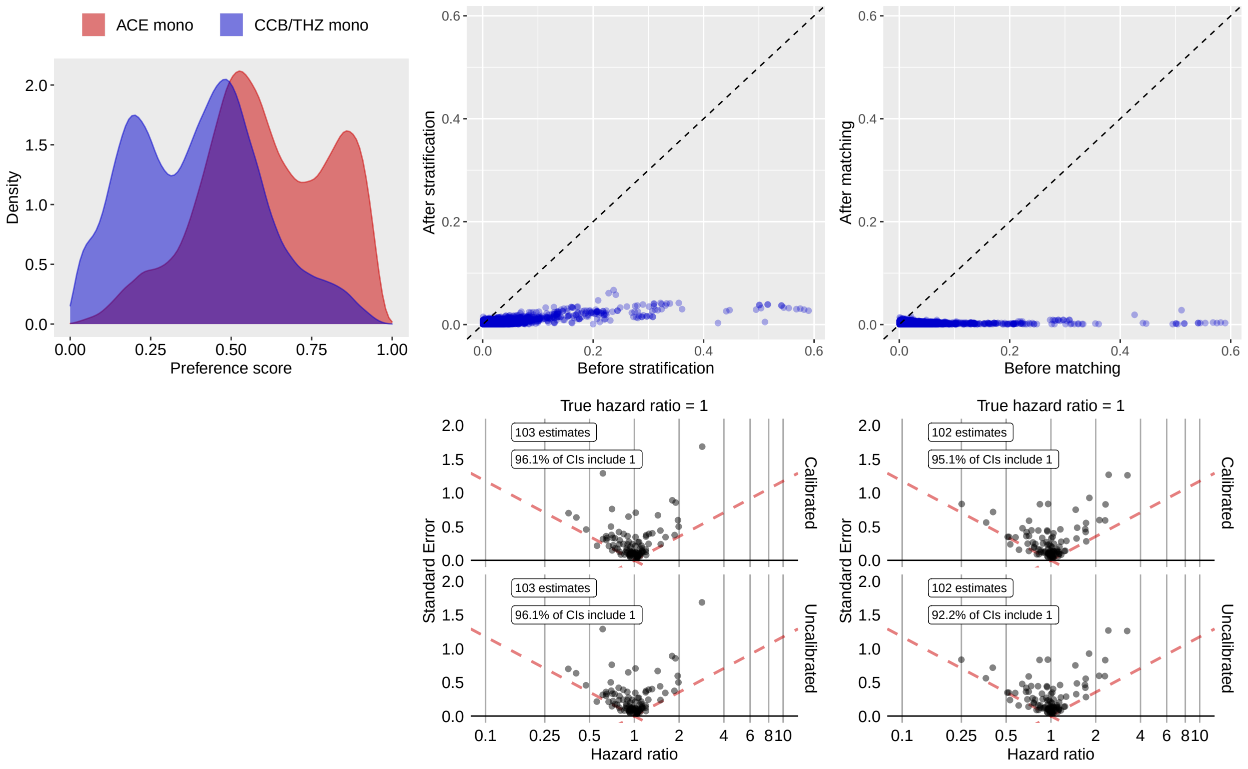

Supplementary Table 121: Most extreme patient characteristics balance diagnostics after stratification for ACE mono vs CCB/THZ mono in the VA-OMOP data source.

	Before stratification			After stratification		
Characteristic (total count = 11228)	T (%)	C (%)	SDf	T (%)	C (%)	SDf
drug_era group during day 0 through 0 days relative to index: Potassium Chloride	1.4	5.7	-0.24	2.6	3.7	-0.07
drug_era group during day -30 through 0 days relative to index: Potassium Chloride	2.5	7.4	-0.23	3.9	5.1	-0.06
measurement below normal range during day -180 through 0 days relative to index: Potassium [Moles/volume] in Serum or Plasma	2.2	7.2	-0.24	3.7	4.9	-0.06
drug_era group during day -180 through 0 days relative to index: Potassium Chloride	7.0	13.2	-0.21	8.9	10.3	-0.05
drug_era group during day -30 through 0 days relative to index: INSULINS AND ANALOGUES	12.5	3.8	0.32	8.5	7.3	0.04
drug_era group during day -180 through 0 days relative to index: Sulfonylureas	14.3	4.2	0.35	9.6	8.4	0.04
drug_era group during day -180 through 0 days relative to index: INSULINS AND ANALOGUES	14.4	4.6	0.34	9.9	8.7	0.04
drug_era group during day 0 through 0 days relative to index: INSULINS AND ANALOGUES	11.4	3.4	0.31	7.7	6.7	0.04

(Continued)	T (%)	C (%)	SDf	T (%)	C (%)	SDf
drug_era group during day -30 through 0 days relative to index: Sulfonylureas	12.5	3.6	0.33	8.4	7.3	0.04
drug_era group during day -180 through 0 days relative to index: Insulins and analogues for injection, long-acting	11.7	3.6	0.31	8.0	6.9	0.04

Supplementary Table 122: Most extreme patient characteristics balance diagnostics after matching for ACE mono vs CCB/THZ mono in the VA-OMOP data source.

	Before matching			After matching		
Characteristic (total count = 10508)	T (%)	C (%)	SDf	T (%)	C (%)	SDf
race = Black or African American	12.6	33.5	-0.51	17.4	16.4	0.03
race = White	78.0	58.6	0.43	73.4	74.3	-0.02
condition_era group during day -180 through 0 days relative to index: Transplanted liver present	0.1	0.1	-0.02	0.1	0.1	-0.02
condition_era group during day -180 through 0 days relative to index: Corneal edema	0.1	0.1	-0.00	0.1	0.1	0.01
drug_era group during day -180 through 0 days relative to index: Ticagrelor	0.1	0.1	0.02	0.1	0.1	0.01
condition_era group during day -180 through 0 days relative to index: Nephritis	0.1	0.1	-0.00	0.1	0.1	0.01
condition_era group during day -180 through 0 days relative to index: Cellulitis of finger	0.1	0.1	-0.01	0.1	0.1	-0.01
condition_era group during day -180 through 0 days relative to index: Cellulitis of hand	0.1	0.1	-0.01	0.1	0.1	-0.01
observation during day -180 through 0 days relative to index: Overexertion, strenuous movements and vibration	0.1	0.1	0.01	0.1	0.1	0.01
measurement above normal range during day -180 through 0 days relative to index: Platelets [#/volume] in Blood	0.1	0.1	-0.01	0.1	0.1	-0.01

Supplementary Figure 30: Preference score distributions, propensity score stratified (middle column) and matched (right column) covariate balance and negative control error distributions (bottom row) for ACE +combo vs CCB/THZ +combo in the VA-OMOP data source.

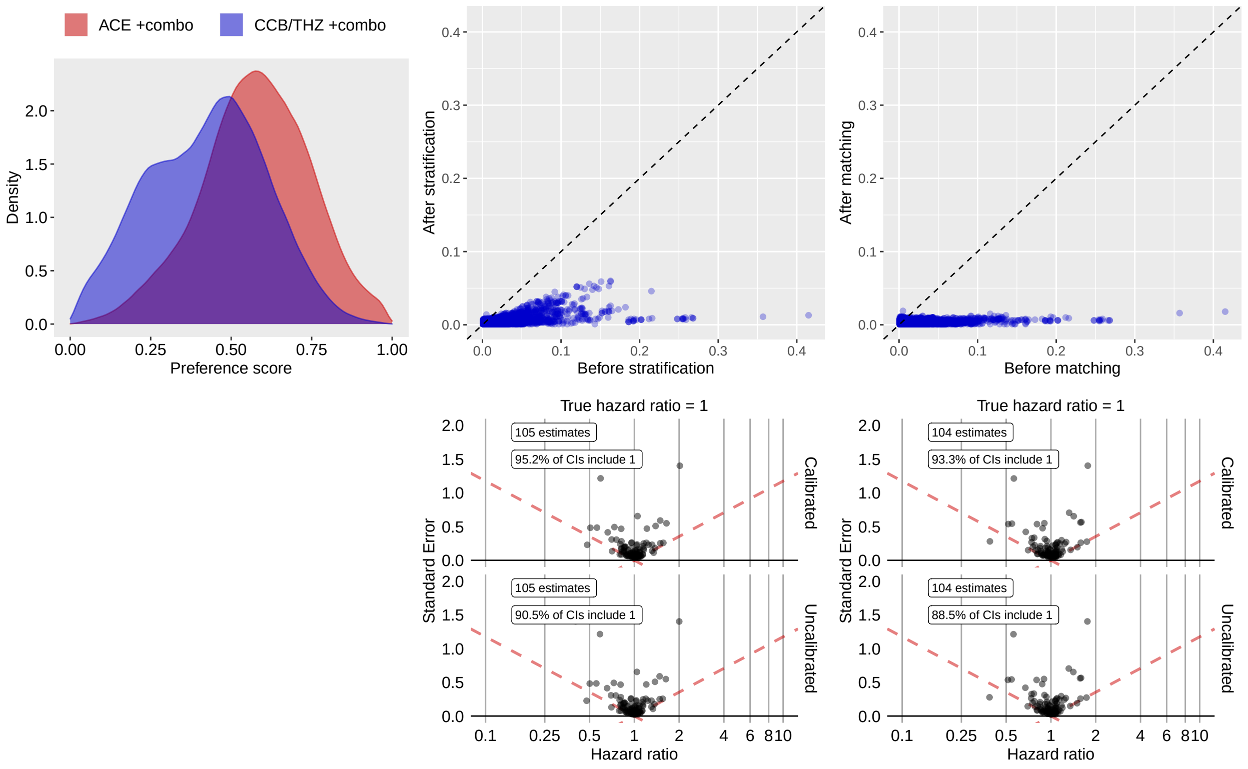

Supplementary Table 123: Most extreme patient characteristics balance diagnostics after stratification for ACE +combo vs CCB/THZ +combo in the VA-OMOP data source.

	Before stratification			After stratification		
Characteristic (total count = 11609)	T (%)	C (%)	SDf	T (%)	C (%)	SDf
condition_era group during day -180 through 0 days relative to index: Systolic heart failure	5.2	2.1	0.16	3.8	2.7	0.06
condition_era group during day -180 through 0 days relative to index: Systolic dysfunction	5.5	2.3	0.16	4.0	3.0	0.06
condition_era group during day -180 through 0 days relative to index: Chronic systolic heart failure	3.9	1.5	0.15	2.8	1.9	0.06
condition_era group during day -180 through 0 days relative to index: Chronic congestive heart failure	2.8	0.9	0.14	2.0	1.3	0.06
condition_era group during day -180 through 0 days relative to index: Cardiomyopathy	3.0	1.0	0.14	2.1	1.4	0.05
condition_era group during day -180 through 0 days relative to index: Myocardial disease	6.6	3.1	0.16	4.9	3.8	0.05
condition_era group during day -180 through 0 days relative to index: Ischemic myocardial dysfunction	1.7	0.5	0.12	1.2	0.7	0.05
condition_era group during day -180 through 0 days relative to index: Generalized ischemic myocardial dysfunction	1.7	0.5	0.12	1.2	0.7	0.05

(Continued)	T (%)	C (%)	SDf	T (%)	C (%)	SDf
condition_era group during day -180 through 0 days relative to index: Myocardial dysfunction	1.7	0.5	0.12	1.2	0.7	0.05
condition_era group during day -180 through 0 days relative to index: Abnormal cardiovascular function	6.7	3.5	0.14	5.3	4.2	0.05

Supplementary Table 124: Most extreme patient characteristics balance diagnostics after matching for ACE +combo vs CCB/THZ +combo in the VA-OMOP data source.

	Before matching			After matching		
Characteristic (total count = 11387)	T (%)	C (%)	SDf	T (%)	C (%)	SDf
condition_era group during day -180 through 0 days relative to index: Hypertrophic cardiomyopathy	0.1	0.1	0.01	0.1	0.1	-0.02
race = Black or African American	12.3	28.7	-0.41	14.6	14.0	0.02
race = White	79.2	63.2	0.36	76.6	77.2	-0.02
Diabetes Comorbidity Severity Index (DCSI)	345.6	311.4	0.12	318.3	322.6	-0.01
CHADS2	232.8	216.3	0.14	222.3	223.9	-0.01
Charlson index - Romano adaptation	394.5	366.0	0.09	368.0	371.8	-0.01
CHADS2VASc	313.1	295.7	0.12	301.4	303.1	-0.01
condition_era group during day -180 through 0 days relative to index: Chronic kidney disease stage 5	0.1	0.7	-0.09	0.1	0.2	-0.01
condition_era group during day -180 through 0 days relative to index: Chronic kidney disease stage 4	0.5	1.9	-0.13	0.6	0.7	-0.01
drug_era group during day -180 through 0 days relative to index: iron sucrose	0.2	0.3	-0.03	0.1	0.2	-0.01

Supplementary Figure 31: Preference score distributions, propensity score stratified (middle column) and matched (right column) covariate balance and negative control error distributions (bottom row) for ACE mono vs CCB mono in the VA-OMOP data source.

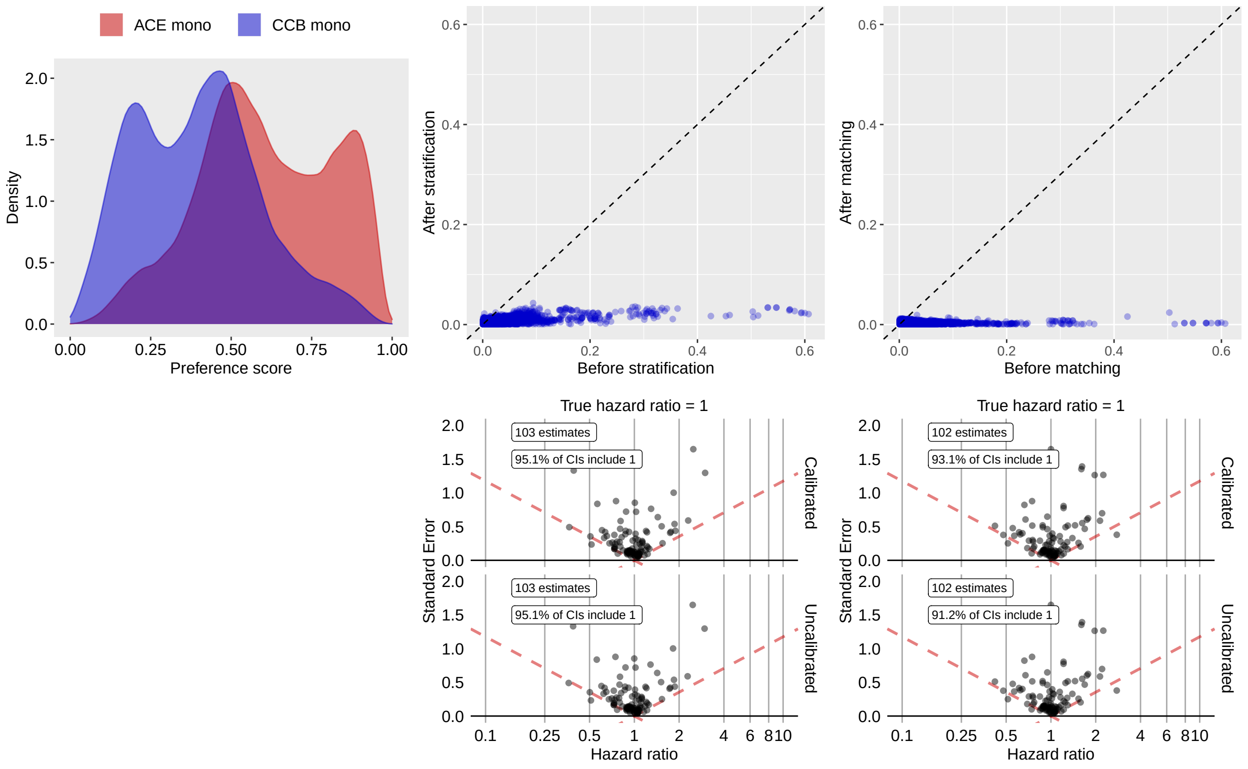

Supplementary Table 125: Most extreme patient characteristics balance diagnostics after stratification for ACE mono vs CCB mono in the VA-OMOP data source.

Characteristic (total count = 11628)	Before stratification			After stratification		
	T (%)	C (%)	SDf	T (%)	C (%)	SDf
condition_era group during day -180 through 0 days relative to index: End-stage renal disease	0.1	0.6	-0.09	0.1	0.3	-0.04
drug_era group during day -30 through 0 days relative to index: Simvastatin	15.8	6.9	0.28	12.6	11.5	0.04
drug_era group during day 0 through 0 days relative to index: Simvastatin	15.1	6.5	0.28	12.1	10.9	0.04
condition_era group during day -180 through 0 days relative to index: Renal failure syndrome	0.8	2.0	-0.10	1.1	1.4	-0.03
drug_era group during day -30 through 0 days relative to index: Metformin	29.0	8.4	0.55	21.6	20.2	0.03
drug_era group during day 0 through 0 days relative to index: Metformin	27.4	7.9	0.53	20.4	19.1	0.03
drug_era group during day -180 through 0 days relative to index: Simvastatin	17.3	7.8	0.29	13.9	12.8	0.03
drug_era group during day -30 through 0 days relative to index: Biguanides	29.0	8.4	0.55	21.6	20.2	0.03

(Continued)	T (%)	C (%)	SDf	T (%)	C (%)	SDf
drug_era group during day 0 through 0 days relative to index: Biguanides	27.4	7.9	0.53	20.4	19.1	0.03
drug_era group during day -30 through 0 days relative to index: Glucagon-like peptide-1 (GLP-1) analogues	2.2	0.4	0.15	1.6	1.2	0.03

Supplementary Table 126: Most extreme patient characteristics balance diagnostics after matching for ACE mono vs CCB mono in the VA-OMOP data source.

	Before matching			After matching		
Characteristic (total count = 11111)	T (%)	C (%)	SDf	T (%)	C (%)	SDf
race = Black or African American	12.6	33.1	-0.50	20.8	19.9	0.02
condition_era group during day -180 through 0 days relative to index: Transplanted liver present	0.1	0.2	-0.03	0.1	0.1	-0.02
race = White	78.0	58.7	0.42	70.0	70.7	-0.02
CHADS2VASc	266.4	254.8	0.09	257.5	255.7	0.01
condition_era group during day -180 through 0 days relative to index: End-stage renal disease	0.1	0.6	-0.09	0.1	0.2	-0.01
measurement during day -180 through 0 days relative to index: Gonadotropin, chorionic (hCG); quantitative	0.1	0.1	-0.02	0.1	0.1	-0.01
measurement during day -30 through 0 days relative to index: Drugs identified in Urine by Screen method	0.1	0.1	0.00	0.1	0.1	0.01
condition_era group during day -180 through 0 days relative to index: Isolated proteinuria	0.1	0.1	0.02	0.1	0.1	0.01
measurement during day -180 through 0 days relative to index: Mitogen stimulated gamma interferon [Units/volume] corrected for background in Blood	0.1	0.1	0.00	0.1	0.1	0.01
procedure_occurrence during day -180 through 0 days relative to index: Nerve conduction studies; 13 or more studies	0.1	0.1	-0.01	0.1	0.1	-0.01

Supplementary Figure 32: Preference score distributions, propensity score stratified (middle column) and matched (right column) covariate balance and negative control error distributions (bottom row) for ACE +combo vs CCB +combo in the VA-OMOP data source.

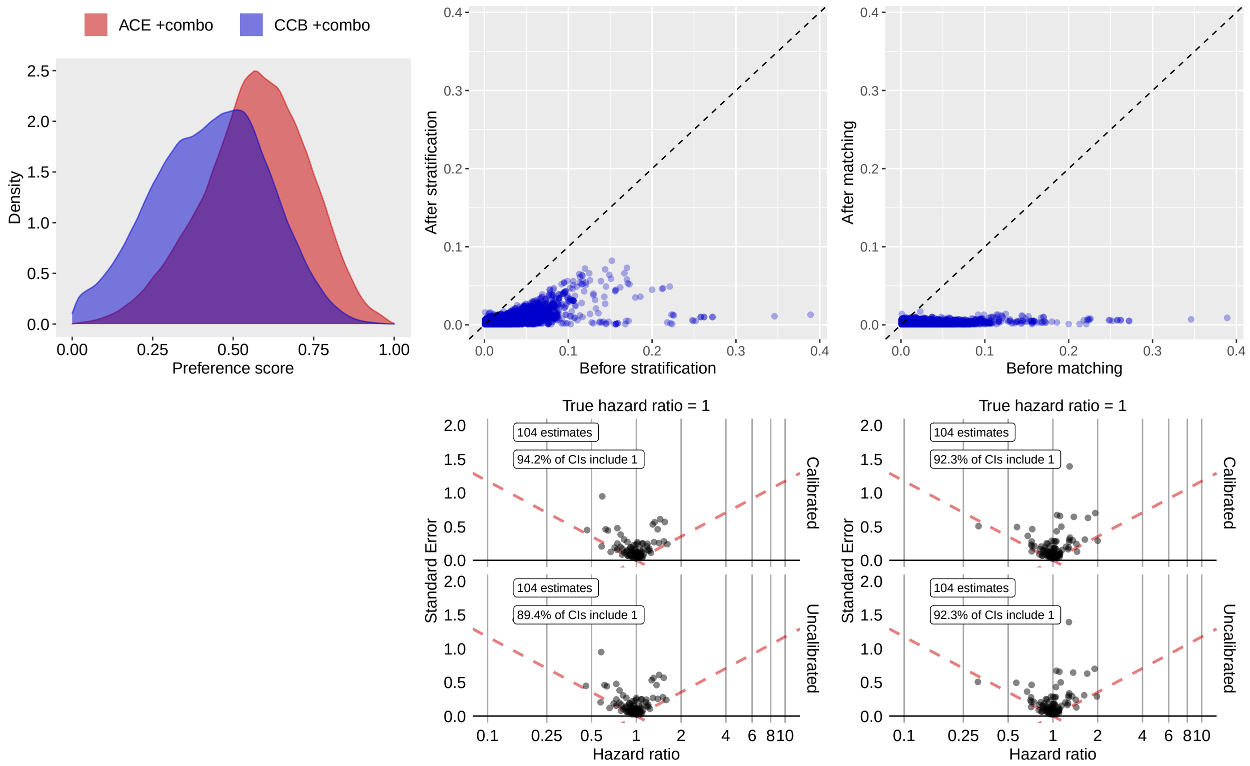

Supplementary Table 127: Most extreme patient characteristics balance diagnostics after stratification for ACE +combo vs CCB +combo in the VA-OMOP data source.

	Before stratification			After stratification		
Characteristic (total count = 12425)	T (%)	C (%)	SDf	T (%)	C (%)	SDf
condition_era group during day -180 through 0 days relative to index: End-stage renal disease	0.2	1.6	-0.15	0.3	1.0	-0.08
condition_era group during day -180 through 0 days relative to index: Chronic kidney disease stage 4	0.5	2.5	-0.17	0.8	1.5	-0.07
condition_era group during day -180 through 0 days relative to index: Chronic kidney disease stage 5	0.1	0.9	-0.12	0.1	0.5	-0.07
drug_era group during day -180 through 0 days relative to index: Drugs for treatment of hyperkalemia and hyperphosphatemia	0.4	1.9	-0.14	0.5	1.2	-0.07
drug_era group during day -30 through 0 days relative to index: Drugs for treatment of hyperkalemia and hyperphosphatemia	0.2	1.3	-0.12	0.3	0.8	-0.07
measurement above normal range during day -180 through 0 days relative to index: Parathyrin.intact [Mass/volume] in Serum or Plasma	0.9	3.3	-0.17	1.3	2.1	-0.07
condition_era group during day -180 through 0 days relative to index: Hypertensive renal failure	0.1	0.9	-0.11	0.1	0.5	-0.07
drug_era group during day -180 through 0 days relative to index: sevelamer	0.1	0.9	-0.12	0.2	0.6	-0.06

(Continued)	T (%)	C (%)	SDf	T (%)	C (%)	SDf
condition_era group during day -180 through 0 days relative to index: Anemia in chronic kidney disease	0.6	2.4	-0.15	0.8	1.5	-0.06
condition_era group during day -30 through 0 days relative to index: End-stage renal disease	0.1	0.9	-0.12	0.2	0.6	-0.06

Supplementary Table 128: Most extreme patient characteristics balance diagnostics after matching for ACE +combo vs CCB +combo in the VA-OMOP data source.

	Before matching			After matching		
Characteristic (total count = 11424)	T (%)	C (%)	SDf	T (%)	C (%)	SDf
condition_era group during day -180 through 0 days relative to index: Cough variant asthma	0.1	0.1	-0.02	0.1	0.1	-0.02
condition_era group during day -180 through 0 days relative to index: Hypertrophic cardiomyopathy	0.1	0.1	-0.00	0.1	0.1	-0.01
drug_era group during day -30 through 0 days relative to index: Drugs for treatment of hyperkalemia and hyperphosphatemia	0.2	1.3	-0.12	0.3	0.4	-0.01
drug_era group during day -180 through 0 days relative to index: sevelamer	0.1	0.9	-0.12	0.2	0.2	-0.01
drug_era group during day -30 through 0 days relative to index: sevelamer	0.1	0.7	-0.10	0.1	0.2	-0.01
drug_era group during day 0 through 0 days relative to index: sevelamer	0.1	0.6	-0.09	0.1	0.1	-0.01
drug_era group during day 0 through 0 days relative to index: Drugs for treatment of hyperkalemia and hyperphosphatemia	0.1	1.0	-0.11	0.2	0.3	-0.01
condition_era group during day -180 through 0 days relative to index: Chronic kidney disease stage 5	0.1	0.9	-0.12	0.1	0.2	-0.01
drug_era group during day -180 through 0 days relative to index: Drugs for treatment of hyperkalemia and hyperphosphatemia	0.4	1.9	-0.14	0.6	0.7	-0.01
condition_era group during day -180 through 0 days relative to index: End-stage renal disease	0.2	1.6	-0.15	0.3	0.4	-0.01

Supplementary Figure 33: Preference score distributions, propensity score stratified (middle column) and matched (right column) covariate balance and negative control error distributions (bottom row) for ACE mono vs THZ mono in the VA-OMOP data source.

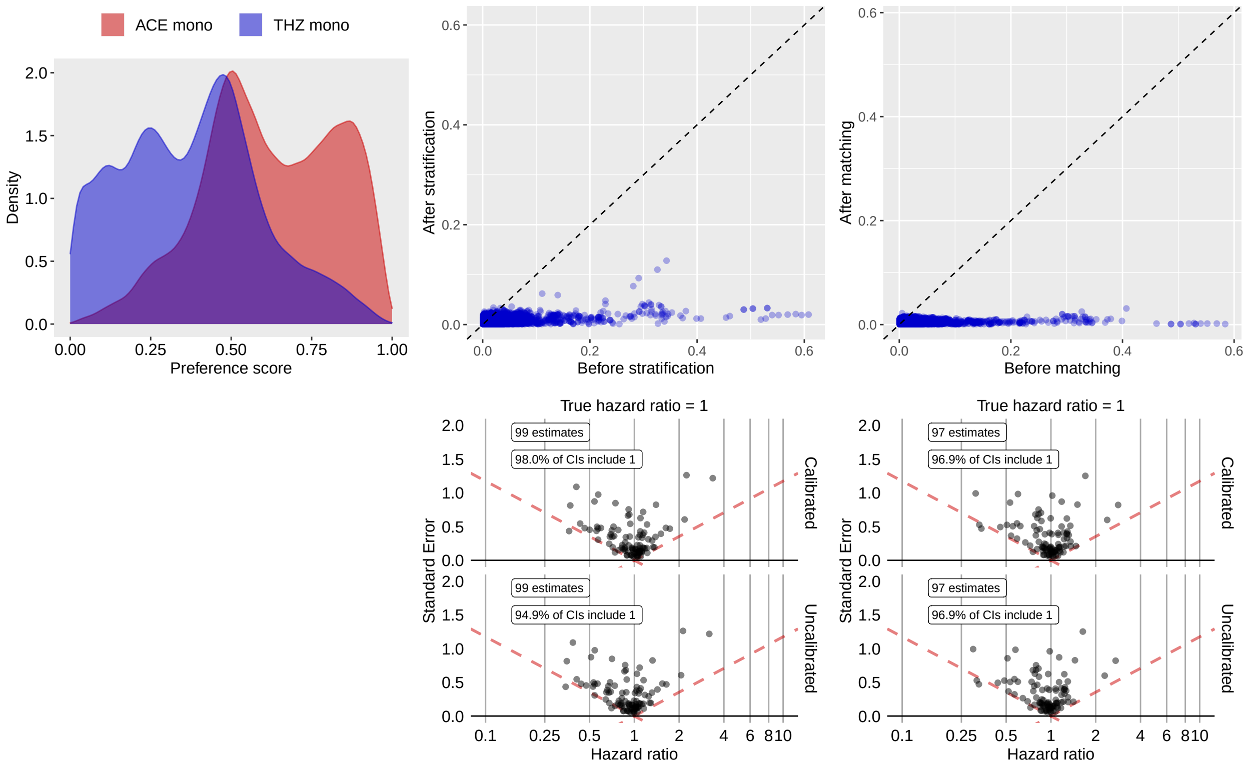

Supplementary Table 129: Most extreme patient characteristics balance diagnostics after stratification for ACE mono vs THZ mono in the VA-OMOP data source.

	Before stratification			After stratification		
Characteristic (total count = 11125)	T (%)	C (%)	SDf	T (%)	C (%)	SDf
drug_era group during day 0 through 0 days relative to index: Potassium Chloride	1.4	8.8	-0.34	1.8	3.9	-0.13
drug_era group during day -30 through 0 days relative to index: Potassium Chloride	2.5	10.4	-0.33	2.9	5.1	-0.11
measurement below normal range during day -180 through 0 days relative to index: Potassium [Moles/volume] in Serum or Plasma	2.2	8.7	-0.29	2.7	4.4	-0.09
drug_era group during day -180 through 0 days relative to index: Potassium Chloride	7.0	15.8	-0.28	7.6	9.7	-0.08
condition_era group during day -180 through 0 days relative to index: Potassium disorder	0.9	2.2	-0.11	0.9	1.6	-0.06
condition_era group during day -180 through 0 days relative to index: Hypokalemia	0.4	1.9	-0.14	0.5	1.1	-0.06
measurement above normal range during day -180 through 0 days relative to index: Carbon dioxide, total [Moles/volume] in Serum or Plasma	2.1	6.8	-0.23	2.7	3.5	-0.05

(Continued)	T (%)	C (%)	SDf	T (%)	C (%)	SDf
drug_era group during day -30 through 0 days relative to index: HMG CoA reductase inhibitors	58.4	43.1	0.31	55.3	53.1	0.04
CHADS2VASc	266.4	238.4	0.23	260.7	266.0	-0.04
drug_era group during day 0 through 0 days relative to index: HMG CoA reductase inhibitors	55.8	41.0	0.30	52.8	50.7	0.04

Supplementary Table 130: Most extreme patient characteristics balance diagnostics after matching for ACE mono vs THZ mono in the VA-OMOP data source.

	Before matching			After matching		
Characteristic (total count = 10789)	T (%)	C (%)	SDf	T (%)	C (%)	SDf
race = Black or African American	12.6	28.8	-0.41	25.7	24.4	0.03
race = White	78.0	63.3	0.33	66.1	67.3	-0.03
condition_era group during day -180 through 0 days relative to index: Corneal edema	0.1	0.1	0.01	0.1	0.1	0.02
gender = MALE	95.3	87.1	0.29	89.8	90.4	-0.02
gender = FEMALE	4.7	12.9	-0.29	10.2	9.6	0.02
condition_era group during day -30 through 0 days relative to index: Gastrointestinal obstruction	0.1	0.1	0.01	0.1	0.1	0.02
measurement below normal range during day -180 through 0 days relative to index: Anion gap 4 in Serum or Plasma	0.2	0.1	0.02	0.1	0.1	0.02
observation during day -180 through 0 days relative to index: Underweight	0.1	0.1	0.01	0.2	0.1	0.02
measurement during day -30 through 0 days relative to index: Lactate (lactic acid)	0.5	0.3	0.03	0.3	0.3	0.02
measurement during day -30 through 0 days relative to index: Lactate [Moles/volume] in Serum or Plasma	0.5	0.3	0.03	0.3	0.3	0.02

Supplementary Figure 34: Preference score distributions, propensity score stratified (middle column) and matched (right column) covariate balance and negative control error distributions (bottom row) for ACE +combo vs THZ +combo in the VA-OMOP data source.

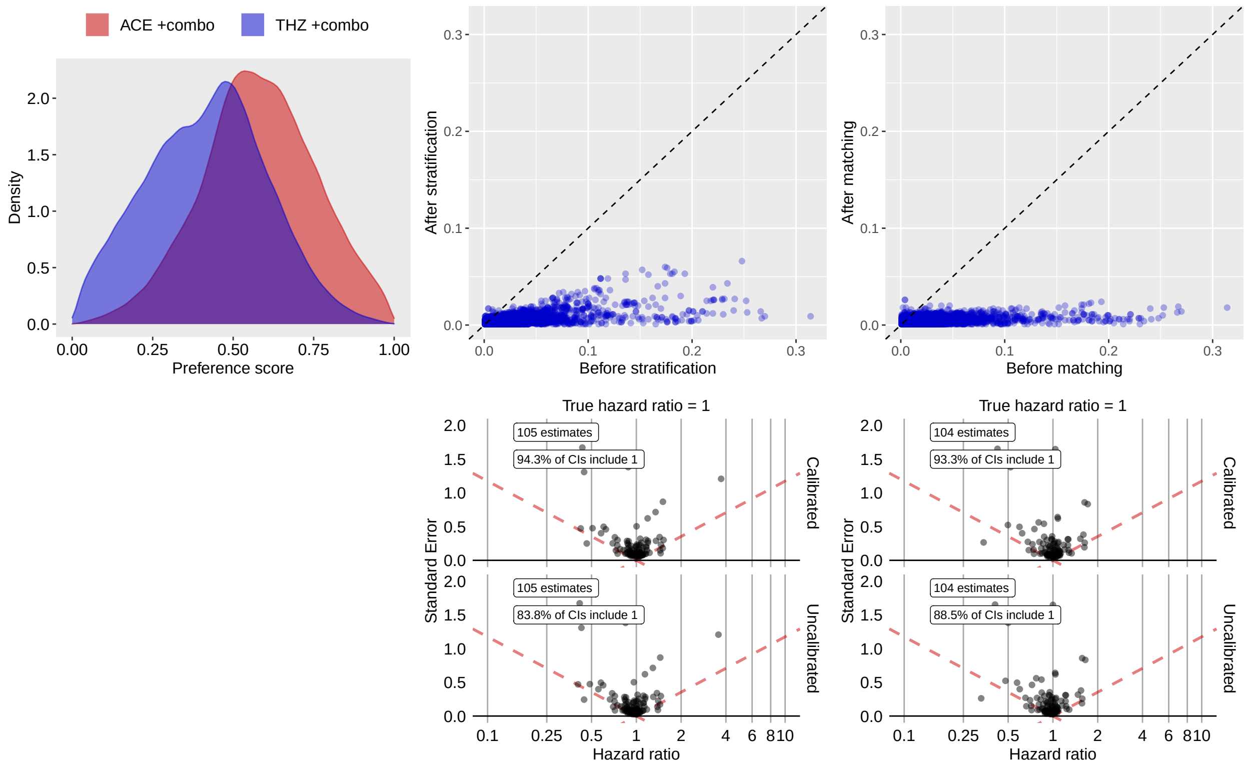

Supplementary Table 131: Most extreme patient characteristics balance diagnostics after stratification for ACE +combo vs THZ +combo in the VA-OMOP data source.

	Before stratification			After stratification		
Characteristic (total count = 11758)	T (%)	C (%)	SDf	T (%)	C (%)	SDf
measurement below normal range during day -180 through 0 days relative to index: Potassium [Moles/volume] in Serum or Plasma	4.3	10.9	-0.25	5.5	7.0	-0.07
condition_era group during day -180 through 0 days relative to index: Systolic heart failure	4.7	1.7	0.17	3.9	2.8	0.06
condition_era group during day -180 through 0 days relative to index: Systolic dysfunction	5.0	1.8	0.18	4.1	3.0	0.06
condition_era group during day -180 through 0 days relative to index: Chronic systolic heart failure	3.5	1.2	0.15	2.9	2.0	0.06
condition_era group during day -180 through 0 days relative to index: Abnormal cardiovascular function	6.3	2.6	0.18	5.3	4.2	0.06
condition_era group during day -180 through 0 days relative to index: Heart failure	7.8	3.4	0.19	6.6	5.3	0.05
condition_era group during day -180 through 0 days relative to index: Congestive heart failure	6.5	2.7	0.18	5.5	4.3	0.05
condition_era group during day -180 through 0 days relative to index: Chronic congestive heart failure	2.5	0.7	0.14	2.0	1.3	0.05

(Continued)	T (%)	C (%)	SDf	T (%)	C (%)	SDf
condition_era group during day -180 through 0 days relative to index: Myocardial disease	6.1	2.5	0.18	5.1	4.0	0.05
condition_era group during day -180 through 0 days relative to index: Chronic heart failure	4.7	1.9	0.16	3.9	3.0	0.05

Supplementary Table 132: Most extreme patient characteristics balance diagnostics after matching for ACE +combo vs THZ +combo in the VA-OMOP data source.

	Before matching			After matching		
Characteristic (total count = 11688)	T (%)	C (%)	SDf	T (%)	C (%)	SDf
condition_era group during day -180 through 0 days relative to index: Hyperaldosteronism	0.1	0.1	0.00	0.1	0.1	0.03
condition_era group during day -180 through 0 days relative to index: Aldosterone disorder	0.1	0.1	0.00	0.1	0.1	0.03
condition_era group during day -180 through 0 days relative to index: Heart failure	7.8	3.4	0.19	3.9	3.4	0.02
condition_era group during day -180 through 0 days relative to index: Abnormal cardiovascular function	6.3	2.6	0.18	3.0	2.6	0.02
condition_era group during day -180 through 0 days relative to index: Congestive heart failure	6.5	2.7	0.18	3.1	2.8	0.02
condition_era group during day -180 through 0 days relative to index: Systolic dysfunction	5.0	1.8	0.18	2.1	1.8	0.02
condition_era group during day -180 through 0 days relative to index: Systolic heart failure	4.7	1.7	0.17	2.0	1.7	0.02
condition_era group during day -180 through 0 days relative to index: Kidney disease	14.4	12.8	0.05	13.3	12.6	0.02
condition_era group during day -180 through 0 days relative to index: Chronic heart failure	4.7	1.9	0.16	2.1	1.9	0.02
condition_era group during day -180 through 0 days relative to index: Renal impairment	11.0	9.8	0.04	10.2	9.6	0.02

Supplementary Figure 35: Preference score distributions, propensity score stratified (middle column) and matched (right column) covariate balance and negative control error distributions (bottom row) for ARB mono vs CCB/THZ mono in the VA-OMOP data source.

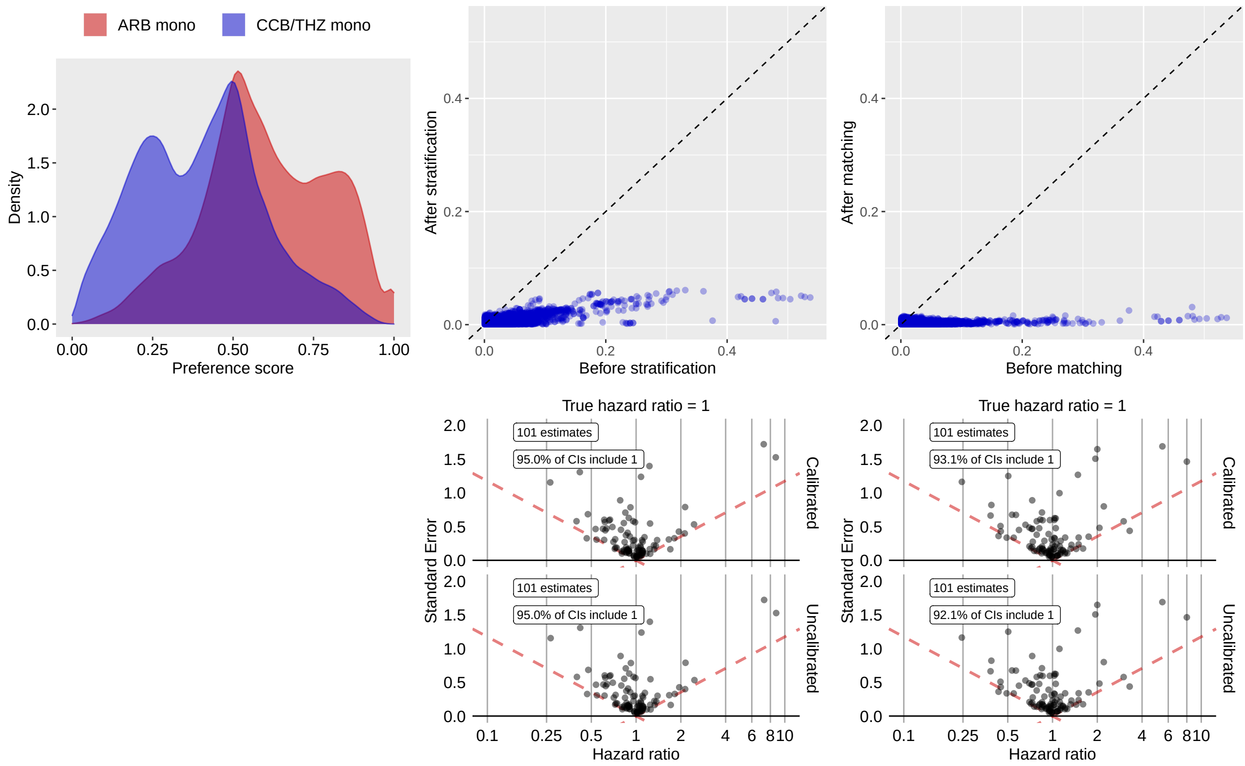

Supplementary Table 133: Most extreme patient characteristics balance diagnostics after stratification for ARB mono vs CCB/THZ mono in the VA-OMOP data source.

Characteristic (total count = 11392)	Before stratification			After stratification		
	T (%)	C (%)	SDf	T (%)	C (%)	SDf
drug_era group during day -180 through 0 days relative to index: INSULINS AND ANALOGUES	14.0	4.6	0.33	7.9	6.4	0.06
drug_era group during day -30 through 0 days relative to index: INSULINS AND ANALOGUES	12.3	3.8	0.32	6.8	5.4	0.06
condition_era group during day -180 through 0 days relative to index: Diabetic complication	21.0	8.4	0.36	13.0	11.0	0.06
drug_era group during day 0 through 0 days relative to index: INSULINS AND ANALOGUES	11.2	3.4	0.30	6.2	4.8	0.06
condition_era group during day -180 through 0 days relative to index: Diabetes mellitus	37.0	16.3	0.48	23.4	21.0	0.06
drug_era group during day -180 through 0 days relative to index: Insulins and analogues for injection, long-acting	11.5	3.6	0.30	6.4	5.1	0.06
condition_era group during day -180 through 0 days relative to index: Type 2 diabetes mellitus	36.3	16.1	0.47	23.1	20.7	0.06
drug_era group during day -180 through 0 days relative to index: Sodium-glucose co-transporter 2 (SGLT2) inhibitors	3.1	0.6	0.19	1.5	0.9	0.06

(Continued)	T (%)	C (%)	SDf	T (%)	C (%)	SDf
drug_era group during day -180 through 0 days relative to index: empagliflozin	3.1	0.6	0.19	1.5	0.9	0.06
drug_era group during day -30 through 0 days relative to index: Insulins and analogues for injection, long-acting	9.9	3.0	0.28	5.5	4.3	0.06

Supplementary Table 134: Most extreme patient characteristics balance diagnostics after matching for ARB mono vs CCB/THZ mono in the VA-OMOP data source.

	Before matching			After matching		
Characteristic (total count = 10757)	T (%)	C (%)	SDf	T (%)	C (%)	SDf
race = Black or African American	13.7	33.5	-0.48	15.3	14.2	0.03
race = White	75.9	58.6	0.38	74.7	75.7	-0.03
Charlson index - Romano adaptation	316.5	266.2	0.18	296.1	300.5	-0.02
CHADS2	208.2	179.4	0.27	200.1	201.8	-0.02
condition_era group during day -180 through 0 days relative to index: Diabetes mellitus	37.0	16.3	0.48	30.3	30.9	-0.01
condition_era group during day -180 through 0 days relative to index: Type 2 diabetes mellitus	36.3	16.1	0.47	29.8	30.5	-0.01
condition_era group during day -180 through 0 days relative to index: Chronic kidney disease stage 5	0.1	0.2	-0.03	0.1	0.1	-0.01
measurement during day -180 through 0 days relative to index: Nuclear Ab pattern [interpretation] in Serum by Immunofluorescence	0.1	0.1	-0.00	0.1	0.1	-0.01
observation during day -180 through 0 days relative to index: Screening for depression performed (DEM)	0.1	0.1	0.01	0.1	0.1	0.01
CHADS2VASc	281.5	249.5	0.25	272.8	274.7	-0.01

Supplementary Figure 36: Preference score distributions, propensity score stratified (middle column) and matched (right column) covariate balance and negative control error distributions (bottom row) for ARB +combo vs CCB/THZ +combo in the VA-OMOP data source.

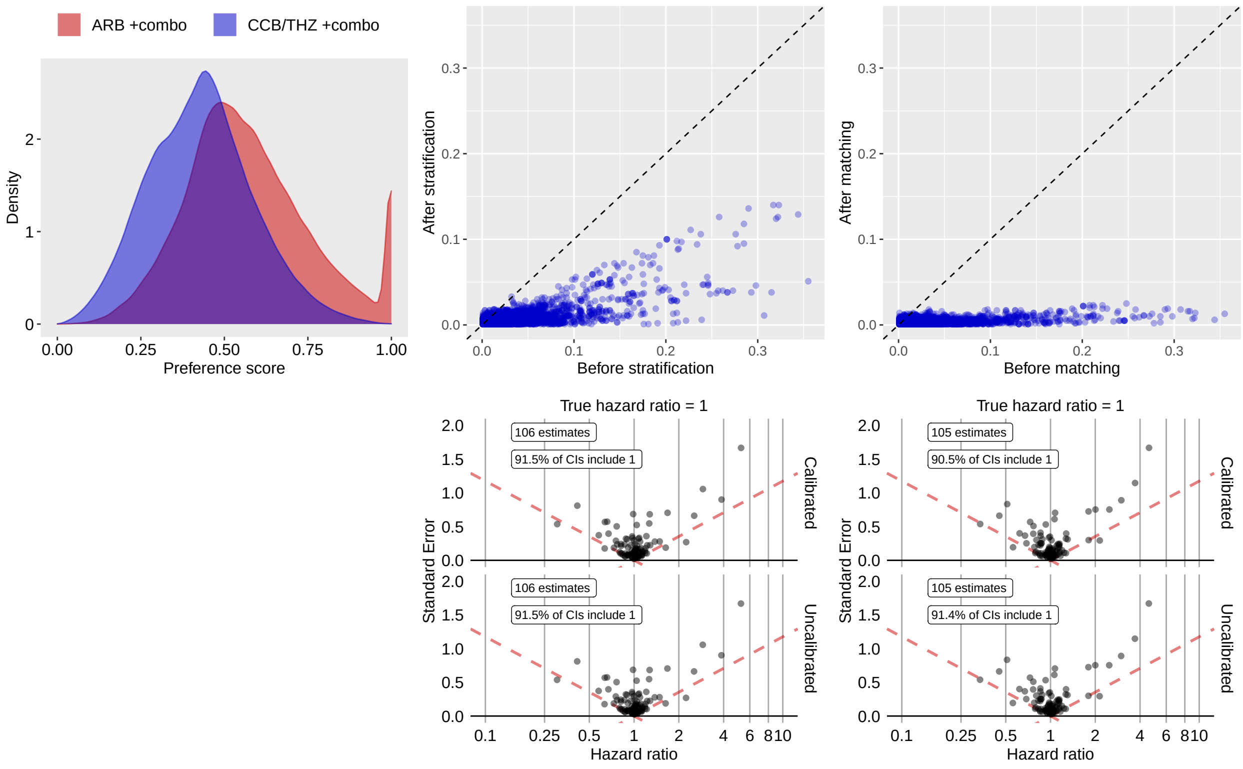

Supplementary Table 135: Most extreme patient characteristics balance diagnostics after stratification for ARB +combo vs CCB/THZ +combo in the VA-OMOP data source.

	Before stratification			After stratification		
Characteristic (total count = 12153)	T (%)	C (%)	SDf	T (%)	C (%)	SDf
condition_era group during day -180 through 0 days relative to index: Systolic dysfunction	9.3	2.0	0.32	5.0	2.4	0.14
condition_era group during day -180 through 0 days relative to index: Systolic heart failure	8.8	1.8	0.32	4.7	2.2	0.14
condition_era group during day -180 through 0 days relative to index: Chronic systolic heart failure	6.9	1.2	0.29	3.6	1.5	0.14
condition_era group during day -180 through 0 days relative to index: Heart failure	13.4	3.9	0.34	7.7	4.6	0.13
condition_era group during day -180 through 0 days relative to index: Abnormal cardiovascular function	11.1	3.0	0.32	6.2	3.5	0.13
condition_era group during day -180 through 0 days relative to index: Chronic congestive heart failure	5.1	0.8	0.26	2.7	1.0	0.13
condition_era group during day -180 through 0 days relative to index: Congestive heart failure	11.3	3.2	0.32	6.4	3.7	0.12
condition_era group during day -180 through 0 days relative to index: Chronic heart failure	8.5	2.1	0.28	4.7	2.5	0.12

(Continued)	T (%)	C (%)	SDf	T (%)	C (%)	SDf
observation during day -180 through 0 days relative to index: History of cardiovascular surgery	4.0	0.6	0.23	2.1	0.8	0.11
condition_era group during day -180 through 0 days relative to index: Cardiomyopathy	4.8	0.9	0.24	2.5	1.1	0.11

Supplementary Table 136: Most extreme patient characteristics balance diagnostics after matching for ARB +combo vs CCB/THZ +combo in the VA-OMOP data source.

	Before matching			After matching		
Characteristic (total count = 11696)	T (%)	C (%)	SDf	T (%)	C (%)	SDf
Diabetes Comorbidity Severity Index (DCSI)	380.2	305.9	0.25	366.6	374.3	-0.03
condition_era group during day -180 through 0 days relative to index: Diabetes mellitus	42.2	32.0	0.21	41.6	42.8	-0.02
condition_era group during day -180 through 0 days relative to index: Type 2 diabetes mellitus	41.6	31.6	0.21	41.0	42.2	-0.02
drug_era group during day -180 through 0 days relative to index: DRUGS USED IN DIABETES	42.9	32.2	0.22	42.6	43.7	-0.02
condition_era group during day -180 through 0 days relative to index: Ischemic myocardial dysfunction	3.0	0.4	0.20	1.5	1.2	0.02
condition_era group during day -180 through 0 days relative to index: Generalized ischemic myocardial dysfunction	3.0	0.4	0.20	1.5	1.2	0.02
condition_era group during day -180 through 0 days relative to index: Myocardial dysfunction	3.0	0.4	0.20	1.5	1.2	0.02
drug_era group during day -30 through 0 days relative to index: DRUGS USED IN DIABETES	40.2	30.1	0.21	40.0	41.0	-0.02
drug_era group during day 0 through 0 days relative to index: DRUGS USED IN DIABETES	38.6	28.8	0.21	38.4	39.4	-0.02
Charlson index - Romano adaptation	428.7	358.6	0.22	413.7	419.7	-0.02

Supplementary Figure 37: Preference score distributions, propensity score stratified (middle column) and matched (right column) covariate balance and negative control error distributions (bottom row) for ARB mono vs CCB mono in the VA-OMOP data source.

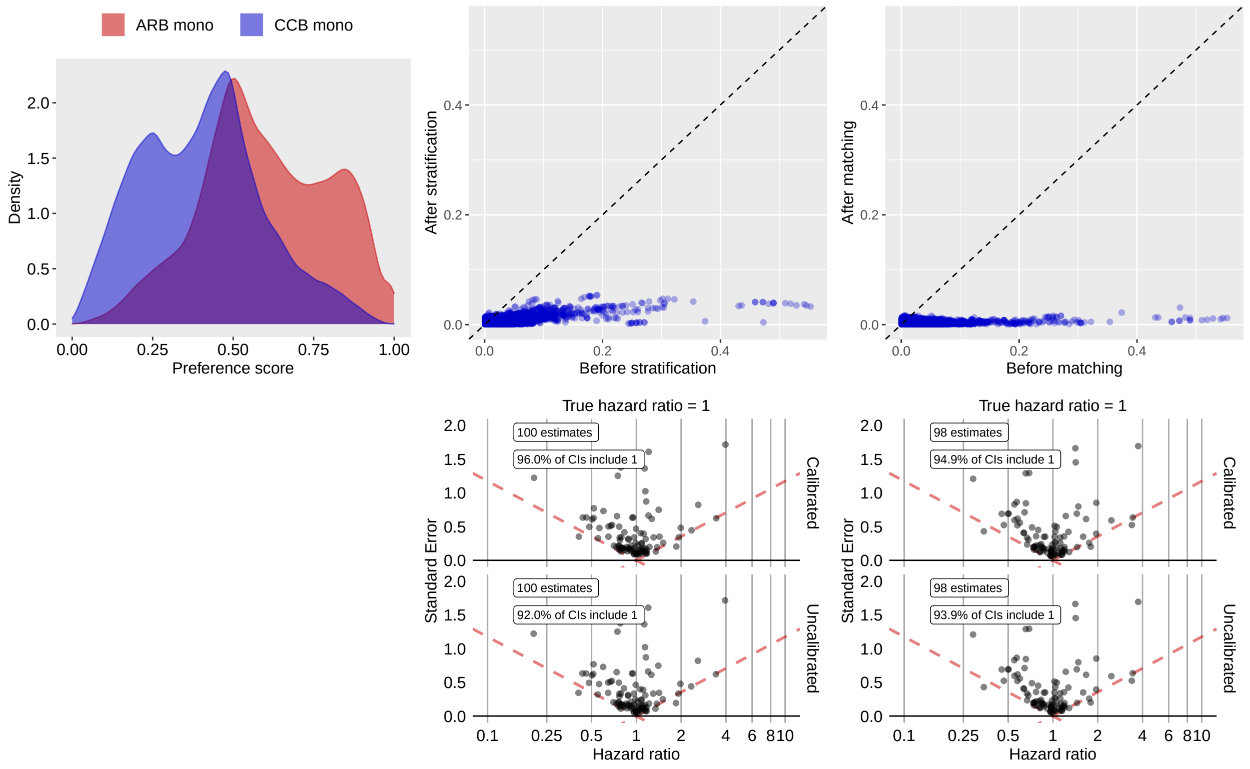

Supplementary Table 137: Most extreme patient characteristics balance diagnostics after stratification for ARB mono vs CCB mono in the VA-OMOP data source.

	Before stratification			After stratification		
Characteristic (total count = 11404)	T (%)	C (%)	SDf	T (%)	C (%)	SDf
drug_era group during day -180 through 0 days relative to index: Sodium-glucose co-transporter 2 (SGLT2) inhibitors	3.1	0.6	0.19	1.7	1.1	0.05
drug_era group during day -180 through 0 days relative to index: empagliflozin	3.1	0.6	0.19	1.7	1.1	0.05
drug_era group during day -30 through 0 days relative to index: Sodium-glucose co-transporter 2 (SGLT2) inhibitors	2.8	0.5	0.18	1.5	1.0	0.05
drug_era group during day 0 through 0 days relative to index: Sodium-glucose co-transporter 2 (SGLT2) inhibitors	2.7	0.5	0.18	1.5	0.9	0.05
drug_era group during day 0 through 0 days relative to index: empagliflozin	2.6	0.5	0.18	1.4	0.9	0.05
drug_era group during day -180 through 0 days relative to index: Glucagon-like peptide-1 (GLP-1) analogues	2.8	0.5	0.18	1.5	1.0	0.05
drug_era group during day -30 through 0 days relative to index: empagliflozin	2.8	0.5	0.18	1.5	1.0	0.05
drug_era group during day -30 through 0 days relative to index: Glucagon-like peptide-1 (GLP-1) analogues	2.5	0.4	0.17	1.3	0.8	0.05

(Continued)	T (%)	C (%)	SDf	T (%)	C (%)	SDf
drug_era group during day 0 through 0 days relative to index: Glucagon-like peptide-1 (GLP-1) analogues	2.3	0.4	0.16	1.3	0.8	0.05
drug_era group during day -30 through 0 days relative to index: INSULINS AND ANALOGUES	12.3	3.9	0.31	7.5	6.3	0.05

Supplementary Table 138: Most extreme patient characteristics balance diagnostics after matching for ARB mono vs CCB mono in the VA-OMOP data source.

	Before matching			After matching		
Characteristic (total count = 10883)	T (%)	C (%)	SDf	T (%)	C (%)	SDf
race = Black or African American	13.7	33.1	-0.47	16.5	15.3	0.03
race = White	75.9	58.7	0.37	73.6	74.6	-0.02
measurement below normal range during day -180 through 0 days relative to index: Leukocytes [#/area] in Urine sediment by Microscopy high power field	0.1	0.1	0.01	0.1	0.1	0.02
condition_era group during day -30 through 0 days relative to index: Hemangioma of skin and subcutaneous tissue	0.1	0.1	-0.01	0.1	0.1	-0.02
condition_era group during day -180 through 0 days relative to index: Chronic kidney disease stage 5	0.1	0.3	-0.05	0.1	0.1	-0.02
measurement during day -180 through 0 days relative to index: Glucose, body fluid, other than blood	0.2	0.1	0.03	0.1	0.1	0.02
drug_era group during day -180 through 0 days relative to index: LIPID MODIFYING AGENTS	63.0	49.6	0.27	57.4	58.1	-0.02
drug_era group during day -180 through 0 days relative to index: LIPID MODIFYING AGENTS, PLAIN	63.0	49.6	0.27	57.4	58.1	-0.02
drug_era group during day -180 through 0 days relative to index: HMG CoA reductase inhibitors	60.9	48.2	0.26	55.5	56.2	-0.02
drug_era group during day -30 through 0 days relative to index: Second-generation cephalosporins	0.1	0.1	0.01	0.1	0.1	0.02

Supplementary Figure 38: Preference score distributions, propensity score stratified (middle column) and matched (right column) covariate balance and negative control error distributions (bottom row) for ARB +combo vs CCB +combo in the VA-OMOP data source.

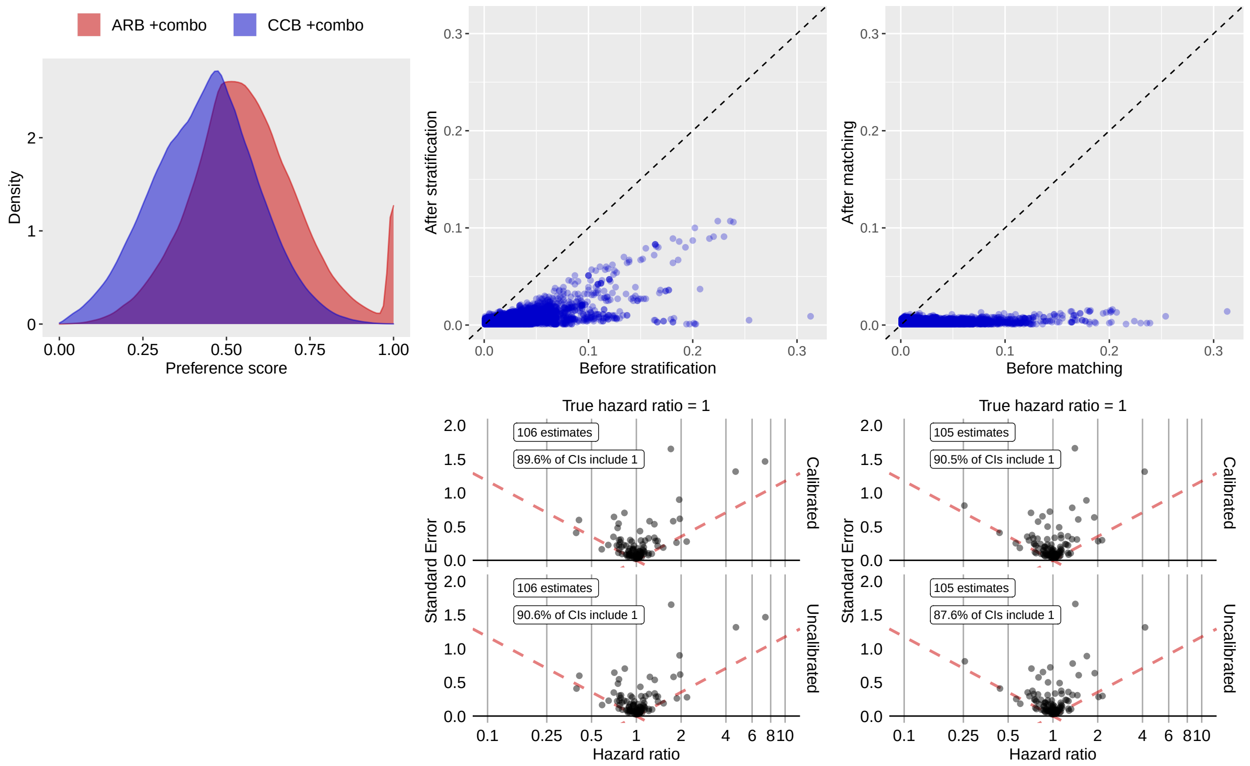

Supplementary Table 139: Most extreme patient characteristics balance diagnostics after stratification for ARB +combo vs CCB +combo in the VA-OMOP data source.

	Before stratification			After stratification		
Characteristic (total count = 12193)	T (%)	C (%)	SDf	T (%)	C (%)	SDf
condition_era group during day -180 through 0 days relative to index: Systolic heart failure	7.0	2.1	0.24	4.6	2.6	0.11
condition_era group during day -180 through 0 days relative to index: Chronic systolic heart failure	5.5	1.4	0.22	3.5	1.8	0.11
condition_era group during day -180 through 0 days relative to index: Systolic dysfunction	7.4	2.3	0.24	4.9	2.9	0.11
condition_era group during day -180 through 0 days relative to index: Chronic congestive heart failure	4.0	0.9	0.20	2.5	1.2	0.10
condition_era group during day -180 through 0 days relative to index: Abnormal cardiovascular function	8.9	3.6	0.22	6.3	4.2	0.09
condition_era group during day -180 through 0 days relative to index: Heart failure	10.8	4.7	0.23	7.7	5.5	0.09
condition_era group during day -180 through 0 days relative to index: Congestive heart failure	9.1	3.8	0.22	6.5	4.5	0.09
observation during day -180 through 0 days relative to index: History of cardiovascular surgery	3.2	0.7	0.18	1.9	0.9	0.09

(Continued)	T (%)	C (%)	SDf	T (%)	C (%)	SDf
condition_era group during day -180 through 0 days relative to index: Chronic heart failure	6.7	2.6	0.20	4.7	3.0	0.09
condition_era group during day -180 through 0 days relative to index: Cardiomyopathy	3.8	1.0	0.19	2.4	1.3	0.09

Supplementary Table 140: Most extreme patient characteristics balance diagnostics after matching for ARB +combo vs CCB +combo in the VA-OMOP data source.

	Before matching			After matching		
Characteristic (total count = 11490)	T (%)	C (%)	SDf	T (%)	C (%)	SDf
drug_era group during day -180 through 0 days relative to index: DRUGS USED IN DIABETES	42.6	32.8	0.20	40.5	41.2	-0.02
condition_era group during day -180 through 0 days relative to index: Diabetes mellitus	41.6	32.9	0.18	39.3	40.1	-0.01
condition_era group during day -180 through 0 days relative to index: Type 2 diabetes mellitus	41.1	32.4	0.18	38.8	39.5	-0.01
drug_era group during day -30 through 0 days relative to index: DRUGS USED IN DIABETES	40.0	30.4	0.20	38.0	38.7	-0.01
race = Black or African American	16.6	29.6	-0.31	17.3	16.8	0.01
drug_era group during day -180 through 0 days relative to index: Metformin	30.5	22.5	0.18	29.2	29.9	-0.01
condition_era group during day -180 through 0 days relative to index: Diabetes mellitus without complication	34.1	26.6	0.16	32.2	32.9	-0.01
condition_era group during day -180 through 0 days relative to index: Type 2 diabetes mellitus without complication	33.8	26.3	0.16	31.9	32.6	-0.01
drug_era group during day -180 through 0 days relative to index: BLOOD GLUCOSE LOWERING DRUGS, EXCL. INSULINS	37.3	27.9	0.20	35.6	36.2	-0.01
drug_era group during day -180 through 0 days relative to index: Biguanides	30.5	22.5	0.18	29.2	29.9	-0.01

Supplementary Figure 39: Preference score distributions, propensity score stratified (middle column) and matched (right column) covariate balance and negative control error distributions (bottom row) for ARB mono vs THZ mono in the VA-OMOP data source.

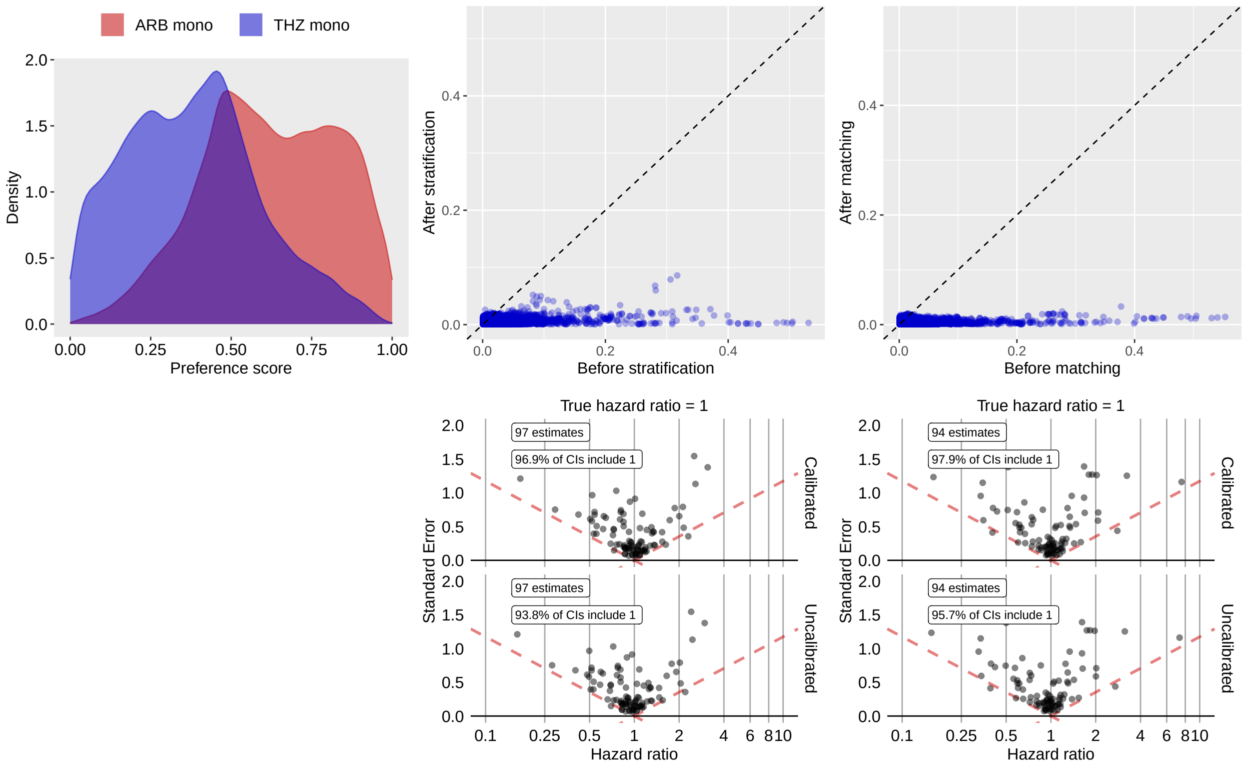

Supplementary Table 141: Most extreme patient characteristics balance diagnostics after stratification for ARB mono vs THZ mono in the VA-OMOP data source.

	Before stratification			After stratification		
Characteristic (total count = 10907)	T (%)	C (%)	SDf	T (%)	C (%)	SDf
drug_era group during day 0 through 0 days relative to index: Potassium Chloride	1.8	8.8	-0.32	3.3	5.0	-0.09
drug_era group during day -30 through 0 days relative to index: Potassium Chloride	2.8	10.4	-0.31	4.5	6.2	-0.08
measurement below normal range during day -180 through 0 days relative to index: Potassium [Moles/volume] in Serum or Plasma	2.3	8.7	-0.28	4.0	5.4	-0.07
drug_era group during day -180 through 0 days relative to index: Potassium Chloride	7.0	15.8	-0.28	9.1	10.9	-0.06
condition_era group during day -180 through 0 days relative to index: Chronic congestive heart failure	0.5	0.1	0.08	0.4	0.1	0.05
condition_era group during day -180 through 0 days relative to index: Systolic heart failure	0.9	0.2	0.10	0.6	0.3	0.05
condition_era group during day -180 through 0 days relative to index: Chronic systolic heart failure	0.6	0.1	0.09	0.4	0.2	0.05
condition_era group during day -180 through 0 days relative to index: Systolic dysfunction	0.9	0.2	0.09	0.7	0.4	0.04

(Continued)	T (%)	C (%)	SDf	T (%)	C (%)	SDf
condition_era group during day -180 through 0 days relative to index: Heart failure	1.8	0.6	0.11	1.4	0.9	0.04
condition_era group during day -180 through 0 days relative to index: Chronic heart failure	0.8	0.2	0.08	0.6	0.3	0.04

Supplementary Table 142: Most extreme patient characteristics balance diagnostics after matching for ARB mono vs THZ mono in the VA-OMOP data source.

	Before matching			After matching		
Characteristic (total count = 10705)	T (%)	C (%)	SDf	T (%)	C (%)	SDf
race = Black or African American	13.7	28.8	-0.38	19.6	18.2	0.03
race = White	75.9	63.3	0.28	71.5	72.6	-0.02
observation during day -180 through 0 days relative to index: Allergy to drug	0.2	0.1	0.02	0.1	0.1	0.02
condition_era group during day -30 through 0 days relative to index: Phobia	0.1	0.1	-0.01	0.1	0.2	-0.02
condition_era group during day -30 through 0 days relative to index: Anxiety state	0.1	0.1	-0.01	0.1	0.2	-0.02
drug_era group during day -30 through 0 days relative to index: CARDIOVASCULAR SYSTEM	59.5	46.6	0.26	49.0	50.0	-0.02
drug_era group during day -30 through 0 days relative to index: LIPID MODIFYING AGENTS	57.5	44.5	0.26	47.0	48.0	-0.02
drug_era group during day -30 through 0 days relative to index: LIPID MODIFYING AGENTS, PLAIN	57.5	44.5	0.26	47.0	48.0	-0.02
condition_era group during day -30 through 0 days relative to index: Phobic disorder	0.1	0.1	-0.01	0.1	0.1	-0.02
drug_era group during day 0 through 0 days relative to index: CARDIOVASCULAR SYSTEM	56.8	44.1	0.26	46.5	47.4	-0.02

Supplementary Figure 40: Preference score distributions, propensity score stratified (middle column) and matched (right column) covariate balance and negative control error distributions (bottom row) for ARB +combo vs THZ +combo in the VA-OMOP data source.

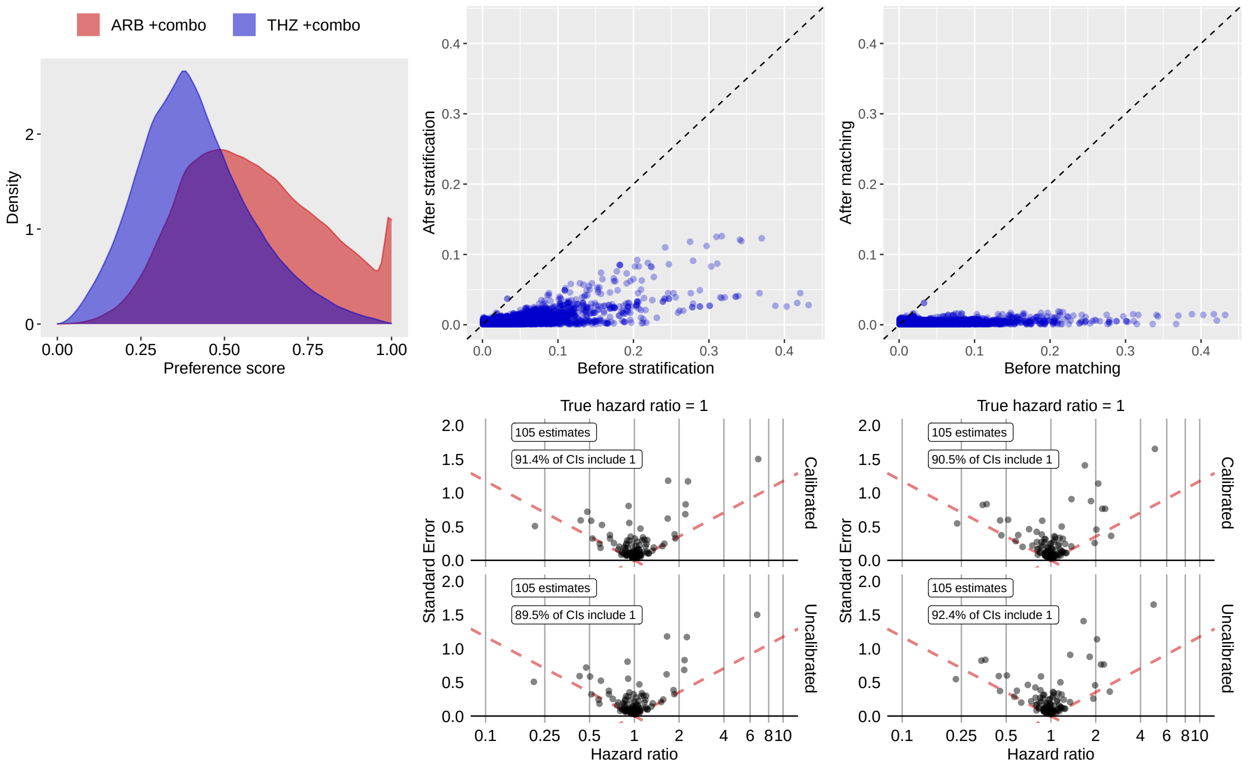

Supplementary Table 143: Most extreme patient characteristics balance diagnostics after stratification for ARB +combo vs THZ +combo in the VA-OMOP data source.

	Before stratification			After stratification		
Characteristic (total count = 11673)	T (%)	C (%)	SDf	T (%)	C (%)	SDf
condition_era group during day -180 through 0 days relative to index: Systolic dysfunction	8.0	1.4	0.32	4.7	2.4	0.13
condition_era group during day -180 through 0 days relative to index: Systolic heart failure	7.5	1.2	0.31	4.4	2.2	0.12
condition_era group during day -180 through 0 days relative to index: Heart failure	12.1	2.6	0.37	7.2	4.4	0.12
condition_era group during day -180 through 0 days relative to index: Abnormal cardiovascular function	9.9	2.0	0.34	5.9	3.3	0.12
condition_era group during day -180 through 0 days relative to index: Congestive heart failure	10.2	2.1	0.34	6.1	3.5	0.12
condition_era group during day -180 through 0 days relative to index: Chronic systolic heart failure	5.8	0.9	0.28	3.3	1.5	0.12
condition_era group during day -180 through 0 days relative to index: Chronic heart failure	7.5	1.4	0.30	4.4	2.4	0.11
condition_era group during day -180 through 0 days relative to index: Chronic congestive heart failure	4.2	0.6	0.24	2.4	1.0	0.11

(Continued)	T (%)	C (%)	SDf	T (%)	C (%)	SDf
observation during day -180 through 0 days relative to index: History of cardiovascular surgery	3.2	0.5	0.20	1.8	0.8	0.09
condition_era group during day -180 through 0 days relative to index: Myocardial disease	8.3	2.2	0.28	5.1	3.3	0.09

Supplementary Table 144: Most extreme patient characteristics balance diagnostics after matching for ARB +combo vs THZ +combo in the VA-OMOP data source.

	Before matching			After matching		
Characteristic (total count = 11329)	T (%)	C (%)	SDf	T (%)	C (%)	SDf
condition_era group during day -180 through 0 days relative to index: Hyperaldosteronism	0.2	0.1	0.03	0.2	0.1	0.03
condition_era group during day -180 through 0 days relative to index: Aldosterone disorder	0.2	0.1	0.03	0.2	0.1	0.03
drug_era group during day -180 through 0 days relative to index: DRUGS USED IN DIABETES	43.9	33.6	0.21	40.4	41.3	-0.02
condition_era group during day -180 through 0 days relative to index: Diabetes mellitus	43.1	32.7	0.22	39.0	39.9	-0.02
condition_era group during day -180 through 0 days relative to index: Type 2 diabetes mellitus	42.5	32.3	0.21	38.5	39.4	-0.02
drug_era group during day -30 through 0 days relative to index: DRUGS USED IN DIABETES	41.2	31.7	0.20	38.1	38.9	-0.02
drug_era group during day 0 through 0 days relative to index: DRUGS USED IN DIABETES	39.5	30.6	0.19	36.7	37.5	-0.02
drug_era group during day -180 through 0 days relative to index: BLOOD GLUCOSE LOWERING DRUGS, EXCL. INSULINS	37.6	30.6	0.15	35.8	36.7	-0.02
condition_era group during day -180 through 0 days relative to index: Angioedema	0.1	0.1	-0.02	0.1	0.1	-0.02
drug_era group during day -180 through 0 days relative to index: sevelamer	0.5	0.1	0.07	0.2	0.2	0.02

CUIMC

Supplementary Figure 41: Preference score distributions, propensity score stratified (middle column) and matched (right column) covariate balance and negative control error distributions (bottom row) for ACE/ARB mono vs CCB/THZ mono in the CUIMC data source.

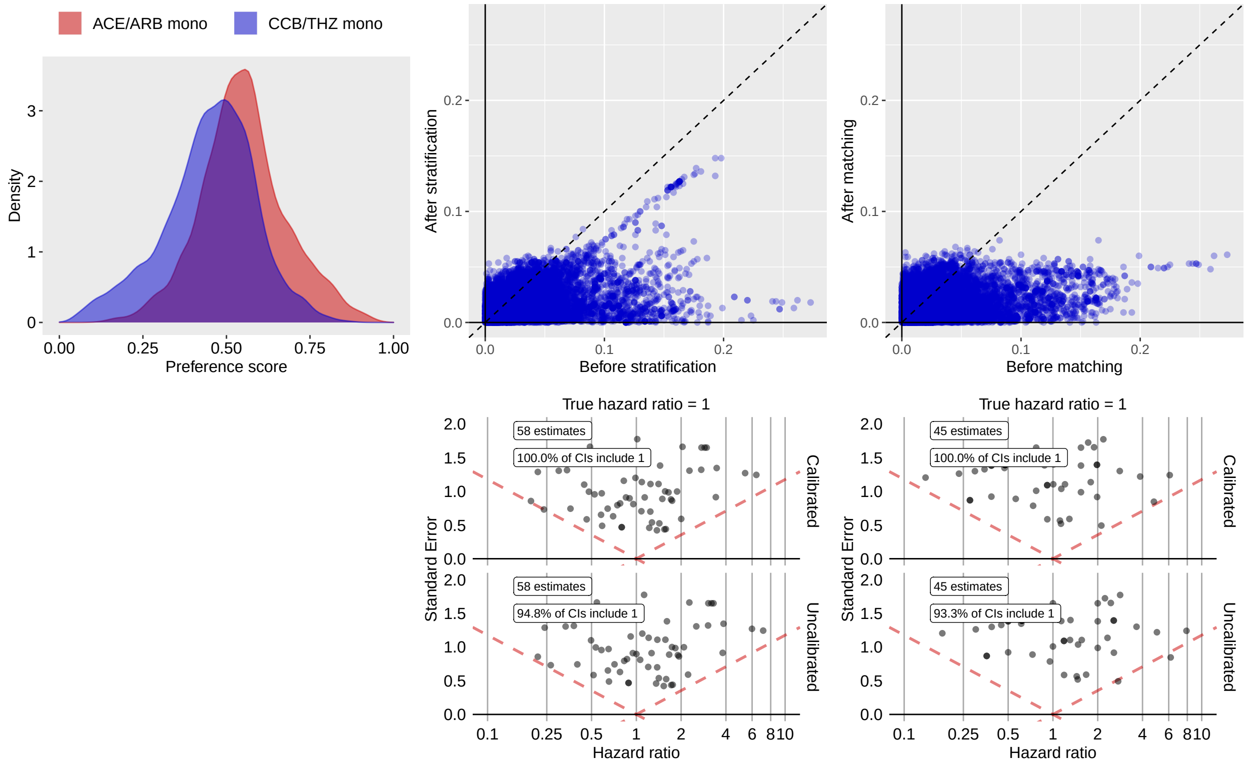

Supplementary Table 145: Most extreme patient characteristics balance diagnostics after stratification for ACE/ARB mono vs CCB/THZ mono in the CUIMC data source.

	Before stratification			After stratification		
Characteristic (total count = 27590)	T (%)	C (%)	SDf	T (%)	C (%)	SDf
condition_era group during day -180 through 0 days relative to index: Complication of pregnancy, childbirth and/or the puerperium	-0.1	2.1	-0.20	0.1	1.4	-0.15
condition_era group during day -180 through 0 days relative to index: Hypertension AND/OR vomiting complicating pregnancy childbirth AND/OR puerperium	-0.1	1.9	-0.19	-0.1	1.3	-0.15
procedure_occurrence during day -180 through 0 days relative to index: Ultrasound, pregnant uterus, real time with image documentation, follow-up (eg, re-evaluation of fetal size by measuring standard growth parameters and amniotic fluid volume, re-evaluation of organ system(s) suspected or confirmed to be abnormal on a prev	-0.1	1.6	-0.18	-0.1	1.1	-0.14
condition_era group during day -30 through 0 days relative to index: Finding related to pregnancy	0.1	1.9	-0.18	0.1	1.3	-0.14

(Continued)	T (%)	C (%)	SDf	T (%)	C (%)	SDf
condition_era group during day -180 through 0 days relative to index: Pregnancy-induced hypertension	-0.1	1.6	-0.18	-0.1	1.1	-0.13
condition_era group during day -180 through 0 days relative to index: Third trimester pregnancy	-0.1	1.6	-0.17	-0.1	1.1	-0.13
condition_era group during day -180 through 0 days relative to index: Finding related to pregnancy	0.2	2.4	-0.19	0.3	1.6	-0.13
drug_era group during day -180 through 0 days relative to index: Witch Hazel	-0.1	1.4	-0.17	-0.1	1.0	-0.13
drug_era group during day -180 through 0 days relative to index: Lanolin	-0.1	1.4	-0.17	-0.1	0.9	-0.13
drug_era group during day -180 through 0 days relative to index: Oxytocin	-0.1	1.4	-0.16	-0.1	0.9	-0.13

Supplementary Table 146: Most extreme patient characteristics balance diagnostics after matching for ACE/ARB mono vs CCB/THZ mono in the CUIMC data source.

	Before matching			After matching		
Characteristic (total count = 26503)	T (%)	C (%)	SDf	T (%)	C (%)	SDf
measurement within normal range during day -180 through 0 days relative to index: Glucose [Mass/volume] in Serum or Plasma	19.3	26.2	-0.17	21.6	18.6	0.07
condition_era group during day -180 through 0 days relative to index: Precapillary pulmonary hypertension	0.2	0.7	-0.08	0.1	0.6	-0.07
condition_era group during day -180 through 0 days relative to index: Congenital anomaly of musculoskeletal system	0.3	0.1	0.04	0.4	0.1	0.07
condition_era group during day -30 through 0 days relative to index: Asteatosis cutis	0.3	-0.1	0.06	0.3	-0.1	0.07
condition_era group during day -180 through 0 days relative to index: Pulmonary arterial hypertension	0.1	0.6	-0.08	0.1	0.5	-0.07
condition_era group during day -30 through 0 days relative to index: Frank hematuria	0.4	0.2	0.05	0.4	0.1	0.07
condition_era group during day -180 through 0 days relative to index: Gastroparesis syndrome	0.3	-0.1	0.06	0.4	0.1	0.07
procedure_occurrence during day -180 through 0 days relative to index: Programming device evaluation (in person) with iterative adjustment of the implantable device to test the function of the device and select optimal permanent programmed values with analysis, review and report by a physician or other qualified health care	0.2	-0.1	0.06	0.3	-0.1	0.06
condition_era group during day -180 through 0 days relative to index: Glomerular disease	0.8	0.5	0.04	0.9	0.4	0.06
condition_era group during day -30 through 0 days relative to index: Raynaud's disease	-0.1	0.3	-0.06	-0.1	0.3	-0.06

Supplementary Figure 42: Preference score distributions, propensity score stratified (middle column) and matched (right column) covariate balance and negative control error distributions (bottom row) for ACE/ARB +combo vs CCB/THZ +combo in the CUIMC data source.

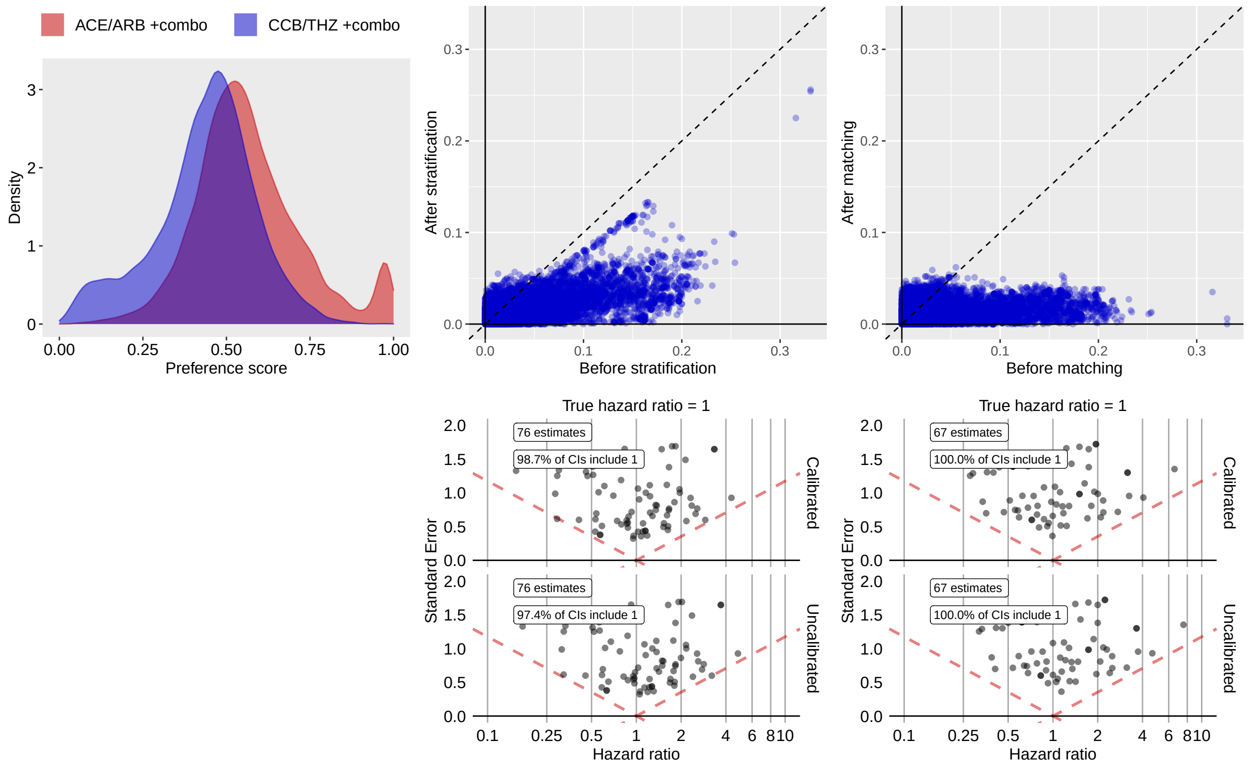

Supplementary Table 147: Most extreme patient characteristics balance diagnostics after stratification for ACE/ARB +combo vs CCB/THZ +combo in the CUIMC data source.

	Before stratification			After stratification		
Characteristic (total count = 32654)	T (%)	C (%)	SDf	T (%)	C (%)	SDf
drug_era group during day 0 through 0 days relative to index: sacubitril	5.3	0.0	0.33	3.3	0.1	0.26
drug_era group during day -30 through 0 days relative to index: sacubitril	5.3	0.0	0.33	3.3	0.1	0.25
drug_era group during day -180 through 0 days relative to index: sacubitril	5.4	0.2	0.32	3.4	0.4	0.23
condition_era group during day -30 through 0 days relative to index: Complication of pregnancy, childbirth and/or the puerperium	0.0	1.5	-0.17	-0.1	1.0	-0.13
condition_era group during day -30 through 0 days relative to index: Hypertension AND/OR vomiting complicating pregnancy childbirth AND/OR puerperium	0.0	1.4	-0.17	-0.1	0.9	-0.13
condition_era group during day -180 through 0 days relative to index: Maternal AND/OR fetal condition affecting labor AND/OR delivery	0.0	1.3	-0.16	-0.1	0.9	-0.13

(Continued)	T (%)	C (%)	SDf	T (%)	C (%)	SDf
procedure_occurrence during day -180 through 0 days relative to index: Ultrasound, pregnant uterus, real time with image documentation, follow-up (eg, re-evaluation of fetal size by measuring standard growth parameters and amniotic fluid volume, re-evaluation of organ system(s) suspected or confirmed to be abnormal on a prev	0.0	1.4	-0.17	-0.1	0.9	-0.13
condition_era group during day -180 through 0 days relative to index: Hypertension AND/OR vomiting complicating pregnancy childbirth AND/OR puerperium	0.1	1.6	-0.17	0.1	1.1	-0.13
condition_era group during day -180 through 0 days relative to index: Third trimester pregnancy	0.0	1.4	-0.16	-0.1	0.9	-0.13
condition_era group during day -180 through 0 days relative to index: Complication of pregnancy, childbirth and/or the puerperium	0.1	1.8	-0.17	0.2	1.2	-0.12

Supplementary Table 148: Most extreme patient characteristics balance diagnostics after matching for ACE/ARB +combo vs CCB/THZ +combo in the CUIMC data source.

	Before matching			After matching		
Characteristic (total count = 31587)	T (%)	C (%)	SDf	T (%)	C (%)	SDf
procedure_occurrence during day -30 through 0 days relative to index: Catheter placement in coronary artery(s) for coronary angiography, including intraprocedural injection(s) for coronary angiography, imaging supervision and interpretation; with left heart catheterization including intraprocedural injection(s) for left ven	0.2	0.6	-0.06	0.2	0.6	-0.06
condition_era group during day -180 through 0 days relative to index: Type 1 diabetes mellitus without complication	0.8	0.5	0.03	0.8	0.3	0.06
condition_era group during day -180 through 0 days relative to index: Fibrocystic disease of breast	0.1	0.2	-0.05	-0.1	0.3	-0.05
condition_era group during day -180 through 0 days relative to index: Angioedema	0.0	0.2	-0.05	-0.1	0.2	-0.05
condition_era group during day -180 through 0 days relative to index: Maternal AND/OR fetal condition affecting labor AND/OR delivery	0.0	1.3	-0.16	-0.1	0.2	-0.05
condition_era group during day -180 through 0 days relative to index: Type 1 diabetes mellitus	1.1	0.8	0.03	1.0	0.5	0.05
condition_era group during day -30 through 0 days relative to index: Coronary occlusion	0.1	0.2	-0.02	-0.1	0.3	-0.05
condition_era group during day -30 through 0 days relative to index: Chronic total occlusion of coronary artery	0.1	0.2	-0.02	-0.1	0.3	-0.05
condition_era group during day -180 through 0 days relative to index: Acute conjunctivitis	0.0	0.2	-0.05	-0.1	0.2	-0.05
condition_era group during day -180 through 0 days relative to index: Acute inflammation of orbit	0.0	0.2	-0.05	-0.1	0.2	-0.05

Supplementary Figure 43: Preference score distributions, propensity score stratified (middle column) and matched (right column) covariate balance and negative control error distributions (bottom row) for ACE/ARB mono vs CCB mono in the CUIMC data source.

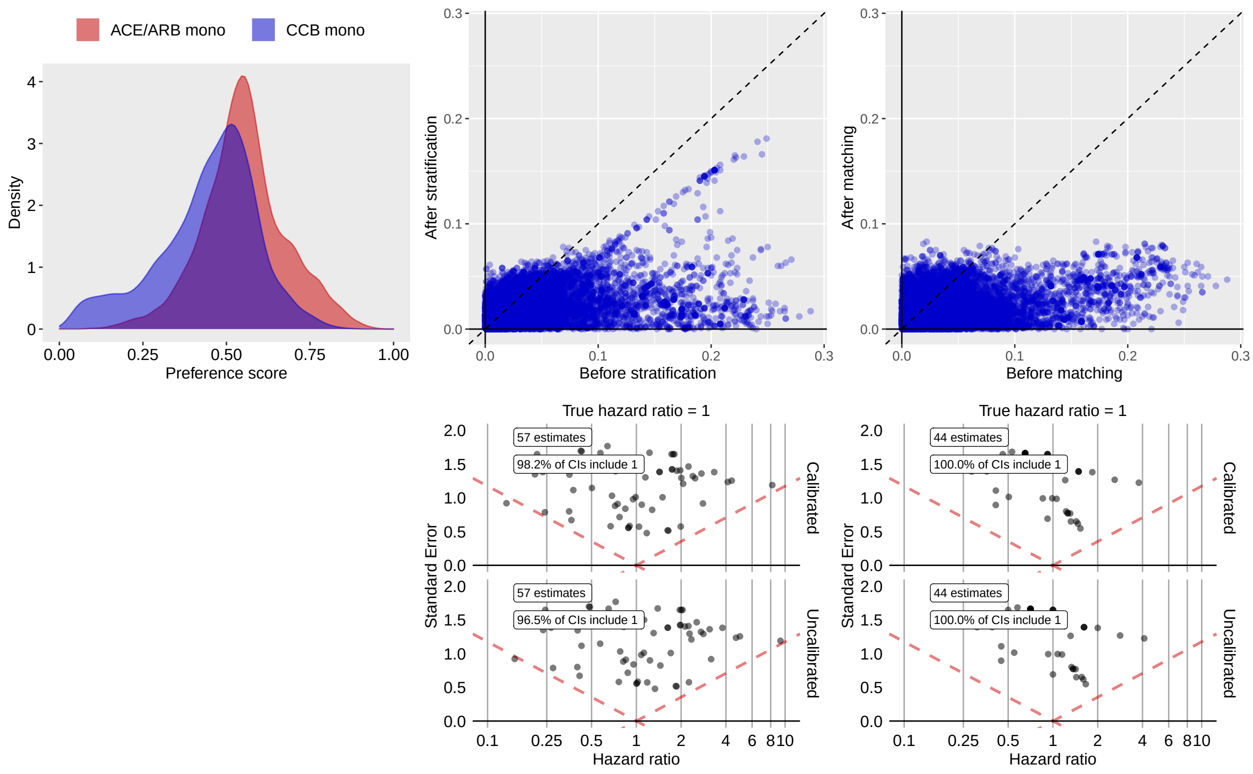

Supplementary Table 149: Most extreme patient characteristics balance diagnostics after stratification for ACE/ARB mono vs CCB mono in the CUIMC data source.

	Before stratification			After stratification		
Characteristic (total count = 27051)	T (%)	C (%)	SDf	T (%)	C (%)	SDf
condition_era group during day -180 through 0 days relative to index: Complication of pregnancy, childbirth and/or the puerperium	-0.1	3.2	-0.25	0.1	1.9	-0.18
condition_era group during day -180 through 0 days relative to index: Hypertension AND/OR vomiting complicating pregnancy childbirth AND/OR puerperium	-0.1	2.9	-0.24	-0.1	1.7	-0.18
condition_era group during day -180 through 0 days relative to index: Finding related to pregnancy	0.2	3.5	-0.24	0.3	2.1	-0.17
procedure_occurrence during day -180 through 0 days relative to index: Ultrasound, pregnant uterus, real time with image documentation, follow-up (eg, re-evaluation of fetal size by measuring standard growth parameters and amniotic fluid volume, re-evaluation of organ system(s) suspected or confirmed to be abnormal on a prev	-0.1	2.4	-0.22	-0.1	1.4	-0.17
condition_era group during day -30 through 0 days relative to index: Finding related to pregnancy	0.1	2.8	-0.23	0.1	1.7	-0.16
condition_era group during day -180 through 0 days relative to index: Pregnancy-induced hypertension	-0.1	2.5	-0.22	-0.1	1.5	-0.16

(Continued)	T (%)	C (%)	SDf	T (%)	C (%)	SDf
condition_era group during day -180 through 0 days relative to index: Third trimester pregnancy	-0.1	2.5	-0.22	-0.1	1.5	-0.16
drug_era group during day -180 through 0 days relative to index: Witch Hazel	-0.1	2.2	-0.21	-0.1	1.3	-0.16
drug_era group during day -180 through 0 days relative to index: Lanolin	-0.1	2.1	-0.20	-0.1	1.3	-0.15
drug_era group during day -180 through 0 days relative to index: Oxytocin	-0.1	2.1	-0.20	-0.1	1.2	-0.15

Supplementary Table 150: Most extreme patient characteristics balance diagnostics after matching for ACE/ARB mono vs CCB mono in the CUIMC data source.

	Before matching			After matching		
Characteristic (total count = 25681)	T (%)	C (%)	SDf	T (%)	C (%)	SDf
measurement below normal range during day -180 through 0 days relative to index: Protein [Mass/volume] in Serum or Plasma	4.0	8.1	-0.17	5.4	3.7	0.08
condition_era group during day -180 through 0 days relative to index: Congenital renal cyst	0.3	0.2	0.02	0.4	-0.2	0.08
condition_era group during day -180 through 0 days relative to index: Multiple congenital cysts of kidney	0.3	0.2	0.02	0.4	-0.2	0.08
drug_era group during day -180 through 0 days relative to index: I.V. SOLUTION ADDITIVES	11.3	18.9	-0.21	14.3	11.6	0.08
drug_era group during day -180 through 0 days relative to index: Electrolyte solutions	11.3	18.9	-0.21	14.3	11.6	0.08
drug_era group during day -180 through 0 days relative to index: I.V. SOLUTIONS	10.5	18.5	-0.23	13.6	11.0	0.08
condition_era group during day -180 through 0 days relative to index: Corneal degeneration	0.3	-0.1	0.07	0.4	-0.2	0.08
drug_era group during day -180 through 0 days relative to index: Sodium Chloride	10.0	18.0	-0.23	13.0	10.5	0.08
measurement during day -30 through 0 days relative to index: Anion gap in Serum or Plasma	20.5	28.3	-0.18	23.6	20.3	0.08
drug_era group during day -180 through 0 days relative to index: Sodium	10.0	18.0	-0.23	13.0	10.5	0.08

Supplementary Figure 44: Preference score distributions, propensity score stratified (middle column) and matched (right column) covariate balance and negative control error distributions (bottom row) for ACE/ARB +combo vs CCB +combo in the CUIMC data source.

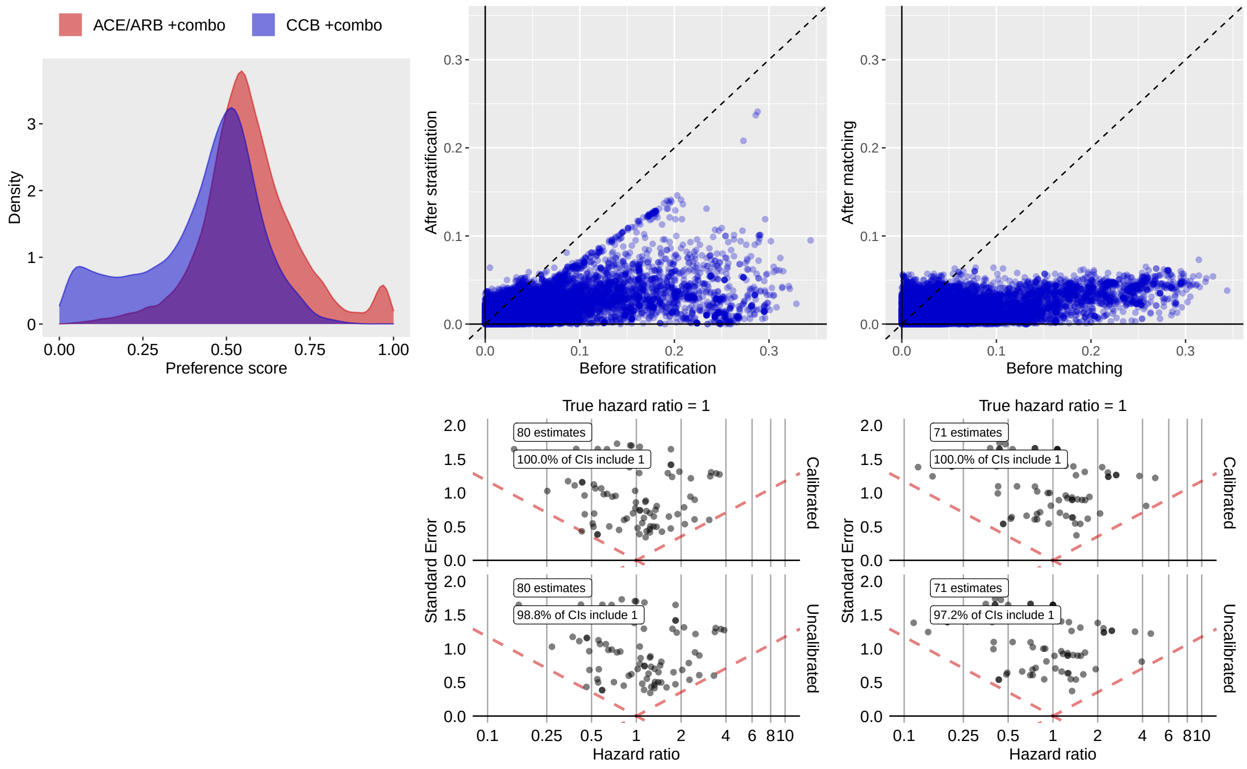

Supplementary Table 151: Most extreme patient characteristics balance diagnostics after stratification for ACE/ARB +combo vs CCB +combo in the CUIMC data source.

	Before stratification			After stratification		
Characteristic (total count = 33550)	T (%)	C (%)	SDf	T (%)	C (%)	SDf
drug_era group during day 0 through 0 days relative to index: sacubitril	4.0	-0.1	0.29	3.0	0.1	0.24
drug_era group during day -30 through 0 days relative to index: sacubitril	4.1	-0.1	0.29	3.0	0.1	0.24
drug_era group during day -180 through 0 days relative to index: sacubitril	4.2	0.2	0.27	3.2	0.4	0.21
condition_era group during day -180 through 0 days relative to index: Second trimester pregnancy	0.0	2.0	-0.20	0.0	1.1	-0.15
procedure_occurrence during day -180 through 0 days relative to index: Ultrasound, pregnant uterus, real time with image documentation, follow-up (eg, re-evaluation of fetal size by measuring standard growth parameters and amniotic fluid volume, re-evaluation of organ system(s) suspected or confirmed to be abnormal on a prev	0.0	1.9	-0.20	0.0	1.0	-0.14
condition_era group during day -180 through 0 days relative to index: Hypertension AND/OR vomiting complicating pregnancy childbirth AND/OR puerperium	0.1	2.3	-0.21	0.1	1.2	-0.14

(Continued)	T (%)	C (%)	SDf	T (%)	C (%)	SDf
condition_era group during day -180 through 0 days relative to index: Maternal AND/OR fetal condition affecting labor AND/OR delivery	0.0	1.8	-0.19	0.0	1.0	-0.14
condition_era group during day -30 through 0 days relative to index: Complication of pregnancy, childbirth and/or the puerperium	0.0	2.0	-0.20	0.0	1.1	-0.14
condition_era group during day -30 through 0 days relative to index: Hypertension AND/OR vomiting complicating pregnancy childbirth AND/OR puerperium	0.0	1.9	-0.19	0.0	1.0	-0.14
procedure_occurrence during day -180 through 0 days relative to index: Ultrasound, pregnant uterus, real time with image documentation, transvaginal	0.0	1.8	-0.19	0.0	1.0	-0.14

Supplementary Table 152: Most extreme patient characteristics balance diagnostics after matching for ACE/ARB +combo vs CCB +combo in the CUIMC data source.

	Before matching			After matching		
Characteristic (total count = 32884)	T (%)	C (%)	SDf	T (%)	C (%)	SDf
measurement below normal range during day -180 through 0 days relative to index: Hematocrit [Volume Fraction] of Blood	10.6	22.1	-0.31	14.1	11.7	0.07
measurement below normal range during day -180 through 0 days relative to index: Protein [Mass/volume] in Serum or Plasma	6.8	14.8	-0.26	8.9	7.1	0.07
procedure_occurrence during day -180 through 0 days relative to index: Insertion of Infusion Device into Superior Vena Cava, Percutaneous Approach	0.4	1.3	-0.10	0.7	0.2	0.06
procedure_occurrence during day -30 through 0 days relative to index: Stereotactic computer-assisted (navigational) procedure; cranial, intradural (List separately in addition to code for primary procedure)	0.0	0.3	-0.08	0.0	0.2	-0.06
drug_era group during day -180 through 0 days relative to index: OTHER OPHTHALMOLOGICALS	16.4	27.4	-0.27	20.1	17.6	0.06
drug_era group during day -180 through 0 days relative to index: Other ophthalmologicals	16.4	27.4	-0.27	20.1	17.6	0.06
measurement below normal range during day -180 through 0 days relative to index: Iron binding capacity.unsaturated [Mass/volume] in Serum or Plasma	0.4	1.4	-0.11	0.7	0.3	0.06
measurement below normal range during day -180 through 0 days relative to index: Sodium [Moles/volume] in Venous blood	0.6	1.2	-0.06	0.9	0.4	0.06
condition_era group during day -180 through 0 days relative to index: Maternal AND/OR fetal condition affecting labor AND/OR delivery	0.0	1.8	-0.19	0.0	0.2	-0.06
drug_era group during day -180 through 0 days relative to index: I.V. SOLUTIONS	16.0	27.3	-0.28	19.7	17.3	0.06

Supplementary Figure 45: Preference score distributions, propensity score stratified (middle column) and matched (right column) covariate balance and negative control error distributions (bottom row) for ACE/ARB mono vs THZ mono in the CUIMC data source.

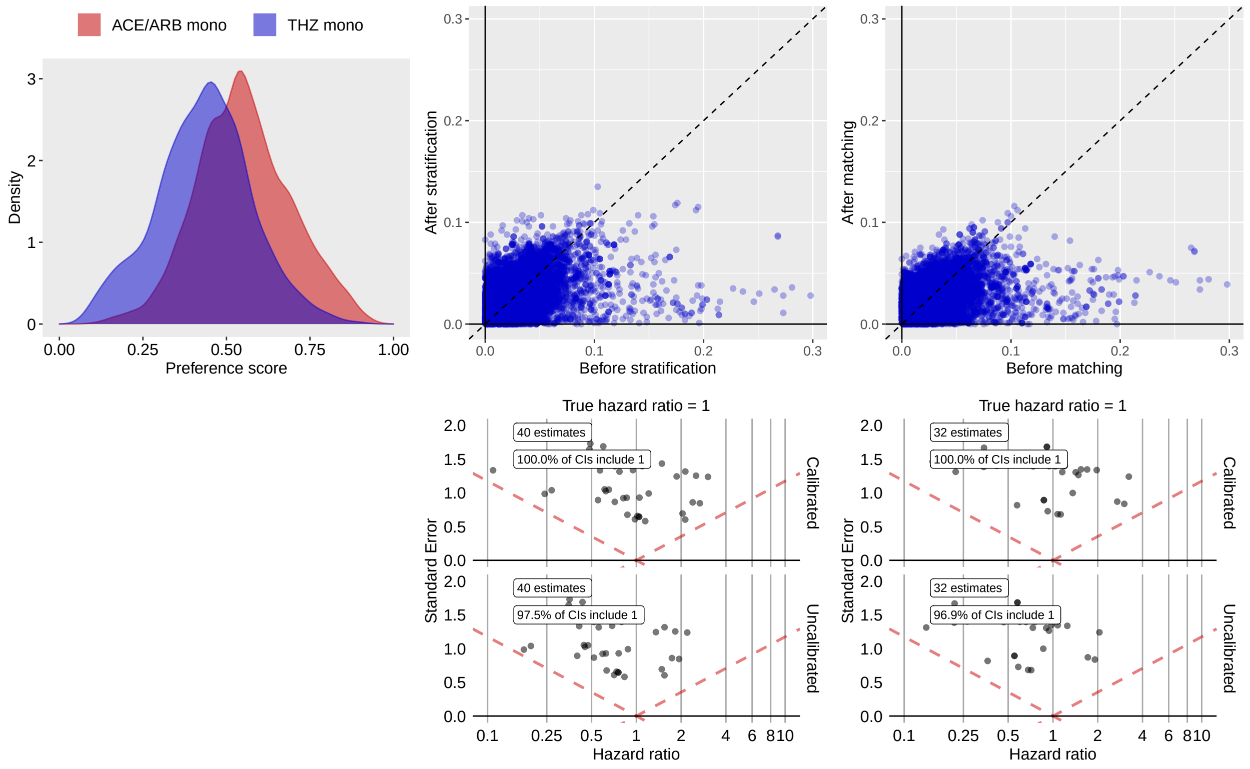

Supplementary Table 153: Most extreme patient characteristics balance diagnostics after stratification for ACE/ARB mono vs THZ mono in the CUIMC data source.

Characteristic (total count = 24311)	Before stratification			After stratification		
	T (%)	C (%)	SDf	T (%)	C (%)	SDf
condition_era group during day -180 through 0 days relative to index: Precapillary pulmonary hypertension	0.2	0.9	-0.10	0.1	1.3	-0.14
drug_era group during day -30 through 0 days relative to index: Insulins and analogues for injection, fast-acting	2.3	0.3	0.18	2.0	0.7	0.12
drug_era group during day -30 through 0 days relative to index: Insulins and analogues for injection, intermediate- or long-acting combined with fast-acting	2.3	0.3	0.17	2.0	0.7	0.12
drug_era group during day -180 through 0 days relative to index: Insulins and analogues for injection, fast-acting	3.3	0.6	0.20	2.8	1.2	0.12
drug_era group during day -180 through 0 days relative to index: Vancomycin	1.1	-0.3	0.10	1.0	-0.3	0.11
drug_era group during day -180 through 0 days relative to index: Insulins and analogues for injection, intermediate- or long-acting combined with fast-acting	3.2	0.6	0.19	2.8	1.2	0.11
condition_era group during day -30 through 0 days relative to index: Abnormal findings on diagnostic imaging of lung	1.4	0.4	0.10	1.3	0.3	0.11

(Continued)	T (%)	C (%)	SDf	T (%)	C (%)	SDf
drug_era group during day -30 through 0 days relative to index: Methylprednisolone	1.8	0.7	0.09	1.7	0.5	0.11
condition_era group during day -30 through 0 days relative to index: Cardiomyopathy	1.3	-0.3	0.14	1.2	-0.3	0.11
measurement during day -180 through 0 days relative to index: Alpha-1-fetoprotein.tumor marker [Mass/volume] in Serum or Plasma	0.4	0.8	-0.05	0.4	1.4	-0.11

Supplementary Table 154: Most extreme patient characteristics balance diagnostics after matching for ACE/ARB mono vs THZ mono in the CUIMC data source.

	Before matching			After matching		
Characteristic (total count = 23553)	T (%)	C (%)	SDf	T (%)	C (%)	SDf
condition_era group during day -180 through 0 days relative to index: Precapillary pulmonary hypertension	0.2	0.9	-0.10	0.1	1.0	-0.12
drug_era group during day -30 through 0 days relative to index: potassium citrate	0.2	1.0	-0.11	0.2	1.0	-0.11
drug_era group during day -180 through 0 days relative to index: potassium citrate	0.3	1.2	-0.10	0.3	1.3	-0.11
drug_era group during day 0 through 0 days relative to index: potassium citrate	0.2	0.9	-0.10	0.2	1.0	-0.11
measurement below normal range during day -180 through 0 days relative to index: Bilirubin.total [Mass/volume] in Serum or Plasma	1.9	1.0	0.07	1.9	0.8	0.10
condition_era group during day -180 through 0 days relative to index: Pulmonary arterial hypertension	0.1	0.7	-0.09	0.1	0.8	-0.10
drug_era group during day -30 through 0 days relative to index: POTASSIUM	0.2	1.0	-0.10	0.2	1.0	-0.10
drug_era group during day -30 through 0 days relative to index: Potassium	0.2	1.0	-0.10	0.2	1.0	-0.10
condition_era group during day -180 through 0 days relative to index: Malaise	0.8	1.4	-0.06	0.5	1.5	-0.10
drug_era group during day -180 through 0 days relative to index: ANTIOBESITY PREPARATIONS, EXCL. DIET PRODUCTS	0.4	1.0	-0.07	0.3	1.0	-0.10

Supplementary Figure 46: Preference score distributions, propensity score stratified (middle column) and matched (right column) covariate balance and negative control error distributions (bottom row) for ACE/ARB +combo vs THZ +combo in the CUIMC data source.

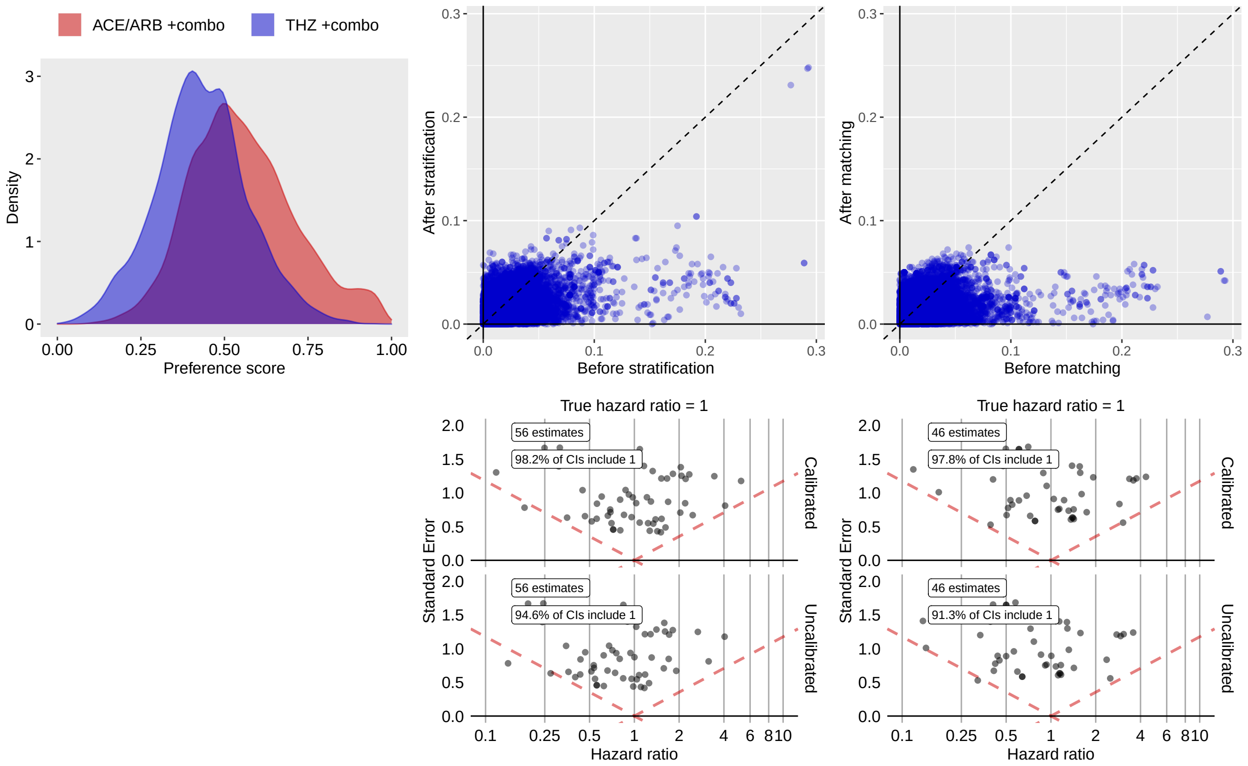

Supplementary Table 155: Most extreme patient characteristics balance diagnostics after stratification for ACE/ARB +combo vs THZ +combo in the CUIMC data source.

	Before stratification			After stratification		
Characteristic (total count = 33670)	T (%)	C (%)	SDf	T (%)	C (%)	SDf
drug_era group during day -30 through 0 days relative to index: sacubitril	4.3	-0.1	0.29	3.5	0.2	0.25
drug_era group during day 0 through 0 days relative to index: sacubitril	4.2	-0.1	0.29	3.5	0.2	0.25
drug_era group during day -180 through 0 days relative to index: sacubitril	4.4	0.3	0.28	3.6	0.4	0.23
condition_era group during day -180 through 0 days relative to index: Systolic dysfunction	8.8	4.1	0.19	7.6	5.1	0.10
condition_era group during day -180 through 0 days relative to index: Systolic heart failure	8.7	4.0	0.19	7.5	5.0	0.10
condition_era group during day -180 through 0 days relative to index: Chronic systolic heart failure	7.4	3.4	0.17	6.4	4.3	0.10
drug_era group during day -30 through 0 days relative to index: potassium citrate	0.3	0.9	-0.09	0.3	1.0	-0.09
drug_era group during day 0 through 0 days relative to index: potassium citrate	0.2	0.8	-0.08	0.3	1.0	-0.09

(Continued)	T (%)	C (%)	SDf	T (%)	C (%)	SDf
procedure_occurrence during day -180 through 0 days relative to index: Renal biopsy; percutaneous, by trocar or needle	0.2	0.6	-0.06	0.2	0.9	-0.09
condition_era group during day -180 through 0 days relative to index: Type 1 diabetes mellitus	1.2	0.3	0.10	1.1	0.4	0.09

Supplementary Table 156: Most extreme patient characteristics balance diagnostics after matching for ACE/ARB +combo vs THZ +combo in the CUIMC data source.

	Before matching			After matching		
Characteristic (total count = 32966)	T (%)	C (%)	SDf	T (%)	C (%)	SDf
observation during day -180 through 0 days relative to index: Dependence on renal dialysis	0.7	0.3	0.06	0.8	0.2	0.07
drug_era group during day 0 through 0 days relative to index: Calcium Gluconate	0.2	0.9	-0.10	0.1	0.6	-0.07
condition_era group during day -30 through 0 days relative to index: Congenital abnormality of lower limb and pelvic girdle	0.3	-0.1	0.04	0.4	-0.1	0.07
drug_era group during day -30 through 0 days relative to index: Lovastatin	0.1	0.5	-0.06	0.1	0.5	-0.07
measurement during day -30 through 0 days relative to index: Varicella zoster virus Ab [Units/volume] in Serum	0.3	0.1	0.04	0.3	-0.1	0.07
measurement above normal range during day -180 through 0 days relative to index: Glucose [Mass/volume] in Cerebral spinal fluid	0.2	-0.1	0.05	0.3	-0.1	0.07
measurement during day -180 through 0 days relative to index: INR in Capillary blood by Coagulation assay	0.6	0.1	0.08	0.5	0.1	0.07
drug_era group during day -30 through 0 days relative to index: DIURETICS	0.0	0.5	-0.08	0.0	0.3	-0.07
measurement above normal range during day -180 through 0 days relative to index: INR in Capillary blood by Coagulation assay	0.6	0.1	0.08	0.5	0.1	0.07
condition_era group during day -30 through 0 days relative to index: Oral lesion	0.3	-0.1	0.06	0.4	-0.1	0.07

Supplementary Figure 47: Preference score distributions, propensity score stratified (middle column) and matched (right column) covariate balance and negative control error distributions (bottom row) for ACE mono vs ARB mono in the CUIMC data source.

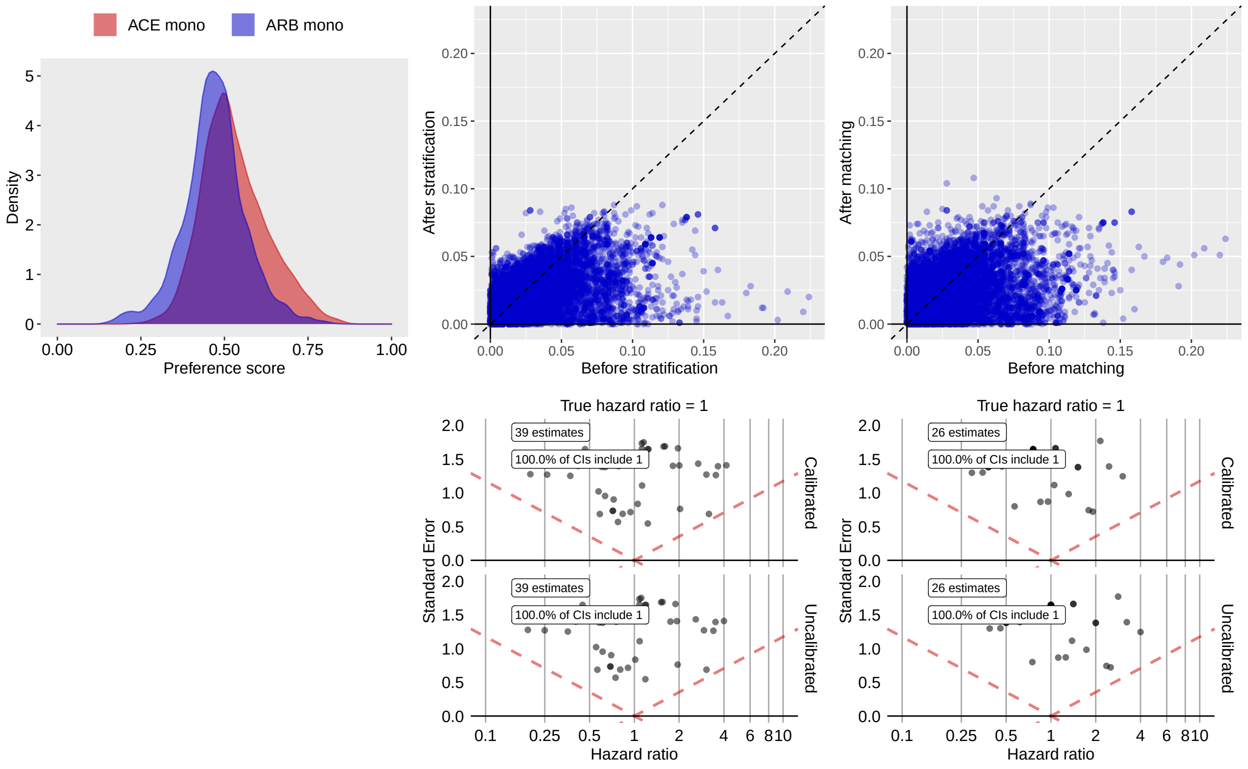

Supplementary Table 157: Most extreme patient characteristics balance diagnostics after stratification for ACE mono vs ARB mono in the CUIMC data source.

	Before stratification			After stratification		
Characteristic (total count = 23425)	T (%)	C (%)	SDf	T (%)	C (%)	SDf
drug_era group during day -180 through 0 days relative to index: Omega-3 Acid Ethyl Esters (USP)	1.1	2.2	-0.09	1.1	2.2	-0.09
drug_era group during day -30 through 0 days relative to index: Nucleoside and nucleotide reverse transcriptase inhibitors	1.4	0.3	0.12	1.1	0.4	0.09
drug_era group during day -180 through 0 days relative to index: abacavir	0.6	-0.2	0.10	0.5	-0.2	0.09
drug_era group during day 0 through 0 days relative to index: Mirtazapine	0.7	1.3	-0.06	0.6	1.4	-0.09
drug_era group during day -180 through 0 days relative to index: Mirtazapine	1.1	1.7	-0.05	0.9	1.9	-0.09
drug_era group during day -180 through 0 days relative to index: Fentanyl	2.9	3.4	-0.03	2.4	3.9	-0.08
drug_era group during day -30 through 0 days relative to index: abacavir	0.6	-0.2	0.10	0.4	-0.2	0.08
drug_era group during day -180 through 0 days relative to index: Opioid anesthetics	2.9	3.4	-0.03	2.4	3.9	-0.08

(Continued)	T (%)	C (%)	SDf	T (%)	C (%)	SDf
drug_era group during day -180 through 0 days relative to index: Phenylpiperidine derivatives	2.9	3.4	-0.03	2.4	3.9	-0.08
observation during day -180 through 0 days relative to index: H/O: drug allergy	1.2	1.6	-0.04	0.9	1.9	-0.08

Supplementary Table 158: Most extreme patient characteristics balance diagnostics after matching for ACE mono vs ARB mono in the CUIMC data source.

	Before matching			After matching		
Characteristic (total count = 22414)	T (%)	C (%)	SDf	T (%)	C (%)	SDf
condition_era group during day -180 through 0 days relative to index: Abnormal findings on diagnostic imaging of lung	2.7	3.5	-0.05	1.8	3.5	-0.11
condition_era group during day -30 through 0 days relative to index: Abnormal findings on diagnostic imaging of lung	1.2	1.6	-0.03	0.6	1.7	-0.10
measurement below normal range during day -180 through 0 days relative to index: Alkaline phosphatase [Enzymatic activity/volume] in Serum or Plasma	0.6	1.2	-0.06	0.4	1.2	-0.09
condition_era group during day -180 through 0 days relative to index: Cardiac transplant disorder	0.2	0.8	-0.09	-0.2	0.8	-0.09
condition_era group during day -180 through 0 days relative to index: Fracture of bones of trunk	0.8	0.2	0.09	0.8	0.2	0.09
condition_era group during day -180 through 0 days relative to index: Acquired trigger finger	0.2	0.6	-0.06	-0.2	0.6	-0.09
drug_era group during day -180 through 0 days relative to index: Mirtazapine	1.1	1.7	-0.05	0.8	1.7	-0.09
measurement within normal range during day -180 through 0 days relative to index: Hematocrit [Volume Fraction] of Blood	22.3	19.1	0.08	17.4	20.9	-0.09
drug_era group during day -180 through 0 days relative to index: abacavir	0.6	-0.2	0.10	0.4	-0.2	0.09
drug_era group during day -30 through 0 days relative to index: abacavir	0.6	-0.2	0.10	0.4	-0.2	0.09

Supplementary Figure 48: Preference score distributions, propensity score stratified (middle column) and matched (right column) covariate balance and negative control error distributions (bottom row) for ACE +combo vs ARB +combo in the CUIMC data source.

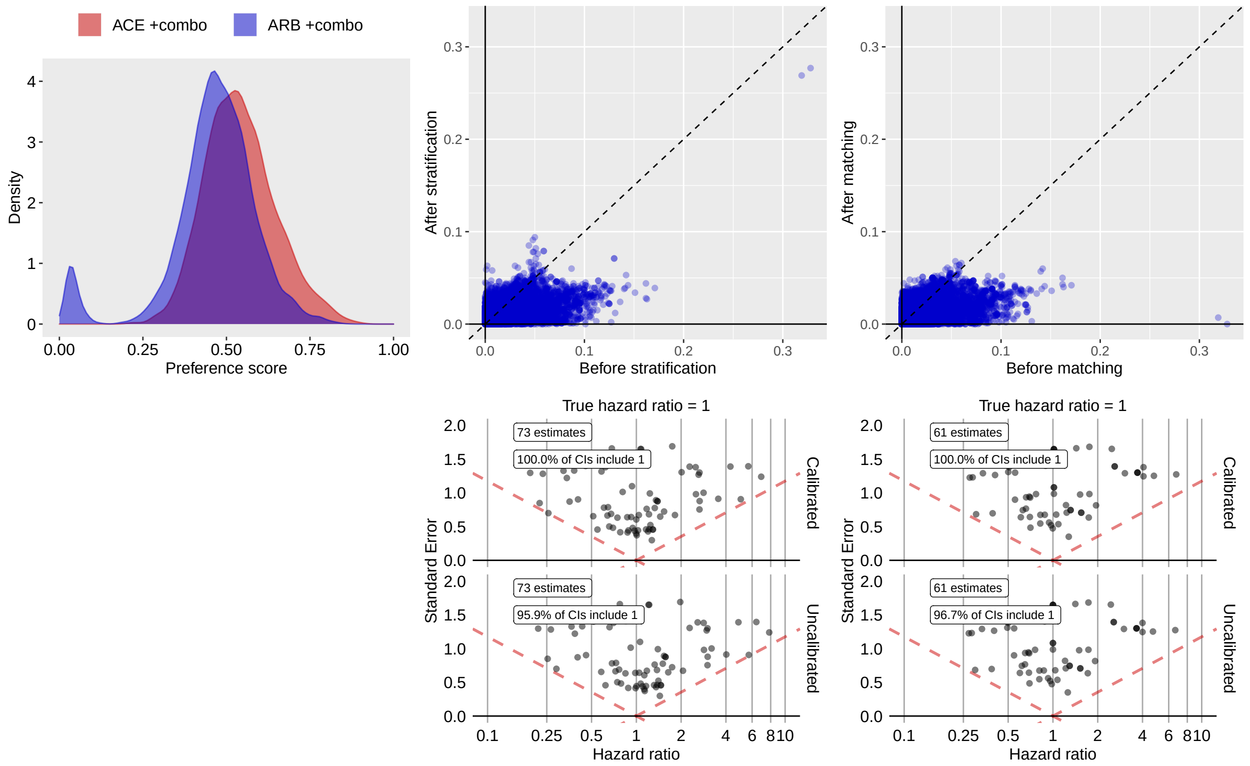

Supplementary Table 159: Most extreme patient characteristics balance diagnostics after stratification for ACE +combo vs ARB +combo in the CUIMC data source.

	Before stratification			After stratification		
Characteristic (total count = 35752)	T (%)	C (%)	SDf	T (%)	C (%)	SDf
drug_era group during day -30 through 0 days relative to index: sacubitril	-0.1	5.1	-0.33	-0.1	3.8	-0.28
drug_era group during day -180 through 0 days relative to index: sacubitril	0.1	5.2	-0.32	0.1	3.9	-0.27
condition_era group during day -180 through 0 days relative to index: Systolic heart failure	5.9	7.1	-0.05	4.7	6.9	-0.09
condition_era group during day -180 through 0 days relative to index: Systolic dysfunction	6.0	7.2	-0.05	4.8	6.9	-0.09
condition_era group during day -180 through 0 days relative to index: Chronic systolic heart failure	5.0	6.0	-0.04	3.9	5.7	-0.09
condition_era group during day -30 through 0 days relative to index: Systolic heart failure	3.1	4.1	-0.05	2.5	3.9	-0.08
condition_era group during day -180 through 0 days relative to index: Abnormal cardiovascular function	8.7	10.4	-0.06	7.8	10.0	-0.08
condition_era group during day -180 through 0 days relative to index: Congestive heart failure	8.8	10.6	-0.06	7.9	10.1	-0.08

(Continued)	T (%)	C (%)	SDf	T (%)	C (%)	SDf
condition_era group during day -30 through 0 days relative to index: Systolic dysfunction	3.2	4.1	-0.05	2.6	3.9	-0.08
condition_era group during day -30 through 0 days relative to index: Chronic systolic heart failure	2.5	3.3	-0.05	1.9	3.1	-0.08

Supplementary Table 160: Most extreme patient characteristics balance diagnostics after matching for ACE +combo vs ARB +combo in the CUIMC data source.

	Before matching			After matching		
Characteristic (total count = 34257)	T (%)	C (%)	SDf	T (%)	C (%)	SDf
procedure_occurrence during day -180 through 0 days relative to index: Performance of Urinary Filtration, Intermittent, Less than 6 Hours Per Day	0.2	0.6	-0.05	0.2	0.6	-0.07
measurement during day -180 through 0 days relative to index: Child weight centiles - finding	0.3	0.0	0.06	0.3	0.0	0.07
measurement during day -180 through 0 days relative to index: Child height centile finding	0.2	0.0	0.06	0.3	0.0	0.06
measurement during day -180 through 0 days relative to index: Body mass index (BMI) [Percentile]	0.2	0.0	0.06	0.3	0.0	0.06
condition_era group during day -180 through 0 days relative to index: Congenital anomaly of cardiac chamber	0.2	0.0	0.06	0.3	0.0	0.06
drug_era group during day 0 through 0 days relative to index: Beta-lactamase sensitive penicillins	-0.1	0.2	-0.05	-0.1	0.2	-0.06
measurement during day -180 through 0 days relative to index: Respiratory rate	27.9	21.8	0.14	22.5	25.0	-0.06
drug_era group during day 0 through 0 days relative to index: Penicillin V	-0.1	0.2	-0.04	-0.1	0.2	-0.06
procedure_occurrence during day -180 through 0 days relative to index: Transthoracic echocardiography for congenital cardiac anomalies; complete	0.4	0.1	0.06	0.4	0.1	0.06
procedure_occurrence during day -30 through 0 days relative to index: Performance of Urinary Filtration, Intermittent, Less than 6 Hours Per Day	0.1	0.3	-0.05	-0.1	0.3	-0.06

Supplementary Figure 49: Preference score distributions, propensity score stratified (middle column) and matched (right column) covariate balance and negative control error distributions (bottom row) for ACE mono vs CCB/THZ mono in the CUIMC data source.

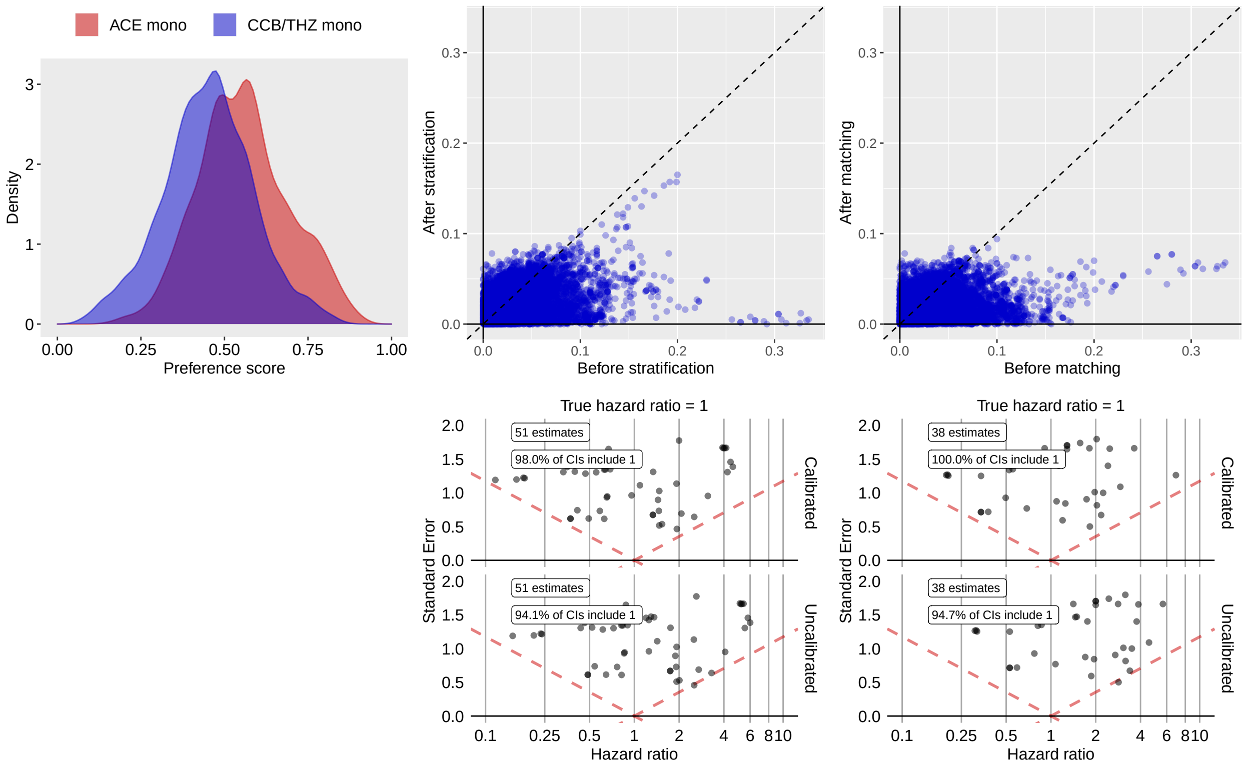

Supplementary Table 161: Most extreme patient characteristics balance diagnostics after stratification for ACE mono vs CCB/THZ mono in the CUIMC data source.

	Before stratification			After stratification		
Characteristic (total count = 25418)	T (%)	C (%)	SDf	T (%)	C (%)	SDf
condition_era group during day -180 through 0 days relative to index: Complication of pregnancy, childbirth and/or the puerperium	-0.2	2.1	-0.20	-0.2	1.6	-0.17
condition_era group during day -180 through 0 days relative to index: Finding related to pregnancy	-0.2	2.4	-0.20	0.3	1.8	-0.16
condition_era group during day -180 through 0 days relative to index: Hypertension AND/OR vomiting complicating pregnancy childbirth AND/OR puerperium	-0.2	1.9	-0.19	-0.2	1.5	-0.16
condition_era group during day -30 through 0 days relative to index: Finding related to pregnancy	-0.2	1.9	-0.19	-0.2	1.5	-0.15
measurement during day -180 through 0 days relative to index: Streptococcus agalactiae DNA [Presence] in Unspecified specimen by NAA with probe detection	-0.2	1.5	-0.17	-0.2	1.2	-0.15
condition_era group during day -180 through 0 days relative to index: Pregnancy-induced hypertension	-0.2	1.6	-0.18	-0.2	1.3	-0.14
drug_era group during day -180 through 0 days relative to index: POSTERIOR PITUITARY LOBE HORMONES	-0.2	1.5	-0.16	-0.2	1.2	-0.14

(Continued)	T (%)	C (%)	SDf	T (%)	C (%)	SDf
condition_era group during day -180 through 0 days relative to index: Pregnant	-0.2	1.4	-0.16	-0.2	1.1	-0.13
observation during day -180 through 0 days relative to index: Routine antenatal care	-0.2	1.2	-0.15	-0.2	0.9	-0.13
measurement during day -30 through 0 days relative to index: Oxygen saturation in Arterial blood	0.2	1.5	-0.14	-0.2	1.2	-0.12

Supplementary Table 162: Most extreme patient characteristics balance diagnostics after matching for ACE mono vs CCB/THZ mono in the CUIMC data source.

	Before matching			After matching		
Characteristic (total count = 24733)	T (%)	C (%)	SDf	T (%)	C (%)	SDf
condition_era group during day -180 through 0 days relative to index: Precapillary pulmonary hypertension	-0.2	0.7	-0.10	-0.2	0.7	-0.09
drug_era group during day -180 through 0 days relative to index: potassium citrate	-0.2	0.5	-0.07	-0.2	0.6	-0.08
procedure_occurrence during day -180 through 0 days relative to index: Ophthalmological services: medical examination and evaluation, with initiation or continuation of diagnostic and treatment program; comprehensive, established patient, 1 or more visits	1.6	1.0	0.05	1.7	0.8	0.08
condition_era group during day -180 through 0 days relative to index: Pulmonary arterial hypertension	-0.2	0.6	-0.09	-0.2	0.6	-0.08
condition_era group during day -180 through 0 days relative to index: Hemorrhage of intracranial meningeal space	-0.2	0.5	-0.08	-0.2	0.5	-0.08
condition_era group during day -180 through 0 days relative to index: Hemorrhage into meningeal space of neuraxis	-0.2	0.5	-0.08	-0.2	0.5	-0.08
condition_era group during day -180 through 0 days relative to index: Allergic disorder of skin	0.5	0.2	0.05	0.5	0.1	0.08
age group: 30-34	1.6	1.5	0.01	1.7	0.8	0.08
drug_era group during day -30 through 0 days relative to index: Metformin	15.4	6.7	0.28	10.6	13.0	-0.08
drug_era group during day -30 through 0 days relative to index: Biguanides	15.4	6.7	0.28	10.6	13.0	-0.08

Supplementary Figure 50: Preference score distributions, propensity score stratified (middle column) and matched (right column) covariate balance and negative control error distributions (bottom row) for ACE +combo vs CCB/THZ +combo in the CUIMC data source.

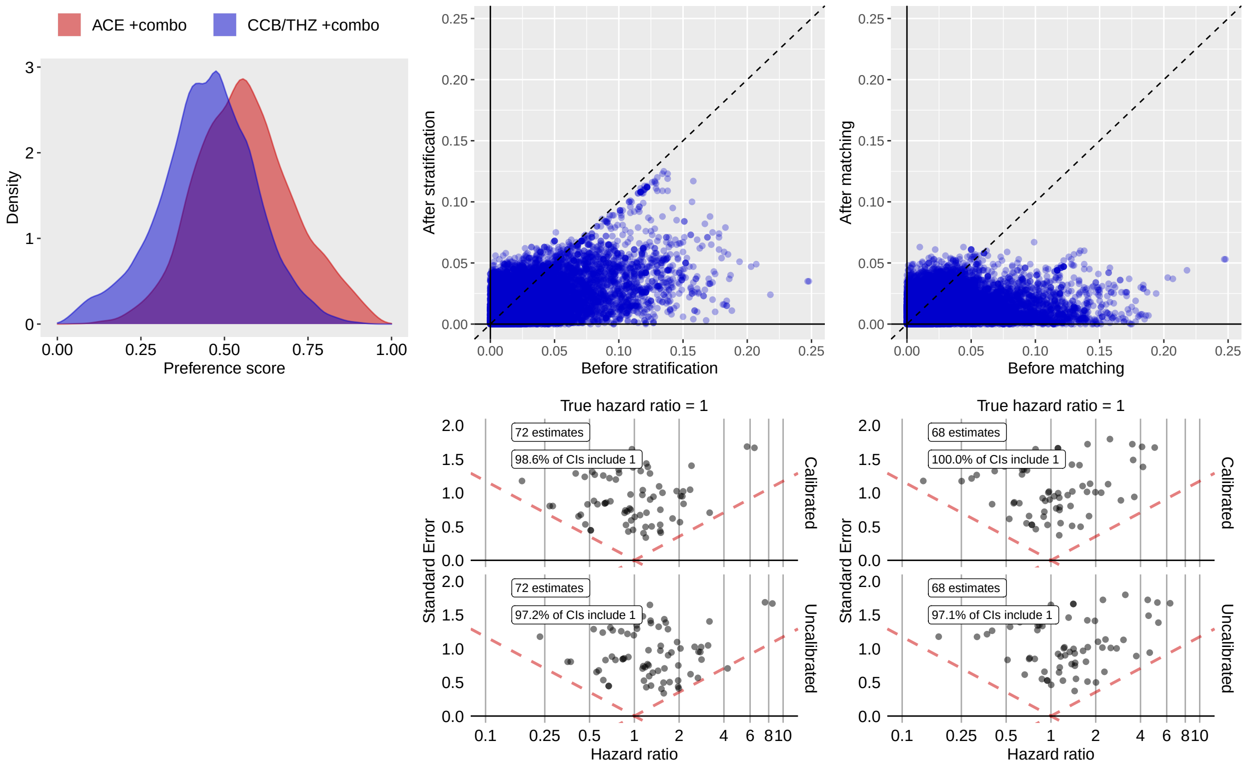

Supplementary Table 163: Most extreme patient characteristics balance diagnostics after stratification for ACE +combo vs CCB/THZ +combo in the CUIMC data source.

Characteristic (total count = 32546)	Before stratification			After stratification		
	T (%)	C (%)	SDf	T (%)	C (%)	SDf
condition_era group during day -180 through 0 days relative to index: Third trimester pregnancy	-0.1	1.0	-0.14	-0.1	0.8	-0.12
condition_era group during day -30 through 0 days relative to index: Complication of pregnancy, childbirth and/or the puerperium	-0.1	1.0	-0.14	-0.1	0.8	-0.12
procedure_occurrence during day -180 through 0 days relative to index: Ultrasound, pregnant uterus, real time with image documentation, follow-up (eg, re-evaluation of fetal size by measuring standard growth parameters and amniotic fluid volume, re-evaluation of organ system(s) suspected or confirmed to be abnormal on a prev	-0.1	0.9	-0.13	-0.1	0.8	-0.12
condition_era group during day -180 through 0 days relative to index: Maternal AND/OR fetal condition affecting labor AND/OR delivery	-0.1	0.9	-0.13	-0.1	0.8	-0.12
condition_era group during day -180 through 0 days relative to index: Hypertension AND/OR vomiting complicating pregnancy childbirth AND/OR puerperium	-0.1	1.1	-0.14	-0.1	0.9	-0.12
drug_era group during day -180 through 0 days relative to index: Witch Hazel	-0.1	0.9	-0.13	-0.1	0.7	-0.12

(Continued)	T (%)	C (%)	SDf	T (%)	C (%)	SDf
procedure_occurrence during day -180 through 0 days relative to index: Ultrasound, transplanted kidney, real time and duplex Doppler with image documentation	0.1	1.6	-0.16	0.3	1.3	-0.12
measurement during day -180 through 0 days relative to index: Streptococcus agalactiae DNA [Presence] in Unspecified specimen by NAA with probe detection	-0.1	1.0	-0.13	-0.1	0.9	-0.12
condition_era group during day -180 through 0 days relative to index: Third trimester pregnancy less than 36 weeks	-0.1	0.8	-0.13	-0.1	0.7	-0.12
condition_era group during day -30 through 0 days relative to index: Finding related to pregnancy	0.1	1.2	-0.13	0.1	1.0	-0.12

Supplementary Table 164: Most extreme patient characteristics balance diagnostics after matching for ACE +combo vs CCB/THZ +combo in the CUIMC data source.

	Before matching			After matching		
Characteristic (total count = 31930)	T (%)	C (%)	SDf	T (%)	C (%)	SDf
drug_era group during day -30 through 0 days relative to index: patiromer	-0.1	0.5	-0.10	-0.1	0.3	-0.07
condition_era group during day -180 through 0 days relative to index: Hypertrophic cardiomyopathy	0.5	0.5	0.01	0.4	0.9	-0.06
procedure_occurrence during day -30 through 0 days relative to index: Catheter placement in coronary artery(s) for coronary angiography, including intraprocedural injection(s) for coronary angiography, imaging supervision and interpretation; with left heart catheterization including intraprocedural injection(s) for left ven	0.3	0.5	-0.04	0.3	0.7	-0.06
condition_era group during day -30 through 0 days relative to index: Pain in thoracic spine	0.2	0.0	0.05	0.2	0.0	0.06
procedure_occurrence during day -180 through 0 days relative to index: Computed tomography, maxillofacial area; without contrast material	0.8	0.6	0.02	0.8	0.3	0.06
condition_era group during day -30 through 0 days relative to index: Musculoskeletal chest pain	0.2	0.0	0.05	0.2	0.0	0.06
condition_era group during day -30 through 0 days relative to index: Thoracic back pain	0.2	0.0	0.05	0.2	0.0	0.06
condition_era group during day -30 through 0 days relative to index: Pain in spine	0.2	0.0	0.05	0.2	0.0	0.06
condition_era group during day -30 through 0 days relative to index: Complication of pregnancy, childbirth and/or the puerperium	-0.1	1.0	-0.14	-0.1	0.2	-0.06
condition_era group during day -180 through 0 days relative to index: Acute disease of eye	-0.1	0.3	-0.06	-0.1	0.2	-0.06

Supplementary Figure 51: Preference score distributions, propensity score stratified (middle column) and matched (right column) covariate balance and negative control error distributions (bottom row) for ACE mono vs CCB mono in the CUIMC data source.

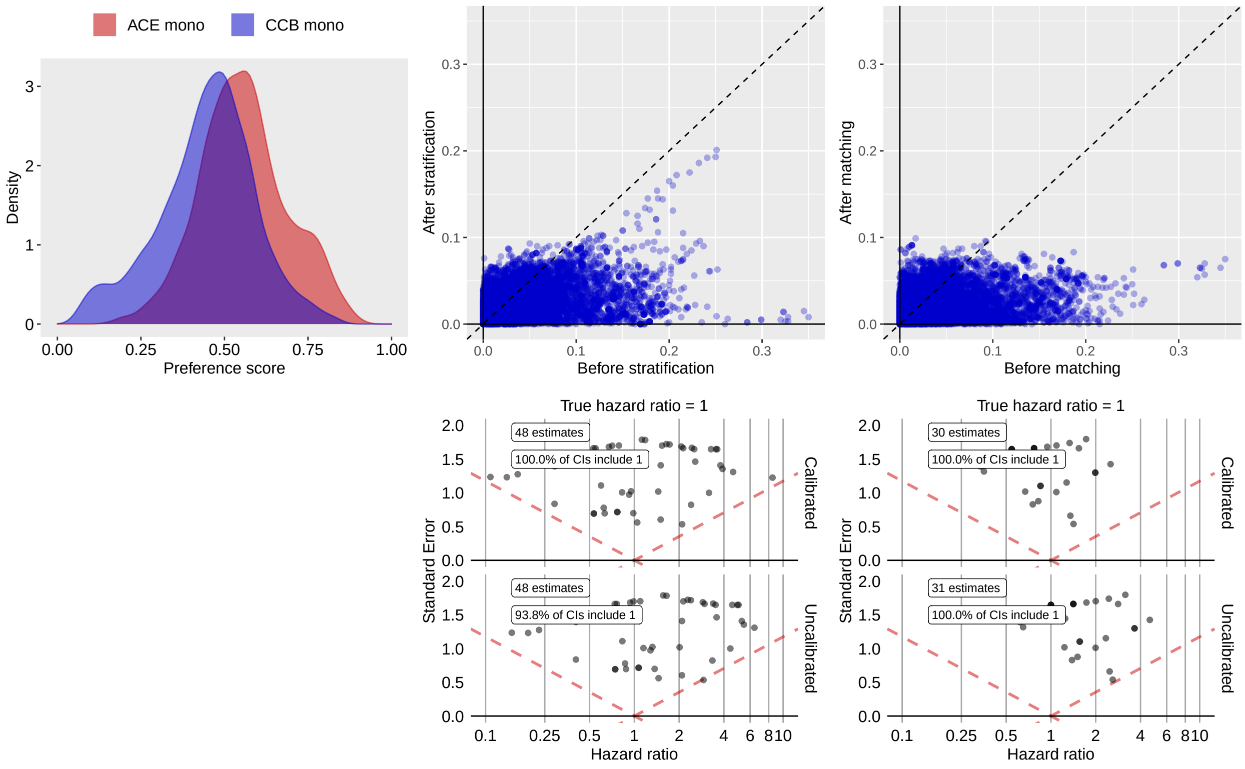

Supplementary Table 165: Most extreme patient characteristics balance diagnostics after stratification for ACE mono vs CCB mono in the CUIMC data source.

Characteristic (total count = 24476)	Before stratification			After stratification		
	T (%)	C (%)	SDf	T (%)	C (%)	SDf
condition_era group during day -180 through 0 days relative to index: Complication of pregnancy, childbirth and/or the puerperium	-0.2	3.2	-0.25	-0.2	2.2	-0.20
condition_era group during day -180 through 0 days relative to index: Finding related to pregnancy	-0.2	3.5	-0.25	0.2	2.5	-0.19
condition_era group during day -180 through 0 days relative to index: Hypertension AND/OR vomiting complicating pregnancy childbirth AND/OR puerperium	-0.2	2.9	-0.24	-0.2	2.1	-0.19
condition_era group during day -30 through 0 days relative to index: Finding related to pregnancy	-0.2	2.8	-0.23	-0.2	2.0	-0.19
condition_era group during day -180 through 0 days relative to index: Pregnancy-induced hypertension	-0.2	2.5	-0.22	-0.2	1.8	-0.17
measurement during day -180 through 0 days relative to index: Streptococcus agalactiae DNA [Presence] in Unspecified specimen by NAA with probe detection	-0.2	2.2	-0.21	-0.2	1.6	-0.17
drug_era group during day -180 through 0 days relative to index: POSTERIOR PITUITARY LOBE HORMONES	-0.2	2.3	-0.20	-0.2	1.6	-0.17

(Continued)	T (%)	C (%)	SDf	T (%)	C (%)	SDf
condition_era group during day -180 through 0 days relative to index: Pregnant	-0.2	2.1	-0.20	-0.2	1.5	-0.16
observation during day -180 through 0 days relative to index: Routine antenatal care	-0.2	1.8	-0.19	-0.2	1.3	-0.15
drug_era group during day -180 through 0 days relative to index: PITUITARY AND HYPOTHALAMIC HORMONES AND ANALOGUES	0.3	2.4	-0.19	0.3	1.7	-0.14

Supplementary Table 166: Most extreme patient characteristics balance diagnostics after matching for ACE mono vs CCB mono in the CUIMC data source.

	Before matching			After matching		
Characteristic (total count = 23410)	T (%)	C (%)	SDf	T (%)	C (%)	SDf
measurement during day -180 through 0 days relative to index: Amphetamine+Methamphetamine [Presence] in Urine	1.7	1.5	0.02	1.9	0.8	0.10
condition_era group during day -180 through 0 days relative to index: Non-neoplastic nevus	1.5	0.6	0.09	1.6	0.6	0.10
condition_era group during day -180 through 0 days relative to index: Precapillary pulmonary hypertension	-0.2	0.6	-0.09	-0.2	0.6	-0.10
measurement during day -180 through 0 days relative to index: Barbiturates [Presence] in Urine by Screen method	1.8	1.6	0.01	2.0	0.9	0.09
measurement during day -180 through 0 days relative to index: Benzodiazepines [Presence] in Urine by Screen method	1.8	1.6	0.01	2.0	0.9	0.09
measurement during day -180 through 0 days relative to index: Opiates [Presence] in Urine by Screen method	1.8	1.6	0.01	2.0	0.9	0.09
measurement during day -180 through 0 days relative to index: Cocaine [Presence] in Urine by Screen method	1.8	1.6	0.01	2.0	0.9	0.09
measurement during day -180 through 0 days relative to index: Methadone [Presence] in Urine by Screen method	1.8	1.6	0.01	2.0	0.9	0.09
condition_era group during day -180 through 0 days relative to index: Corneal degeneration	0.4	-0.1	0.08	0.5	-0.1	0.09
condition_era group during day -180 through 0 days relative to index: Pulmonary arterial hypertension	-0.2	0.6	-0.09	-0.2	0.5	-0.09

Supplementary Figure 52: Preference score distributions, propensity score stratified (middle column) and matched (right column) covariate balance and negative control error distributions (bottom row) for ACE +combo vs CCB +combo in the CUIMC data source.

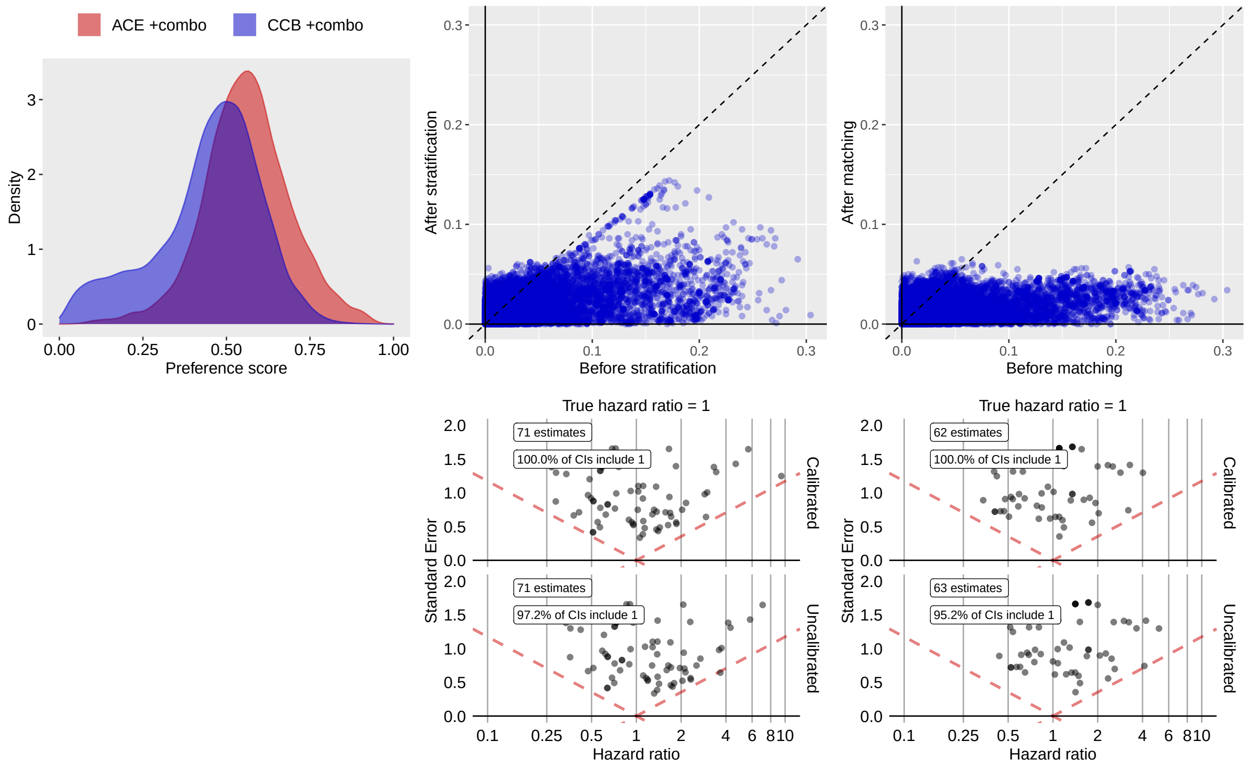

Supplementary Table 167: Most extreme patient characteristics balance diagnostics after stratification for ACE +combo vs CCB +combo in the CUIMC data source.

	Before stratification			After stratification		
Characteristic (total count = 31882)	T (%)	C (%)	SDf	T (%)	C (%)	SDf
condition_era group during day -30 through 0 days relative to index: Complication of pregnancy, childbirth and/or the puerperium	-0.1	1.5	-0.17	-0.1	1.1	-0.14
procedure_occurrence during day -180 through 0 days relative to index: Ultrasound, pregnant uterus, real time with image documentation, follow-up (eg, re-evaluation of fetal size by measuring standard growth parameters and amniotic fluid volume, re-evaluation of organ system(s) suspected or confirmed to be abnormal on a prev	-0.1	1.4	-0.17	-0.1	1.0	-0.14
condition_era group during day -180 through 0 days relative to index: Hypertension AND/OR vomiting complicating pregnancy childbirth AND/OR puerperium	-0.1	1.7	-0.18	-0.1	1.2	-0.14
condition_era group during day -180 through 0 days relative to index: Maternal AND/OR fetal condition affecting labor AND/OR delivery	-0.1	1.4	-0.16	-0.1	1.0	-0.14
condition_era group during day -180 through 0 days relative to index: Complication of pregnancy, childbirth and/or the puerperium	0.1	1.8	-0.18	0.1	1.3	-0.14
drug_era group during day -180 through 0 days relative to index: Witch Hazel	-0.1	1.3	-0.16	-0.1	0.9	-0.14

(Continued)	T (%)	C (%)	SDf	T (%)	C (%)	SDf
condition_era group during day -180 through 0 days relative to index: Third trimester pregnancy less than 36 weeks	-0.1	1.3	-0.16	-0.1	0.9	-0.14
condition_era group during day -180 through 0 days relative to index: Third trimester pregnancy	-0.1	1.5	-0.17	-0.1	1.1	-0.14
procedure_occurrence during day -180 through 0 days relative to index: Ultrasound, transplanted kidney, real time and duplex Doppler with image documentation	0.1	2.2	-0.20	0.3	1.6	-0.13
measurement during day -180 through 0 days relative to index: Streptococcus agalactiae DNA [Presence] in Unspecified specimen by NAA with probe detection	-0.1	1.5	-0.17	0.1	1.1	-0.13

Supplementary Table 168: Most extreme patient characteristics balance diagnostics after matching for ACE +combo vs CCB +combo in the CUIMC data source.

	Before matching			After matching		
Characteristic (total count = 31187)	T (%)	C (%)	SDf	T (%)	C (%)	SDf
condition_era group during day -30 through 0 days relative to index: Neuropathy of lower limb	0.7	0.3	0.06	0.7	0.2	0.07
procedure_occurrence during day -180 through 0 days relative to index: Computed tomography, maxillofacial area; without contrast material	0.7	0.6	0.01	0.8	0.3	0.06
measurement during day -180 through 0 days relative to index: Hepatitis B virus core IgM Ab [Presence] in Serum or Plasma by Immunoassay	-0.1	0.3	-0.07	-0.1	0.3	-0.06
condition_era group during day -180 through 0 days relative to index: Laceration of upper limb	0.3	0.1	0.05	0.3	0.0	0.06
condition_era group during day -180 through 0 days relative to index: Open wound of upper limb	0.4	0.2	0.05	0.4	0.1	0.06
measurement below normal range during day -180 through 0 days relative to index: Iron binding capacity.unsaturated [Mass/volume] in Serum or Plasma	0.5	1.3	-0.09	0.5	0.2	0.06
procedure_occurrence during day -30 through 0 days relative to index: Catheter placement in coronary artery(s) for coronary angiography, including intraprocedural injection(s) for coronary angiography, imaging supervision and interpretation; with left heart catheterization including intraprocedural injection(s) for left ven	0.2	0.7	-0.07	0.2	0.6	-0.06
condition_era group during day -180 through 0 days relative to index: EKG ST segment changes	0.9	0.3	0.07	0.7	0.3	0.06
condition_era group during day -180 through 0 days relative to index: ST segment elevation	0.9	0.3	0.07	0.7	0.3	0.06
condition_era group during day -180 through 0 days relative to index: Acute ST segment elevation myocardial infarction	0.9	0.3	0.07	0.7	0.3	0.06

Supplementary Figure 53: Preference score distributions, propensity score stratified (middle column) and matched (right column) covariate balance and negative control error distributions (bottom row) for ACE mono vs THZ mono in the CUIMC data source.

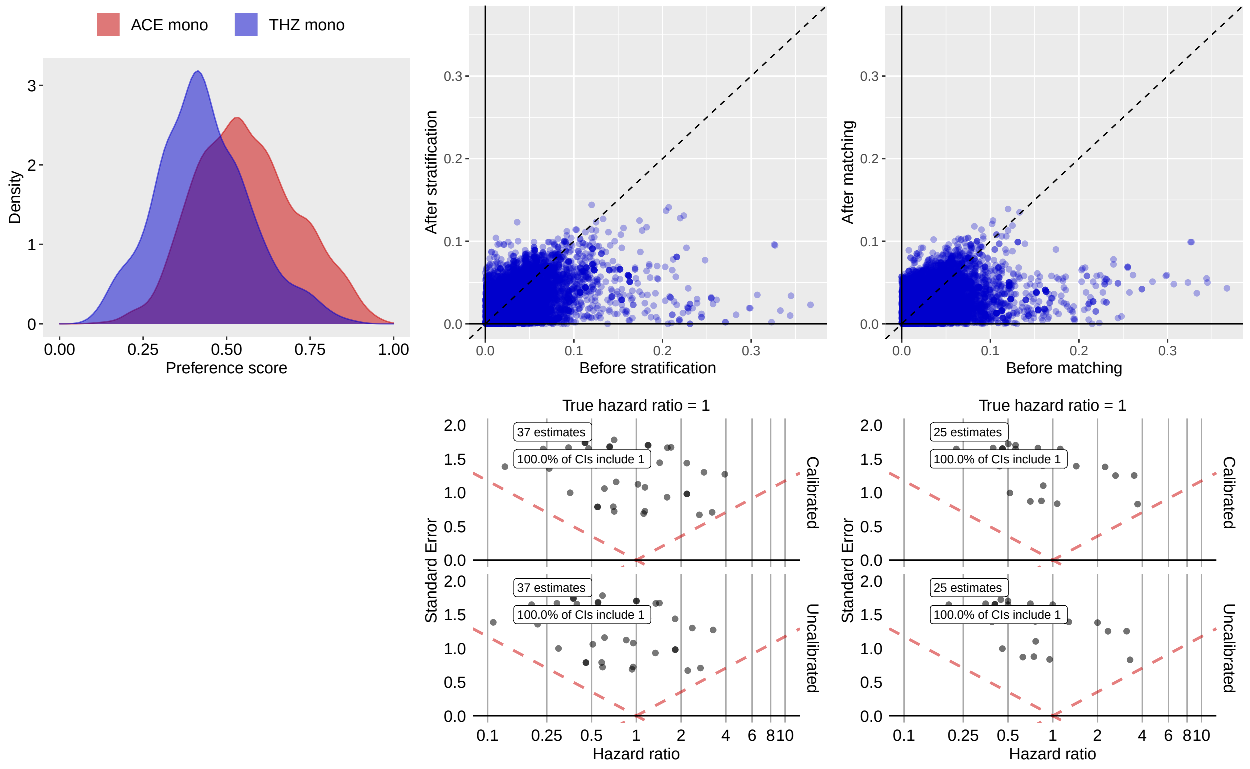

Supplementary Table 169: Most extreme patient characteristics balance diagnostics after stratification for ACE mono vs THZ mono in the CUIMC data source.

Characteristic (total count = 20510)	Before stratification			After stratification		
	T (%)	C (%)	SDf	T (%)	C (%)	SDf
condition_era group during day -180 through 0 days relative to index: Precapillary pulmonary hypertension	-0.2	0.9	-0.12	-0.2	1.2	-0.14
drug_era group during day -30 through 0 days relative to index: Insulins and analogues for injection, fast-acting	2.9	0.3	0.21	2.2	0.6	0.14
drug_era group during day -30 through 0 days relative to index: Insulins and analogues for injection, intermediate- or long-acting combined with fast-acting	2.9	0.3	0.20	2.2	0.6	0.14
drug_era group during day -180 through 0 days relative to index: Insulins and analogues for injection, fast-acting	3.9	0.6	0.22	2.9	1.1	0.13
drug_era group during day -180 through 0 days relative to index: Insulins and analogues for injection, intermediate- or long-acting combined with fast-acting	3.8	0.6	0.22	2.9	1.1	0.13
drug_era group during day -180 through 0 days relative to index: potassium citrate	-0.2	1.2	-0.13	-0.2	1.1	-0.13

(Continued)	T (%)	C (%)	SDf	T (%)	C (%)	SDf
measurement below normal range during day -180 through 0 days relative to index: Carbon dioxide, total [Moles/volume] in Serum or Plasma	2.4	0.4	0.17	2.1	0.6	0.12
drug_era group during day -180 through 0 days relative to index: Vancomycin	1.4	-0.3	0.12	1.1	-0.3	0.12
procedure_occurrence during day -180 through 0 days relative to index: Moderate sedation services provided by the same physician or other qualified health care professional performing the diagnostic or therapeutic service that the sedation supports, requiring the presence of an independent trained observer to assist in the m	0.9	1.3	-0.04	0.7	2.2	-0.12
drug_era group during day 0 through 0 days relative to index: Insulins and analogues for injection, fast-acting	2.4	0.3	0.18	1.8	0.6	0.12

Supplementary Table 170: Most extreme patient characteristics balance diagnostics after matching for ACE mono vs THZ mono in the CUIMC data source.

	Before matching			After matching		
Characteristic (total count = 19656)	T (%)	C (%)	SDf	T (%)	C (%)	SDf
condition_era group during day -180 through 0 days relative to index: Precapillary pulmonary hypertension	-0.2	0.9	-0.12	-0.1	1.1	-0.14
drug_era group during day -180 through 0 days relative to index: potassium citrate	-0.2	1.2	-0.13	-0.1	1.2	-0.14
age group: 20-24	0.9	-0.3	0.10	1.0	-0.4	0.12
condition_era group during day -180 through 0 days relative to index: Pulmonary arterial hypertension	-0.2	0.7	-0.10	-0.1	0.9	-0.12
drug_era group during day -30 through 0 days relative to index: potassium citrate	-0.2	1.0	-0.12	-0.1	1.0	-0.12
condition_era group during day -180 through 0 days relative to index: Malaise	0.6	1.4	-0.08	0.4	1.5	-0.12
race = Other Pacific Islander	1.1	0.3	0.09	1.4	-0.4	0.11
drug_era group during day 0 through 0 days relative to index: potassium citrate	-0.2	0.9	-0.12	-0.1	0.9	-0.11
drug_era group during day -180 through 0 days relative to index: POTASSIUM	0.3	1.2	-0.11	0.3	1.2	-0.11
drug_era group during day -180 through 0 days relative to index: Potassium	0.3	1.2	-0.11	0.3	1.2	-0.11

Supplementary Figure 54: Preference score distributions, propensity score stratified (middle column) and matched (right column) covariate balance and negative control error distributions (bottom row) for ACE +combo vs THZ +combo in the CUIMC data source.

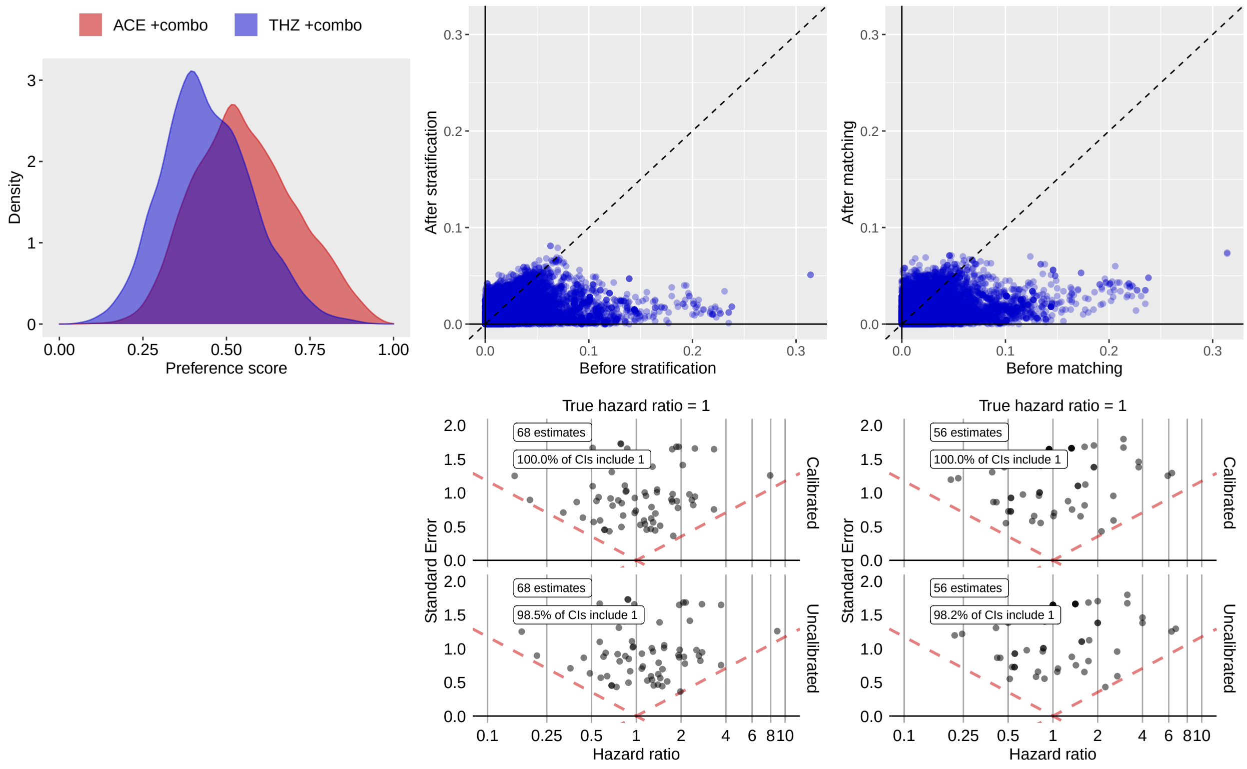

Supplementary Table 171: Most extreme patient characteristics balance diagnostics after stratification for ACE +combo vs THZ +combo in the CUIMC data source.

	Before stratification			After stratification		
Characteristic (total count = 31377)	T (%)	C (%)	SDf	T (%)	C (%)	SDf
condition_era group during day -30 through 0 days relative to index: Cardiogenic shock	0.2	0.6	-0.06	0.1	0.6	-0.08
condition_era group during day -30 through 0 days relative to index: Disease affecting entire cardiovascular system	0.2	0.6	-0.06	0.1	0.6	-0.08
drug_era group during day -30 through 0 days relative to index: Dobutamine	0.1	0.6	-0.07	0.1	0.6	-0.08
drug_era group during day 0 through 0 days relative to index: Calcium Gluconate	0.1	0.5	-0.07	0.1	0.5	-0.07
age group: 20-24	0.4	0.1	0.06	0.5	0.1	0.07
condition_era group during day -30 through 0 days relative to index: Schizophrenia	0.3	-0.1	0.07	0.3	-0.1	0.07
measurement during day -30 through 0 days relative to index: cycloSPORINE [Mass/volume] in Blood	-0.1	0.3	-0.07	-0.1	0.3	-0.07
drug_era group during day -180 through 0 days relative to index: Dobutamine	0.3	0.7	-0.05	0.3	0.7	-0.07

(Continued)	T (%)	C (%)	SDf	T (%)	C (%)	SDf
measurement during day -30 through 0 days relative to index: Fractional oxyhemoglobin in Arterial blood	0.3	0.9	-0.07	0.3	0.9	-0.07
measurement during day -30 through 0 days relative to index: Base deficit in Arterial blood	0.3	0.9	-0.07	0.3	0.9	-0.07

Supplementary Table 172: Most extreme patient characteristics balance diagnostics after matching for ACE +combo vs THZ +combo in the CUIMC data source.

	Before matching			After matching		
Characteristic (total count = 30114)	T (%)	C (%)	SDf	T (%)	C (%)	SDf
gender = MALE	55.5	40.0	0.31	48.8	52.5	-0.07
gender = FEMALE	44.5	60.0	-0.31	51.1	47.5	0.07
procedure_occurrence during day -180 through 0 days relative to index: Ultrasound, transplanted kidney, real time and duplex Doppler with image documentation	0.3	0.6	-0.05	0.2	0.6	-0.07
condition_era group during day -180 through 0 days relative to index: Malignant epithelial neoplasm of skin	0.6	0.3	0.05	0.7	0.2	0.07
condition_era group during day -180 through 0 days relative to index: Basal cell carcinoma of skin	0.6	0.3	0.05	0.7	0.2	0.07
condition_era group during day -180 through 0 days relative to index: Malignant basal cell neoplasm of skin	0.6	0.3	0.05	0.7	0.2	0.07
age group: 20-24	0.4	0.1	0.06	0.5	0.1	0.07
condition_era group during day -180 through 0 days relative to index: Ischemic heart disease	9.8	6.4	0.12	6.0	7.8	-0.07
drug_era group during day -30 through 0 days relative to index: Sodium polystyrene sulfonate	0.3	0.5	-0.02	0.2	0.6	-0.07
drug_era group during day -180 through 0 days relative to index: Sodium polystyrene sulfonate	0.8	0.9	-0.01	0.5	1.1	-0.07

Supplementary Figure 55: Preference score distributions, propensity score stratified (middle column) and matched (right column) covariate balance and negative control error distributions (bottom row) for ARB mono vs CCB/THZ mono in the CUIMC data source.

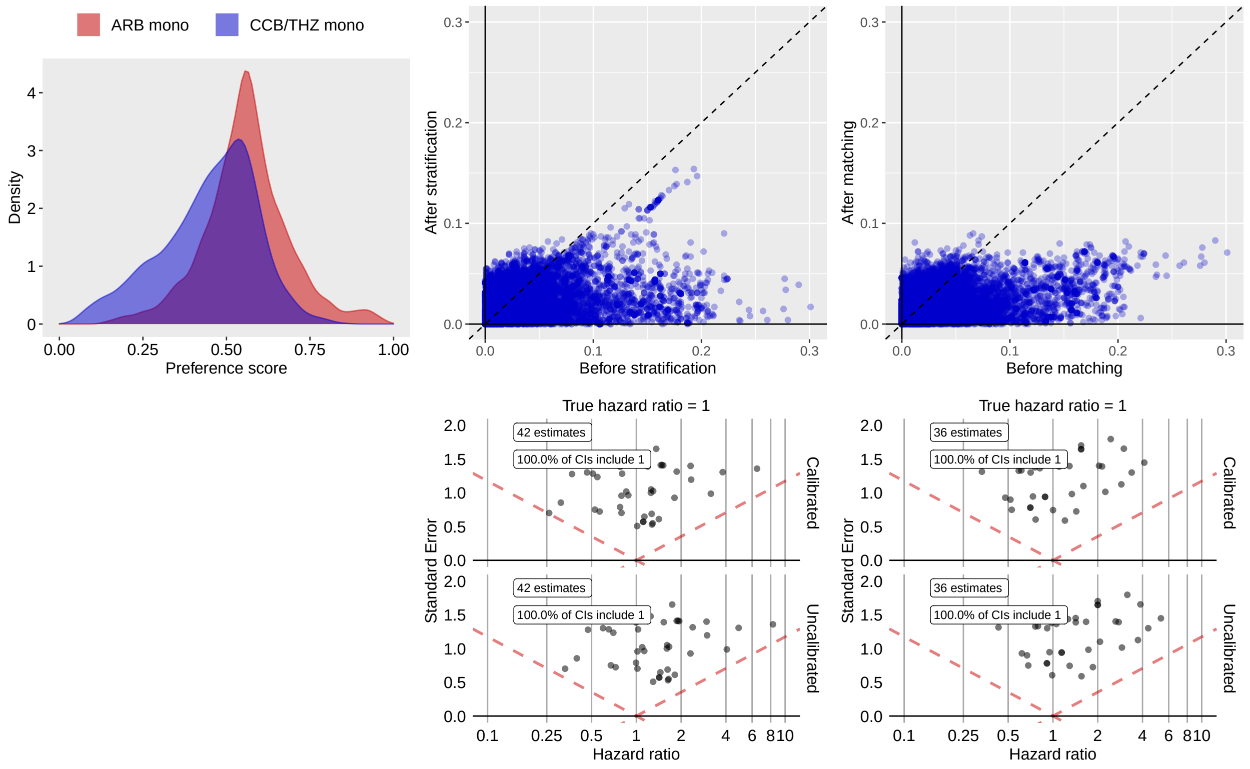

Supplementary Table 173: Most extreme patient characteristics balance diagnostics after stratification for ARB mono vs CCB/THZ mono in the CUIMC data source.

Characteristic (total count = 25708)	Before stratification			After stratification		
	T (%)	C (%)	SDf	T (%)	C (%)	SDf
condition_era group during day -180 through 0 days relative to index: Hypertension AND/OR vomiting complicating pregnancy childbirth AND/OR puerperium	-0.2	1.9	-0.19	-0.2	1.5	-0.15
condition_era group during day -30 through 0 days relative to index: Finding related to pregnancy	-0.2	1.9	-0.18	-0.2	1.5	-0.15
condition_era group during day -180 through 0 days relative to index: Complication of pregnancy, childbirth and/or the puerperium	-0.2	2.1	-0.20	0.2	1.6	-0.15
condition_era group during day -180 through 0 days relative to index: Finding related to pregnancy	0.2	2.4	-0.19	0.4	1.8	-0.14
condition_era group during day -180 through 0 days relative to index: Pregnancy-induced hypertension	-0.2	1.6	-0.18	-0.2	1.2	-0.14
procedure_occurrence during day -180 through 0 days relative to index: Ultrasound, pregnant uterus, real time with image documentation, follow-up (eg, re-evaluation of fetal size by measuring standard growth parameters and amniotic fluid volume, re-evaluation of organ system(s) suspected or confirmed to be abnormal on a prev	-0.2	1.6	-0.17	-0.2	1.2	-0.14

(Continued)	T (%)	C (%)	SDf	T (%)	C (%)	SDf
condition_era group during day -180 through 0 days relative to index: Third trimester pregnancy	-0.2	1.6	-0.17	-0.2	1.2	-0.13
condition_era group during day -180 through 0 days relative to index: Pregnant	-0.2	1.4	-0.16	-0.2	1.1	-0.13
drug_era group during day -180 through 0 days relative to index: Witch Hazel	-0.2	1.4	-0.16	-0.2	1.1	-0.13
drug_era group during day -180 through 0 days relative to index: Lanolin	-0.2	1.4	-0.16	-0.2	1.0	-0.12

Supplementary Table 174: Most extreme patient characteristics balance diagnostics after matching for ARB mono vs CCB/THZ mono in the CUIMC data source.

	Before matching			After matching		
Characteristic (total count = 25015)	T (%)	C (%)	SDf	T (%)	C (%)	SDf
condition_era group during day -180 through 0 days relative to index: Precapillary pulmonary hypertension	0.2	0.7	-0.07	-0.2	0.8	-0.09
condition_era group during day -180 through 0 days relative to index: Pulmonary arterial hypertension	0.2	0.6	-0.06	-0.2	0.7	-0.09
condition_era group during day -180 through 0 days relative to index: Migraine without aura	1.0	0.4	0.07	1.1	0.4	0.09
measurement during day -30 through 0 days relative to index: Thyroxine (T4) [Mass/volume] in Serum or Plasma	1.6	0.9	0.07	1.7	0.8	0.08
measurement during day -180 through 0 days relative to index: Heart rate	14.5	26.0	-0.29	15.2	12.4	0.08
measurement during day -180 through 0 days relative to index: Hematocrit [Volume Fraction] of Blood	24.5	33.8	-0.21	24.7	21.2	0.08
observation during day -180 through 0 days relative to index: H/O: drug allergy	1.6	1.7	-0.01	1.7	0.8	0.08
drug_era group during day -180 through 0 days relative to index: Calcium Chloride	3.0	6.0	-0.15	3.3	2.0	0.08
measurement during day -30 through 0 days relative to index: Platelet mean volume [Entitic volume] in Blood by Automated count	16.1	22.9	-0.17	16.5	13.7	0.08
measurement during day -30 through 0 days relative to index: Anion gap in Serum or Plasma	18.5	25.7	-0.17	18.8	15.8	0.08

Supplementary Figure 56: Preference score distributions, propensity score stratified (middle column) and matched (right column) covariate balance and negative control error distributions (bottom row) for ARB +combo vs CCB/THZ +combo in the CUIMC data source.

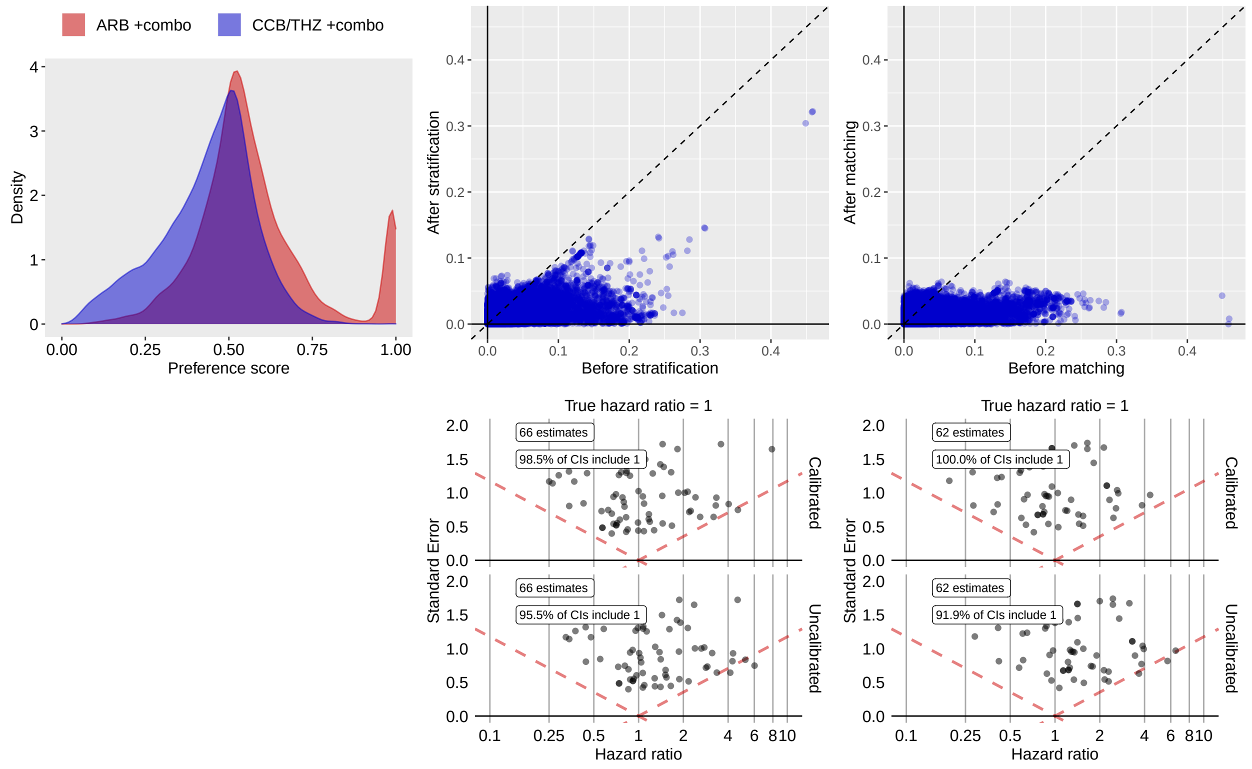

Supplementary Table 175: Most extreme patient characteristics balance diagnostics after stratification for ARB +combo vs CCB/THZ +combo in the CUIMC data source.

	Before stratification			After stratification		
Characteristic (total count = 32448)	T (%)	C (%)	SDf	T (%)	C (%)	SDf
drug_era group during day 0 through 0 days relative to index: sacubitril	9.6	0.0	0.46	5.0	0.0	0.32
drug_era group during day -30 through 0 days relative to index: sacubitril	9.6	0.0	0.46	5.0	0.0	0.32
drug_era group during day -180 through 0 days relative to index: sacubitril	9.7	0.2	0.45	5.1	0.2	0.30
condition_era group during day -180 through 0 days relative to index: Systolic heart failure	11.5	3.6	0.31	7.3	3.9	0.15
condition_era group during day -180 through 0 days relative to index: Systolic dysfunction	11.7	3.6	0.31	7.3	4.0	0.14
condition_era group during day -30 through 0 days relative to index: Systolic heart failure	6.7	1.9	0.24	4.3	2.0	0.13
condition_era group during day -30 through 0 days relative to index: Systolic dysfunction	6.8	1.9	0.24	4.3	2.0	0.13
condition_era group during day -30 through 0 days relative to index: Complication of pregnancy, childbirth and/or the puerperium	-0.1	1.2	-0.14	-0.1	0.9	-0.13

(Continued)	T (%)	C (%)	SDf	T (%)	C (%)	SDf
condition_era group during day -30 through 0 days relative to index: Hypertension AND/OR vomiting complicating pregnancy childbirth AND/OR puerperium	-0.1	1.1	-0.14	-0.1	0.8	-0.13
condition_era group during day -180 through 0 days relative to index: Chronic systolic heart failure	9.9	3.0	0.28	6.0	3.3	0.13

Supplementary Table 176: Most extreme patient characteristics balance diagnostics after matching for ARB +combo vs CCB/THZ +combo in the CUIMC data source.

	Before matching			After matching		
Characteristic (total count = 31581)	T (%)	C (%)	SDf	T (%)	C (%)	SDf
condition_era group during day -30 through 0 days relative to index: Complication of pregnancy, childbirth and/or the puerperium	-0.1	1.2	-0.14	-0.1	0.3	-0.06
drug_era group during day -30 through 0 days relative to index: Lovastatin	0.1	0.4	-0.05	0.1	0.4	-0.06
procedure_occurrence during day -180 through 0 days relative to index: Ultrasound, pregnant uterus, real time with image documentation, follow-up (eg, re-evaluation of fetal size by measuring standard growth parameters and amniotic fluid volume, re-evaluation of organ system(s) suspected or confirmed to be abnormal on a prev	-0.1	1.1	-0.14	-0.1	0.3	-0.06
drug_era group during day -180 through 0 days relative to index: Lovastatin	0.2	0.4	-0.05	0.1	0.5	-0.06
condition_era group during day -180 through 0 days relative to index: Hypertension AND/OR vomiting complicating pregnancy childbirth AND/OR puerperium	-0.1	1.3	-0.15	-0.1	0.3	-0.06
condition_era group during day -180 through 0 days relative to index: Precapillary pulmonary hypertension	1.6	1.5	0.01	0.8	1.5	-0.06
condition_era group during day -180 through 0 days relative to index: Pregnant	-0.1	1.0	-0.13	-0.1	0.3	-0.06
drug_era group during day 0 through 0 days relative to index: Lovastatin	0.1	0.3	-0.05	0.1	0.4	-0.06
condition_era group during day -30 through 0 days relative to index: Testicular hypofunction	0.3	0.1	0.05	0.3	0.1	0.06
condition_era group during day -180 through 0 days relative to index: Proliferative retinopathy with diabetes mellitus	0.1	0.4	-0.07	-0.1	0.3	-0.06

Supplementary Figure 57: Preference score distributions, propensity score stratified (middle column) and matched (right column) covariate balance and negative control error distributions (bottom row) for ARB mono vs CCB mono in the CUIMC data source.

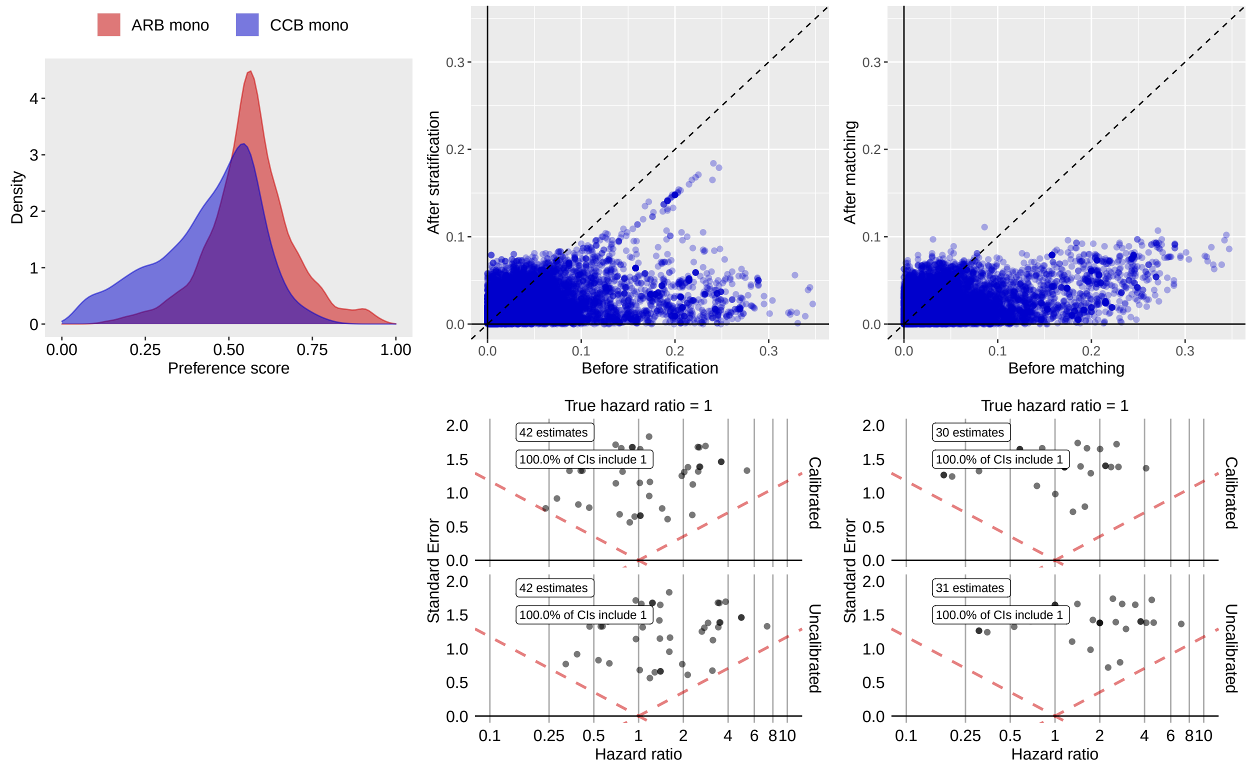

Supplementary Table 177: Most extreme patient characteristics balance diagnostics after stratification for ARB mono vs CCB mono in the CUIMC data source.

Characteristic (total count = 24829)	Before stratification			After stratification		
	T (%)	C (%)	SDf	T (%)	C (%)	SDf
condition_era group during day -180 through 0 days relative to index: Hypertension AND/OR vomiting complicating pregnancy childbirth AND/OR puerperium	-0.2	2.9	-0.24	-0.2	2.0	-0.18
condition_era group during day -180 through 0 days relative to index: Complication of pregnancy, childbirth and/or the puerperium	-0.2	3.2	-0.25	0.2	2.1	-0.18
condition_era group during day -30 through 0 days relative to index: Finding related to pregnancy	-0.2	2.8	-0.23	0.2	2.0	-0.17
condition_era group during day -180 through 0 days relative to index: Pregnancy-induced hypertension	-0.2	2.5	-0.22	-0.2	1.7	-0.17
condition_era group during day -180 through 0 days relative to index: Finding related to pregnancy	0.2	3.5	-0.24	0.4	2.4	-0.17
procedure_occurrence during day -180 through 0 days relative to index: Ultrasound, pregnant uterus, real time with image documentation, follow-up (eg, re-evaluation of fetal size by measuring standard growth parameters and amniotic fluid volume, re-evaluation of organ system(s) suspected or confirmed to be abnormal on a prev	-0.2	2.4	-0.22	-0.2	1.6	-0.17

(Continued)	T (%)	C (%)	SDf	T (%)	C (%)	SDf
condition_era group during day -180 through 0 days relative to index: Third trimester pregnancy	-0.2	2.5	-0.21	-0.2	1.6	-0.16
condition_era group during day -180 through 0 days relative to index: Pregnant	-0.2	2.1	-0.20	-0.2	1.4	-0.15
drug_era group during day -180 through 0 days relative to index: Witch Hazel	-0.2	2.2	-0.21	-0.2	1.4	-0.15
drug_era group during day -180 through 0 days relative to index: Lanolin	-0.2	2.1	-0.20	-0.2	1.4	-0.15

Supplementary Table 178: Most extreme patient characteristics balance diagnostics after matching for ARB mono vs CCB mono in the CUIMC data source.

	Before matching			After matching		
Characteristic (total count = 23846)	T (%)	C (%)	SDf	T (%)	C (%)	SDf
condition_era group during day -180 through 0 days relative to index: Migraine without aura	1.0	0.3	0.09	1.0	0.2	0.11
measurement during day -180 through 0 days relative to index: Hematocrit [Volume Fraction] of Blood	24.5	36.9	-0.27	26.1	21.5	0.11
measurement during day -180 through 0 days relative to index: Heart rate	14.5	28.4	-0.34	16.7	13.1	0.10
measurement during day -180 through 0 days relative to index: Platelets [#/volume] in Blood	23.2	35.5	-0.28	24.6	20.6	0.10
observation during day -180 through 0 days relative to index: H/O: drug allergy	1.6	2.0	-0.03	1.8	0.7	0.10
drug_era group during day -180 through 0 days relative to index: I.V. SOLUTION ADDITIVES	10.2	18.9	-0.25	11.4	8.5	0.10
drug_era group during day -180 through 0 days relative to index: Electrolyte solutions	10.2	18.9	-0.25	11.4	8.5	0.10
measurement during day -180 through 0 days relative to index: Leukocytes [#/volume] in Urine	8.4	13.9	-0.17	9.3	6.7	0.10
measurement during day -180 through 0 days relative to index: Respiratory rate	13.2	26.7	-0.34	15.2	11.9	0.10
drug_era group during day -180 through 0 days relative to index: BLOOD SUBSTITUTES AND PERFUSION SOLUTIONS	10.7	19.7	-0.25	11.9	9.0	0.09

Supplementary Figure 58: Preference score distributions, propensity score stratified (middle column) and matched (right column) covariate balance and negative control error distributions (bottom row) for ARB +combo vs CCB +combo in the CUIMC data source.

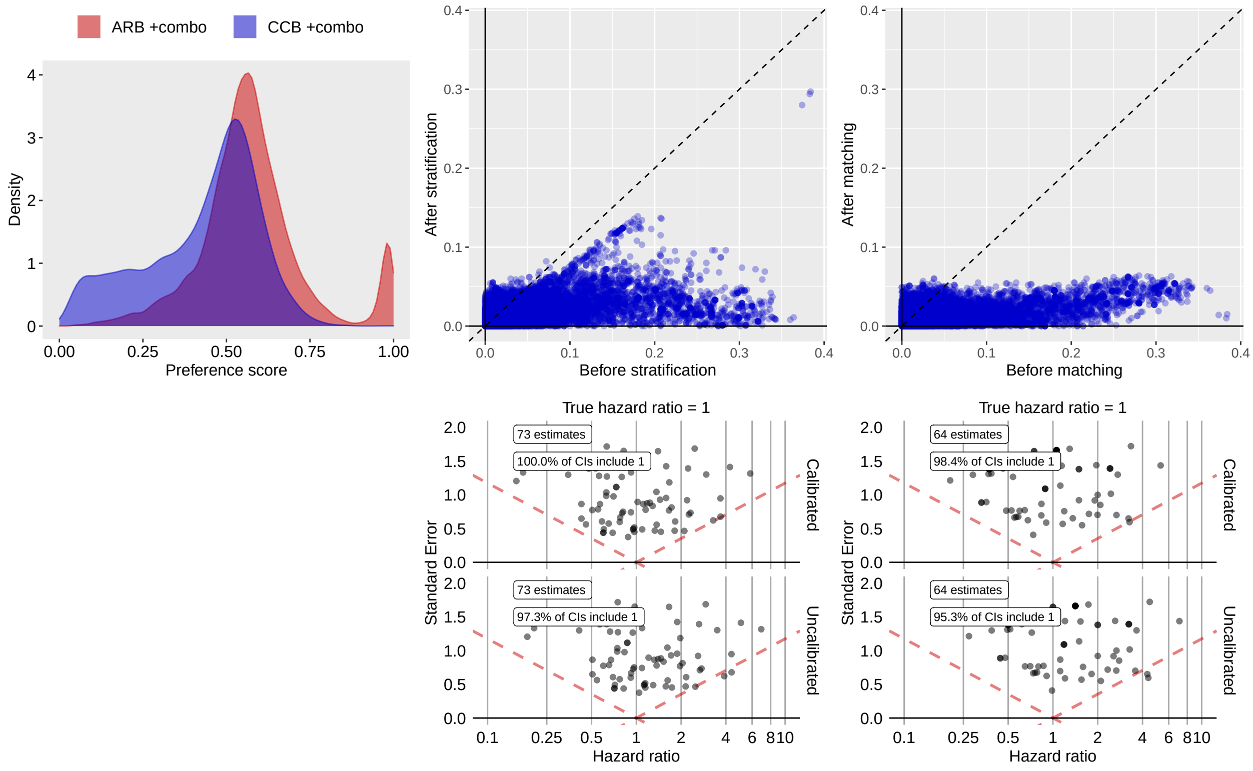

Supplementary Table 179: Most extreme patient characteristics balance diagnostics after stratification for ARB +combo vs CCB +combo in the CUIMC data source.

Characteristic (total count = 32375)	Before stratification			After stratification		
	T (%)	C (%)	SDf	T (%)	C (%)	SDf
drug_era group during day 0 through 0 days relative to index: sacubitril	6.9	-0.1	0.38	4.3	0.0	0.30
drug_era group during day -30 through 0 days relative to index: sacubitril	6.9	-0.1	0.38	4.3	0.1	0.29
drug_era group during day -180 through 0 days relative to index: sacubitril	7.1	0.2	0.37	4.5	0.3	0.28
condition_era group during day -180 through 0 days relative to index: Second trimester pregnancy	-0.1	1.6	-0.18	-0.1	1.1	-0.14
condition_era group during day -180 through 0 days relative to index: Systolic heart failure	8.5	3.6	0.21	6.7	3.6	0.14
condition_era group during day -180 through 0 days relative to index: Systolic dysfunction	8.6	3.7	0.21	6.7	3.7	0.14

(Continued)	T (%)	C (%)	SDf	T (%)	C (%)	SDf
procedure_occurrence during day -180 through 0 days relative to index: Ultrasound, pregnant uterus, real time with image documentation, follow-up (eg, re-evaluation of fetal size by measuring standard growth parameters and amniotic fluid volume, re-evaluation of organ system(s) suspected or confirmed to be abnormal on a prev	-0.1	1.6	-0.18	-0.1	1.0	-0.14
condition_era group during day -180 through 0 days relative to index: Hypertension AND/OR vomiting complicating pregnancy childbirth AND/OR puerperium	0.1	1.8	-0.18	0.1	1.2	-0.13
condition_era group during day -30 through 0 days relative to index: Systolic heart failure	5.0	1.8	0.18	3.9	1.7	0.13
condition_era group during day -30 through 0 days relative to index: Hypertension AND/OR vomiting complicating pregnancy childbirth AND/OR puerperium	-0.1	1.5	-0.17	-0.1	1.0	-0.13

Supplementary Table 180: Most extreme patient characteristics balance diagnostics after matching for ARB +combo vs CCB +combo in the CUIMC data source.

	Before matching			After matching		
Characteristic (total count = 31197)	T (%)	C (%)	SDf	T (%)	C (%)	SDf
measurement during day -30 through 0 days relative to index: Lymphocytes [#/volume] in Blood by Automated count	16.9	28.5	-0.28	18.0	15.6	0.06
measurement during day -30 through 0 days relative to index: Neutrophils [#/volume] in Blood by Automated count	16.5	27.5	-0.27	17.6	15.3	0.06
measurement during day -30 through 0 days relative to index: Globulin [Mass/volume] in Serum	16.5	28.5	-0.29	17.4	15.1	0.06
measurement during day -30 through 0 days relative to index: Platelet mean volume [Entitic volume] in Blood by Automated count	18.8	32.5	-0.32	20.2	17.7	0.06
measurement during day -30 through 0 days relative to index: Neutrophils/100 leukocytes in Blood by Automated count	16.5	27.4	-0.27	17.6	15.3	0.06
measurement during day -30 through 0 days relative to index: Eosinophils/100 leukocytes in Blood by Automated count	16.5	27.4	-0.27	17.6	15.3	0.06
measurement during day -30 through 0 days relative to index: Basophils [#/volume] in Blood by Automated count	16.5	27.4	-0.27	17.6	15.2	0.06
measurement during day -30 through 0 days relative to index: Basophils/100 leukocytes in Blood by Automated count	16.7	28.1	-0.28	17.8	15.5	0.06
measurement during day -180 through 0 days relative to index: Leukocytes [#/volume] in Urine	11.6	20.8	-0.25	12.5	10.5	0.06
measurement during day -30 through 0 days relative to index: Eosinophils [#/volume] in Blood by Automated count	16.5	27.4	-0.27	17.6	15.3	0.06

Supplementary Figure 59: Preference score distributions, propensity score stratified (middle column) and matched (right column) covariate balance and negative control error distributions (bottom row) for ARB mono vs THZ mono in the CUIMC data source.

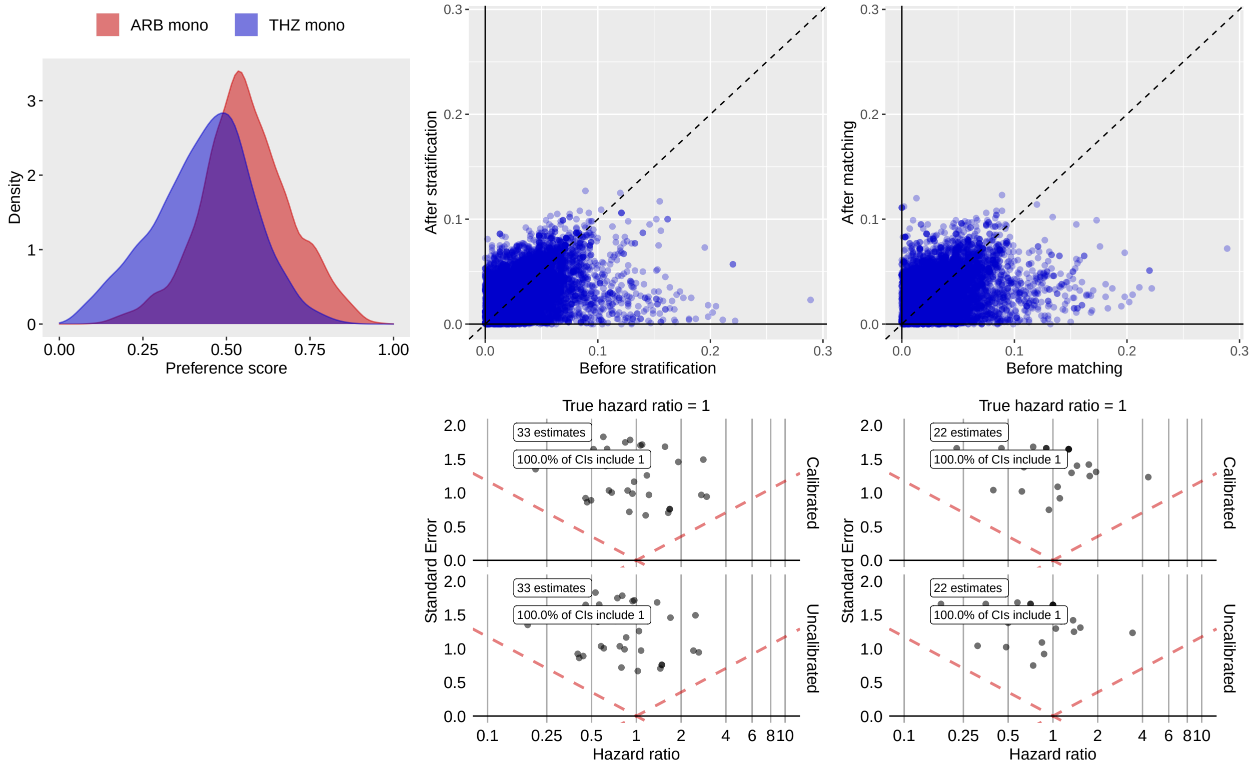

Supplementary Table 181: Most extreme patient characteristics balance diagnostics after stratification for ARB mono vs THZ mono in the CUIMC data source.

	Before stratification			After stratification		
Characteristic (total count = 20772)	T (%)	C (%)	SDf	T (%)	C (%)	SDf
condition_era group during day -180 through 0 days relative to index: Precapillary pulmonary hypertension	0.2	0.9	-0.09	0.2	1.2	-0.13
condition_era group during day -30 through 0 days relative to index: Abnormal findings on diagnostic imaging of lung	1.6	0.4	0.12	1.5	0.3	0.12
condition_era group during day -30 through 0 days relative to index: Cardiomyopathy	1.5	-0.3	0.15	1.2	-0.3	0.12
race = Asian	2.1	0.9	0.10	2.0	0.7	0.11
condition_era group during day -180 through 0 days relative to index: Diarrhea	1.4	0.6	0.08	1.4	0.4	0.11
condition_era group during day -30 through 0 days relative to index: Proteinuria	1.5	0.3	0.12	1.3	0.3	0.11
drug_era group during day -30 through 0 days relative to index: Methylprednisolone	2.2	0.7	0.12	1.9	0.7	0.11
condition_era group during day -30 through 0 days relative to index: Abnormal presence of protein	1.5	0.3	0.12	1.3	0.3	0.11

(Continued)	T (%)	C (%)	SDf	T (%)	C (%)	SDf
measurement below normal range during day -180 through 0 days relative to index: Anion gap in Serum or Plasma	1.4	0.5	0.09	1.4	0.4	0.11
measurement during day -180 through 0 days relative to index: Alpha-1-fetoprotein.tumor marker [Mass/volume] in Serum or Plasma	0.3	0.8	-0.06	0.3	1.2	-0.10

Supplementary Table 182: Most extreme patient characteristics balance diagnostics after matching for ARB mono vs THZ mono in the CUIMC data source.

	Before matching			After matching		
Characteristic (total count = 20087)	T (%)	C (%)	SDf	T (%)	C (%)	SDf
condition_era group during day -180 through 0 days relative to index: Precapillary pulmonary hypertension	0.2	0.9	-0.09	0.1	1.1	-0.12
drug_era group during day -180 through 0 days relative to index: Calcium Chloride	3.0	2.8	0.01	3.6	1.7	0.12
condition_era group during day -30 through 0 days relative to index: Abnormal findings on diagnostic imaging of lung	1.6	0.4	0.12	1.4	-0.4	0.11
drug_era group during day -180 through 0 days relative to index: ANESTHETICS, GENERAL	3.4	3.3	0.00	4.1	2.2	0.11
drug_era group during day -180 through 0 days relative to index: Fentanyl	3.4	3.3	0.00	4.1	2.2	0.11
drug_era group during day -180 through 0 days relative to index: Opioid anesthetics	3.4	3.3	0.00	4.1	2.2	0.11
drug_era group during day -180 through 0 days relative to index: Phenylpiperidine derivatives	3.4	3.3	0.00	4.1	2.2	0.11
condition_era group during day -180 through 0 days relative to index: Diarrhea	1.4	0.6	0.08	1.3	-0.4	0.11
drug_era group during day -30 through 0 days relative to index: potassium citrate	0.3	1.0	-0.09	0.3	1.2	-0.11
condition_era group during day -180 through 0 days relative to index: Pulmonary arterial hypertension	0.2	0.7	-0.07	0.1	0.9	-0.11

Supplementary Figure 60: Preference score distributions, propensity score stratified (middle column) and matched (right column) covariate balance and negative control error distributions (bottom row) for ARB +combo vs THZ +combo in the CUIMC data source.

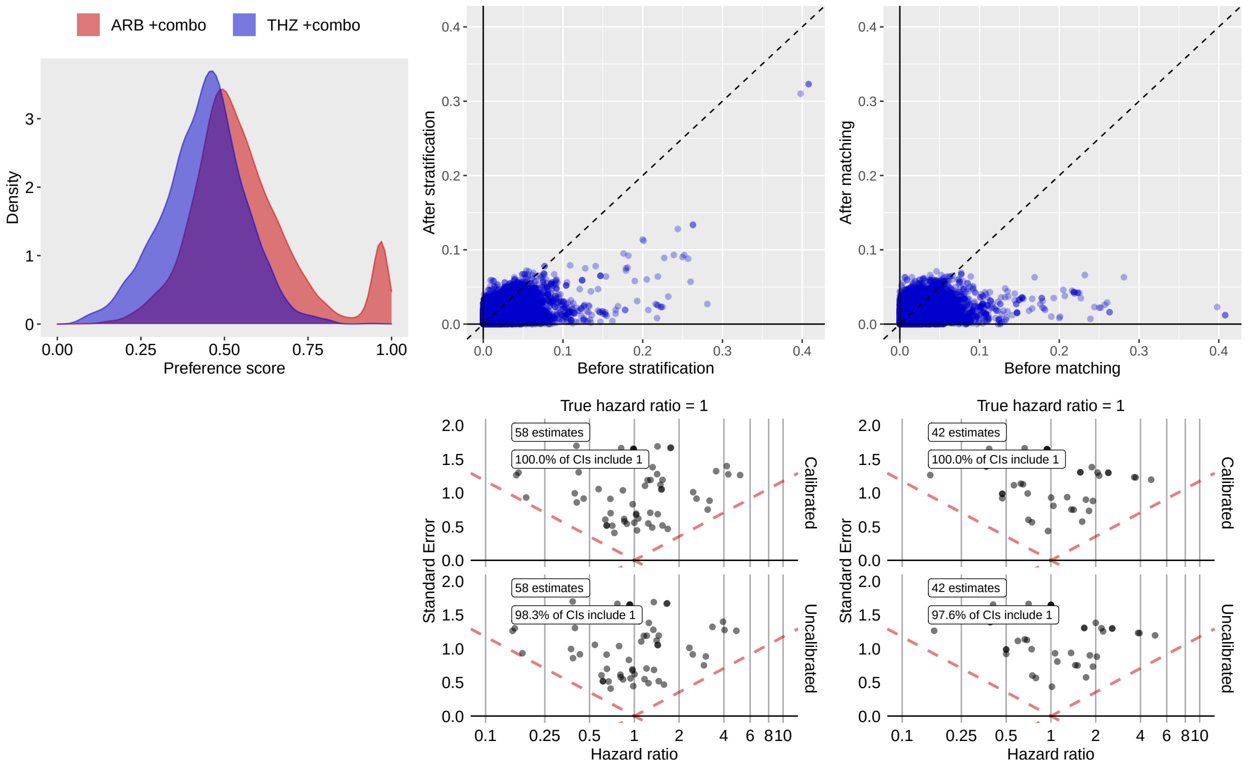

Supplementary Table 183: Most extreme patient characteristics balance diagnostics after stratification for ARB +combo vs THZ +combo in the CUIMC data source.

	Before stratification			After stratification		
Characteristic (total count = 31001)	T (%)	C (%)	SDf	T (%)	C (%)	SDf
drug_era group during day -30 through 0 days relative to index: sacubitril	7.8	-0.1	0.41	5.2	0.1	0.32
drug_era group during day 0 through 0 days relative to index: sacubitril	7.8	-0.1	0.41	5.2	0.1	0.32
drug_era group during day -180 through 0 days relative to index: sacubitril	7.9	0.2	0.40	5.3	0.3	0.31
condition_era group during day -180 through 0 days relative to index: Systolic heart failure	10.1	3.6	0.26	7.5	4.3	0.13
condition_era group during day -180 through 0 days relative to index: Systolic dysfunction	10.2	3.6	0.26	7.5	4.4	0.13
condition_era group during day -180 through 0 days relative to index: Chronic systolic heart failure	8.6	3.0	0.24	6.4	3.6	0.13
condition_era group during day -30 through 0 days relative to index: Systolic heart failure	5.9	2.0	0.20	4.3	2.3	0.11
condition_era group during day -30 through 0 days relative to index: Systolic dysfunction	5.9	2.0	0.20	4.4	2.4	0.11

(Continued)	T (%)	C (%)	SDf	T (%)	C (%)	SDf
condition_era group during day -30 through 0 days relative to index: Chronic systolic heart failure	4.7	1.6	0.18	3.4	1.9	0.10
condition_era group during day -180 through 0 days relative to index: Abnormal cardiovascular function	13.7	6.2	0.25	10.6	7.9	0.09

Supplementary Table 184: Most extreme patient characteristics balance diagnostics after matching for ARB +combo vs THZ +combo in the CUIMC data source.

	Before matching			After matching		
Characteristic (total count = 29737)	T (%)	C (%)	SDf	T (%)	C (%)	SDf
condition_era group during day -30 through 0 days relative to index: Asteatosis cutis	0.3	-0.1	0.05	0.3	-0.1	0.07
procedure_occurrence during day -180 through 0 days relative to index: Performance of Urinary Filtration, Intermittent, Less than 6 Hours Per Day	0.8	0.3	0.08	0.6	0.2	0.07
measurement during day -180 through 0 days relative to index: INR in Capillary blood by Coagulation assay	0.7	0.2	0.08	0.5	0.1	0.07
measurement above normal range during day -180 through 0 days relative to index: INR in Capillary blood by Coagulation assay	0.7	0.2	0.08	0.5	0.1	0.07
condition_era group during day -180 through 0 days relative to index: Infection AND/OR inflammatory reaction due to internal prosthetic device, implant AND/OR graft	0.2	0.1	0.04	0.3	-0.1	0.07
condition_era group during day -180 through 0 days relative to index: Structural disorder of heart	34.9	24.4	0.23	23.9	26.8	-0.07
condition_era group during day -180 through 0 days relative to index: Lesion of toe	0.5	0.2	0.05	0.5	0.2	0.07
drug_era group during day -30 through 0 days relative to index: Lovastatin	0.1	0.4	-0.07	0.1	0.4	-0.07
drug_era group during day -180 through 0 days relative to index: Lovastatin	0.2	0.5	-0.06	0.2	0.5	-0.06
observation during day -180 through 0 days relative to index: Dependence on renal dialysis	0.8	0.2	0.09	0.6	0.2	0.06

Effect estimates

PS Stratification

SIDIAP

Supplementary Table 185: Risk estimates for COVID-19 diagnosis across propensity score stratified, prevalent-user cohorts in the SIDIAP data source. We report uncalibrated and calibrated hazard ratios (HRs) and their 95% confidence intervals (CIs). (+) indicates in-combination cohorts.

		Uncalibrated			Calibrated		
Target	Comparator	HR	95% CI	p	HR	95% CI	p
ACE/ARB	CCB/THZ	1.00	(0.84 - 1.19)	0.98	1.02	(0.86 - 1.21)	0.76
ACE/ARB+	CCB/THZ+	1.05	(0.91 - 1.22)	0.51	1.06	(0.92 - 1.24)	0.47
ACE/ARB	CCB	1.08	(0.87 - 1.34)	0.49	1.07	(0.87 - 1.33)	0.57
ACE/ARB+	CCB+	1.06	(0.90 - 1.26)	0.50	1.04	(0.88 - 1.24)	0.67
ACE/ARB	THZ	0.90	(0.69 - 1.17)	0.41	1.01	(0.73 - 1.39)	0.77
ACE/ARB+	THZ+	1.09	(0.87 - 1.37)	0.46	1.23	(0.98 - 1.54)	0.11
ACE	ARB	0.94	(0.75 - 1.19)	0.59	1.05	(0.83 - 1.33)	0.70
ACE+	ARB+	0.91	(0.80 - 1.05)	0.20	0.93	(0.79 - 1.10)	0.46
ACE	CCB/THZ	1.02	(0.85 - 1.22)	0.85	1.05	(0.88 - 1.25)	0.68
ACE+	CCB/THZ+	0.97	(0.85 - 1.11)	0.66	0.98	(0.86 - 1.12)	0.75
ACE	CCB	1.09	(0.88 - 1.36)	0.46	1.09	(0.88 - 1.36)	0.50
ACE+	CCB+	1.00	(0.85 - 1.17)	0.96	0.98	(0.83 - 1.14)	0.72
ACE	THZ	0.90	(0.69 - 1.18)	0.42	1.02	(0.78 - 1.33)	0.78
ACE+	THZ+	0.99	(0.84 - 1.17)	0.88	1.04	(0.84 - 1.30)	0.70
ARB	CCB/THZ	1.10	(0.86 - 1.42)	0.44	1.01	(0.79 - 1.30)	0.76
ARB+	CCB/THZ+	1.12	(0.92 - 1.36)	0.23	1.08	(0.89 - 1.31)	0.47
ARB	CCB	1.17	(0.88 - 1.55)	0.28	1.06	(0.79 - 1.40)	0.68
ARB+	CCB+	1.12	(0.93 - 1.34)	0.25	1.09	(0.91 - 1.32)	0.43
ARB	THZ	1.04	(0.75 - 1.45)	0.82	1.03	(0.63 - 1.71)	0.77
ARB+	THZ+	1.19	(0.98 - 1.45)	0.08	1.16	(0.96 - 1.41)	0.18

Supplementary Table 186: Risk estimates for COVID-19 hospitalization across propensity score stratifed, prevalent-user cohorts in the SIDIAP data source. We report uncalibrated and calibrated hazard ratios (HRs) and their 95% confidence intervals (CIs). (+) indicates in-combination cohorts.

			Uncalibrated			Calibrated	
Target	Comparator	HR	95% CI	p	HR	95% CI	p
ACE/ARB	CCB/THZ	0.98	(0.75 - 1.28)	0.85	1.00	(0.77 - 1.30)	0.85
ACE/ARB+	CCB/THZ+	1.01	(0.80 - 1.27)	0.96	1.02	(0.81 - 1.28)	0.82
ACE/ARB	CCB	1.04	(0.76 - 1.45)	0.81	1.04	(0.75 - 1.43)	0.80
ACE/ARB+	CCB+	0.95	(0.74 - 1.22)	0.67	0.93	(0.73 - 1.20)	0.63
ACE/ARB	THZ	0.95	(0.63 - 1.48)	0.80	1.07	(0.67 - 1.70)	0.76
ACE/ARB+	THZ+	1.22	(0.85 - 1.81)	0.31	1.37	(0.94 - 2.00)	0.12
ACE	ARB	1.04	(0.73 - 1.53)	0.83	1.16	(0.80 - 1.70)	0.47
ACE+	ARB+	0.89	(0.72 - 1.12)	0.32	0.91	(0.72 - 1.15)	0.48
ACE	CCB/THZ	0.99	(0.75 - 1.32)	0.96	1.02	(0.77 - 1.35)	0.83
ACE+	CCB/THZ+	0.93	(0.75 - 1.15)	0.51	0.94	(0.76 - 1.16)	0.61
ACE	CCB	1.06	(0.76 - 1.49)	0.75	1.06	(0.76 - 1.48)	0.74
ACE+	CCB+	0.88	(0.70 - 1.11)	0.26	0.86	(0.68 - 1.08)	0.23
ACE	THZ	0.94	(0.62 - 1.49)	0.80	1.07	(0.69 - 1.65)	0.77
ACE+	THZ+	1.01	(0.77 - 1.32)	0.97	1.06	(0.78 - 1.44)	0.70
ARB	CCB/THZ	0.98	(0.65 - 1.45)	0.91	0.89	(0.60 - 1.34)	0.61
ARB+	CCB/THZ+	1.04	(0.76 - 1.40)	0.79	1.00	(0.74 - 1.36)	0.83
ARB	CCB	1.05	(0.67 - 1.64)	0.83	0.95	(0.61 - 1.49)	0.79
ARB+	CCB+	1.00	(0.75 - 1.33)	0.99	0.98	(0.73 - 1.30)	0.82
ARB	THZ	0.94	(0.55 - 1.63)	0.83	0.94	(0.48 - 1.82)	0.81
ARB+	THZ+	1.27	(0.93 - 1.72)	0.13	1.24	(0.91 - 1.69)	0.20

Supplementary Table 187: Risk estimates for pneumonia across propensity score stratifed, prevalent-user cohorts in the SIDIAP data source. We report uncalibrated and calibrated hazard ratios (HRs) and their 95% confidence intervals (CIs). (+) indicates in-combination cohorts.

			Uncalibrated			Calibrated	
Target	Comparator	HR	95% CI	p	HR	95% CI	p
ACE/ARB	CCB/THZ	1.32	(0.78 - 2.37)	0.32	1.36	(0.78 - 2.37)	0.30
ACE/ARB+	CCB/THZ+	1.14	(0.74 - 1.81)	0.55	1.16	(0.74 - 1.81)	0.53
ACE/ARB	CCB	1.98	(0.95 - 4.81)	0.10	1.97	(0.88 - 4.43)	0.10
ACE/ARB+	CCB+	1.24	(0.74 - 2.20)	0.44	1.22	(0.71 - 2.09)	0.49
ACE/ARB	THZ	0.87	(0.43 - 2.01)	0.73	0.98	(0.45 - 2.17)	0.91
ACE/ARB+	THZ+	1.01	(0.55 - 2.03)	0.97	1.14	(0.60 - 2.18)	0.69
ACE	ARB	0.83	(0.45 - 1.67)	0.59	0.93	(0.48 - 1.81)	0.84
ACE+	ARB+	1.01	(0.67 - 1.56)	0.97	1.03	(0.67 - 1.58)	0.89
ACE	CCB/THZ	1.28	(0.73 - 2.34)	0.41	1.32	(0.74 - 2.36)	0.37
ACE+	CCB/THZ+	1.17	(0.77 - 1.78)	0.47	1.18	(0.78 - 1.79)	0.45
ACE	CCB	1.94	(0.91 - 4.79)	0.12	1.95	(0.85 - 4.47)	0.12
ACE+	CCB+	1.30	(0.80 - 2.22)	0.31	1.28	(0.76 - 2.13)	0.36
ACE	THZ	0.77	(0.37 - 1.81)	0.52	0.87	(0.40 - 1.93)	0.74
ACE+	THZ+	1.01	(0.63 - 1.66)	0.98	1.06	(0.64 - 1.77)	0.81
ARB	CCB/THZ	1.55	(0.71 - 3.26)	0.26	1.42	(0.66 - 3.03)	0.38
ARB+	CCB/THZ+	1.17	(0.65 - 1.98)	0.59	1.12	(0.64 - 1.95)	0.69
ARB	CCB	2.19	(0.86 - 6.00)	0.12	1.97	(0.75 - 5.22)	0.17
ARB+	CCB+	0.93	(0.54 - 1.60)	0.79	0.91	(0.53 - 1.57)	0.75
ARB	THZ	1.21	(0.48 - 3.24)	0.69	1.21	(0.43 - 3.38)	0.71
ARB+	THZ+	1.12	(0.59 - 2.04)	0.73	1.09	(0.59 - 2.02)	0.79

Supplementary Table 188: Risk estimates for PAAS across propensity score stratifed, prevalent-user cohorts in the SIDIAP data source. We report uncalibrated and calibrated hazard ratios (HRs) and their 95% confidence intervals (CIs). (+) indicates in-combination cohorts.

Target	Comparator	Uncalibrated HR	Uncalibrated 95% CI	Uncalibrated p	Calibrated HR	Calibrated 95% CI	Calibrated p
ACE/ARB	CCB/THZ	1.33	(0.79 - 2.34)	0.30	1.36	(0.79 - 2.34)	0.28
ACE/ARB+	CCB/THZ+	1.09	(0.72 - 1.69)	0.70	1.10	(0.72 - 1.69)	0.67
ACE/ARB	CCB	1.87	(0.94 - 4.28)	0.11	1.86	(0.87 - 3.99)	0.11
ACE/ARB+	CCB+	1.17	(0.72 - 2.02)	0.54	1.15	(0.69 - 1.93)	0.59
ACE/ARB	THZ	0.89	(0.44 - 2.05)	0.77	1.01	(0.46 - 2.21)	0.91
ACE/ARB+	THZ+	0.87	(0.49 - 1.65)	0.64	0.98	(0.53 - 1.78)	0.90
ACE	ARB	0.90	(0.48 - 1.79)	0.74	1.00	(0.52 - 1.93)	0.91
ACE+	ARB+	1.01	(0.68 - 1.56)	0.95	1.03	(0.67 - 1.58)	0.87
ACE	CCB/THZ	1.31	(0.76 - 2.34)	0.35	1.35	(0.76 - 2.37)	0.32
ACE+	CCB/THZ+	1.13	(0.75 - 1.69)	0.57	1.14	(0.76 - 1.71)	0.54
ACE	CCB	1.88	(0.92 - 4.36)	0.11	1.89	(0.87 - 4.12)	0.11
ACE+	CCB+	1.25	(0.78 - 2.09)	0.37	1.23	(0.75 - 2.01)	0.42
ACE	THZ	0.80	(0.39 - 1.87)	0.58	0.91	(0.41 - 1.99)	0.81
ACE+	THZ+	0.95	(0.60 - 1.54)	0.81	1.00	(0.61 - 1.64)	0.91
ARB	CCB/THZ	1.49	(0.69 - 3.09)	0.30	1.36	(0.64 - 2.88)	0.43
ARB+	CCB/THZ+	1.15	(0.65 - 1.92)	0.62	1.10	(0.64 - 1.88)	0.73
ARB	CCB	1.98	(0.80 - 5.17)	0.15	1.79	(0.70 - 4.55)	0.23
ARB+	CCB+	0.94	(0.55 - 1.60)	0.82	0.92	(0.54 - 1.57)	0.78
ARB	THZ	1.21	(0.48 - 3.24)	0.69	1.21	(0.43 - 3.38)	0.71
ARB+	THZ+	1.04	(0.56 - 1.85)	0.91	1.01	(0.56 - 1.84)	0.91

VA-OMOP

Supplementary Table 189: Risk estimates for COVID-19 diagnosis across propensity score stratified, prevalent-user cohorts in the VA-OMOP data source. We report uncalibrated and calibrated hazard ratios (HRs) and their 95% confidence intervals (CIs). (+) indicates in-combination cohorts.

			Uncalibrated			Calibrated	
Target	Comparator	HR	95% CI	p	HR	95% CI	p
ACE/ARB	CCB/THZ	0.90	(0.70 - 1.16)	0.43	0.91	(0.71 - 1.17)	0.48
ACE/ARB+	CCB/THZ+	0.97	(0.82 - 1.14)	0.69	0.98	(0.81 - 1.18)	0.80
ACE/ARB	CCB	0.87	(0.66 - 1.15)	0.33	0.89	(0.66 - 1.19)	0.41
ACE/ARB+	CCB+	0.83	(0.71 - 0.98)	0.03	0.85	(0.71 - 1.02)	0.08
ACE/ARB	THZ	0.80	(0.55 - 1.19)	0.27	0.85	(0.58 - 1.25)	0.42
ACE/ARB+	THZ+	1.08	(0.87 - 1.35)	0.51	1.12	(0.88 - 1.43)	0.35
ACE	ARB	0.73	(0.51 - 1.05)	0.09	0.73	(0.51 - 1.04)	0.09
ACE+	ARB+	0.83	(0.71 - 0.97)	0.02	0.84	(0.71 - 0.99)	0.04
ACE	CCB/THZ	0.77	(0.57 - 1.01)	0.07	0.77	(0.57 - 1.03)	0.07
ACE+	CCB/THZ+	0.90	(0.76 - 1.07)	0.24	0.91	(0.75 - 1.10)	0.33
ACE	CCB	0.73	(0.53 - 1.01)	0.06	0.74	(0.53 - 1.03)	0.07
ACE+	CCB+	0.78	(0.66 - 0.91)	< 0.01	0.79	(0.66 - 0.94)	< 0.01
ACE	THZ	0.66	(0.44 - 1.01)	0.05	0.70	(0.46 - 1.06)	0.10
ACE+	THZ+	1.05	(0.86 - 1.28)	0.66	1.09	(0.87 - 1.37)	0.45
ARB	CCB/THZ	1.12	(0.77 - 1.58)	0.55	1.11	(0.78 - 1.58)	0.57
ARB+	CCB/THZ+	1.21	(0.98 - 1.48)	0.07	1.21	(0.99 - 1.49)	0.07
ARB	CCB	1.14	(0.77 - 1.66)	0.50	1.15	(0.76 - 1.73)	0.51
ARB+	CCB+	1.07	(0.88 - 1.29)	0.50	1.06	(0.88 - 1.28)	0.54
ARB	THZ	1.07	(0.65 - 1.75)	0.80	1.12	(0.68 - 1.84)	0.66
ARB+	THZ+	1.35	(1.09 - 1.67)	< 0.01	1.37	(1.10 - 1.70)	< 0.01

Supplementary Table 190: Risk estimates for COVID-19 hospitalization across propensity score stratifed, prevalent-user cohorts in the VA-OMOP data source. We report uncalibrated and calibrated hazard ratios (HRs) and their 95% confidence intervals (CIs). (+) indicates in-combination cohorts.

Target	Comparator	Uncalibrated HR	Uncalibrated 95% CI	Uncalibrated p	Calibrated HR	Calibrated 95% CI	Calibrated p
ACE/ARB	CCB/THZ	1.12	(0.81 - 1.53)	0.50	1.12	(0.82 - 1.54)	0.47
ACE/ARB+	CCB/THZ+	0.97	(0.81 - 1.16)	0.76	0.98	(0.81 - 1.20)	0.87
ACE/ARB	CCB	0.96	(0.68 - 1.35)	0.81	0.98	(0.69 - 1.39)	0.88
ACE/ARB+	CCB+	0.88	(0.74 - 1.05)	0.15	0.90	(0.74 - 1.09)	0.27
ACE/ARB	THZ	1.07	(0.65 - 1.84)	0.80	1.13	(0.67 - 1.91)	0.65
ACE/ARB+	THZ+	1.35	(1.05 - 1.77)	0.02	1.41	(1.06 - 1.87)	0.02
ACE	ARB	0.69	(0.46 - 1.05)	0.08	0.69	(0.46 - 1.04)	0.08
ACE+	ARB+	0.94	(0.80 - 1.11)	0.46	0.95	(0.80 - 1.13)	0.58
ACE	CCB/THZ	1.02	(0.71 - 1.46)	0.90	1.03	(0.71 - 1.47)	0.89
ACE+	CCB/THZ+	0.95	(0.79 - 1.13)	0.55	0.96	(0.79 - 1.16)	0.64
ACE	CCB	0.84	(0.57 - 1.23)	0.37	0.85	(0.57 - 1.25)	0.40
ACE+	CCB+	0.87	(0.74 - 1.04)	0.12	0.89	(0.74 - 1.07)	0.21
ACE	THZ	1.07	(0.62 - 1.92)	0.80	1.13	(0.65 - 1.99)	0.66
ACE+	THZ+	1.09	(0.87 - 1.36)	0.47	1.13	(0.88 - 1.45)	0.33
ARB	CCB/THZ	1.54	(0.99 - 2.35)	0.05	1.53	(0.99 - 2.35)	0.06
ARB+	CCB/THZ+	1.00	(0.80 - 1.24)	0.98	1.00	(0.81 - 1.25)	0.95
ARB	CCB	1.38	(0.88 - 2.16)	0.16	1.39	(0.87 - 2.23)	0.17
ARB+	CCB+	1.02	(0.83 - 1.25)	0.82	1.02	(0.83 - 1.25)	0.86
ARB	THZ	1.71	(0.90 - 3.32)	0.11	1.80	(0.94 - 3.45)	0.08
ARB+	THZ+	1.17	(0.93 - 1.47)	0.17	1.19	(0.94 - 1.51)	0.14

Supplementary Table 191: Risk estimates for pneumonia across propensity score stratifed, prevalent-user cohorts in the VA-OMOP data source. We report uncalibrated and calibrated hazard ratios (HRs) and their 95% confidence intervals (CIs). (+) indicates in-combination cohorts.

Target	Comparator	Uncalibrated HR	Uncalibrated 95% CI	Uncalibrated p	Calibrated HR	Calibrated 95% CI	Calibrated p
ACE/ARB	CCB/THZ	0.94	(0.78 - 1.13)	0.53	0.95	(0.79 - 1.14)	0.61
ACE/ARB+	CCB/THZ+	0.95	(0.86 - 1.06)	0.39	0.96	(0.84 - 1.11)	0.60
ACE/ARB	CCB	0.90	(0.74 - 1.11)	0.33	0.92	(0.73 - 1.15)	0.44
ACE/ARB+	CCB+	0.89	(0.80 - 0.99)	0.03	0.91	(0.79 - 1.04)	0.15
ACE/ARB	THZ	0.97	(0.71 - 1.33)	0.83	1.02	(0.74 - 1.40)	0.90
ACE/ARB+	THZ+	1.07	(0.93 - 1.25)	0.35	1.12	(0.93 - 1.35)	0.23
ACE	ARB	0.90	(0.69 - 1.17)	0.42	0.90	(0.69 - 1.16)	0.42
ACE+	ARB+	0.93	(0.84 - 1.03)	0.15	0.94	(0.84 - 1.05)	0.30
ACE	CCB/THZ	0.91	(0.74 - 1.12)	0.37	0.91	(0.74 - 1.13)	0.41
ACE+	CCB/THZ+	0.96	(0.86 - 1.07)	0.47	0.97	(0.85 - 1.11)	0.65
ACE	CCB	0.87	(0.69 - 1.08)	0.21	0.87	(0.69 - 1.10)	0.26
ACE+	CCB+	0.90	(0.81 - 1.00)	0.05	0.91	(0.81 - 1.03)	0.15
ACE	THZ	0.92	(0.67 - 1.30)	0.64	0.97	(0.70 - 1.36)	0.88
ACE+	THZ+	1.06	(0.93 - 1.21)	0.38	1.11	(0.93 - 1.31)	0.24
ARB	CCB/THZ	1.03	(0.78 - 1.35)	0.83	1.02	(0.78 - 1.35)	0.86
ARB+	CCB/THZ+	1.06	(0.93 - 1.20)	0.39	1.06	(0.93 - 1.20)	0.39
ARB	CCB	1.03	(0.76 - 1.37)	0.86	1.03	(0.75 - 1.43)	0.85
ARB+	CCB+	1.00	(0.88 - 1.13)	0.97	0.99	(0.88 - 1.12)	0.91
ARB	THZ	1.11	(0.74 - 1.68)	0.62	1.16	(0.77 - 1.75)	0.47
ARB+	THZ+	1.10	(0.96 - 1.26)	0.15	1.12	(0.97 - 1.30)	0.12

Supplementary Table 192: Risk estimates for PAAS across propensity score stratifed, prevalent-user cohorts in the VA-OMOP data source. We report uncalibrated and calibrated hazard ratios (HRs) and their 95% confidence intervals (CIs). (+) indicates in-combination cohorts.

		Uncalibrated			Calibrated		
Target	Comparator	HR	95% CI	p	HR	95% CI	p
ACE/ARB	CCB/THZ	1.14	(1.00 - 1.29)	0.05	1.14	(1.00 - 1.30)	0.05
ACE/ARB+	CCB/THZ+	1.17	(1.08 - 1.27)	< 0.01	1.18	(1.05 - 1.33)	< 0.01
ACE/ARB	CCB	1.09	(0.94 - 1.25)	0.25	1.11	(0.93 - 1.31)	0.25
ACE/ARB+	CCB+	1.15	(1.07 - 1.25)	< 0.01	1.18	(1.05 - 1.32)	< 0.01
ACE/ARB	THZ	1.05	(0.85 - 1.29)	0.67	1.11	(0.90 - 1.36)	0.35
ACE/ARB+	THZ+	1.06	(0.96 - 1.18)	0.25	1.11	(0.95 - 1.28)	0.17
ACE	ARB	1.02	(0.86 - 1.22)	0.83	1.02	(0.85 - 1.21)	0.85
ACE+	ARB+	1.05	(0.98 - 1.12)	0.18	1.06	(0.97 - 1.16)	0.18
ACE	CCB/THZ	1.17	(1.02 - 1.34)	0.03	1.17	(1.01 - 1.36)	0.03
ACE+	CCB/THZ+	1.18	(1.09 - 1.27)	< 0.01	1.19	(1.07 - 1.32)	< 0.01
ACE	CCB	1.14	(0.98 - 1.32)	0.10	1.15	(0.97 - 1.36)	0.12
ACE+	CCB+	1.16	(1.07 - 1.25)	< 0.01	1.18	(1.06 - 1.30)	< 0.01
ACE	THZ	1.07	(0.86 - 1.34)	0.54	1.13	(0.91 - 1.41)	0.29
ACE+	THZ+	1.04	(0.95 - 1.13)	0.38	1.08	(0.94 - 1.24)	0.24
ARB	CCB/THZ	1.10	(0.91 - 1.33)	0.33	1.09	(0.90 - 1.32)	0.38
ARB+	CCB/THZ+	1.08	(0.98 - 1.18)	0.12	1.08	(0.98 - 1.18)	0.14
ARB	CCB	1.08	(0.87 - 1.32)	0.49	1.08	(0.84 - 1.38)	0.53
ARB+	CCB+	1.11	(1.01 - 1.21)	0.02	1.10	(1.01 - 1.20)	0.05
ARB	THZ	0.98	(0.75 - 1.29)	0.91	1.03	(0.79 - 1.35)	0.82
ARB+	THZ+	0.88	(0.80 - 0.96)	< 0.01	0.89	(0.81 - 0.99)	0.04

CUIMC

Supplementary Table 193: Risk estimates for COVID-19 diagnosis across propensity score stratified, prevalent-user cohorts in the CUIMC data source. We report uncalibrated and calibrated hazard ratios (HRs) and their 95% confidence intervals (CIs). (+) indicates in-combination cohorts.

			Uncalibrated			Calibrated	
Target	Comparator	HR	95% CI	p	HR	95% CI	p
ACE/ARB	CCB/THZ	1.62	(0.91 - 2.93)	0.10	1.46	(0.81 - 2.62)	0.22
ACE/ARB+	CCB/THZ+	1.41	(0.95 - 2.10)	0.09	1.28	(0.86 - 1.90)	0.27
ACE/ARB	CCB	1.21	(0.63 - 2.37)	0.57	1.06	(0.55 - 2.05)	0.78
ACE/ARB+	CCB+	1.17	(0.79 - 1.77)	0.43	1.09	(0.73 - 1.64)	0.70
ACE/ARB	THZ	2.10	(0.82 - 6.52)	0.16	2.90	(1.03 - 8.17)	0.05
ACE/ARB+	THZ+	1.44	(0.84 - 2.63)	0.21	1.86	(1.05 - 3.29)	0.04
ACE	ARB	0.58	(0.25 - 1.30)	0.20	0.61	(0.27 - 1.38)	0.25
ACE+	ARB+	0.65	(0.43 - 0.98)	0.04	0.58	(0.38 - 0.87)	0.01
ACE	CCB/THZ	1.17	(0.52 - 2.48)	0.69	0.90	(0.40 - 2.00)	0.79
ACE+	CCB/THZ+	0.86	(0.49 - 1.44)	0.58	0.65	(0.38 - 1.12)	0.14
ACE	CCB	0.89	(0.37 - 2.03)	0.79	0.64	(0.27 - 1.50)	0.33
ACE+	CCB+	0.97	(0.60 - 1.56)	0.92	0.78	(0.48 - 1.26)	0.34
ACE	THZ	1.43	(0.47 - 4.90)	0.55	1.71	(0.53 - 5.54)	0.38
ACE+	THZ+	0.90	(0.51 - 1.58)	0.71	0.81	(0.46 - 1.42)	0.48
ARB	CCB/THZ	2.20	(1.10 - 4.31)	0.02	1.72	(0.87 - 3.40)	0.13
ARB+	CCB/THZ+	2.19	(1.44 - 3.27)	< 0.01	1.69	(1.12 - 2.55)	0.02
ARB	CCB	1.96	(0.90 - 4.26)	0.09	1.44	(0.66 - 3.12)	0.39
ARB+	CCB+	1.54	(1.00 - 2.36)	0.05	1.30	(0.85 - 1.99)	0.27
ARB	THZ	2.63	(0.93 - 8.67)	0.09	2.97	(0.98 - 9.06)	0.06
ARB+	THZ+	1.69	(1.03 - 2.79)	0.04	1.79	(1.09 - 2.94)	0.03

Supplementary Table 194: Risk estimates for COVID-19 hospitalization across propensity score stratifed, prevalent-user cohorts in the CUIMC data source. We report uncalibrated and calibrated hazard ratios (HRs) and their 95% confidence intervals (CIs). (+) indicates in-combination cohorts.

		Uncalibrated			Calibrated		
Target	Comparator	HR	95% CI	p	HR	95% CI	p
ACE/ARB	CCB/THZ	1.17	(0.68 - 2.01)	0.57	1.05	(0.61 - 1.81)	0.78
ACE/ARB+	CCB/THZ+	1.01	(0.71 - 1.41)	0.97	0.91	(0.65 - 1.28)	0.62
ACE/ARB	CCB	0.98	(0.54 - 1.75)	0.93	0.86	(0.48 - 1.54)	0.65
ACE/ARB+	CCB+	0.88	(0.64 - 1.22)	0.45	0.82	(0.59 - 1.13)	0.31
ACE/ARB	THZ	1.30	(0.51 - 4.04)	0.61	1.80	(0.64 - 5.05)	0.28
ACE/ARB+	THZ+	1.17	(0.72 - 1.98)	0.53	1.51	(0.92 - 2.50)	0.13
ACE	ARB	1.02	(0.45 - 2.36)	0.97	1.06	(0.46 - 2.41)	0.79
ACE+	ARB+	0.89	(0.62 - 1.29)	0.54	0.79	(0.55 - 1.14)	0.23
ACE	CCB/THZ	1.37	(0.70 - 2.56)	0.34	1.05	(0.54 - 2.04)	0.80
ACE+	CCB/THZ+	1.01	(0.66 - 1.51)	0.95	0.77	(0.51 - 1.16)	0.24
ACE	CCB	1.16	(0.57 - 2.25)	0.68	0.83	(0.42 - 1.65)	0.63
ACE+	CCB+	0.86	(0.58 - 1.26)	0.46	0.69	(0.47 - 1.02)	0.08
ACE	THZ	1.49	(0.53 - 4.89)	0.48	1.78	(0.59 - 5.41)	0.32
ACE+	THZ+	1.21	(0.77 - 1.92)	0.41	1.09	(0.69 - 1.71)	0.71
ARB	CCB/THZ	1.03	(0.47 - 2.07)	0.95	0.80	(0.38 - 1.68)	0.58
ARB+	CCB/THZ+	1.21	(0.79 - 1.81)	0.37	0.94	(0.62 - 1.42)	0.73
ARB	CCB	0.87	(0.38 - 1.88)	0.73	0.63	(0.28 - 1.42)	0.29
ARB+	CCB+	0.97	(0.66 - 1.41)	0.88	0.82	(0.56 - 1.20)	0.35
ARB	THZ	1.42	(0.45 - 5.14)	0.57	1.61	(0.47 - 5.43)	0.45
ARB+	THZ+	1.34	(0.81 - 2.24)	0.26	1.42	(0.85 - 2.36)	0.20

Supplementary Table 195: Risk estimates for pneumonia across propensity score stratifed, prevalent-user cohorts in the CUIMC data source. We report uncalibrated and calibrated hazard ratios (HRs) and their 95% confidence intervals (CIs). (+) indicates in-combination cohorts.

Target	Comparator	Uncalibrated HR	Uncalibrated 95% CI	Uncalibrated p	Calibrated HR	Calibrated 95% CI	Calibrated p
ACE/ARB	CCB/THZ	1.15	(0.46 - 2.79)	0.77	1.03	(0.42 - 2.54)	0.87
ACE/ARB+	CCB/THZ+	1.17	(0.61 - 2.22)	0.63	1.06	(0.55 - 2.03)	0.85
ACE/ARB	CCB	1.47	(0.53 - 4.23)	0.47	1.29	(0.46 - 3.64)	0.63
ACE/ARB+	CCB+	1.26	(0.68 - 2.38)	0.47	1.17	(0.62 - 2.20)	0.64
ACE/ARB	THZ	0.58	(0.17 - 2.63)	0.43	0.80	(0.20 - 3.13)	0.75
ACE/ARB+	THZ+	0.98	(0.43 - 2.52)	0.97	1.27	(0.53 - 3.05)	0.61
ACE	ARB	0.33	(0.07 - 1.28)	0.14	0.35	(0.08 - 1.49)	0.16
ACE+	ARB+	0.44	(0.22 - 0.84)	0.02	0.39	(0.20 - 0.77)	< 0.01
ACE	CCB/THZ	0.66	(0.15 - 2.19)	0.55	0.51	(0.13 - 1.97)	0.34
ACE+	CCB/THZ+	0.62	(0.23 - 1.41)	0.30	0.47	(0.19 - 1.16)	0.11
ACE	CCB	0.99	(0.20 - 3.78)	0.99	0.71	(0.17 - 3.06)	0.66
ACE+	CCB+	0.62	(0.24 - 1.39)	0.28	0.49	(0.20 - 1.19)	0.12
ACE	THZ	0.42	(0.07 - 2.48)	0.34	0.50	(0.09 - 2.97)	0.46
ACE+	THZ+	0.70	(0.29 - 1.65)	0.43	0.63	(0.26 - 1.51)	0.31
ARB	CCB/THZ	1.95	(0.67 - 5.32)	0.21	1.52	(0.54 - 4.30)	0.43
ARB+	CCB/THZ+	1.90	(0.93 - 3.69)	0.07	1.47	(0.74 - 2.92)	0.30
ARB	CCB	2.33	(0.74 - 7.33)	0.15	1.71	(0.54 - 5.40)	0.37
ARB+	CCB+	1.85	(0.97 - 3.50)	0.06	1.56	(0.82 - 2.97)	0.19
ARB	THZ	1.15	(0.28 - 6.06)	0.86	1.30	(0.28 - 6.01)	0.74
ARB+	THZ+	1.61	(0.73 - 3.77)	0.26	1.70	(0.75 - 3.88)	0.21

Supplementary Table 196: Risk estimates for PAAS across propensity score stratifed, prevalent-user cohorts in the CUIMC data source. We report uncalibrated and calibrated hazard ratios (HRs) and their 95% confidence intervals (CIs). (+) indicates in-combination cohorts.

Target	Comparator	Uncalibrated HR	Uncalibrated 95% CI	Uncalibrated p	Calibrated HR	Calibrated 95% CI	Calibrated p
ACE/ARB	CCB/THZ	1.37	(0.70 - 2.65)	0.36	1.23	(0.63 - 2.40)	0.55
ACE/ARB+	CCB/THZ+	1.28	(0.79 - 2.07)	0.31	1.16	(0.72 - 1.87)	0.58
ACE/ARB	CCB	1.12	(0.55 - 2.29)	0.76	0.99	(0.48 - 2.01)	0.83
ACE/ARB+	CCB+	1.05	(0.68 - 1.64)	0.81	0.98	(0.63 - 1.52)	0.83
ACE/ARB	THZ	3.60	(0.71 - 65.79)	0.27	4.97	(0.52 - 47.72)	0.17
ACE/ARB+	THZ+	1.67	(0.79 - 4.10)	0.22	2.15	(0.95 - 4.90)	0.08
ACE	ARB	0.46	(0.15 - 1.23)	0.14	0.48	(0.17 - 1.35)	0.18
ACE+	ARB+	0.54	(0.33 - 0.85)	< 0.01	0.48	(0.30 - 0.76)	< 0.01
ACE	CCB/THZ	1.04	(0.37 - 2.49)	0.94	0.79	(0.30 - 2.08)	0.66
ACE+	CCB/THZ+	0.81	(0.43 - 1.44)	0.50	0.61	(0.33 - 1.13)	0.13
ACE	CCB	0.92	(0.32 - 2.33)	0.87	0.66	(0.24 - 1.79)	0.44
ACE+	CCB+	0.80	(0.45 - 1.36)	0.43	0.64	(0.37 - 1.11)	0.13
ACE	THZ	3.74	(0.56 - 74.29)	0.29	4.48	(0.39 - 51.44)	0.23
ACE+	THZ+	0.87	(0.44 - 1.71)	0.68	0.78	(0.39 - 1.53)	0.48
ARB	CCB/THZ	2.11	(0.95 - 4.50)	0.06	1.65	(0.76 - 3.60)	0.22
ARB+	CCB/THZ+	2.07	(1.23 - 3.40)	< 0.01	1.60	(0.96 - 2.65)	0.09
ARB	CCB	1.63	(0.71 - 3.62)	0.24	1.19	(0.53 - 2.69)	0.68
ARB+	CCB+	1.53	(0.96 - 2.40)	0.07	1.29	(0.82 - 2.03)	0.32
ARB	THZ	6.61	(1.11 - 130.10)	0.12	7.46	(0.69 - 80.75)	0.10
ARB+	THZ+	2.35	(1.25 - 4.68)	0.01	2.50	(1.29 - 4.84)	< 0.01

Meta-analysis

These meta-analytic estimates are based on all data source estimates, not just those that pass diagnostics

Supplementary Table 197: Risk estimates for COVID-19 diagnosis across propensity score stratified, prevalent-user cohorts in the Meta-analysis across data sources. We report uncalibrated and calibrated hazard ratios (HRs) and their 95% confidence intervals (CIs). (+) indicates in-combination cohorts.

			Uncalibrated			Calibrated		
Target	Comparator	HR	95% CI	p	HR	95% CI	p	i^2
ACE/ARB	CCB/THZ	1.01	(0.83 - 1.24)	0.91	1.02	(0.83 - 1.25)	0.87	0.39
ACE/ARB+	CCB/THZ+	1.05	(0.91 - 1.21)	0.50	1.05	(0.91 - 1.23)	0.50	0.34
ACE/ARB	CCB	1.01	(0.86 - 1.19)	0.92	1.02	(0.85 - 1.22)	0.85	0.00
ACE/ARB+	CCB+	0.98	(0.80 - 1.19)	0.81	0.99	(0.80 - 1.23)	0.93	0.61
ACE/ARB	THZ	0.92	(0.68 - 1.23)	0.56	0.97	(0.73 - 1.31)	0.88	0.31
ACE/ARB+	THZ+	1.11	(0.95 - 1.29)	0.20	1.17	(0.98 - 1.39)	0.08	0.00
ACE	ARB	0.84	(0.68 - 1.04)	0.12	0.85	(0.69 - 1.05)	0.14	0.11
ACE+	ARB+	0.85	(0.76 - 0.96)	0.01	0.86	(0.76 - 0.98)	0.03	0.23
ACE	CCB/THZ	0.93	(0.75 - 1.15)	0.50	0.93	(0.74 - 1.17)	0.52	0.34
ACE+	CCB/THZ+	0.94	(0.85 - 1.04)	0.25	0.95	(0.83 - 1.08)	0.39	0.00
ACE	CCB	0.91	(0.68 - 1.23)	0.55	0.92	(0.67 - 1.25)	0.58	0.50
ACE+	CCB+	0.89	(0.73 - 1.08)	0.25	0.91	(0.74 - 1.12)	0.35	0.58
ACE	THZ	0.83	(0.64 - 1.07)	0.16	0.88	(0.68 - 1.15)	0.36	0.13
ACE+	THZ+	1.00	(0.89 - 1.14)	0.94	1.05	(0.92 - 1.21)	0.48	0.00
ARB	CCB/THZ	1.23	(0.92 - 1.65)	0.17	1.21	(0.90 - 1.62)	0.22	0.43
ARB+	CCB/THZ+	1.36	(1.01 - 1.83)	0.04	1.36	(1.01 - 1.83)	0.04	0.76
ARB	CCB	1.21	(0.97 - 1.51)	0.09	1.21	(0.93 - 1.57)	0.14	0.00
ARB+	CCB+	1.13	(0.98 - 1.31)	0.09	1.13	(0.96 - 1.31)	0.13	0.16
ARB	THZ	1.13	(0.82 - 1.56)	0.46	1.17	(0.85 - 1.63)	0.34	0.20
ARB+	THZ+	1.29	(1.12 - 1.48)	< 0.01	1.31	(1.14 - 1.52)	< 0.01	0.00

Supplementary Table 198: Risk estimates for COVID-19 hospitalization across propensity score stratifed, prevalent-user cohorts in the Meta-analysis across data sources. We report uncalibrated and calibrated hazard ratios (HRs) and their 95% confidence intervals (CIs). (+) indicates in-combination cohorts.

Target	Comparator	HR	Uncalibrated 95% CI	p	HR	Calibrated 95% CI	p	i^2
ACE/ARB	CCB/THZ	1.05	(0.87 - 1.27)	0.63	1.05	(0.87 - 1.28)	0.61	0.00
ACE/ARB+	CCB/THZ+	0.99	(0.87 - 1.13)	0.86	0.99	(0.86 - 1.14)	0.89	0.00
ACE/ARB	CCB	1.00	(0.80 - 1.24)	0.98	1.01	(0.80 - 1.27)	0.93	0.00
ACE/ARB+	CCB+	0.90	(0.79 - 1.02)	0.11	0.91	(0.79 - 1.06)	0.23	0.00
ACE/ARB	THZ	1.02	(0.74 - 1.40)	0.90	1.08	(0.79 - 1.48)	0.61	0.00
ACE/ARB+	THZ+	1.28	(1.05 - 1.57)	0.01	1.36	(1.09 - 1.68)	< 0.01	0.00
ACE	ARB	0.88	(0.66 - 1.17)	0.37	0.88	(0.66 - 1.17)	0.41	0.12
ACE+	ARB+	0.92	(0.81 - 1.04)	0.19	0.93	(0.82 - 1.06)	0.28	0.00
ACE	CCB/THZ	1.04	(0.84 - 1.28)	0.74	1.04	(0.84 - 1.29)	0.74	0.00
ACE+	CCB/THZ+	0.95	(0.83 - 1.08)	0.41	0.95	(0.82 - 1.11)	0.52	0.00
ACE	CCB	0.98	(0.77 - 1.24)	0.86	0.98	(0.77 - 1.26)	0.88	0.00
ACE+	CCB+	0.87	(0.77 - 0.99)	0.04	0.89	(0.76 - 1.03)	0.11	0.00
ACE	THZ	1.03	(0.74 - 1.43)	0.87	1.09	(0.78 - 1.52)	0.61	0.00
ACE+	THZ+	1.07	(0.91 - 1.26)	0.40	1.12	(0.94 - 1.33)	0.20	0.00
ARB	CCB/THZ	1.18	(0.87 - 1.61)	0.29	1.16	(0.85 - 1.58)	0.35	0.18
ARB+	CCB/THZ+	1.04	(0.89 - 1.23)	0.61	1.04	(0.89 - 1.23)	0.62	0.00
ARB	CCB	1.15	(0.86 - 1.55)	0.35	1.15	(0.83 - 1.60)	0.39	0.00
ARB+	CCB+	1.01	(0.86 - 1.17)	0.92	1.00	(0.85 - 1.18)	0.94	0.00
ARB	THZ	1.22	(0.82 - 1.81)	0.32	1.27	(0.86 - 1.89)	0.24	0.00
ARB+	THZ+	1.22	(1.03 - 1.45)	0.02	1.24	(1.04 - 1.48)	0.02	0.00

Supplementary Table 199: Risk estimates for pneumonia across propensity score stratifed, prevalent-user cohorts in the Meta-analysis across data sources. We report uncalibrated and calibrated hazard ratios (HRs) and their 95% confidence intervals (CIs). (+) indicates in-combination cohorts.

			Uncalibrated			Calibrated		
Target	Comparator	HR	95% CI	p	HR	95% CI	p	i^2
ACE/ARB	CCB/THZ	0.98	(0.83 - 1.17)	0.83	0.99	(0.83 - 1.18)	0.88	0.00
ACE/ARB+	CCB/THZ+	0.97	(0.87 - 1.07)	0.54	0.97	(0.87 - 1.09)	0.62	0.00
ACE/ARB	CCB	1.20	(0.71 - 2.03)	0.50	1.21	(0.71 - 2.07)	0.48	0.51
ACE/ARB+	CCB+	0.95	(0.78 - 1.16)	0.65	0.97	(0.79 - 1.20)	0.77	0.18
ACE/ARB	THZ	0.93	(0.70 - 1.24)	0.62	0.99	(0.74 - 1.32)	0.93	0.00
ACE/ARB+	THZ+	1.07	(0.92 - 1.24)	0.37	1.13	(0.95 - 1.34)	0.15	0.00
ACE	ARB	0.87	(0.68 - 1.10)	0.24	0.87	(0.68 - 1.11)	0.27	0.00
ACE+	ARB+	0.84	(0.61 - 1.17)	0.30	0.85	(0.61 - 1.18)	0.34	0.58
ACE	CCB/THZ	0.94	(0.78 - 1.14)	0.52	0.94	(0.77 - 1.15)	0.55	0.00
ACE+	CCB/THZ+	0.97	(0.87 - 1.07)	0.53	0.97	(0.86 - 1.10)	0.67	0.00
ACE	CCB	1.08	(0.64 - 1.84)	0.76	1.09	(0.64 - 1.86)	0.76	0.41
ACE+	CCB+	0.94	(0.73 - 1.20)	0.60	0.95	(0.73 - 1.23)	0.69	0.25
ACE	THZ	0.88	(0.65 - 1.19)	0.40	0.93	(0.69 - 1.27)	0.66	0.00
ACE+	THZ+	1.05	(0.92 - 1.19)	0.46	1.10	(0.95 - 1.26)	0.20	0.00
ARB	CCB/THZ	1.15	(0.85 - 1.54)	0.36	1.13	(0.84 - 1.51)	0.43	0.08
ARB+	CCB/THZ+	1.15	(0.89 - 1.49)	0.27	1.15	(0.89 - 1.49)	0.28	0.27
ARB	CCB	1.43	(0.79 - 2.59)	0.23	1.43	(0.78 - 2.63)	0.24	0.46
ARB+	CCB+	1.09	(0.80 - 1.49)	0.58	1.09	(0.79 - 1.49)	0.61	0.43
ARB	THZ	1.13	(0.78 - 1.63)	0.52	1.17	(0.81 - 1.69)	0.40	0.00
ARB+	THZ+	1.11	(0.98 - 1.27)	0.10	1.13	(0.99 - 1.30)	0.08	0.00

Supplementary Table 200: Risk estimates for PAAS across propensity score stratifed, prevalent-user cohorts in the Meta-analysis across data sources. We report uncalibrated and calibrated hazard ratios (HRs) and their 95% confidence intervals (CIs). (+) indicates in-combination cohorts.

Target	Comparator	HR	Uncalibrated 95% CI	p	HR	Calibrated 95% CI	p	i^2
ACE/ARB	CCB/THZ	1.15	(1.02 - 1.30)	0.02	1.16	(1.02 - 1.31)	0.03	0.00
ACE/ARB+	CCB/THZ+	1.17	(1.08 - 1.26)	< 0.01	1.17	(1.07 - 1.28)	< 0.01	0.00
ACE/ARB	CCB	1.11	(0.97 - 1.27)	0.14	1.12	(0.95 - 1.31)	0.18	0.00
ACE/ARB+	CCB+	1.15	(1.06 - 1.25)	< 0.01	1.17	(1.05 - 1.30)	< 0.01	0.00
ACE/ARB	THZ	1.05	(0.86 - 1.28)	0.66	1.11	(0.91 - 1.36)	0.31	0.00
ACE/ARB+	THZ+	1.06	(0.96 - 1.18)	0.23	1.12	(0.99 - 1.28)	0.08	0.00
ACE	ARB	0.96	(0.74 - 1.23)	0.73	0.96	(0.75 - 1.24)	0.78	0.13
ACE+	ARB+	0.87	(0.61 - 1.25)	0.46	0.88	(0.62 - 1.27)	0.50	0.74
ACE	CCB/THZ	1.17	(1.03 - 1.34)	0.02	1.18	(1.02 - 1.36)	0.03	0.00
ACE+	CCB/THZ+	1.17	(1.09 - 1.26)	< 0.01	1.18	(1.06 - 1.31)	< 0.01	0.00
ACE	CCB	1.15	(0.99 - 1.33)	0.06	1.15	(0.98 - 1.37)	0.09	0.00
ACE+	CCB+	1.15	(1.07 - 1.24)	< 0.01	1.17	(1.05 - 1.30)	< 0.01	0.00
ACE	THZ	1.06	(0.86 - 1.31)	0.59	1.13	(0.91 - 1.40)	0.29	0.00
ACE+	THZ+	1.03	(0.95 - 1.12)	0.45	1.08	(0.97 - 1.20)	0.14	0.00
ARB	CCB/THZ	1.29	(0.90 - 1.85)	0.16	1.27	(0.89 - 1.83)	0.19	0.34
ARB+	CCB/THZ+	1.30	(0.89 - 1.91)	0.18	1.30	(0.89 - 1.91)	0.18	0.68
ARB	CCB	1.20	(0.89 - 1.62)	0.24	1.20	(0.86 - 1.68)	0.28	0.16
ARB+	CCB+	1.13	(0.98 - 1.30)	0.09	1.12	(0.96 - 1.30)	0.14	0.11
ARB	THZ	1.11	(0.68 - 1.80)	0.68	1.15	(0.71 - 1.87)	0.57	0.22
ARB+	THZ+	1.20	(0.70 - 2.07)	0.51	1.22	(0.71 - 2.11)	0.47	0.77

PS Matching

SIDIAP

Supplementary Table 201: Risk estimates for COVID-19 diagnosis across propensity score matched, prevalent-user cohorts in the SIDIAP data source. We report uncalibrated and calibrated hazard ratios (HRs) and their 95% confidence intervals (CIs). (+) indicates in-combination cohorts.

			Uncalibrated			Calibrated	
Target	Comparator	HR	95% CI	p	HR	95% CI	p
ACE/ARB	CCB/THZ	1.12	(0.90 - 1.39)	0.31	1.16	(0.93 - 1.44)	0.24
ACE/ARB+	CCB/THZ+	0.99	(0.82 - 1.20)	0.93	1.02	(0.85 - 1.23)	0.76
ACE/ARB	CCB	1.21	(0.93 - 1.59)	0.16	1.21	(0.93 - 1.58)	0.21
ACE/ARB+	CCB+	1.15	(0.94 - 1.41)	0.17	1.13	(0.93 - 1.39)	0.28
ACE/ARB	THZ	0.92	(0.68 - 1.26)	0.57	1.02	(0.72 - 1.44)	0.80
ACE/ARB+	THZ+	1.13	(0.88 - 1.47)	0.36	1.25	(0.96 - 1.62)	0.12
ACE	ARB	0.91	(0.69 - 1.19)	0.47	1.01	(0.77 - 1.32)	0.79
ACE+	ARB+	0.90	(0.76 - 1.07)	0.23	0.95	(0.74 - 1.22)	0.67
ACE	CCB/THZ	1.10	(0.88 - 1.39)	0.41	1.14	(0.90 - 1.43)	0.34
ACE+	CCB/THZ+	0.91	(0.76 - 1.09)	0.30	0.95	(0.79 - 1.15)	0.66
ACE	CCB	1.05	(0.80 - 1.39)	0.72	1.06	(0.76 - 1.46)	0.73
ACE+	CCB+	0.99	(0.82 - 1.21)	0.96	0.98	(0.81 - 1.19)	0.78
ACE	THZ	0.93	(0.68 - 1.29)	0.65	1.02	(0.74 - 1.40)	0.81
ACE+	THZ+	0.97	(0.79 - 1.20)	0.77	1.02	(0.83 - 1.26)	0.78
ARB	CCB/THZ	1.10	(0.77 - 1.56)	0.60	0.98	(0.69 - 1.39)	0.81
ARB+	CCB/THZ+	1.11	(0.88 - 1.39)	0.38	1.06	(0.84 - 1.33)	0.65
ARB	CCB	1.11	(0.73 - 1.67)	0.63	1.01	(0.67 - 1.52)	0.82
ARB+	CCB+	1.21	(0.94 - 1.56)	0.14	1.18	(0.88 - 1.57)	0.32
ARB	THZ	0.94	(0.58 - 1.52)	0.79	0.98	(0.61 - 1.59)	0.81
ARB+	THZ+	1.19	(0.92 - 1.54)	0.18	1.14	(0.88 - 1.48)	0.35

Supplementary Table 202: Risk estimates for COVID-19 hospitalization across propensity score matched, prevalent-user cohorts in the SIDIAP data source. We report uncalibrated and calibrated hazard ratios (HRs) and their 95% confidence intervals (CIs). (+) indicates in-combination cohorts.

			Uncalibrated			Calibrated	
Target	Comparator	HR	95% CI	p	HR	95% CI	p
ACE/ARB	CCB/THZ	1.03	(0.74 - 1.46)	0.86	1.07	(0.76 - 1.50)	0.73
ACE/ARB+	CCB/THZ+	0.92	(0.69 - 1.26)	0.61	0.96	(0.71 - 1.29)	0.77
ACE/ARB	CCB	1.19	(0.78 - 1.87)	0.43	1.19	(0.77 - 1.85)	0.45
ACE/ARB+	CCB+	1.15	(0.85 - 1.57)	0.37	1.13	(0.84 - 1.53)	0.45
ACE/ARB	THZ	1.00	(0.61 - 1.72)	0.99	1.11	(0.64 - 1.92)	0.72
ACE/ARB+	THZ+	1.19	(0.77 - 1.90)	0.45	1.31	(0.84 - 2.05)	0.24
ACE	ARB	1.35	(0.83 - 2.28)	0.24	1.50	(0.91 - 2.49)	0.12
ACE+	ARB+	0.89	(0.66 - 1.19)	0.41	0.94	(0.66 - 1.32)	0.70
ACE	CCB/THZ	0.98	(0.67 - 1.44)	0.90	1.00	(0.69 - 1.47)	0.87
ACE+	CCB/THZ+	0.97	(0.72 - 1.31)	0.84	1.02	(0.76 - 1.37)	0.86
ACE	CCB	1.02	(0.66 - 1.61)	0.94	1.02	(0.63 - 1.65)	0.87
ACE+	CCB+	0.84	(0.63 - 1.14)	0.25	0.83	(0.61 - 1.12)	0.26
ACE	THZ	0.91	(0.55 - 1.59)	0.74	1.01	(0.59 - 1.71)	0.87
ACE+	THZ+	1.04	(0.74 - 1.49)	0.82	1.10	(0.77 - 1.56)	0.63
ARB	CCB/THZ	0.81	(0.42 - 1.51)	0.52	0.72	(0.38 - 1.37)	0.33
ARB+	CCB/THZ+	0.89	(0.59 - 1.29)	0.54	0.84	(0.57 - 1.24)	0.41
ARB	CCB	0.91	(0.45 - 1.79)	0.79	0.83	(0.42 - 1.65)	0.61
ARB+	CCB+	1.04	(0.69 - 1.57)	0.83	1.02	(0.66 - 1.56)	0.87
ARB	THZ	0.61	(0.27 - 1.36)	0.23	0.64	(0.29 - 1.43)	0.29
ARB+	THZ+	1.40	(0.89 - 2.21)	0.15	1.34	(0.85 - 2.12)	0.22

Supplementary Table 203: Risk estimates for pneumonia across propensity score matched, prevalent-user cohorts in the SIDIAP data source. We report uncalibrated and calibrated hazard ratios (HRs) and their 95% confidence intervals (CIs). (+) indicates in-combination cohorts.

		Uncalibrated			Calibrated		
Target	Comparator	HR	95% CI	p	HR	95% CI	p
ACE/ARB	CCB/THZ	0.96	(0.51 - 1.87)	0.91	1.00	(0.52 - 1.90)	0.93
ACE/ARB+	CCB/THZ+	0.83	(0.49 - 1.42)	0.49	0.86	(0.50 - 1.45)	0.57
ACE/ARB	CCB	1.38	(0.58 - 3.81)	0.50	1.38	(0.54 - 3.53)	0.50
ACE/ARB+	CCB+	1.03	(0.58 - 1.94)	0.93	1.01	(0.55 - 1.86)	0.92
ACE/ARB	THZ	0.92	(0.40 - 2.43)	0.86	1.03	(0.41 - 2.58)	0.91
ACE/ARB+	THZ+	0.93	(0.46 - 2.05)	0.85	1.03	(0.49 - 2.17)	0.91
ACE	ARB	0.56	(0.25 - 1.30)	0.17	0.62	(0.27 - 1.43)	0.27
ACE+	ARB+	1.07	(0.62 - 1.90)	0.81	1.13	(0.63 - 2.04)	0.67
ACE	CCB/THZ	1.14	(0.55 - 2.52)	0.73	1.18	(0.55 - 2.53)	0.68
ACE+	CCB/THZ+	0.96	(0.54 - 1.72)	0.88	1.01	(0.56 - 1.80)	0.93
ACE	CCB	1.05	(0.41 - 3.02)	0.92	1.05	(0.38 - 2.89)	0.91
ACE+	CCB+	0.80	(0.45 - 1.47)	0.47	0.79	(0.44 - 1.43)	0.45
ACE	THZ	0.71	(0.26 - 2.27)	0.53	0.78	(0.26 - 2.30)	0.65
ACE+	THZ+	0.98	(0.52 - 1.93)	0.96	1.03	(0.54 - 2.00)	0.91
ARB	CCB/THZ	1.06	(0.27 - 3.60)	0.93	0.94	(0.26 - 3.44)	0.92
ARB+	CCB/THZ+	0.98	(0.50 - 1.82)	0.94	0.93	(0.49 - 1.77)	0.83
ARB	CCB	1.41	(0.44 - 4.59)	0.56	1.29	(0.40 - 4.19)	0.67
ARB+	CCB+	0.69	(0.33 - 1.40)	0.32	0.67	(0.32 - 1.41)	0.30
ARB	THZ	0.44	(0.11 - 1.62)	0.24	0.47	(0.12 - 1.79)	0.27
ARB+	THZ+	1.30	(0.51 - 3.23)	0.58	1.24	(0.49 - 3.12)	0.65

Supplementary Table 204: Risk estimates for PAAS across propensity score matched, prevalent-user cohorts in the SIDIAP data source. We report uncalibrated and calibrated hazard ratios (HRs) and their 95% confidence intervals (CIs). (+) indicates in-combination cohorts.

			Uncalibrated			Calibrated	
Target	Comparator	HR	95% CI	p	HR	95% CI	p
ACE/ARB	CCB/THZ	0.99	(0.54 - 1.88)	0.99	1.03	(0.55 - 1.92)	0.91
ACE/ARB+	CCB/THZ+	0.83	(0.50 - 1.38)	0.46	0.85	(0.51 - 1.42)	0.55
ACE/ARB	CCB	1.37	(0.61 - 3.49)	0.48	1.37	(0.57 - 3.27)	0.48
ACE/ARB+	CCB+	1.00	(0.58 - 1.81)	0.99	0.98	(0.55 - 1.74)	0.92
ACE/ARB	THZ	0.92	(0.40 - 2.43)	0.86	1.03	(0.41 - 2.58)	0.91
ACE/ARB+	THZ+	0.84	(0.43 - 1.79)	0.63	0.93	(0.45 - 1.90)	0.84
ACE	ARB	0.66	(0.30 - 1.49)	0.31	0.73	(0.33 - 1.63)	0.46
ACE+	ARB+	1.08	(0.64 - 1.88)	0.79	1.14	(0.64 - 2.02)	0.65
ACE	CCB/THZ	1.18	(0.58 - 2.49)	0.66	1.21	(0.59 - 2.50)	0.61
ACE+	CCB/THZ+	0.97	(0.56 - 1.72)	0.92	1.02	(0.58 - 1.80)	0.92
ACE	CCB	1.15	(0.48 - 3.03)	0.77	1.15	(0.45 - 2.93)	0.77
ACE+	CCB+	0.83	(0.48 - 1.49)	0.53	0.82	(0.47 - 1.45)	0.51
ACE	THZ	0.73	(0.27 - 2.34)	0.57	0.81	(0.28 - 2.36)	0.69
ACE+	THZ+	0.89	(0.49 - 1.69)	0.72	0.94	(0.50 - 1.75)	0.85
ARB	CCB/THZ	0.87	(0.23 - 2.84)	0.83	0.77	(0.22 - 2.74)	0.69
ARB+	CCB/THZ+	0.97	(0.51 - 1.77)	0.93	0.92	(0.50 - 1.72)	0.81
ARB	CCB	1.41	(0.44 - 4.59)	0.56	1.29	(0.40 - 4.19)	0.67
ARB+	CCB+	0.74	(0.36 - 1.47)	0.41	0.72	(0.35 - 1.48)	0.38
ARB	THZ	0.44	(0.11 - 1.62)	0.24	0.47	(0.12 - 1.79)	0.27
ARB+	THZ+	1.07	(0.45 - 2.44)	0.88	1.02	(0.44 - 2.38)	0.93

VA-OMOP

Supplementary Table 205: Risk estimates for COVID-19 diagnosis across propensity score matched, prevalent-user cohorts in the VA-OMOP data source. We report uncalibrated and calibrated hazard ratios (HRs) and their 95% confidence intervals (CIs). (+) indicates in-combination cohorts.

		Uncalibrated			Calibrated		
Target	Comparator	HR	95% CI	p	HR	95% CI	p
ACE/ARB	CCB/THZ	1.04	(0.75 - 1.44)	0.81	1.06	(0.76 - 1.46)	0.74
ACE/ARB+	CCB/THZ+	1.03	(0.83 - 1.27)	0.79	1.03	(0.83 - 1.29)	0.77
ACE/ARB	CCB	0.94	(0.67 - 1.32)	0.70	0.95	(0.66 - 1.36)	0.77
ACE/ARB+	CCB+	0.94	(0.77 - 1.14)	0.52	0.94	(0.77 - 1.16)	0.56
ACE/ARB	THZ	0.83	(0.53 - 1.34)	0.43	0.87	(0.55 - 1.38)	0.57
ACE/ARB+	THZ+	1.26	(0.97 - 1.64)	0.09	1.29	(0.97 - 1.73)	0.08
ACE	ARB	0.76	(0.52 - 1.12)	0.15	0.75	(0.52 - 1.10)	0.15
ACE+	ARB+	0.84	(0.70 - 1.00)	0.05	0.85	(0.71 - 1.02)	0.08
ACE	CCB/THZ	1.00	(0.70 - 1.44)	0.99	1.00	(0.70 - 1.44)	0.96
ACE+	CCB/THZ+	0.86	(0.70 - 1.06)	0.16	0.87	(0.70 - 1.08)	0.21
ACE	CCB	0.70	(0.48 - 1.03)	0.07	0.70	(0.47 - 1.04)	0.08
ACE+	CCB+	0.88	(0.72 - 1.08)	0.23	0.89	(0.73 - 1.10)	0.28
ACE	THZ	0.78	(0.48 - 1.29)	0.32	0.81	(0.49 - 1.33)	0.41
ACE+	THZ+	1.14	(0.90 - 1.45)	0.30	1.18	(0.92 - 1.52)	0.20
ARB	CCB/THZ	1.18	(0.79 - 1.74)	0.40	1.18	(0.80 - 1.76)	0.41
ARB+	CCB/THZ+	1.16	(0.92 - 1.46)	0.19	1.17	(0.93 - 1.47)	0.19
ARB	CCB	1.17	(0.75 - 1.79)	0.49	1.17	(0.75 - 1.81)	0.49
ARB+	CCB+	1.12	(0.90 - 1.38)	0.32	1.11	(0.89 - 1.38)	0.37
ARB	THZ	0.92	(0.50 - 1.76)	0.80	0.95	(0.51 - 1.79)	0.88
ARB+	THZ+	1.29	(1.00 - 1.65)	0.05	1.31	(1.02 - 1.68)	0.03

Supplementary Table 206: Risk estimates for COVID-19 hospitalization across propensity score matched, prevalent-user cohorts in the VA-OMOP data source. We report uncalibrated and calibrated hazard ratios (HRs) and their 95% confidence intervals (CIs). (+) indicates in-combination cohorts.

			Uncalibrated			Calibrated	
Target	Comparator	HR	95% CI	p	HR	95% CI	p
ACE/ARB	CCB/THZ	1.27	(0.86 - 1.91)	0.24	1.29	(0.87 - 1.92)	0.21
ACE/ARB+	CCB/THZ+	1.03	(0.83 - 1.27)	0.81	1.03	(0.83 - 1.28)	0.79
ACE/ARB	CCB	0.98	(0.66 - 1.48)	0.91	0.99	(0.65 - 1.51)	0.96
ACE/ARB+	CCB+	1.04	(0.85 - 1.28)	0.70	1.05	(0.84 - 1.30)	0.69
ACE/ARB	THZ	1.21	(0.66 - 2.36)	0.55	1.28	(0.68 - 2.40)	0.45
ACE/ARB+	THZ+	1.39	(1.05 - 1.86)	0.03	1.43	(1.05 - 1.94)	0.02
ACE	ARB	0.69	(0.45 - 1.06)	0.08	0.68	(0.45 - 1.05)	0.08
ACE+	ARB+	0.93	(0.78 - 1.11)	0.42	0.94	(0.78 - 1.13)	0.51
ACE	CCB/THZ	1.12	(0.73 - 1.74)	0.60	1.13	(0.73 - 1.74)	0.58
ACE+	CCB/THZ+	0.99	(0.81 - 1.21)	0.93	1.00	(0.80 - 1.24)	0.95
ACE	CCB	0.87	(0.56 - 1.38)	0.55	0.87	(0.55 - 1.37)	0.56
ACE+	CCB+	0.93	(0.76 - 1.14)	0.50	0.94	(0.76 - 1.16)	0.57
ACE	THZ	1.66	(0.84 - 3.59)	0.17	1.72	(0.83 - 3.56)	0.14
ACE+	THZ+	1.11	(0.87 - 1.43)	0.42	1.15	(0.89 - 1.50)	0.29
ARB	CCB/THZ	1.72	(1.08 - 2.72)	0.02	1.72	(1.08 - 2.74)	0.02
ARB+	CCB/THZ+	0.97	(0.76 - 1.22)	0.79	0.97	(0.77 - 1.23)	0.81
ARB	CCB	1.45	(0.89 - 2.32)	0.13	1.45	(0.89 - 2.35)	0.14
ARB+	CCB+	1.10	(0.87 - 1.37)	0.42	1.09	(0.87 - 1.37)	0.47
ARB	THZ	1.85	(0.79 - 5.08)	0.20	1.91	(0.75 - 4.85)	0.18
ARB+	THZ+	1.14	(0.87 - 1.48)	0.34	1.16	(0.89 - 1.52)	0.27

Supplementary Table 207: Risk estimates for pneumonia across propensity score matched, prevalent-user cohorts in the VA-OMOP data source. We report uncalibrated and calibrated hazard ratios (HRs) and their 95% confidence intervals (CIs). (+) indicates in-combination cohorts.

Target	Comparator	Uncalibrated HR	Uncalibrated 95% CI	Uncalibrated p	Calibrated HR	Calibrated 95% CI	Calibrated p
ACE/ARB	CCB/THZ	0.89	(0.73 - 1.10)	0.29	0.91	(0.74 - 1.12)	0.38
ACE/ARB+	CCB/THZ+	0.96	(0.85 - 1.09)	0.54	0.97	(0.84 - 1.11)	0.62
ACE/ARB	CCB	0.94	(0.75 - 1.19)	0.61	0.96	(0.74 - 1.23)	0.72
ACE/ARB+	CCB+	1.00	(0.89 - 1.13)	0.98	1.00	(0.87 - 1.15)	0.93
ACE/ARB	THZ	0.92	(0.65 - 1.33)	0.65	0.97	(0.68 - 1.38)	0.86
ACE/ARB+	THZ+	1.11	(0.94 - 1.32)	0.21	1.15	(0.94 - 1.40)	0.18
ACE	ARB	0.92	(0.70 - 1.22)	0.54	0.91	(0.69 - 1.21)	0.53
ACE+	ARB+	0.98	(0.89 - 1.09)	0.75	0.99	(0.89 - 1.12)	0.91
ACE	CCB/THZ	0.92	(0.73 - 1.17)	0.49	0.92	(0.73 - 1.17)	0.54
ACE+	CCB/THZ+	0.98	(0.87 - 1.11)	0.77	0.99	(0.85 - 1.14)	0.85
ACE	CCB	0.93	(0.73 - 1.20)	0.59	0.93	(0.72 - 1.20)	0.61
ACE+	CCB+	1.02	(0.90 - 1.15)	0.77	1.03	(0.91 - 1.17)	0.67
ACE	THZ	0.98	(0.68 - 1.45)	0.92	1.02	(0.70 - 1.49)	0.91
ACE+	THZ+	1.03	(0.89 - 1.19)	0.72	1.07	(0.90 - 1.26)	0.44
ARB	CCB/THZ	1.05	(0.77 - 1.41)	0.74	1.05	(0.78 - 1.42)	0.74
ARB+	CCB/THZ+	0.92	(0.79 - 1.05)	0.23	0.92	(0.80 - 1.06)	0.26
ARB	CCB	1.16	(0.84 - 1.59)	0.36	1.16	(0.84 - 1.61)	0.37
ARB+	CCB+	0.97	(0.84 - 1.12)	0.70	0.97	(0.83 - 1.12)	0.66
ARB	THZ	0.95	(0.59 - 1.55)	0.84	0.98	(0.61 - 1.58)	0.93
ARB+	THZ+	0.97	(0.82 - 1.14)	0.71	0.99	(0.84 - 1.17)	0.90

Supplementary Table 208: Risk estimates for PAAS across propensity score matched, prevalent-user cohorts in the VA-OMOP data source. We report uncalibrated and calibrated hazard ratios (HRs) and their 95% confidence intervals (CIs). (+) indicates in-combination cohorts.

Target	Comparator	Uncalibrated HR	Uncalibrated 95% CI	Uncalibrated p	Calibrated HR	Calibrated 95% CI	Calibrated p
ACE/ARB	CCB/THZ	1.14	(0.99 - 1.32)	0.07	1.16	(1.00 - 1.34)	0.06
ACE/ARB+	CCB/THZ+	1.16	(1.06 - 1.27)	< 0.01	1.16	(1.04 - 1.29)	< 0.01
ACE/ARB	CCB	1.17	(1.00 - 1.38)	0.05	1.19	(0.99 - 1.44)	0.06
ACE/ARB+	CCB+	1.23	(1.12 - 1.34)	< 0.01	1.23	(1.10 - 1.37)	< 0.01
ACE/ARB	THZ	1.02	(0.81 - 1.29)	0.87	1.07	(0.85 - 1.35)	0.57
ACE/ARB+	THZ+	1.05	(0.94 - 1.17)	0.37	1.08	(0.92 - 1.27)	0.31
ACE	ARB	1.05	(0.88 - 1.27)	0.58	1.05	(0.87 - 1.26)	0.62
ACE+	ARB+	1.09	(1.02 - 1.17)	0.02	1.10	(1.01 - 1.20)	0.03
ACE	CCB/THZ	1.13	(0.96 - 1.33)	0.14	1.14	(0.97 - 1.34)	0.13
ACE+	CCB/THZ+	1.18	(1.08 - 1.28)	< 0.01	1.18	(1.05 - 1.33)	< 0.01
ACE	CCB	1.15	(0.97 - 1.37)	0.11	1.15	(0.96 - 1.38)	0.14
ACE+	CCB+	1.22	(1.12 - 1.33)	< 0.01	1.23	(1.12 - 1.35)	< 0.01
ACE	THZ	1.13	(0.89 - 1.45)	0.33	1.17	(0.92 - 1.50)	0.20
ACE+	THZ+	1.00	(0.91 - 1.10)	0.97	1.04	(0.92 - 1.18)	0.52
ARB	CCB/THZ	1.12	(0.91 - 1.37)	0.28	1.12	(0.91 - 1.38)	0.31
ARB+	CCB/THZ+	0.97	(0.88 - 1.07)	0.54	0.97	(0.88 - 1.07)	0.61
ARB	CCB	1.19	(0.95 - 1.48)	0.13	1.19	(0.95 - 1.49)	0.15
ARB+	CCB+	1.06	(0.96 - 1.17)	0.25	1.05	(0.94 - 1.17)	0.38
ARB	THZ	0.96	(0.71 - 1.30)	0.77	0.99	(0.73 - 1.34)	0.91
ARB+	THZ+	0.83	(0.74 - 0.92)	< 0.01	0.84	(0.76 - 0.94)	< 0.01

CUIMC

Supplementary Table 209: Risk estimates for COVID-19 diagnosis across propensity score matched, prevalent-user cohorts in the CUIMC data source. We report uncalibrated and calibrated hazard ratios (HRs) and their 95% confidence intervals (CIs). (+) indicates in-combination cohorts.

Target	Comparator	HR	Uncalibrated 95% CI	p	HR	Calibrated 95% CI	p
ACE/ARB	CCB/THZ	0.86	(0.25 - 2.76)	0.81	0.67	(0.20 - 2.20)	0.53
ACE/ARB+	CCB/THZ+	2.71	(1.17 - 6.75)	0.03	2.36	(0.98 - 5.68)	0.06
ACE/ARB	CCB	1.40	(0.35 - 6.79)	0.66	1.29	(0.29 - 5.62)	0.74
ACE/ARB+	CCB+	1.66	(0.80 - 3.72)	0.19	1.78	(0.82 - 3.85)	0.15
ACE/ARB	THZ	1.17	(0.22 - 8.65)	0.87	1.84	(0.29 - 11.58)	0.52
ACE/ARB+	THZ+	1.70	(0.64 - 5.38)	0.33	2.08	(0.71 - 6.04)	0.18
ACE	ARB	0.29	(0.01 - 2.06)	0.33	0.22	(0.02 - 2.63)	0.24
ACE+	ARB+	0.53	(0.20 - 1.28)	0.18	0.54	(0.21 - 1.36)	0.20
ACE	CCB/THZ	1.44	(0.38 - 5.45)	0.59	0.92	(0.24 - 3.48)	0.86
ACE+	CCB/THZ+	0.57	(0.20 - 1.40)	0.25	0.45	(0.17 - 1.18)	0.11
ACE	CCB	1.29	(0.30 - 5.57)	0.73	0.71	(0.16 - 3.04)	0.65
ACE+	CCB+	1.10	(0.38 - 2.99)	0.86	0.85	(0.31 - 2.39)	0.77
ACE	THZ	1.87	(0.22 - 39.01)	0.64	2.10	(0.16 - 27.78)	0.57
ACE+	THZ+	1.41	(0.37 - 5.81)	0.63	1.33	(0.33 - 5.28)	0.69
ARB	CCB/THZ	2.90	(0.60 - 15.94)	0.20	2.25	(0.43 - 11.61)	0.34
ARB+	CCB/THZ+	1.95	(0.90 - 4.25)	0.09	1.31	(0.61 - 2.85)	0.51
ARB	CCB	1.61	(0.17 - 16.38)	0.68	0.93	(0.10 - 9.07)	0.88
ARB+	CCB+	2.67	(1.11 - 6.97)	0.04	2.00	(0.80 - 5.00)	0.15
ARB	THZ	1.15	(0.19 - 8.89)	0.89	1.47	(0.21 - 10.14)	0.70
ARB+	THZ+	1.26	(0.39 - 4.32)	0.70	1.19	(0.36 - 3.93)	0.78

Supplementary Table 210: Risk estimates for COVID-19 hospitalization across propensity score matched, prevalent-user cohorts in the CUIMC data source. We report uncalibrated and calibrated hazard ratios (HRs) and their 95% confidence intervals (CIs). (+) indicates in-combination cohorts.

			Uncalibrated			Calibrated	
Target	Comparator	HR	95% CI	p	HR	95% CI	p
ACE/ARB	CCB/THZ	1.35	(0.61 - 2.96)	0.45	1.04	(0.47 - 2.31)	0.80
ACE/ARB+	CCB/THZ+	1.03	(0.61 - 1.70)	0.92	0.89	(0.53 - 1.49)	0.67
ACE/ARB	CCB	1.08	(0.47 - 2.56)	0.86	0.99	(0.42 - 2.32)	0.81
ACE/ARB+	CCB+	1.31	(0.80 - 2.18)	0.29	1.40	(0.85 - 2.31)	0.21
ACE/ARB	THZ	1.95	(0.31 - 38.20)	0.59	3.07	(0.28 - 34.01)	0.36
ACE/ARB+	THZ+	1.65	(0.83 - 3.57)	0.18	2.01	(0.97 - 4.16)	0.06
ACE	ARB	0.80	(0.20 - 3.02)	0.75	0.61	(0.16 - 2.37)	0.50
ACE+	ARB+	1.07	(0.62 - 1.84)	0.80	1.09	(0.63 - 1.87)	0.74
ACE	CCB/THZ	1.10	(0.45 - 2.48)	0.83	0.70	(0.30 - 1.65)	0.47
ACE+	CCB/THZ+	1.17	(0.69 - 1.93)	0.54	0.92	(0.55 - 1.54)	0.73
ACE	CCB	1.69	(0.70 - 4.00)	0.24	0.92	(0.39 - 2.21)	0.73
ACE+	CCB+	0.83	(0.48 - 1.38)	0.49	0.65	(0.38 - 1.10)	0.12
ACE	THZ	1.60	(0.26 - 30.50)	0.70	1.79	(0.17 - 19.26)	0.63
ACE+	THZ+	1.62	(0.84 - 3.17)	0.15	1.52	(0.79 - 2.95)	0.24
ARB	CCB/THZ	0.83	(0.23 - 2.43)	0.76	0.65	(0.20 - 2.11)	0.47
ARB+	CCB/THZ+	0.89	(0.46 - 1.60)	0.70	0.60	(0.32 - 1.11)	0.12
ARB	CCB	0.59	(0.13 - 1.90)	0.44	0.34	(0.09 - 1.30)	0.12
ARB+	CCB+	1.04	(0.59 - 1.76)	0.90	0.77	(0.45 - 1.34)	0.40
ARB	THZ	0.59	(0.08 - 5.19)	0.63	0.76	(0.09 - 6.13)	0.79
ARB+	THZ+	2.31	(1.01 - 5.93)	0.06	2.17	(0.90 - 5.25)	0.10

Supplementary Table 211: Risk estimates for pneumonia across propensity score matched, prevalent-user cohorts in the CUIMC data source. We report uncalibrated and calibrated hazard ratios (HRs) and their 95% confidence intervals (CIs). (+) indicates in-combination cohorts.

Target	Comparator	HR	Uncalibrated 95% CI	p	HR	Calibrated 95% CI	p
ACE/ARB	CCB/THZ	1.74	(0.39 - 7.50)	0.46	1.34	(0.31 - 5.89)	0.69
ACE/ARB+	CCB/THZ+	1.38	(0.53 - 3.42)	0.50	1.20	(0.47 - 3.06)	0.71
ACE/ARB	CCB	1.00	(0.24 - 4.23)	1.00	0.92	(0.22 - 3.89)	0.86
ACE/ARB+	CCB+	2.60	(1.00 - 8.02)	0.07	2.78	(0.98 - 7.86)	0.06
ACE/ARB	THZ	0.49	(0.09 - 3.91)	0.47	0.78	(0.12 - 5.21)	0.80
ACE/ARB+	THZ+	2.46	(0.66 - 16.08)	0.27	3.00	(0.61 - 14.87)	0.18
ACE	ARB	0.50	(0.02 - 5.22)	0.62	0.38	(0.03 - 5.69)	0.49
ACE+	ARB+	0.62	(0.24 - 1.50)	0.32	0.63	(0.25 - 1.60)	0.34
ACE	CCB/THZ	0.62	(0.09 - 2.94)	0.60	0.40	(0.07 - 2.31)	0.32
ACE+	CCB/THZ+	0.62	(0.14 - 1.91)	0.47	0.49	(0.13 - 1.79)	0.28
ACE	CCB	1.56	(0.20 - 9.50)	0.65	0.85	(0.12 - 5.80)	0.85
ACE+	CCB+	0.53	(0.12 - 1.67)	0.35	0.42	(0.11 - 1.55)	0.20
ACE	THZ	0.27	(0.01 - 7.38)	0.44	0.30	(0.01 - 8.30)	0.48
ACE+	THZ+	1.04	(0.36 - 2.94)	0.95	0.98	(0.34 - 2.80)	0.87
ARB	CCB/THZ	4.59	(0.97 - 21.37)	0.05	3.55	(0.75 - 16.68)	0.12
ARB+	CCB/THZ+	1.59	(0.58 - 4.00)	0.35	1.07	(0.41 - 2.81)	0.86
ARB	CCB	1.36	(0.17 - 7.33)	0.75	0.79	(0.12 - 5.13)	0.80
ARB+	CCB+	1.99	(0.83 - 4.71)	0.12	1.48	(0.62 - 3.54)	0.39
ARB+	THZ+	3.49	(0.88 - 23.15)	0.13	3.29	(0.64 - 16.86)	0.16

Supplementary Table 212: Risk estimates for PAAS across propensity score matched, prevalent-user cohorts in the CUIMC data source. We report uncalibrated and calibrated hazard ratios (HRs) and their 95% confidence intervals (CIs). (+) indicates in-combination cohorts.

		Uncalibrated			Calibrated		
Target	Comparator	HR	95% CI	p	HR	95% CI	p
ACE/ARB	CCB/THZ	1.42	(0.53 - 3.58)	0.47	1.09	(0.42 - 2.83)	0.81
ACE/ARB+	CCB/THZ+	1.12	(0.54 - 2.40)	0.78	0.97	(0.46 - 2.06)	0.85
ACE/ARB	CCB	1.22	(0.42 - 3.71)	0.73	1.12	(0.38 - 3.32)	0.81
ACE/ARB+	CCB+	2.52	(1.19 - 5.97)	0.02	2.70	(1.21 - 6.04)	0.02
ACE/ARB+	THZ+	2.10	(0.81 - 7.22)	0.18	2.57	(0.86 - 7.68)	0.09
ACE	ARB	0.67	(0.09 - 4.02)	0.68	0.51	(0.07 - 3.43)	0.50
ACE+	ARB+	0.51	(0.26 - 0.95)	0.04	0.52	(0.27 - 0.99)	0.05
ACE	CCB/THZ	0.56	(0.13 - 1.80)	0.40	0.36	(0.10 - 1.36)	0.15
ACE+	CCB/THZ+	0.93	(0.40 - 1.97)	0.87	0.74	(0.33 - 1.63)	0.46
ACE	CCB	1.30	(0.33 - 4.30)	0.69	0.71	(0.20 - 2.55)	0.61
ACE+	CCB+	0.67	(0.30 - 1.35)	0.29	0.52	(0.25 - 1.09)	0.09
ACE+	THZ+	1.12	(0.38 - 3.24)	0.84	1.05	(0.36 - 3.07)	0.87
ARB	CCB/THZ	2.21	(0.61 - 7.29)	0.21	1.71	(0.50 - 5.89)	0.41
ARB+	CCB/THZ+	1.40	(0.64 - 2.85)	0.38	0.94	(0.45 - 1.99)	0.83
ARB	CCB	1.26	(0.26 - 4.52)	0.75	0.73	(0.18 - 3.04)	0.67
ARB+	CCB+	1.60	(0.88 - 2.88)	0.12	1.20	(0.66 - 2.17)	0.58
ARB+	THZ+	2.56	(0.99 - 7.89)	0.07	2.41	(0.86 - 6.79)	0.10

Meta-analysis

These meta-analytic estimates are based on all data source estimates, not just those that pass diagnostics

Supplementary Table 213: Risk estimates for COVID-19 diagnosis across propensity score matched, prevalent-user cohorts in the Meta-analysis across data sources. We report uncalibrated and calibrated hazard ratios (HRs) and their 95% confidence intervals (CIs). (+) indicates in-combination cohorts.

			Uncalibrated			Calibrated		
Target	Comparator	HR	95% CI	p	HR	95% CI	p	i^2
ACE/ARB	CCB/THZ	1.09	(0.91 - 1.30)	0.35	1.10	(0.92 - 1.32)	0.31	0.00
ACE/ARB+	CCB/THZ+	1.09	(0.84 - 1.41)	0.52	1.09	(0.84 - 1.41)	0.54	0.59
ACE/ARB	CCB	1.10	(0.90 - 1.36)	0.35	1.12	(0.88 - 1.43)	0.35	0.00
ACE/ARB+	CCB+	1.07	(0.87 - 1.31)	0.52	1.06	(0.86 - 1.31)	0.57	0.42
ACE/ARB	THZ	0.89	(0.69 - 1.15)	0.38	0.94	(0.73 - 1.21)	0.65	0.00
ACE/ARB+	THZ+	1.20	(1.00 - 1.44)	0.05	1.26	(1.01 - 1.57)	0.04	0.00
ACE	ARB	0.85	(0.68 - 1.05)	0.14	0.85	(0.68 - 1.06)	0.16	0.00
ACE+	ARB+	0.86	(0.76 - 0.97)	0.02	0.87	(0.77 - 0.99)	0.04	0.00
ACE	CCB/THZ	1.08	(0.89 - 1.31)	0.45	1.08	(0.89 - 1.31)	0.45	0.00
ACE+	CCB/THZ+	0.88	(0.77 - 1.01)	0.06	0.88	(0.75 - 1.04)	0.13	0.00
ACE	CCB	0.91	(0.66 - 1.24)	0.54	0.91	(0.65 - 1.27)	0.54	0.34
ACE+	CCB+	0.94	(0.82 - 1.08)	0.40	0.94	(0.82 - 1.08)	0.42	0.00
ACE	THZ	0.89	(0.68 - 1.16)	0.39	0.93	(0.71 - 1.22)	0.61	0.00
ACE+	THZ+	1.04	(0.89 - 1.22)	0.61	1.09	(0.93 - 1.28)	0.31	0.00
ARB	CCB/THZ	1.16	(0.90 - 1.51)	0.26	1.16	(0.89 - 1.50)	0.28	0.00
ARB+	CCB/THZ+	1.16	(0.99 - 1.36)	0.06	1.16	(0.99 - 1.36)	0.08	0.00
ARB	CCB	1.14	(0.85 - 1.54)	0.38	1.13	(0.84 - 1.52)	0.42	0.00
ARB+	CCB+	1.22	(0.96 - 1.56)	0.10	1.21	(0.94 - 1.56)	0.13	0.40
ARB	THZ	0.94	(0.64 - 1.37)	0.74	0.97	(0.67 - 1.41)	0.88	0.00
ARB+	THZ+	1.24	(1.04 - 1.48)	0.02	1.26	(1.06 - 1.50)	0.01	0.00

Supplementary Table 214: Risk estimates for COVID-19 hospitalization across propensity score matched, prevalent-user cohorts in the Meta-analysis across data sources. We report uncalibrated and calibrated hazard ratios (HRs) and their 95% confidence intervals (CIs). (+) indicates in-combination cohorts.

			Uncalibrated			Calibrated		
Target	Comparator	HR	95% CI	p	HR	95% CI	p	i^2
ACE/ARB	CCB/THZ	1.15	(0.90 - 1.47)	0.28	1.16	(0.90 - 1.49)	0.25	0.00
ACE/ARB+	CCB/THZ+	1.00	(0.85 - 1.17)	0.96	0.99	(0.84 - 1.18)	0.93	0.00
ACE/ARB	CCB	1.07	(0.81 - 1.42)	0.63	1.09	(0.80 - 1.48)	0.59	0.00
ACE/ARB+	CCB+	1.10	(0.93 - 1.29)	0.26	1.09	(0.92 - 1.29)	0.31	0.00
ACE/ARB	THZ	1.10	(0.74 - 1.63)	0.65	1.16	(0.78 - 1.72)	0.48	0.00
ACE/ARB+	THZ+	1.35	(1.08 - 1.70)	< 0.01	1.42	(1.09 - 1.84)	< 0.01	0.00
ACE	ARB	0.93	(0.55 - 1.55)	0.77	0.93	(0.56 - 1.55)	0.78	0.51
ACE+	ARB+	0.93	(0.80 - 1.07)	0.32	0.94	(0.81 - 1.09)	0.41	0.00
ACE	CCB/THZ	1.04	(0.80 - 1.37)	0.76	1.04	(0.80 - 1.37)	0.75	0.00
ACE+	CCB/THZ+	1.00	(0.85 - 1.17)	0.99	1.01	(0.84 - 1.21)	0.93	0.00
ACE	CCB	1.01	(0.75 - 1.36)	0.96	1.01	(0.74 - 1.39)	0.94	0.00
ACE+	CCB+	0.89	(0.76 - 1.05)	0.18	0.89	(0.76 - 1.05)	0.19	0.00
ACE	THZ	1.14	(0.75 - 1.74)	0.55	1.19	(0.78 - 1.82)	0.42	0.00
ACE+	THZ+	1.12	(0.92 - 1.37)	0.24	1.17	(0.96 - 1.44)	0.12	0.00
ARB	CCB/THZ	1.16	(0.65 - 2.04)	0.62	1.15	(0.65 - 2.04)	0.62	0.50
ARB+	CCB/THZ+	0.94	(0.78 - 1.14)	0.52	0.94	(0.78 - 1.14)	0.53	0.00
ARB	CCB	1.14	(0.75 - 1.74)	0.53	1.13	(0.75 - 1.72)	0.56	0.11
ARB+	CCB+	1.08	(0.90 - 1.30)	0.42	1.07	(0.88 - 1.31)	0.50	0.00
ARB	THZ	0.95	(0.42 - 2.15)	0.91	0.99	(0.44 - 2.23)	0.97	0.39
ARB+	THZ+	1.30	(0.98 - 1.72)	0.07	1.32	(0.99 - 1.75)	0.06	0.22

Supplementary Table 215: Risk estimates for pneumonia across propensity score matched, prevalent-user cohorts in the Meta-analysis across data sources. We report uncalibrated and calibrated hazard ratios (HRs) and their 95% confidence intervals (CIs). (+) indicates in-combination cohorts.

Target	Comparator	Uncalibrated HR	Uncalibrated 95% CI	p	Calibrated HR	Calibrated 95% CI	p	i^2
ACE/ARB	CCB/THZ	0.91	(0.75 - 1.11)	0.35	0.92	(0.75 - 1.12)	0.44	0.00
ACE/ARB+	CCB/THZ+	0.96	(0.85 - 1.08)	0.51	0.96	(0.84 - 1.09)	0.53	0.00
ACE/ARB	CCB	0.96	(0.77 - 1.20)	0.74	0.98	(0.76 - 1.26)	0.86	0.00
ACE/ARB+	CCB+	1.11	(0.78 - 1.58)	0.58	1.10	(0.77 - 1.57)	0.60	0.38
ACE/ARB	THZ	0.91	(0.65 - 1.25)	0.55	0.95	(0.69 - 1.32)	0.79	0.00
ACE/ARB+	THZ+	1.11	(0.95 - 1.31)	0.20	1.16	(0.95 - 1.43)	0.14	0.00
ACE	ARB	0.87	(0.67 - 1.13)	0.29	0.87	(0.67 - 1.13)	0.31	0.00
ACE+	ARB+	0.98	(0.88 - 1.09)	0.71	0.99	(0.89 - 1.10)	0.87	0.00
ACE	CCB/THZ	0.93	(0.74 - 1.17)	0.54	0.93	(0.74 - 1.17)	0.57	0.00
ACE+	CCB/THZ+	0.98	(0.87 - 1.10)	0.70	0.98	(0.85 - 1.14)	0.80	0.00
ACE	CCB	0.95	(0.75 - 1.20)	0.66	0.95	(0.73 - 1.24)	0.66	0.00
ACE+	CCB+	1.00	(0.89 - 1.13)	0.96	1.00	(0.89 - 1.13)	0.92	0.00
ACE	THZ	0.93	(0.66 - 1.33)	0.71	0.98	(0.69 - 1.40)	0.91	0.00
ACE+	THZ+	1.02	(0.89 - 1.18)	0.73	1.07	(0.92 - 1.24)	0.37	0.00
ARB	CCB/THZ	1.34	(0.66 - 2.72)	0.42	1.33	(0.66 - 2.71)	0.43	0.40
ARB+	CCB/THZ+	0.93	(0.81 - 1.07)	0.29	0.93	(0.81 - 1.06)	0.32	0.00
ARB	CCB	1.18	(0.87 - 1.60)	0.28	1.17	(0.86 - 1.59)	0.32	0.00
ARB+	CCB+	1.02	(0.68 - 1.51)	0.94	1.01	(0.67 - 1.51)	0.96	0.41
ARB	THZ	0.85	(0.51 - 1.44)	0.55	0.88	(0.52 - 1.49)	0.64	0.08
ARB+	THZ+	1.11	(0.72 - 1.72)	0.64	1.13	(0.73 - 1.75)	0.60	0.25

Supplementary Table 216: Risk estimates for PAAS across propensity score matched, prevalent-user cohorts in the Meta-analysis across data sources. We report uncalibrated and calibrated hazard ratios (HRs) and their 95% confidence intervals (CIs). (+) indicates in-combination cohorts.

		Uncalibrated			Calibrated			
Target	Comparator	HR	95% CI	p	HR	95% CI	p	i^2
ACE/ARB	CCB/THZ	1.14	(0.99 - 1.31)	0.07	1.15	(1.00 - 1.33)	0.06	0.00
ACE/ARB+	CCB/THZ+	1.15	(1.05 - 1.25)	< 0.01	1.14	(1.04 - 1.26)	< 0.01	0.00
ACE/ARB	CCB	1.18	(1.01 - 1.38)	0.04	1.20	(0.98 - 1.46)	0.07	0.00
ACE/ARB+	CCB+	1.29	(0.92 - 1.82)	0.14	1.29	(0.91 - 1.81)	0.15	0.44
ACE/ARB	THZ	1.01	(0.81 - 1.27)	0.90	1.07	(0.86 - 1.34)	0.57	0.00
ACE/ARB+	THZ+	1.05	(0.95 - 1.17)	0.35	1.10	(0.93 - 1.30)	0.24	0.00
ACE	ARB	1.03	(0.86 - 1.23)	0.78	1.03	(0.86 - 1.23)	0.75	0.00
ACE+	ARB+	0.92	(0.62 - 1.36)	0.69	0.93	(0.63 - 1.38)	0.73	0.62
ACE	CCB/THZ	1.12	(0.96 - 1.31)	0.15	1.12	(0.96 - 1.31)	0.17	0.00
ACE+	CCB/THZ+	1.17	(1.08 - 1.27)	< 0.01	1.17	(1.04 - 1.32)	< 0.01	0.00
ACE	CCB	1.15	(0.97 - 1.37)	0.10	1.16	(0.95 - 1.41)	0.12	0.00
ACE+	CCB+	1.00	(0.70 - 1.44)	0.99	1.00	(0.70 - 1.44)	0.97	0.51
ACE	THZ	1.11	(0.87 - 1.40)	0.40	1.16	(0.91 - 1.47)	0.24	0.00
ACE+	THZ+	1.00	(0.91 - 1.10)	1.00	1.05	(0.94 - 1.16)	0.41	0.00
ARB	CCB/THZ	1.13	(0.93 - 1.39)	0.23	1.13	(0.92 - 1.38)	0.26	0.00
ARB+	CCB/THZ+	0.98	(0.89 - 1.07)	0.62	0.98	(0.88 - 1.07)	0.65	0.00
ARB	CCB	1.20	(0.96 - 1.48)	0.10	1.19	(0.95 - 1.47)	0.14	0.00
ARB+	CCB+	1.08	(0.83 - 1.41)	0.55	1.08	(0.82 - 1.42)	0.60	0.29
ARB	THZ	0.87	(0.54 - 1.42)	0.58	0.90	(0.56 - 1.46)	0.68	0.15
ARB+	THZ+	1.11	(0.62 - 1.99)	0.73	1.13	(0.63 - 2.02)	0.69	0.59

Negative control outcomes

We selected negative controls using a process similar to that outlined in Voss et al. [1]. We first constructed a list of all conditions that satisfy the following criteria with respect to all drug exposures in the LEGEND-HTN study [2]:

- No Medline abstract where the MeSH terms suggests a drug-condition association [3],
- No mention of the drug-condition pair on a US product label in the “Adverse Drug Reactions” or “Postmarketing” section [4],
- No US spontaneous reports suggesting that the pair is in an adverse event relationship [5, 6],
- OMOP vocabulary does not suggest that the drug is indicated for the condition,
- Vocabulary conditional concepts are usable (i.e., not too broad, not suggestive of an adverse event relationship, no pregnancy related), and
- Exact condition concept itself is used in patient level data.

We then optimized the remaining condition concepts, such that parent concepts remove children as defined by the OMOP vocabulary and performed manual review to exclude any pairs that may still be in a causal relationship or too similar to the study outcome. This process led to a candidate list of 123 negative controls:

Supplementary Table 217: Negative control outcomes.

Condition concept
Abnormal cervical smear
Abnormal pupil
Abrasion and/or friction burn of trunk without infection
Absence of breast
Absent kidney
Acariasis
Acid reflux
Acquired hallux valgus
Acquired keratoderma
Acquired trigger finger
Acute conjunctivitis
Amputated foot
Amyloidosis
Anal and rectal polyp
Ankylosing spondylitis
Aseptic necrosis of bone
Astigmatism
Bell’s palsy
Benign epithelial neoplasm of skin
Burn of forearm
Calcaneal spur
Cannabis abuse
Cervical somatic dysfunction
Chalazion
Changes in skin texture
Chondromalacia
Chondromalacia of patella
Cocaine abuse
Colostomy present
Complication due to Crohn’s disease
Contact dermatitis
Contusion of knee
Crohn’s disease
Croup
Derangement of knee
Difficulty sleeping
Disorder of eye due to diabetes mellitus
Disproportion of reconstructed breast
Effects of hunger
Endocarditis
Endometrial hyperplasia
Endometriosis
Enthesopathy

Epicondylitis
Epidermoid cyst
Epstein-Barr virus disease
Feces contents abnormal
Foreign body in orifice
Fracture of upper limb
Gallstone
Ganglion cyst
Genetic predisposition
Genital herpes simplex
Hammer toe
Hemangioma
Hereditary thrombophilia
Herpes zoster without complication
High risk sexual behavior
Hodgkin's disease
Homocystinuria
Human papilloma virus infection
Hypoglycemic coma
Hypopituitarism
Ileostomy present
Impacted cerumen
Impetigo
Impingement syndrome of shoulder region
Ingrowing nail
Injury of knee
Iridocyclitis
Irregular periods
Irritable bowel syndrome
Kwashiorkor
Late effect of contusion
Late effect of motor vehicle accident
Lesion of cervix
Leukorrhea
Lyme disease
Macular drusen
Malignant neoplasm of endocrine gland
Melena
Mononeuropathy
Nicotine dependence
Noise effects on inner ear
Nonspecific tuberculin test reaction
Non-toxic multinodular goiter
Onychomycosis
Onychomycosis due to dermatophyte

Opioid abuse
Osteochondropathy
Paraplegia
Passing flatus
Polyp of intestine
Postviral fatigue syndrome
Presbyopia
Problem related to lifestyle
Psychalgia
Ptotic breast
Pulmonary tuberculosis
Rectal mass
Regular astigmatism
Sarcoidosis
Scar
Senile hyperkeratosis
Septic shock
Sjögren’s syndrome
Somatic dysfunction of lumbar region
Splinter of face, without major open wound
Sprain of ankle
Strain of rotator cuff capsule
Tear film insufficiency
Tietze’s disease
Tobacco dependence syndrome
Tonsillitis
Toxic goiter
Ulcerative colitis
Vaginitis and vulvovaginitis
Verruca vulgaris
Viral conjunctivitis
Viral hepatitis
Visceroptosis
Wristdrop
Wrist joint pain

References

[1] Voss EA, Boyce RD, Ryan PB, van der Lei J, Rijnbeek PR, Schuemie MJ. Accuracy of an automated knowledge base for identifying drug adverse reactions. J Biomed Inform. 2017;66:72–81.

[2] Suchard MA, Schuemie MJ, Krumholz HM, You SC, Chen R, Pratt N, et al. Comprehensive comparative effectiveness and safety of first-line antihypertensive drug classes: a systematic, multinational, large-scale analysis. The Lancet. 2019;394(10211):1816–1826.

[3] Winnenburg R, Sorbello A, Ripple A, Harpaz R, Tonning J, Szarfman A, et al. Leveraging MEDLINE indexing for pharmacovigilance–Inherent limitations and mitigation strategies. Journal of Biomedical Informatics. 2015;57:425–435.

[4] Duke J, Friedlin J, Li X. Consistency in the safety labeling of bioequivalent medications. Pharmacoepidemiology and Drug Safety. 2013;22(3):294–301.

[5] Evans S, Waller PC, Davis S. Use of proportional reporting ratios (PRRs) for signal generation from spontaneous adverse drug reaction reports. Pharmacoepidemiology and Drug Safety. 2001;10(6):483–486.

[6] Banda JM, Evans L, Vanguri RS, Tatonetti NP, Ryan PB, Shah NH. A curated and standardized adverse drug event resource to accelerate drug safety research. Scientific data. 2016;3:160026.
